# Disease and Medication Context Shape Ex Vivo Metabolite Stability: A Pilot Study in Systemic Lupus Erythematosus

**DOI:** 10.3390/metabo15110738

**Published:** 2025-11-12

**Authors:** Fabian Schmitt, Susanne Nguyen, Paul Christoph Claßen, Myriam Meineck, Mathias Hagen, Julia Weinmann-Menke, Thierry Schmidlin

**Affiliations:** 1 Institute of Immunology, University Medical Center, Johannes Gutenberg University Mainz, 55131 Mainz, Germany; schmittf@uni-mainz.de (F.S.); nguyensu@uni-mainz.de (S.N.); hagen@uni-mainz.de (M.H.); 2 Department of Internal Medicine I, University Medical Center, Johannes Gutenberg University Mainz, 55131 Mainz, Germany; classenp@uni-mainz.de (P.C.C.); myriam.meineck@unimedizin-mainz.de (M.M.); julia.weinmann-menke@unimedizin-mainz.de (J.W.-M.); 3 Research Center for Immunotherapy (FZI), University Medical Center, Johannes-Gutenberg University Mainz, 55131 Mainz, Germany; 4 German Center for Cardiovascular Research (DZHK), Partner Site Rhine Main, 55131 Mainz, Germany

**Keywords:** metabolomics, pre-analytical variation, metabolite stability, systemic lupus erythematosus, medication effects, LC-MS, biobanking, serum

## Abstract

**Background/Objectives**: Pre-analytical variation is a major challenge in metabolomics, yet most stability studies have focused on healthy volunteers and have overlooked the impact of disease and medication. To address this gap, we conducted a pilot study in systemic lupus erythematosus (SLE) to assess serum metabolite stability under delayed centrifugation. **Methods**: Peripheral blood from 10 SLE patients and 5 healthy controls (HC) was stored at room temperature for 1–24 h before processing and analyzed by untargeted LC-MS-based metabolomics. This design enabled direct evaluation of the effect of pre-analytical delay within the context of clinical heterogeneity. **Results**: Principal component trajectories showed reproducible temporal shifts in HC but dispersed patterns in SLE, indicating disease- and treatment-related influences. Linear mixed-effects models identified metabolites with condition-specific kinetics, including glucose, choline, glycerophosphocholine, and pyroglutamic acid. Mycophenolate intake was further associated with distinct AMP dynamics. **Conclusions**: These findings demonstrate that both disease state and medication reshape apparent metabolite stability, highlighting the need for strictly controlled sample handling and well-characterized clinical cohorts in metabolomics studies.

## 1. Introduction

Metabolomics laboratories and facilities are increasingly approached to analyze large-scale pre-collected patient cohorts and biobank samples [1,2,3,4]. While such studies hold enormous potential, they are also particularly vulnerable to pre-analytical variation [5,6,7,8,9,10,11]. Several reports have already shown that even subtle differences in sample handling can alter metabolite profiles, with most prior work focusing on healthy volunteers or animals [12,13]. These studies generally tend to either caution from embarking on cohort analysis with poor pre-analytics at all [14] or aim to establish stability metrics to identify robust metabolites within the confines of a given, potentially suboptimal pre-analytical condition [15]. This approach is valuable, yet it risks oversimplification; disease status and medication use, two defining characteristics of clinical cohorts, remain largely overlooked in the assessment of metabolite stability. Ignoring these factors may lead to misinterpretation of metabolic signatures, confounding biological findings with pre-analytical artifacts.

In systemic lupus erythematosus (SLE), a complex autoimmune disease characterized by metabolic heterogeneity and broad medication exposure, these issues are particularly pronounced [16,17]. Here, we present a small pilot study we performed in preparation of setting up a large-scale SLE cohort, where we specifically evaluated the ex vivo stability of serum metabolites under delayed serum centrifugation in both healthy control (HC) and SLE patients. Our goal was to investigate whether notions of ex vivo metabolite stability signatures hold true in the context of samples affected by underlying SLE and medication.

## 2. Materials and Methods

### 2.1. Chemicals and Reagents

All solvents used in this study were LC-MS grade. Water (H_2_O, Cat. No. 701074), acetonitrile (ACN—Cat. No. 701881), and methanol (MeOH—Cat. No. 701091) were purchased from PanReac AppliChem GmbH (Darmstadt, Germany). Formic acid (FA) and NIST^®^ SRM^®^ 1950 “metabolites in human plasma” were obtained from Merck KGaA (Darmstadt, Germany). The Kinetex F5 column (2.6 µm, 100 Å, 150 × 2.1 mm) was obtained from Phenomenex (Aschaffenburg, Germany), and the S-Monovette^®^ Serum Gel tubes for blood collection were purchased from Sarstedt (Cat. No. 04.1925.001, Nümbrecht, Gemany).

### 2.2. Study Design and Serum Draw

Peripheral blood samples were collected from 10 SLE patients during routine outpatient clinic visits and from 5 healthy volunteers. Venipuncture was performed by a qualified nurse using S-Monovette^®^ Serum Gel tubes. All collections were conducted between 09:25 h (earliest) and 13:10 h (latest). For each participant, tubes were kept at room temperature for six predefined pre-centrifugation delay times (1 h, 2 h, 3 h, 4 h, 8 h, and 24 h). Subsequently, samples were centrifuged at 3000 rpm for 5 min at room temperature, and the resulting serum was stored at −80 °C until further processing.

### 2.3. Automated Metabolite Extraction

Prior to metabolite extraction, serum samples were thawed at 4 °C. For each sample, 25 µL of serum were transferred into a 96-well plate. All extraction steps were performed on a Tecan Freedom EVO 100 liquid handling platform (Tecan, Männedorf, Switzerland) as previously described [18,19,20,21]. Briefly, each sample was diluted with 45 µL of LC-MS-grade water precooled to 4 °C, followed by addition of 210 µL of LC-MS-grade MeOH precooled to −20 °C. Samples were subsequently shaken at 1000 rpm for 5 min at 4 °C, followed by incubation at −20 °C for 2 h. Protein precipitates were pelleted by centrifugation at 6000× *g* for 30 min at 4 °C. A 150 µL aliquot of each supernatant was transferred into a new 96-well plate and dried under a constant nitrogen flow. Precipitates were reconstituted in 100 µL of water and subsequently shaken at 300 rpm for 5 min at 4 °C. Each sample was then split into three aliquots. QC samples were prepared by pooling 2 µL of each individual sample. NIST^®^ SRM^®^ 1950 reference material (Merck KGaA, Darmstadt, Germany) was prepared using the same protocol manually in 1.5 mL Eppendorf tubes.

### 2.4. LC-MS Analysis

LC-MS analysis was performed on an Agilent 1290 Infinity II UHPLC system (Agilent Technologies, Waldbronn, Germany) coupled to a Sciex ZenoTOF 7600 mass spectrometer (Sciex, Darmstadt, Germany) equipped with an OptiFlow electrospray ionization (ESI) source. All samples were analyzed in positive ion mode.

Reversed-phase (RP) chromatographic separation of metabolites was carried out on a Kinetex F5 column (2.6 µm, 100 Å, 150 × 2.1 mm, Phenomenex, Aschaffenburg, Germany). The mobile phase consisted of water (H_2_O) with 0.1% formic acid (FA) as solvent A and acetonitrile (ACN) with 0.1% FA as solvent B. A 1 µL aliquot of each metabolite extract was injected and loaded onto the column using mobile phase A at eject speed 400 µL/min.

Chromatographic separation was achieved using an 11 min LC method starting with 0% B at 200 µL/min and held until 2.1 min, followed by a linear increase to 95% B until 6.5 min, which was maintained until 8.0 min. At 8.0 min, the flow was switched to offline mode via an external post-column valve, and the flowrate was increased linearly to 1 mL/min until 8.15 min, then maintained until 9.15 min. The solvent composition was subsequently changed to 0% B until 9.30 min and held until 10.30 min. Subsequently, the flowrate was decreased to 200 µL/min until 10.45 min and maintained until 11 min for pressure stabilization and re-equilibration of the column.

The column compartment temperature was maintained at 50 °C and the multisampler temperature was kept at 4 °C throughout the analytical sequence.

Ion source and gas parameters were as follows: ion source gas 1 and 2 were set to 45 psi, the curtain gas to 35 psi, and the collisionally activated dissociation (CAD) gas to 7. The ion source temperature was maintained at 400 °C, and the spray voltage was set to 5000 V.

All samples were analyzed in data-dependent acquisition (DDA) mode. Time-of-flight (TOF) MS survey scans were acquired with an accumulation time of 100 ms over a mass range of 70–1000 *m*/*z*, using a declustering potential (DP) of 80 V and a collision energy (CE) of 10 V without CE spread. Information-dependent acquisition (IDA) criteria parameters were set to select the TOP 11 most intense precursor ions for fragmentation, using an intensity threshold of 100 cps, a dynamic exclusion (DE) of 6 s after 2 occurrences, and dynamic background subtraction enabled. TOF MS/MS spectra for selected precursors were acquired over a mass range of 40–1000 *m*/*z*, using an accumulation time of 25 ms, and collision-induced dissociation (CID) with a CE of 35 V and a CE spread of 15 V. Zeno pulsing was enabled with Zeno threshold set to 20,000 cps.

All study samples were processed and analyzed in a single experimental batch. Batch design included samples, processing blanks, solvent blanks, and multiple QC samples. At the beginning of the batch, two solvent blank samples were analyzed followed by a processing blank, followed by five pooled QC samples for column conditioning. Three NIST^®^ SRM^®^ 1950 samples were subsequently injected to verify system suitability. Subsequent sample analysis followed random order. After every 14 samples, an additional pooled QC injection was performed, followed by in-batch calibration using the X500 calibration solution. In-batch calibration maintained a TOF resolution of 33,000–38,000 across the 70–1000 *m*/*z* range for MS1 and 28,000–38,000 for MS2, ensuring a mass accuracy better than 5 ppm throughout data acquisition. Every calibration was followed by a NIST^®^ SRM^®^ 1950 injection. Eventually the batch was concluded with the injection of a processing blank followed by a solvent blank.

### 2.5. Metabolite Annotation

Raw .wiff2 files were converted into centroid .mzML files using MSConvert prior to processing in MS-DIAL (ver. 5.5.250221, Tokyo University of Agriculture and Technology, Yokohama, Japan) [22]. Metabolite annotation used untargeted spectral matching against both an in-house spectral library previously described in Schmitt et al. [18] and the public ESI(+)-MS/MS library of authentic standards. Mass tolerances were set to 0.01 Da and 0.025 Da for MS1 and MS2, respectively, with the MS1 mass error distribution of annotated features shown in Figure S1. Peak detection was performed using a minimum peak amplitude of 500 and a mass slice width of 0.1 Da. Peak smoothing was applied using a linear weighted moving average method with a smoothing level of three scans and a minimum peak width of five scans. Adduct detection included [M + H]^+^, [M + NH_4_]^+^, [M + Na]^+^, [M + K]^+^, [M + H–H_2_O]^+^, [M + H–2H_2_O]^+^, and [2M + Na]^+^. Peak alignment was carried out with a retention time (RT) tolerance of 0.2 min and an MS1 tolerance of 0.015 Da. All matched metabolites were manually curated based on match scores, visual assessment of the spectral match, and, for in-house library matches, RT consistency. Over-annotated features were relabeled accordingly; both the original annotations and curated annotation are provided in Table S1. In parallel, metabolite annotation was performed in SIRIUS [23] and selected high-confidence annotation were used to further annotate MS-DIAL features. Automated feature integration of MS-DIAL was further manually reviewed and adjusted for all annotated metabolites. Area under the curve (AUC) values were exported as quantitative measures of metabolite abundances for downstream statistical analysis.

### 2.6. Statistical Analysis

All statistical analyses were performed in R (v4.3.1, R Foundation for Statistical Computing, Vienna, Austria) using the tidyverse, lme4, lmerTest, emmeans, pwr, and performance packages.

#### 2.6.1. Quality Control Filtering

No batch correction or normalization was applied to the metabolite abundances prior to statistical analysis. Instead, analytical precision was assessed from repeated injections of pooled QC samples. For each metabolite, the coefficient of variation (CV) was calculated, and metabolites with CV < 25% were retained for primary statistical analyses. In addition, for contextual interpretation of hypoxanthine metabolism, inosine was visualized despite not meeting this threshold (CV = 85.6%).

#### 2.6.2. Baseline Analyses

Principal component analysis (PCA) was performed on T1 (1 h) samples to visualize overall separation of SLE and healthy control (HC) samples. Univariate group comparisons at T1 were conducted by Welch’s *t*-test, with Benjamini–Hochberg false discovery rate (FDR) correction. Volcano plots were used to highlight differentially abundant metabolites. Outlier samples were assessed at T1 by two complementary approaches: robust z-score (fraction of metabolites deviating >3 SD within group) and robust Mahalanobis distance in PCA space.

#### 2.6.3. Ex Vivo Stability Modeling

For each metabolite with CV < 25%, linear mixed-effects (LME) models were fitted with fixed effects of condition (SLE vs. HC), time (T1–T6), and their interaction, and a random intercept for patient ID. Models were evaluated by marginal and conditional R^2^ (variance explained by fixed effects vs. fixed + random effects). Type III ANOVA was used to test the significance of main and interaction terms, with FDR adjustment across metabolites. Estimated marginal means (EMMs) were computed per condition and time point and visualized for selected metabolites.

#### 2.6.4. Visualization of Ex Vivo Stability Effects

Global ex vivo stability effects were visualized by PCA trajectories (samples connected across time). Per-metabolite stability was visualized using (i) R^2^ scatter plots (marginal vs. conditional) and (ii) EMM trajectories for metabolites.

#### 2.6.5. Drug-Related Analyses

To explore treatment effects, drug exposure metadata was integrated at the patient level. At baseline (T1), SLE patients were stratified by drug exposure (e.g., prednisolone, hydroxychloroquine (HCQ), and mycophenolate). For each metabolite, group differences were tested using Welch’s t-test and Wilcoxon rank-sum test, with effect sizes estimated by Hedges’ g and FDR correction per drug. These exploratory analyses considered all detected metabolites to avoid missing potential drug-related features, including xenobiotics. In contrast, longitudinal analyses were restricted to metabolites meeting the QC criterion of CV < 25% in pooled QC samples. LME models restricted to SLE samples tested for Time × Drug interactions, with drug exposure encoded as a binary factor. Interaction *p*-values were FDR-adjusted per drug. Significant or nominal associations were further visualized by EMM trajectories, highlighting drug-positive and drug-negative subgroups.

#### 2.6.6. Power Analysis

To evaluate the sensitivity of our study to detect metabolite differences between SLE patients (*n* = 8) and healthy controls (*n* = 5) at T1, we performed post hoc power calculations. Statistical power was estimated across a range of standardized effect sizes (Cohen’s *d* = 0.2–1.2) for two-sample unpaired t-tests with unequal group sizes, using a significance threshold of α = 0.05. The analysis identified the minimum effect size detectable with 80% power and plotted power curves illustrating the relationship between effect size and statistical power. Labels for the 80% power threshold and maximum achieved power were added to aid interpretation (Figure S2).

### 2.7. Visualization and Presentation

Visualization was performed in R and with the assistance of BioRender (Toronto, ON, Canada, http://www.biorender.com, accessed on 8 September 2025). During the preparation of this manuscript, the authors used AI-based tools (GPT-5, OpenAI, San Francisco, CA, USA and Claude Sonnet 4, Anthropic, San Francisco, CA, USA) to assist with repetitive code formatting, metabolite name harmonization, and language refinement. These tools were used strictly for supportive purposes under full author supervision. All analyses, data interpretation, and conclusions were produced, verified, and approved by the authors.

## 3. Results

### 3.1. Study Design and Experimental Context

**Figure 1:**
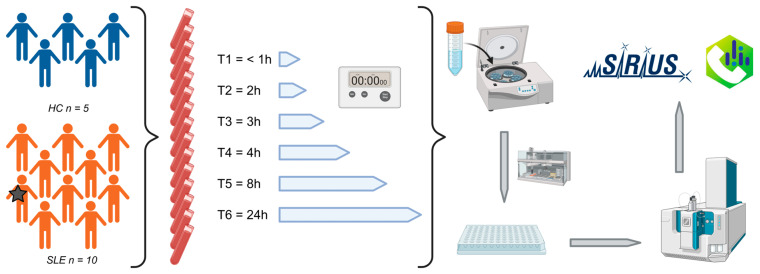
Overview of study design and analytical workflow. Peripheral blood from 5 healthy controls (HC, blue) and 10 systemic lupus erythematosus (SLE, orange) patients was collected into S-Monovette^®^ Serum Gel tubes and held at room temperature for six predefined pre-centrifugation delays (1–24 h) before serum isolation and storage at −80 °C. Metabolites were extracted by automated methanol precipitation, analyzed by untargeted LC–MS, and annotated using MS-DIAL and SIRIUS. The star marks the patient sampled during an acute disease flare (SLE02).

In this study we set out to investigate the ex vivo stability of serum metabolites under delayed centrifugation comparing SLE patients under different medication regimens and HC. Reflecting common approaches in biobanking strategies, we collected peripheral blood of individuals into S-Monovette^®^ Serum Gel tubes and subjected them to six different defined pre-centrifugation delays at room temperature: 1 h, 2 h, 3 h, 4 h, 8 h, and 24 h (Figure 1). Our pilot cohort contained 5 healthy volunteers and 10 SLE patients. Baseline characteristics of patients and controls are summarized in Table 1. One patient (SLE02) presented during an acute disease activity increase and was receiving tacrolimus, while all others presented during routine visitation. SLE patients were older than healthy controls (48 ± 16 vs. 33 ± 5 years, *p* = 0.043) and predominantly female (90%). At the time of sampling, disease activity was low (mean SLE Disease Activity Index 2000 (SLEDAI-2k) 2.6 ± 2.1; mean Physician Global Assessment (PGA) score 0.8 ± 0.6), with 60% of patients in Lupus Low Disease Activity State (LLDAS) and 20% fulfilling Definitions of Remission in SLE (DORIS) remission criteria. Complement consumption was common (low C3c observed in 30%; low C4 observed in 60%) in this patient group. Antiphospholipid antibodies were present in 40%. All participants were of Caucasian ethnicity.

### 3.2. Analytical Pipeline and Feature Filtering

All samples were subjected to methanol-based metabolite extraction and were subsequently analyzed by untargeted LC-MS-based metabolomics. In total, 108 distinct metabolites represented by 113 features (including 5 pairs of different adducts) were included in the analysis, covering diverse biochemical classes such as amino acids, acylcarnitines, organic acids, lipids, and nucleosides (Table S1).

Prior to statistical analysis, we ranked metabolites according to their technical variance observed in repeated injections of pooled sample quality controls (QCs). A total of 58 metabolites with a coefficient of variation (CV) < 25% were included in the main statistical analyses, while 50 higher-CV metabolites were retained for qualitative interpretation (Table S2).

Initially, we specifically explored the detectability of medication-related features. Prednisolone and hydroxychloroquine (HCQ) were directly detected in the serum of SLE patients with documented intake. Similarly, mycophenolic acid (MPA), the active compound in CellCept and Myfortic, was also detected. Two distinct features were annotated as MPA, both with high MS/MS spectral similarity but different RTs (6.2 min and 7.1 min).

### 3.3. Baseline Metabolic Differences in SLE vs. HC

**Figure 2:**
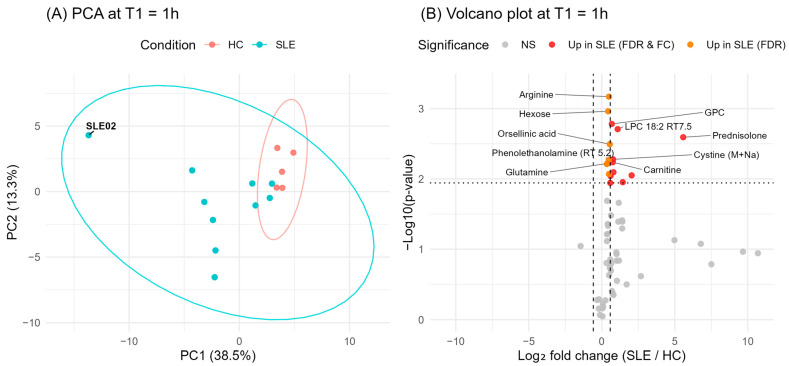
Baseline metabolic differences between SLE patients and HC at T1. (**A**) Principal component analysis (PCA) of serum metabolite profiles at T1. (**B**) Volcano plot of SLE vs. HC at T1. Dots represent individual metabolites; features significantly different between groups after FDR correction are shown in orange, with those exhibiting a log_2_ fold change > 0.58 highlighted in red.

Next, we set out to establish a robust baseline for the ex vivo stability analyses by comparing metabolite abundances between HC and SLE patients at the earliest available processing time point T1, 1 h after collection. The baseline comparison was intended to capture pre-existing metabolic differences between HC and SLE, as they could influence and confound the interpretation of the subsequent ex vivo stability trends. For visualization we performed principal component analysis (PCA) on all T1 measurements. This unsupervised view provides a compact summary of global variance indicating whether HC and SLE already separate before ex vivo changes occur. We further complemented this with a T1 volcano plot (SLE vs. HC) to highlight the specific metabolites that most strongly contribute to the baseline separation (Figure 2, Figure S3 and Table S3).

The PCA at T1 revealed relatively compact clustering of HC samples, whereas SLE samples showed greater dispersion. One SLE patient (SLE02) displayed a great distance from both the HC cluster and the other SLE samples, indicative of an outlier profile. The volcano plot of SLE vs. HC at T1 showed that most differences were due to higher metabolite levels in SLE. These metabolites included the corticosteroid prednisolone and several lipid-related metabolites such as lysophosphatidylcholine (18:2) (LPC 18:2) and glycerophosphocholine (GPC). We further observed elevated levels of the amino acids arginine, glutamine (together with its in-source fragment pyroglutamic acid), cystine, and hydroxyproline and the urea-cycle-related metabolite citrulline in SLE compared to HC. Post hoc power calculations were performed for the T1 comparison between SLE (*n* = 8) and HC (*n* = 5). The analysis estimated statistical power across medium effect sizes (Cohen’s *d* = 0.5) and indicated that the achieved power was below 0.8, with a maximum power of 0.53 at *d* = 1.2 (Figure S2).

### 3.4. Global Effects of Delayed Centrifugation: PCA Trajectories

Before proceeding to the ex vivo stability analyses, we addressed outliers that could confound the interpretation of temporal trends. We decided not to include data from patient SLE02 in the systematic stability analysis, as SLE02 appeared as the most extreme case in PCA. This was further supported by two complementary, data-driven analyses we performed on the T1 dataset: (1) Mahalanobis distances in the multivariate metabolite space, capturing global deviations from the group centroid (Figure S4, Table S4), and (2) a robust z-score analysis within each condition, quantifying the fraction of metabolites deviating by more than 3 standard deviations from the group median (Figure S5, Table S4). Both approaches consistently identified patient SLE02 as a pronounced outlier, with deviation metrics exceeding those of all other patients, resulting in exclusion of the patient from subsequent analysis. In addition, patient SLE01 was excluded due to a technical error affecting the T3 measurement, which prevented complete temporal profiling for this individual in the PCA. This left us with *n* = 5 HC samples and *n* = 8 SLE samples for the subsequent statistical analysis.

**Figure 3:**
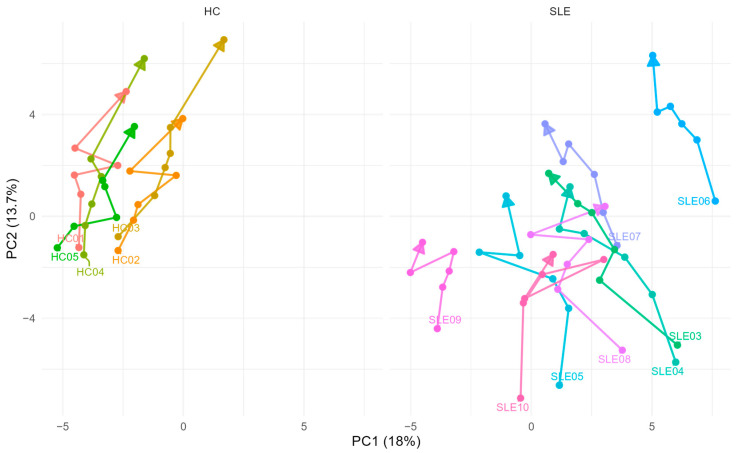
Global effects of delayed centrifugation on serum metabolite profiles. PCA trajectories of SLE patients and HC from T1 to T6. Each line connects repeated measurements from the same individual across the six pre-centrifugation delay times.

We first investigated ex vivo metabolite stability over time by PCA analysis to visually assess whether temporal movement in multivariate change upon centrifugation delay differs between HC and SLE. Figure 3 displays the updated PCA plot, with scores connected from T = 1 h to T = 24 h for each patient. HC samples exhibited more consistent shifts along PC2, whereas SLE trajectories showed greater dispersion.

### 3.5. Individual Metabolite Stability Patterns Assessed by Linear Mixed-Effects Modeling

**Figure 4:**
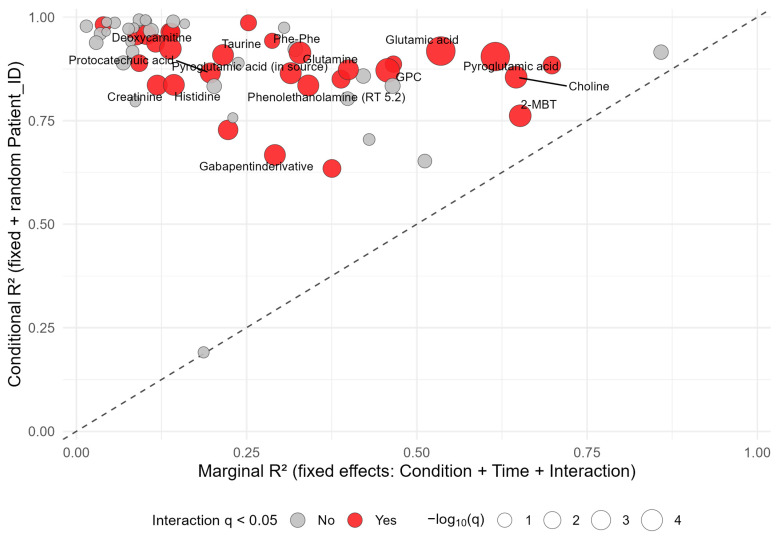
Relationship between marginal and conditional R^2^ values from per-metabolite linear mixed-effects models of serum metabolite stability during delayed centrifugation. Each point represents one metabolite; point size encodes the strength of the condition × time interaction (−log_10_ q-value), and color indicates statistical significance (red, q < 0.05). The dotted line marks equality between marginal and conditional R^2^.

To systematically assess the relative contributions of disease condition and individual variability to metabolite ex vivo stability profiles, we compared marginal (fixed effects only) and conditional (fixed and random effects) R^2^ values from linear mixed-effects models (Figure 4, Table S5). Most metabolites exhibited substantially higher conditional than marginal R^2^ values, indicating strong inter-individual differences in ex vivo metabolite abundance trajectories. A subset of metabolites showed both high marginal R^2^ and significant condition × time interactions (q < 0.05). These metabolites included for instance choline and pyroglutamic acid. Other metabolites such as histidine and betaine were characterized by high conditional but low marginal R^2^ despite significant interactions (q < 0.05).

**Figure 5:**
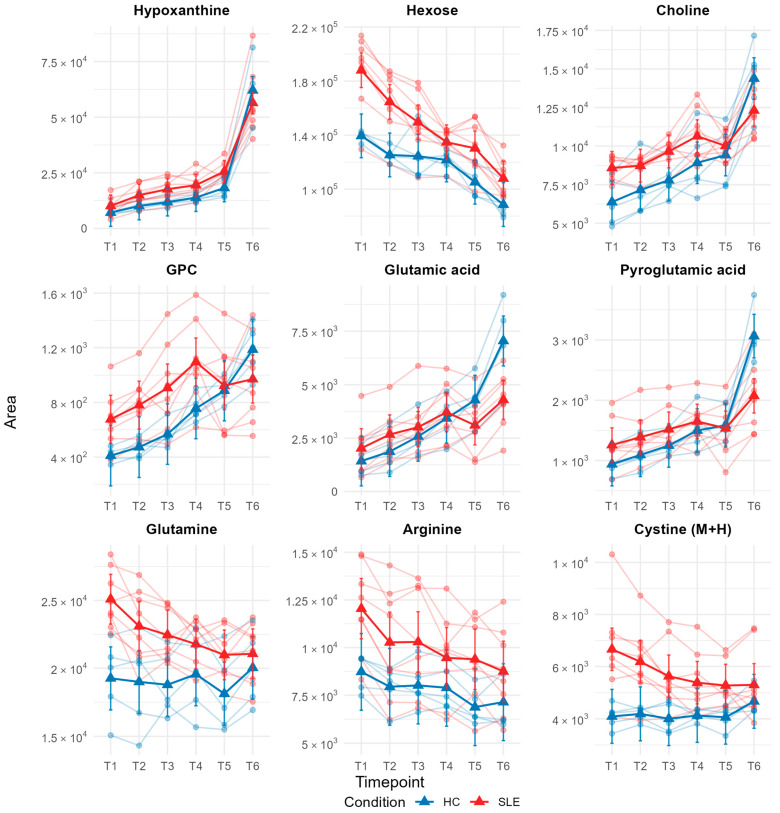
Estimated marginal means (EMMs) over time for selected metabolites (marginal R^2^ ≥ 0.30) from linear mixed-effects models (blue = healthy controls (HC); red = systemic lupus erythematosus (SLE)). Each panel shows individual trajectories (thin lines) and condition-specific EMMs (solid lines with triangles) with 95% confidence intervals. *Y*-axes are scaled individually per metabolite.

Building on our R^2^-based ranking, we next focused on the set of metabolites with high explained variance from fixed effects (marginal R^2^ ≥ 0.30) while restricting the list to common endogenous compounds. This yielded nine candidates, for which we visualized estimated marginal means from the mixed-effects models across all time points (Figure 5—LME plots for all other metabolites are available in Figure S6). These profiles highlight metabolites with reproducible, time-dependent differences between conditions.

Hypoxanthine showed the highest marginal R^2^ (0.86) across all metabolites and a q-value of 0.08, consistent with a strong time-dependent effect that is largely independent of patient condition.

Hexose shows a decline over time but with a steeper drop in SLE. The marginal R^2^ (0.70) indicates that the condition and time effects explain most of the variance, with a significant condition × time interaction (q = 0.0047).

Choline and GPC both showed markedly increased levels over time in HC and SLE. For choline, the strong marginal R^2^ (0.65) and highly significant interaction (q = 3 × 10^−5^) indicate a robust, condition-specific pattern. GPC likewise exhibited a high marginal R^2^ (0.46) and strong interaction (q = 1.75 × 10^−6^), supporting a reliable group difference in temporal behavior. Both metabolites were more abundant in SLE than HC at baseline, yet their ex vivo accumulation rates in SLE were substantially slower, eventually leading to a reversed abundance pattern at T6 (24 h).

Glutamic and pyroglutamic acid both show a slightly elevated starting concentration in HC over SLE. During room temperature delay, both metabolites increase in both groups, but the increase is consistently steeper in HC. By the final time point (T6, 24 h), HC overtakes SLE, yielding a clear condition × time crossover. Both metabolites show high marginal R^2^ values of 0.54 for glutamic acid and 0.62 for pyroglutamic acid, respectively, as well as strong interaction values (both q-values ~10^−10^).

Glutamine levels were substantially elevated in SLE compared to HC at baseline. Over the room temperature delay, glutamine clearly declines in SLE, whereas concentrations in HC remain comparatively stable. In the mixed-effects model, the marginal R^2^ is ~0.40 with a significant condition × time interaction (q ~ 3 × 10^−4^), indicating a reproducible, condition-specific trajectory. Of note, while glutamine concentrations seem to converge for HC and SLE, they did not fully equalize.

Arginine displays a slight decrease over time in both groups, though at consistently higher levels in SLE. The marginal R^2^ is 0.32 and the interaction is not significant (q ~ 0.067).

Cystine showed a clear condition × time effect (marginal R^2^ = 0.389; conditional R^2^ = 0.850; q = 0.0032). At baseline (T1), SLE serum contained more cystine than HC. Over the ex vivo hold, HC rose slightly while SLE declined modestly, increasingly converging over the 24 h delay.

An interesting pattern emerged for the five carnitine-related metabolites in our panel (Figure S6). Although their marginal R^2^ values are modest (0.09–0.22), indicating that inter-individual variability dominates over main effects, four out of five exhibit a significant condition × time interaction (deoxycarnitine: q = 4.1 × 10^−5^; carnitine: q = 5.4 × 10^−4^; hydroxydecanoylcarnitine: q = 0.0032; acetylcarnitine: q = 0.0050), reflecting a slight decline in SLE over time versus near-flat or mildly rising trajectories in HC. Medium-chain acylcarnitine (C10) follows the same trend but does not reach significance (q = 0.06).

Several other metabolites met the false discovery rate (FDR) threshold for interaction despite modest marginal R^2^ (taurine, histidine, creatinine, betaine, citrulline, and paraxanthine).

### 3.6. Effects of Medication on Metabolite Ex Vivo Stability

We further investigated whether we could observe drug-intake-associated effects on metabolite ex vivo decay. For this exploratory baseline analysis, all detected metabolites were considered, including drug-related signals and features not passing QC CV < 25%. When stratifying SLE patients at baseline (T1) by medication exposure, no metabolite differences remained significant after correction for multiple testing. It is noteworthy, though, that several metabolites showed nominal associations with treatment exposure in Welch’s t-test. These include signals for the drug itself or its adducts, such as prednisolone being upregulated upon prednisolone intake (Welch *p* = 0.007), HCQ being upregulated upon HCQ intake (Welch *p* = 0.003), and one of the two features annotated as MPA being upregulated upon mycophenolate intake (Welch *p* = 0.005). Another example is histidine, which at T1 showed nominal association to prednisolone intake (Welch *p* = 0.004, down by approximately 20–25% upon drug intake, Table S6).

**Figure 6:**
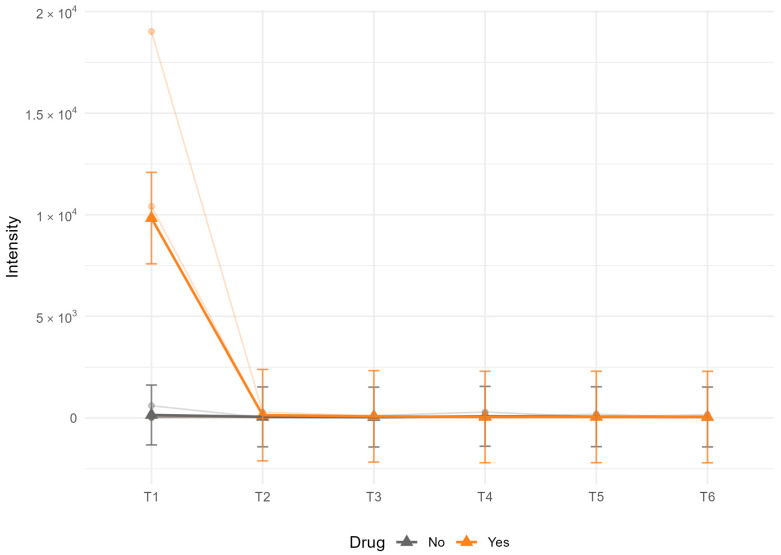
Effect of mycophenolate treatment on AMP stability during delayed centrifugation. Estimated marginal means (EMMs) from linear mixed-effects models showing serum AMP trajectories over time (1–24 h) in systemic lupus erythematosus (SLE) patients stratified by mycophenolate exposure. Error bars represent 95% confidence intervals for the EMMs.

In contrast to baseline stratification, linear mixed models assessing drug-by-time interactions of QC-passed metabolites (CV < 25%) revealed a significant association between mycophenolate (CellCept/Myfortic) exposure and AMP dynamics (q = 0.00067, Figure 6). Patients receiving mycophenolate showed distinct temporal AMP trajectories compared with untreated patients, while no other drug class yielded FDR-significant interactions. Several additional drug–metabolite pairs exhibited nominal drug-by-time effects (LME *p* < 0.05), including adenosine in patients receiving SGLT2 inhibitor or prednisolone and cholate in patients receiving HCQ, but they did not survive multiple testing adjustment (Figure S7, Table S7).

## 4. Discussion

### 4.1. Study Overview and Key Findings

This pilot study aimed to characterize the ex vivo stability of serum metabolites under delayed centrifugation, comparing SLE patients with HC. The study design reflected conditions commonly encountered in biobanking workflows, with defined pre-centrifugation delays ranging from 1 to 24 h. We applied untargeted LC-MS-based metabolomics and used 58 quantitatively robust metabolites (QC CV < 25%) to assess both technical and biological variability across these time points and modeled temporal behavior using linear mixed-effects analysis.

The approach enabled simultaneous assessment of overall temporal drift, inter-individual variability, and potential condition-dependent effects. As expected, inter-individual differences accounted for a large proportion of overall variance across metabolites, reflected by generally higher conditional than marginal R^2^ values. Nevertheless, several metabolites exhibited comparatively high marginal R^2^, indicating reproducible time- and condition-related trends beyond individual variability. Among these, a smaller subset additionally exhibited significant condition × time interactions, indicating distinct ex vivo stability patterns between SLE and HC. In addition, exploratory analyses considered potential influences of medication exposure on apparent metabolite stability.

Previous studies have systematically examined pre-analytical effects in metabolomics, mostly using plasma or serum from healthy volunteers. Reviews by Stevens et al. 2019 and Yin et al. 2015 highlighted that processing delays, temperature, and freeze–thaw cycles markedly alter metabolite concentrations, underscoring the need for standardized handling protocols [13,24]. These studies, however, addressed methodological reproducibility rather than disease-specific influences. The present study therefore adds a novel pathophysiological perspective, showing that pre-analytical metabolite stability can be modulated by disease- and drug-related factors in SLE. This integration of methodological and biological dimensions fills an important gap between controlled laboratory assessments and the complex reality of clinical metabolomics.

### 4.2. Methodological Considerations and Analytical Framework

The present work was designed as a small-scale pilot study to evaluate ex vivo metabolite stability under controlled pre-centrifugation delays. The cohort comprised 10 SLE patients and 5 healthy controls, which allowed for paired temporal profiling but limits the generalizability of quantitative conclusions. Despite the modest sample size, the data provide a structured proof of concept for modeling time-dependent metabolite behavior in clinically heterogeneous cohorts. The experimental setup was chosen to reflect common procedures in clinical research and biobanking emphasizing translational relevance rather than experimental optimization. This is also reflected in our decision to define T1 as the 1 h time point. While a theoretical 0 h reference would provide the most direct measure of the in vivo state, immediate serum processing is rarely feasible in large-scale clinical studies due to both practical aspects of sample collection and the minimum required clotting time (typically 30 min) prior to centrifugation [25,26]. It is likely that, during that time, ongoing enzymatic and cellular activities alter the small molecule composition of the serum, essentially representing a post-clotting rather than an instantaneous metabolic state. The T1 samples therefore represent the earliest practical time point in our design, closely reflecting possible scenarios of routine clinical workflows during multi-patient recruiting. In future studies the use of plasma rather than serum could allow for an earlier and more accurate capture of the short-term metabolic changes, as anticoagulant tubes allow for immediate centrifugation without any clotting delay. This would allow an additional assessment of the ex vivo stability alterations happening during the first hour. While EDTA plasma is indeed increasingly becoming the unspoken matrix of choice for metabolomics studies investigating circulating biomarkers [27,28] and has been shown to result in more reproducible metabolomics data compared to serum [29], serum provides more sensitivity in biomarker detection, while both matrices are capable of reflecting biological alteration between samples to a similar extent [29]. It is questionable, however, whether the required immediate processing, even in the case of plasma, is possible in clinical settings where the metabolic analysis of ever-increasing cohort sizes gains momentum. Therefore, we deliberately aligned our baseline time point with the practical feasibility currently implemented in the collection of our main SLE cohort.

The analytical workflow followed a deliberately conservative design, validated in our previous study [18], to ensure technical robustness and to avoid conflating potential analytical variation with ex vivo stability effects. Accordingly, we focused exclusively on the more stable positive ion mode and applied QC-based coefficient of variation (CV) thresholds as the primary quality metric, rather than using more complex batch-correction algorithms that could potentially overcorrect analytical effects of interest.

Together, these experimental and analytical choices define the scope of the study as an exploratory yet technically stringent assessment of ex vivo metabolite stability.

Compound annotation relied primarily on MS/MS spectral matching against curated reference libraries, supplemented by in silico fragmentation analysis using SIRIUS for metabolites lacking authentic spectra. Annotation levels thus varied between compounds, following accepted metabolomics reporting standards. For patients obtaining mycophenolate treatment, two distinct features were annotated as MPA, both with high MS/MS spectral similarity but different RTs (6.2 min and 7.1 min). We hypothesize that the 6.2 min feature arises from in-source fragmentation of the phase II conjugate MPA-glucuronide. Although this metabolite was not conclusively annotated in MS-DIAL, the assumption is supported by (1) co-eluting peaks at 497.1643 Th, 514.1915 Th, and 519.1473 Th, consistent with [M + H]^+^, [M + NH_4_]^+^, and [M + Na]^+^ adducts of MPA-glucuronide, and (2) earlier elution in reverse-phase chromatography, consistent with the increased polarity of MPA-glucuronide relative to MPA (Figure S8).

### 4.3. Baseline Metabolic Context and Sources of Variability

At the earliest processing time point (T1, 1 h), several metabolites differed between SLE patients and HC. The group separation observed by PCA primarily reflected higher inter-individual variability among SLE samples and the presence of drug-related and disease-associated signals. Especially one SLE patient (SLE02) displayed a great distance from both the HC cluster and other SLE samples and was also marked in two distinct outlier analyses, which was consistent with the individual’s medical history presenting during an acute disease flare rather than a routine visit. Elevated baseline levels of prednisolone and hydroxychloroquine corresponded to documented medication use, confirming the analytical detection of these compounds. Beyond direct drug signals, differences were observed for several amino acids and lipid-related metabolites, including LPC 18:2, GPC, arginine, glutamine, citrulline, cysteine, and hydroxyproline. While these differences align with previous reports of altered amino acid metabolism in SLE, interpretation warrants caution. As highlighted in previous work, amino acid profiles in SLE can be significantly influenced by non-disease factors, including therapy, comorbidities, diet, and even gut microbiota composition [16]. Consequently, although these signals clearly distinguish the two groups in our cohort and are relevant for subsequent interpretation of metabolite stability trends, they should not be over-interpreted as evidence for underlying SLE disease-specific metabolic signatures. Post hoc power analysis indicated that, given the sample sizes (n_SLE = 8, n_HC = 5), the achieved statistical power was below 0.8 for medium effect sizes (Cohen’s d = 0.5) (Figure S2), highlighting that smaller or moderate metabolite differences may not have been detectable. Consequently, some observed differences may not be reproducible in independent cohorts, emphasizing the need for cautious interpretation and validation in larger studies. However, these preliminary baseline differences suggest that intrinsic metabolic changes in SLE patients affect multiple pathways, including immune regulation, oxidative stress, and tissue remodeling. Such alterations could substantially influence the ex vivo stability of individual metabolites.

### 4.4. Multivariate Trends in Ex Vivo Metabolic Drift

Our subsequent PCA analysis across all time points provides an overview of ex vivo metabolic drift and overall reproducibility (Figure 3). Generally, it appears as if HC patients clustered more tightly in their temporal behavior shifting consistently in one direction along PC2, whereas SLE trajectories were more dispersed, consistent with the initial higher inter-patient heterogeneity. This highlights that ex vivo metabolite stability seems to be highly sensitive to pre-analytical handling, and that the additional variability introduced by disease and medication means that only strictly controlled preparation of well-characterized patient cohorts allows for data that can be meaningfully interpreted. An overall convergence across time was not observed, although the data might hint at a slight convergence of samples along PC1 across a full 24 h.

### 4.5. Metabolite-Specific Stability Patterns and Biological Context

In our linear mixed-effects modeling, we turned our attention to a closer inspection of the selected individual metabolites that were characterized by reproducible, time-dependent differences between conditions. It is important to emphasize that the potential mechanistic explanations we discuss below are speculative in nature: while biologically plausible, our study design does not provide direct mechanistic data. The interpretations should therefore be viewed as hypothesis-generating, serving to illustrate potential biological pathways that may underlie the observed stability patterns.

Hypoxanthine is a well-established marker of pre-analytical purine degradation in blood, reflecting the sequential breakdown of ATP to ADP to AMP to adenosine to inosine and finally to hypoxanthine [6]. In our data, hypoxanthine showed the highest overall time-dependent increase, largely independent of disease state. Of note, inosine displayed a similar pattern but did not pass QC criteria (CV = 85.6%) and was therefore not included into the modeled set. A qualitative visualization is provided for contextualization in Figure S9 but excluded from modeling and inference. Hence, our data supports the previously suggested use of hypoxanthine as a universal pre-analytical quality marker while also showing it to be largely independent of disease state and medication.

In contrast Hexose levels declined over time in both groups but with a steeper drop in SLE. While our methodology cannot distinguish various isobaric sugar molecules, it is reasonable to assume that the majority of the signal originates from glucose. The observed ex vivo decline of glucose over time is consistent with the extensive literature on pre-analytical glycolysis effects reported in the context of clinical chemistry [30,31] and metabolomics [5,6,7,32,33,34]. The steeper decline in SLE compared to HC indicates a faster apparent decay rate. However, this difference may partly arise from higher baseline concentrations in SLE, which can influence the apparent slope even if degradation kinetics are similar. Higher baseline glucose values in SLE are not consistently reported in the literature [35,36,37], yet exposure to medication [38,39] or intrinsic metabolic alterations such as inflammation-related insulin resistance [40,41,42,43,44] or simply differences in fasting adherence could plausibly contribute to elevated baseline values observed here. Of note, two SLE patients were treated with SGLT2 inhibitors; however, their glucose profiles were not markedly different from other SLE patients. This observation aligns with previous metabolomics studies in SGLT2 inhibitor-treated cohorts, which similarly reported no major impact on fasting glucose concentrations, provided they are under stable medication regimes [18].

Choline and GPC were both more abundant in SLE than HC at baseline, yet their ex vivo accumulation rates in SLE were substantially slower, eventually leading to a reversed abundance pattern at T6 (24 h). The observed increases are consistent with ongoing ex vivo phosphatidylcholine turnover in whole blood at room temperature via phospholipase-mediated pathways [45]. The faster accumulation in HC could reflect greater substrate availability, fewer medication-related effects on phospholipase and lysosomal activity, or differences in lipoprotein and cell activation profiles. In contrast, slower apparent generation in SLE may arise from altered membrane composition, treatment-related enzyme modulation, or competing metabolic fluxes. The slower ex vivo accumulation of choline and GPC may, in part, be influenced by altered lipoprotein composition. In SLE, there is an increased proportion of triglyceride-rich, phospholipid-poor particles such as very-low-density lipoprotein (VLDL), whereas phospholipid-rich particles, including high-density lipoprotein (HDL), along with Apolipoprotein A (ApoA), are often reduced [46,47,48]. This shift likely limits the availability of substrate for phospholipase-mediated hydrolysis. This pattern may also reflect an enhanced metabolic demand in SLE, where released choline and GPC are more rapidly re-incorporated into biosynthetic pathways for membrane repair and cellular maintenance, even under ex vivo conditions. The higher baseline levels likely reflect the chronic inflammatory state and associated membrane turnover, while the attenuated accumulation may suggest that the metabolic machinery remains more active in SLE samples compared to healthy controls [49,50]. In addition, glucocorticoids are known to modulate lipid metabolism, including effects on phospholipids and triacylglycerides, while HCQ as well as other immunosuppressants are also known to affect lipid metabolism [51,52,53]. Thus, a combination of medication effects, disease activity, and comorbidity likely contributes to choline and GPC alterations [16], making it difficult to properly separate underlying mechanisms without a drug-naïve SLE cohort.

Glutamic and pyroglutamic acid both show a slightly elevated starting concentration in HC over SLE. During room temperature delay, both metabolites increase in both groups, but the increase is consistently steeper in HC. By the final time point (T6, 24 h), HC overtakes SLE. The joint rise in glutamic acid and pyroglutamic acid during whole-blood incubation is biologically plausible and likely largely attributable to pre-analytical processes. Leukocytes and erythrocytes can release amino acids during ex vivo delay and sustained glutamine to glutamic acid conversion due to glutaminase activity could further contribute to increasing levels of free glutamic acid [54,55,56,57,58]. Similarly, residual γ-glutamyl cycle activity can result in pyroglutamic acid accumulation as well as cyclization of N-terminal glutamyl residues in peptides and proteins during ex vivo proteolysis [59,60]. There are different potential explanations for the stark contrast of the ex vivo kinetic observed for glutamic and pyroglutamic acid between HC and SLE. First and foremost, common SLE therapies (e.g., glucocorticoids, HCQ, and mycophenolate) dampen leukocyte activation [61], while glucocorticoids also alter protein metabolism, often increasing protein catabolism and amino acid release [62,63]. Ex vivo, this can reduce the rate of amino acid release and peptide processing, flattening the time-dependent rise in SLE compared to HC. Moreover, even if heightened immune cell glutaminolysis and altered redox handling occurs in SLE, a more rapid re-utilization might occur through fueling the TCA cycle or through transamination, resulting in a more limited net accumulation in serum during the pre-analytical delay [64,65]. An additional explanation for the attenuated ex vivo rise in glutamic and pyroglutamic acid in SLE may be increased glutamic acid utilization for glutathione synthesis. SLE immune cells experience elevated oxidative stress [66,67], which may persist ex vivo, driving higher glutathione demand [40,67]. Consequently, glutamic acid would be consumed intracellularly and pyroglutamic acid may be recycled via 5-oxoprolinase, reducing net serum accumulation compared to healthy controls [68,69].

Glutamine levels were substantially elevated in SLE compared to HC at baseline. Over the room temperature delay, glutamine clearly declines in SLE, whereas concentrations in HC remain comparatively stable. The inverse behavior of glutamine relative to the rising glutamic acid and pyroglutamic acid is consistent with previously reported ongoing ex vivo glutaminolysis and peptide processing in whole blood [5]. It seems fair to assume that leukocytes and erythrocytes remain metabolically active until centrifugation and can lead to a continued conversion of glutamine to glutamic acid consistent with trends we observed for both glutamic acid and pyroglutamic acid [5]. The different trends observed for HC and SLE might be the result of a higher baseline and a faster cellular consumption in SLE if more activated immune cells are present. Furthermore, SLE therapies (glucocorticoids, HCQ, and mycophenolate) can shift leukocyte metabolism [70], while chronic oxidative stress in SLE alters redox handling and increases glutathione turnover [67]. This perturbed γ-glutamyl cycle may indirectly pull on glutamine/glutamic acid pools, which may contribute to pyroglutamic acid accumulation over time [71].

Arginine displays a slight decrease over time in both groups, though at consistently higher levels in SLE. The overall higher abundance of arginine in SLE compared to HC might reflect a combination of corticosteroid-related proteolysis, disease-associated protein turnover, and altered cationic amino acid transport at baseline [16,72,73].

Cystine showed a clear condition × time effect. At baseline (T1), SLE serum contained more cystine than HC, consistent with a more oxidizing extracellular redox state. Over the ex vivo hold, HC rose slightly while SLE declined modestly, increasingly converging over the 24 h delay. We interpret this bidirectional drift as being consistent with ex vivo thiol-disulfide equilibration towards a common steady state rather than a persistent condition-specific development.

Finally, carnitine-related metabolites in our panel displayed a slight decline in SLE over time versus near-flat or mildly rising trajectories in HC. This could be explained by greater ex vivo clearance in SLE via enhanced cellular uptake. This would be consistent with SLE-specific higher immune cell activation and corticosteroid-/immunomodulator-mediated increased cellular uptake.

Several other metabolites met the FDR threshold for interaction despite modest marginal R^2^ (taurine, histidine, creatinine, betaine, citrulline, and paraxanthine). Their high conditional R^2^ indicates that between-subject offsets dominate variance, with small but reproducible condition-dependent time-course differences. These features likely capture subtle individual shifts rather than major group drivers.

It should be noted that mechanistic interpretations remain tentative. The present pilot study was not designed to directly test metabolic pathways. Nonetheless, the systematic patterning across related metabolites supports the biological plausibility of condition-specific ex vivo stability effects.

### 4.6. Pharmacological Modulation of Apparent Metabolite Stability

Medication exposure emerged as a potential contributor to apparent ex vivo metabolite dynamics, although statistical power for subgroup analysis was inherently limited by the cohort size and the treatment heterogeneity. At baseline, several drug-related signals, including those corresponding to prednisolone, hydroxychloroquine, and MPA (including MPA-G), were readily detected and correlated with documented intake. These findings confirm the analytical specificity of the LC-MS workflow but also underscore that direct detection of administered compounds and their metabolites can confound group-level comparisons in mixed-treatment cohorts.

Beyond the direct drug signals, nominal associations were observed for several endogenous metabolites, including lower histidine concentrations in prednisolone-treated patients. This observation is biologically plausible, as glucocorticoids broadly influence amino acid metabolism and protein turnover, for example, to shift amino acid pools toward gluconeogenic use [74,75]. The associations did not survive multiple-testing correction at T1, which is expected in the context of a small sample set in combination with highly varied drug regimens. Hence, although the direction and magnitude are consistent with steroid-related metabolic effects, this result so far is primarily considered exploratory.

Among time-dependent effects, the most prominent signal was the association between mycophenolate exposure and altered AMP trajectories during delayed centrifugation. Patients receiving mycophenolate (CellCept/Myfortic) showed distinct temporal AMP patterns compared with untreated SLE patients in an FDR-significant manner. This is noteworthy given mycophenolate’s known effects on nucleotide metabolism through inosine monophosphate dehydrogenase inhibition [76]. While at this stage fully exploratory and not statistically powerful, this observation supports the biological plausibility of drug–metabolite interactions in shaping systemic metabolic signatures and highlights the importance of considering treatment regimens in longitudinal analyses.

These results emphasize that medication intake represents an important, and often underappreciated, determinant of metabolic stability in clinical studies. Especially in heterogeneous patient populations, unaccounted drug exposure can alter both baseline profiles and ex vivo decay kinetics. Consequently, pharmacological information should be considered integral to study design and interpretation, ideally through stratified sampling, inclusion as covariates in modeling, or parallel in vitro stability testing of affected compounds.

### 4.7. Implications for Clinical Sampling and Biobank Use

Our findings highlight several important considerations for clinical sampling and the use of archived serum specimens. First, we observed that multiple metabolites, including glucose, choline, and the glutamic acid/pyroglutamic acid pair, are highly sensitive to pre-centrifugation delays at room temperature, with divergent kinetics between SLE and HC. For glutamic and pyroglutamic acid in particular, condition-specific accumulation rates can invert relative group differences over time, meaning that unrecorded or uneven delays may obscure or even misrepresent true biological differences. Similarly, delayed centrifugation impacted purine degradation markers such as hypoxanthine, a widely recognized indicator of sample quality [6], underscoring its utility as a universal pre-analytical control independent of disease status.

These results emphasize the need to minimize room-temperature holding times in clinical studies, especially in cohorts with heterogeneous treatment backgrounds. When immediate processing is not feasible, careful documentation of the delay duration is critical, and statistical modeling approaches should retain condition × time interaction terms to avoid conflating pre-analytical artifacts with disease-related biology. Finally, the observation that drug exposures (e.g., mycophenolate affecting AMP trajectories) can shape apparent ex vivo stability highlights the importance of integrating medication history when interpreting both baseline and stability-associated metabolic signals. Collectively, these implications underscore that rigorous pre-analytical control and transparent documentation are prerequisites for reliable metabolomics in SLE and similar patient cohorts.

### 4.8. Implications for SLE and Biomarker Studies

Beyond the methodological implications, our results also raise a more exploratory perspective: condition-specific ex vivo stability patterns may themselves contain biological information. For example, the divergent kinetics of choline and GPC between SLE and healthy controls suggest distinct underlying enzymatic activities that persist or are even exclusive to ex vivo conditions. Such kinetic metabolism fingerprints could, in principle, act as indirect markers of enzymatic activity and may even carry predictive potential for clinical outcomes. Likewise, the observed association between mycophenolate intake and altered AMP trajectories illustrates how medication exposure can shape ex vivo metabolite dynamics in a manner that reflects known pharmacological mechanism. Taken together, these examples highlight that what is commonly dismissed as pre-analytical noise may in fact encode clinically meaningful information, including disease-related alterations in enzymatic activities, drug action, and treatment response. While entirely speculative at this stage, this concept invites future studies to explore ex vivo stability patterns as a novel layer of biomarker discovery.

### 4.9. Limitations of the Study

This study has been designed as a pilot study in preparation to commencing well-informed sample collection for a larger SLE cohort. Hence, the cohort size was small (*n* = 5 HC, *n* = 8 SLE after exclusions), which limits statistical power and generalizability and did not allow for proper control of age and sex. For medication-stratified analyses, subgroup sizes were even smaller, calling for confirmation of observed effects in larger, stratified cohorts. The heterogeneous nature of the SLE population, encompassing varied disease activity states and treatment regimens, further complicates disentangling disease-intrinsic from drug-related effects. Our design did not include an immediate processing control (T0), restricting our ability to capture the earliest degradation events and to establish absolute in vivo baselines. Hence, T1 is a pragmatic baseline in the context of feasibility in clinical sample collection, yet it cannot be regarded as a ground truth baseline measurement. Finally, although linear mixed-effects models provide an appropriate framework for repeated measures, the multiple testing burden across >50 metabolites in such a small dataset increases the risk of false positives, and all mechanistic interpretations remain speculative without targeted biochemical validation. Collectively, these constraints mean our findings should be interpreted as proof of concept rather than definitive characterization of metabolite stability in SLE. Yet they do highlight the need for standardized pre-analytical controls in a disease-specific manner and the risks but also the chances of pre-analytical centrifugation delay in clinical metabolomics.

## 5. Conclusions

Our study highlights that pre-analytical variation in serum metabolomics cannot be evaluated in isolation from disease and medication context. While prior research has emphasized on metabolite stability in healthy individuals, our findings demonstrate that patients with SLE display distinct ex vivo kinetics that are further shaped by drug exposure. Failure to account for these factors risks false assumptions about disease biology and biomarker validity. At the same time, the reproducible divergence of stability profiles between SLE and HC suggests that ex vivo dynamics may provide new biological insights, potentially serving as complementary markers of enzymatic activity and clinical status. Moreover, the example of mycophenolate-associated AMP trajectories illustrates that stability patterns can also reflect pharmacological action, opening the possibility of using ex vivo kinetics as indirect markers of drug response. Taken together, these observations stress the need for rigorous pre-analytical documentation in metabolomics, while also opening the door to novel uses of stability patterns as informative features in both disease characterization and therapeutic monitoring.

## Figures and Tables

**Table 1:** Demographic, clinical, and serological baseline characteristics of the study cohort.

Characteristics	SLE (*n* = 10 ^1^)	HC (*n* = 5 ^1^)	*p*-Value ^2^
Age—yr	48 ± 16	33 ± 5	0.043
Female sex	9/10 (90%)	3/5 (60%)	0.2
**Immunosuppressive medication**			
Antimalarial agent	7/10 (70%)		
Glucocorticoids	5/10 (50%)		
Mycophenolate	3/10 (30%)		
Belimumab	5/10 (50%)		
Azathioprine	2/10 (20%)		
Tacrolimus	1/10 (10%)		
**Disease activity**			
SLEDAI-2k	2.60 ± 2.12		
PGA Score	0.80 ± 0.63		
LLDAS	6/10 (60%)		
DORIS	2/10 (20%)		
**Serology**			
Anti-ds-DNA-Antibodies—IU/mL	31 ± 41		
Hypocomplementemia C3c	3/10 (30%)		
Hypocomplementemia C4	6/10 (60%)		
Antiphospholipid antibodies	4/10 (40%)		

^1^ *n*/N (%); Mean ± SD. ^2^ Wilcoxon rank sum test; Fisher’s exact test.

## Data Availability

Raw measurement data and aggregated data are not publicly available due to institutional data protection regulations. To meet the general idea of verification and reproducibility of scientific findings, we offer access to data at the local database on request via the corresponding author. The R code used for data analysis is available on GitHub: https://github.com/c373s/SLE_Stability.

## Supplementary Materials

The following supporting information can be downloaded at: https://www.mdpi.com/article/10.3390/metabo15110738/s1, Figure S1: MS1 peak density distribution of annotated features. Histogram of MS1 mass errors for all annotated features. Mass errors were calculated based on average *m*/*z* to reference *m*/*z* in Dalton. Red line indicates zero error. Panel statistics include the count of features (n) and mean mass error (Da). Figure S2: Post hoc power analysis for SLE vs. HC comparisons at T1. Power curves were computed across a range of Cohen’s d effect sizes based on the observed sample sizes (n_SLE = 8, n_HC = 5). The red dashed line indicates the conventional 80% power threshold, and the blue dashed line shows the minimum detectable effect size at 80% power. Figure S3: Side-by-side volcano plots showing raw versus FDR-adjusted significance at T1. The left panel displays significance based on raw *p*-values (*p* < 0.05), and the right panel shows FDR-adjusted significance (FDR < 0.05). Top metabolites are labeled consistently across panels to illustrate the impact of multiple testing correction. Figure S4: Mahalanobis distance analysis of the T1 dataset. Distances represent the multivariate deviation of each patient from the condition centroid in metabolite space. SLE02 displays the largest deviation by a wide margin. Figure S5: Robust z-score analysis of the T1 dataset within each condition. Bars show the fraction of metabolites per patient deviating >3 standard deviations from the group median. SLE02 shows the highest fraction of outliers across all participants. Figure S6: Estimated marginal mean (EMM) trajectories for all modeled metabolites. Each panel shows raw intensities per patient (points and thin lines) and model-predicted means with 95% confidence intervals (triangles and error bars) from linear mixed-effects models (Condition × Time_f, random intercept for Patient_ID). Panel subtitles report marginal R^2^ (variance explained by fixed effects), conditional R^2^ (fixed + random effects), and the false discovery rate (FDR)-adjusted q-value for the Condition × Time interaction. Positive q-values below 0.05 indicate significantly different temporal trajectories between SLE and HC. Figure S7: Estimated marginal mean (EMM) trajectories for all QC-passed metabolites (CV < 25%) by drug exposure in SLE patients. Linear mixed-effects models were fitted separately for each metabolite with fixed effects for time (T1–T6), drug exposure (binary factor), and their interaction, and Patient_ID as a random intercept. Each panel shows raw patient trajectories (grey = drug−, orange = drug+) and model-predicted means with 95% confidence intervals. Panel subtitles report marginal R^2^ (variance explained by fixed effects), conditional R^2^ (variance explained by fixed + random effects), and the FDR-adjusted q-value for the time × drug interaction. Panels are ordered within each drug by increasing interaction q-value. The figure is provided as a multi-page PDF (295 pages). Figure S8: Extracted ion chromatograms of mycophenolic acid (MPA) and putative MPA-glucuronide (MPA-G) adducts detected in serum of SLE patients. Top left: MPA [M + H]^+^ at 321.1329 Th (retention time ~6.2 min). Top right and bottom panels: co-eluting peaks at 497.1643 Th, 514.1915 Th, and 519.1473 Th, consistent with expected MPA-glucuronide [M + H]^+^, [M + NH_4_]^+^, and [M + Na]^+^ features, respectively. Blue traces represent individual serum samples; green traces represent traces of the pooled QC samples. The MPA-G features eluted earlier than MPA, consistent with the increased polarity of the glucuronide conjugate and supporting the hypothesis that the 6.2 min MPA feature arises from in-source fragmentation of MPA-glucuronide. Figure S9: Time-course of inosine intensities in HC and SLE. Lines denote individual patients. Inosine is shown for pathway context relative to hypoxanthine but was excluded from statistical modeling due to high analytical variability (QC CV = 85.6%). Table S1: Feature annotation information. Columns: Average RT—average retention time across aligned features, Average *m*/*z*—average *m*/*z* across aligned features; Reference *m*/*z*—*m*/*z* of monoisotopic reference mass; Dalton Mass Error—mass error of average *m*/*z* to reference *m*/*z* in Dalton; MS-DIAL Annotation—original output from MS-DIAL prior to curation; Curated Common/Generic Name—final metabolite/feature label used throughout study; Notes—e.g., explanation of abbreviations, dietary/contaminant features, and annotation confidence; Annotation Method—MSMLS: validated by in-house library of standards, Public: annotated via public spectral libraries and MS/MS match, SIRIUS: structure prediction by SIRIUS. Table S2: QC-based variability metrics per metabolite. Table summarizing coefficient of variation (CV) across pooled QC samples for all annotated metabolite features. Columns: Curated Common/Generic Name—feature label used throughout the study; Mean; SD—standard deviation; CV [%]—coefficient of variation in%; n_QC—number of QC samples used for CV calculation. Features with QC CV < 25% were retained for statistical modeling, while features with higher variability were excluded from inferential analyses. Table S3: Baseline (T1) group comparison between SLE and HC. Summary statistics for all metabolites at baseline (T1), used to generate the volcano plot. Columns: Metabolite—curated common/generic name; n_C—number of HC samples with non-missing values; n_SLE—number of SLE samples with non-missing values; mean_C—mean intensity in HC; mean_SLE—mean intensity in SLE; log2_fc—log2 fold change, SLE/HC; *p*_value—Welch’s *t*-test; FDR—Benjamini–Hochberg adjusted p; neglog10p—–log_10_(p); fdr_sig—Boolean flag indicating whether the metabolite passes FDR < 0.05; p_sig—Boolean flag indicating whether the metabolite passes nominal *p* < 0.05; direction—classification into upregulated, downregulated, or not significant. Table S4: Outlier detection analysis. Summary of outlier metrics per sample based on z-score and Mahalanobis distance. Columns: Condition—sample group, SLE or HC; Patient_ID; outlier_frac_3sd—fraction of metabolites with intensity deviating more than 3 standard deviations from the group mean; outlier_frac_2_5—fraction of metabolites exceeding 2.5 SD; Condition—sample group, SLE or HC; Mahalanobis2—squared Mahalanobis distance of the sample in multivariate space relative to its group; p_outlier—associated *p*-value for the Mahalanobis distance under a χ^2^ distribution. These metrics were used to identify potential technical or biological outliers. Table S5: Results of linear mixed-effects modeling of metabolite intensities, including model fit metrics, Type III ANOVA of fixed effects, and estimated contrasts between SLE and HC at individual timepoints. (A) Model fit and interaction significance for each metabolite from linear mixed-effects models (LME). Each metabolite was modeled as a function of condition (HC vs. SLE), time (T1–T6), and their interaction, with Patient_ID included as a random intercept. Columns: Metabolite—compound name; R^2^_marginal—proportion of variance explained by the fixed effects (condition, time, and interaction); R^2^_conditional—proportion of variance explained by the full model including fixed and random effects; q_value—false discovery rate (FDR) adjusted *p*-value (Benjamini–Hochberg) for the interaction term. (B) Type III ANOVA results from linear mixed-effects models (LME). Each metabolite was modeled as a function of condition (HC vs. SLE), time (T1–T6), and their interaction, with Patient_ID included as a random intercept. Columns: Metabolite—compound name; term—fixed effect tested (Condition, Time_f, or Condition × Time_f interaction); NumDF—numerator degrees of freedom; DenDF—denominator degrees of freedom (Satterthwaite approximation); F value—F-statistic for the effect; *p*_value—raw *p*-value for the fixed effect. (C) Estimated marginal mean contrasts between SLE and HC for each metabolite at each timepoint, derived from linear mixed-effects models (LME). For each metabolite and timepoint (T1–T6), the model estimated the mean difference in raw intensity units between SLE and HC (SLE–HC), accounting for repeated measures by including Patient_ID as a random intercept. Columns: Metabolite—compound name; Time—sampling timepoint; estimate_SLE_minus_HC—model-based difference in mean intensity between SLE and HC at this timepoint (positive = higher in SLE); SE—standard error of the estimate; df—Satterthwaite-approximated denominator degrees of freedom; t.ratio—test statistic (estimate ÷ SE); *p*_value—raw *p*-value for the SLE vs. HC contrast at this timepoint. Table S6: Baseline (T1) drug–metabolite associations in SLE patients. For each drug exposure, SLE patients were stratified into drug+ and drug− subgroups, and group differences in metabolite intensities were tested. Columns: Metabolite—compound name; n_pos/n_neg—number of patients in drug+ vs. drug− groups; log2FC—log_2_ fold change of mean intensity (drug+/drug−); p_t—Welch’s t-test *p*-value; p_wilcox—Wilcoxon rank-sum test *p*-value; hedges_g—effect size (Hedges’ g); drug—medication used for stratification; q_t—FDR-adjusted *p*-value (Benjamini–Hochberg) from Welch’s t-tests. These analyses included all detected metabolites, including drug-related features and those not passing QC filtering, and should be considered exploratory. Table S7: Linear mixed-effects (LME) models testing for drug-by-time interactions in SLE patients. Analyses were restricted to metabolites meeting QC criteria (CV < 25% in pooled QC samples). Columns: Metabolite—compound name; p_interaction—raw *p*-value for the time × drug interaction; R^2^_marginal—variance explained by fixed effects (Time, Drug, and Time × Drug); R^2^_conditional—variance explained by the full model including random effects; drug—drug exposure variable; q_interaction—false discovery rate (FDR, Benjamini–Hochberg) adjusted *p*-value for the interaction term.
