# Supplementary material for: Disease and Medication Context Shape Ex Vivo Metabolite Stability: A Pilot Study in Systemic Lupus Erythematosus
Source: Metabolites. 2025 Nov 12;15(11):738. doi: 10.3390/metabo15110738 (PMC12654355; doi:10.3390/metabo15110738)
Supplement: Supplementary file 1 [file metabolites-15-00738-s001.zip › Supplementary Figure S6.pdf]

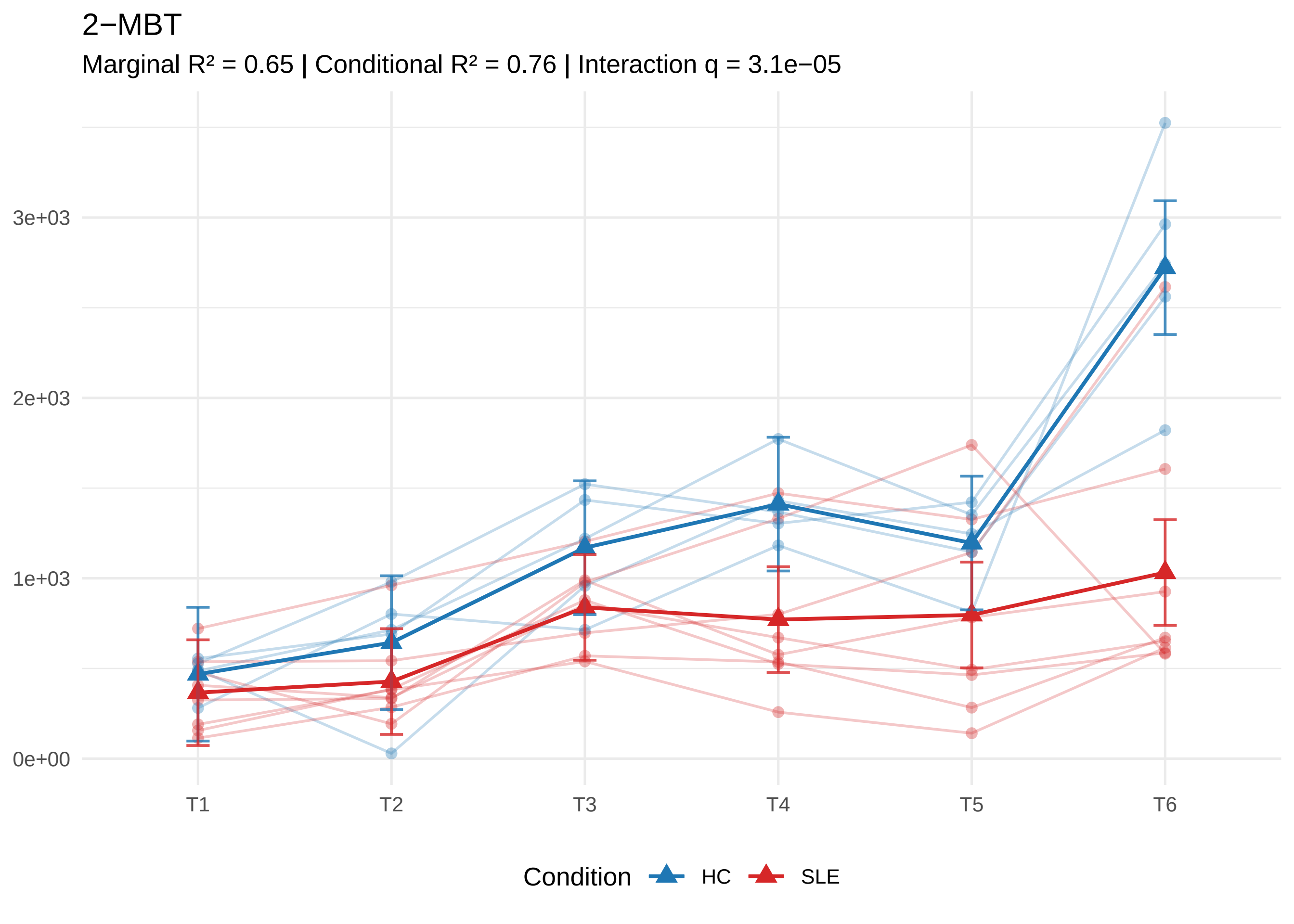

2−MBT
Marginal R² = 0.65 | Conditional R² = 0.76 | Interaction q = 3.1e−05
3e+03
2e+03
1e+03
0e+00
T1
T2
T3
T4
T5
T6
Condition
HC
SLE

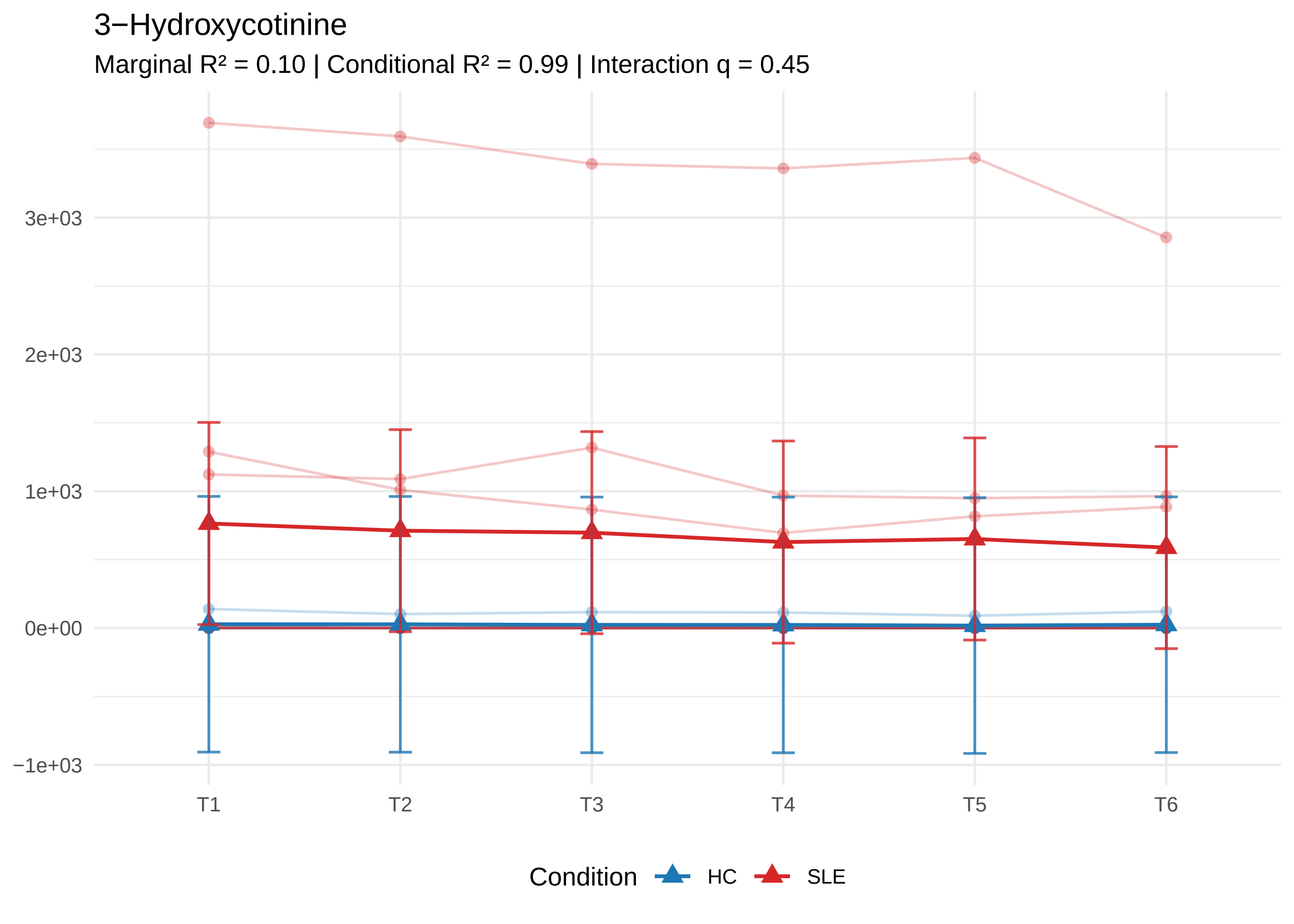
3−Hydroxycotinine
Marginal R² = 0.10 | Conditional R² = 0.99 | Interaction q = 0.45
3e+03
2e+03
1e+03
0e+00
−1e+03
T1
T2
T3
T4
T5
T6
Condition
HC
SLE

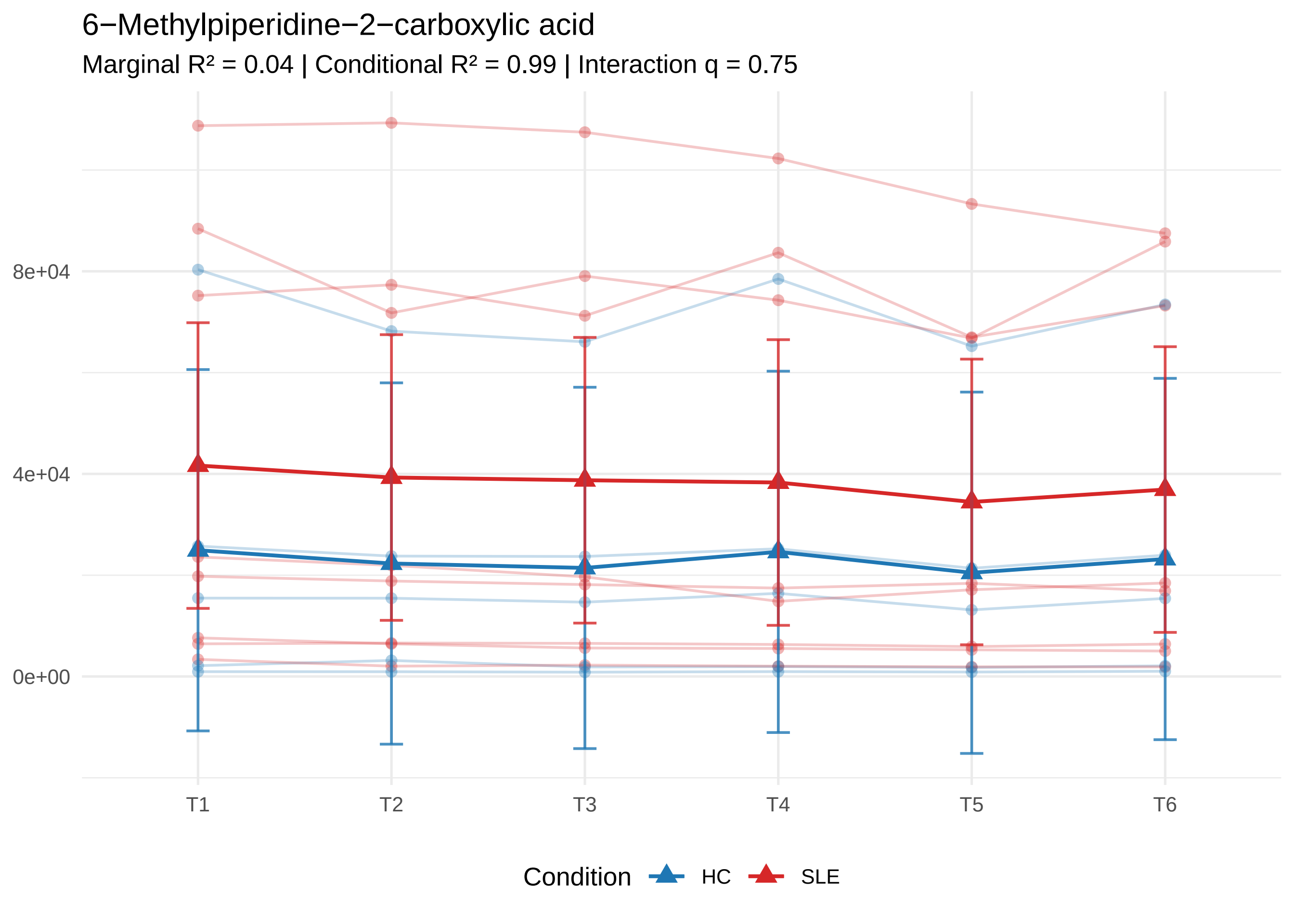

6−Methylpiperidine−2−carboxylic acid
Marginal R² = 0.04 | Conditional R² = 0.99 | Interaction q = 0.75
8e+04
4e+04
0e+00
T1
T2
T3
T4
T5
T6
Condition
HC
SLE

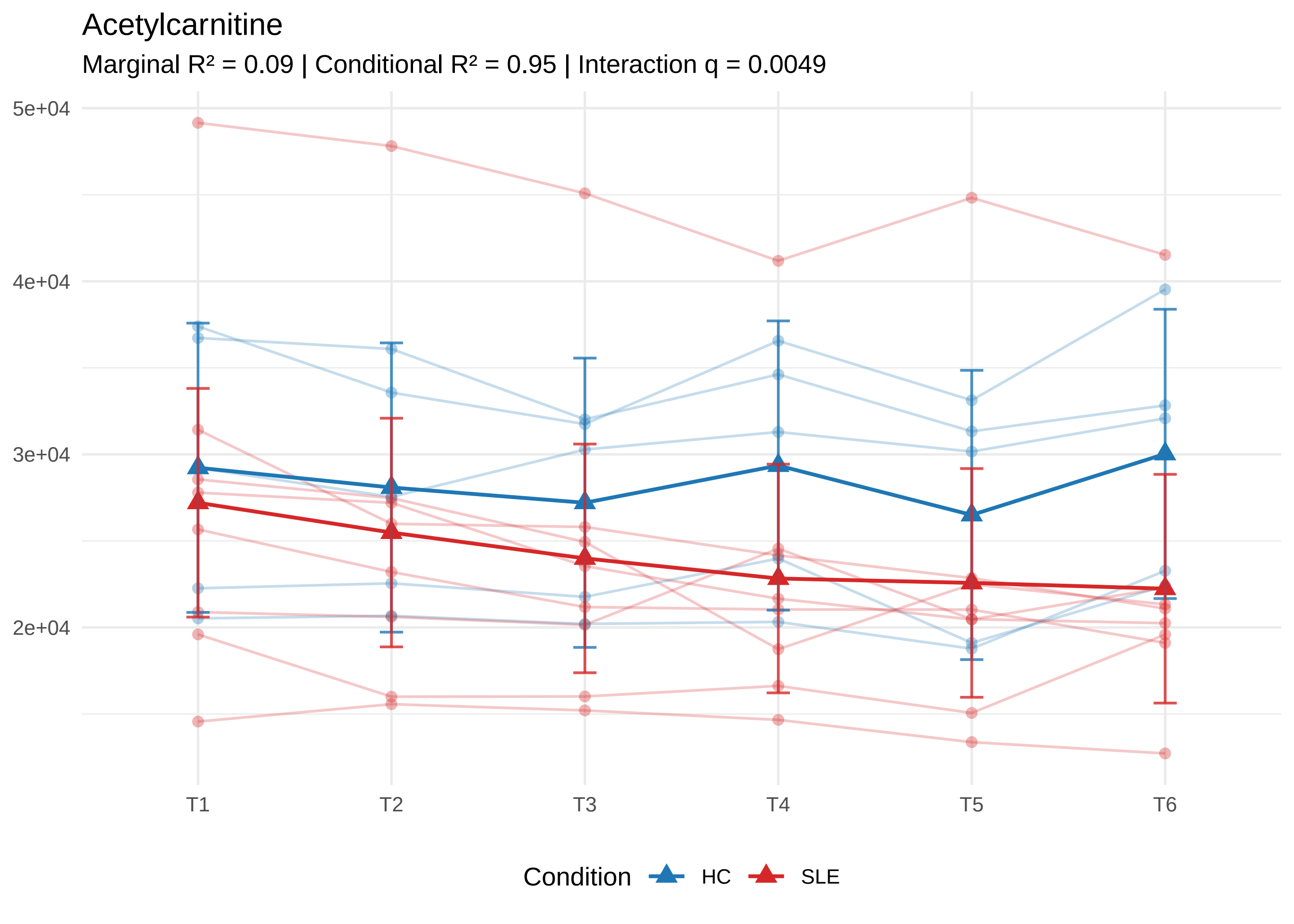
Acetylcarnitine
Marginal R² = 0.09 | Conditional R² = 0.95 | Interaction q = 0.0049
5e+04
4e+04
3e+04
2e+04
T1
T2
T3
T4
T5
T6
Condition
HC
SLE

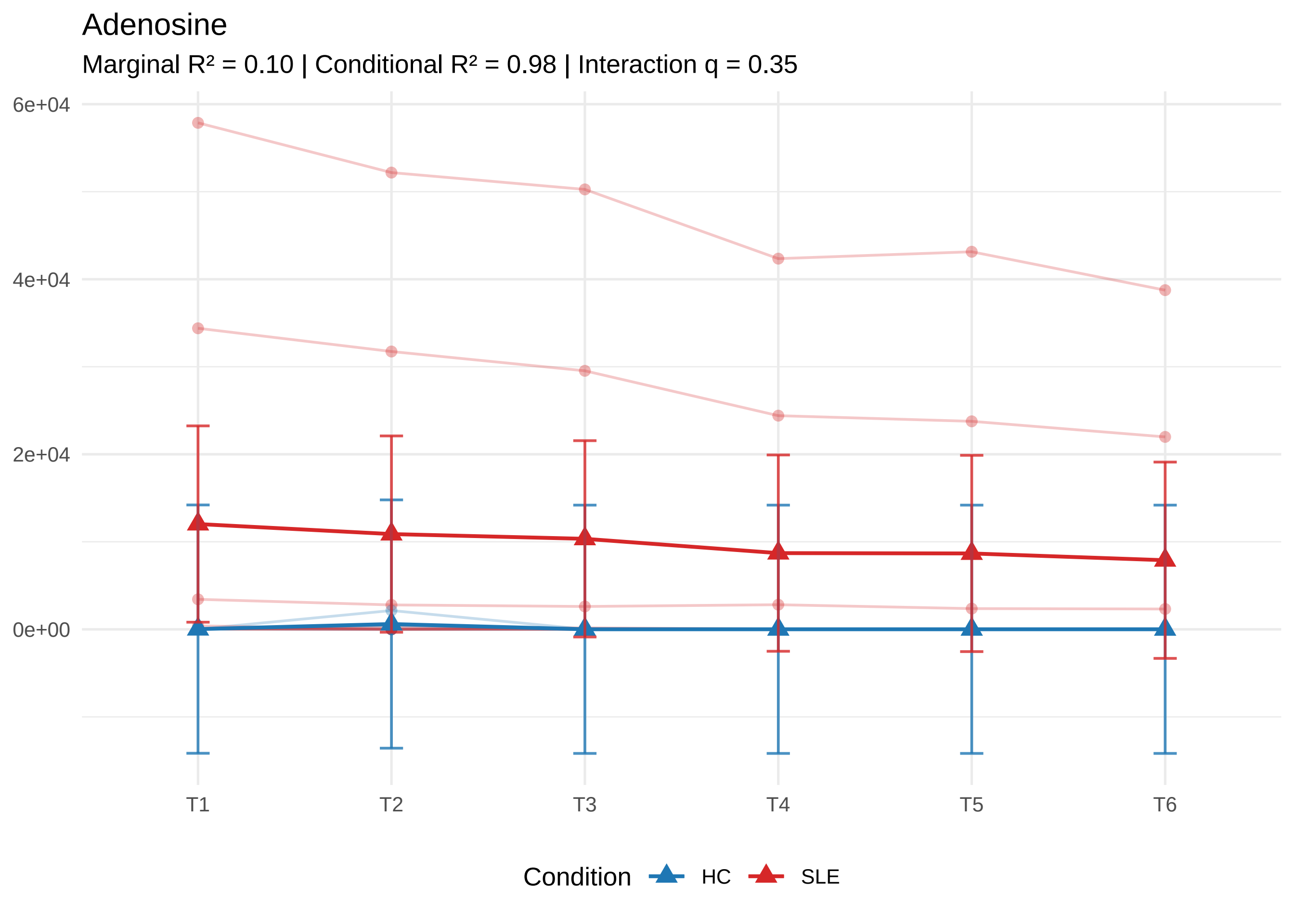
Adenosine
Marginal R² = 0.10 | Conditional R² = 0.98 | Interaction q = 0.35
6e+04
4e+04
2e+04
0e+00
T1
T2
T3
T4
T5
T6
Condition
HC
SLE

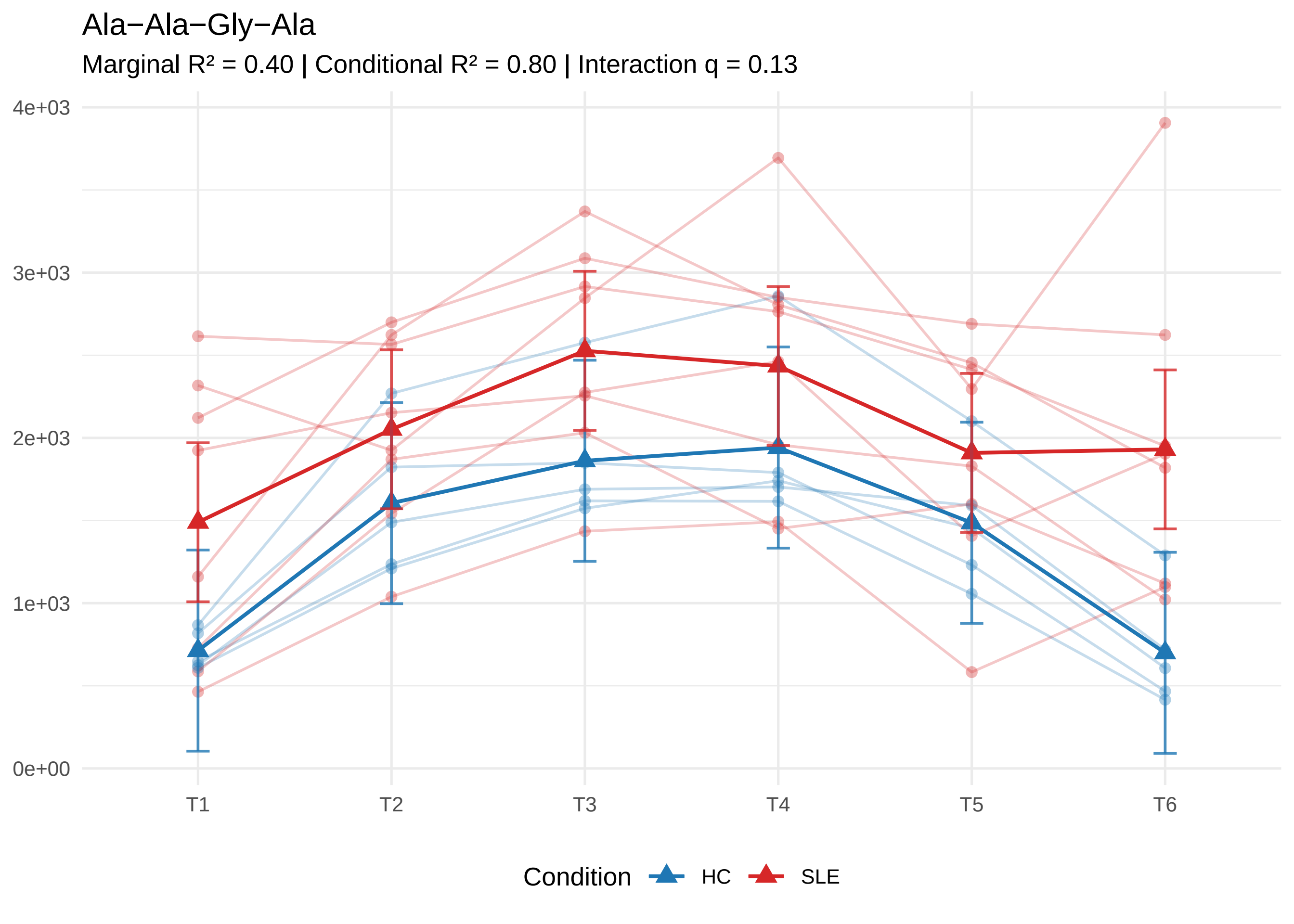

Ala−Ala−Gly−Ala
Marginal R² = 0.40 | Conditional R² = 0.80 | Interaction q = 0.13
4e+03
3e+03
2e+03
1e+03
0e+00
T1
T2
T3
T4
T5
T6
Condition
HC
SLE

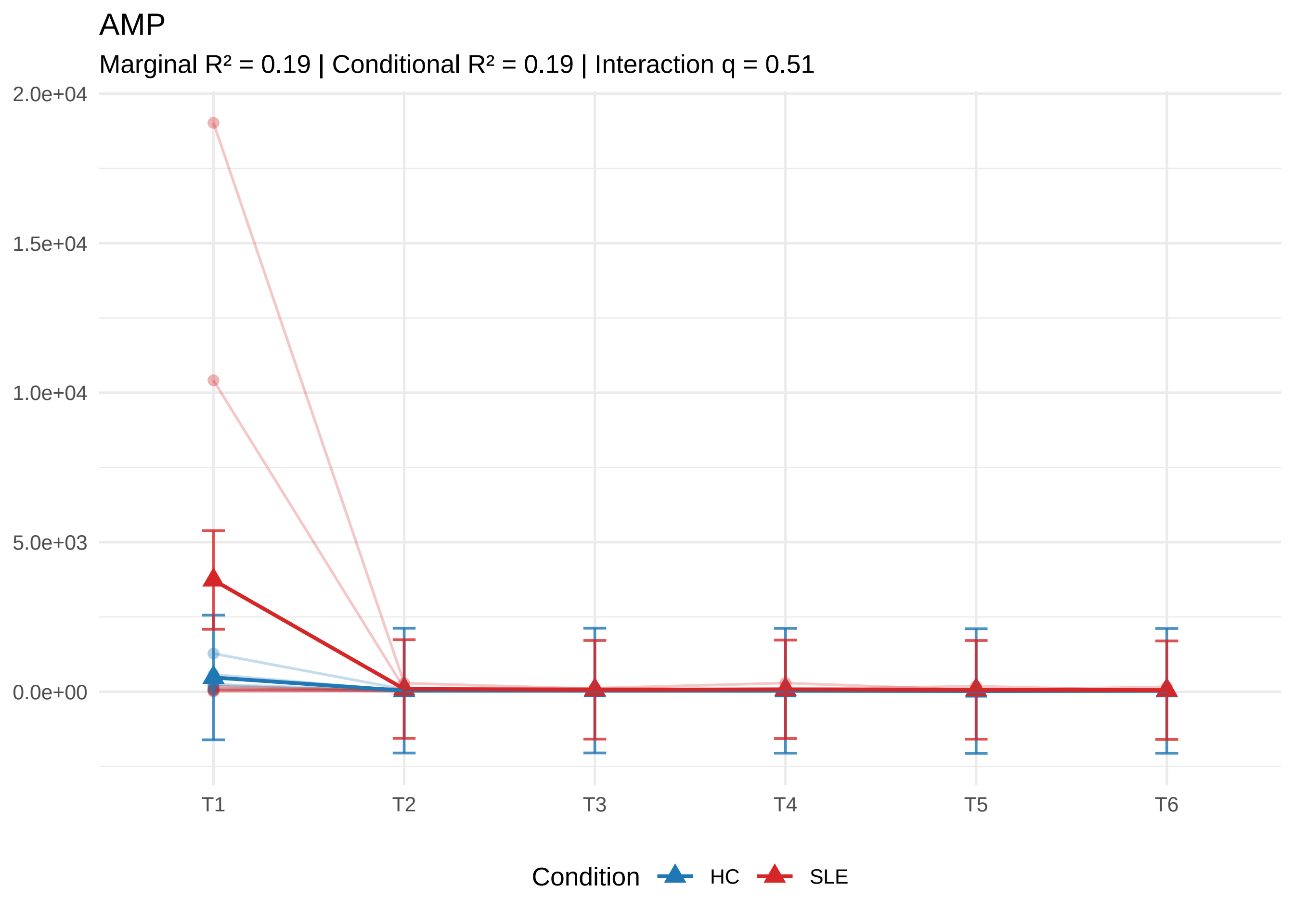
AMP
Marginal R² = 0.19 | Conditional R² = 0.19 | Interaction q = 0.51
2.0e+04
1.5e+04
1.0e+04
5.0e+03
0.0e+00
T1
T2
T3
T4
T5
T6
Condition
HC
SLE

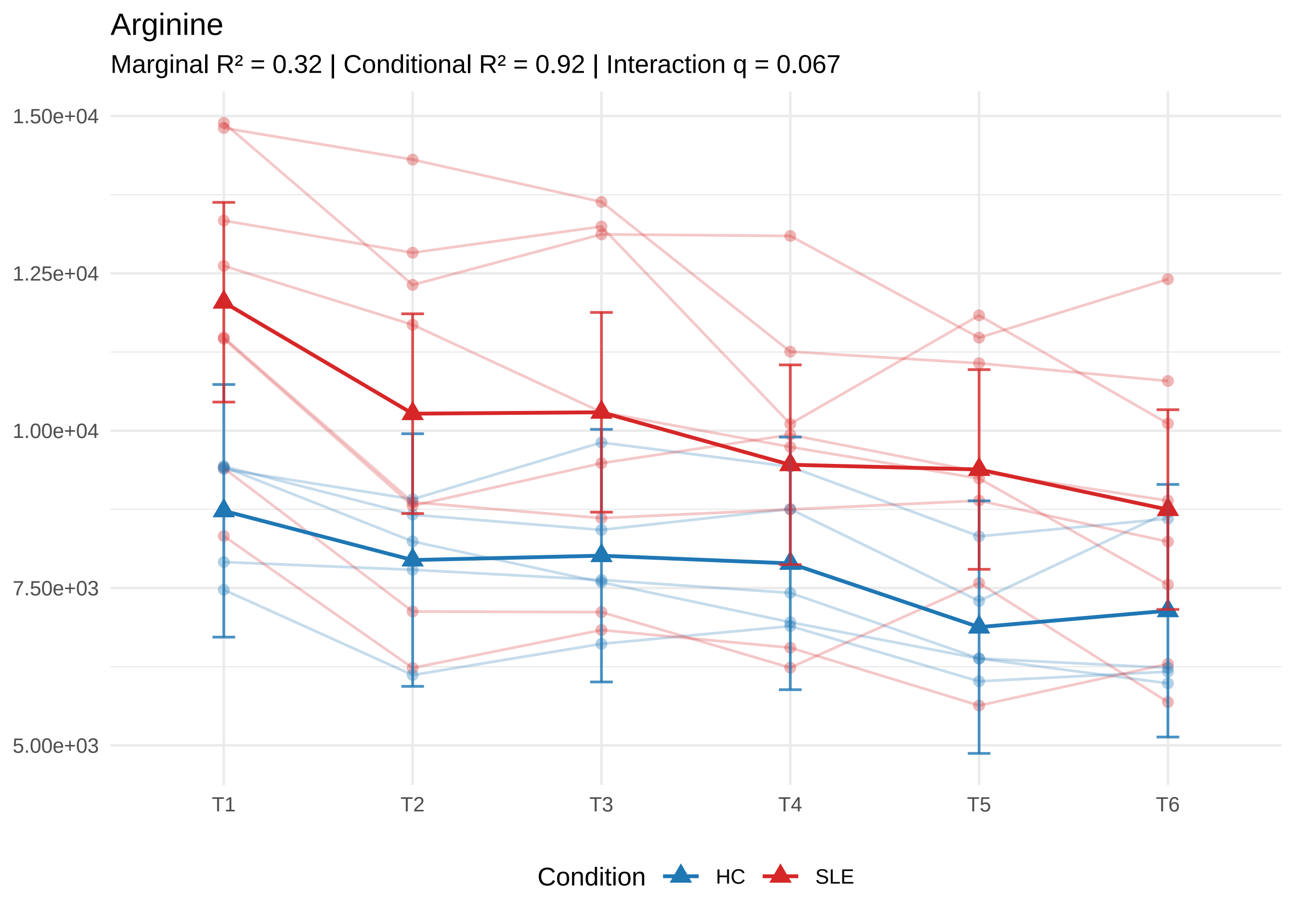

Arginine
Marginal R² = 0.32 | Conditional R² = 0.92 | Interaction q = 0.067
1.50e+04
1.25e+04
1.00e+04
7.50e+03
5.00e+03
T1
T2
T3
T4
T5
T6
Condition
HC
SLE

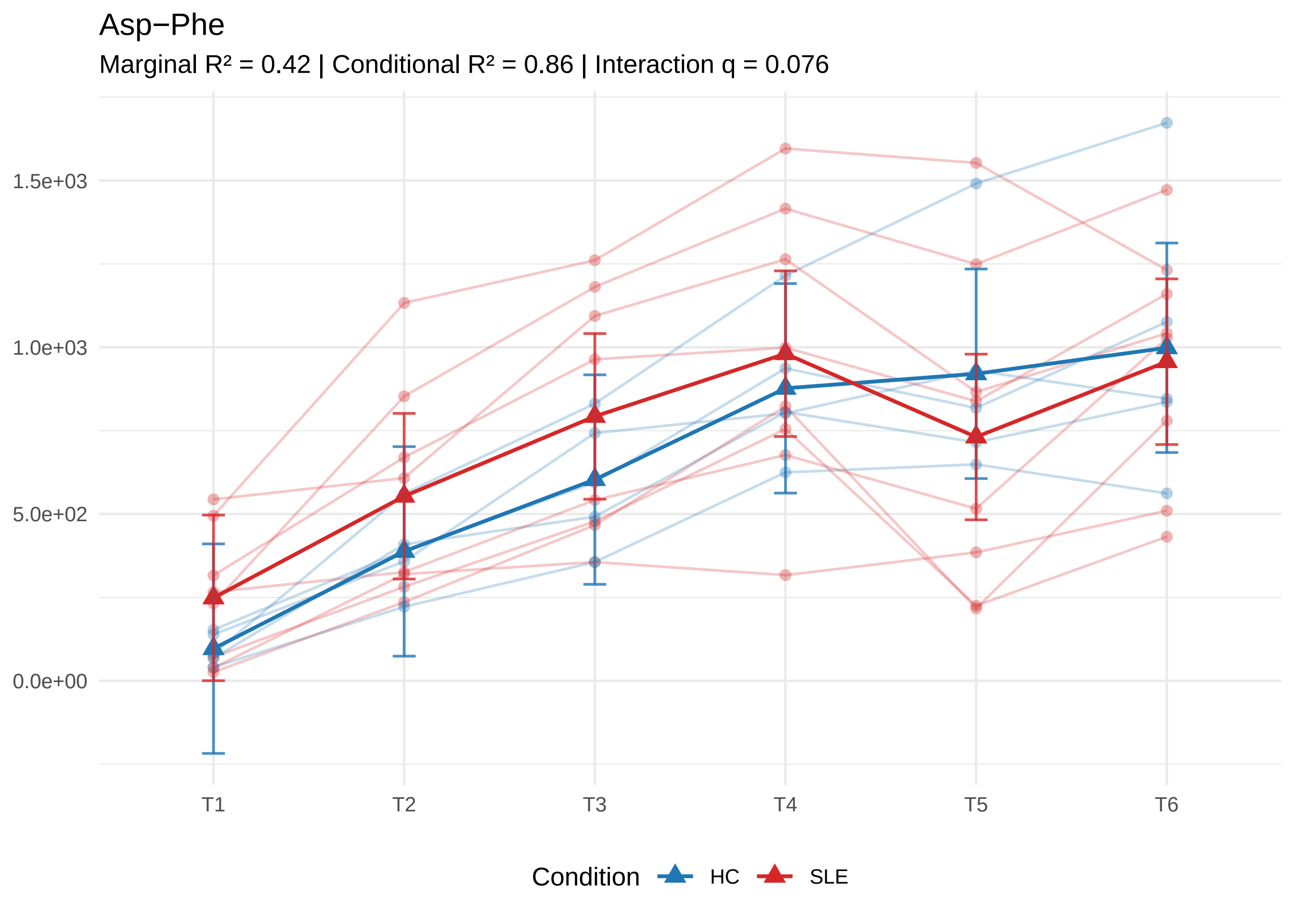
Asp−Phe
Marginal R² = 0.42 | Conditional R² = 0.86 | Interaction q = 0.076
1.5e+03
1.0e+03
5.0e+02
0.0e+00
T1
T2
T3
T4
T5
T6
Condition
HC
SLE

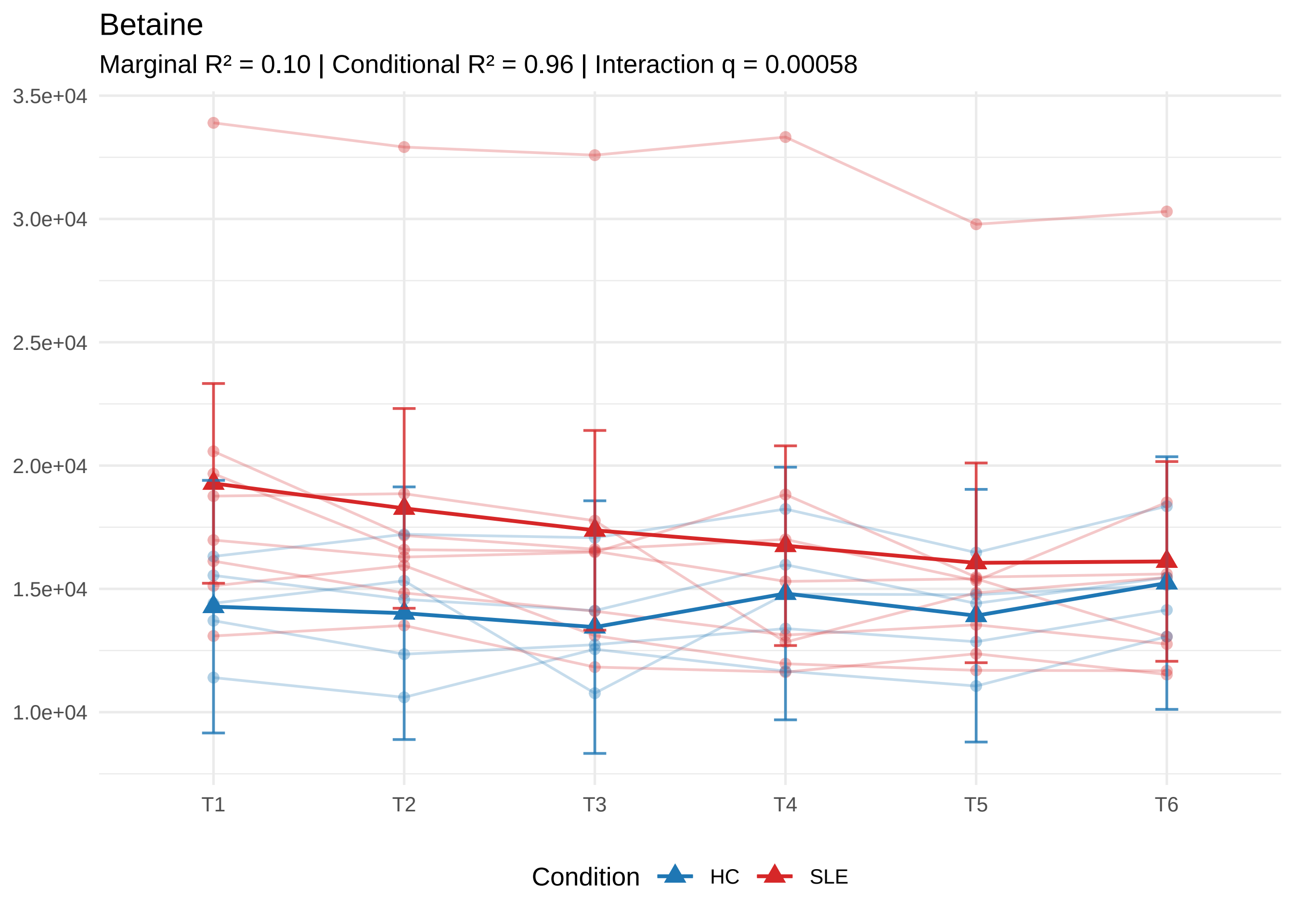

Betaine
Marginal R² = 0.10 | Conditional R² = 0.96 | Interaction q = 0.00058
3.5e+04
3.0e+04
2.5e+04
2.0e+04
1.5e+04
1.0e+04
T1
T2
T3
T4
T5
T6
Condition
HC
SLE

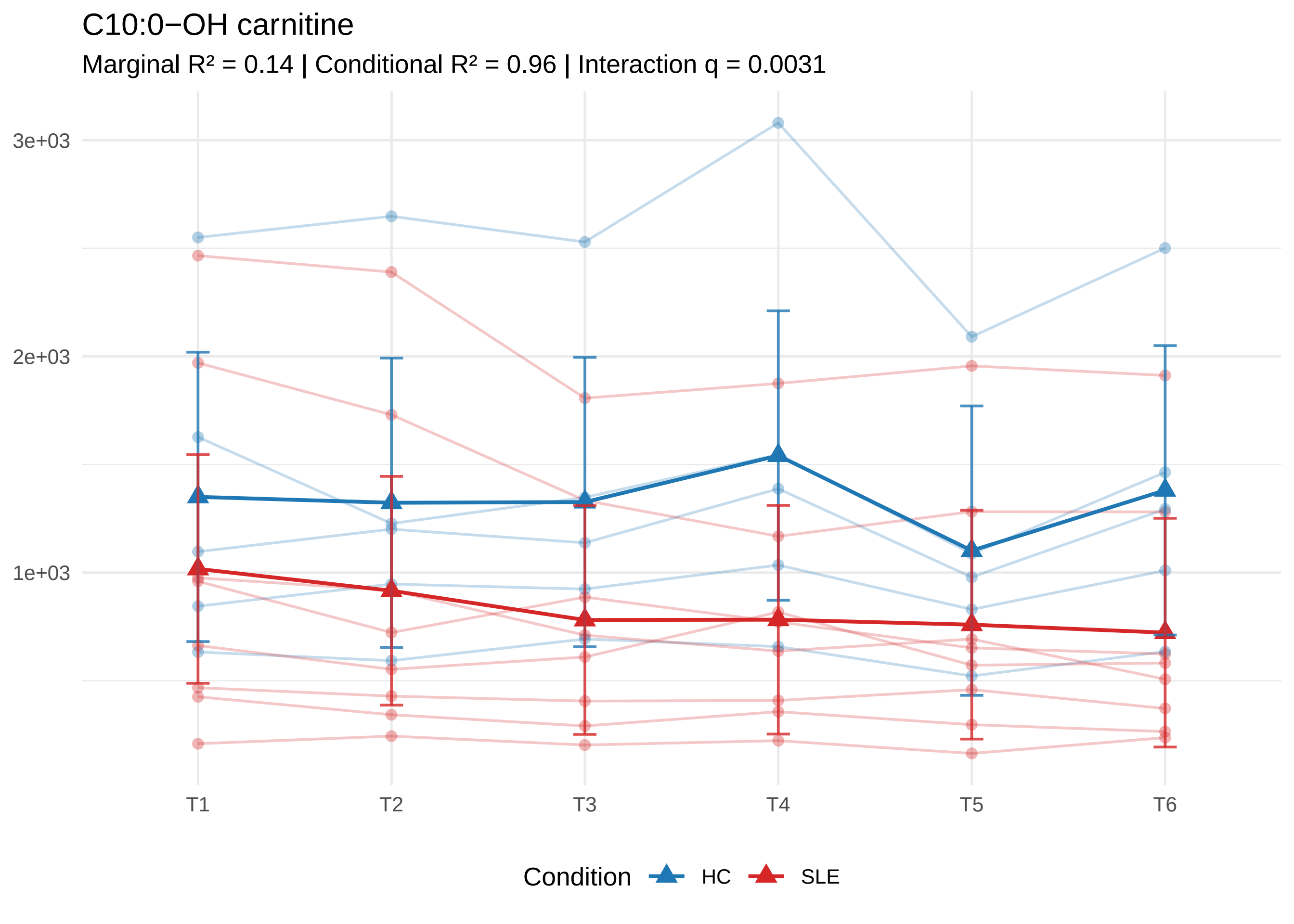
C10:0−OH carnitine
Marginal R² = 0.14 | Conditional R² = 0.96 | Interaction q = 0.0031
3e+03
2e+03
1e+03
T1
T2
T3
T4
T5
T6
Condition
HC
SLE

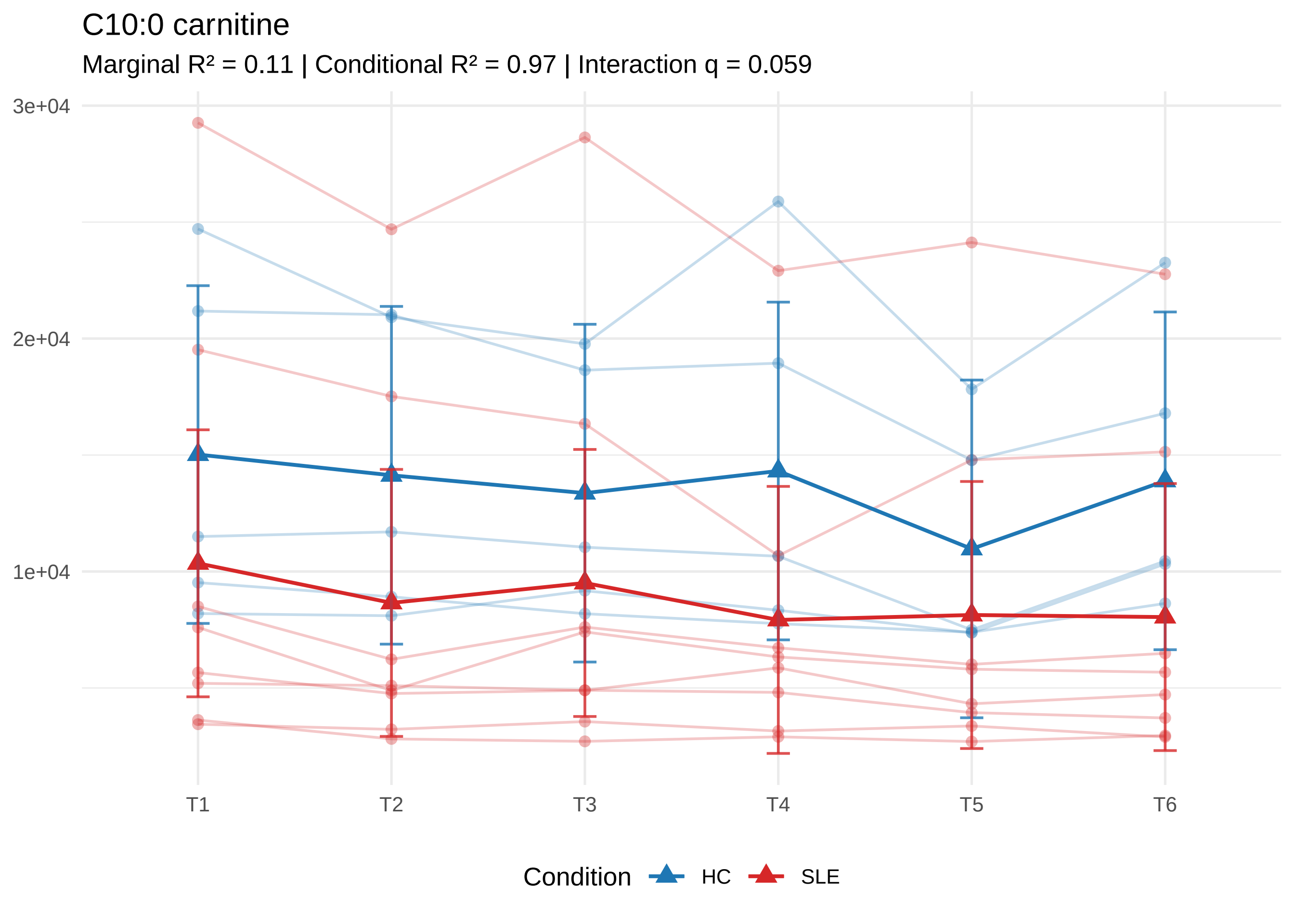

C10:0 carnitine
Marginal R² = 0.11 | Conditional R² = 0.97 | Interaction q = 0.059
3e+04
2e+04
1e+04
T1
T2
T3
T4
T5
T6
Condition
HC
SLE

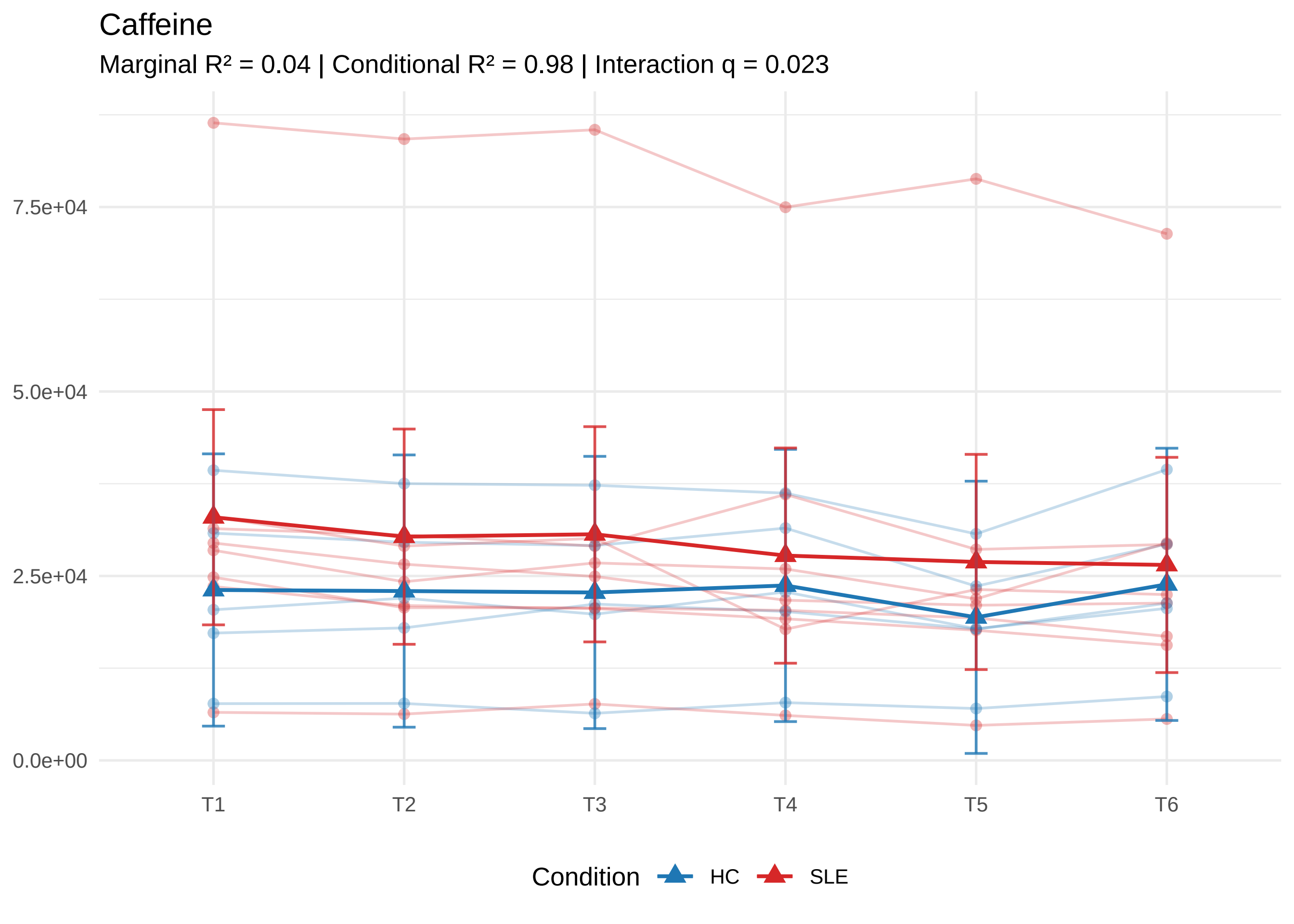

Caffeine
Marginal R² = 0.04 | Conditional R² = 0.98 | Interaction q = 0.023
7.5e+04
5.0e+04
2.5e+04
0.0e+00
T1
T2
T3
T4
T5
T6
Condition
HC
SLE

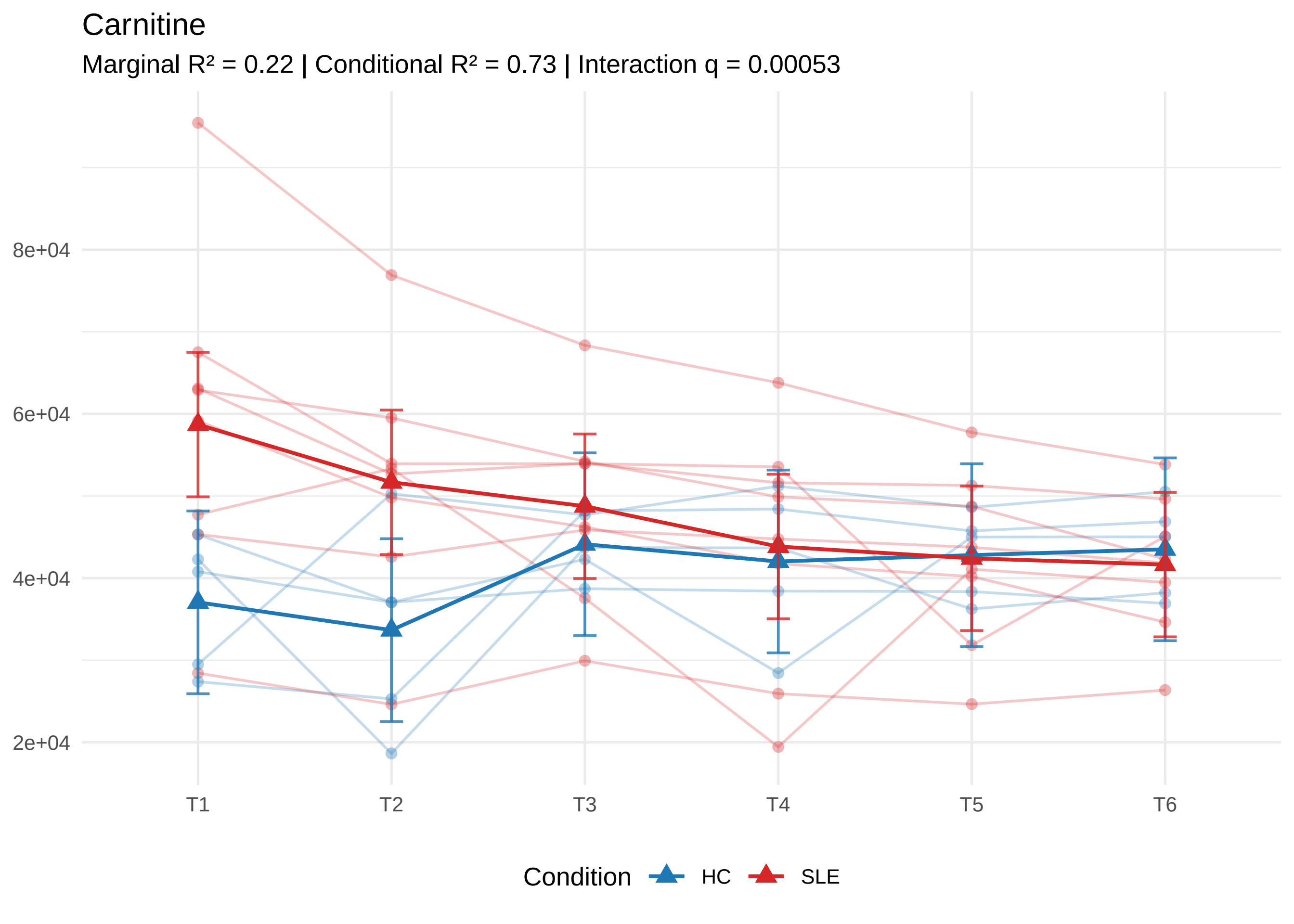

Carnitine
Marginal R² = 0.22 | Conditional R² = 0.73 | Interaction q = 0.00053
8e+04
6e+04
4e+04
2e+04
T1
T2
T3
T4
T5
T6
Condition
HC
SLE

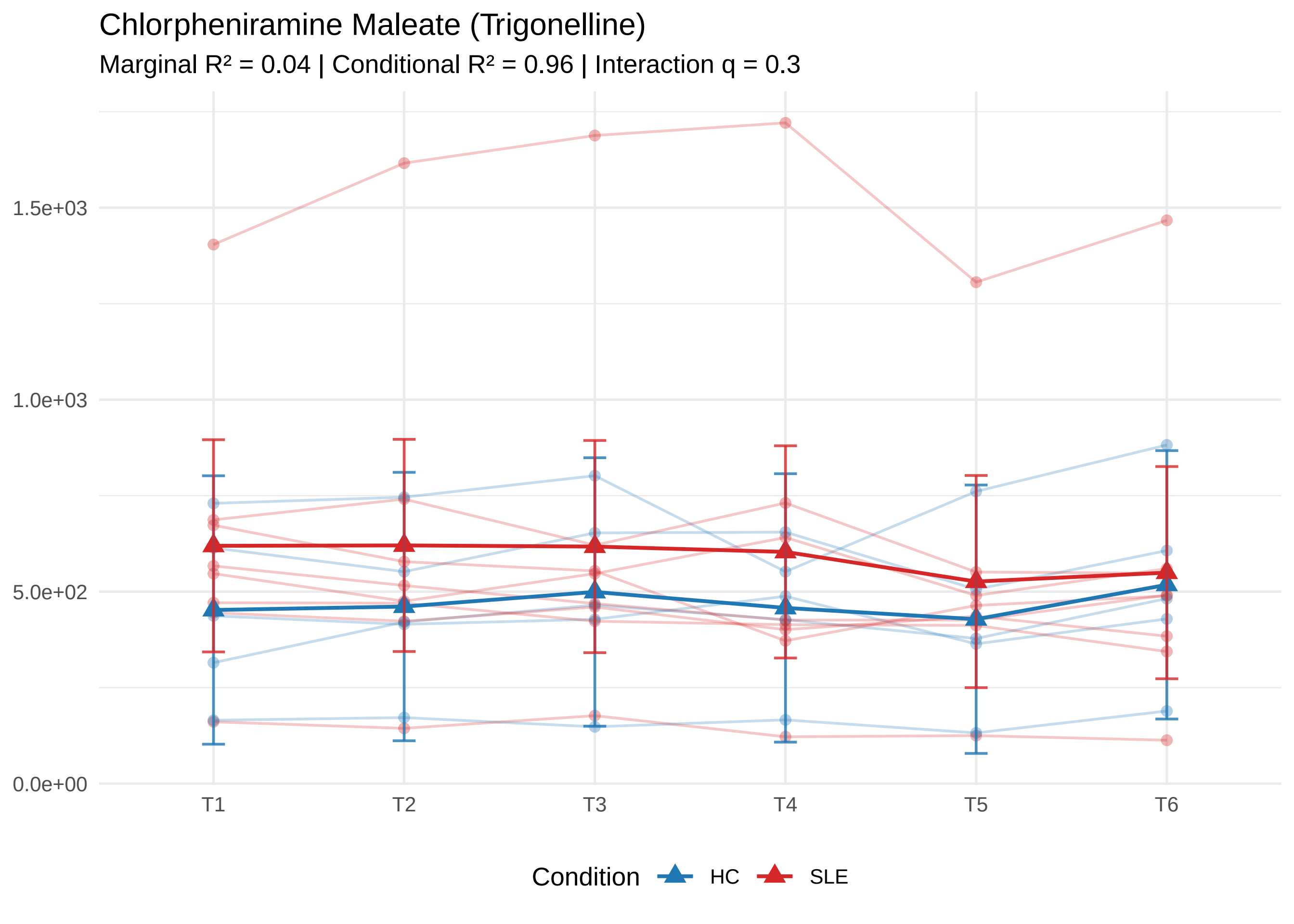
Chlorpheniramine Maleate (Trigonelline)
Marginal R² = 0.04 | Conditional R² = 0.96 | Interaction q = 0.3
1.5e+03
1.0e+03
5.0e+02
0.0e+00
T1
T2
T3
T4
T5
T6
Condition
HC
SLE

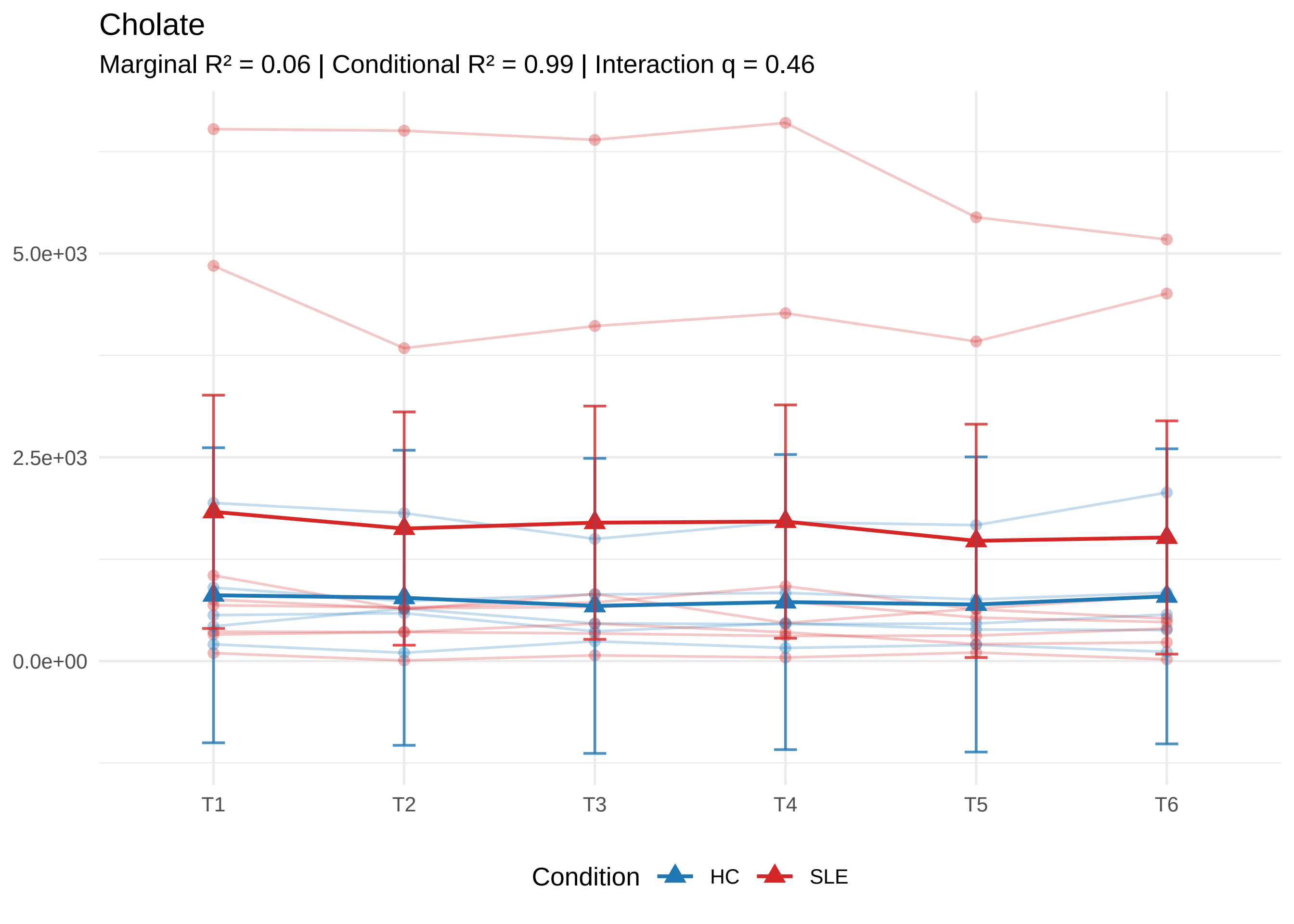
Cholate
Marginal R² = 0.06 | Conditional R² = 0.99 | Interaction q = 0.46
5.0e+03
2.5e+03
0.0e+00
T1
T2
T3
T4
T5
T6
Condition
HC
SLE

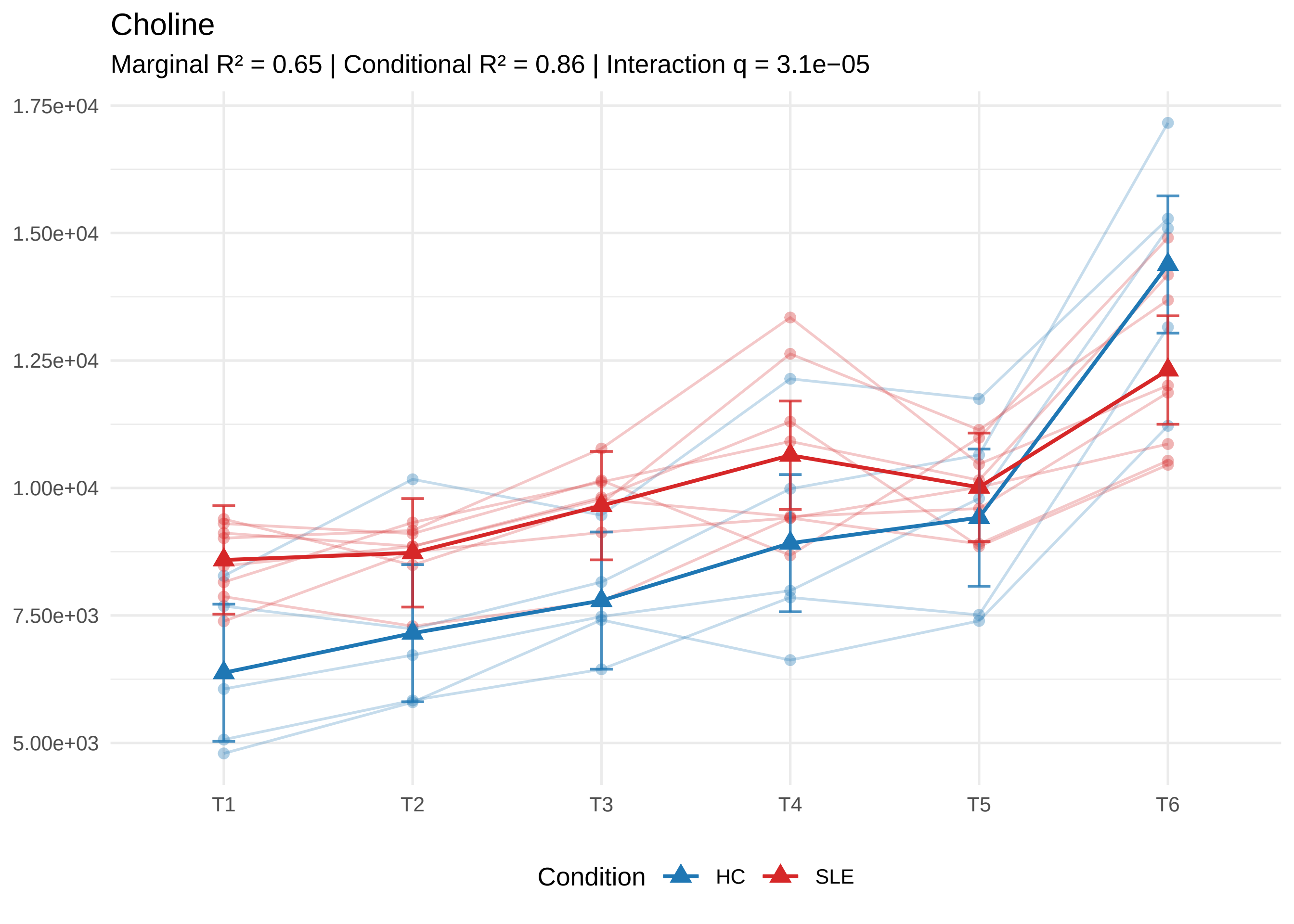

Choline
Marginal R² = 0.65 | Conditional R² = 0.86 | Interaction q = 3.1e−05
1.75e+04
1.50e+04
1.25e+04
1.00e+04
7.50e+03
5.00e+03
T1
T2
T3
T4
T5
T6
Condition
HC
SLE

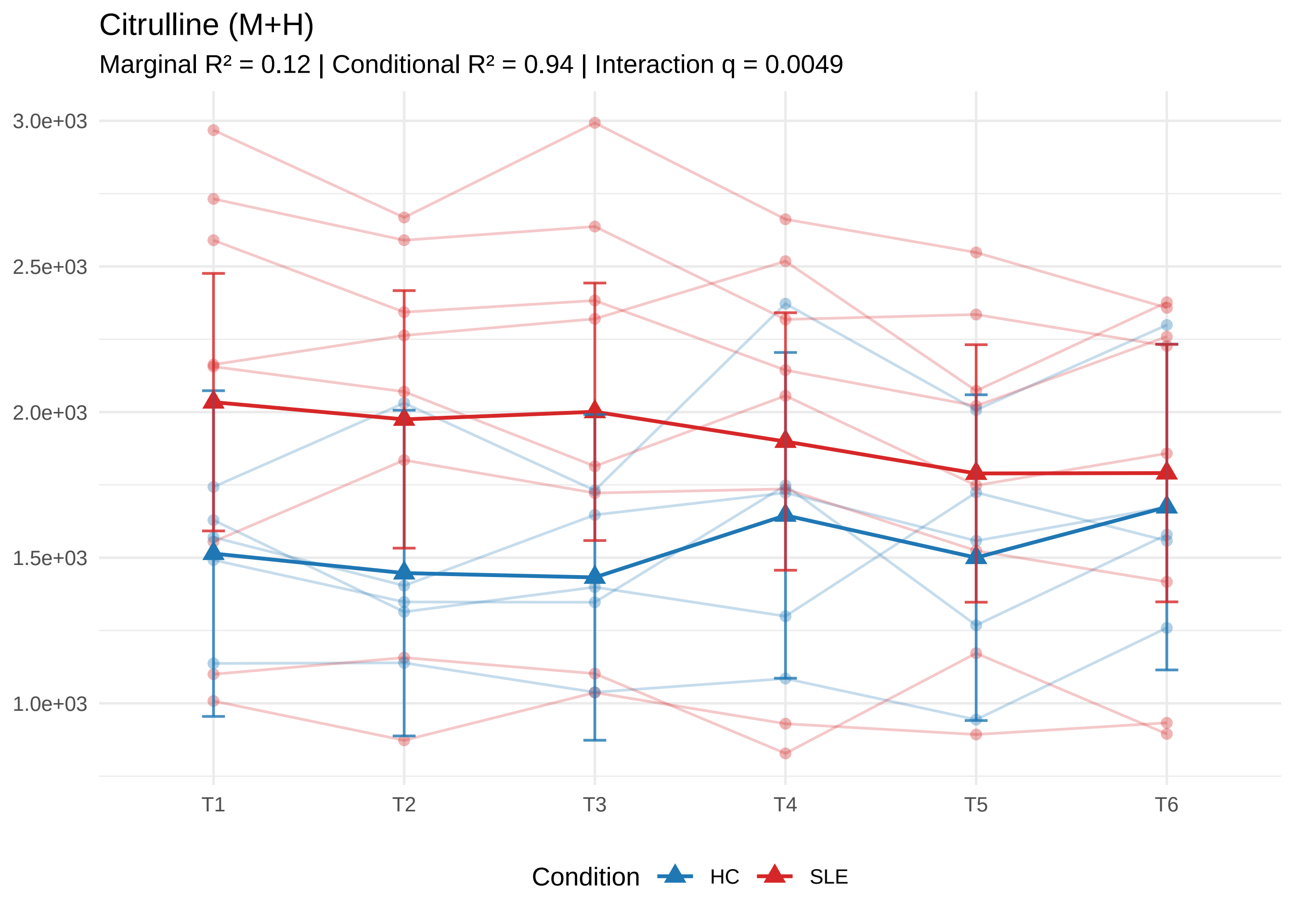

Citrulline (M+H)
Marginal R² = 0.12 | Conditional R² = 0.94 | Interaction q = 0.0049
3.0e+03
2.5e+03
2.0e+03
1.5e+03
1.0e+03
T1
T2
T3
T4
T5
T6
Condition
HC
SLE

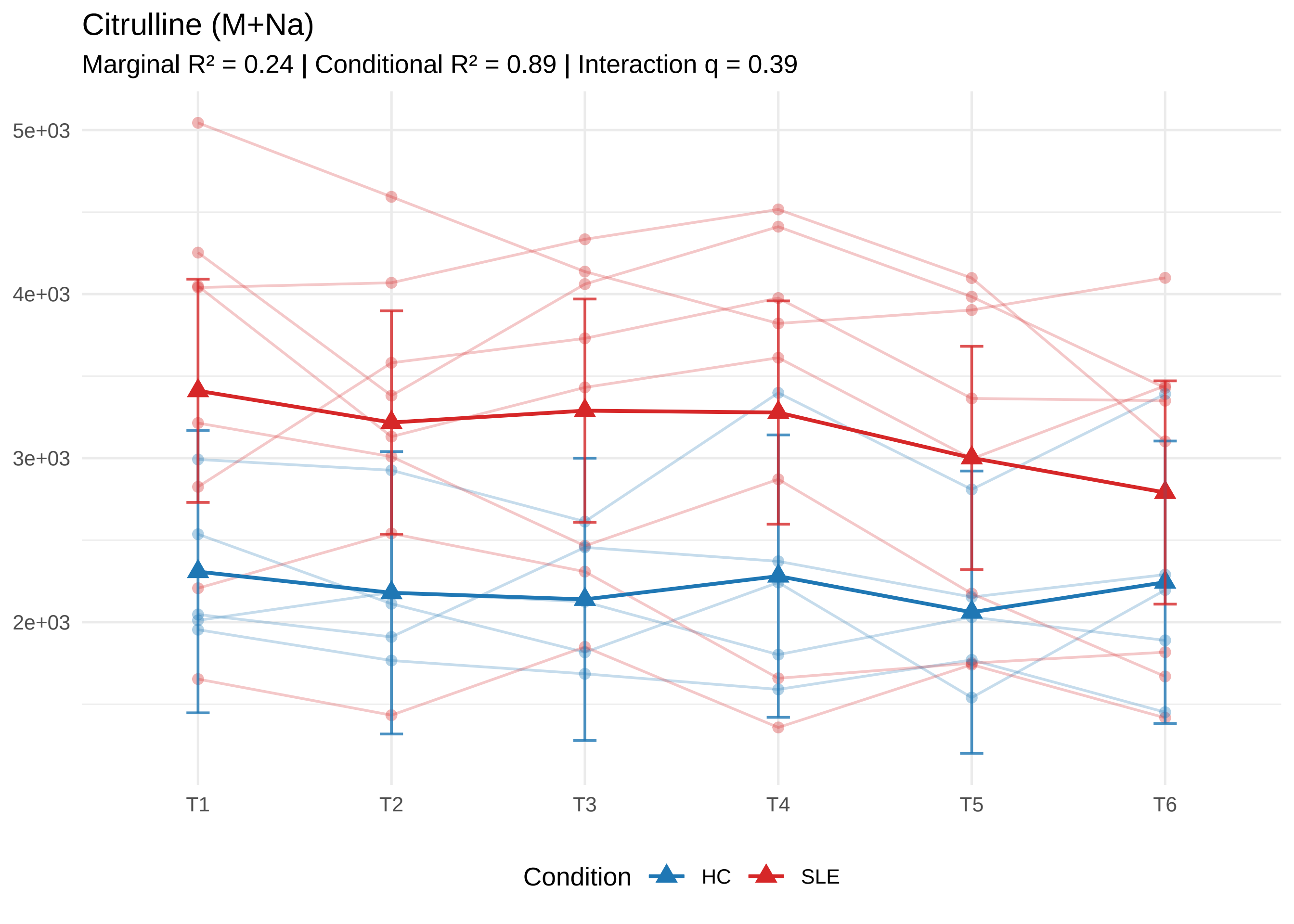
Citrulline (M+Na)
Marginal R² = 0.24 | Conditional R² = 0.89 | Interaction q = 0.39
5e+03
4e+03
3e+03
2e+03
T1
T2
T3
T4
T5
T6
Condition
HC
SLE

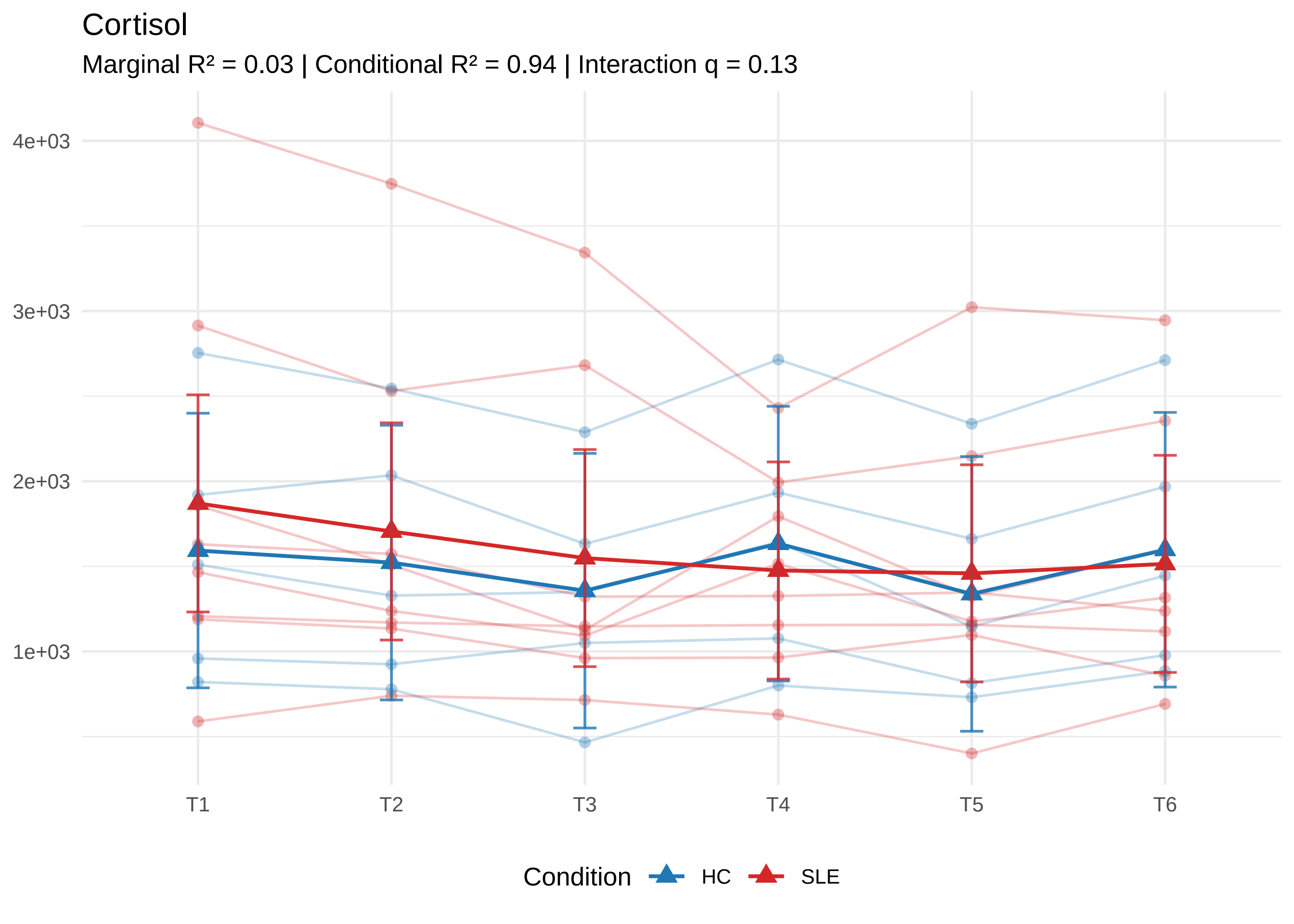
Cortisol
Marginal R² = 0.03 | Conditional R² = 0.94 | Interaction q = 0.13
4e+03
3e+03
2e+03
1e+03
T1
T2
T3
T4
T5
T6
Condition
HC
SLE

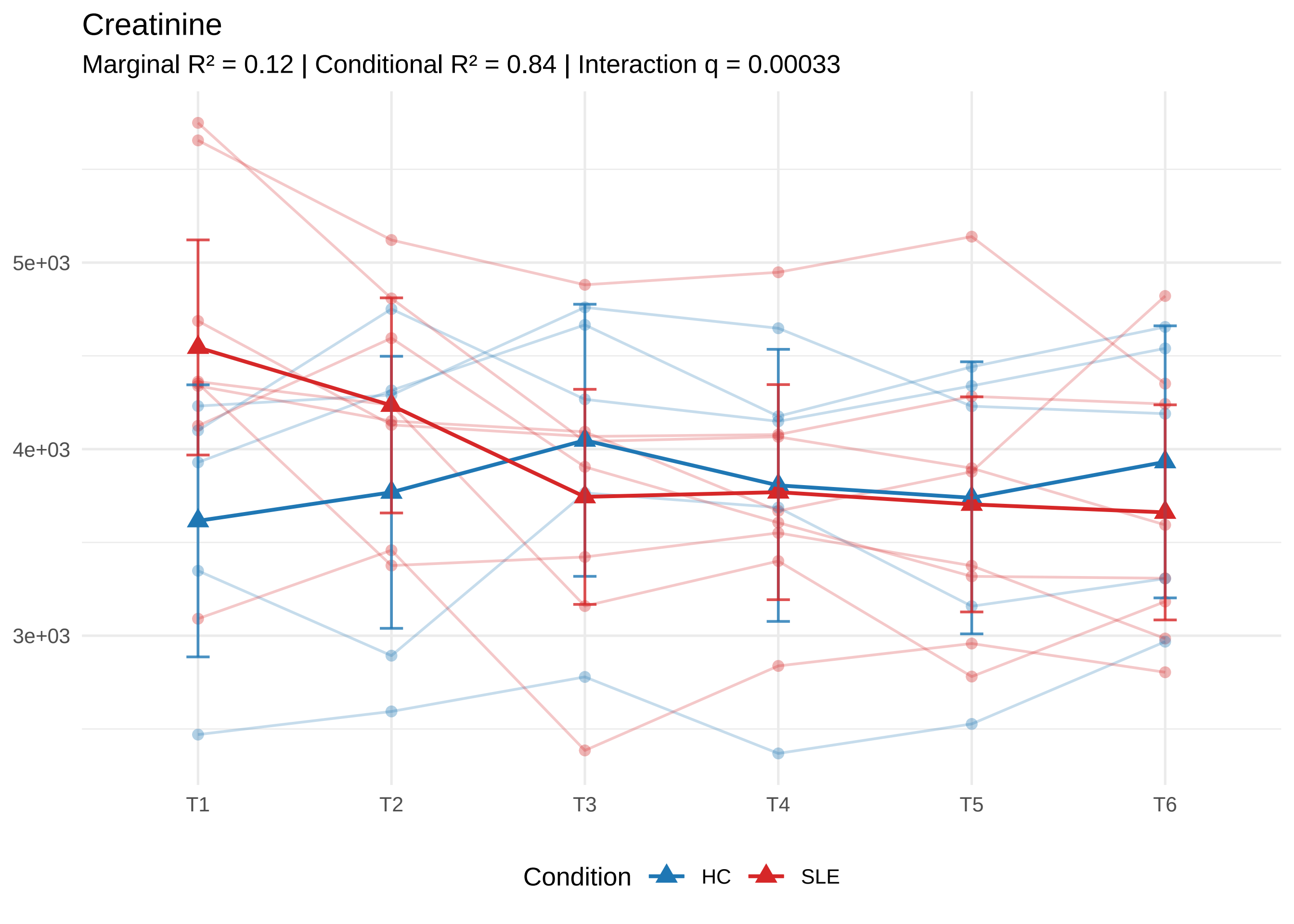

Creatinine
Marginal R² = 0.12 | Conditional R² = 0.84 | Interaction q = 0.00033
5e+03
4e+03
3e+03
T1
T2
T3
T4
T5
T6
Condition
HC
SLE

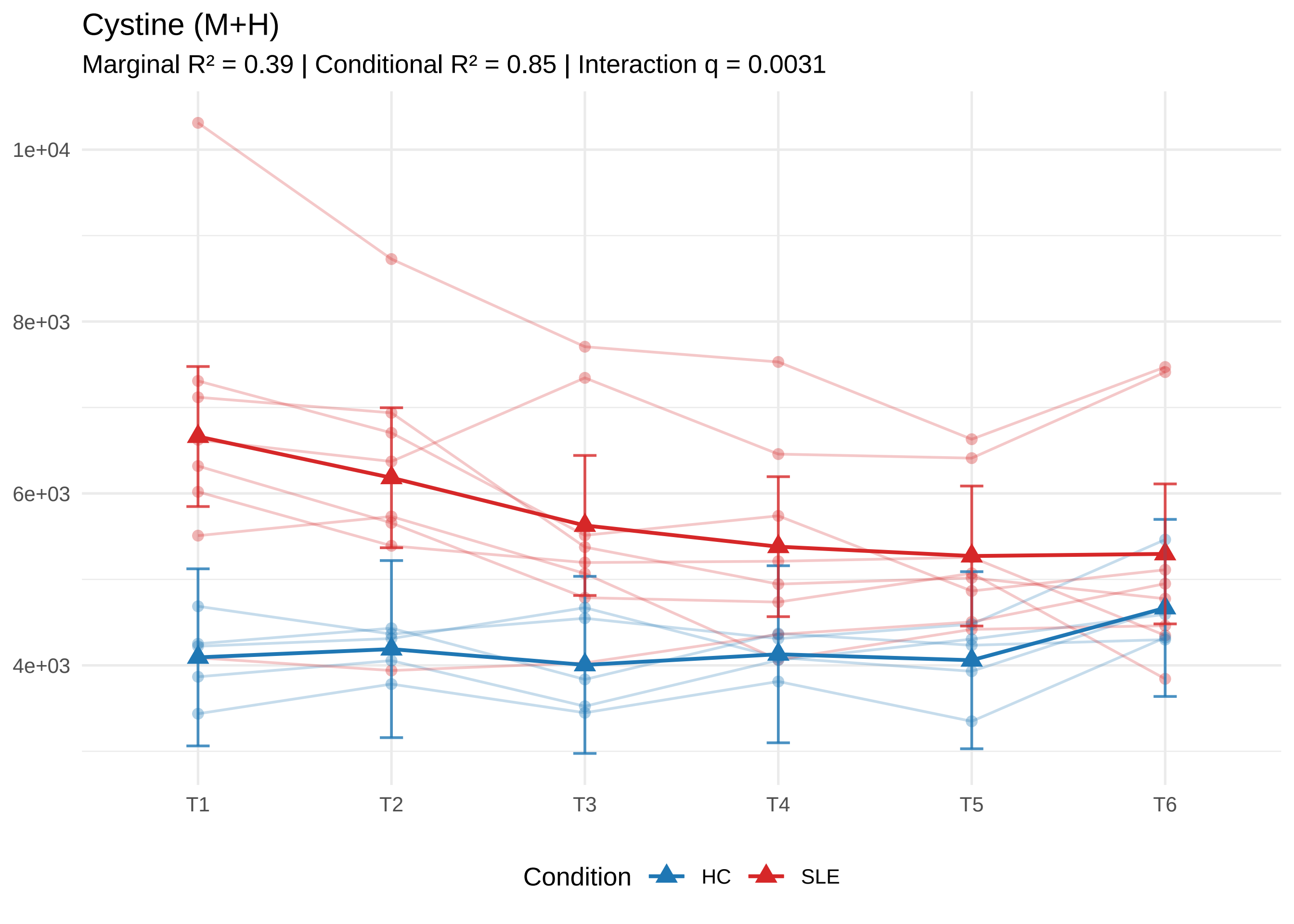

Cystine (M+H)
Marginal R² = 0.39 | Conditional R² = 0.85 | Interaction q = 0.0031
1e+04
8e+03
6e+03
4e+03
T1
T2
T3
T4
T5
T6
Condition
HC
SLE

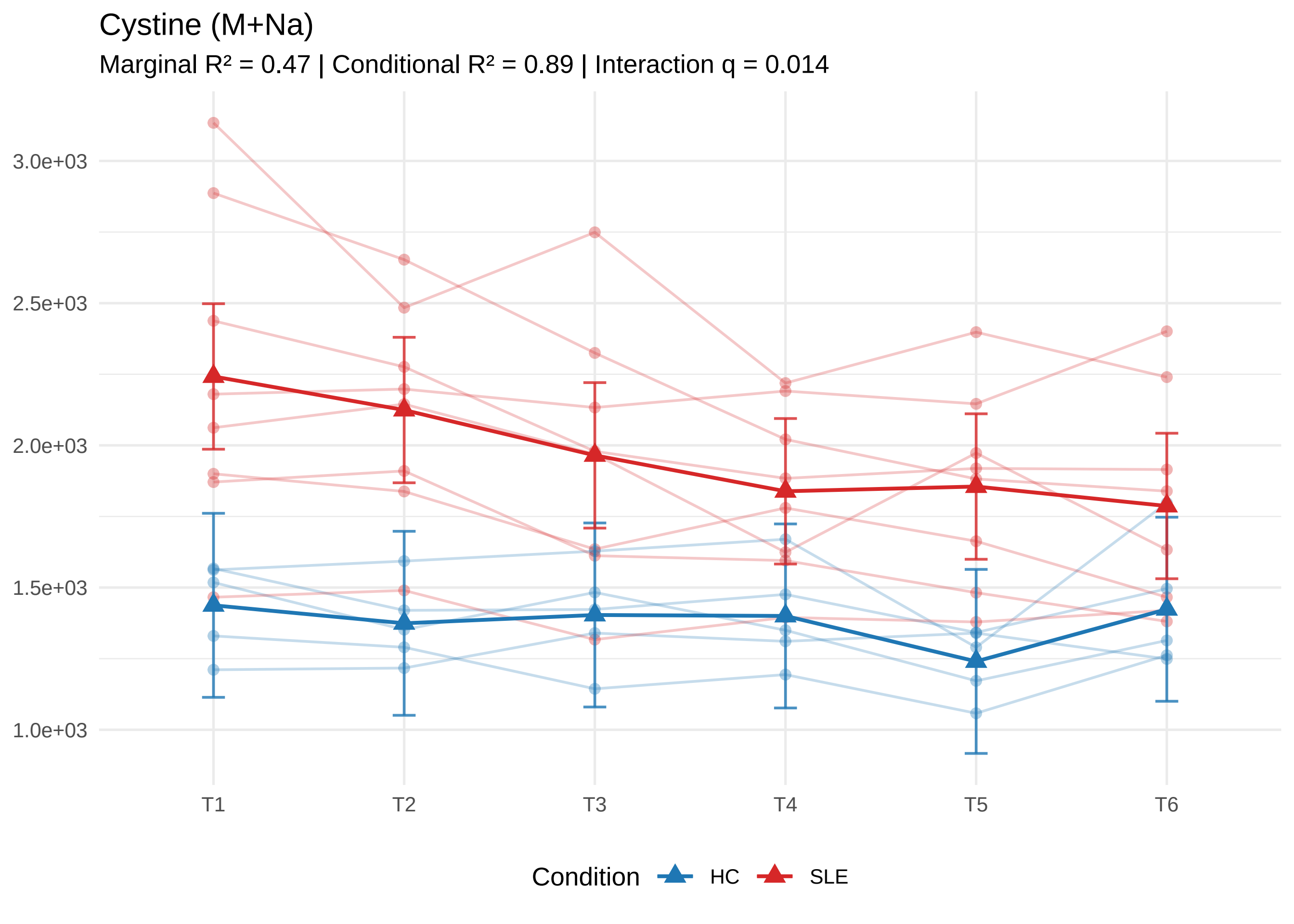

Cystine (M+Na)
Marginal R² = 0.47 | Conditional R² = 0.89 | Interaction q = 0.014
3.0e+03
2.5e+03
2.0e+03
1.5e+03
1.0e+03
T1
T2
T3
T4
T5
T6
Condition
HC
SLE

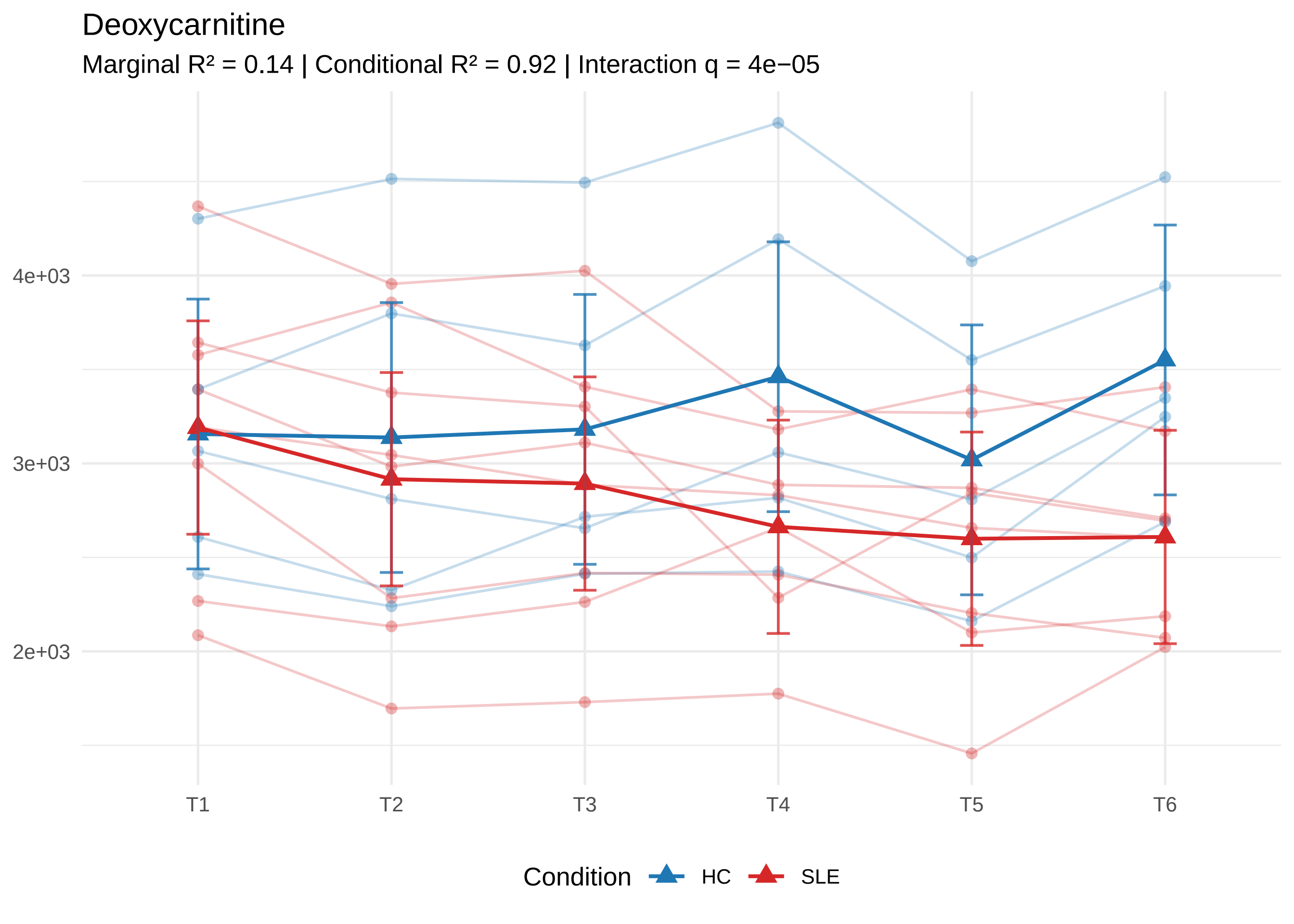

Deoxycarnitine
Marginal R² = 0.14 | Conditional R² = 0.92 | Interaction q = 4e−05
4e+03
3e+03
2e+03
T1
T2
T3
T4
T5
T6
Condition
HC
SLE

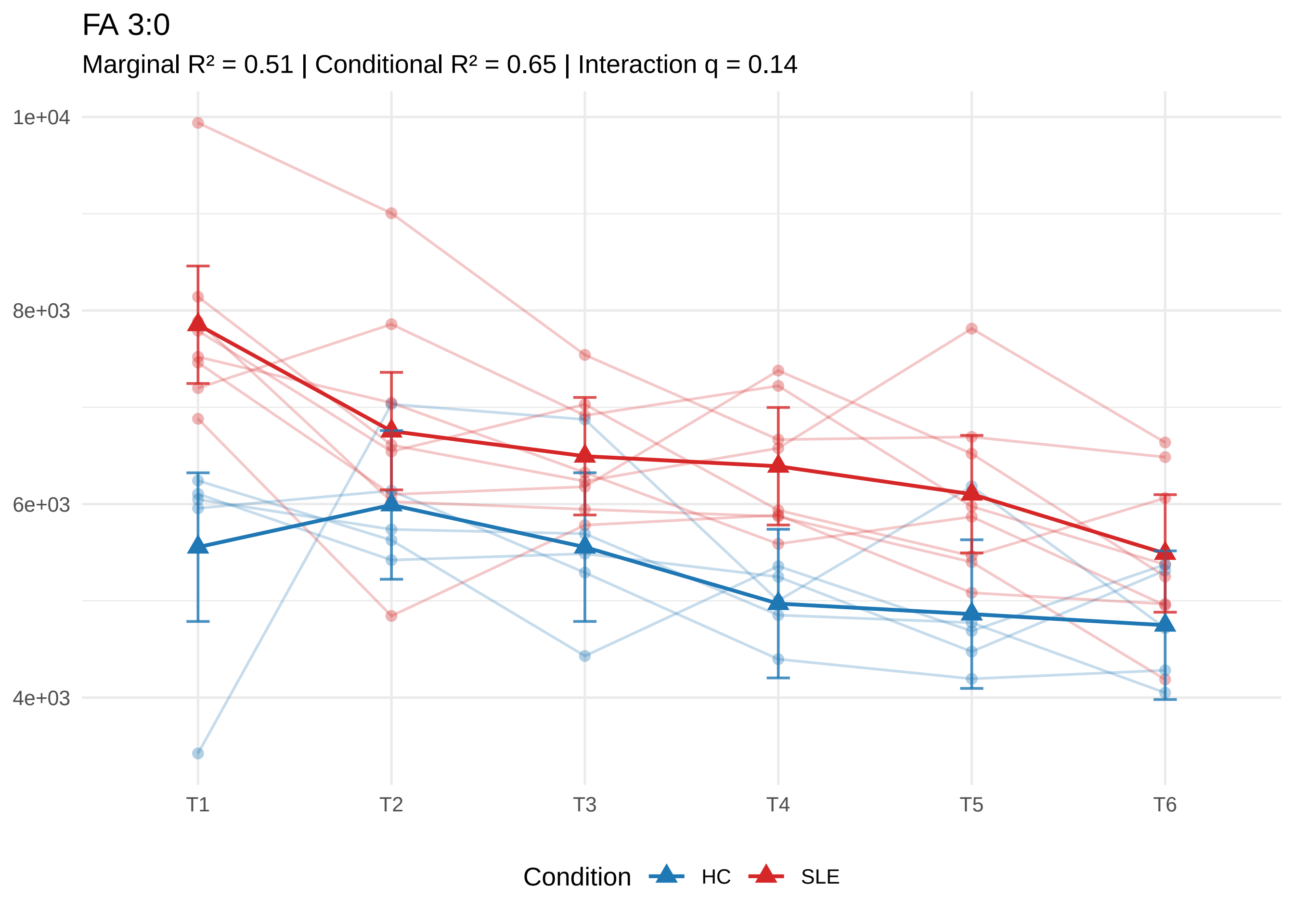

FA 3:0
Marginal R² = 0.51 | Conditional R² = 0.65 | Interaction q = 0.14
1e+04
8e+03
6e+03
4e+03
T1
T2
T3
T4
T5
T6
Condition
HC
SLE

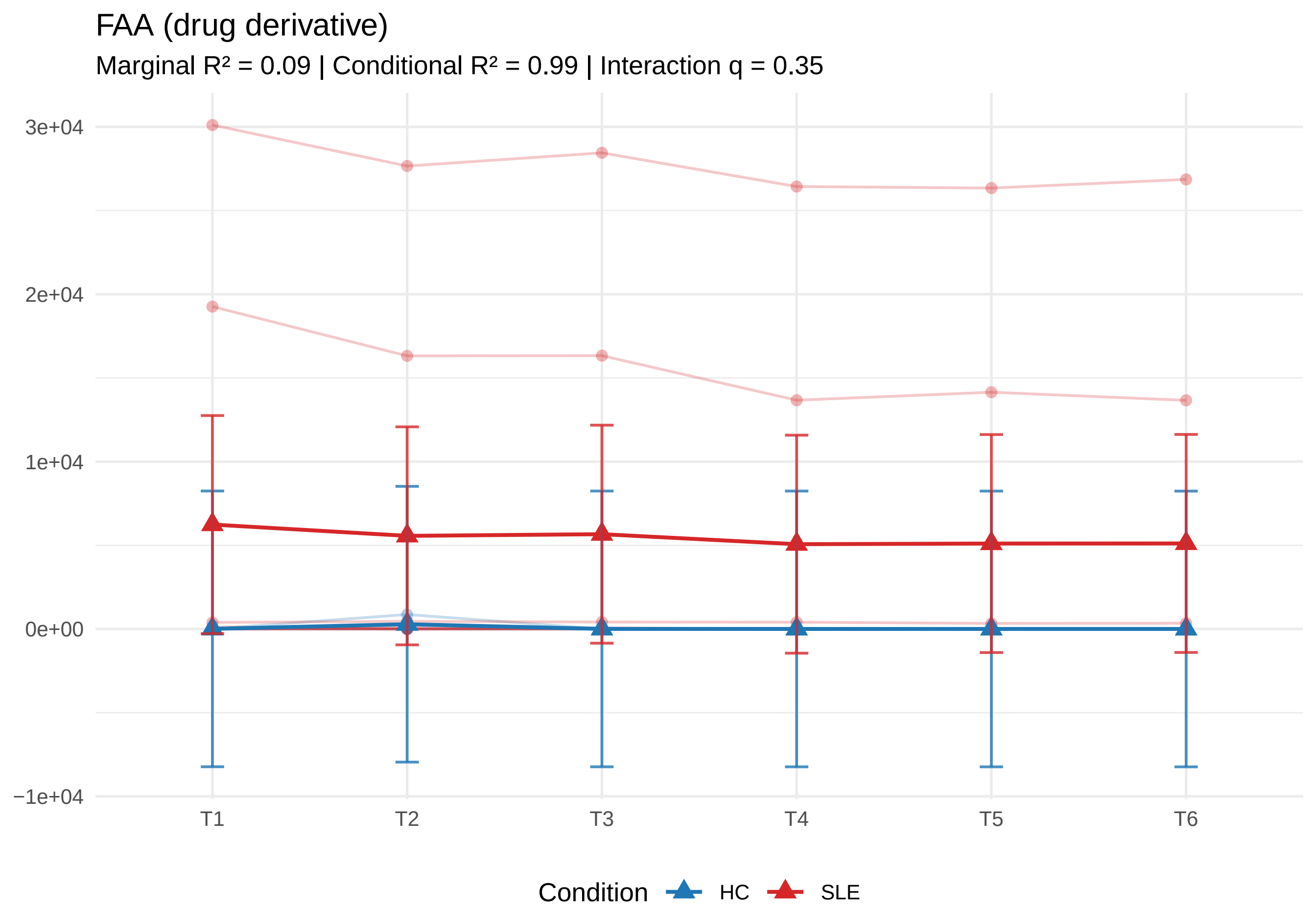
FAA (drug derivative)
Marginal R² = 0.09 | Conditional R² = 0.99 | Interaction q = 0.35
3e+04
2e+04
1e+04
0e+00
−1e+04
T1
T2
T3
T4
T5
T6
Condition
HC
SLE

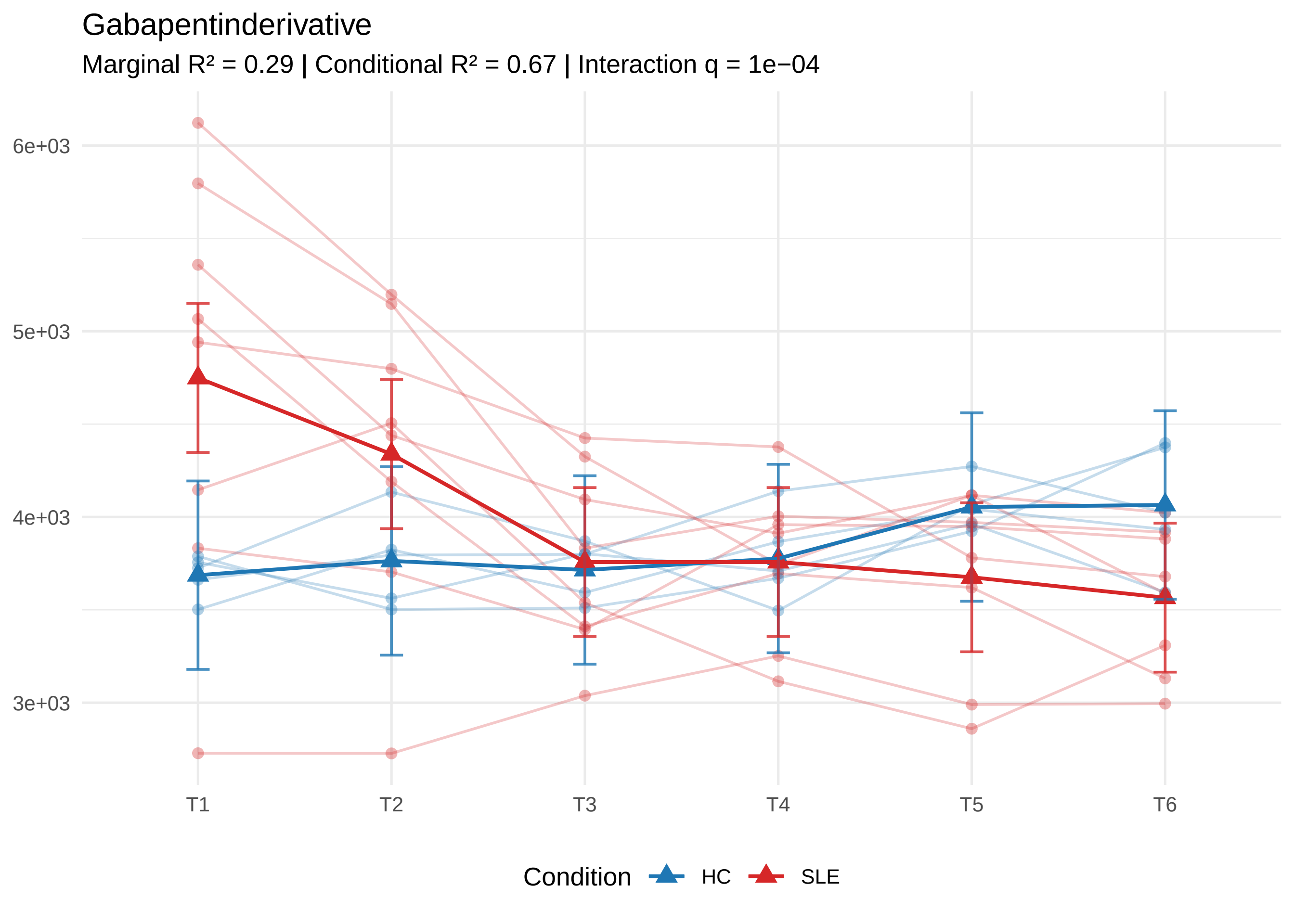
Gabapentinderivative
Marginal R² = 0.29 | Conditional R² = 0.67 | Interaction q = 1e−04
6e+03
5e+03
4e+03
3e+03
T1
T2
T3
T4
T5
T6
Condition
HC
SLE

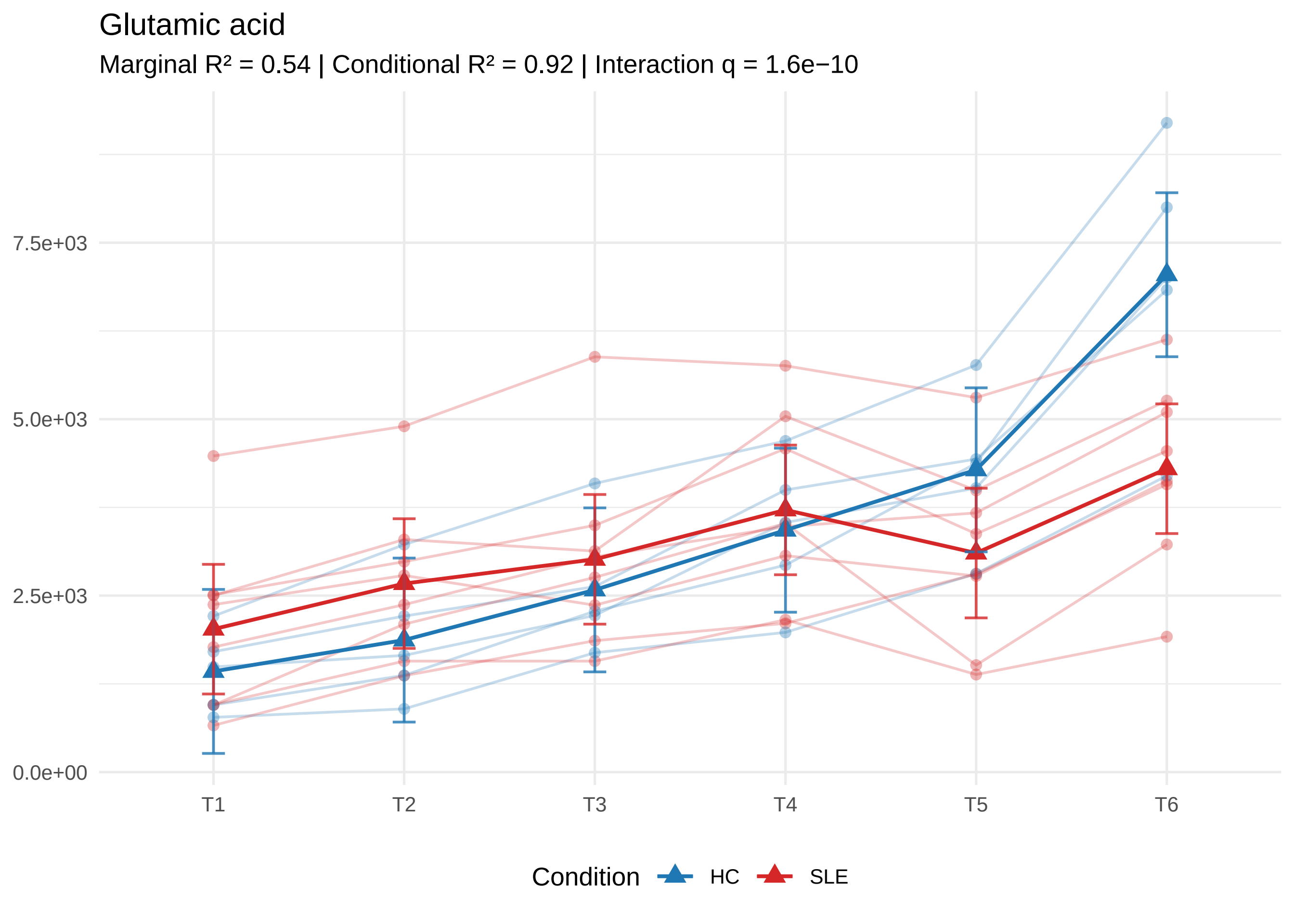

Glutamic acid
Marginal R² = 0.54 | Conditional R² = 0.92 | Interaction q = 1.6e−10
7.5e+03
5.0e+03
2.5e+03
0.0e+00
T1
T2
T3
T4
T5
T6
Condition
HC
SLE

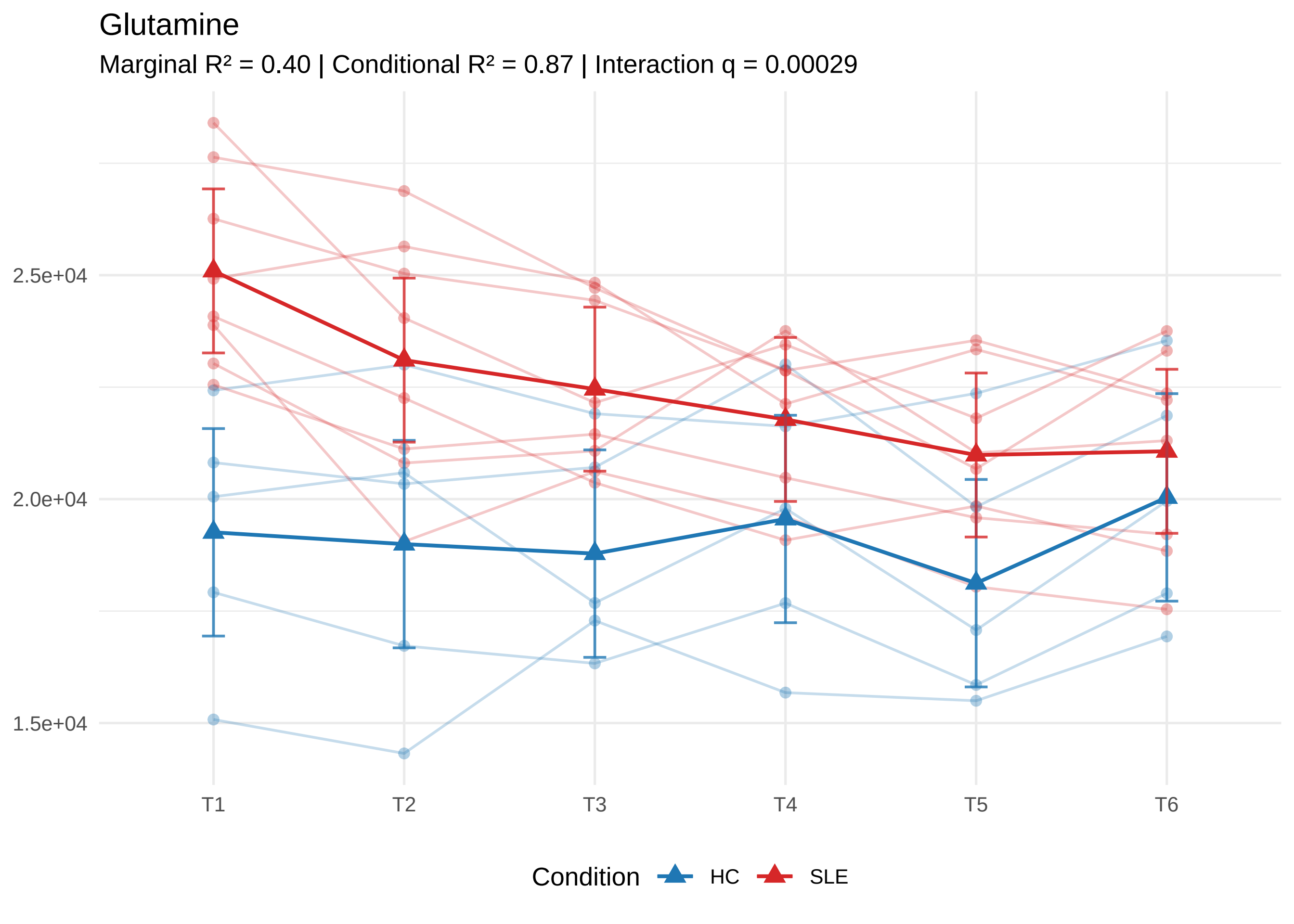
Glutamine
Marginal R² = 0.40 | Conditional R² = 0.87 | Interaction q = 0.00029
2.5e+04
2.0e+04
1.5e+04
T1
T2
T3
T4
T5
T6
Condition
HC
SLE

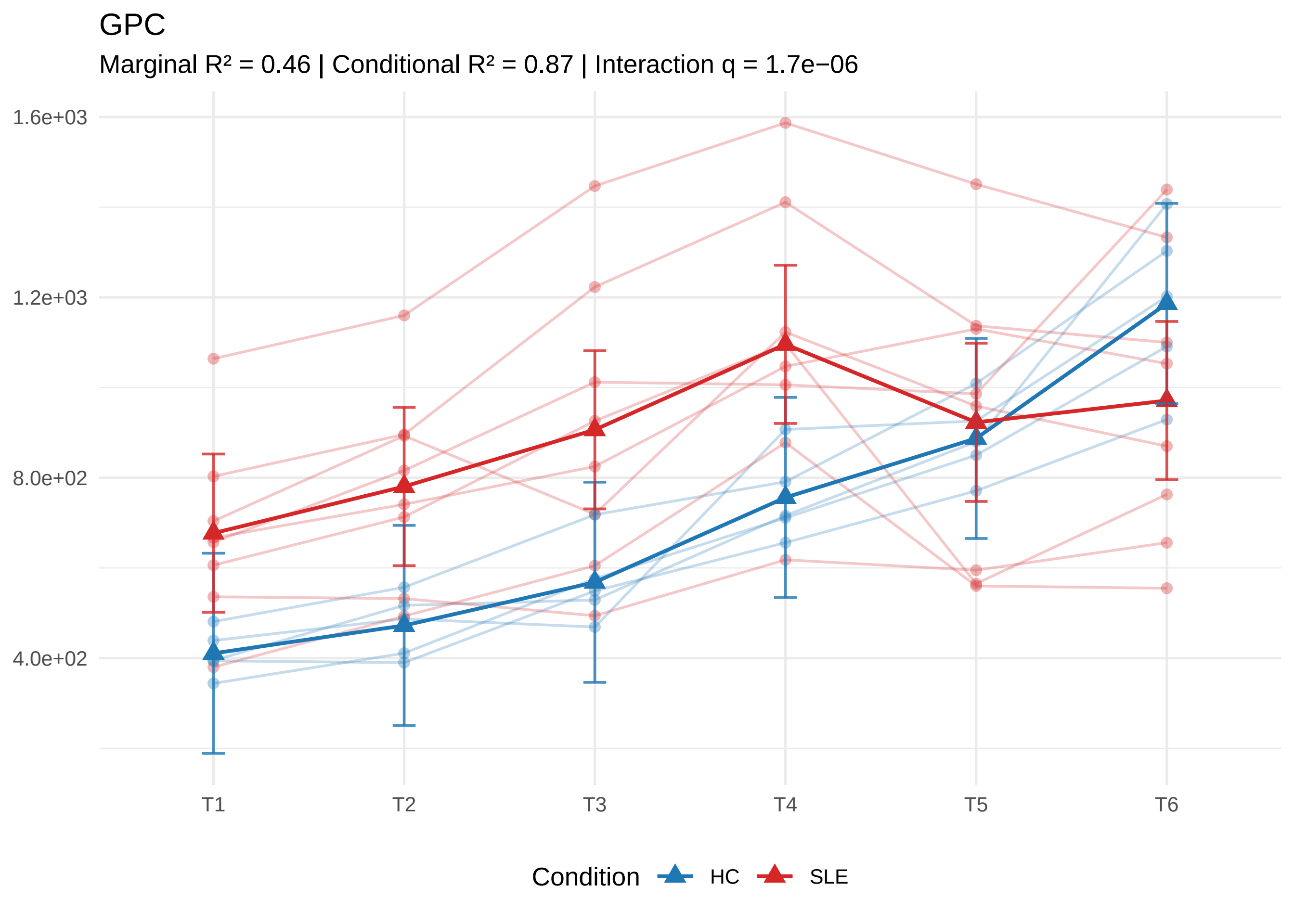
GPC
Marginal R² = 0.46 | Conditional R² = 0.87 | Interaction q = 1.7e−06
1.6e+03
1.2e+03
8.0e+02
4.0e+02
T1
T2
T3
T4
T5
T6
Condition
HC
SLE

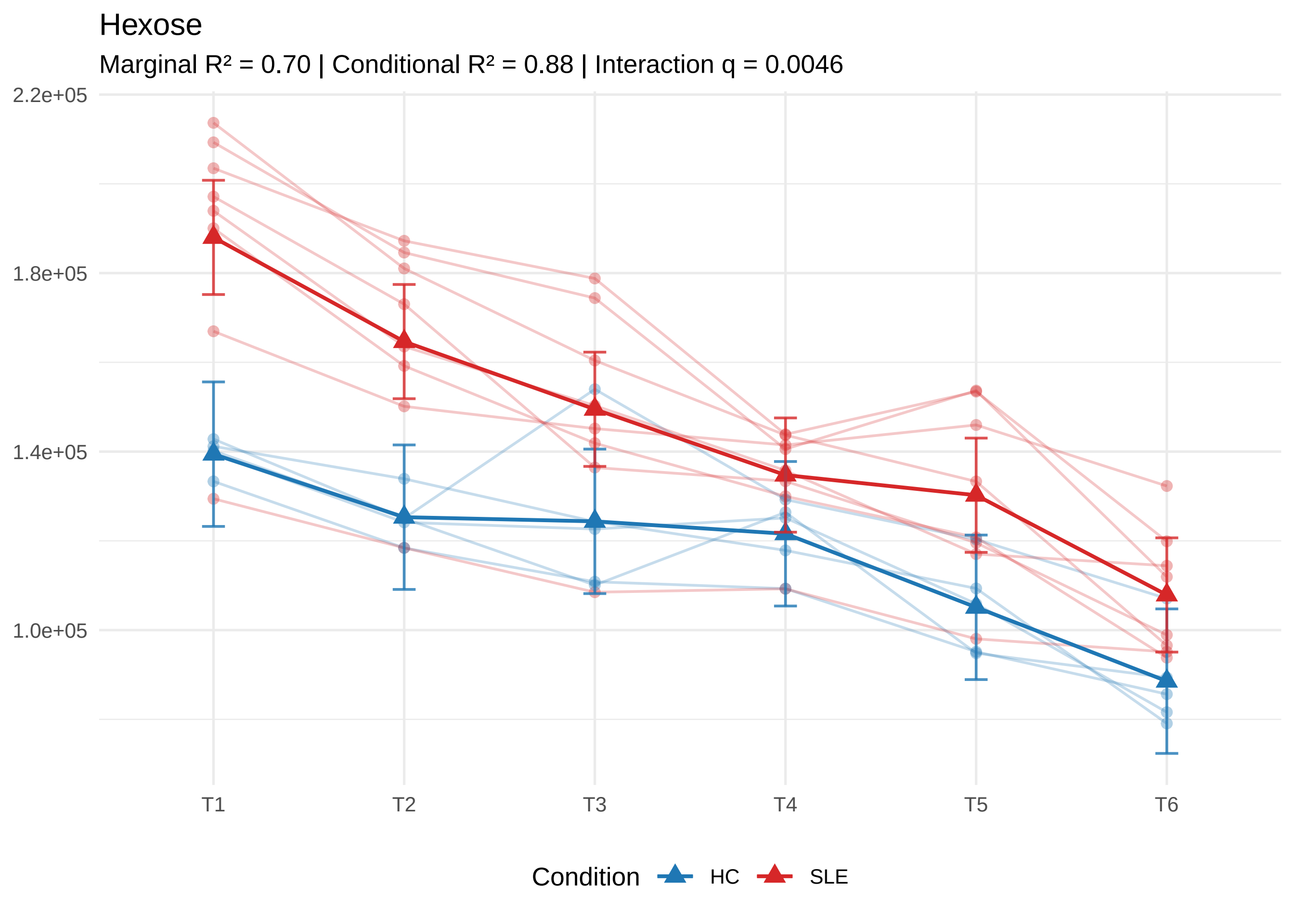
Hexose
Marginal R² = 0.70 | Conditional R² = 0.88 | Interaction q = 0.0046
2.2e+05
1.8e+05
1.4e+05
1.0e+05
T1
T2
T3
T4
T5
T6
Condition
HC
SLE

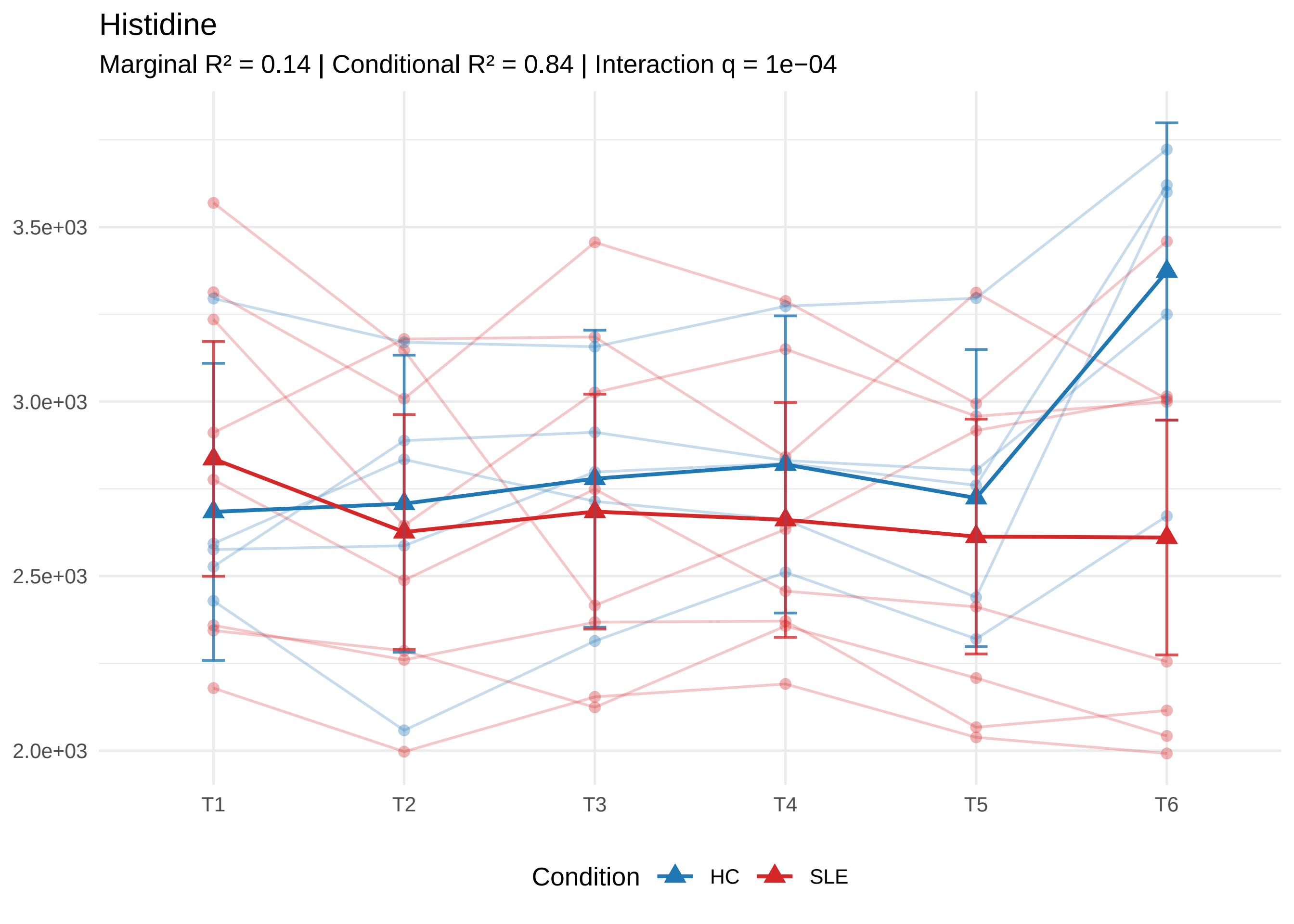
Histidine
Marginal R² = 0.14 | Conditional R² = 0.84 | Interaction q = 1e−04
3.5e+03
3.0e+03
2.5e+03
2.0e+03
T1
T2
T3
T4
T5
T6
Condition
HC
SLE

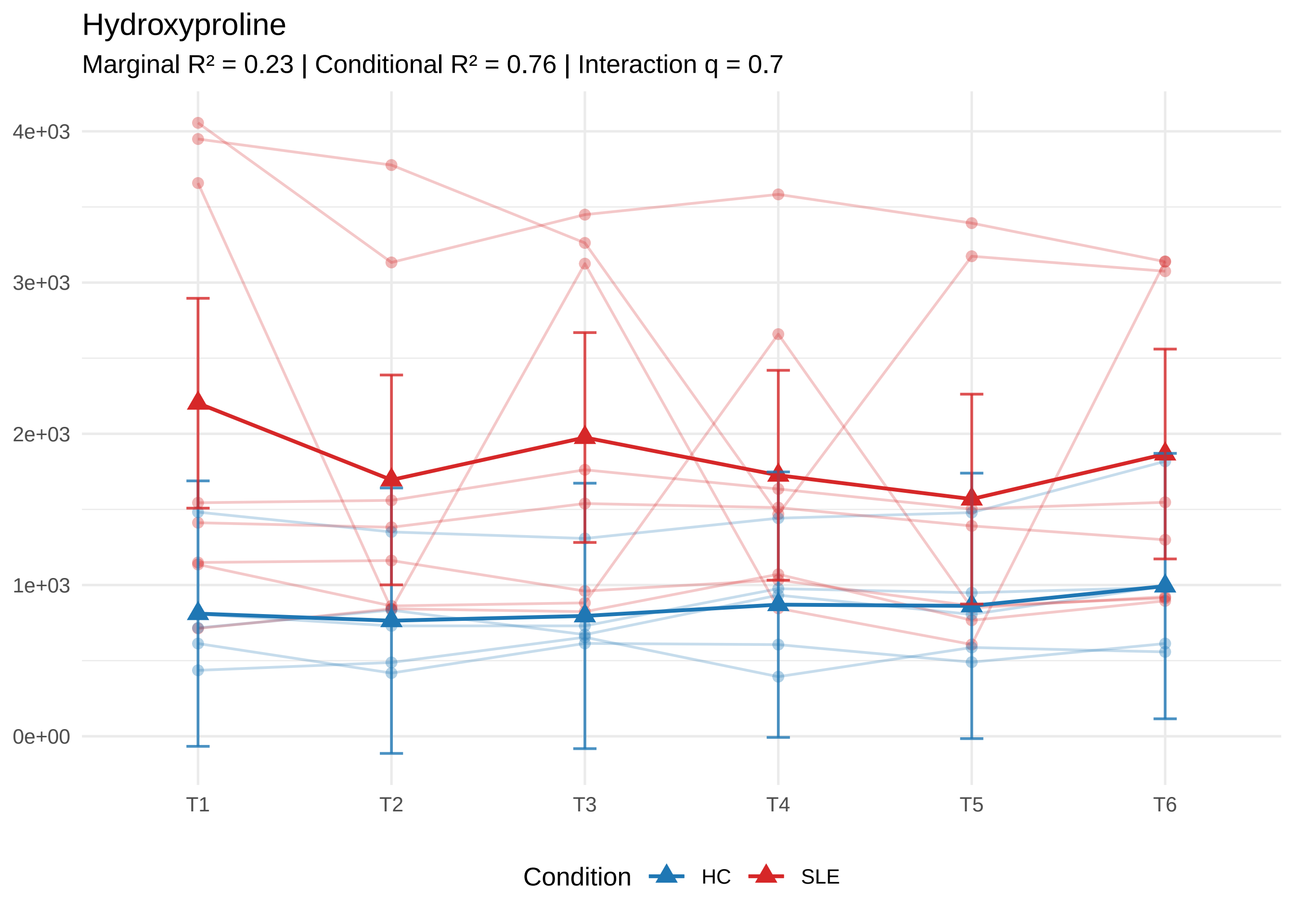
Hydroxyproline
Marginal R² = 0.23 | Conditional R² = 0.76 | Interaction q = 0.7
4e+03
3e+03
2e+03
1e+03
0e+00
T1
T2
T3
T4
T5
T6
Condition
HC
SLE

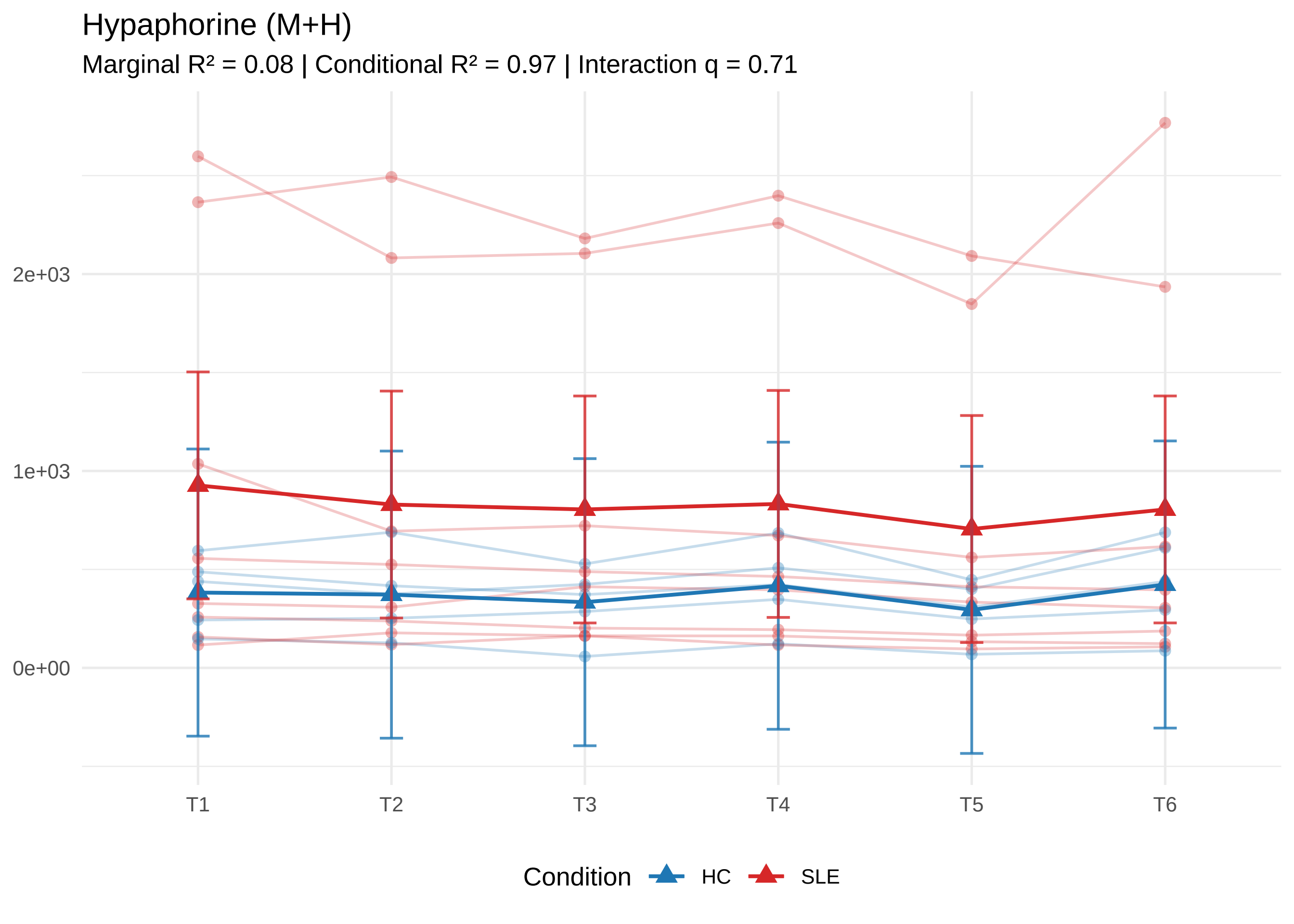
Hypaphorine (M+H)
Marginal R² = 0.08 | Conditional R² = 0.97 | Interaction q = 0.71
2e+03
1e+03
0e+00
T1
T2
T3
T4
T5
T6
Condition
HC
SLE

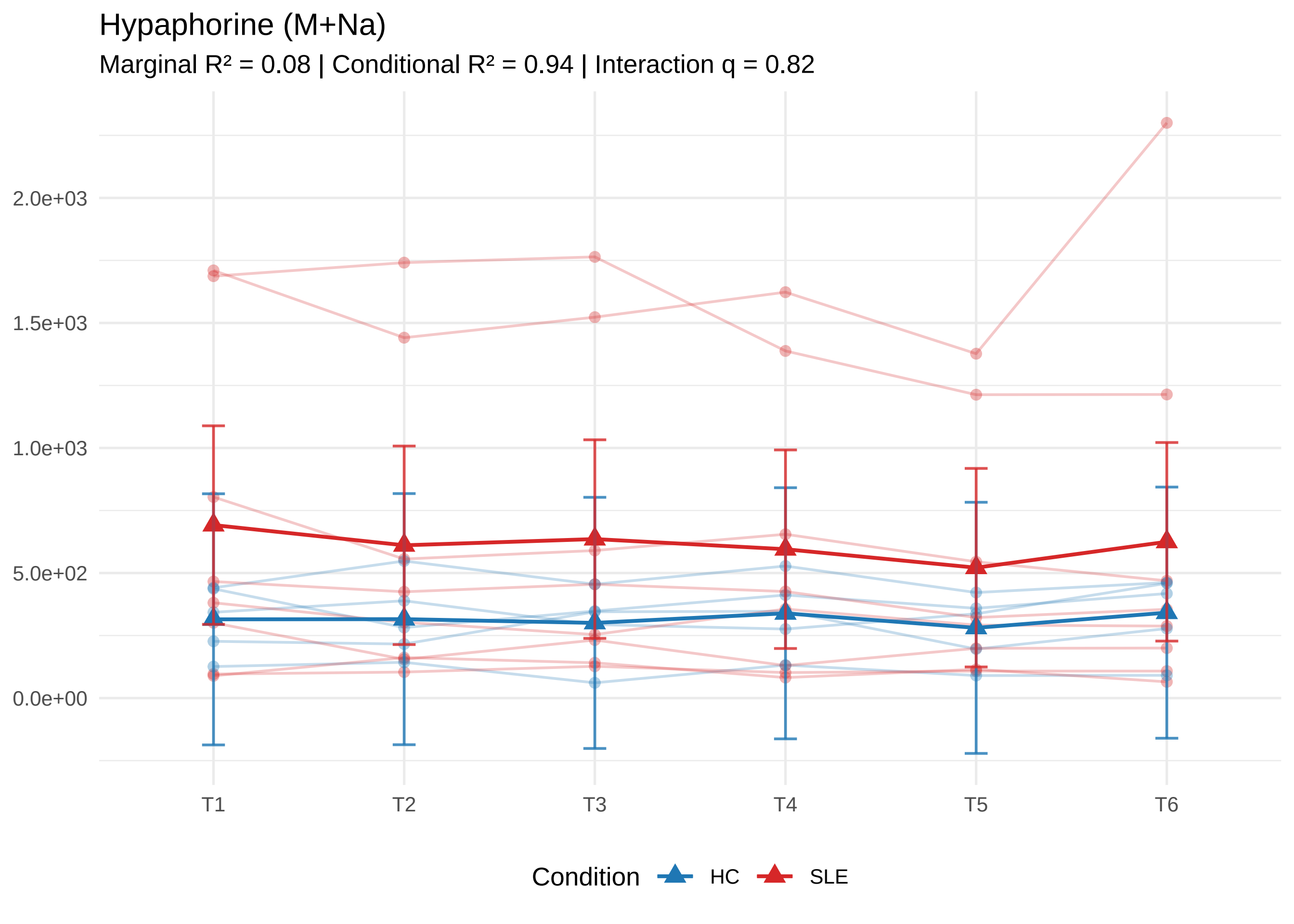

Hypaphorine (M+Na)
Marginal R² = 0.08 | Conditional R² = 0.94 | Interaction q = 0.82
2.0e+03
1.5e+03
1.0e+03
5.0e+02
0.0e+00
T1
T2
T3
T4
T5
T6
Condition
HC
SLE

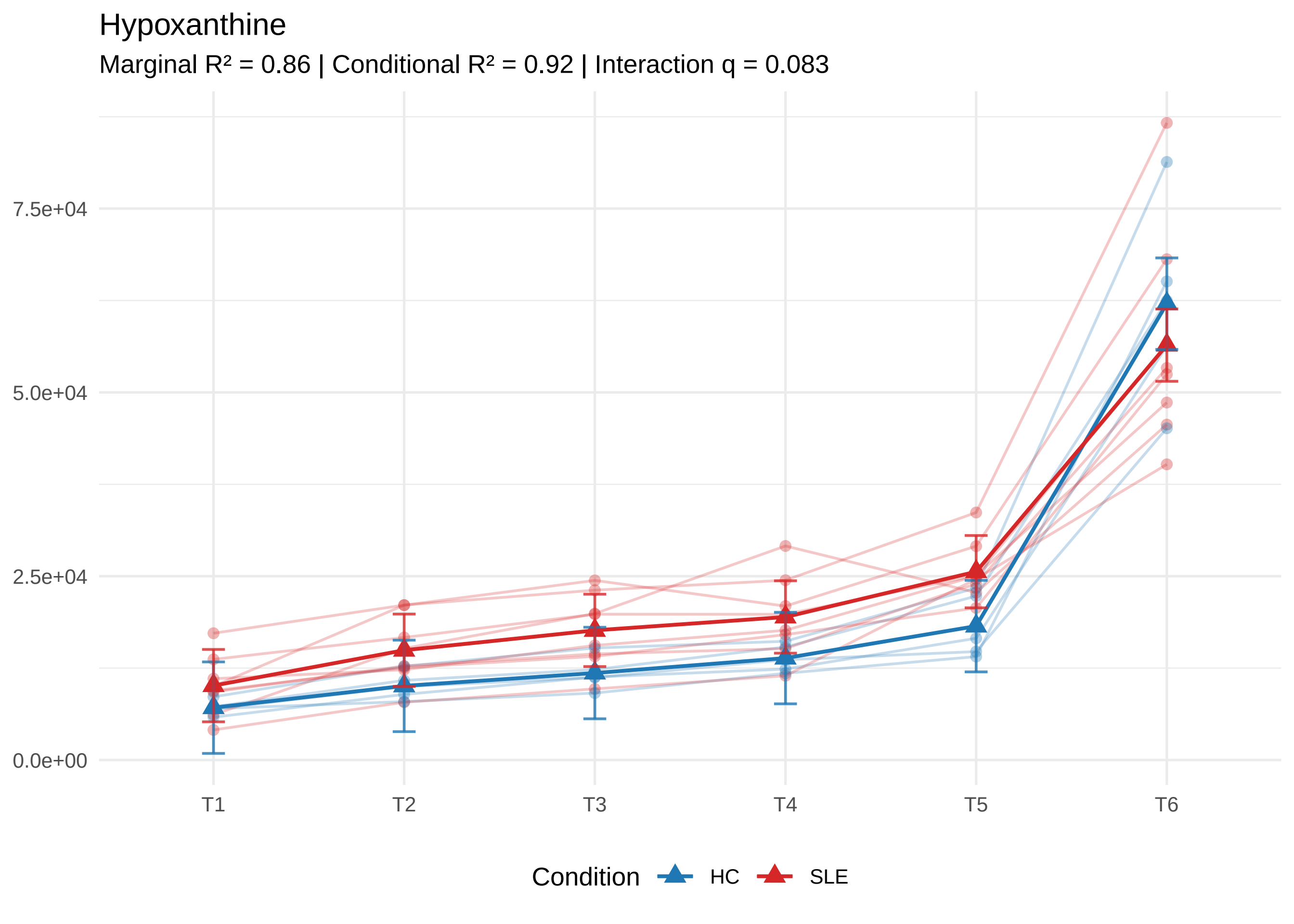

Hypoxanthine
Marginal R² = 0.86 | Conditional R² = 0.92 | Interaction q = 0.083
7.5e+04
5.0e+04
2.5e+04
0.0e+00
T1
T2
T3
T4
T5
T6
Condition
HC
SLE

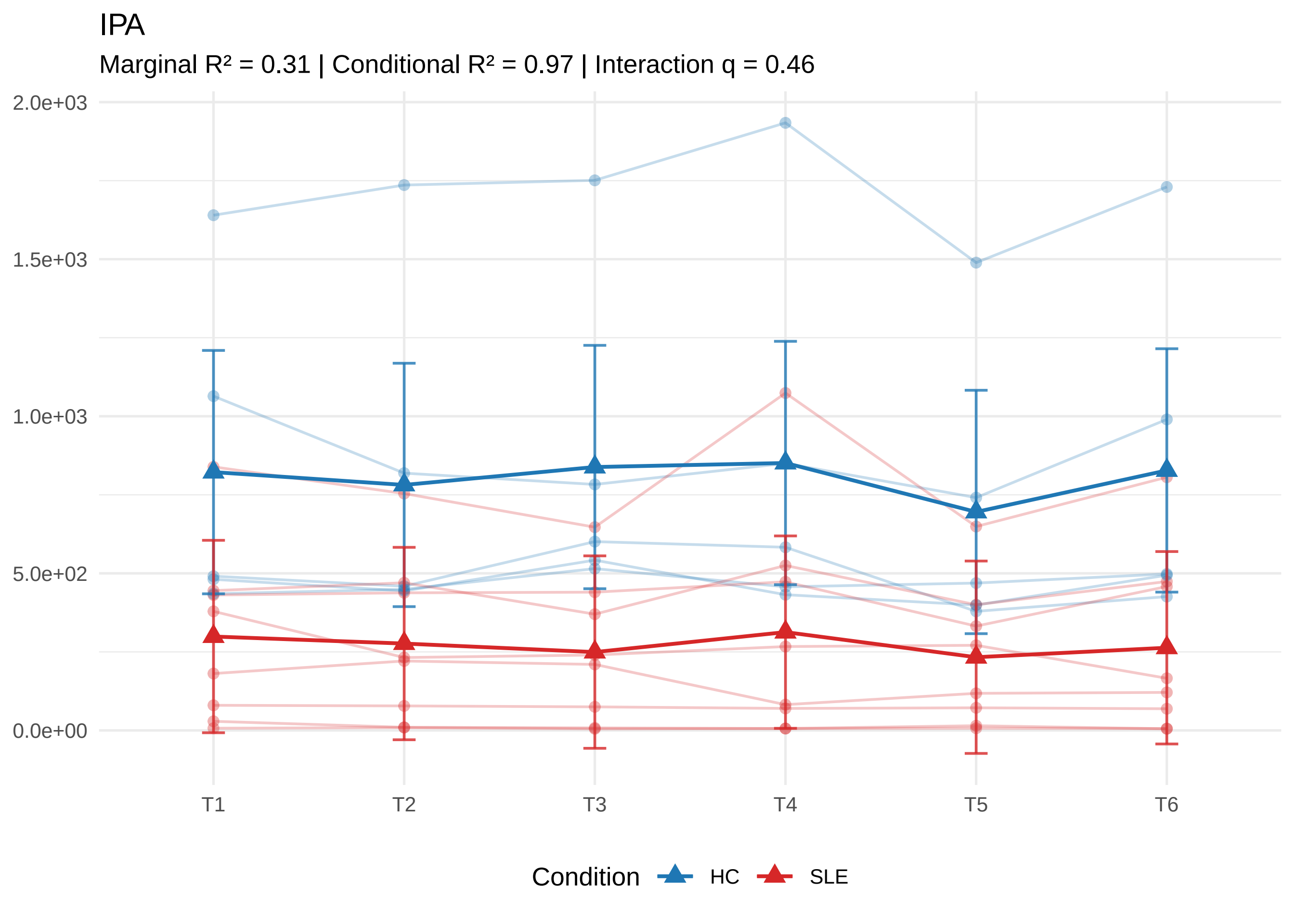

IPA
Marginal R² = 0.31 | Conditional R² = 0.97 | Interaction q = 0.46
2.0e+03
1.5e+03
1.0e+03
5.0e+02
0.0e+00
T1
T2
T3
T4
T5
T6
Condition
HC
SLE

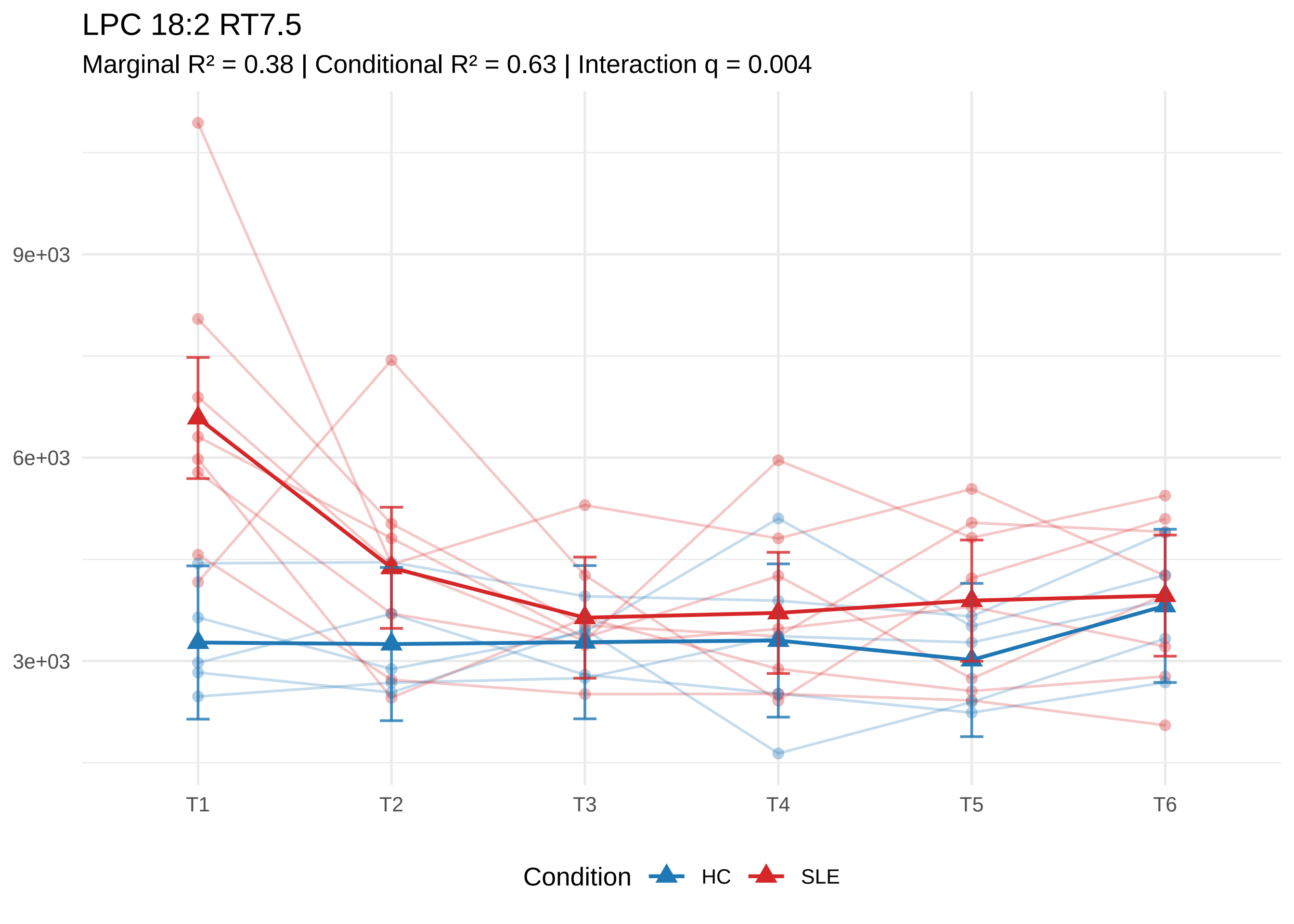

LPC 18:2 RT7.5
Marginal R² = 0.38 | Conditional R² = 0.63 | Interaction q = 0.004
9e+03
6e+03
3e+03
T1
T2
T3
T4
T5
T6
Condition
HC
SLE

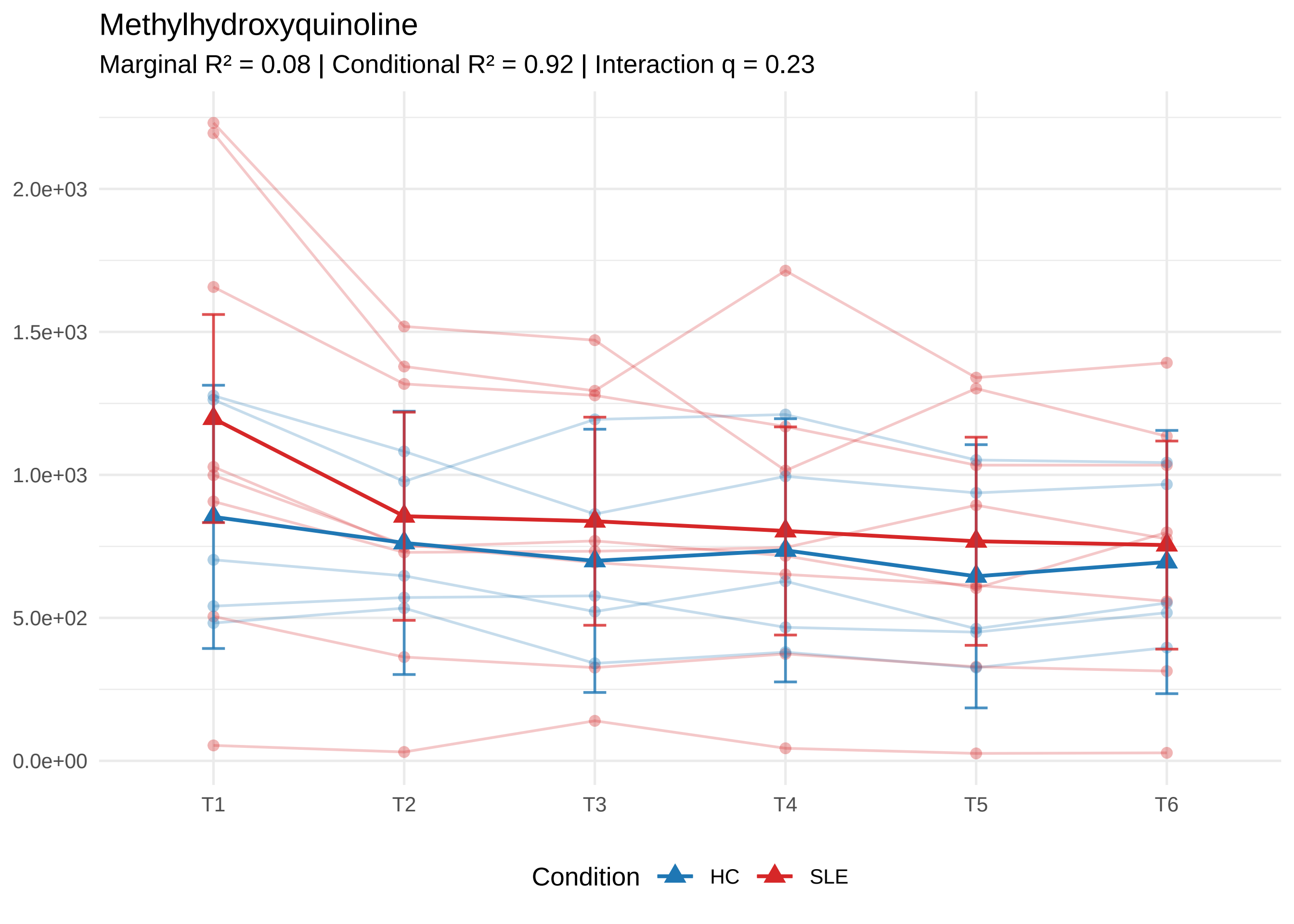
Methylhydroxyquinoline
Marginal R² = 0.08 | Conditional R² = 0.92 | Interaction q = 0.23
2.0e+03
1.5e+03
1.0e+03
5.0e+02
0.0e+00
T1
T2
T3
T4
T5
T6
Condition
HC
SLE

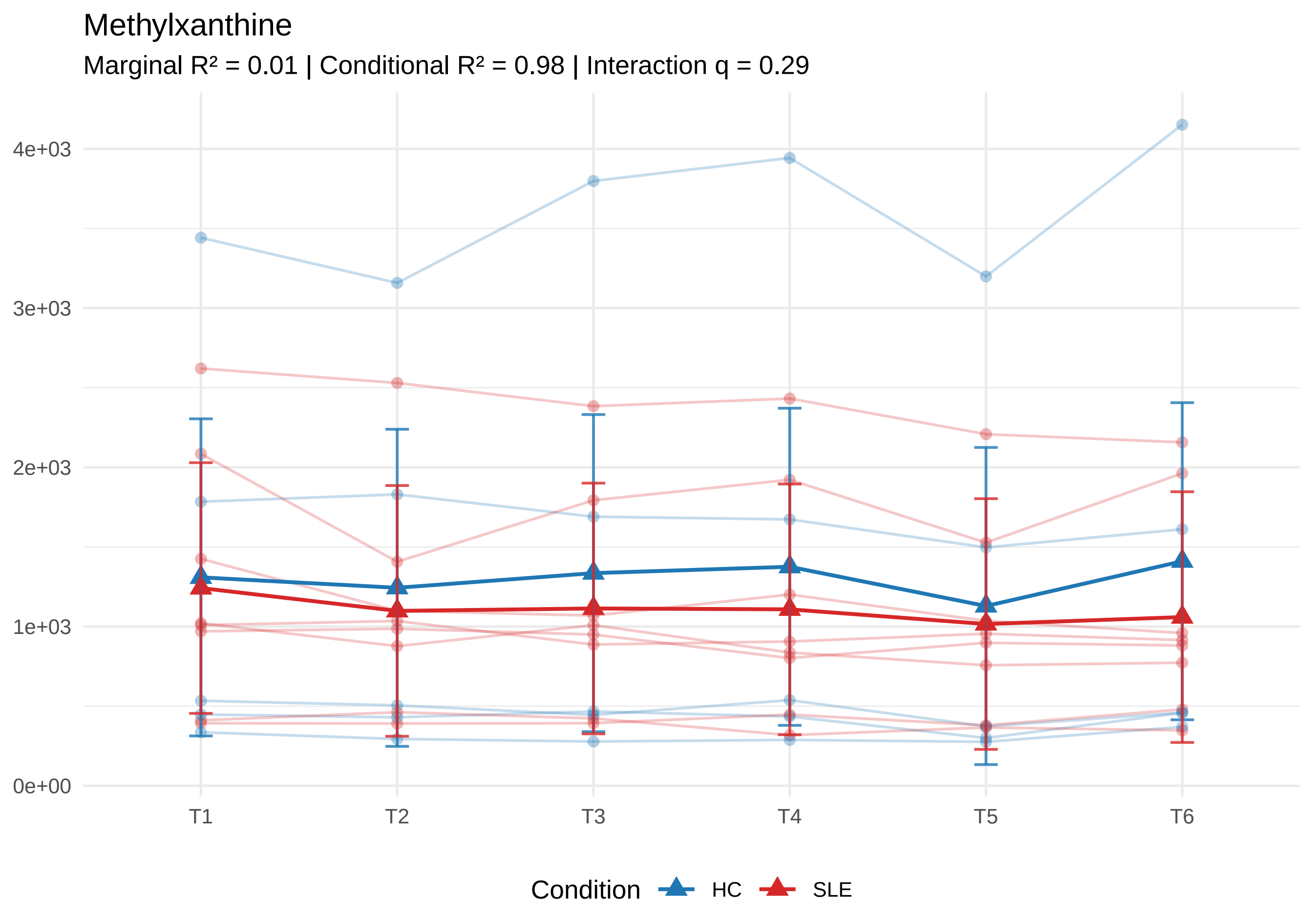
Methylxanthine
Marginal R² = 0.01 | Conditional R² = 0.98 | Interaction q = 0.29
4e+03
3e+03
2e+03
1e+03
0e+00
T1
T2
T3
T4
T5
T6
Condition
HC
SLE

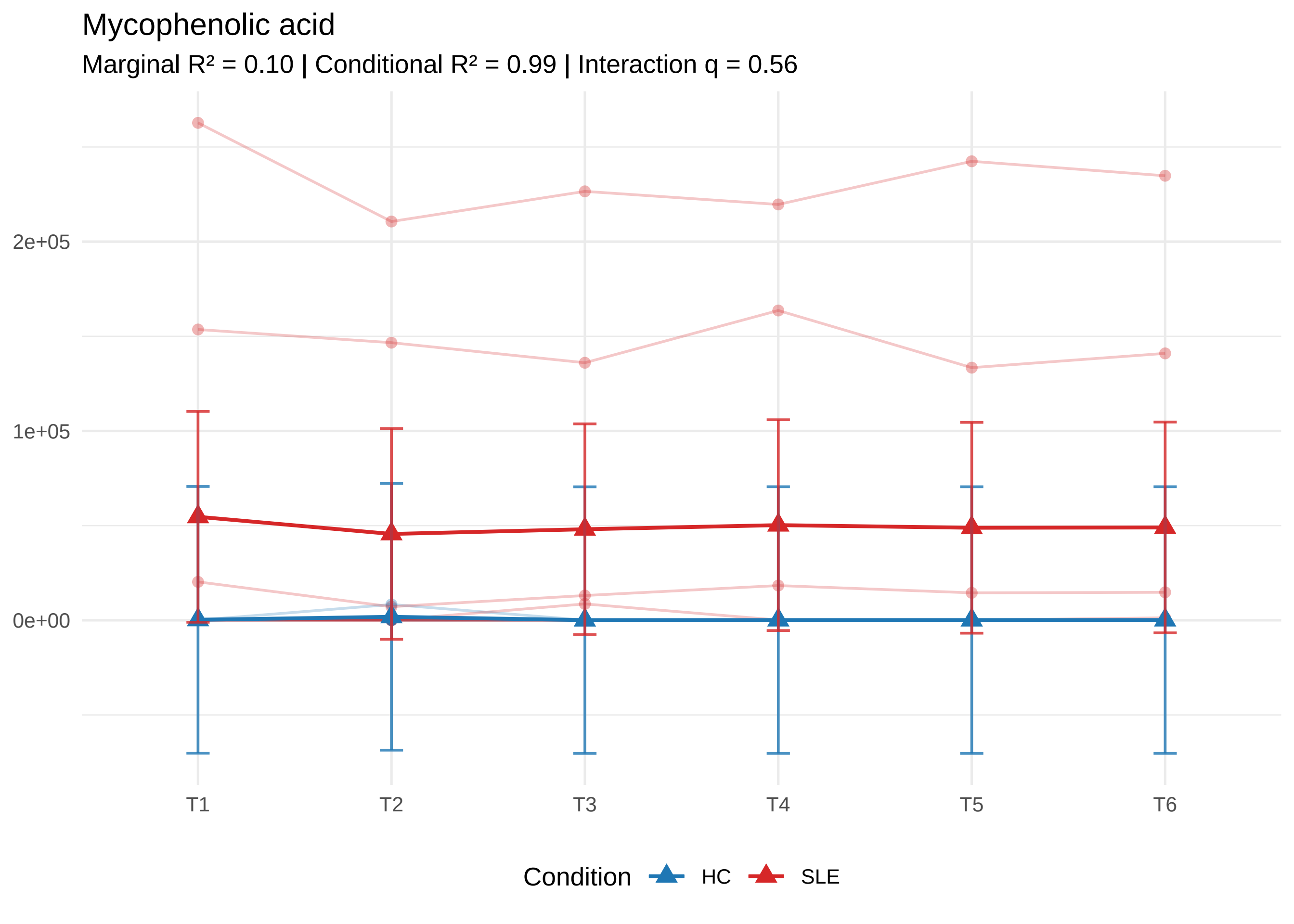
Mycophenolic acid
Marginal R² = 0.10 | Conditional R² = 0.99 | Interaction q = 0.56
2e+05
1e+05
0e+00
T1
T2
T3
T4
T5
T6
Condition
HC
SLE

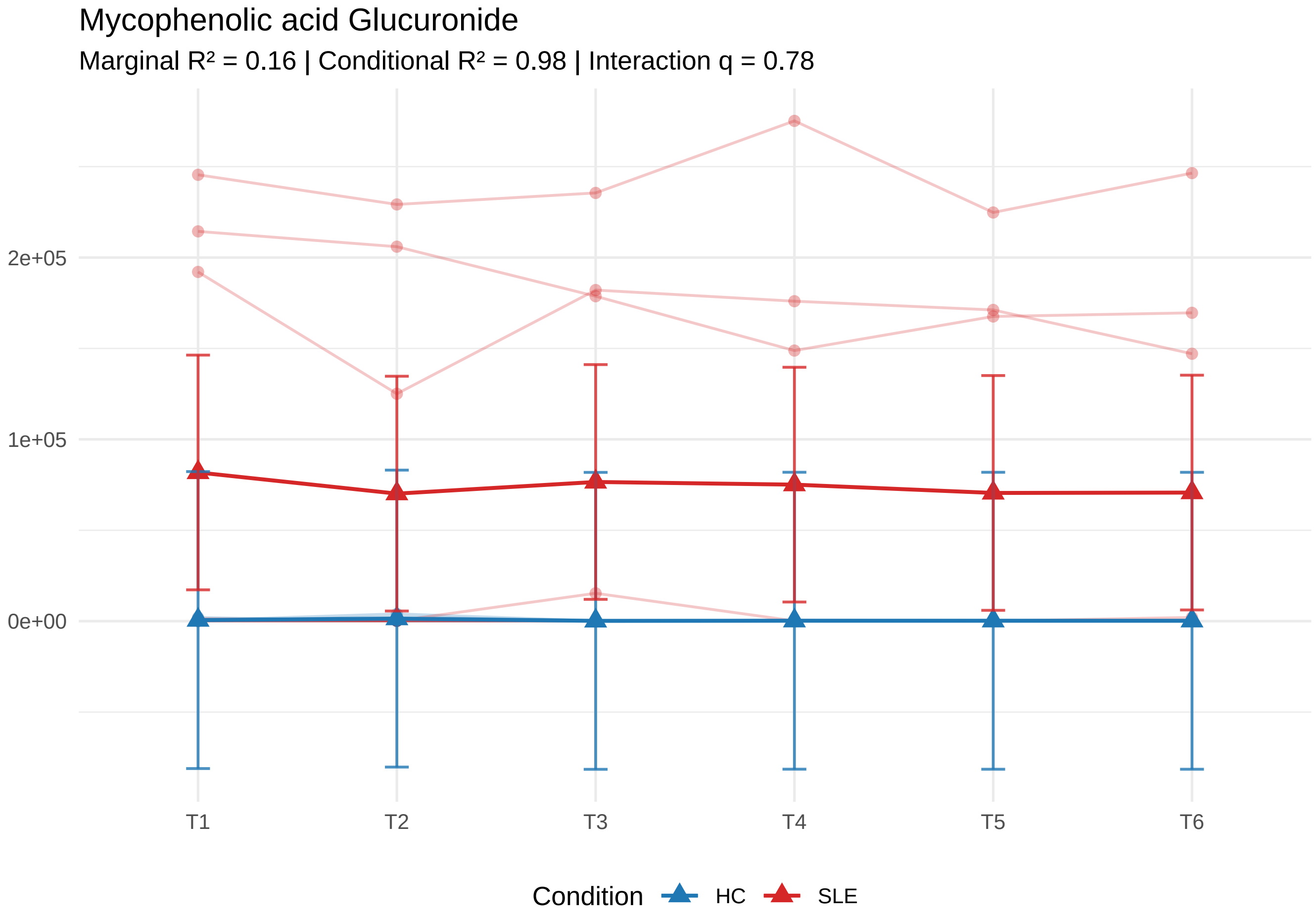
Mycophenolic acid Glucuronide
Marginal R² = 0.16 | Conditional R² = 0.98 | Interaction q = 0.78
2e+05
1e+05
0e+00
T1
T2
T3
T4
T5
T6
Condition
HC
SLE

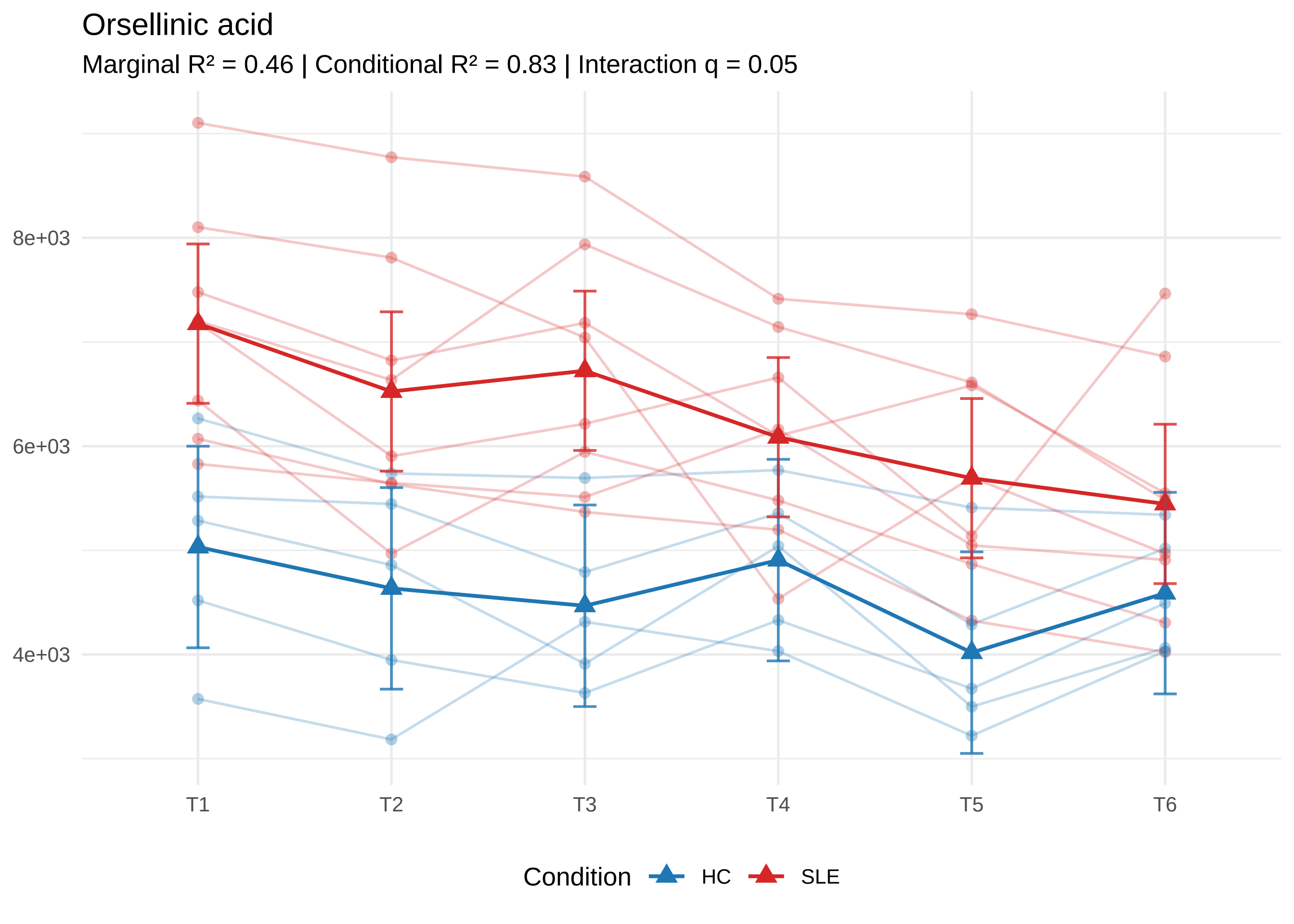

Orsellinic acid
Marginal R² = 0.46 | Conditional R² = 0.83 | Interaction q = 0.05
8e+03
6e+03
4e+03
T1
T2
T3
T4
T5
T6
Condition
HC
SLE

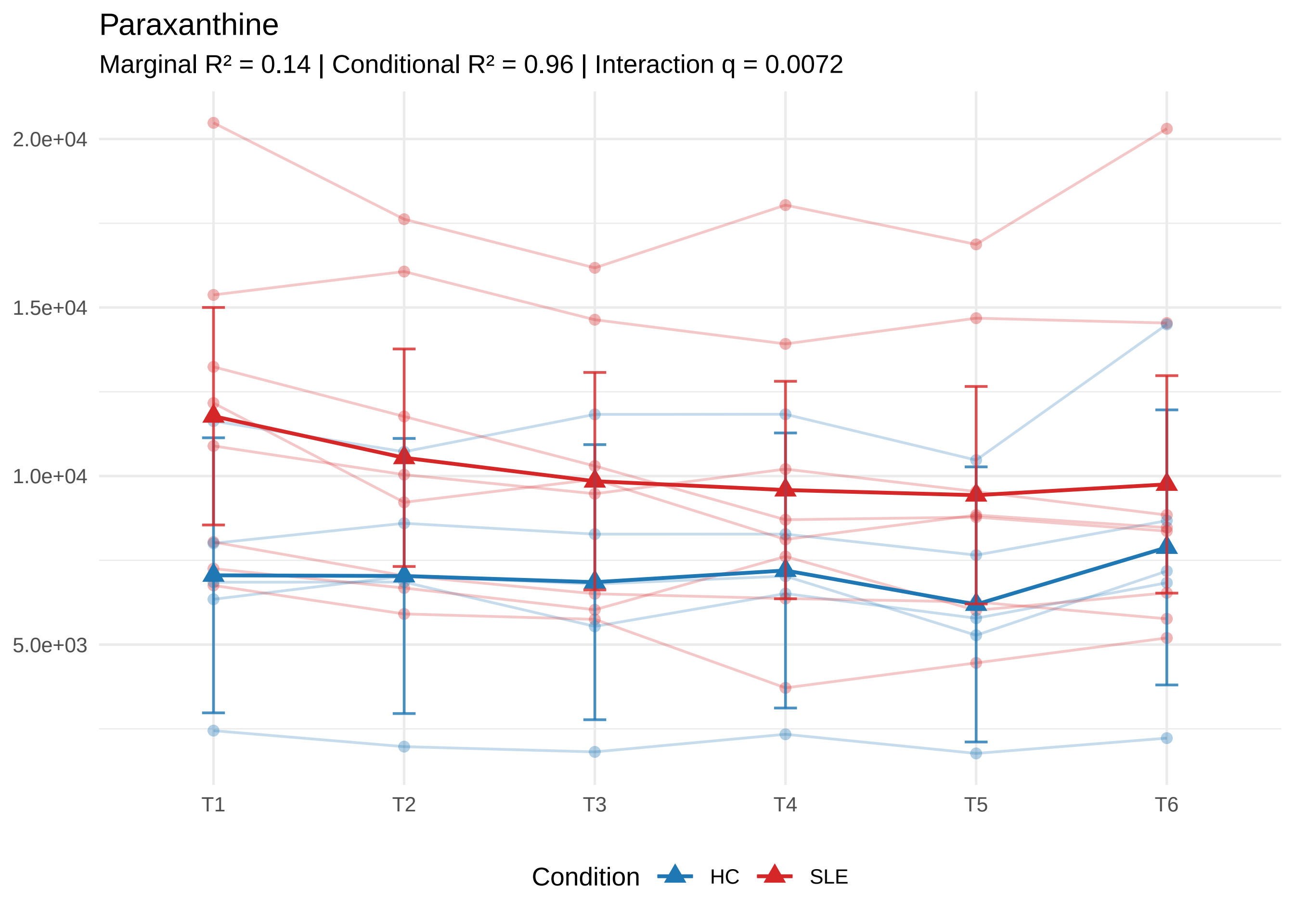

Paraxanthine
Marginal R² = 0.14 | Conditional R² = 0.96 | Interaction q = 0.0072
2.0e+04
1.5e+04
1.0e+04
5.0e+03
T1
T2
T3
T4
T5
T6
Condition
HC
SLE

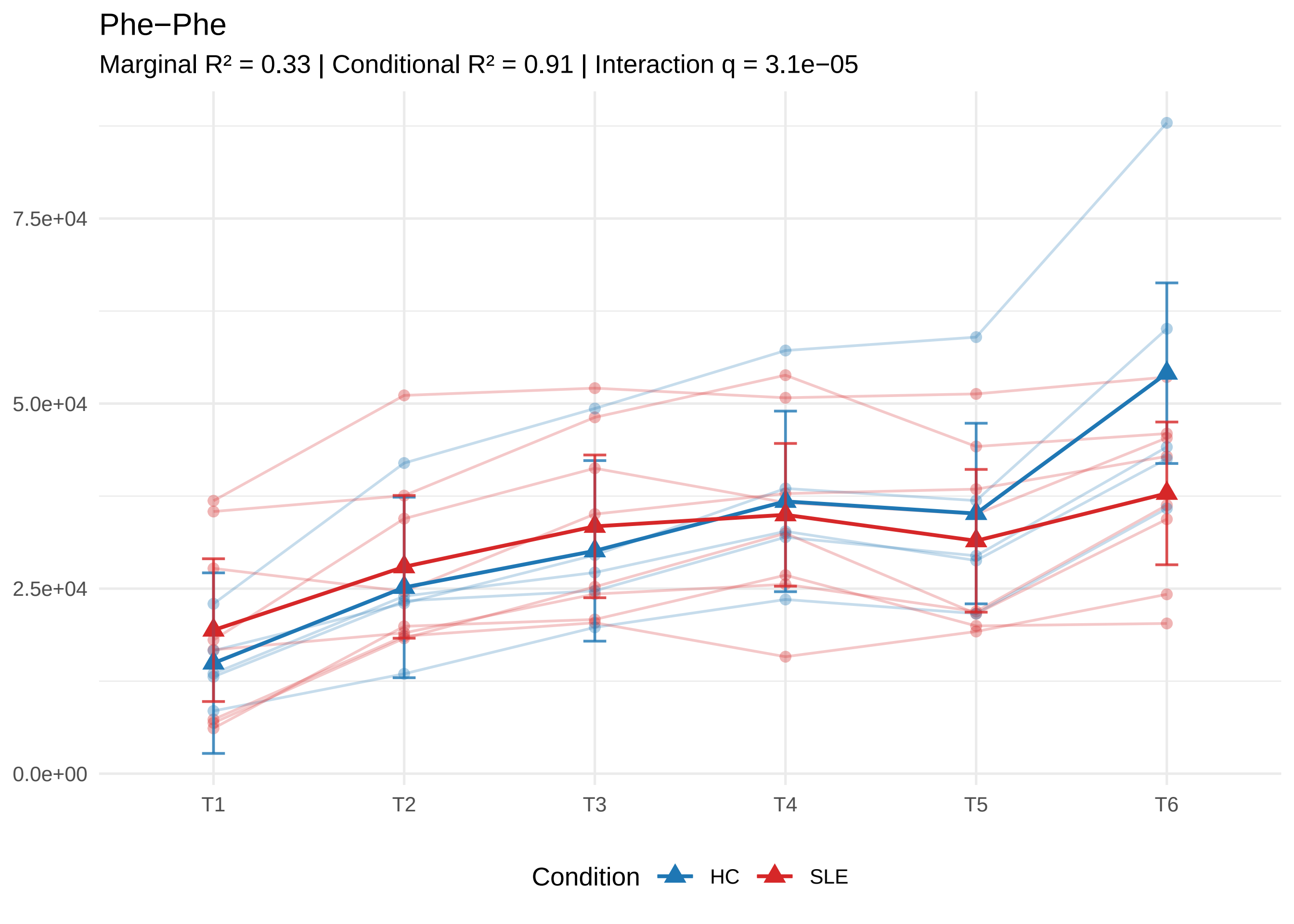

Phe−Phe
Marginal R² = 0.33 | Conditional R² = 0.91 | Interaction q = 3.1e−05
7.5e+04
5.0e+04
2.5e+04
0.0e+00
T1
T2
T3
T4
T5
T6
Condition
HC
SLE

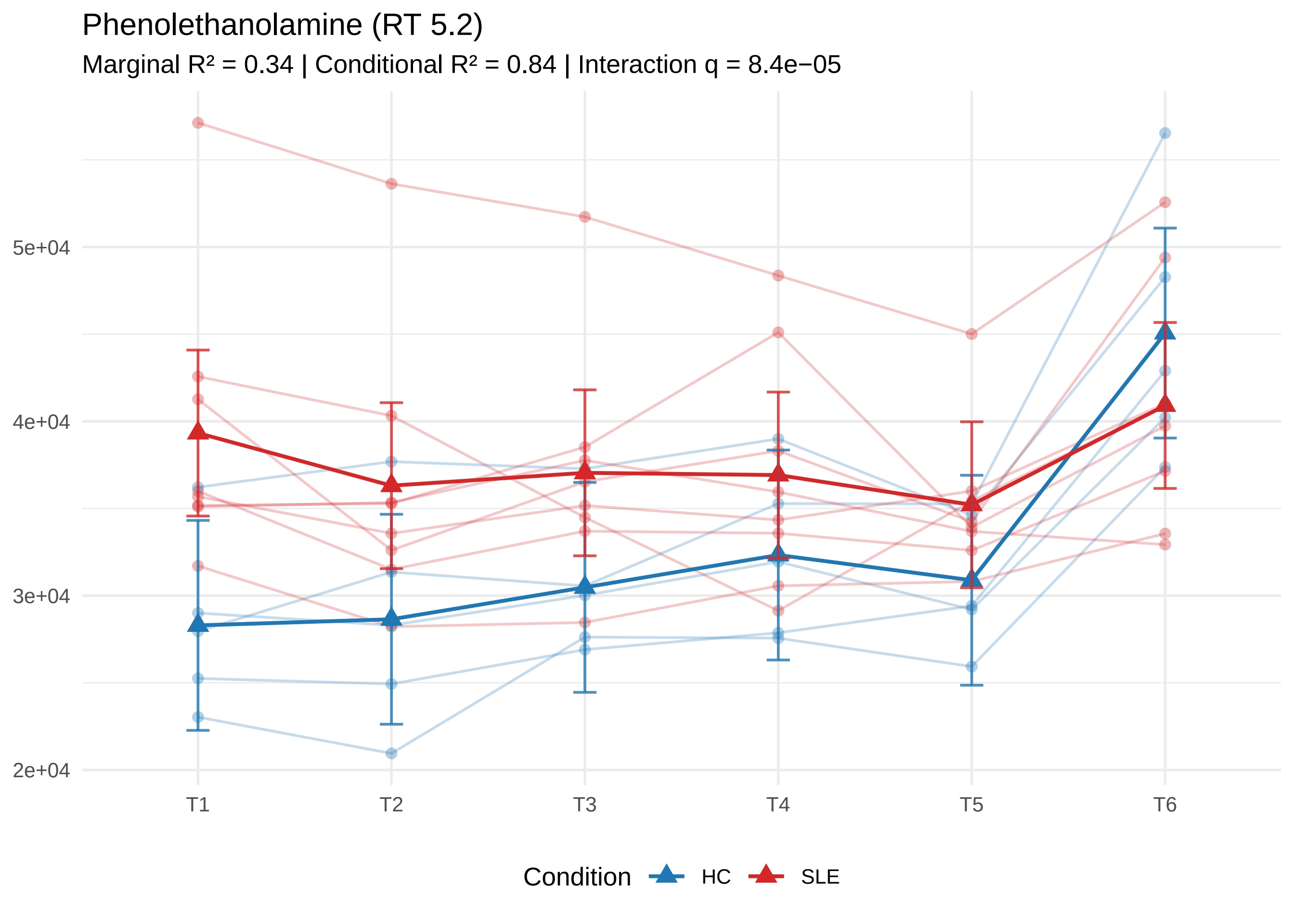
Phenolethanolamine (RT 5.2)
Marginal R² = 0.34 | Conditional R² = 0.84 | Interaction q = 8.4e−05
5e+04
4e+04
3e+04
2e+04
T1
T2
T3
T4
T5
T6
Condition
HC
SLE

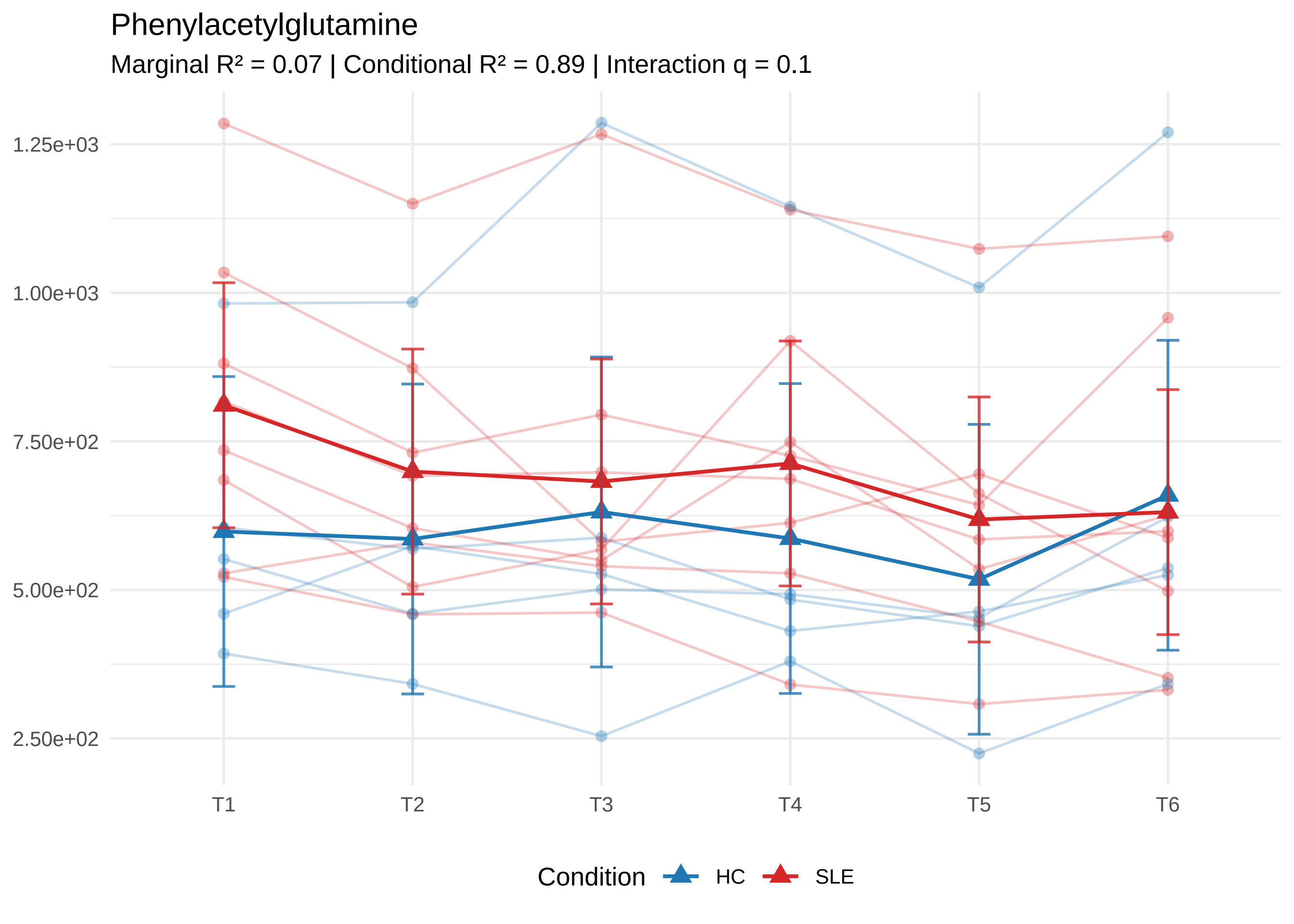
Phenylacetylglutamine
Marginal R² = 0.07 | Conditional R² = 0.89 | Interaction q = 0.1
1.25e+03
1.00e+03
7.50e+02
5.00e+02
2.50e+02
T1
T2
T3
T4
T5
T6
Condition
HC
SLE

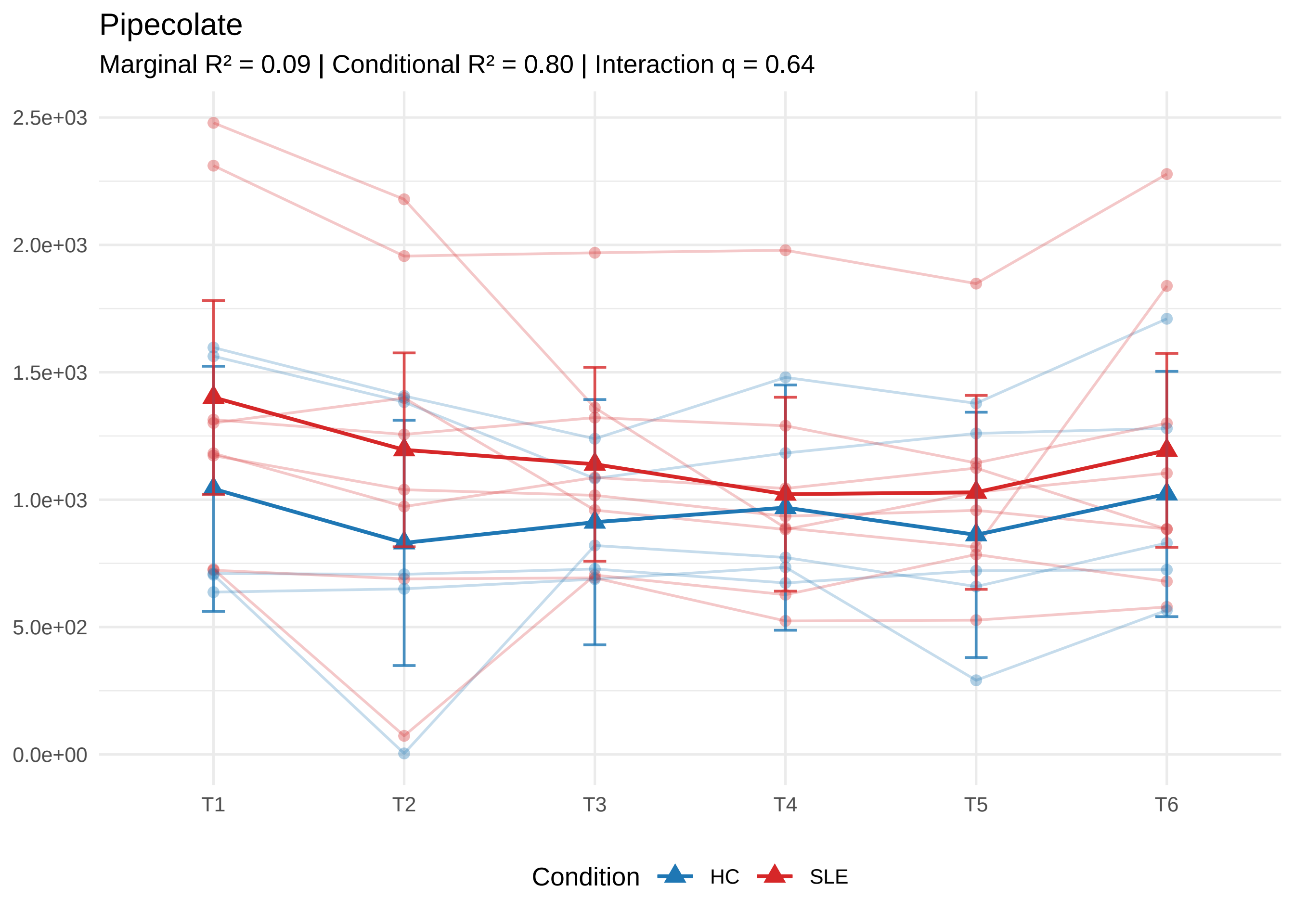

Pipecolate
Marginal R² = 0.09 | Conditional R² = 0.80 | Interaction q = 0.64
2.5e+03
2.0e+03
1.5e+03
1.0e+03
5.0e+02
0.0e+00
T1
T2
T3
T4
T5
T6
Condition
HC
SLE

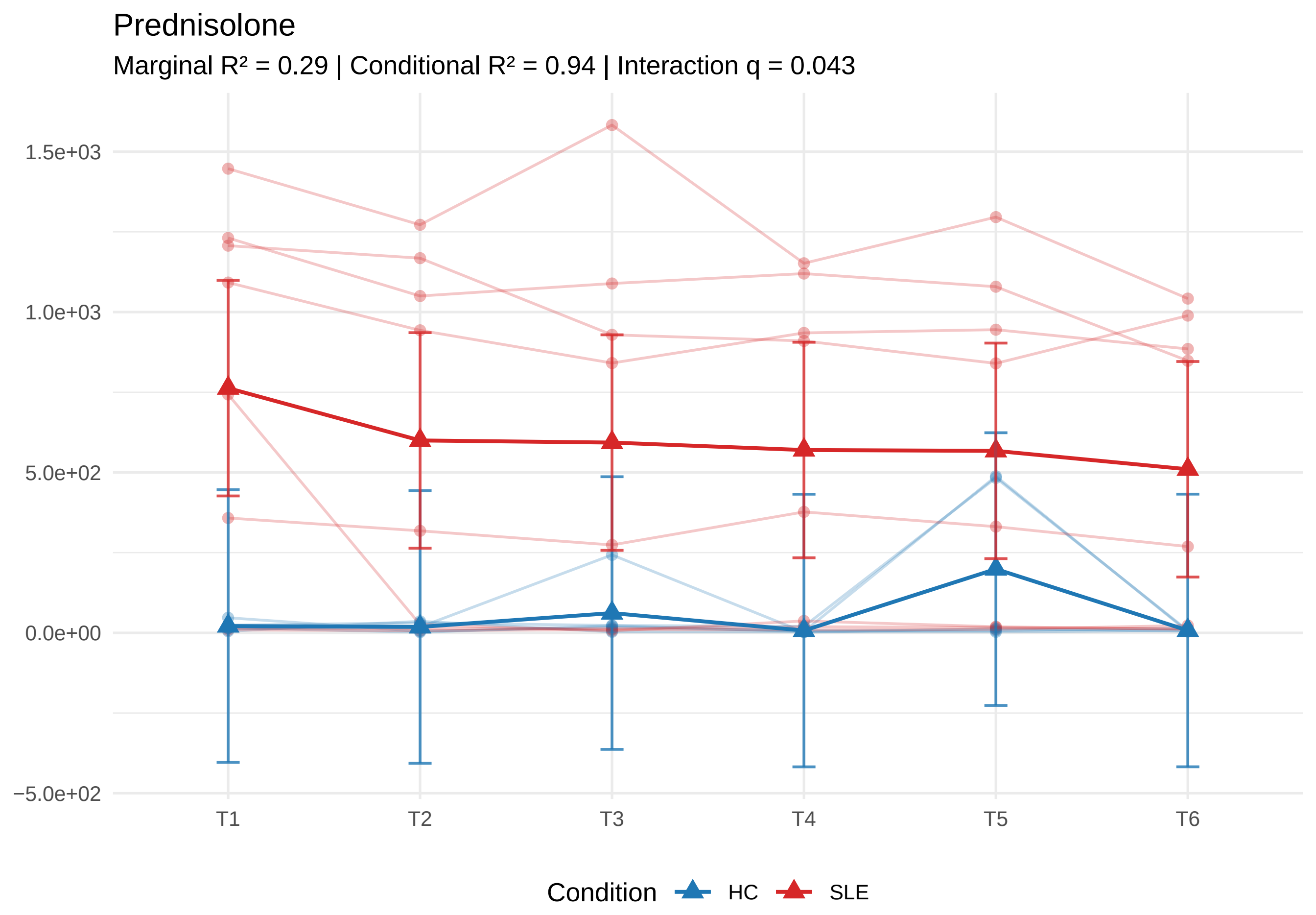

Prednisolone
Marginal R² = 0.29 | Conditional R² = 0.94 | Interaction q = 0.043
1.5e+03
1.0e+03
5.0e+02
0.0e+00
−5.0e+02
T1
T2
T3
T4
T5
T6
Condition
HC
SLE

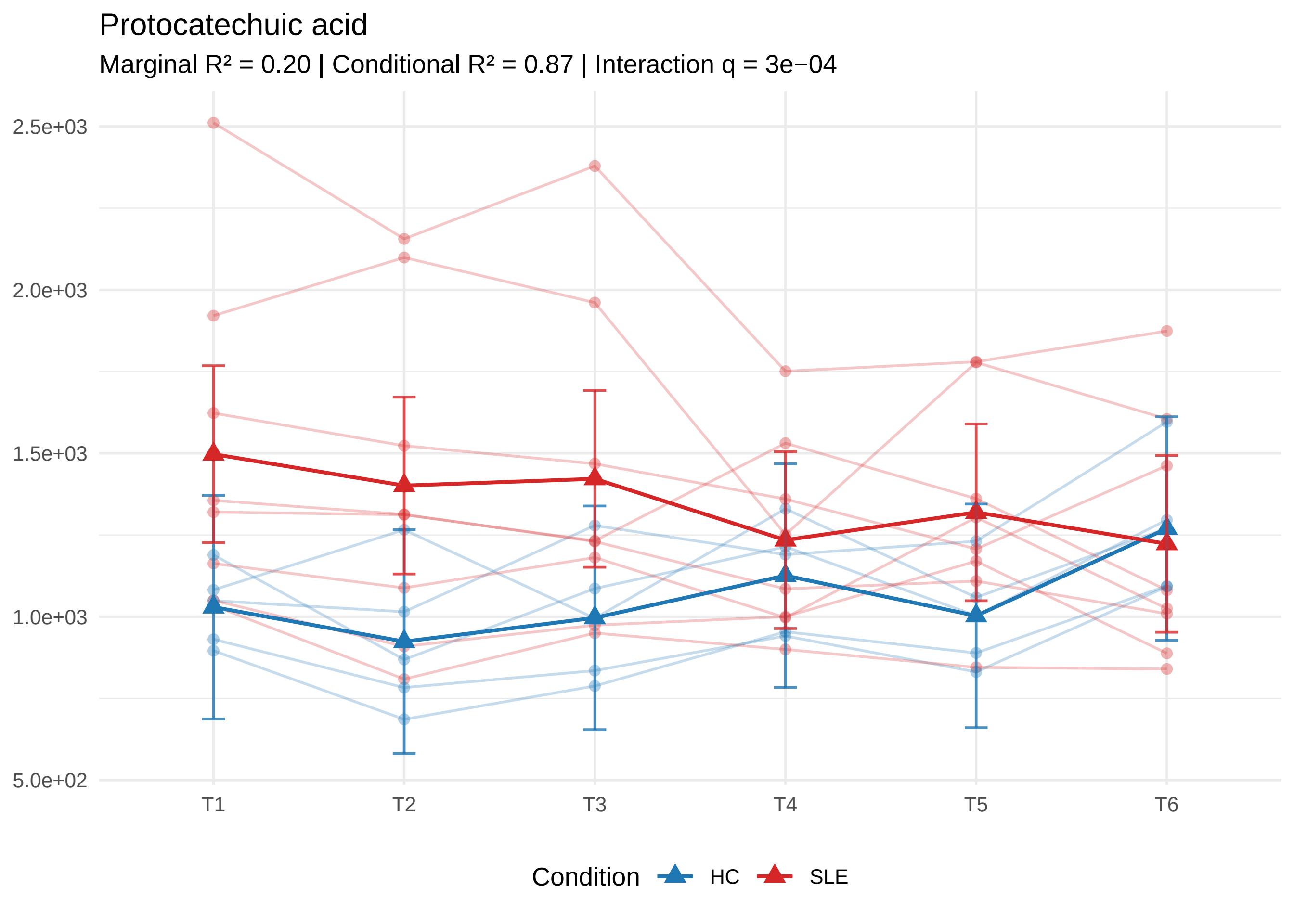

Protocatechuic acid
Marginal R² = 0.20 | Conditional R² = 0.87 | Interaction q = 3e−04
2.5e+03
2.0e+03
1.5e+03
1.0e+03
5.0e+02
T1
T2
T3
T4
T5
T6
Condition
HC
SLE

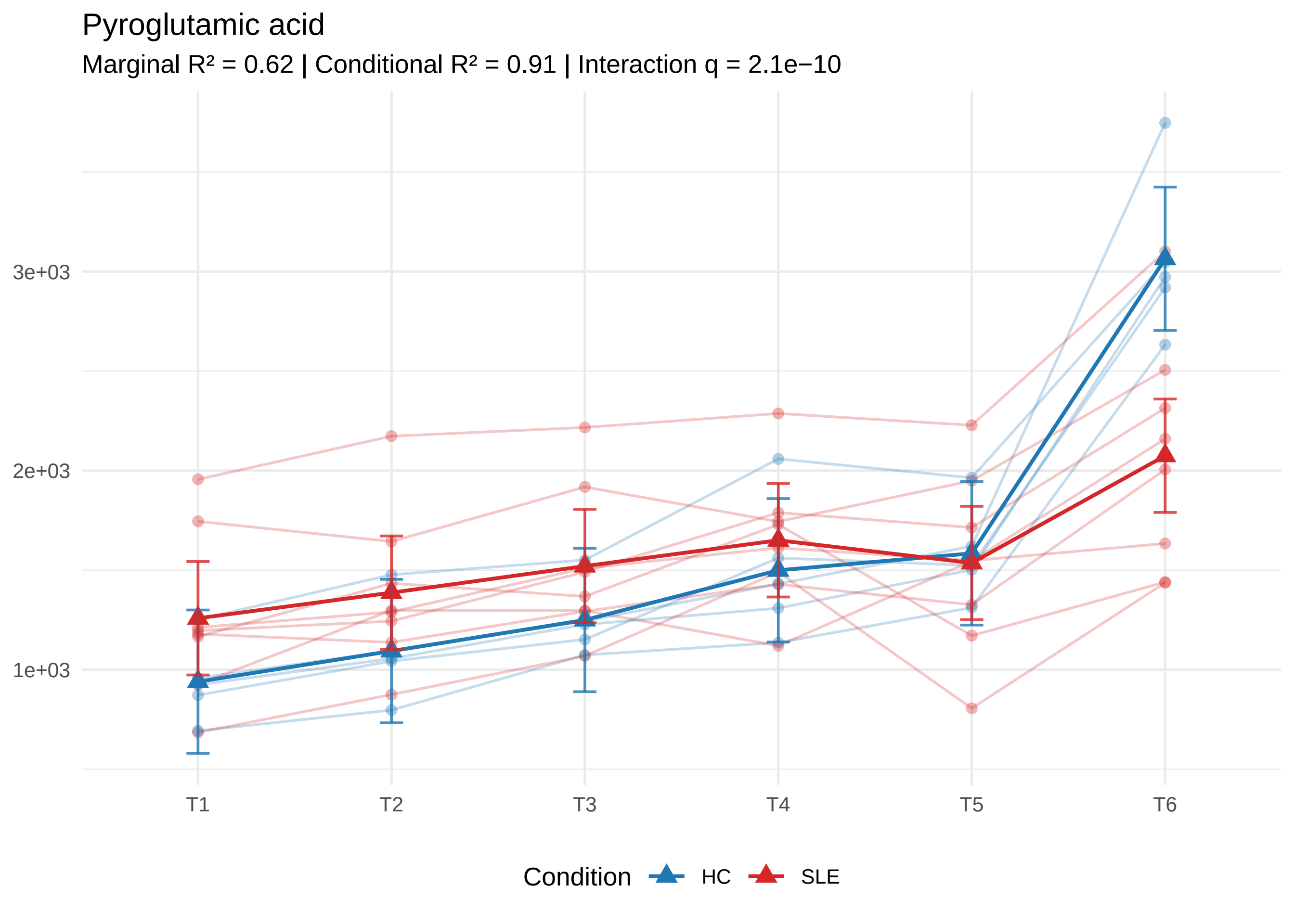
Pyroglutamic acid
Marginal R² = 0.62 | Conditional R² = 0.91 | Interaction q = 2.1e−10
3e+03
2e+03
1e+03
T1
T2
T3
T4
T5
T6
Condition
HC
SLE

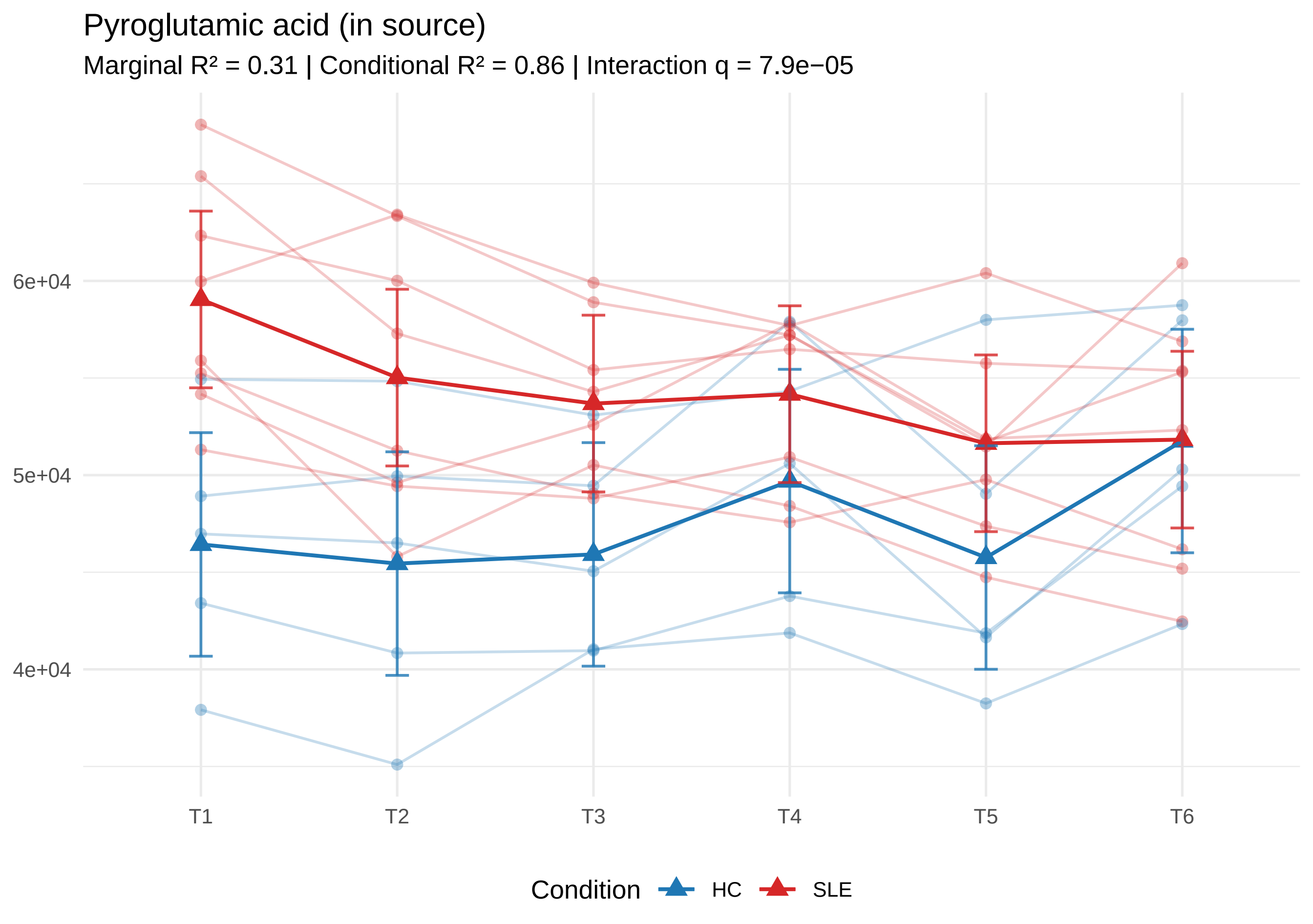
Pyroglutamic acid (in source)
Marginal R² = 0.31 | Conditional R² = 0.86 | Interaction q = 7.9e−05
6e+04
5e+04
4e+04
T1
T2
T3
T4
T5
T6
Condition
HC
SLE

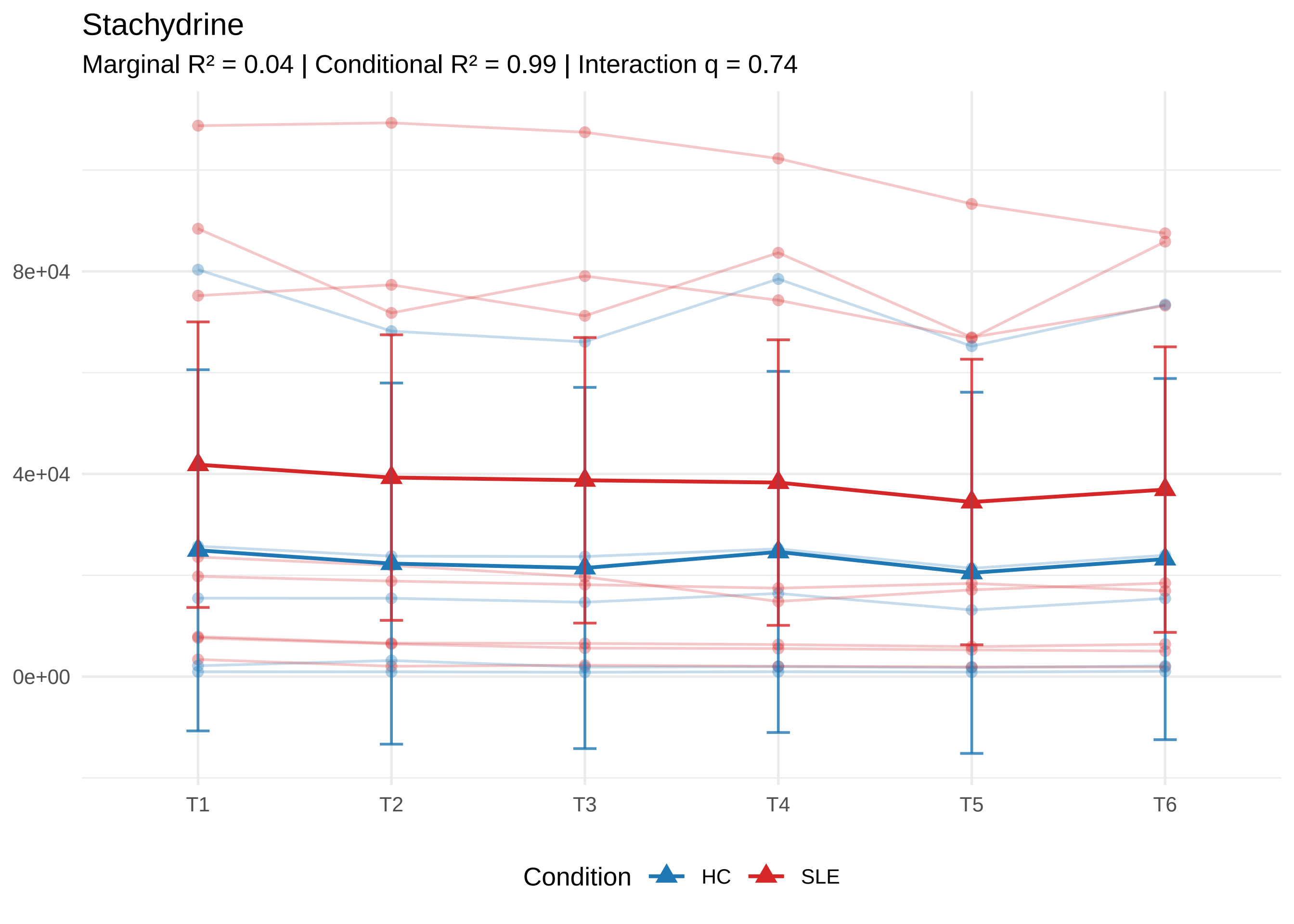

Stachydrine
Marginal R² = 0.04 | Conditional R² = 0.99 | Interaction q = 0.74
8e+04
4e+04
0e+00
T1
T2
T3
T4
T5
T6
Condition
HC
SLE

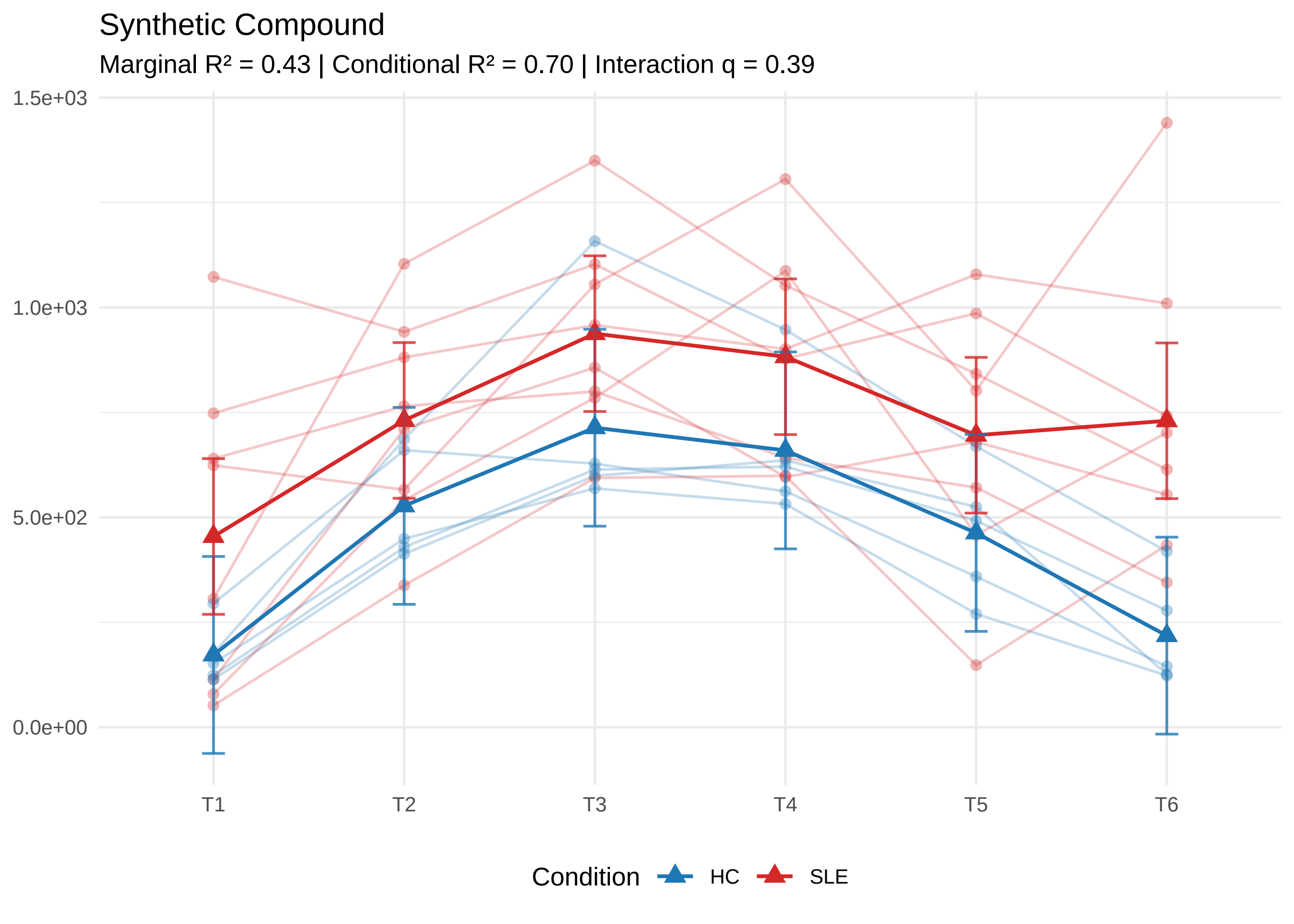
Synthetic Compound
Marginal R² = 0.43 | Conditional R² = 0.70 | Interaction q = 0.39
1.5e+03
1.0e+03
5.0e+02
0.0e+00
T1
T2
T3
T4
T5
T6
Condition
HC
SLE

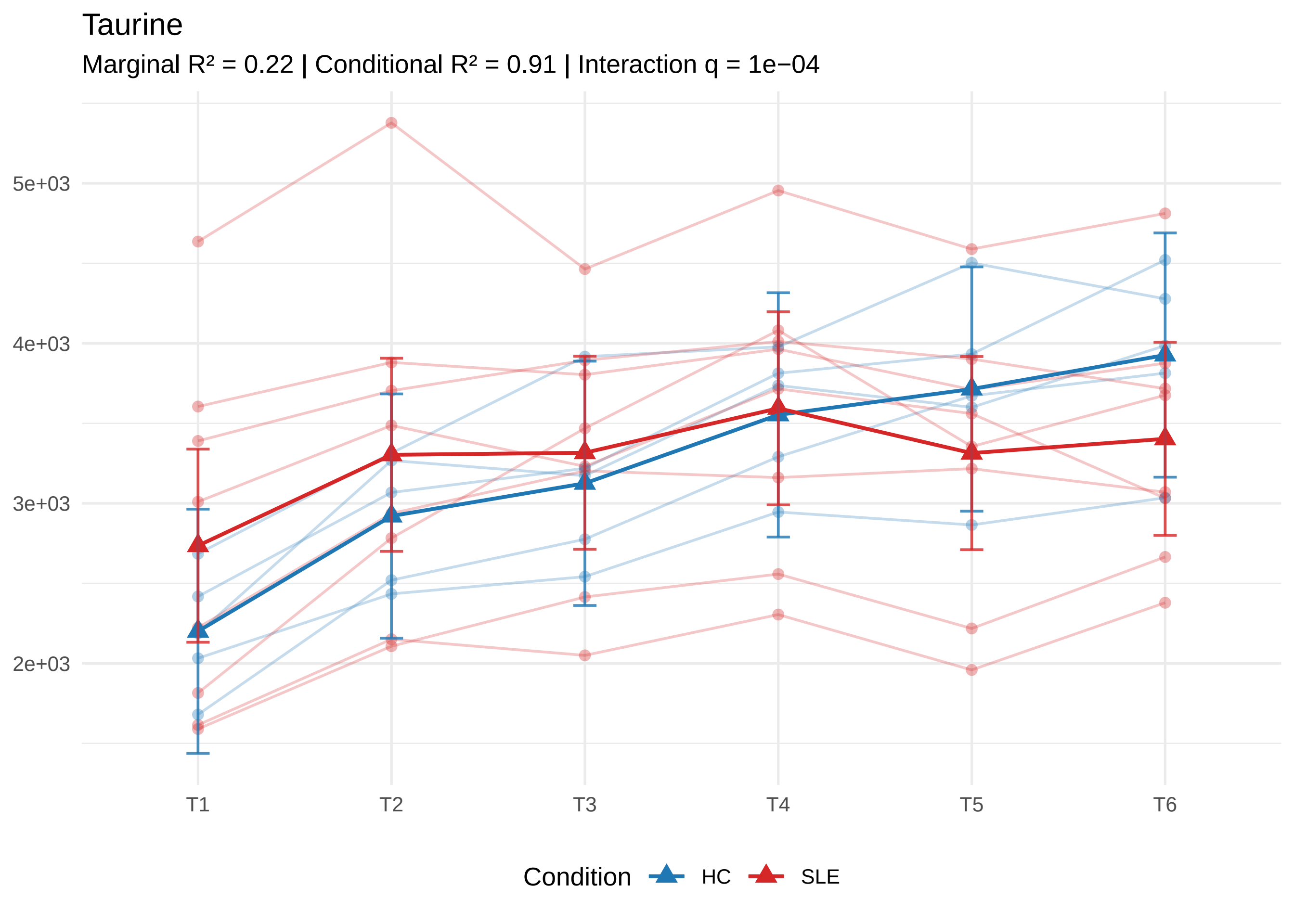
Taurine
Marginal R² = 0.22 | Conditional R² = 0.91 | Interaction q = 1e−04
5e+03
4e+03
3e+03
2e+03
T1
T2
T3
T4
T5
T6
Condition
HC
SLE

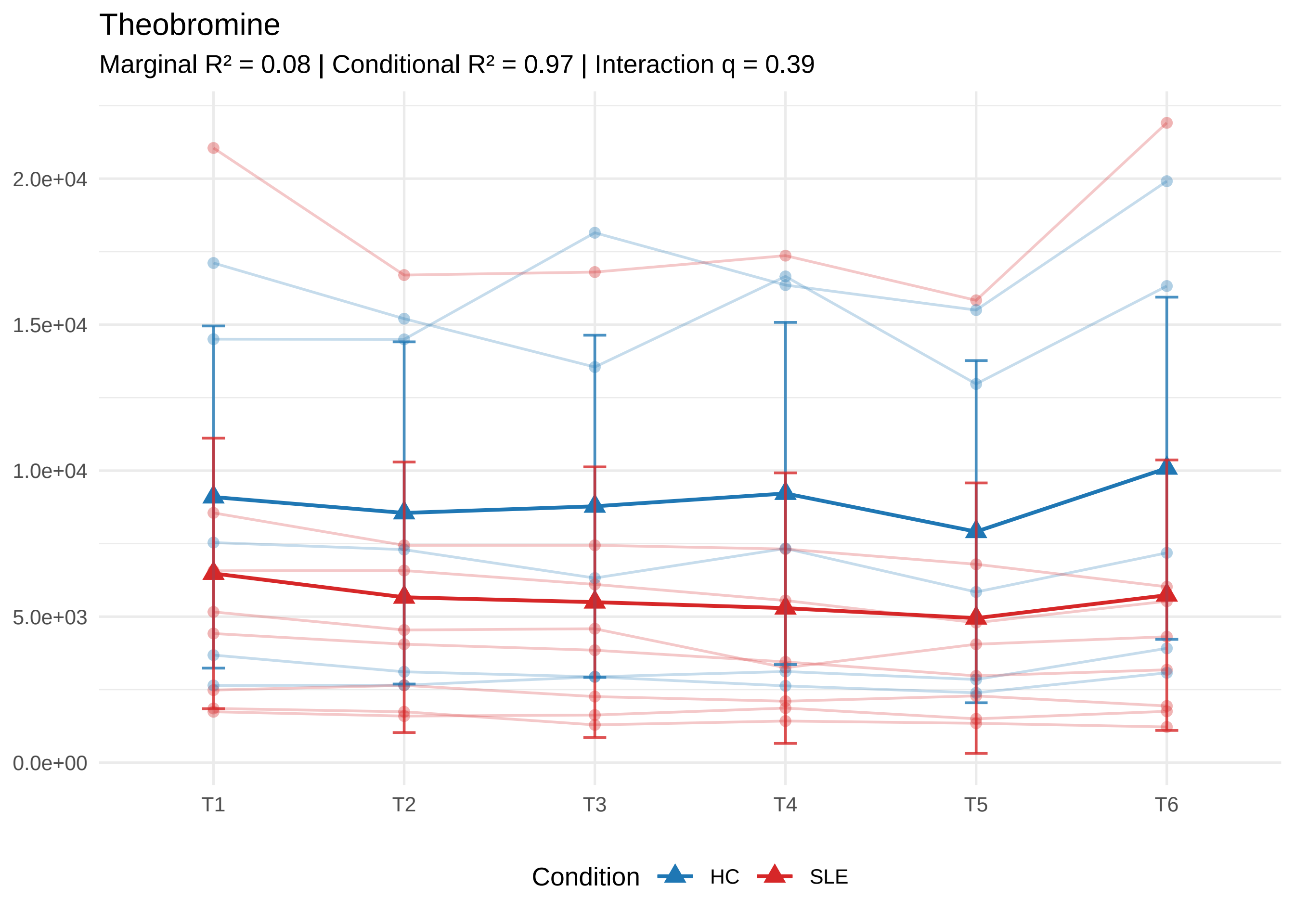
Theobromine
Marginal R² = 0.08 | Conditional R² = 0.97 | Interaction q = 0.39
2.0e+04
1.5e+04
1.0e+04
5.0e+03
0.0e+00
T1
T2
T3
T4
T5
T6
Condition
HC
SLE

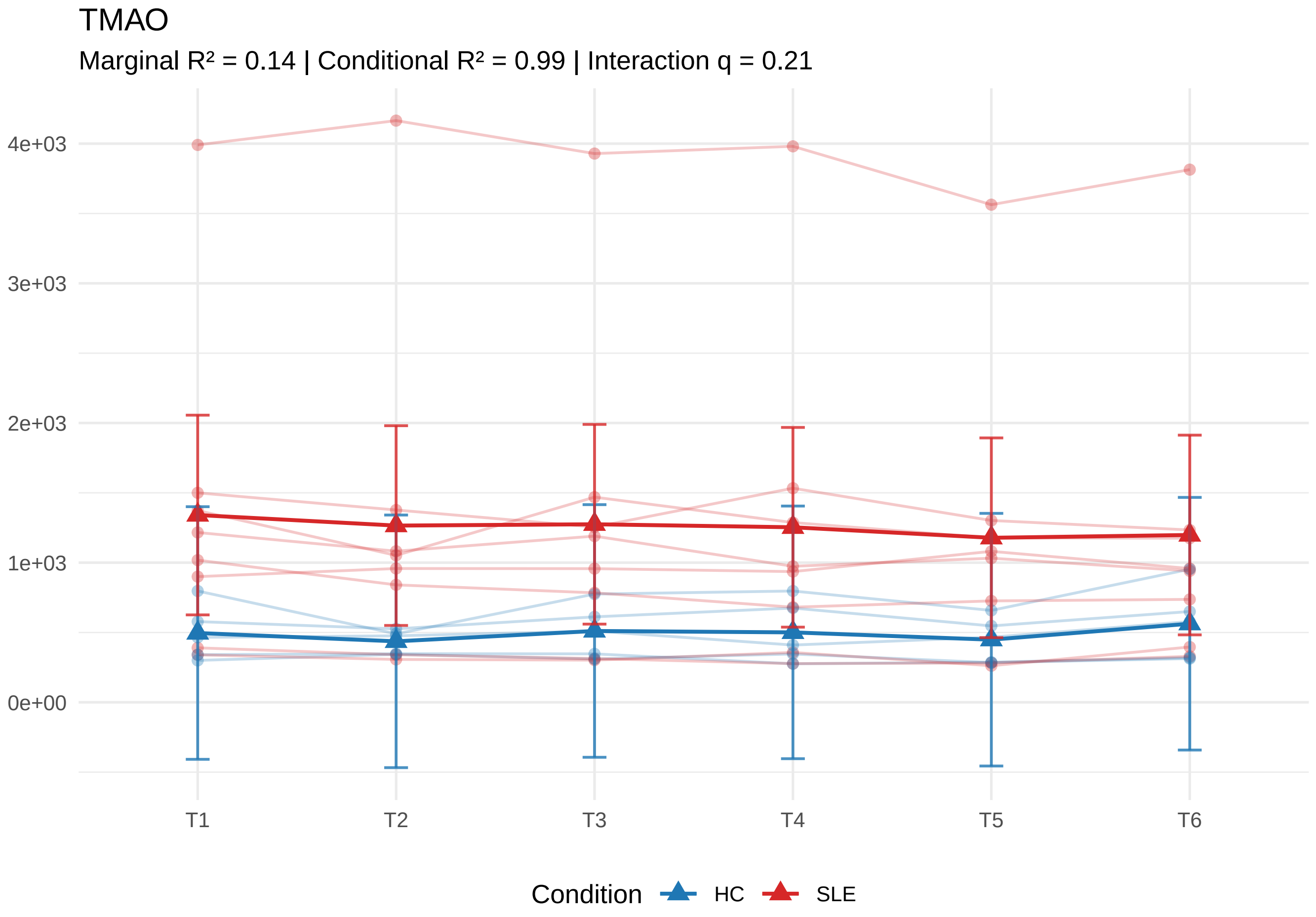
TMAO
Marginal R² = 0.14 | Conditional R² = 0.99 | Interaction q = 0.21
4e+03
3e+03
2e+03
1e+03
0e+00
T1
T2
T3
T4
T5
T6
Condition
HC
SLE

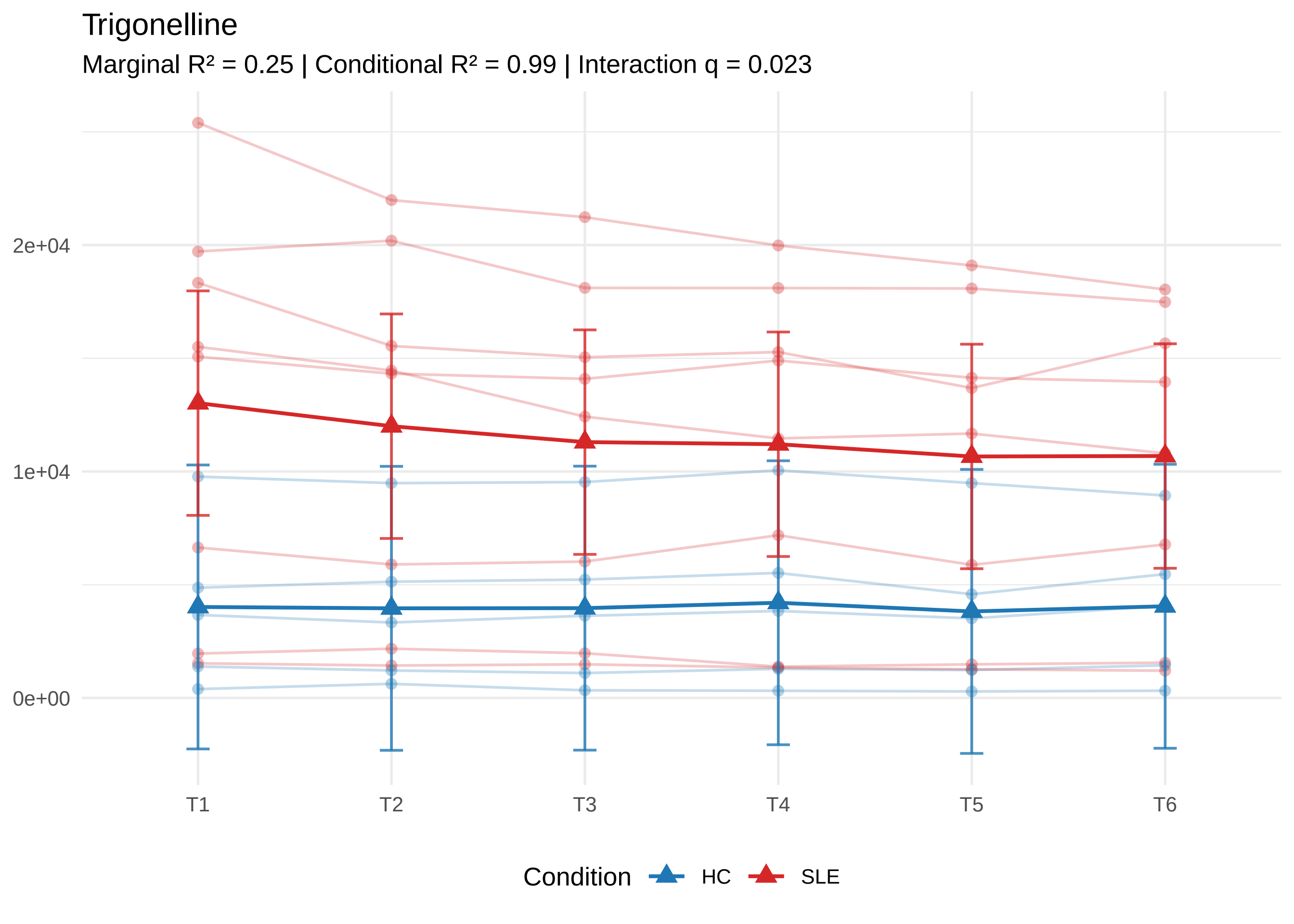

Trigonelline
Marginal R² = 0.25 | Conditional R² = 0.99 | Interaction q = 0.023
2e+04
1e+04
0e+00
T1
T2
T3
T4
T5
T6
Condition
HC
SLE

UDCA

Marginal R^2 = 0.04 | Conditional R^2 = 0.96 | Interaction q = 0.94

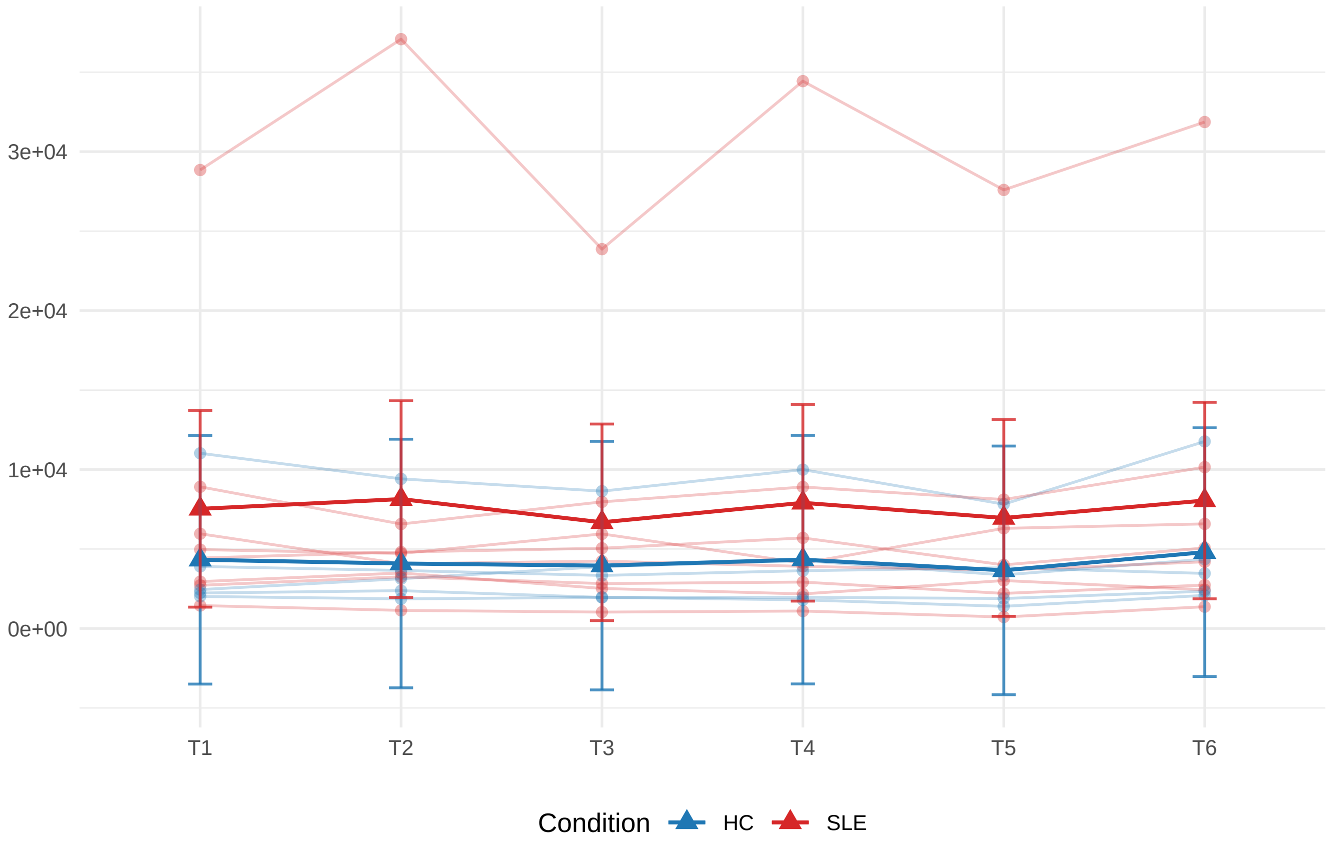

Uric acid

Marginal R^2 = 0.09 | Conditional R^2 = 0.89 | Interaction q = 0.011

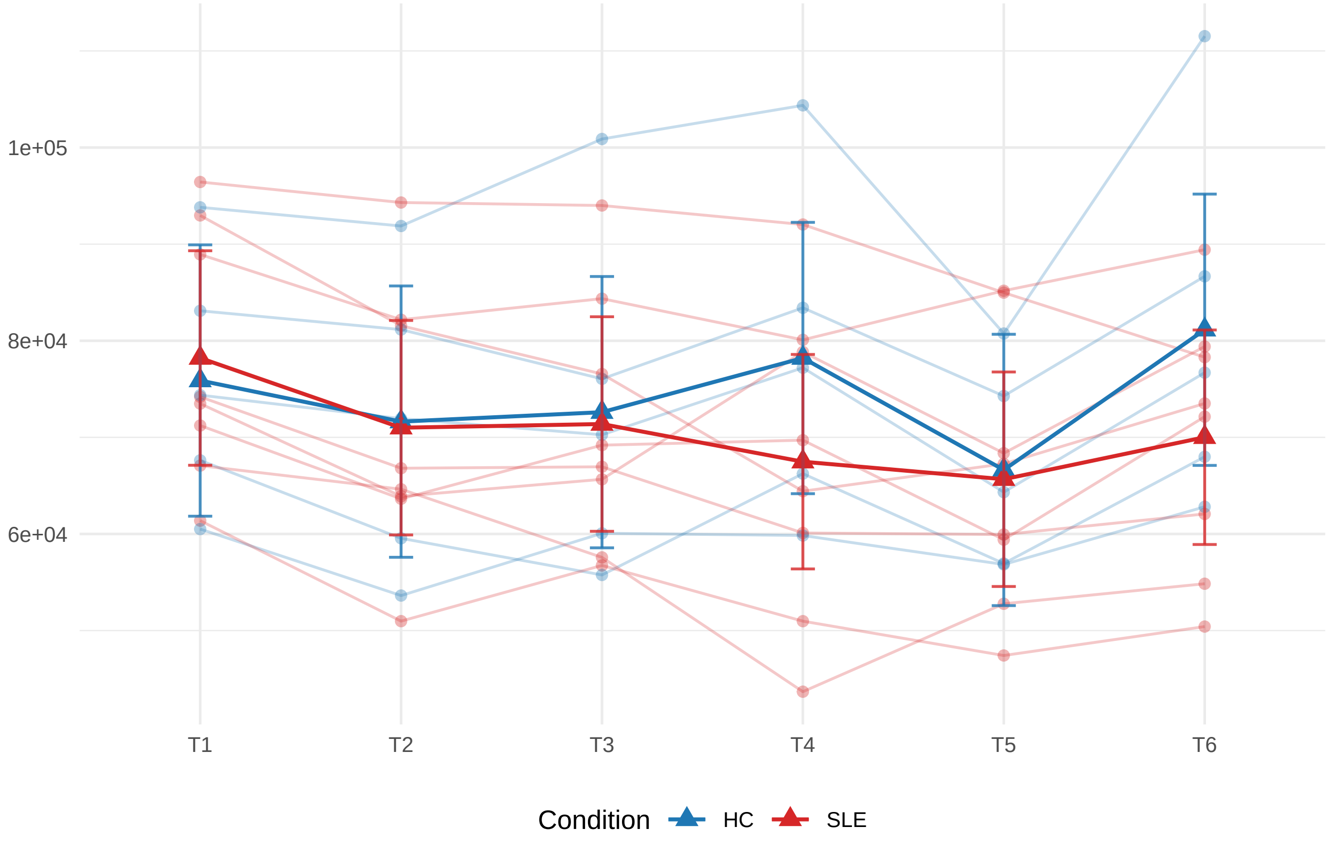

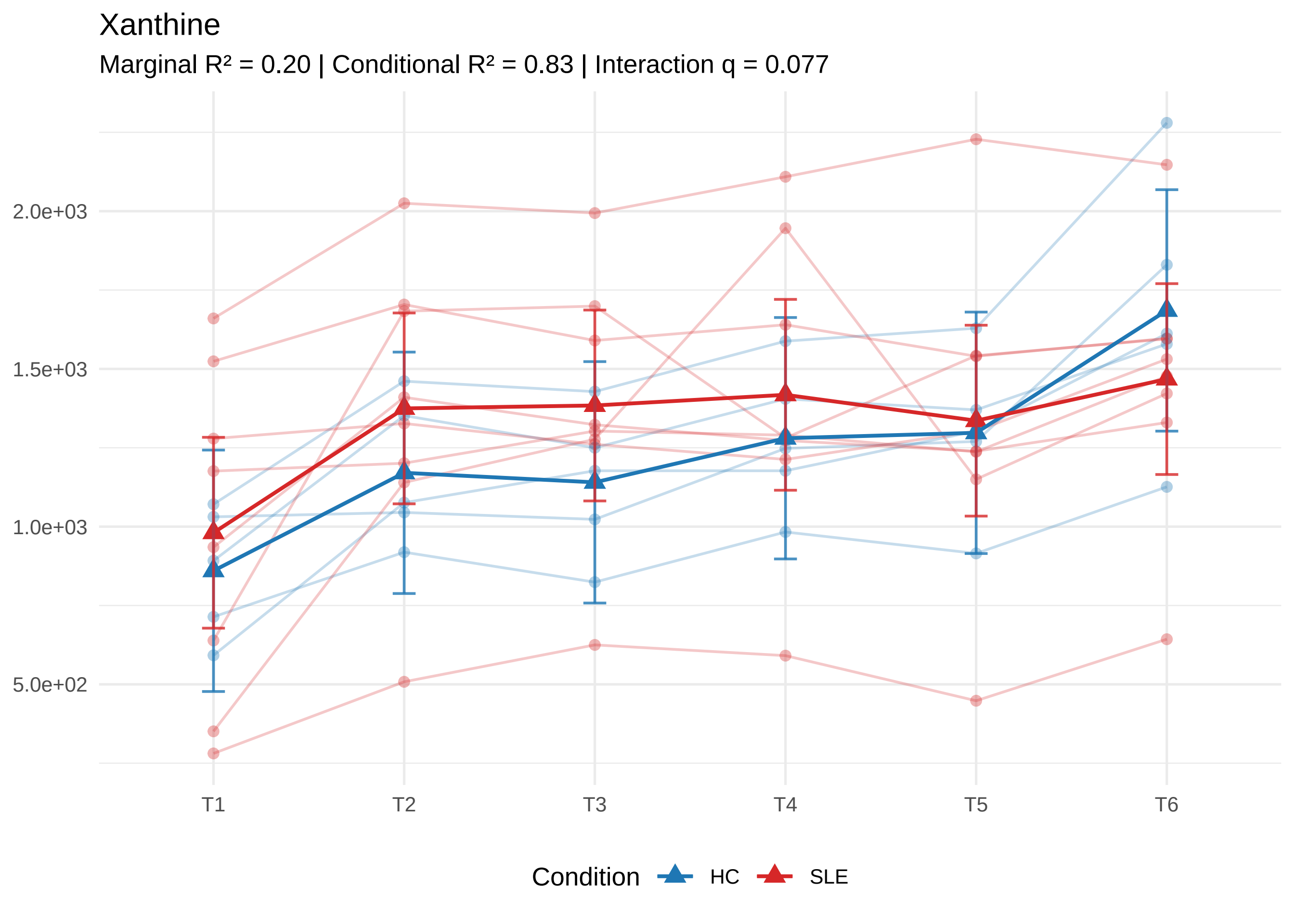
Xanthine
Marginal R² = 0.20 | Conditional R² = 0.83 | Interaction q = 0.077
2.0e+03
1.5e+03
1.0e+03
5.0e+02
T1
T2
T3
T4
T5
T6
Condition
HC
SLE
