# Supplementary material for: Disease and Medication Context Shape Ex Vivo Metabolite Stability: A Pilot Study in Systemic Lupus Erythematosus
Source: Metabolites. 2025 Nov 12;15(11):738. doi: 10.3390/metabo15110738 (PMC12654355; doi:10.3390/metabo15110738)
Supplement: Supplementary file 1 [file metabolites-15-00738-s001.zip › Supplementary Figure S7.pdf]

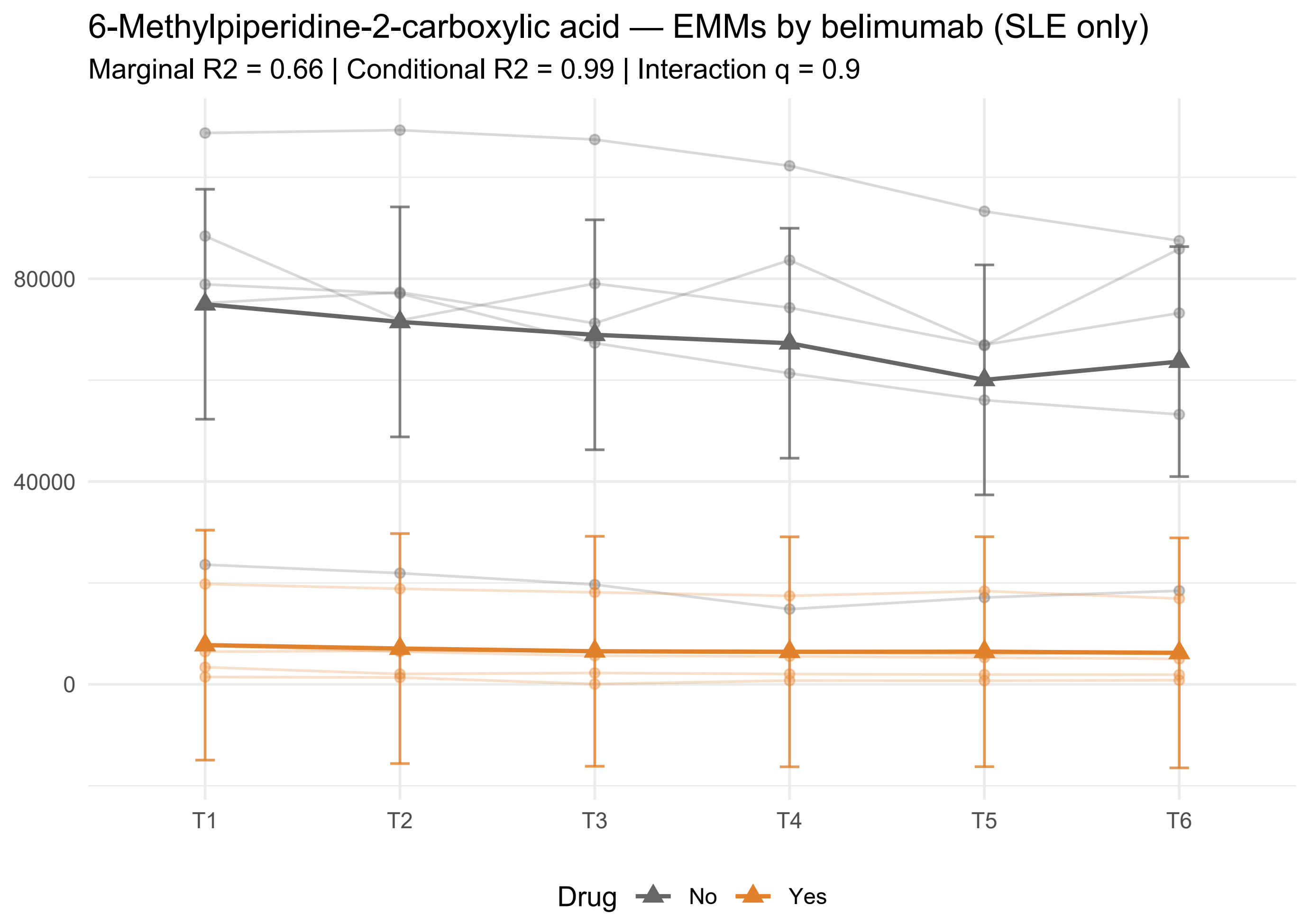

6-Methylpiperidine-2-carboxylic acid — EMMs by belimumab (SLE only)
Marginal R2 = 0.66 | Conditional R2 = 0.99 | Interaction q = 0.9
80000
40000
0
T1
T2
T3
T4
T5
T6
Drug
No
Yes

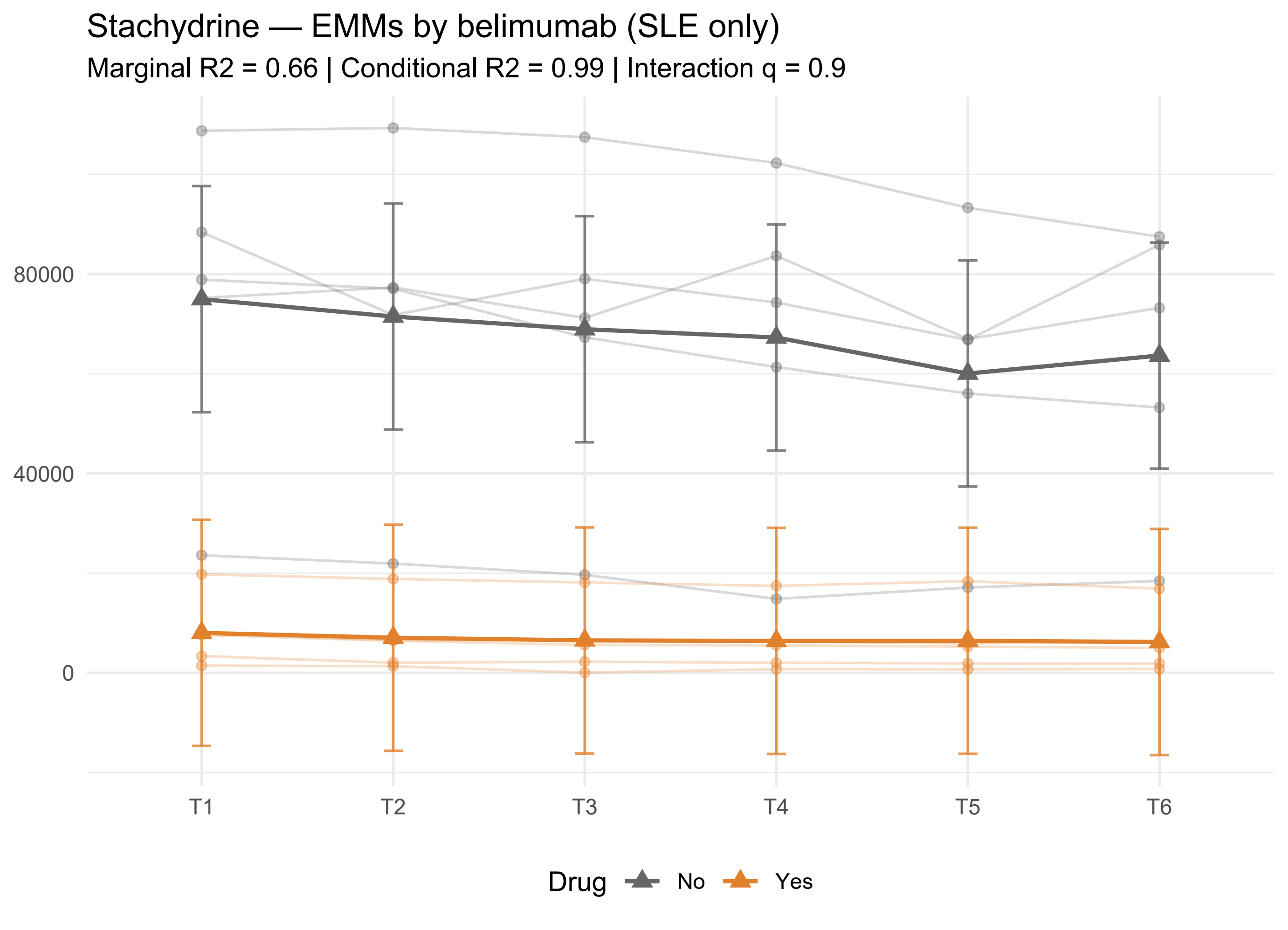

Stachydrine — EMMs by belimumab (SLE only)
Marginal R2 = 0.66 | Conditional R2 = 0.99 | Interaction q = 0.9
80000
40000
0
T1
T2
T3
T4
T5
T6
Drug
No
Yes

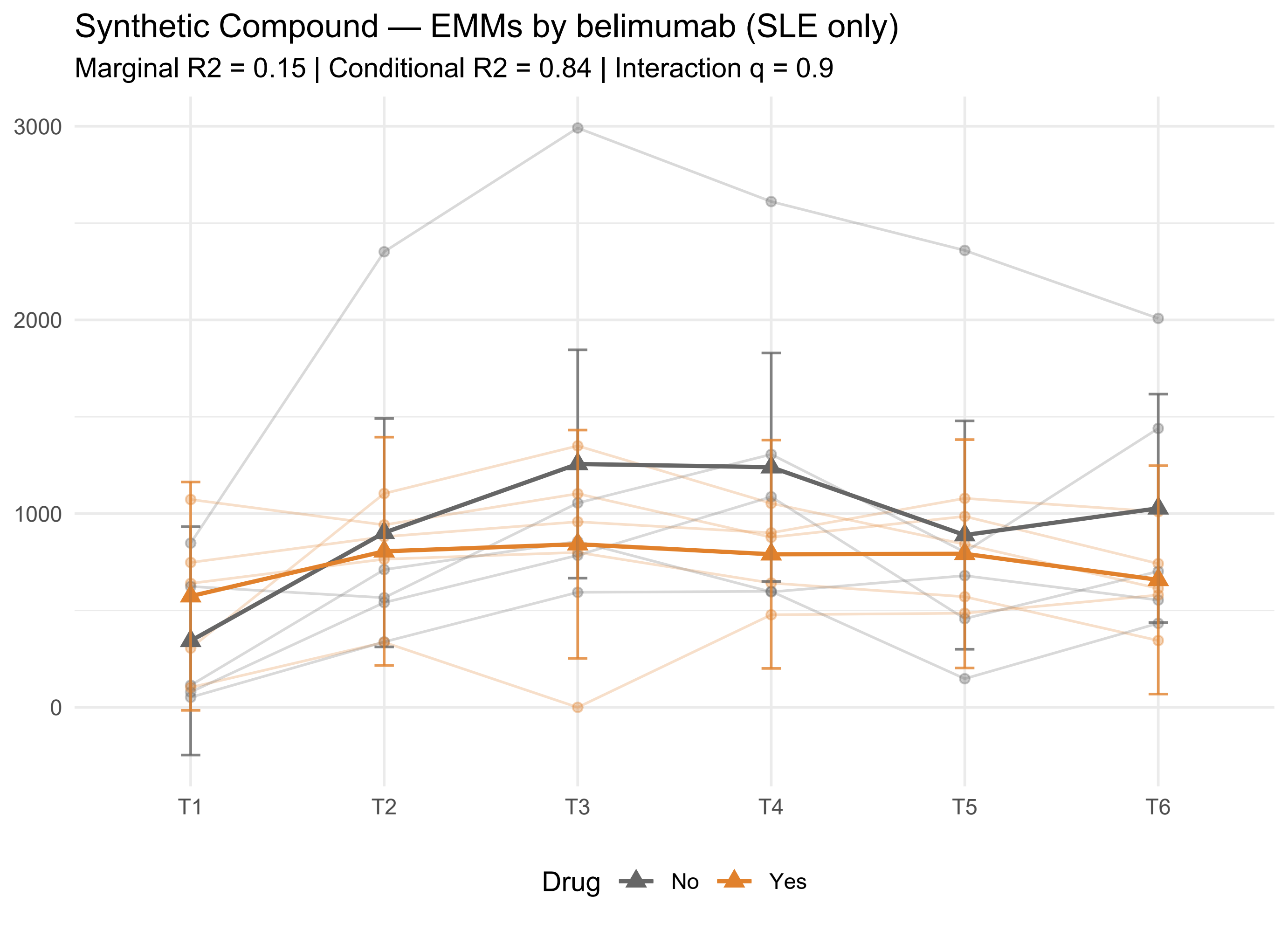
Synthetic Compound — EMMs by belimumab (SLE only)
Marginal R2 = 0.15 | Conditional R2 = 0.84 | Interaction q = 0.9
3000
2000
1000
0
T1
T2
T3
T4
T5
T6
Drug
No
Yes

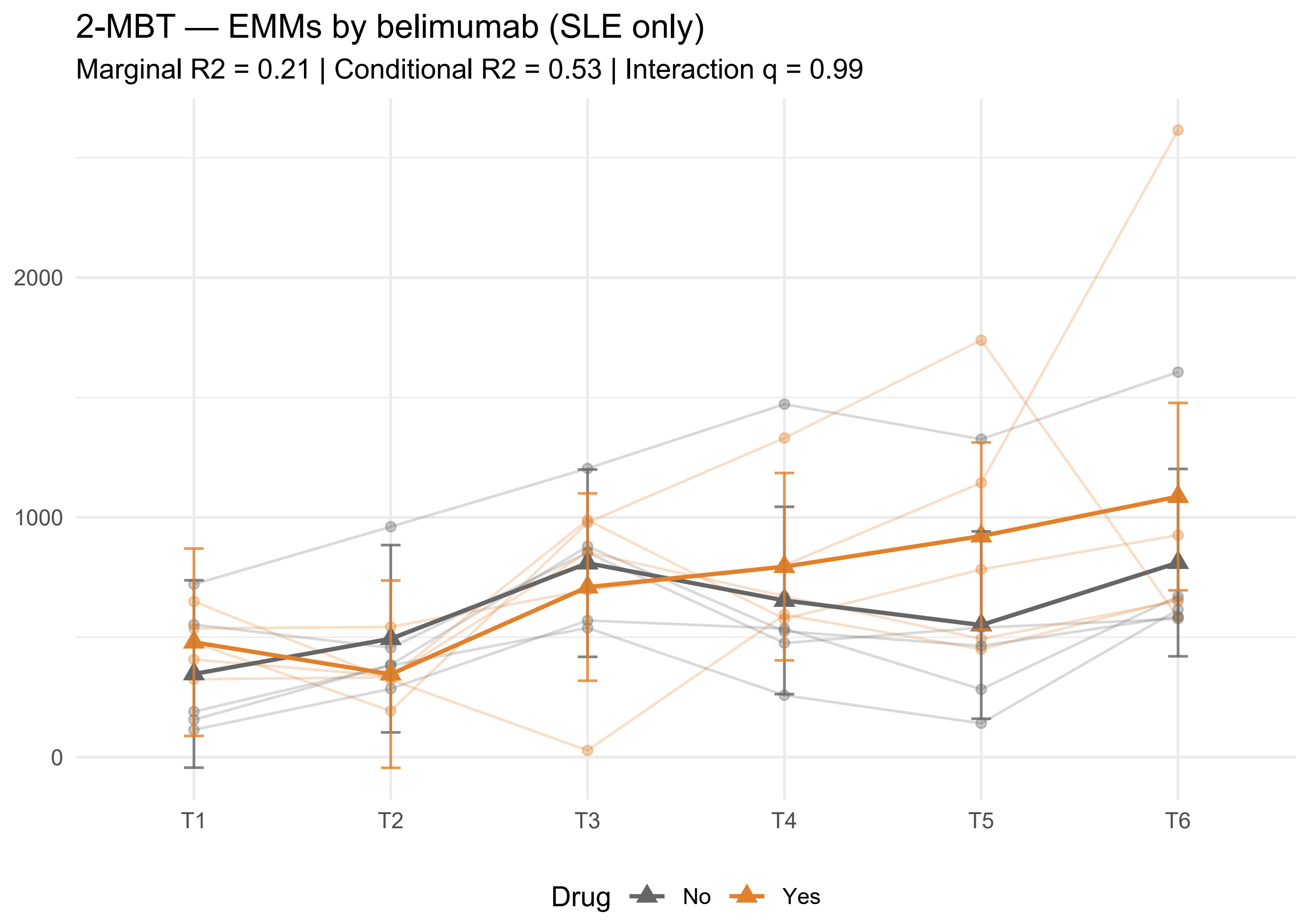

2-MBT — EMMs by belimumab (SLE only)
Marginal R2 = 0.21 | Conditional R2 = 0.53 | Interaction q = 0.99
2000
1000
0
T1
T2
T3
T4
T5
T6
Drug
No
Yes

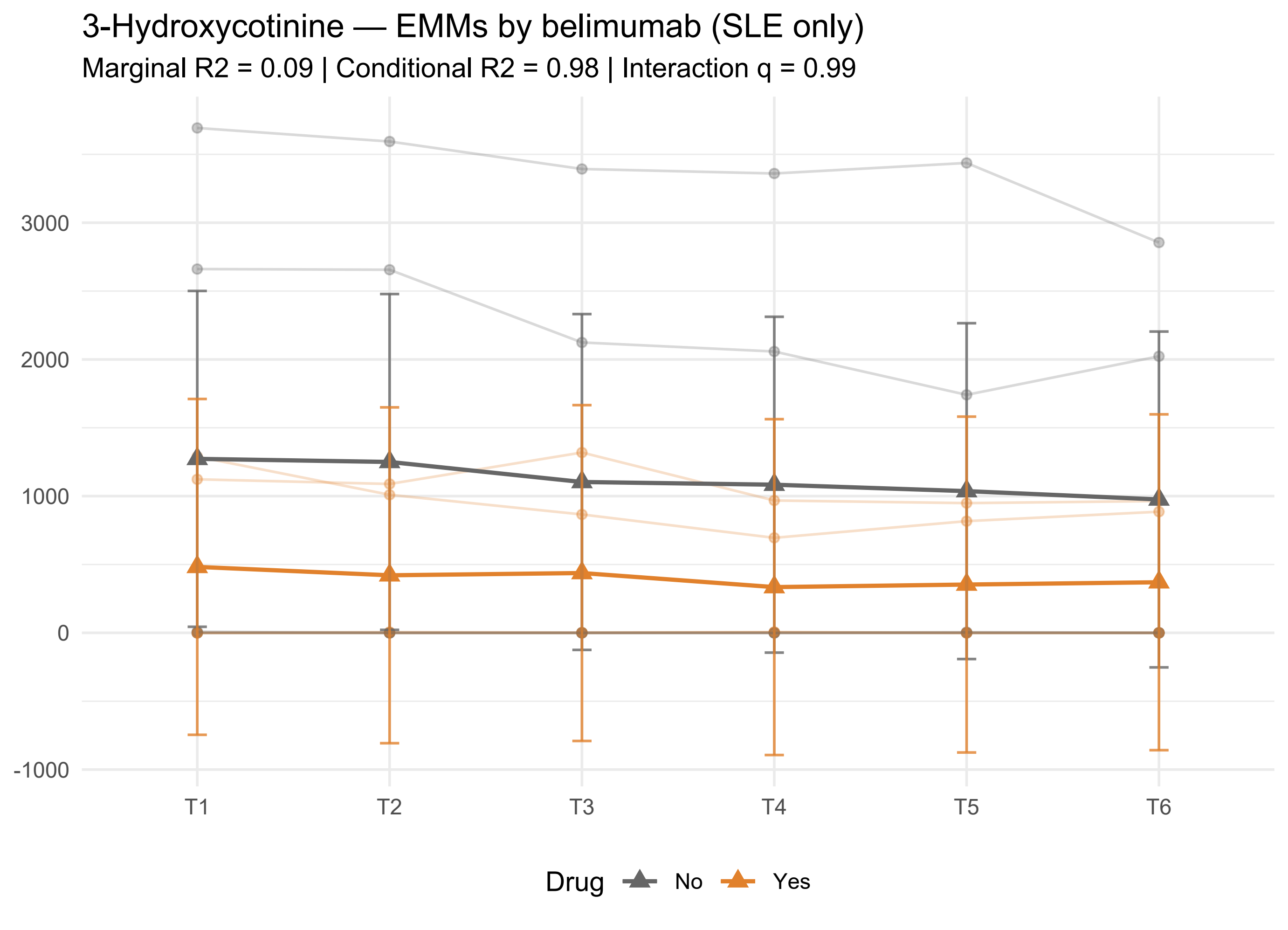

3-Hydroxycotinine — EMMs by belimumab (SLE only)
Marginal R2 = 0.09 | Conditional R2 = 0.98 | Interaction q = 0.99
3000
2000
1000
0
-1000
T1
T2
T3
T4
T5
T6
Drug
No
Yes

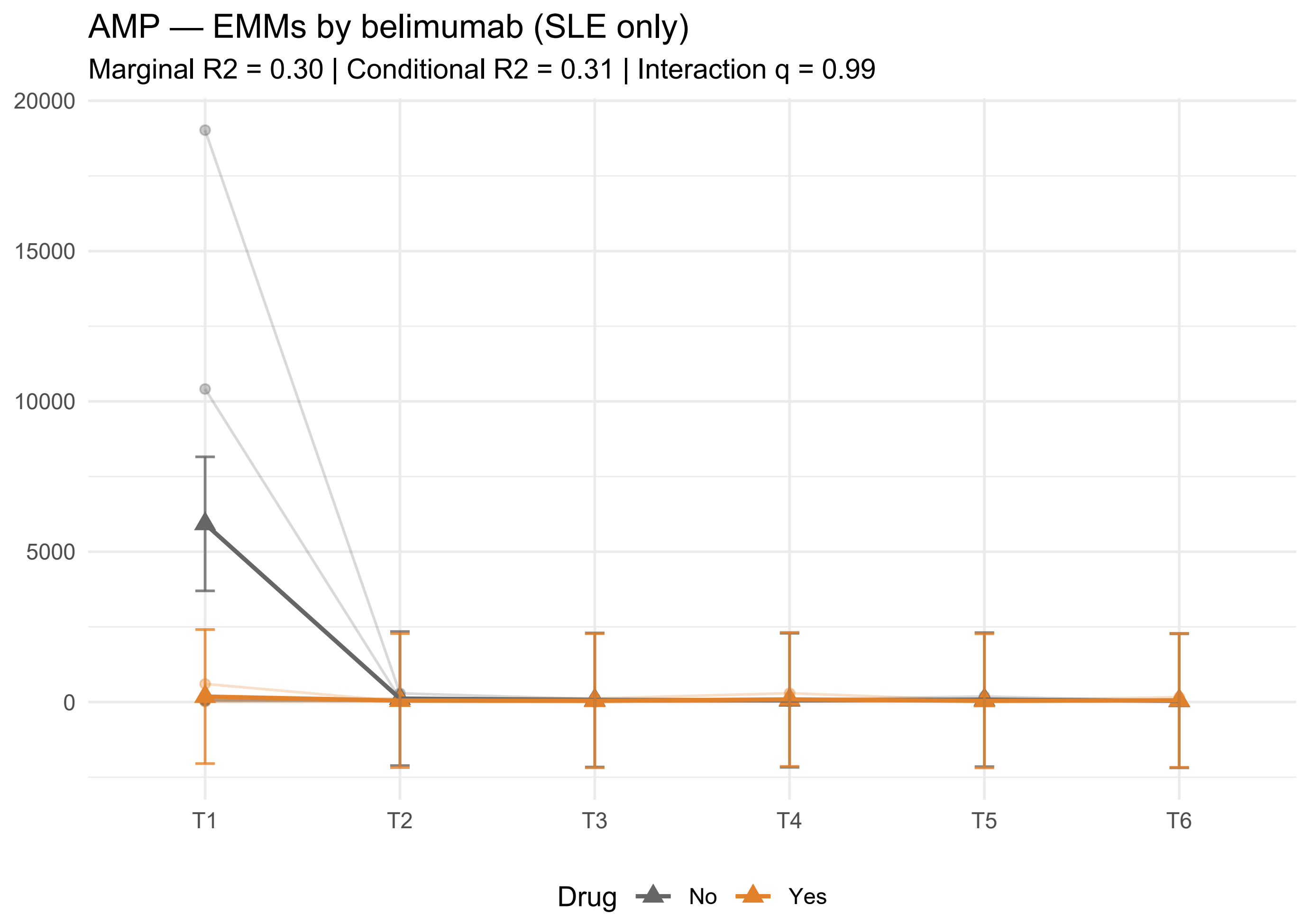
AMP — EMMs by belimumab (SLE only)
Marginal R2 = 0.30 | Conditional R2 = 0.31 | Interaction q = 0.99
20000
15000
10000
5000
0
T1
T2
T3
T4
T5
T6
Drug
No
Yes

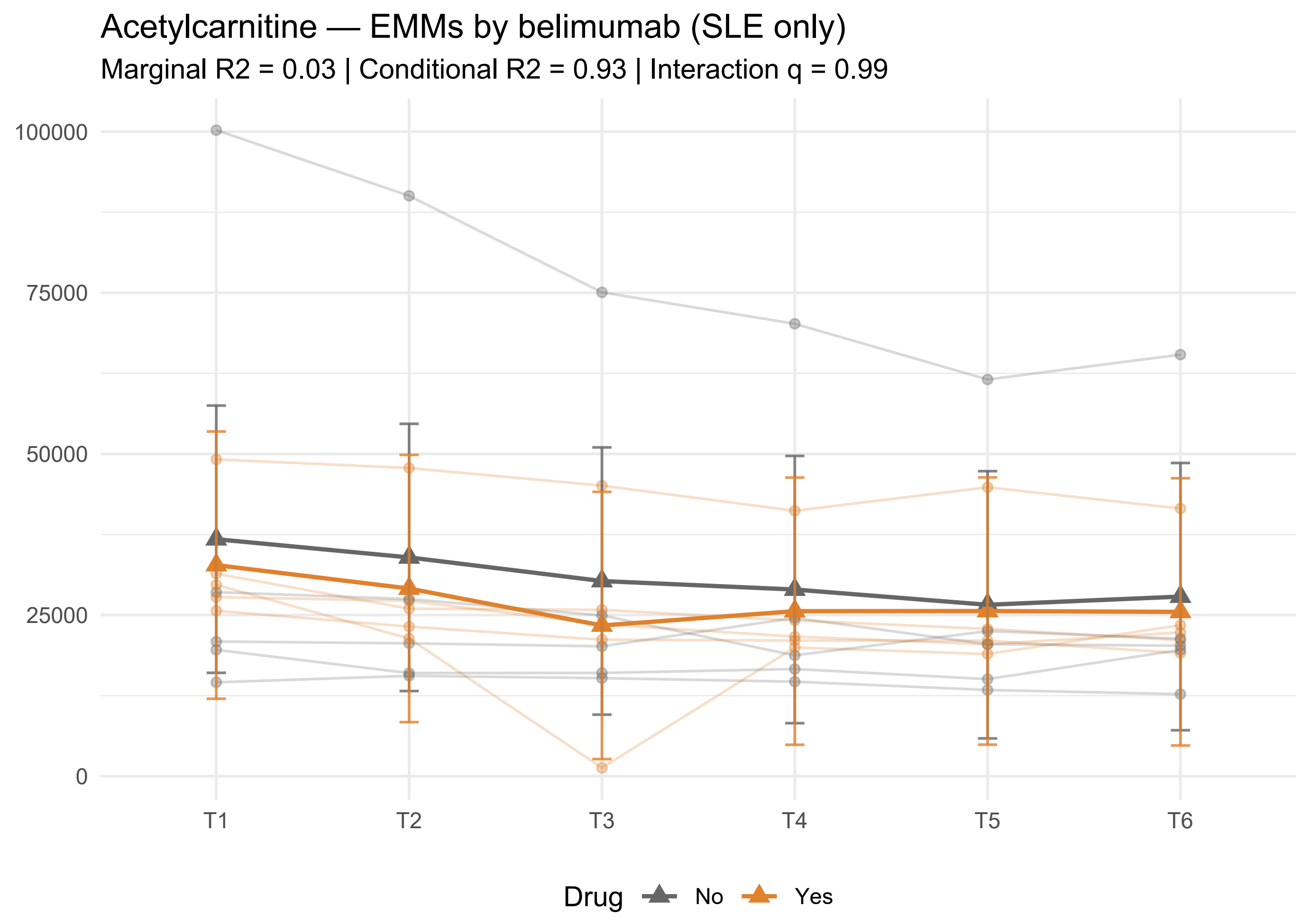
Acetylcarnitine — EMMs by belimumab (SLE only)
Marginal R2 = 0.03 | Conditional R2 = 0.93 | Interaction q = 0.99
100000
75000
50000
25000
0
T1
T2
T3
T4
T5
T6
Drug
No
Yes

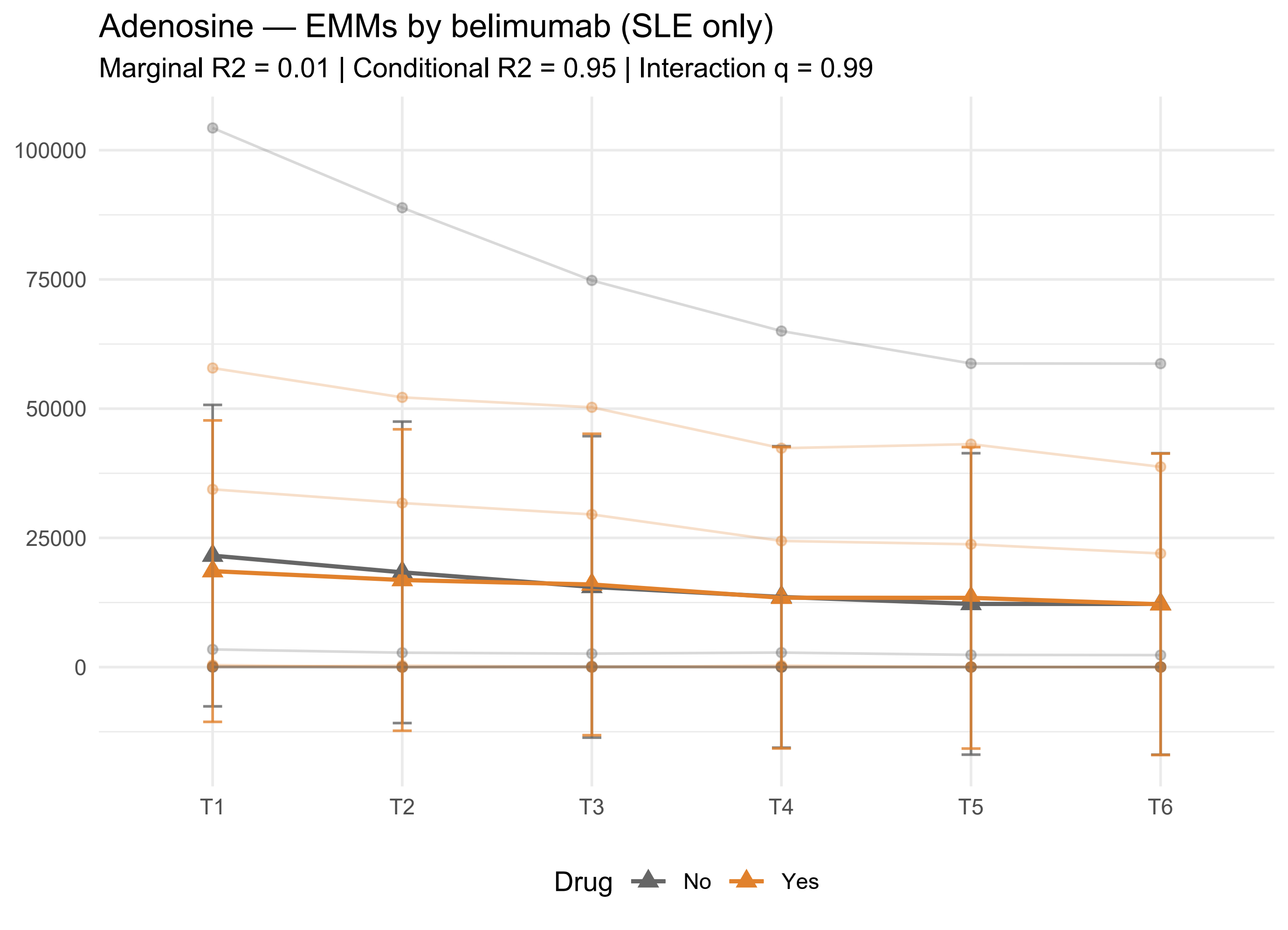

Adenosine — EMMs by belimumab (SLE only)
Marginal R2 = 0.01 | Conditional R2 = 0.95 | Interaction q = 0.99
100000
75000
50000
25000
0
T1
T2
T3
T4
T5
T6
Drug
No
Yes

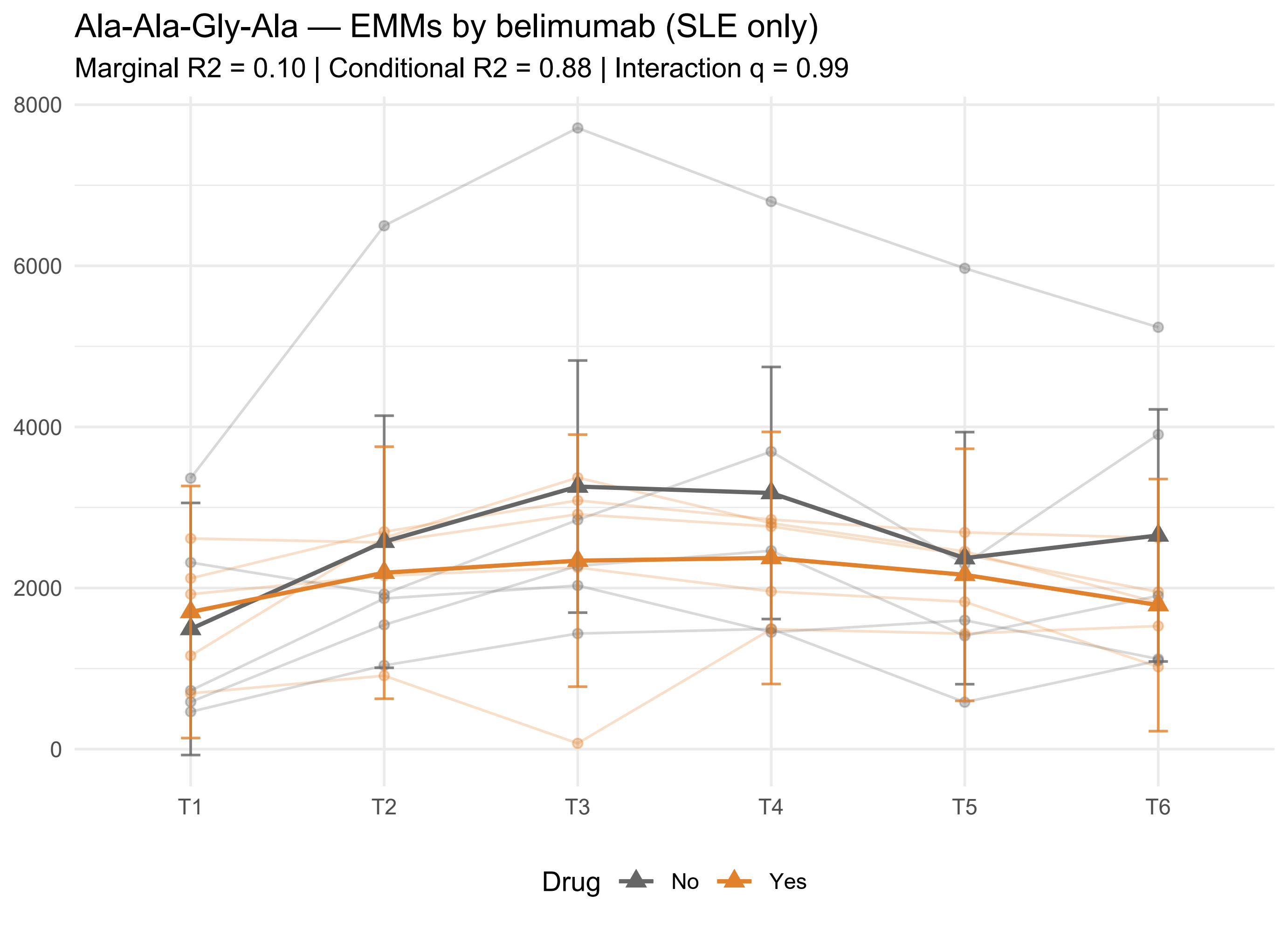
Ala-Ala-Gly-Ala — EMMs by belimumab (SLE only)
Marginal R2 = 0.10 | Conditional R2 = 0.88 | Interaction q = 0.99
8000
6000
4000
2000
0
T1
T2
T3
T4
T5
T6
Drug
No
Yes

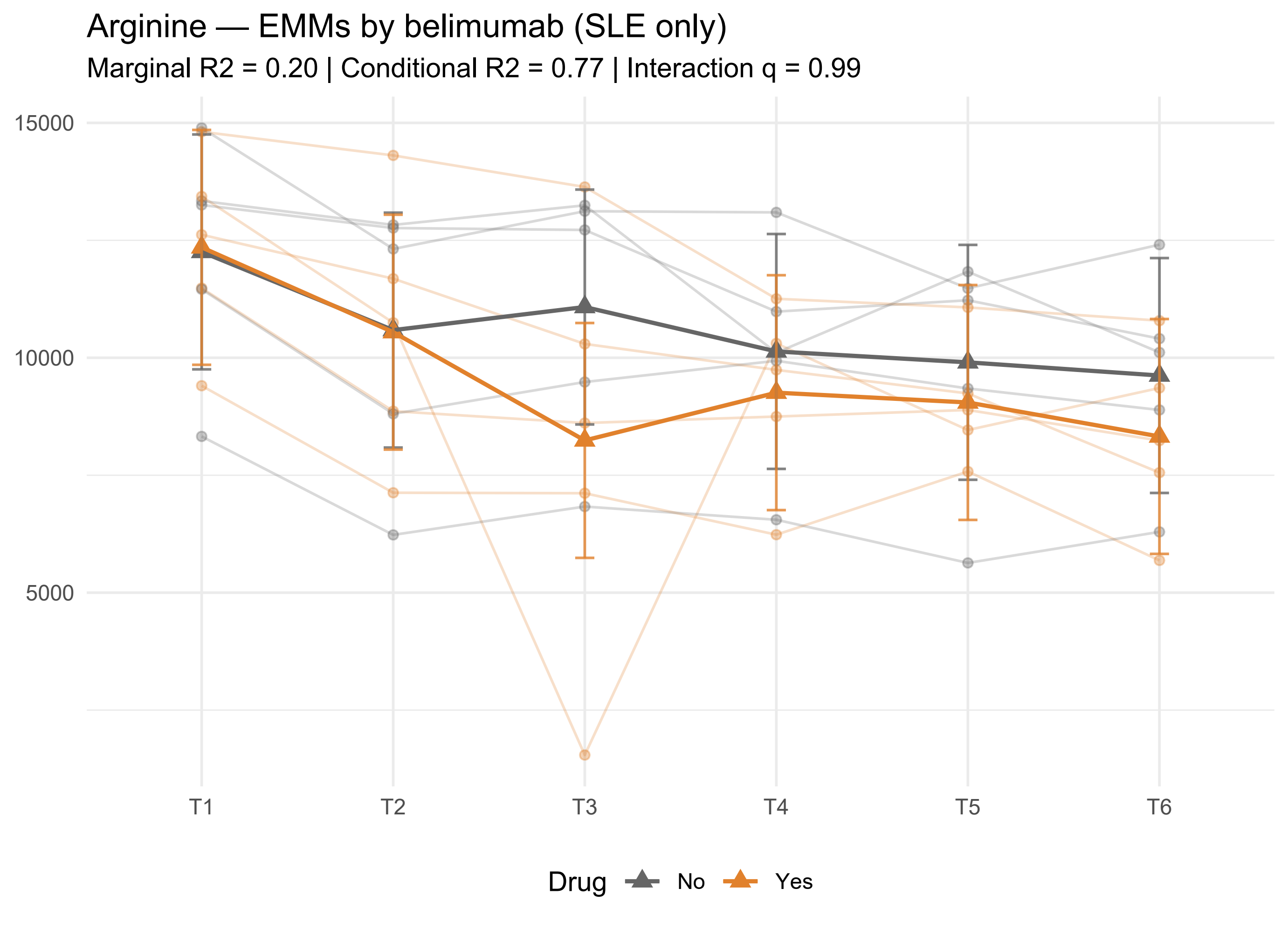
Arginine — EMMs by belimumab (SLE only)
Marginal R2 = 0.20 | Conditional R2 = 0.77 | Interaction q = 0.99
15000
10000
5000
T1
T2
T3
T4
T5
T6
Drug
No
Yes

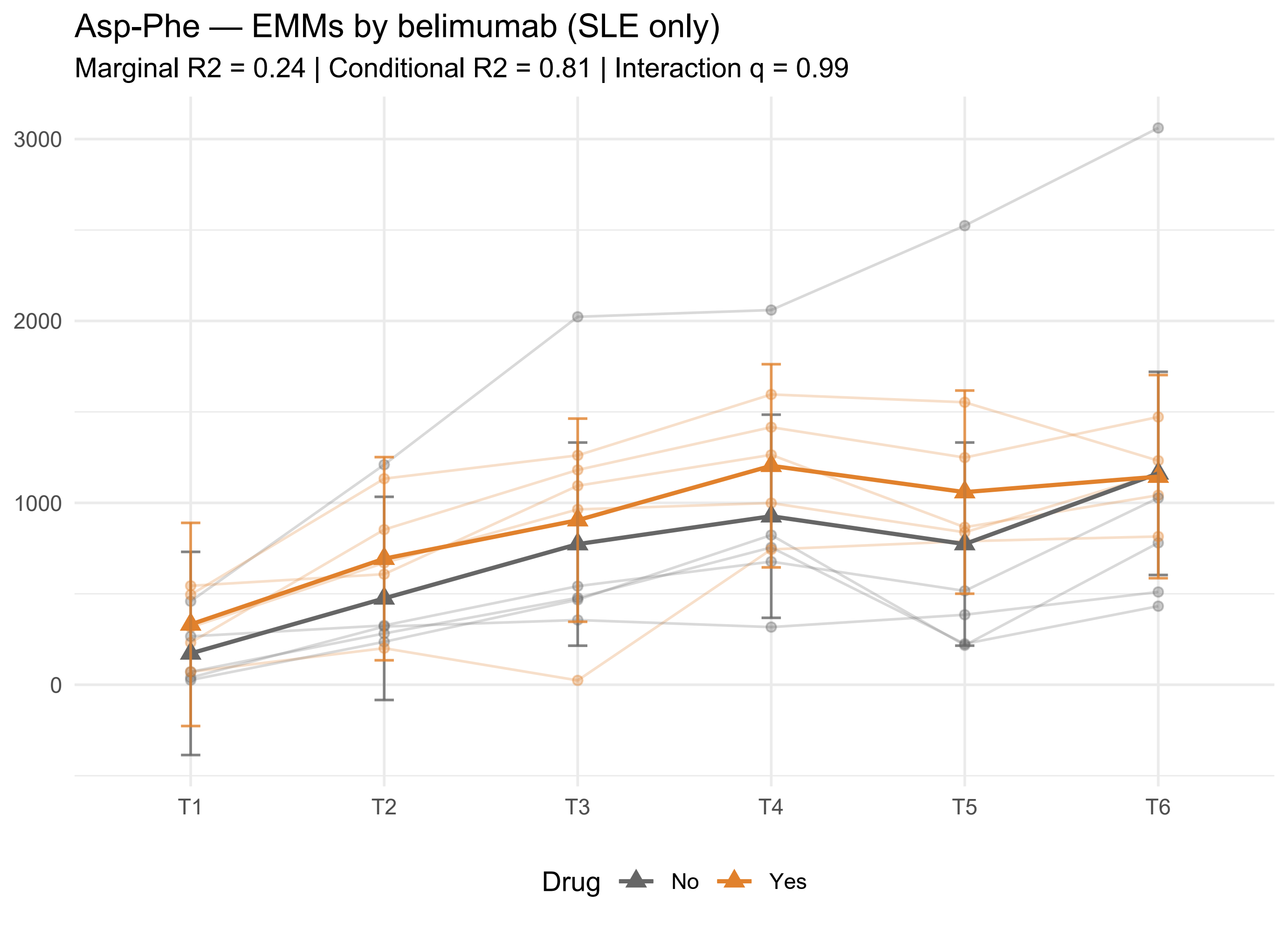
Asp-Phe — EMMs by belimumab (SLE only)
Marginal R2 = 0.24 | Conditional R2 = 0.81 | Interaction q = 0.99
3000
2000
1000
0
T1
T2
T3
T4
T5
T6
Drug
No
Yes

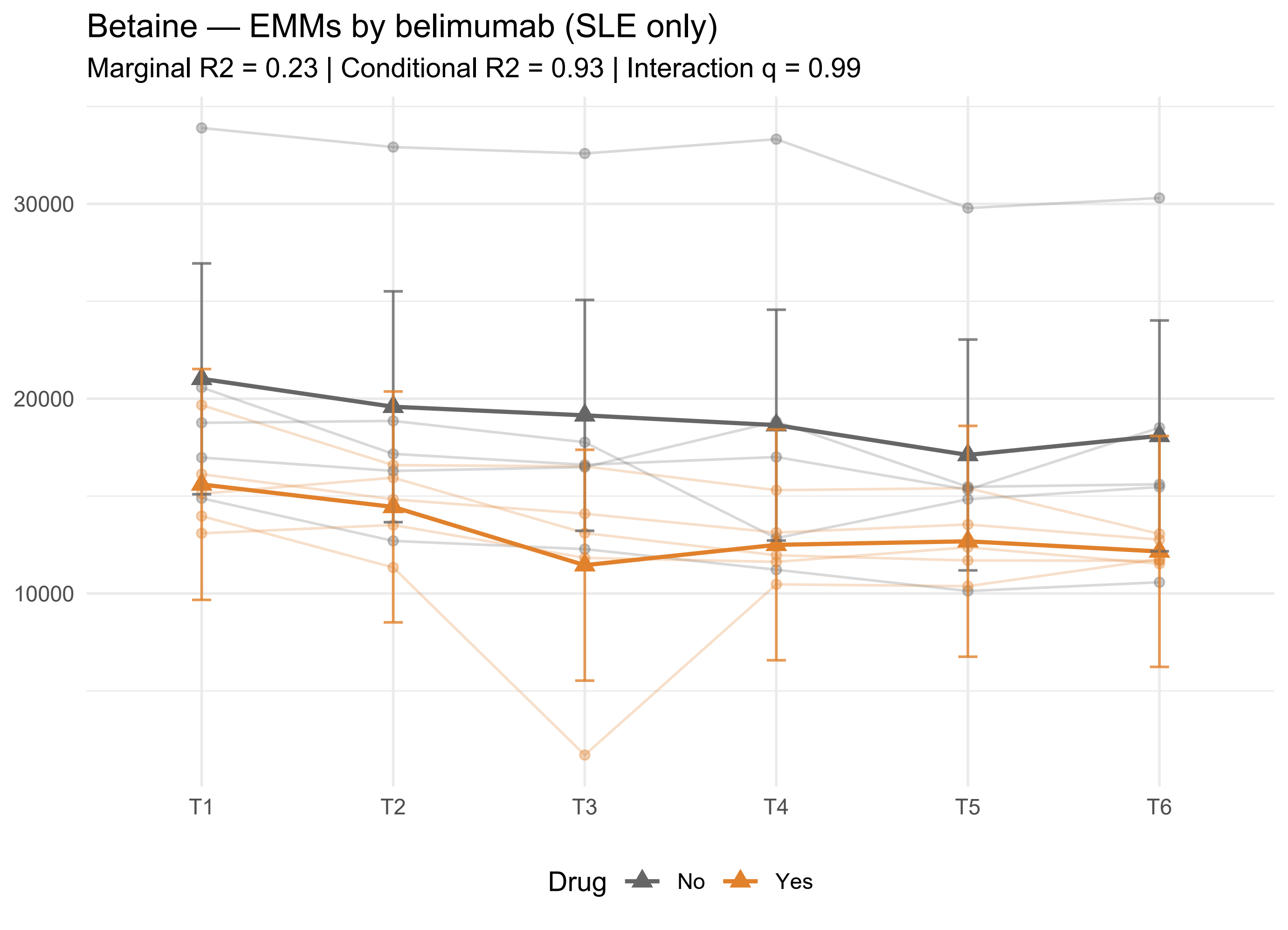

Betaine — EMMs by belimumab (SLE only)
Marginal R2 = 0.23 | Conditional R2 = 0.93 | Interaction q = 0.99
30000
20000
10000
T1
T2
T3
T4
T5
T6
Drug
No
Yes

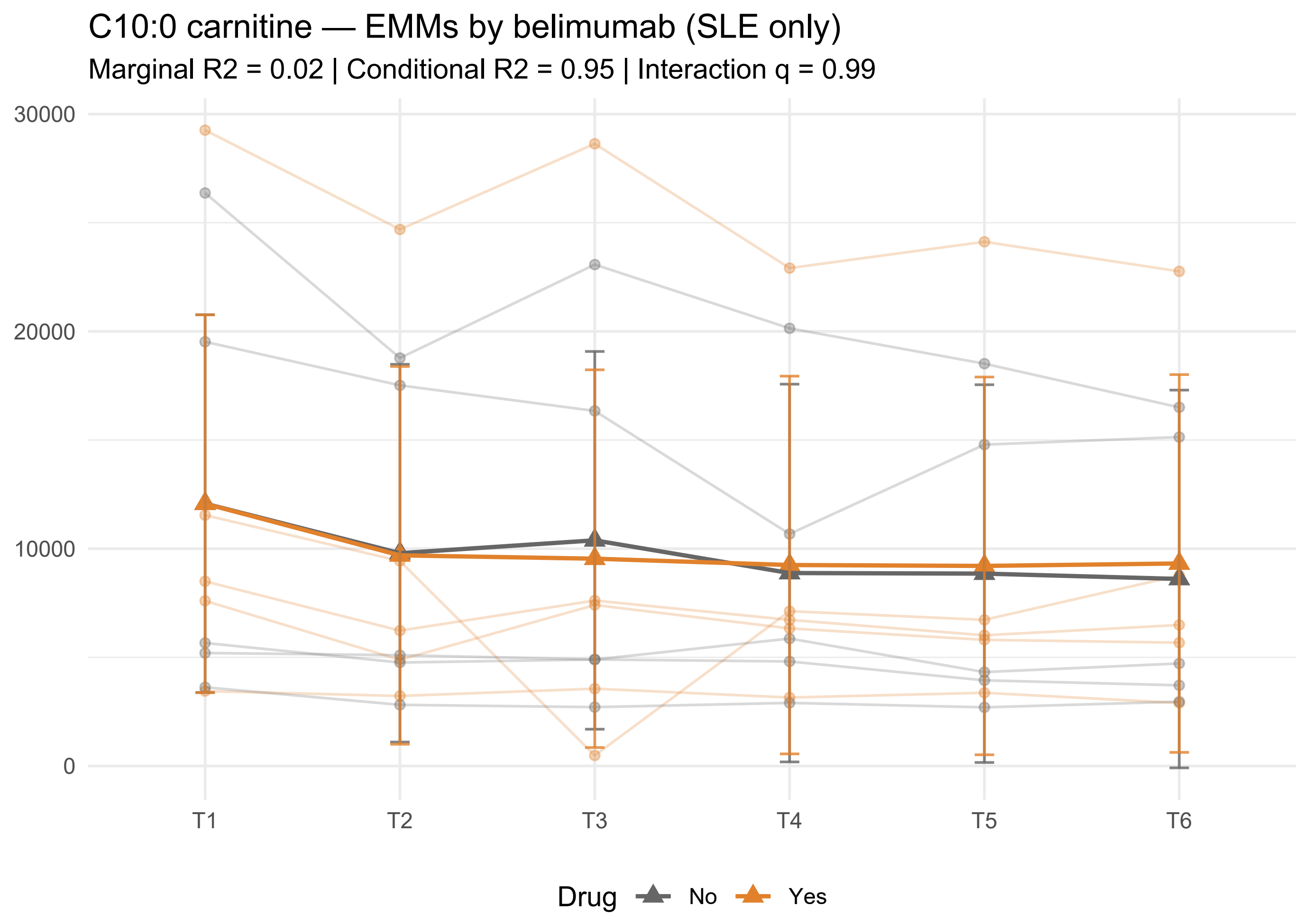

C10:0 carnitine — EMMs by belimumab (SLE only)
Marginal R2 = 0.02 | Conditional R2 = 0.95 | Interaction q = 0.99
30000
20000
10000
0
T1
T2
T3
T4
T5
T6
Drug
No
Yes

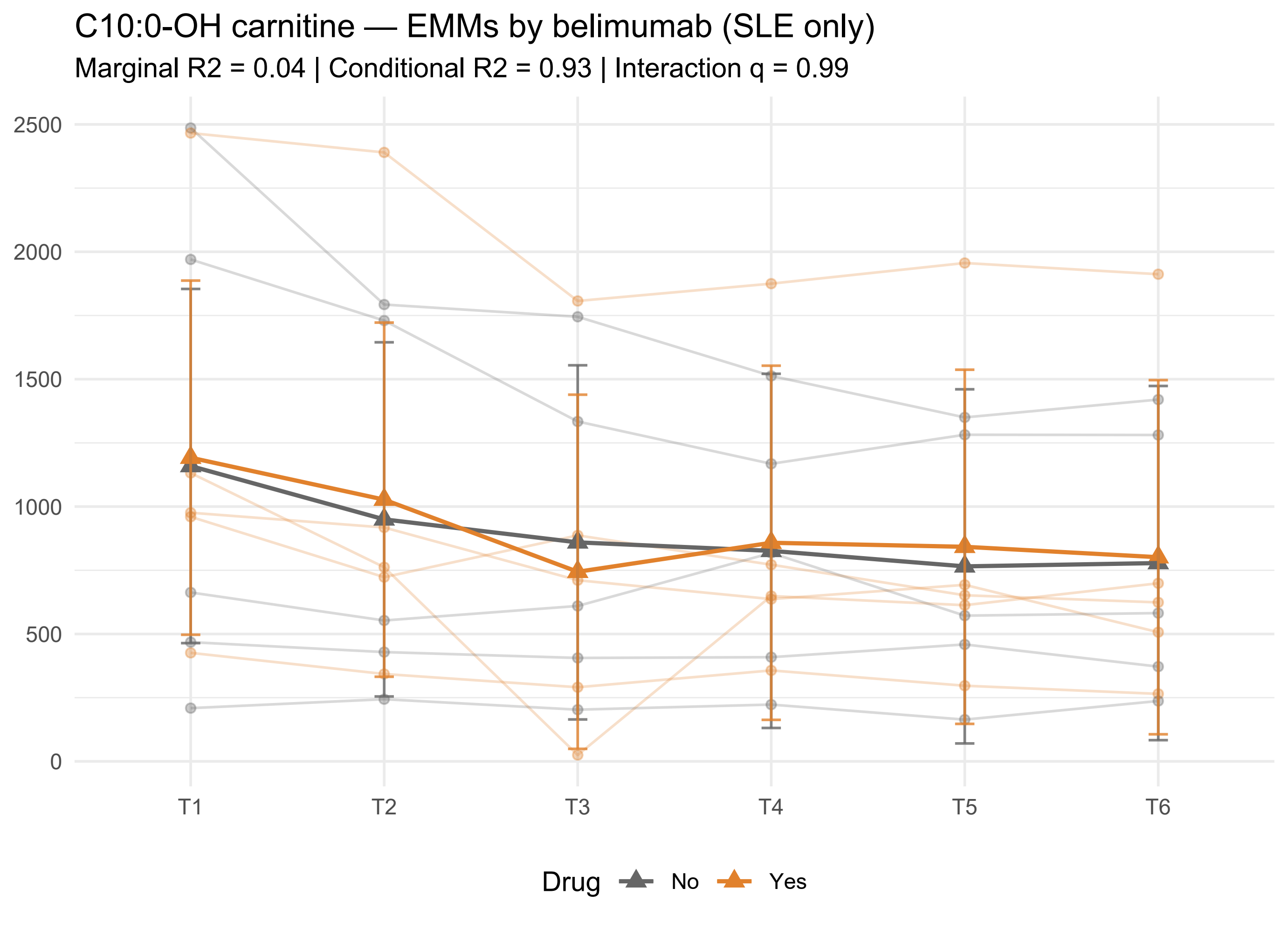

C10:0-OH carnitine — EMMs by belimumab (SLE only)
Marginal R2 = 0.04 | Conditional R2 = 0.93 | Interaction q = 0.99
2500
2000
1500
1000
500
0
T1
T2
T3
T4
T5
T6
Drug
No
Yes

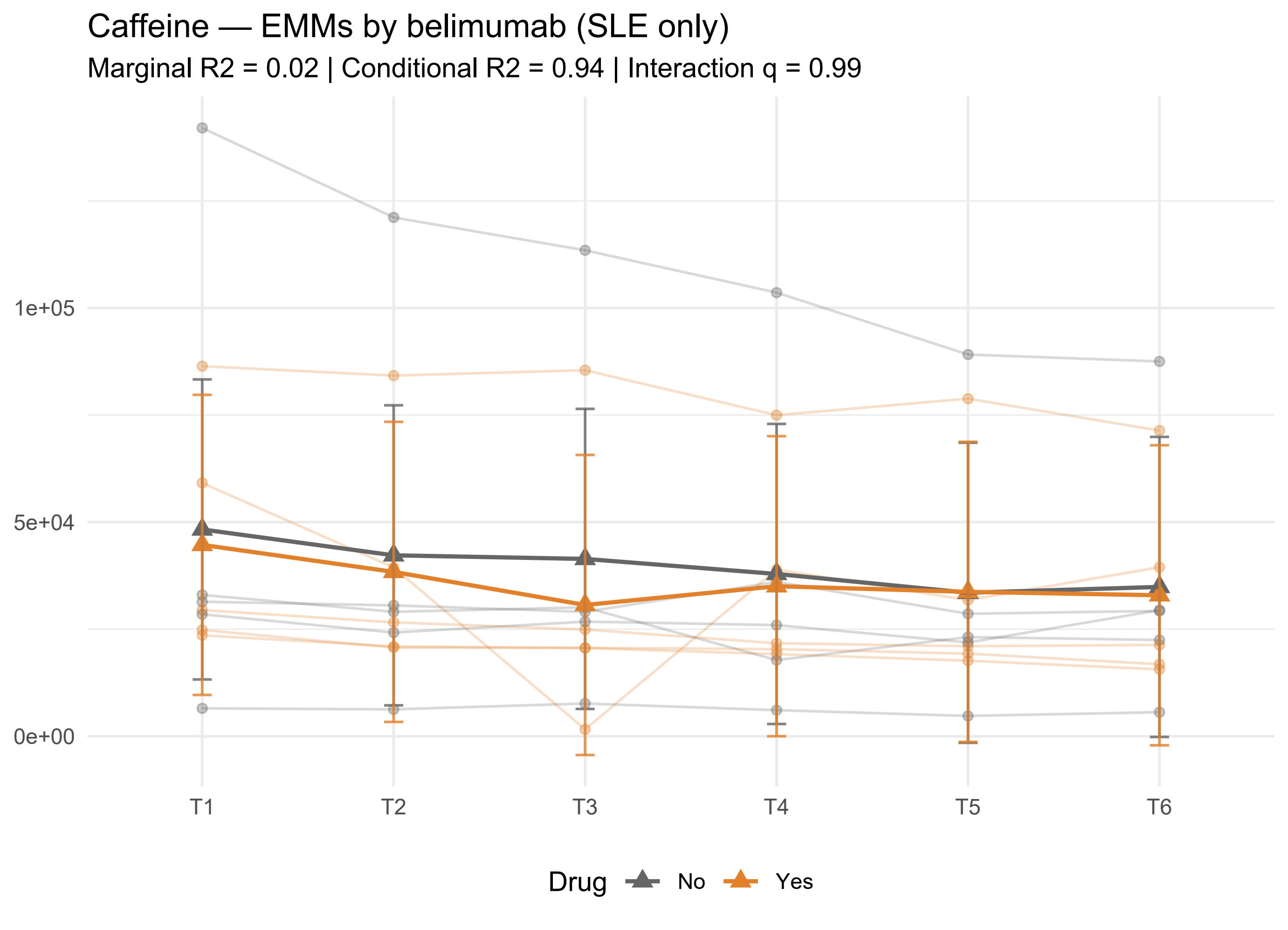

Caffeine — EMMs by belimumab (SLE only)
Marginal R2 = 0.02 | Conditional R2 = 0.94 | Interaction q = 0.99
1e+05
5e+04
0e+00
T1
T2
T3
T4
T5
T6
Drug
No
Yes

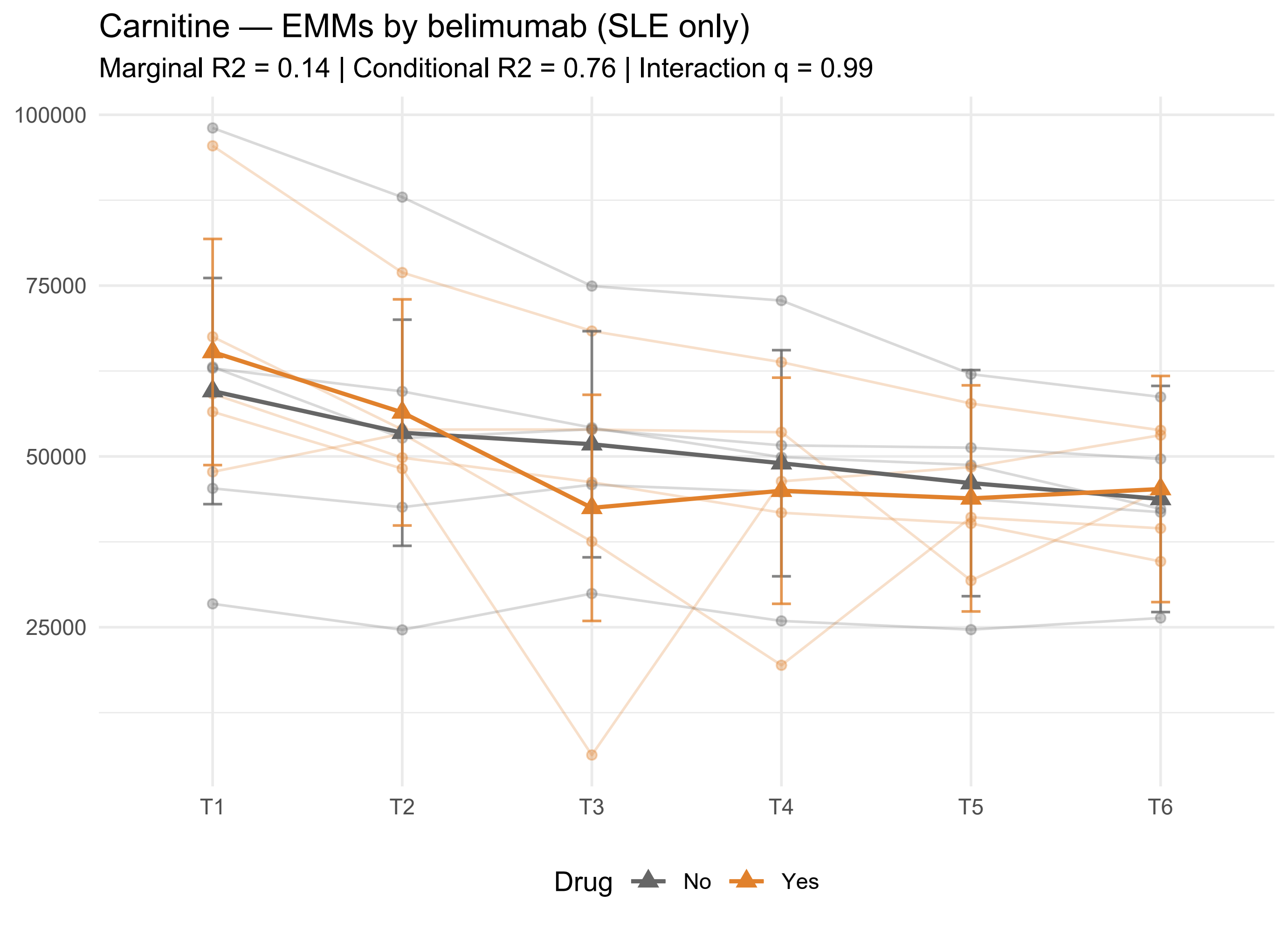

Carnitine — EMMs by belimumab (SLE only)
Marginal R2 = 0.14 | Conditional R2 = 0.76 | Interaction q = 0.99
100000
75000
50000
25000
T1
T2
T3
T4
T5
T6
Drug
No
Yes

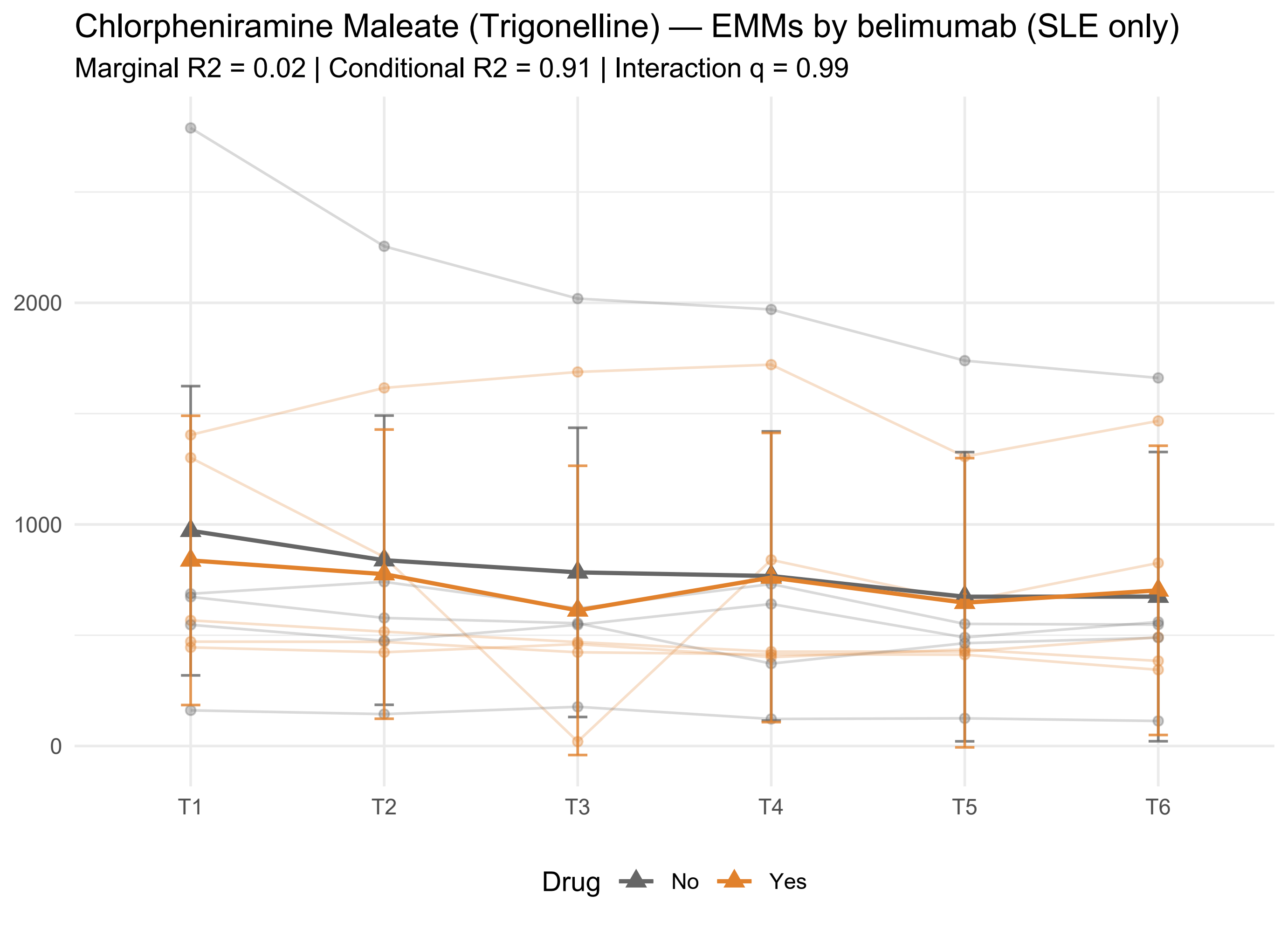

Chlorpheniramine Maleate (Trigonelline) — EMMs by belimumab (SLE only)
Marginal R2 = 0.02 | Conditional R2 = 0.91 | Interaction q = 0.99
2000
1000
0
T1
T2
T3
T4
T5
T6
Drug
No
Yes

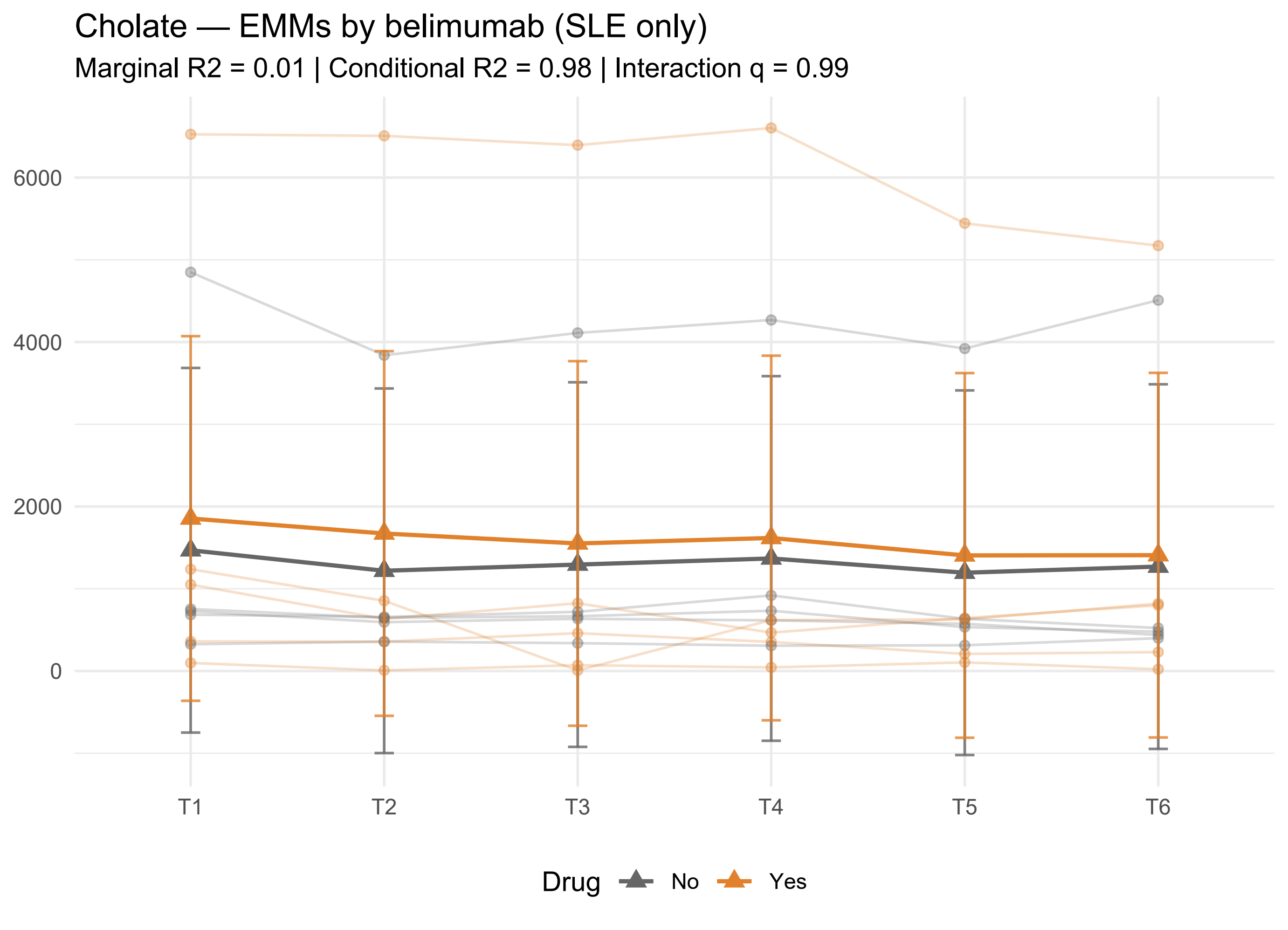

Cholate — EMMs by belimumab (SLE only)
Marginal R2 = 0.01 | Conditional R2 = 0.98 | Interaction q = 0.99
6000
4000
2000
0
T1
T2
T3
T4
T5
T6
Drug
No
Yes

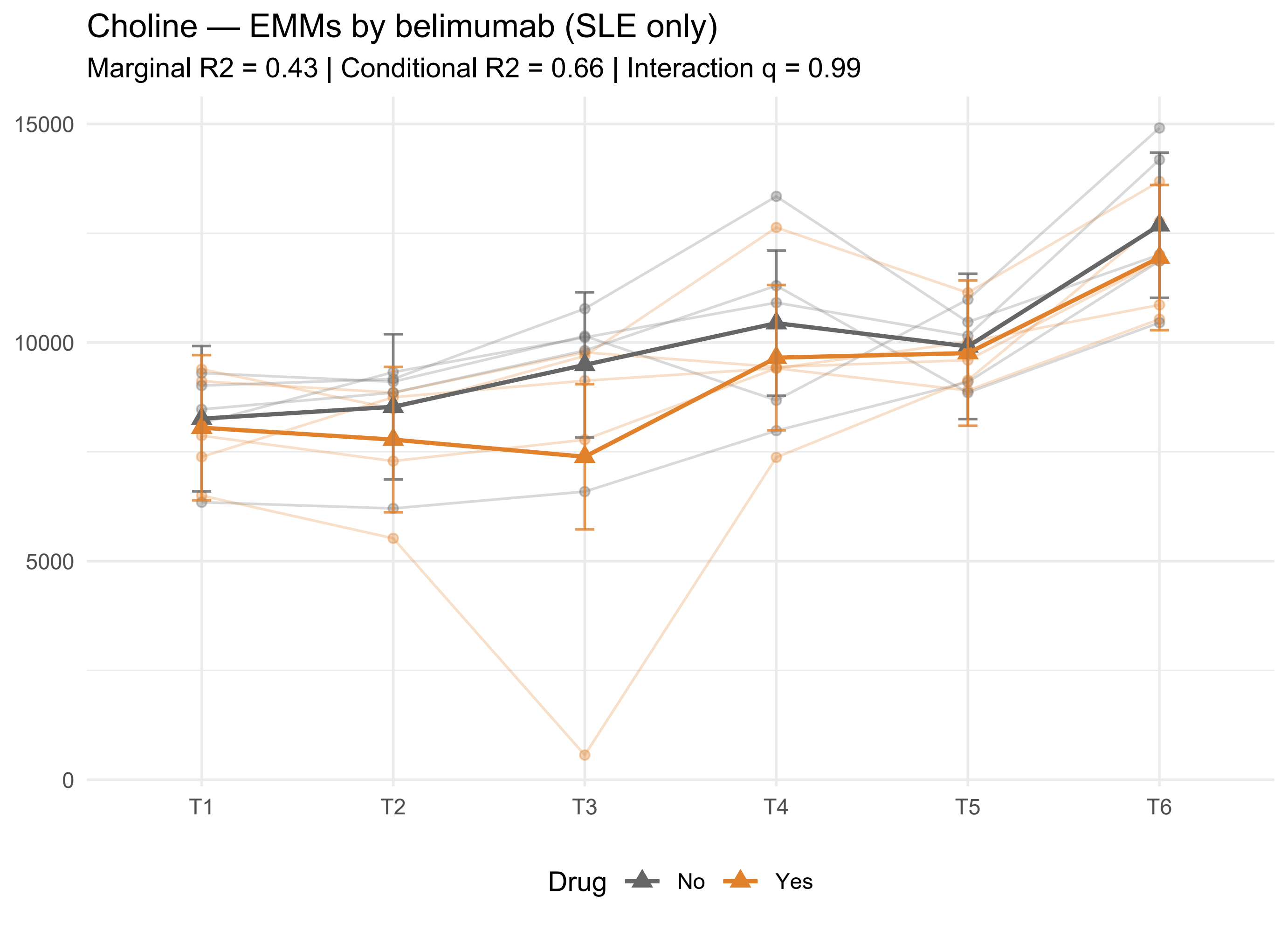

Choline — EMMs by belimumab (SLE only)
Marginal R2 = 0.43 | Conditional R2 = 0.66 | Interaction q = 0.99
15000
10000
5000
0
T1
T2
T3
T4
T5
T6
Drug
No
Yes

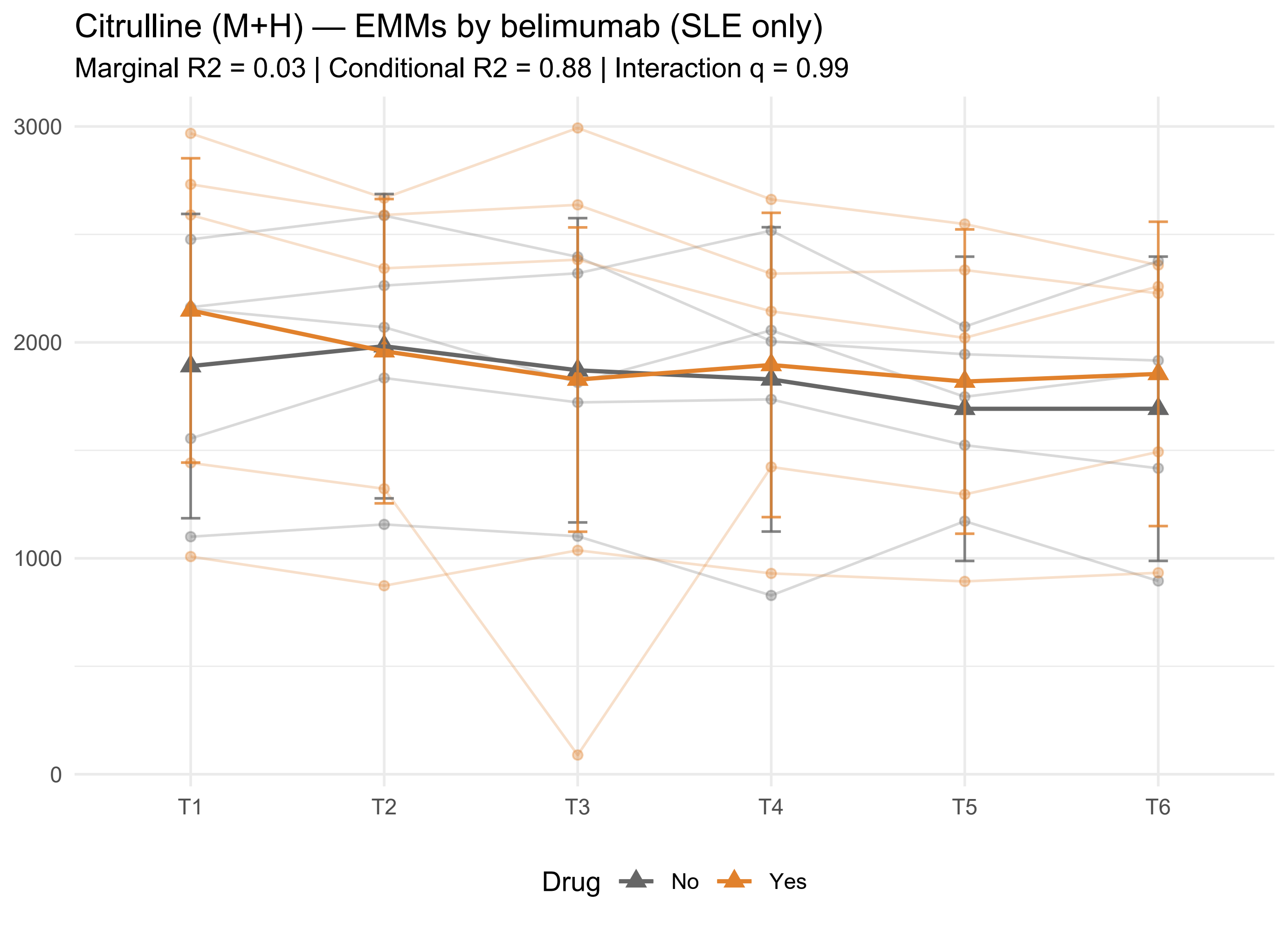

Citrulline (M+H) — EMMs by belimumab (SLE only)
Marginal R2 = 0.03 | Conditional R2 = 0.88 | Interaction q = 0.99
3000
2000
1000
0
T1
T2
T3
T4
T5
T6
Drug
No
Yes

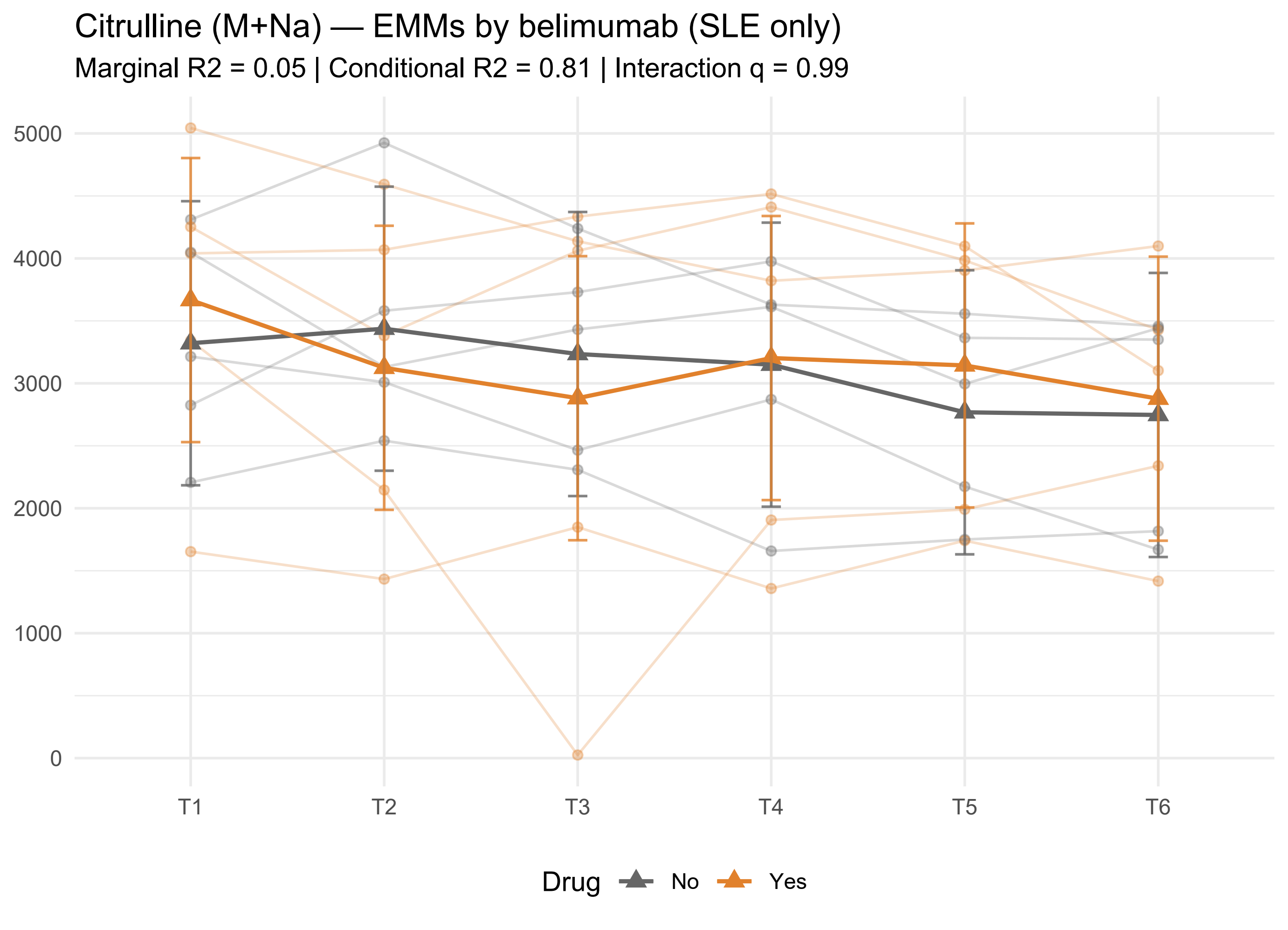
Citrulline (M+Na) — EMMs by belimumab (SLE only)
Marginal R2 = 0.05 | Conditional R2 = 0.81 | Interaction q = 0.99
5000
4000
3000
2000
1000
0
T1
T2
T3
T4
T5
T6
Drug
No
Yes

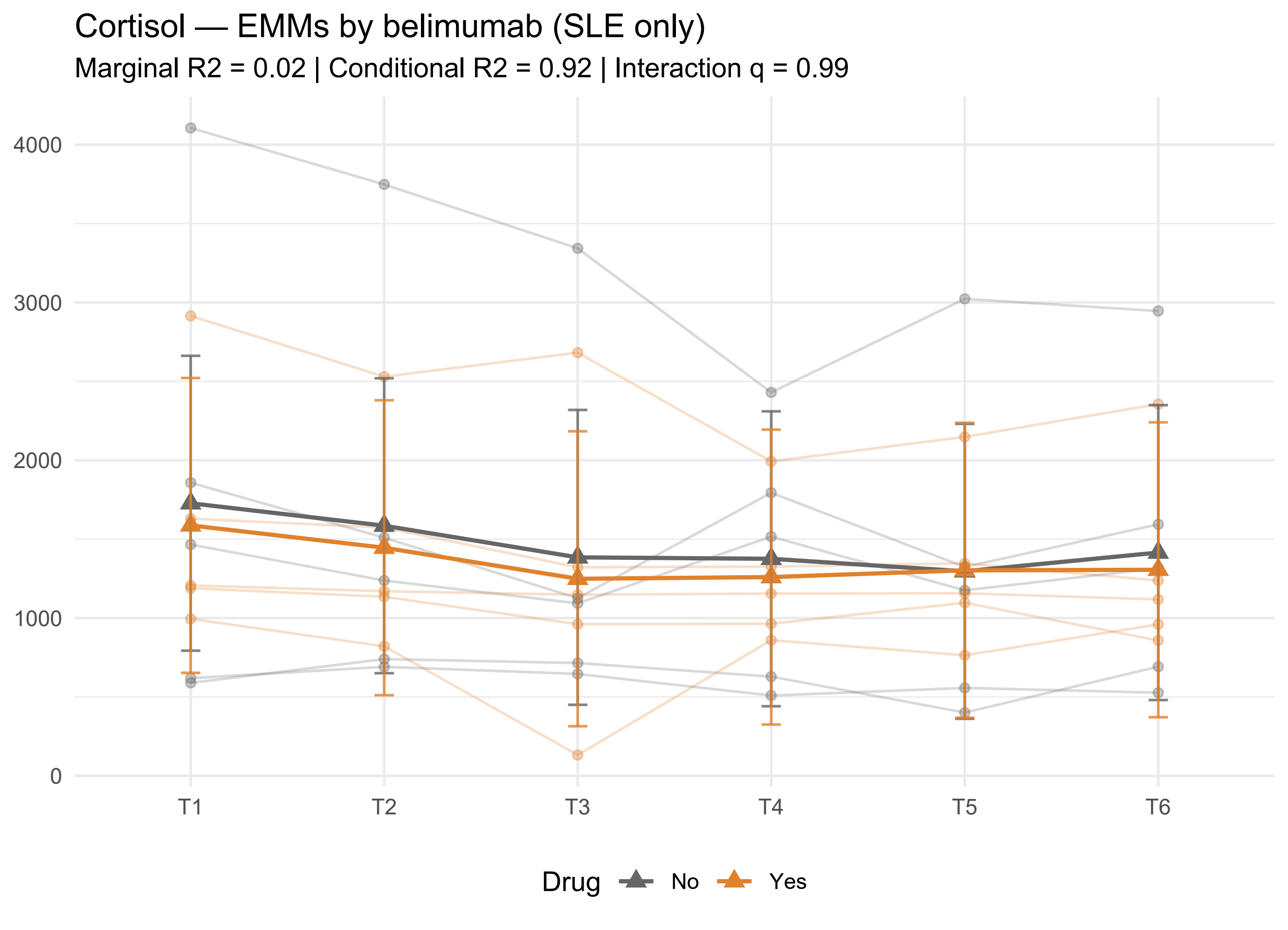

Cortisol — EMMs by belimumab (SLE only)
Marginal R2 = 0.02 | Conditional R2 = 0.92 | Interaction q = 0.99
4000
3000
2000
1000
0
T1
T2
T3
T4
T5
T6
Drug
No
Yes

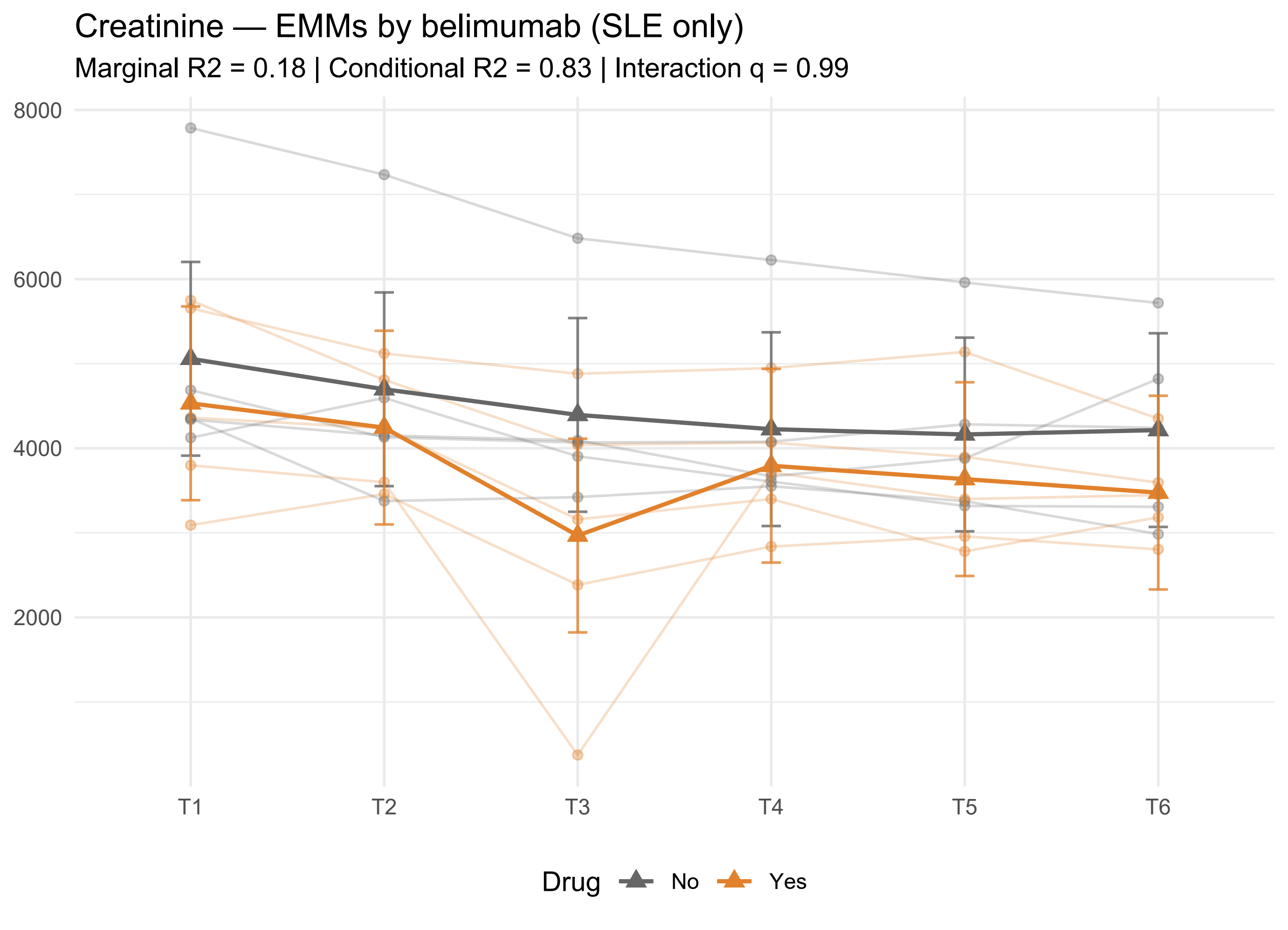

Creatinine — EMMs by belimumab (SLE only)
Marginal R2 = 0.18 | Conditional R2 = 0.83 | Interaction q = 0.99
8000
6000
4000
2000
T1
T2
T3
T4
T5
T6
Drug
No
Yes

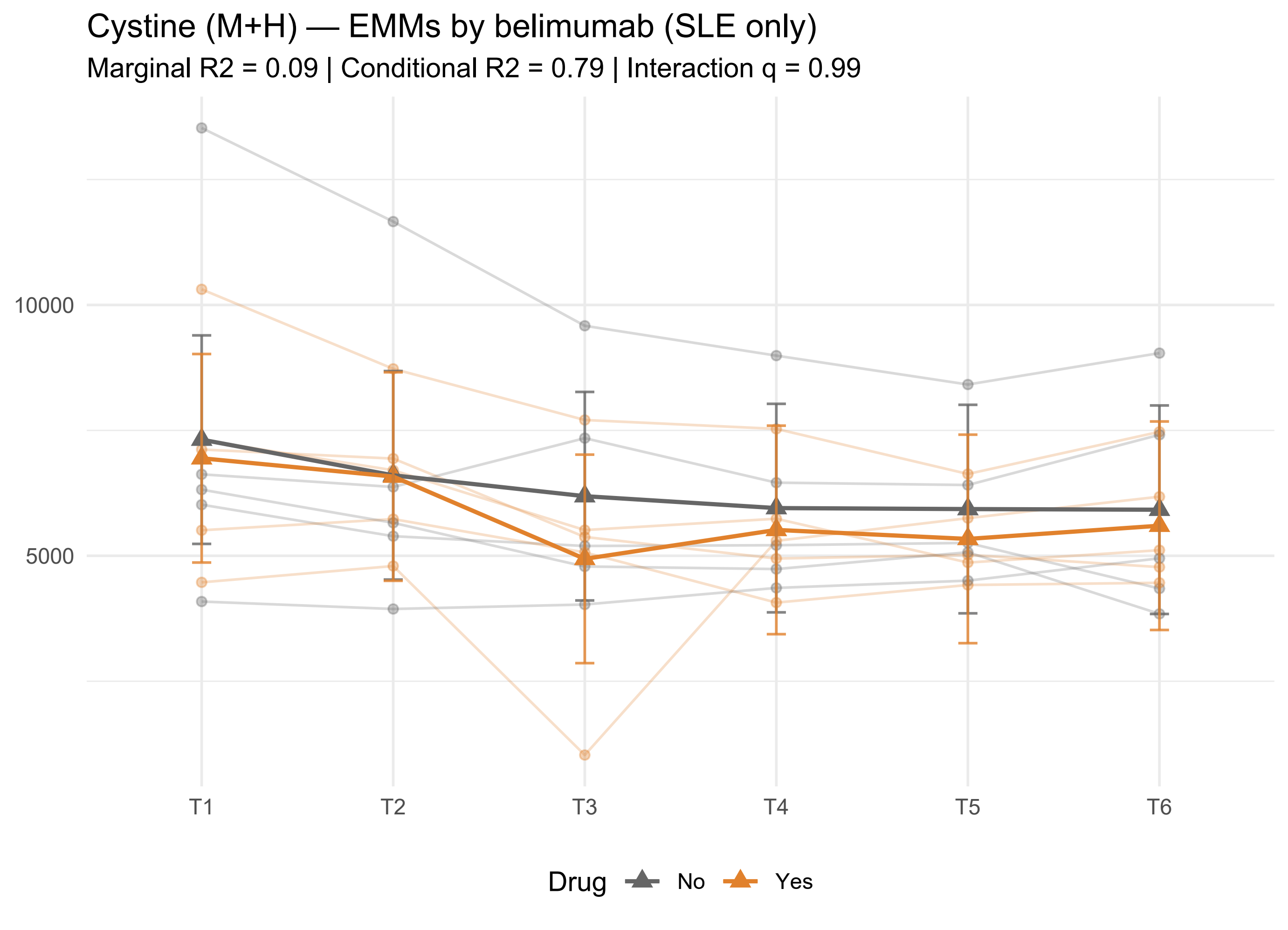

Cystine (M+H) — EMMs by belimumab (SLE only)
Marginal R2 = 0.09 | Conditional R2 = 0.79 | Interaction q = 0.99
10000
5000
T1
T2
T3
T4
T5
T6
Drug
No
Yes

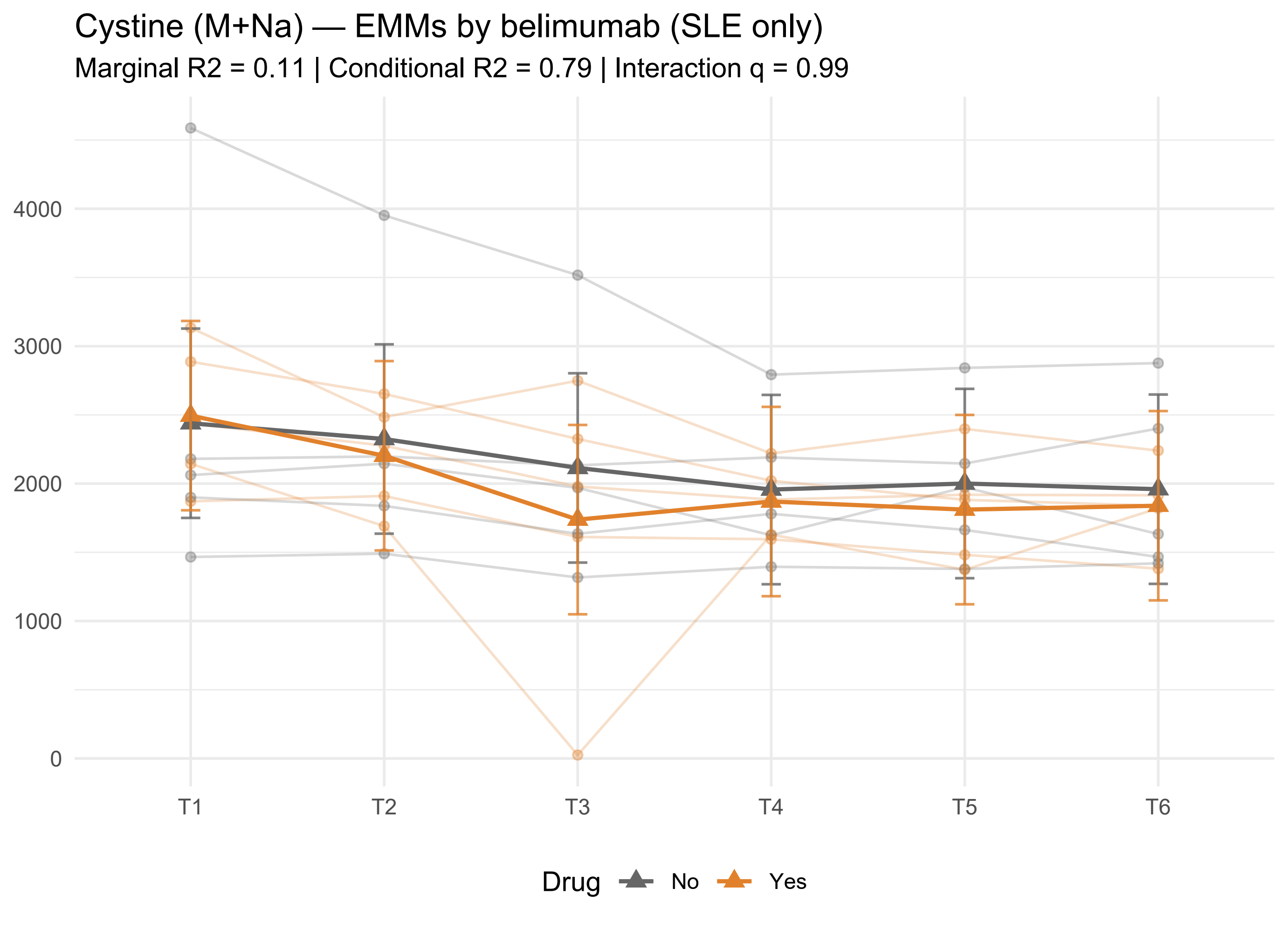

Cystine (M+Na) — EMMs by belimumab (SLE only)
Marginal R2 = 0.11 | Conditional R2 = 0.79 | Interaction q = 0.99
4000
3000
2000
1000
0
T1
T2
T3
T4
T5
T6
Drug
No
Yes

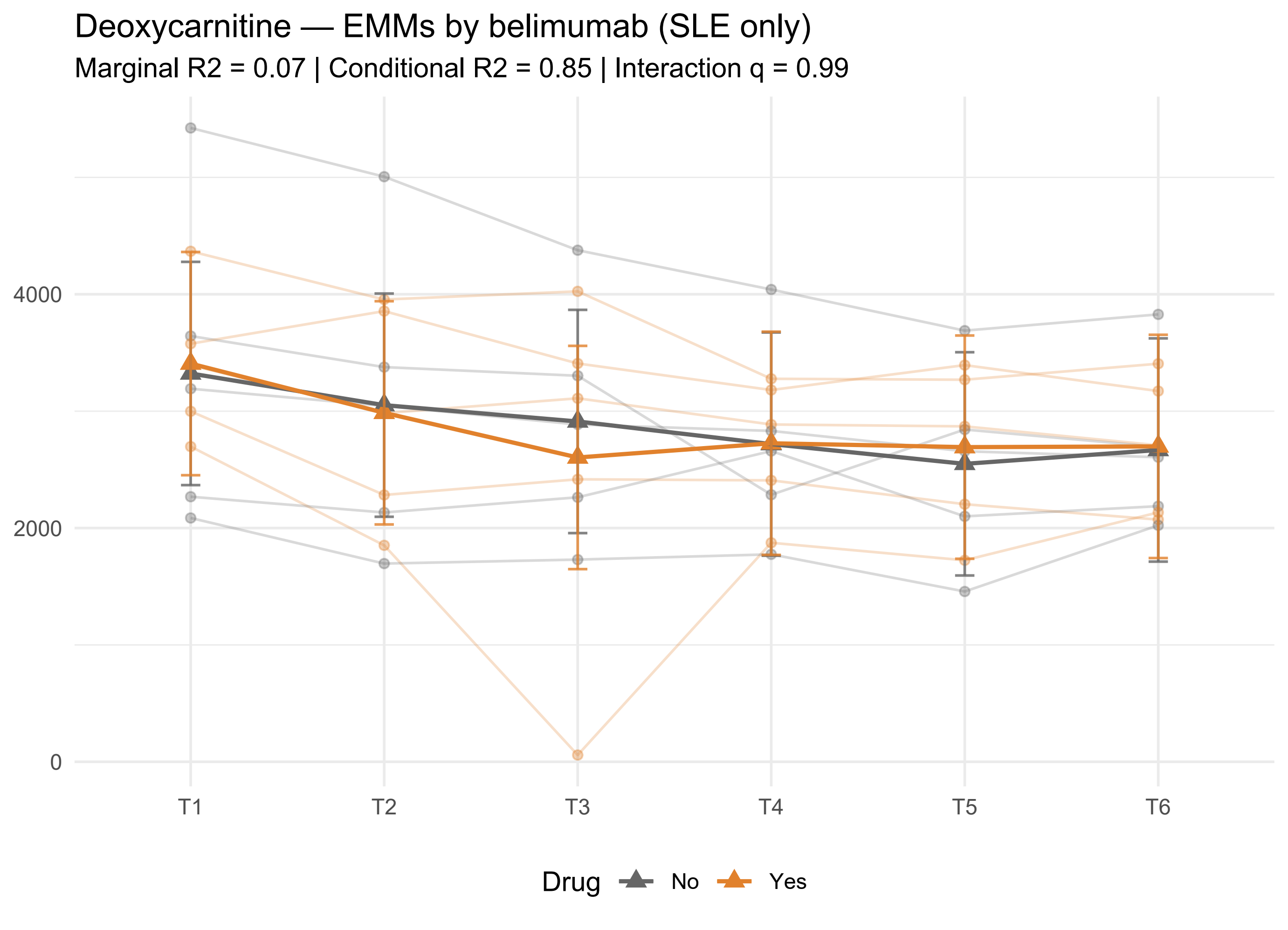
Deoxycarnitine — EMMs by belimumab (SLE only)
Marginal R2 = 0.07 | Conditional R2 = 0.85 | Interaction q = 0.99
4000
2000
0
T1
T2
T3
T4
T5
T6
Drug
No
Yes

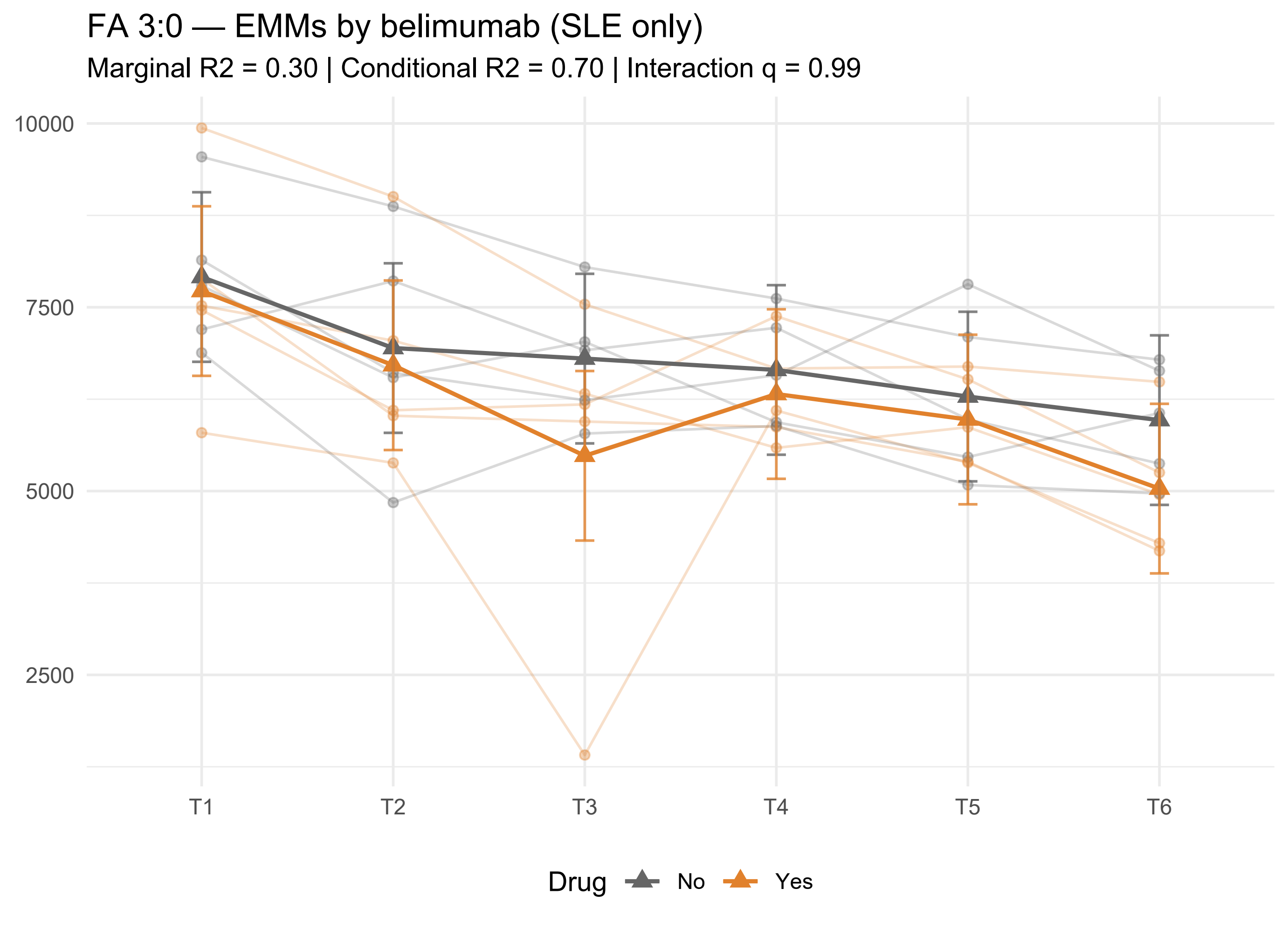

FA 3:0 — EMMs by belimumab (SLE only)
Marginal R2 = 0.30 | Conditional R2 = 0.70 | Interaction q = 0.99
10000
7500
5000
2500
T1
T2
T3
T4
T5
T6
Drug
No
Yes

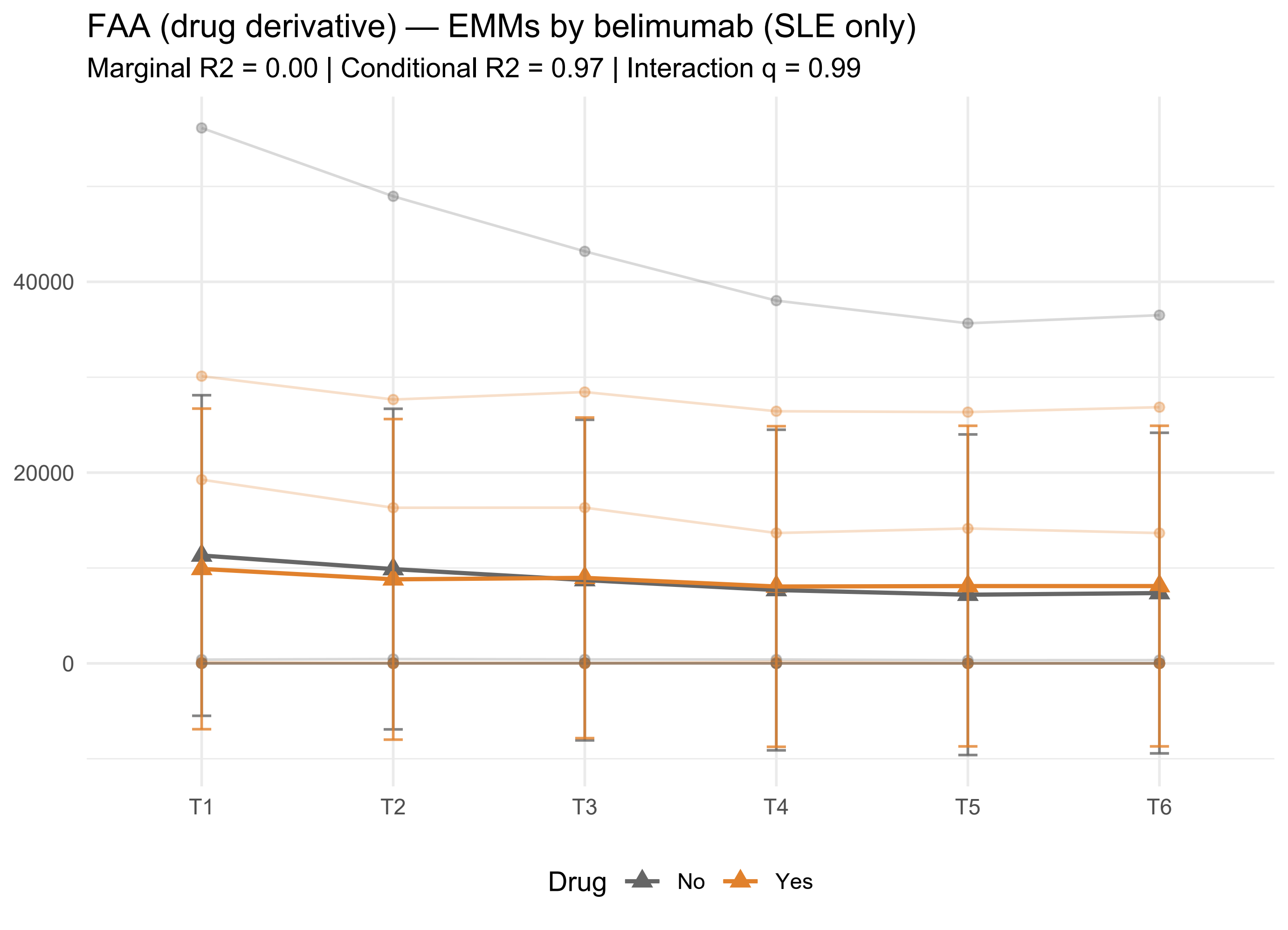

FAA (drug derivative) — EMMs by belimumab (SLE only)
Marginal R2 = 0.00 | Conditional R2 = 0.97 | Interaction q = 0.99
40000
20000
0
T1
T2
T3
T4
T5
T6
Drug
No
Yes

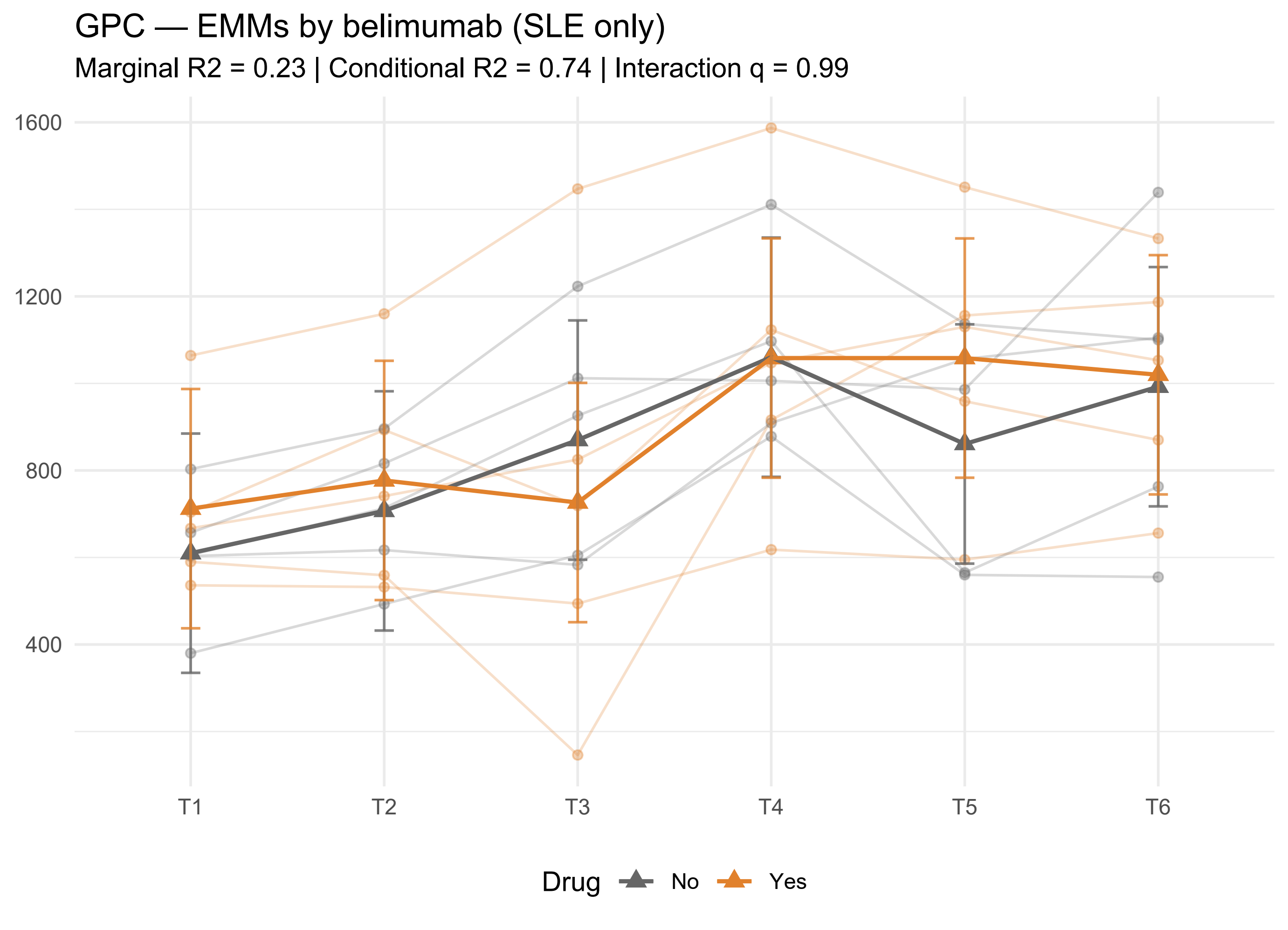
GPC — EMMs by belimumab (SLE only)
Marginal R2 = 0.23 | Conditional R2 = 0.74 | Interaction q = 0.99
1600
1200
800
400
T1
T2
T3
T4
T5
T6
Drug
No
Yes

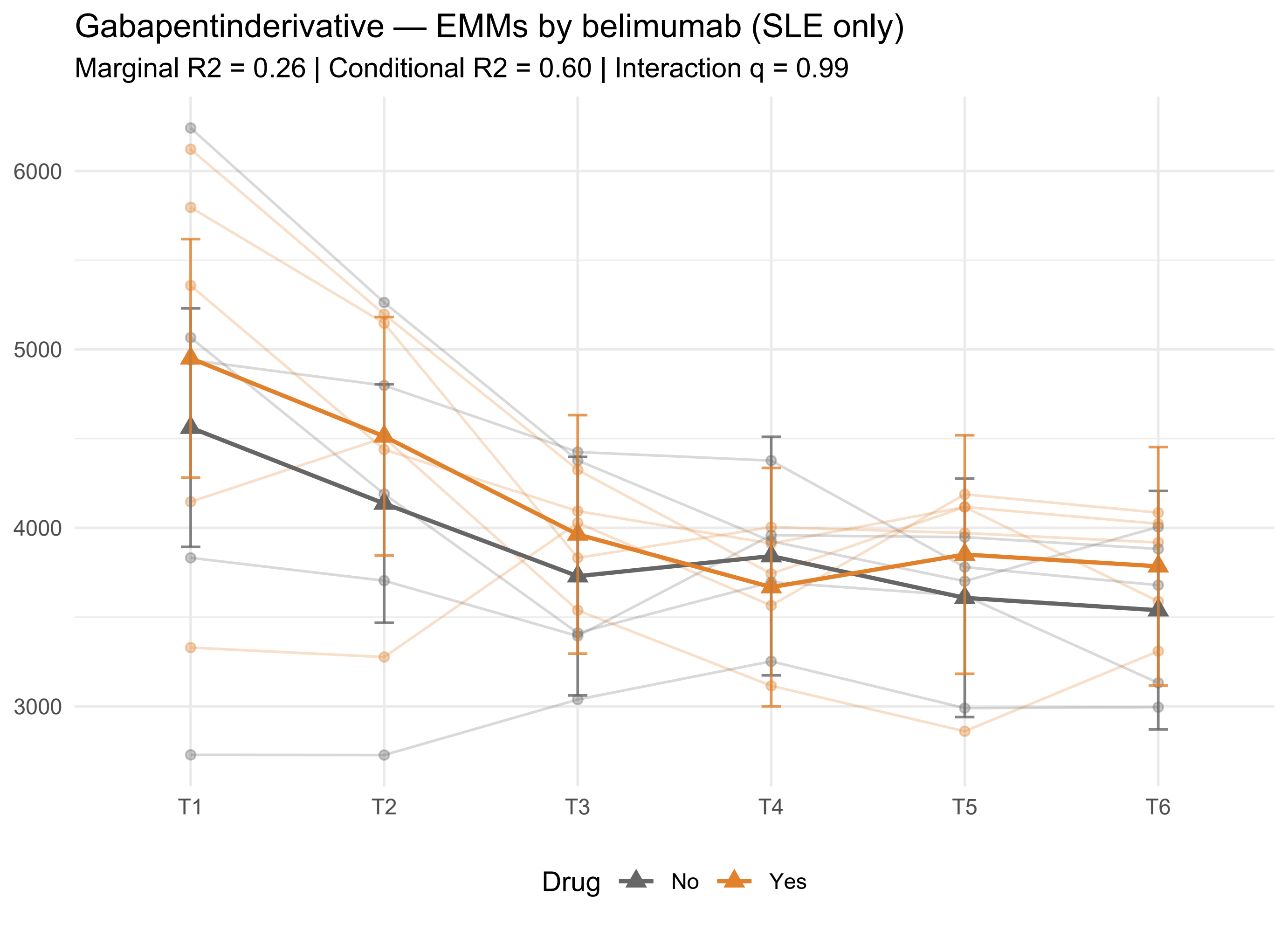
Gabapentinderivative — EMMs by belimumab (SLE only)
Marginal R2 = 0.26 | Conditional R2 = 0.60 | Interaction q = 0.99
6000
5000
4000
3000
T1
T2
T3
T4
T5
T6
Drug
No
Yes

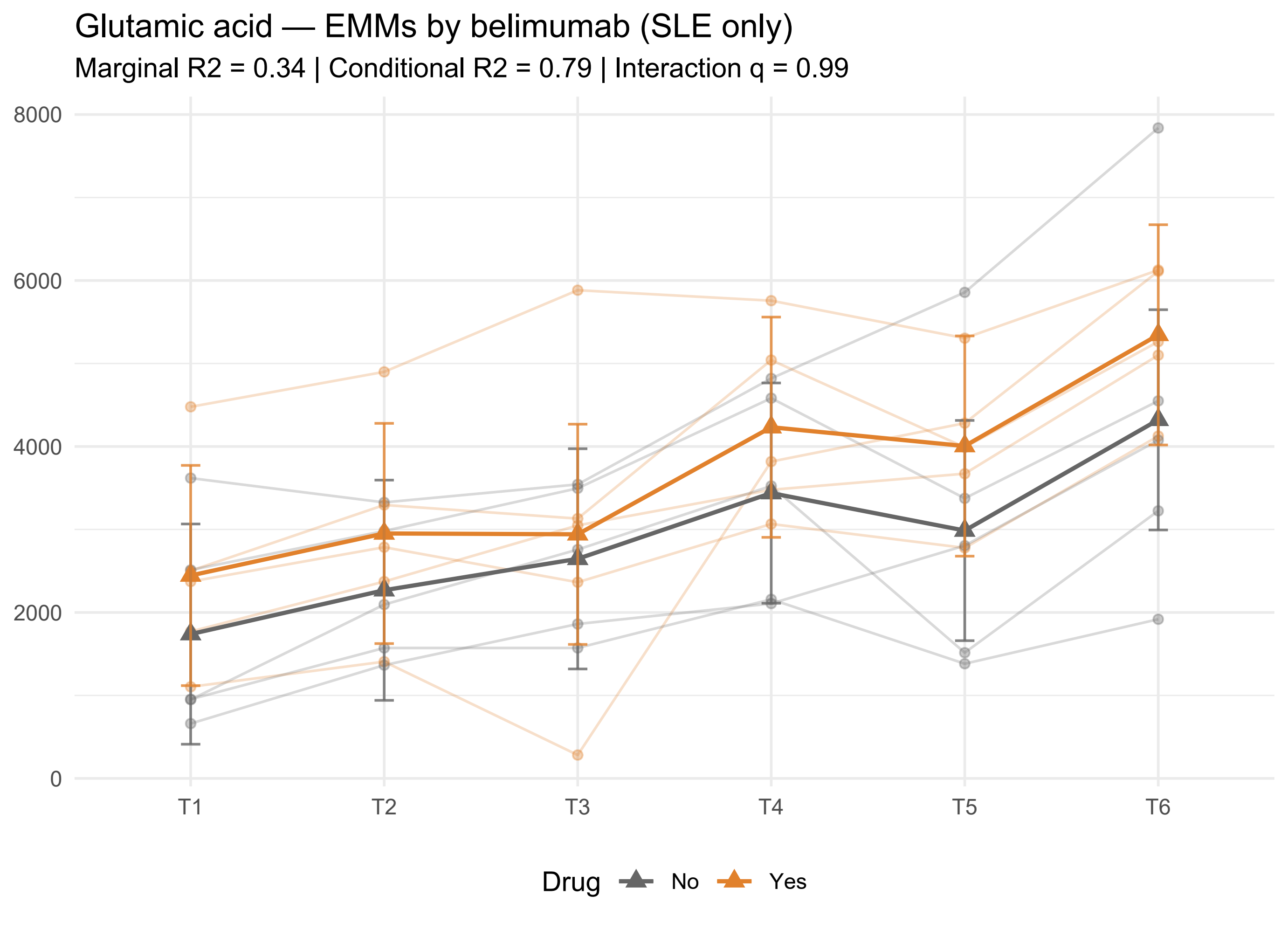
Glutamic acid — EMMs by belimumab (SLE only)
Marginal R2 = 0.34 | Conditional R2 = 0.79 | Interaction q = 0.99
8000
6000
4000
2000
0
T1
T2
T3
T4
T5
T6
Drug
No
Yes

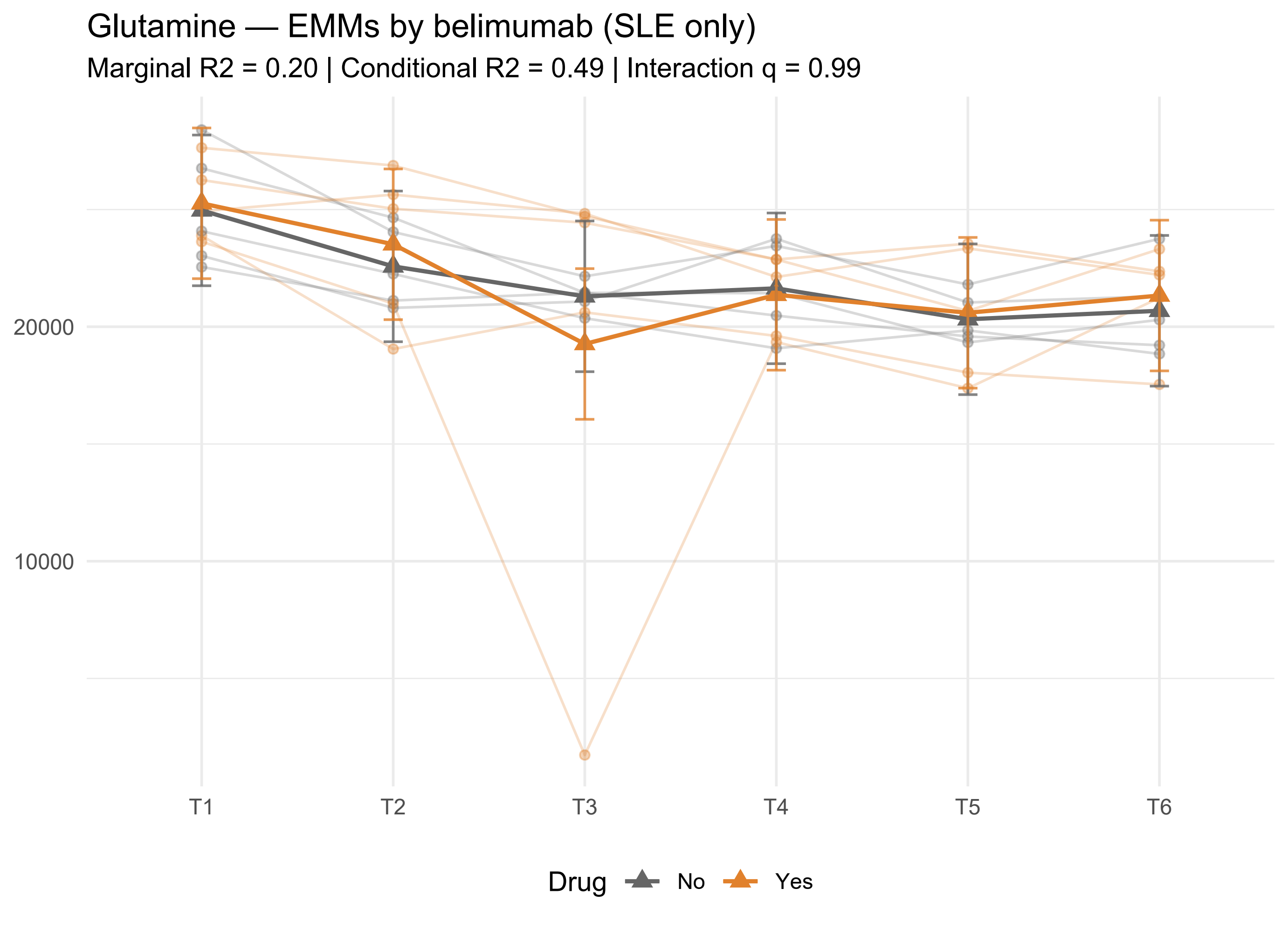

Glutamine — EMMs by belimumab (SLE only)
Marginal R2 = 0.20 | Conditional R2 = 0.49 | Interaction q = 0.99
20000
10000
T1
T2
T3
T4
T5
T6
Drug
No
Yes

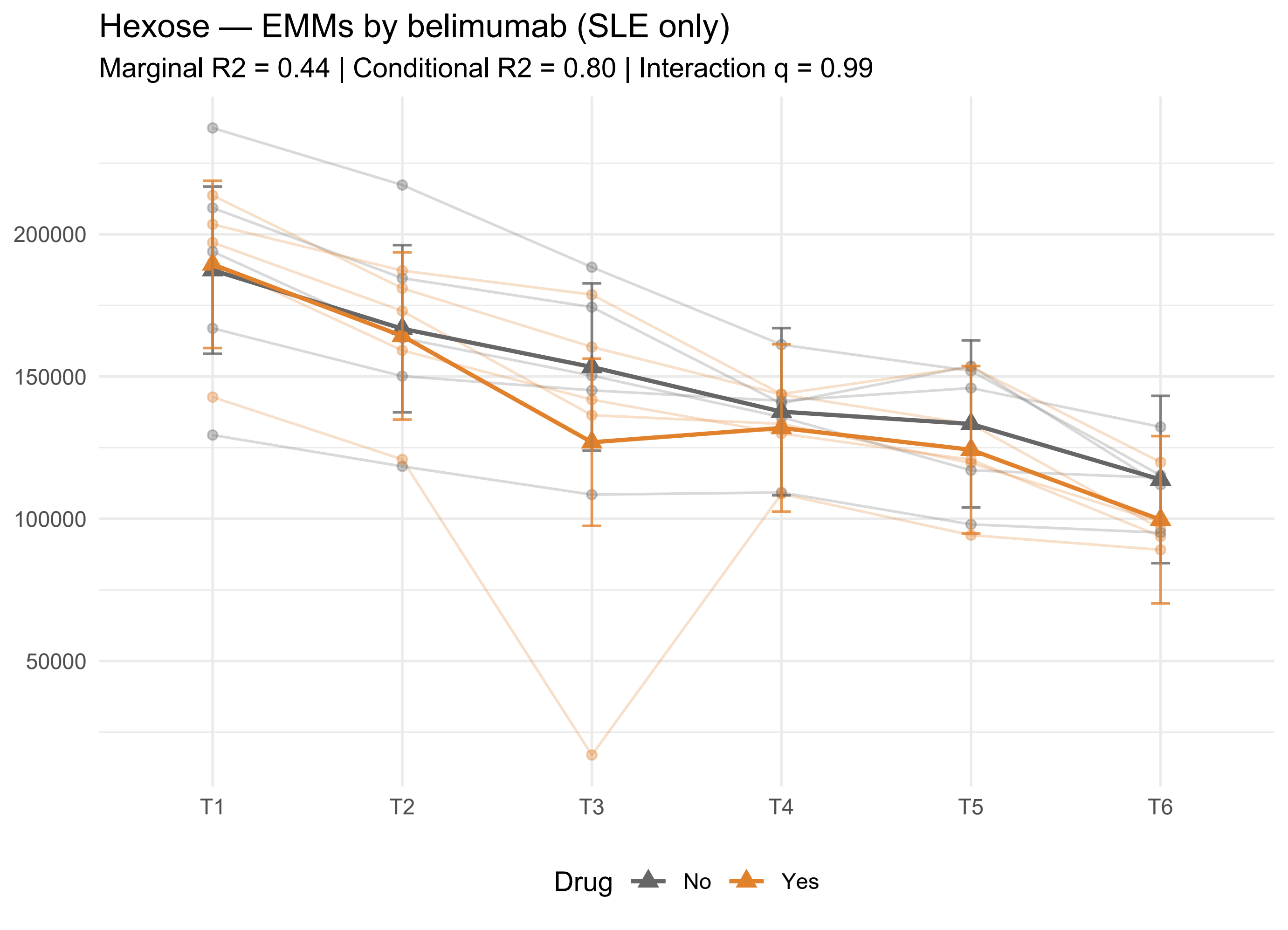

Hexose — EMMs by belimumab (SLE only)
Marginal R2 = 0.44 | Conditional R2 = 0.80 | Interaction q = 0.99
200000
150000
100000
50000
T1
T2
T3
T4
T5
T6
Drug
No
Yes

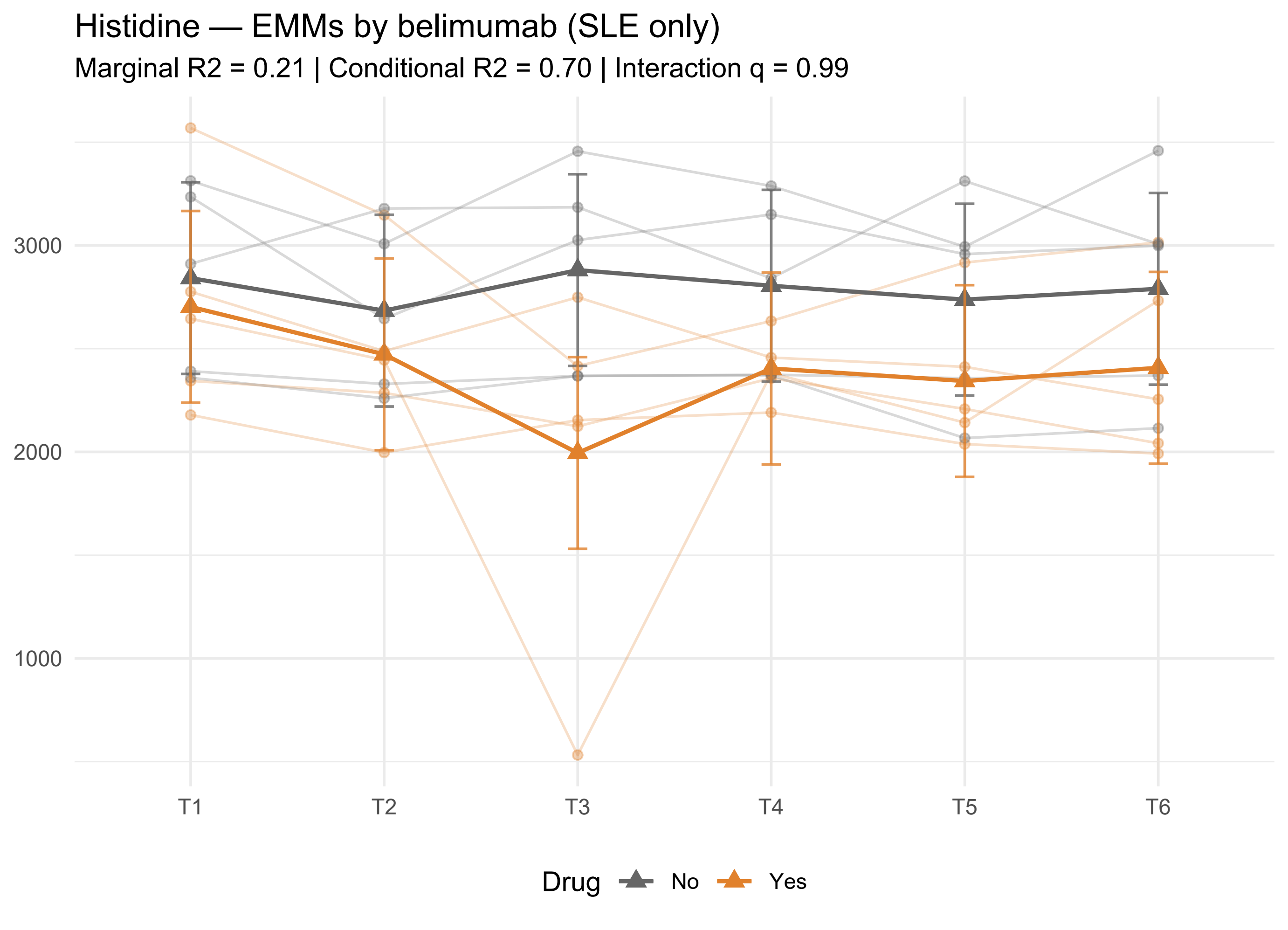

Histidine — EMMs by belimumab (SLE only)
Marginal R2 = 0.21 | Conditional R2 = 0.70 | Interaction q = 0.99
3000
2000
1000
T1
T2
T3
T4
T5
T6
Drug
No
Yes

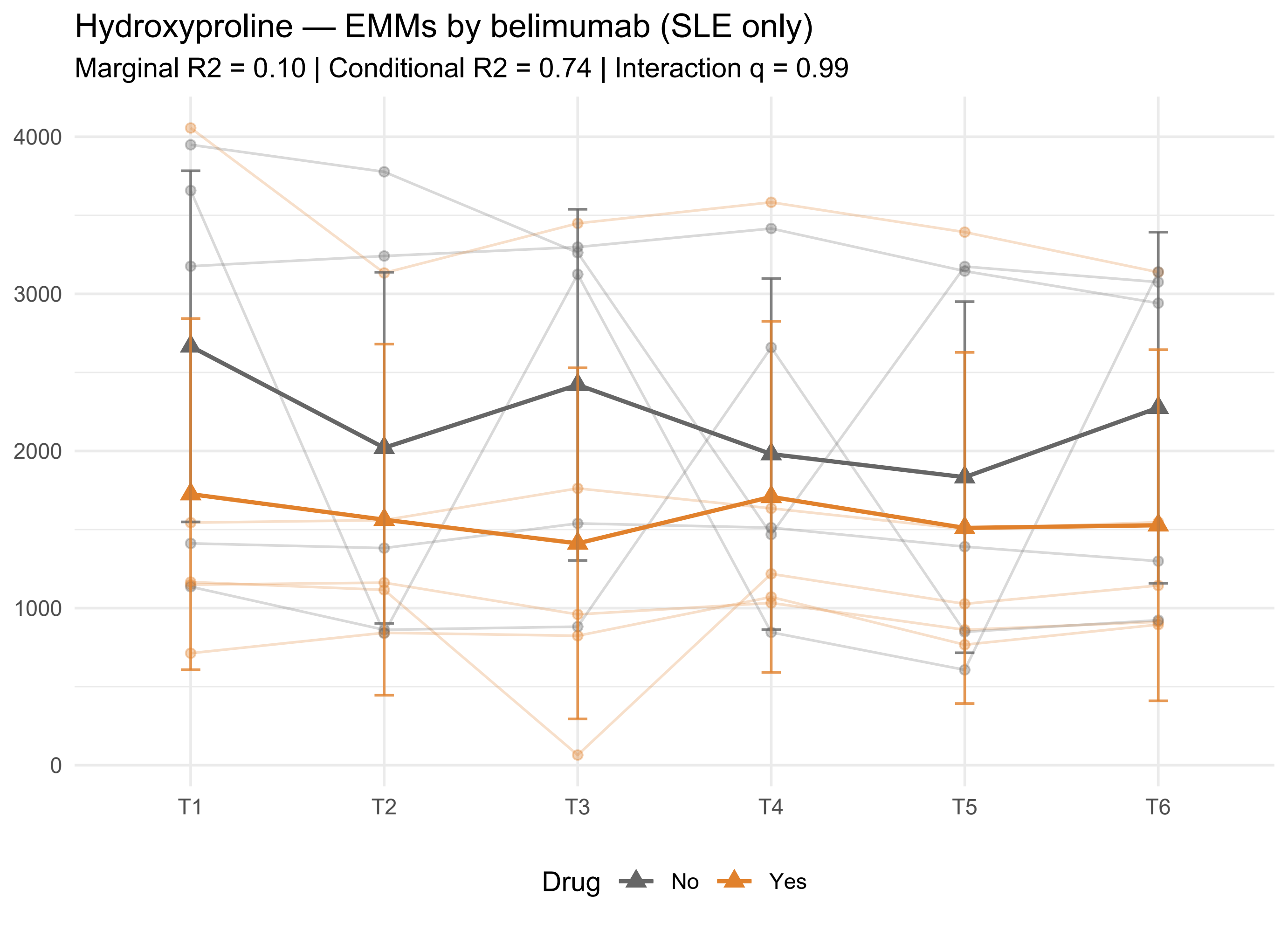
Hydroxyproline — EMMs by belimumab (SLE only)
Marginal R2 = 0.10 | Conditional R2 = 0.74 | Interaction q = 0.99
4000
3000
2000
1000
0
T1
T2
T3
T4
T5
T6
Drug
No
Yes

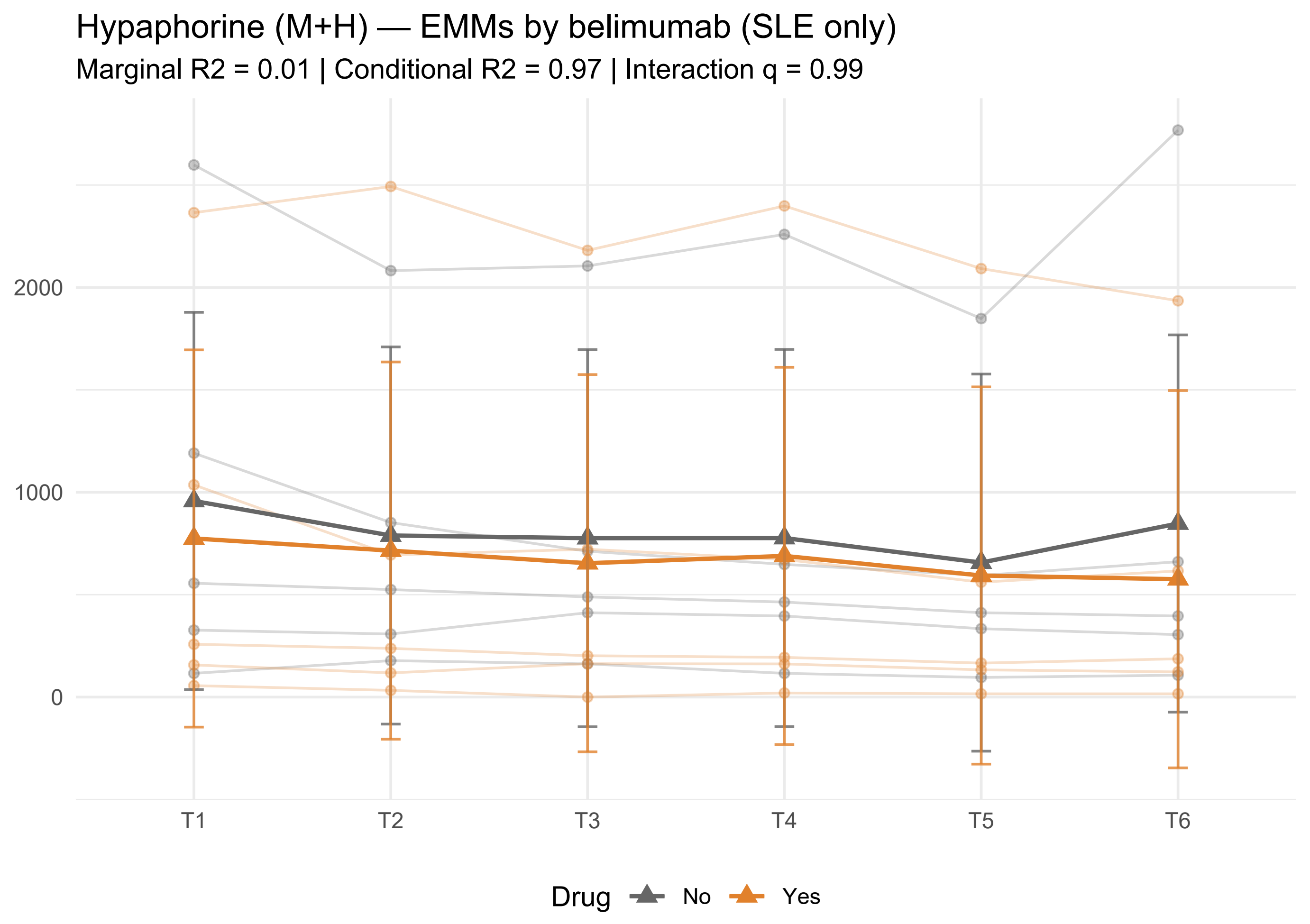

Hypaphorine (M+H) — EMMs by belimumab (SLE only)
Marginal R2 = 0.01 | Conditional R2 = 0.97 | Interaction q = 0.99
2000
1000
0
T1
T2
T3
T4
T5
T6
Drug
No
Yes

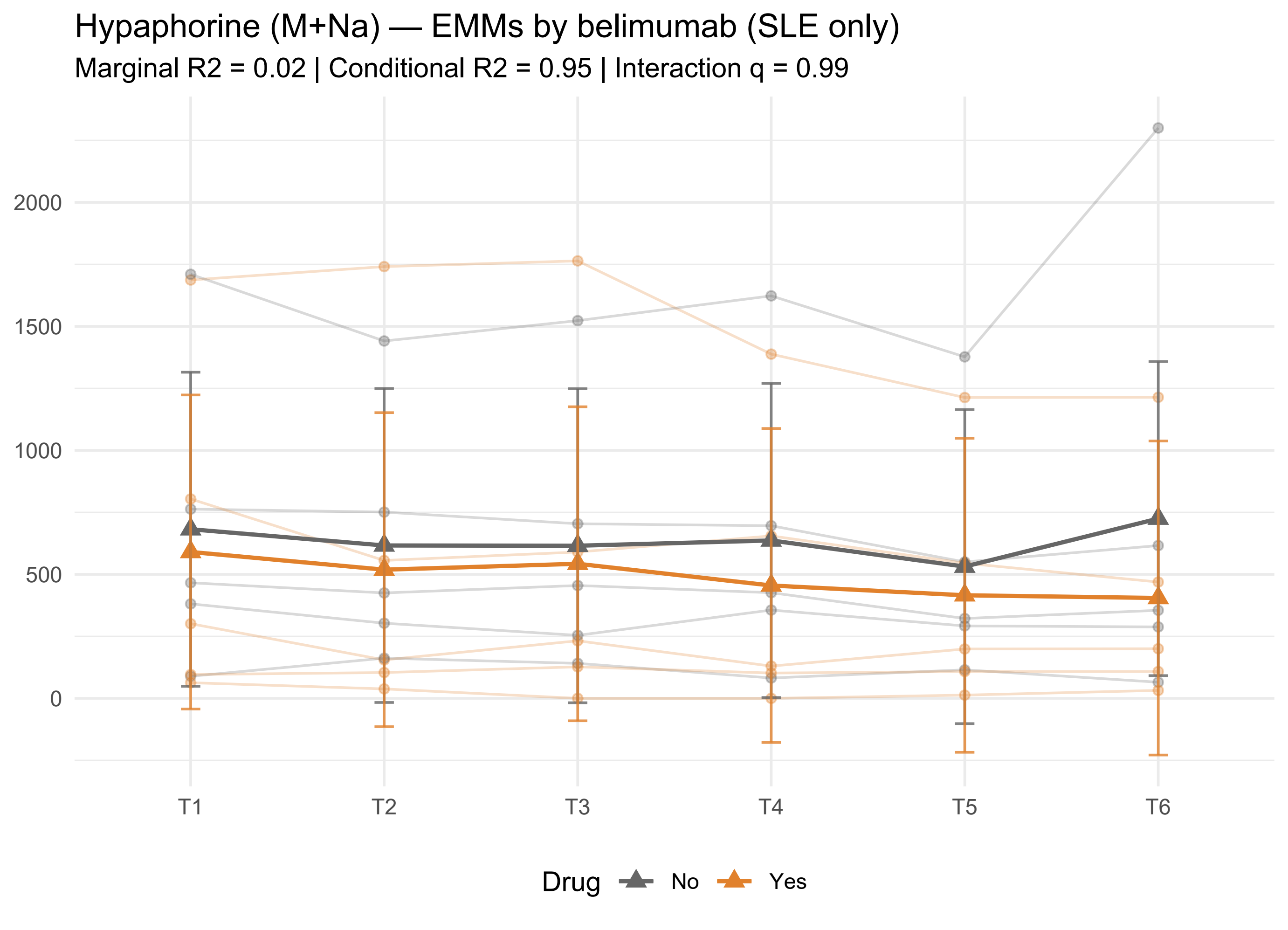
Hypaphorine (M+Na) — EMMs by belimumab (SLE only)
Marginal R2 = 0.02 | Conditional R2 = 0.95 | Interaction q = 0.99
2000
1500
1000
500
0
T1
T2
T3
T4
T5
T6
Drug
No
Yes

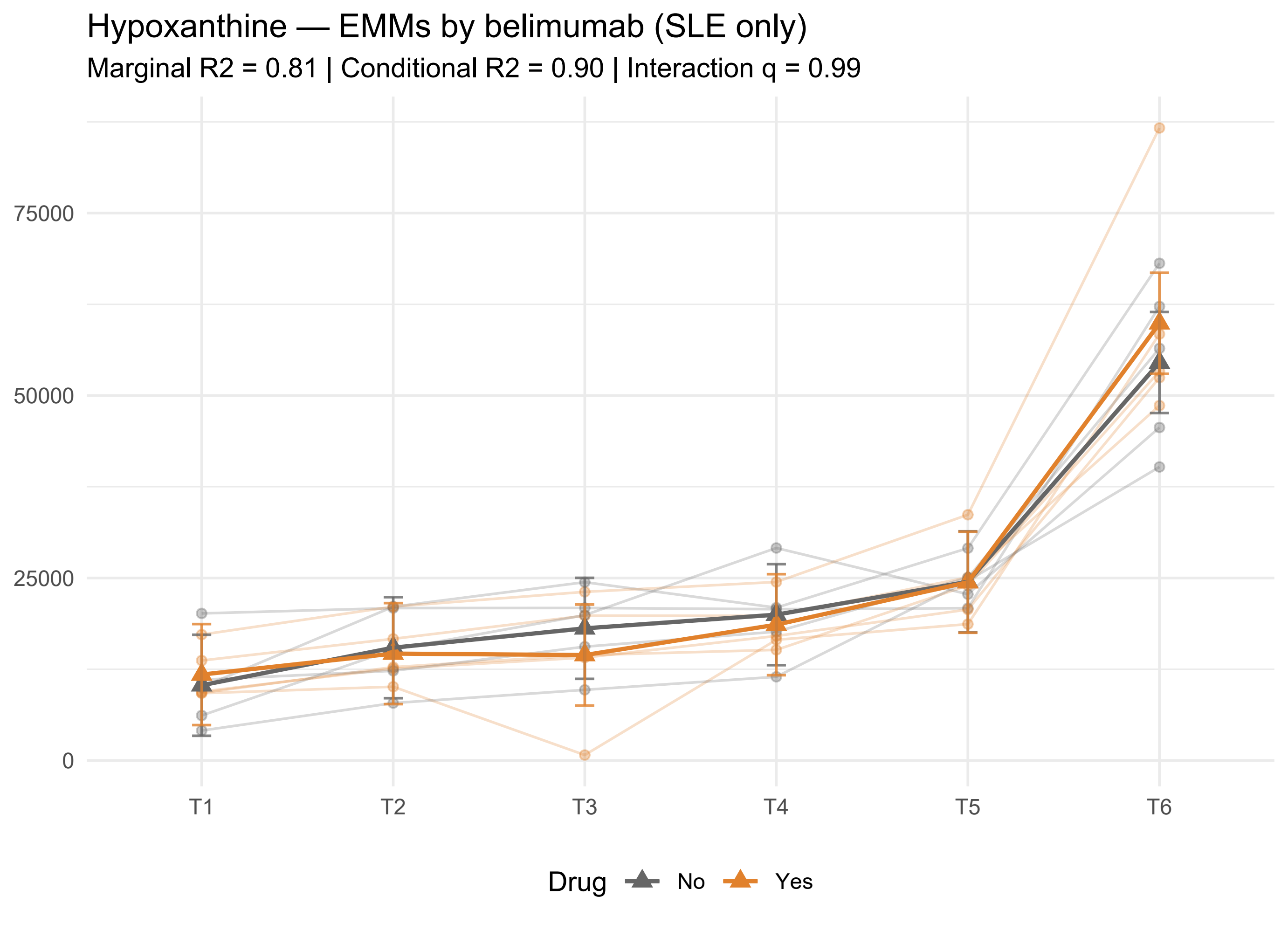

Hypoxanthine — EMMs by belimumab (SLE only)
Marginal R2 = 0.81 | Conditional R2 = 0.90 | Interaction q = 0.99
75000
50000
25000
0
T1
T2
T3
T4
T5
T6
Drug
No
Yes

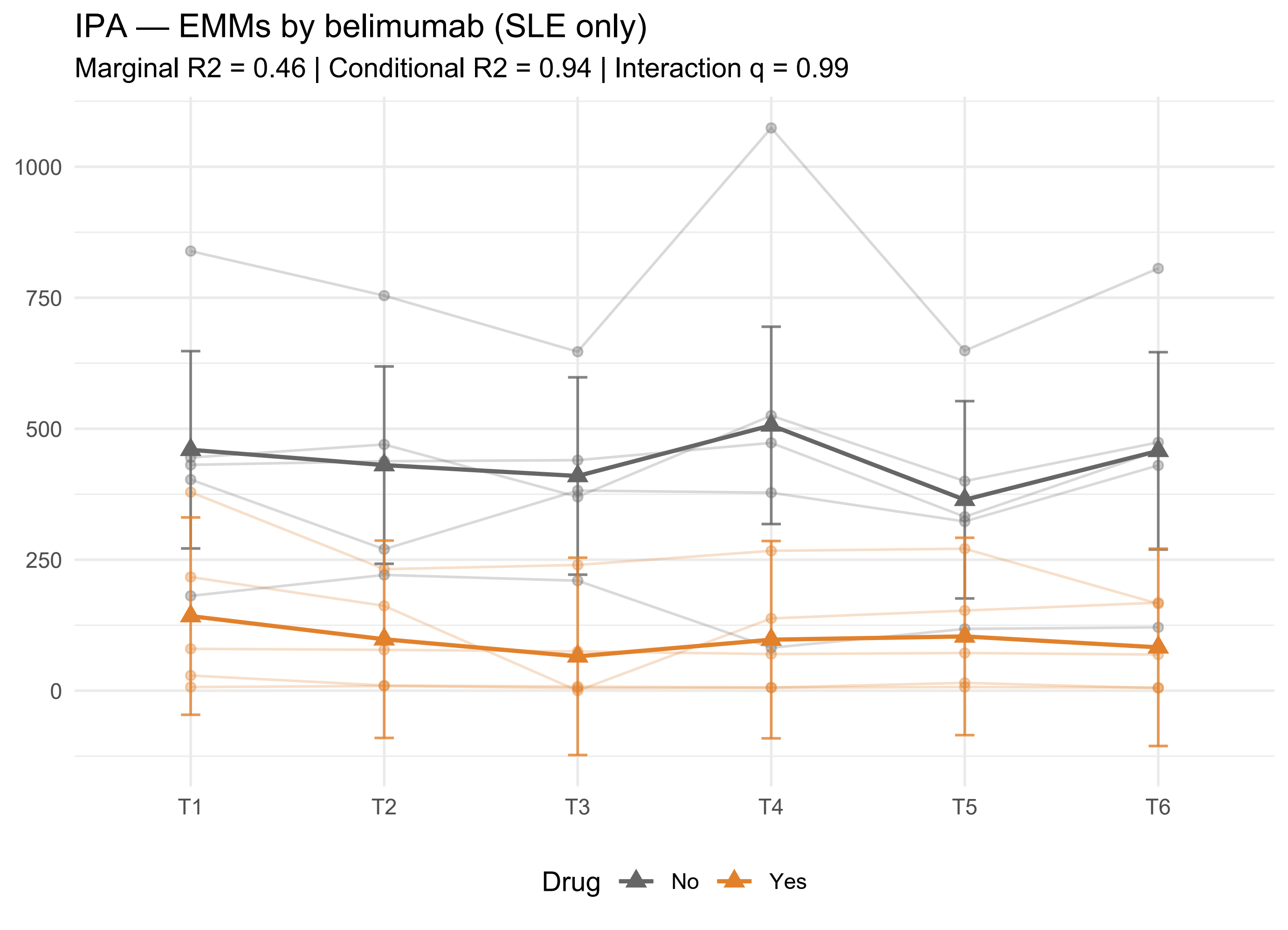

IPA — EMMs by belimumab (SLE only)
Marginal R2 = 0.46 | Conditional R2 = 0.94 | Interaction q = 0.99
1000
750
500
250
0
T1
T2
T3
T4
T5
T6
Drug
No
Yes

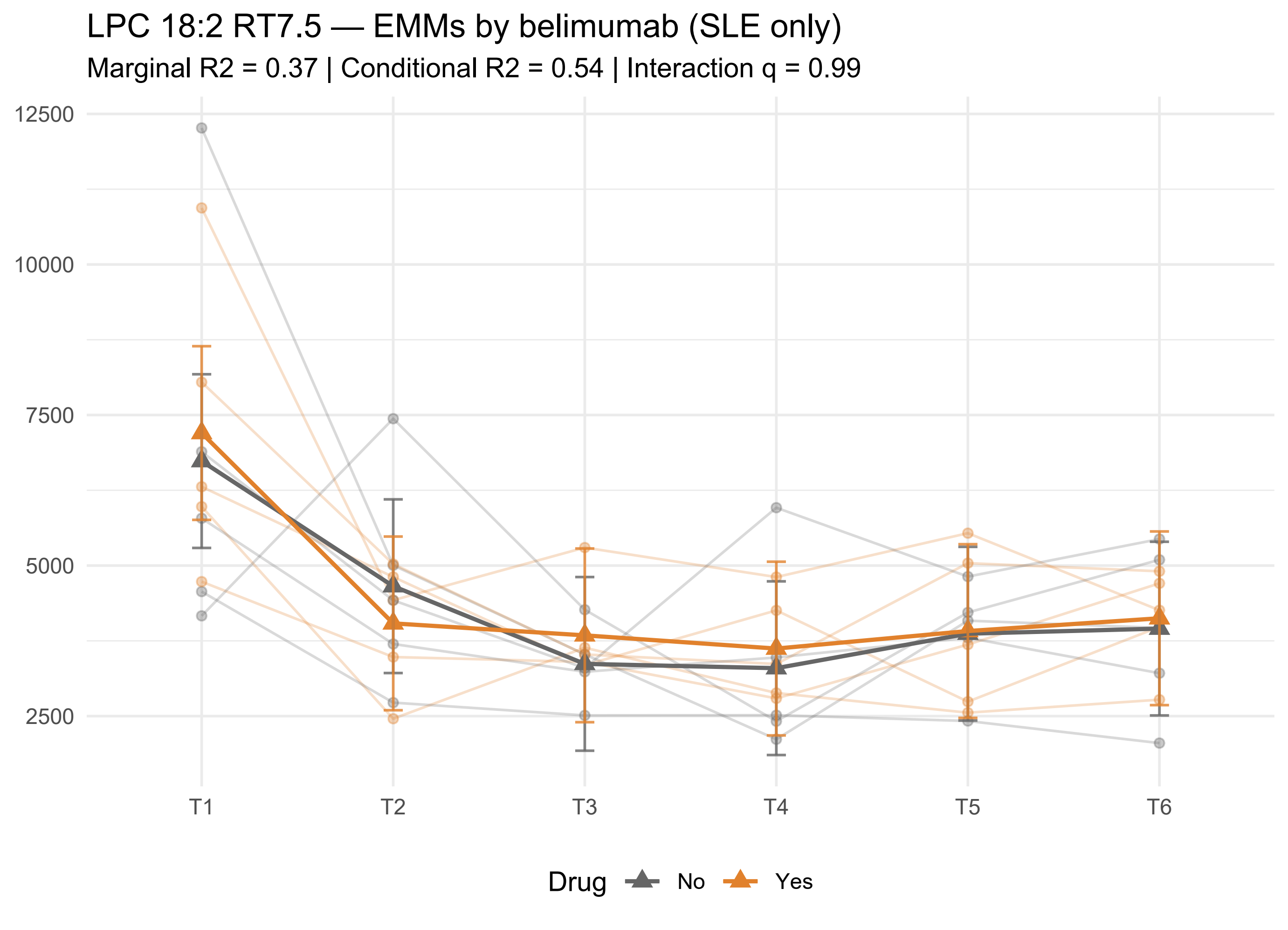

LPC 18:2 RT7.5 — EMMs by belimumab (SLE only)
Marginal R2 = 0.37 | Conditional R2 = 0.54 | Interaction q = 0.99
12500
10000
7500
5000
2500
T1
T2
T3
T4
T5
T6
Drug
No
Yes

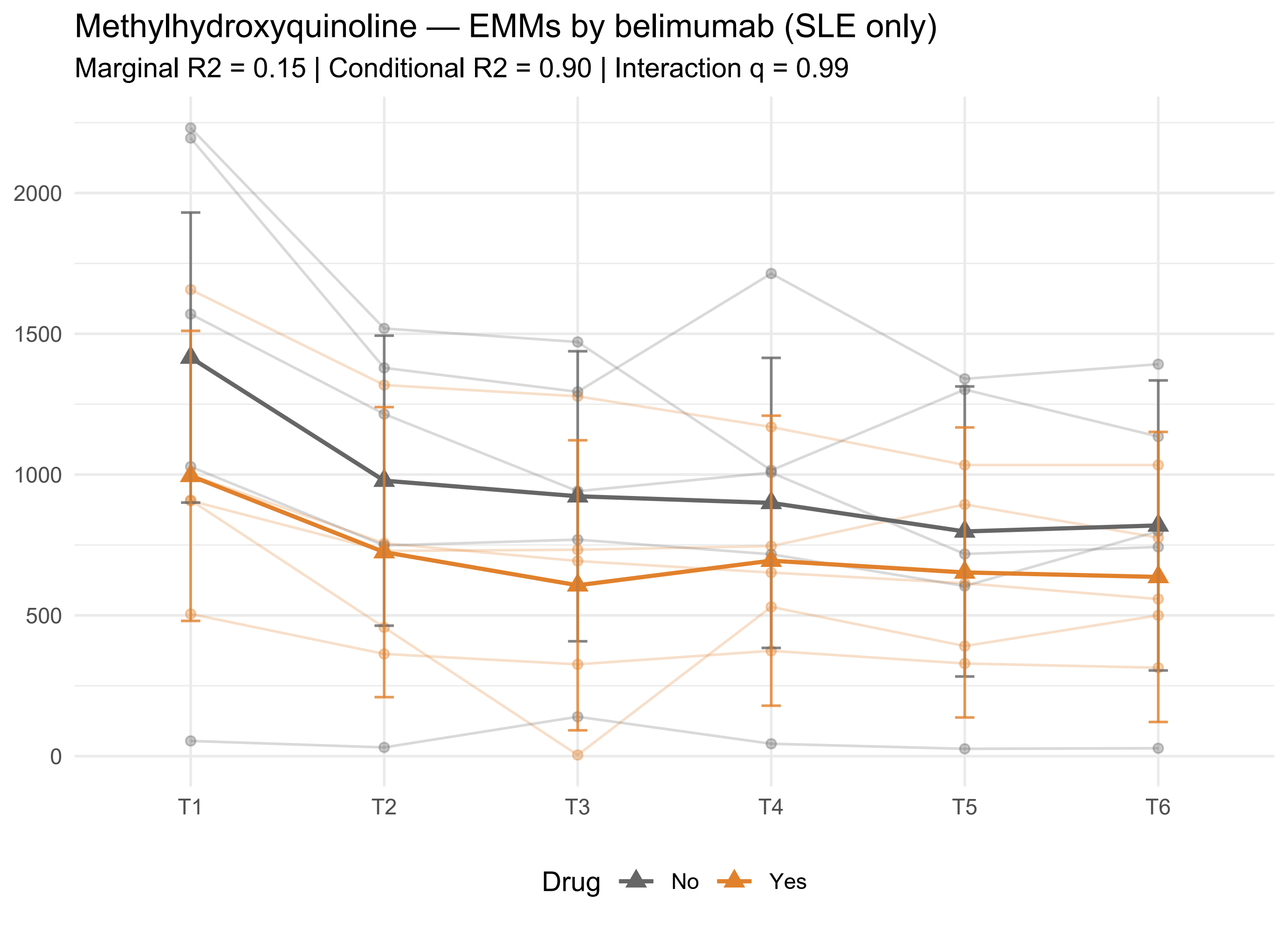

Methylhydroxyquinoline — EMMs by belimumab (SLE only)
Marginal R2 = 0.15 | Conditional R2 = 0.90 | Interaction q = 0.99
2000
1500
1000
500
0
T1
T2
T3
T4
T5
T6
Drug
No
Yes

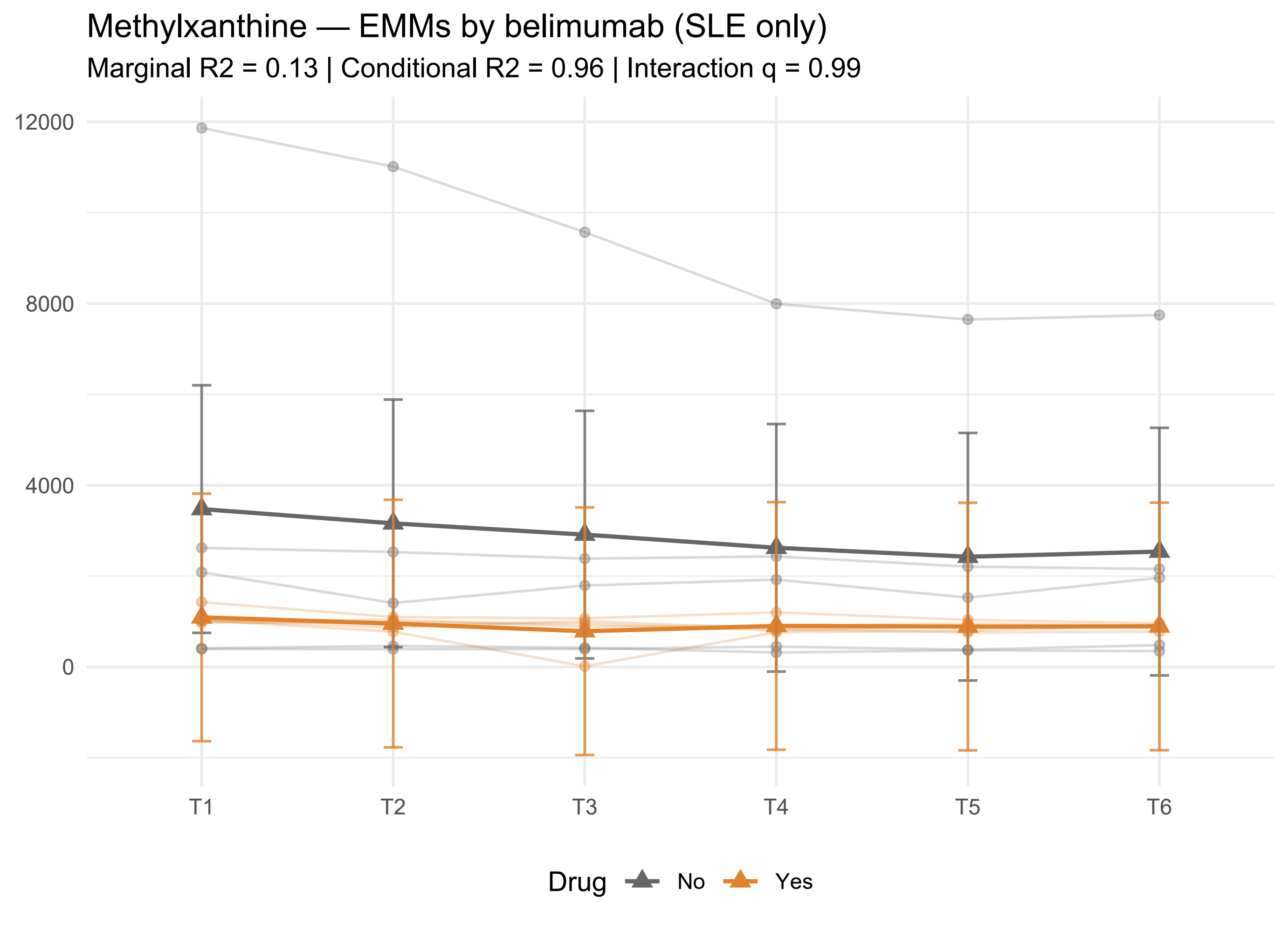

Methylxanthine — EMMs by belimumab (SLE only)
Marginal R2 = 0.13 | Conditional R2 = 0.96 | Interaction q = 0.99
12000
8000
4000
0
T1
T2
T3
T4
T5
T6
Drug
No
Yes

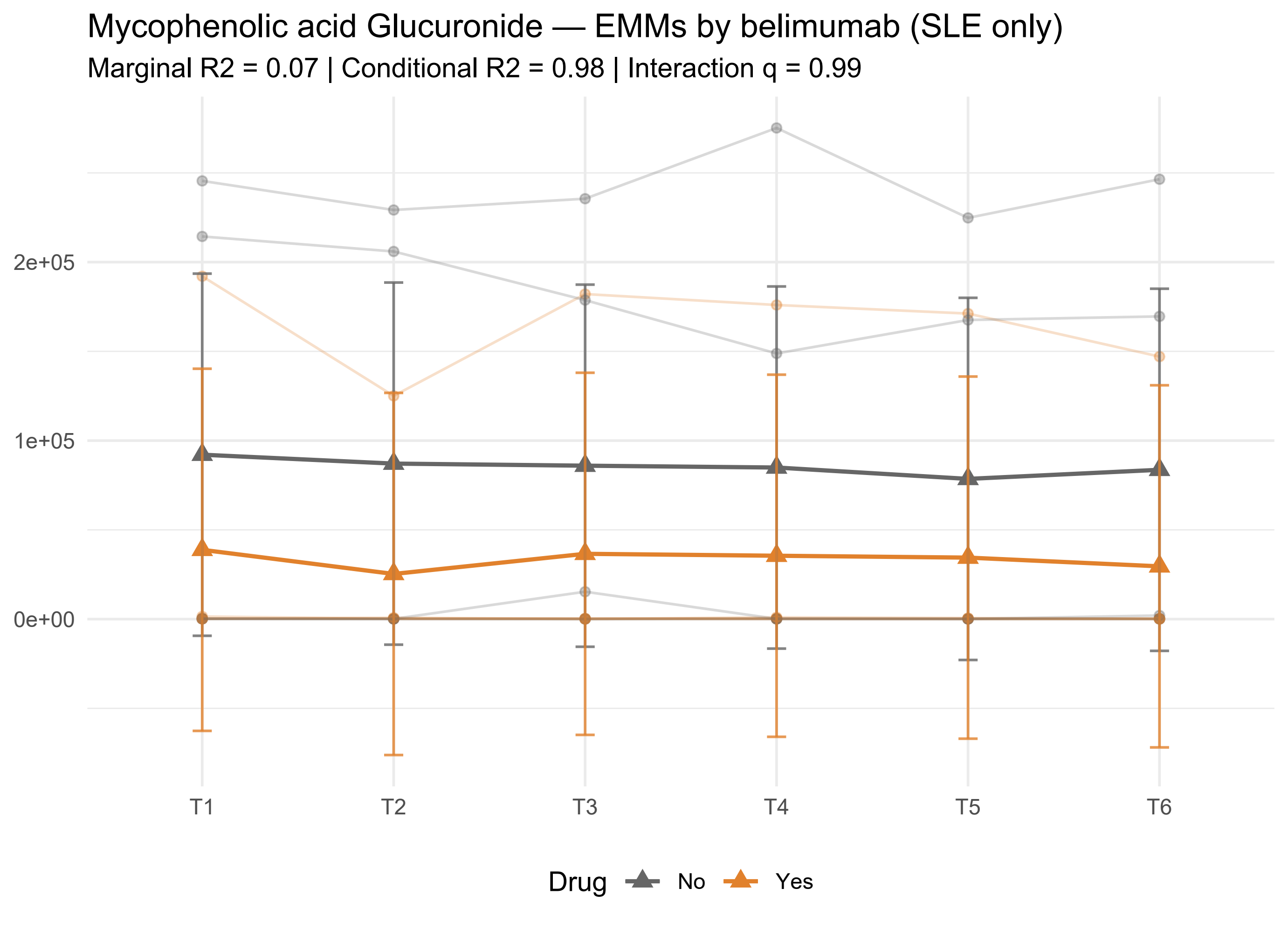

Mycophenolic acid Glucuronide — EMMs by belimumab (SLE only)
Marginal R2 = 0.07 | Conditional R2 = 0.98 | Interaction q = 0.99
2e+05
1e+05
0e+00
T1
T2
T3
T4
T5
T6
Drug
No
Yes

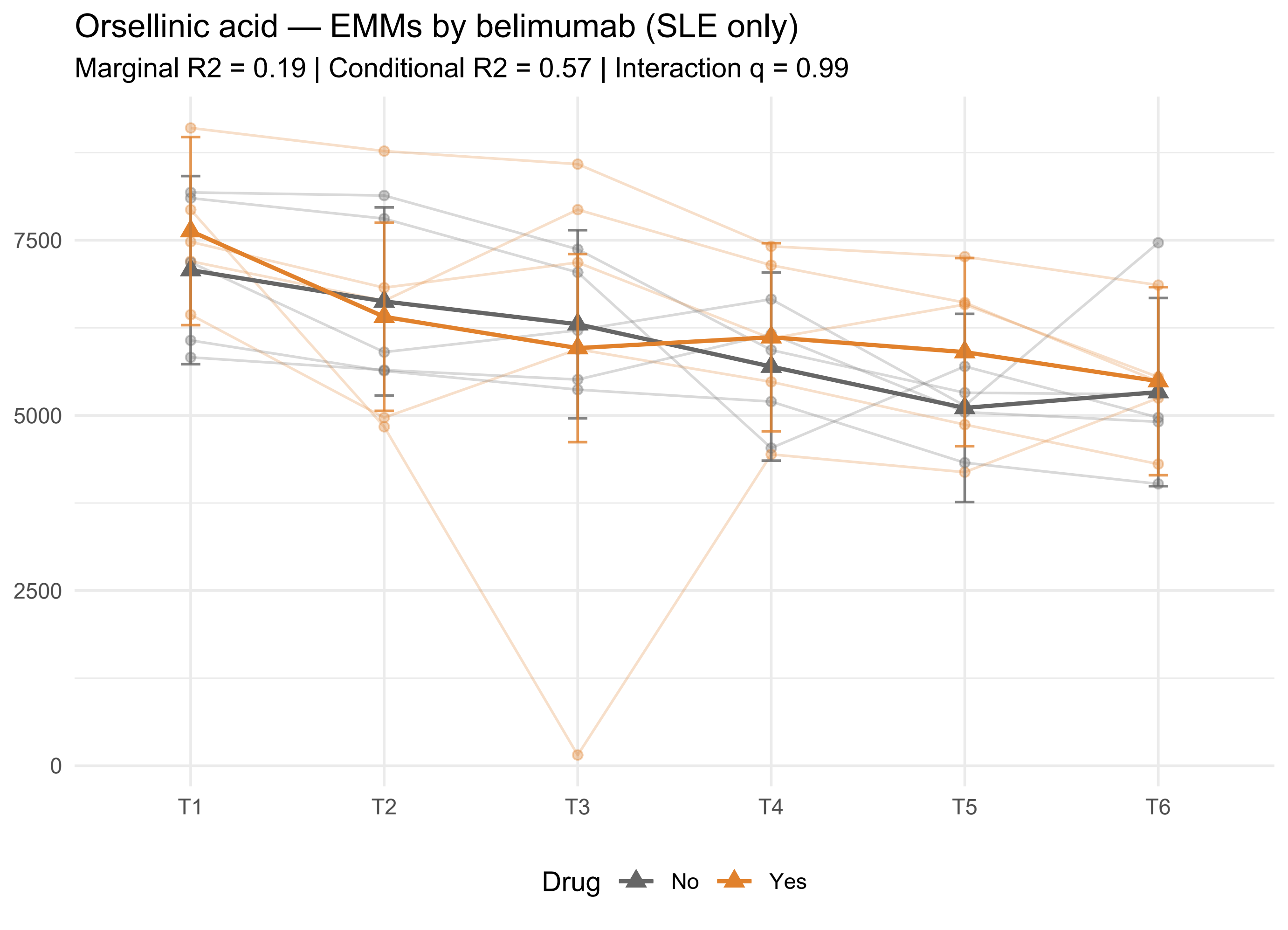

Orsellinic acid — EMMs by belimumab (SLE only)
Marginal R2 = 0.19 | Conditional R2 = 0.57 | Interaction q = 0.99
7500
5000
2500
0
T1
T2
T3
T4
T5
T6
Drug
No
Yes

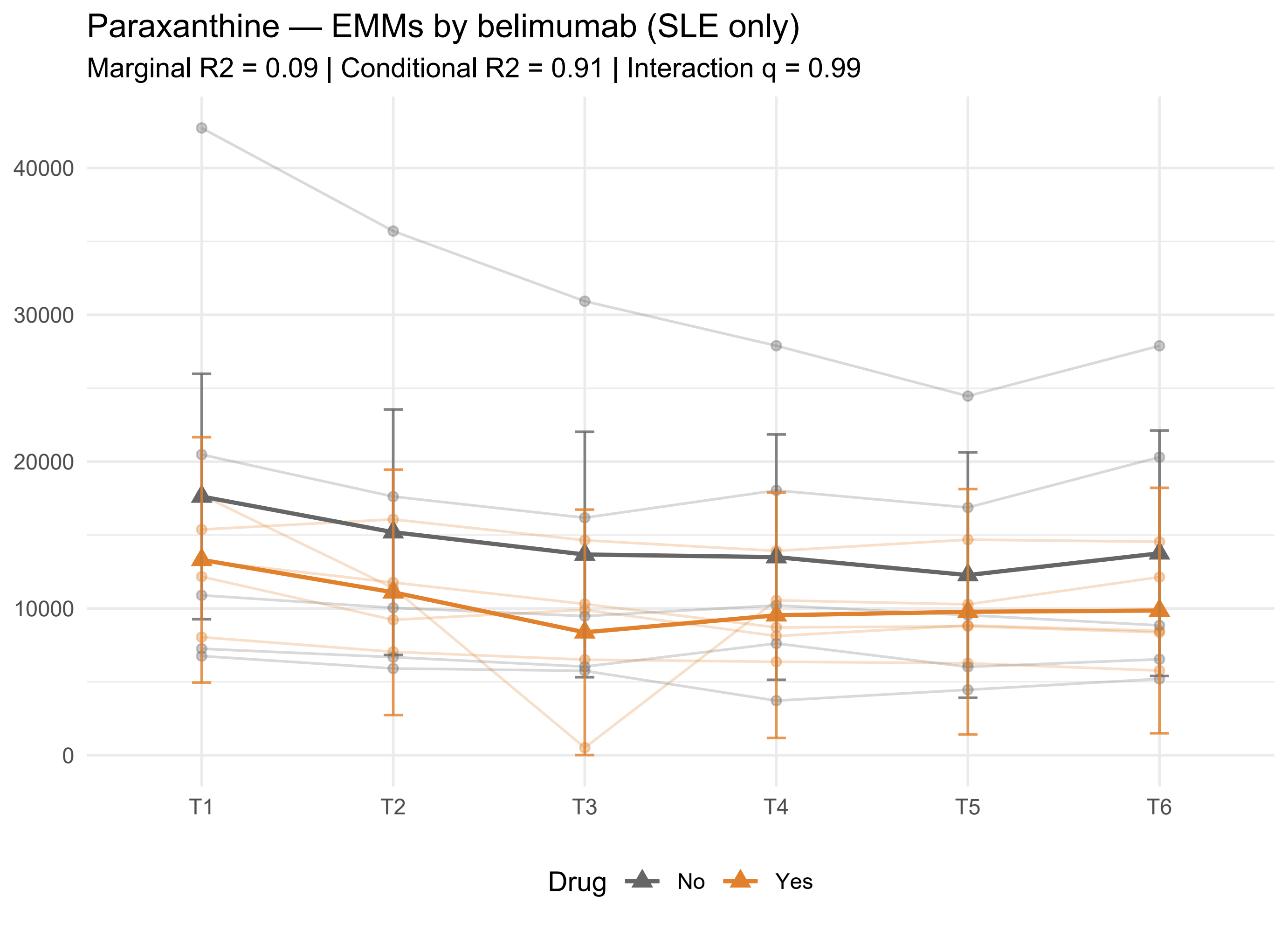

Paraxanthine — EMMs by belimumab (SLE only)
Marginal R2 = 0.09 | Conditional R2 = 0.91 | Interaction q = 0.99
40000
30000
20000
10000
0
T1
T2
T3
T4
T5
T6
Drug
No
Yes

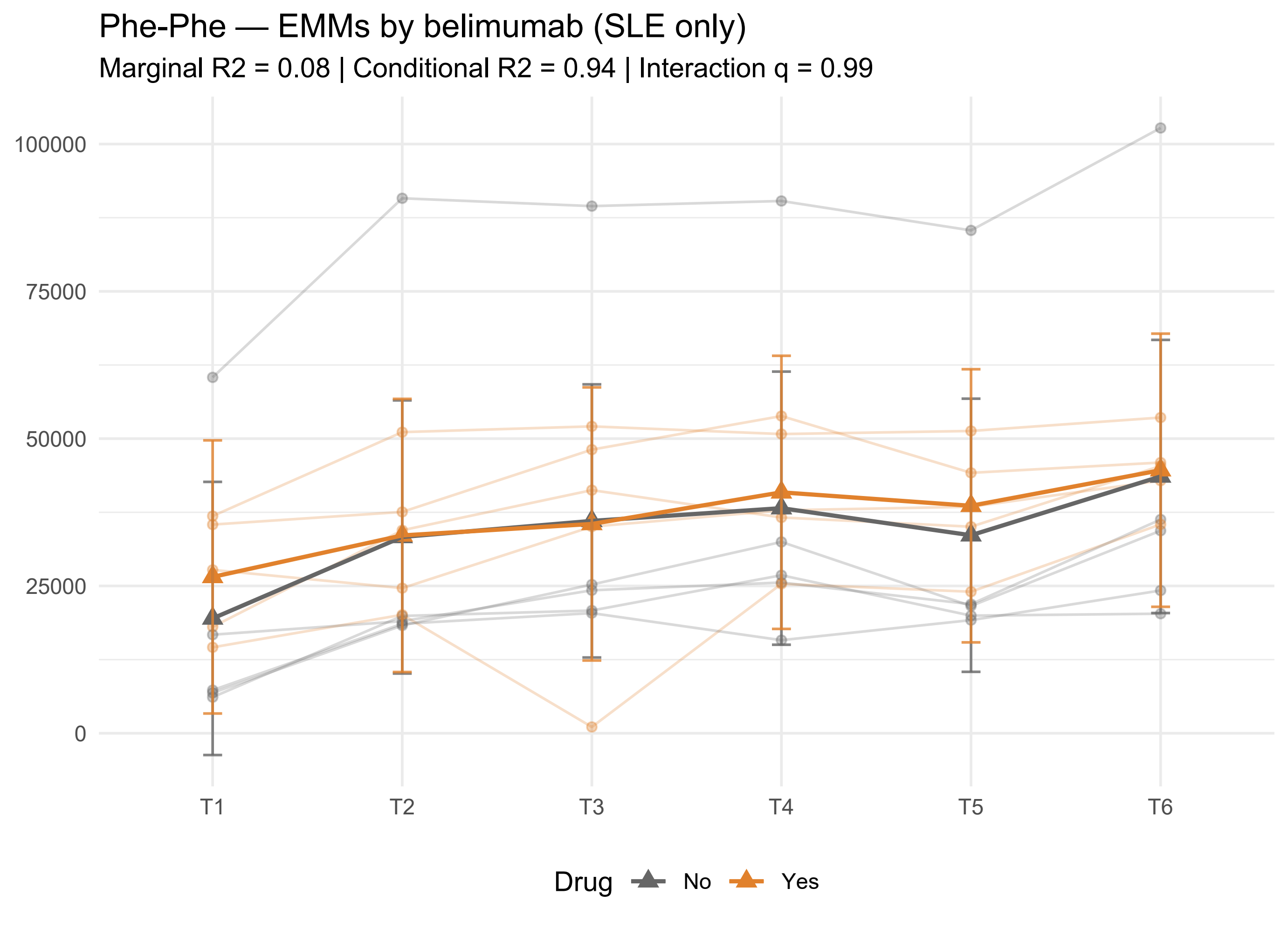

Phe-Phe — EMMs by belimumab (SLE only)
Marginal R2 = 0.08 | Conditional R2 = 0.94 | Interaction q = 0.99
100000
75000
50000
25000
0
T1
T2
T3
T4
T5
T6
Drug
No
Yes

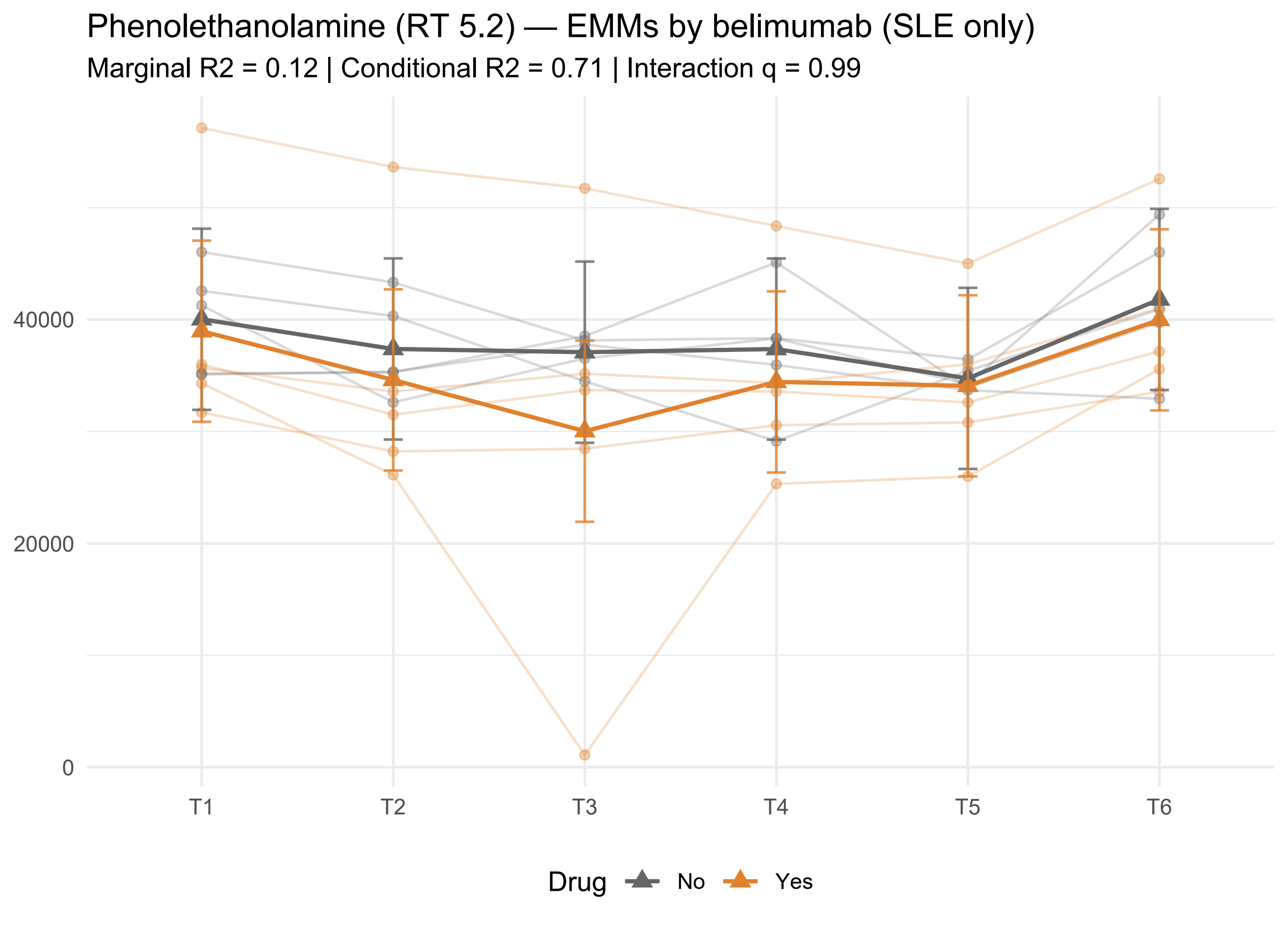

Phenolethanolamine (RT 5.2) — EMMs by belimumab (SLE only)
Marginal R2 = 0.12 | Conditional R2 = 0.71 | Interaction q = 0.99
40000
20000
0
T1
T2
T3
T4
T5
T6
Drug
No
Yes

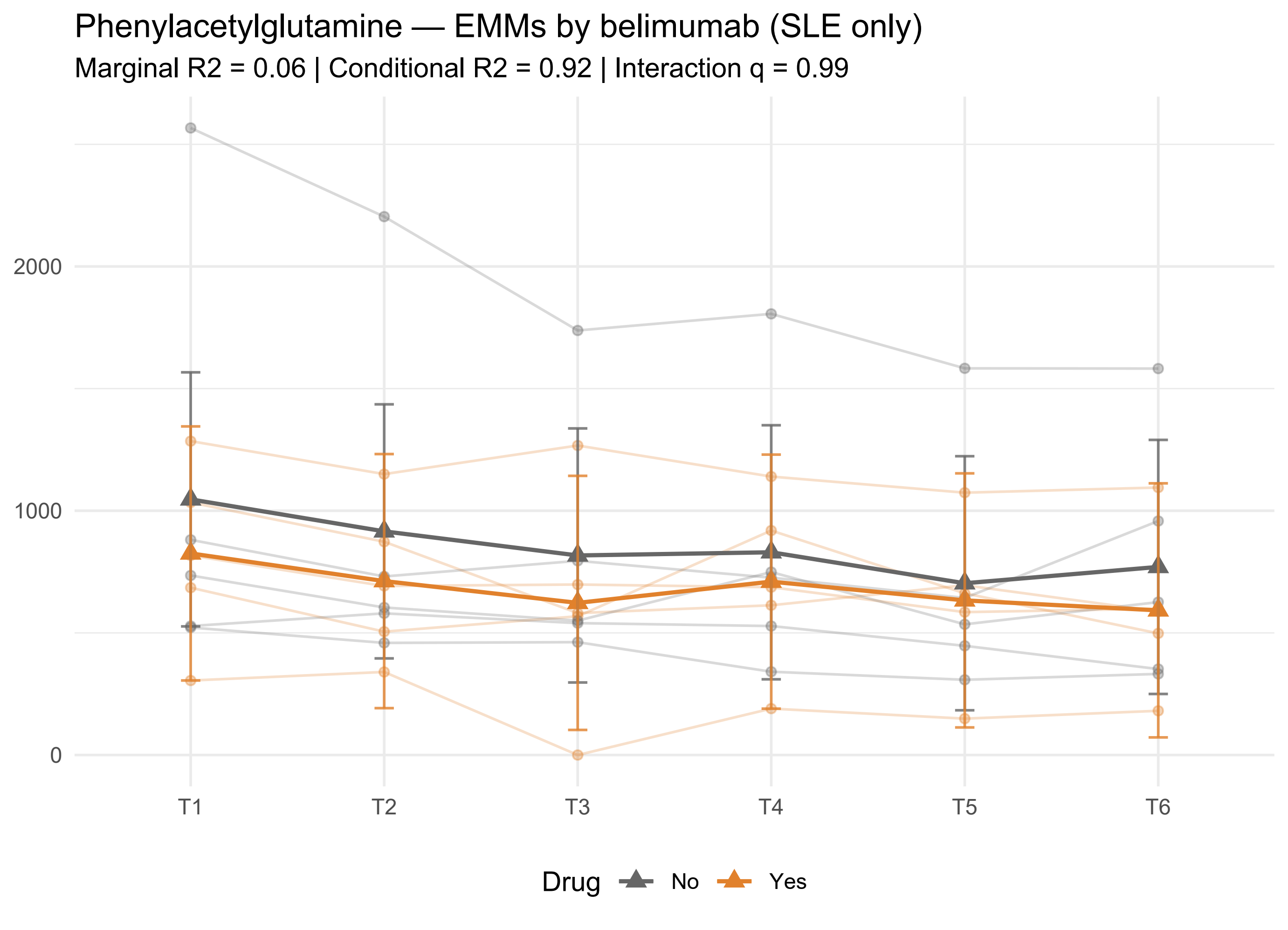
Phenylacetylglutamine — EMMs by belimumab (SLE only)
Marginal R2 = 0.06 | Conditional R2 = 0.92 | Interaction q = 0.99
2000
1000
0
T1
T2
T3
T4
T5
T6
Drug
No
Yes

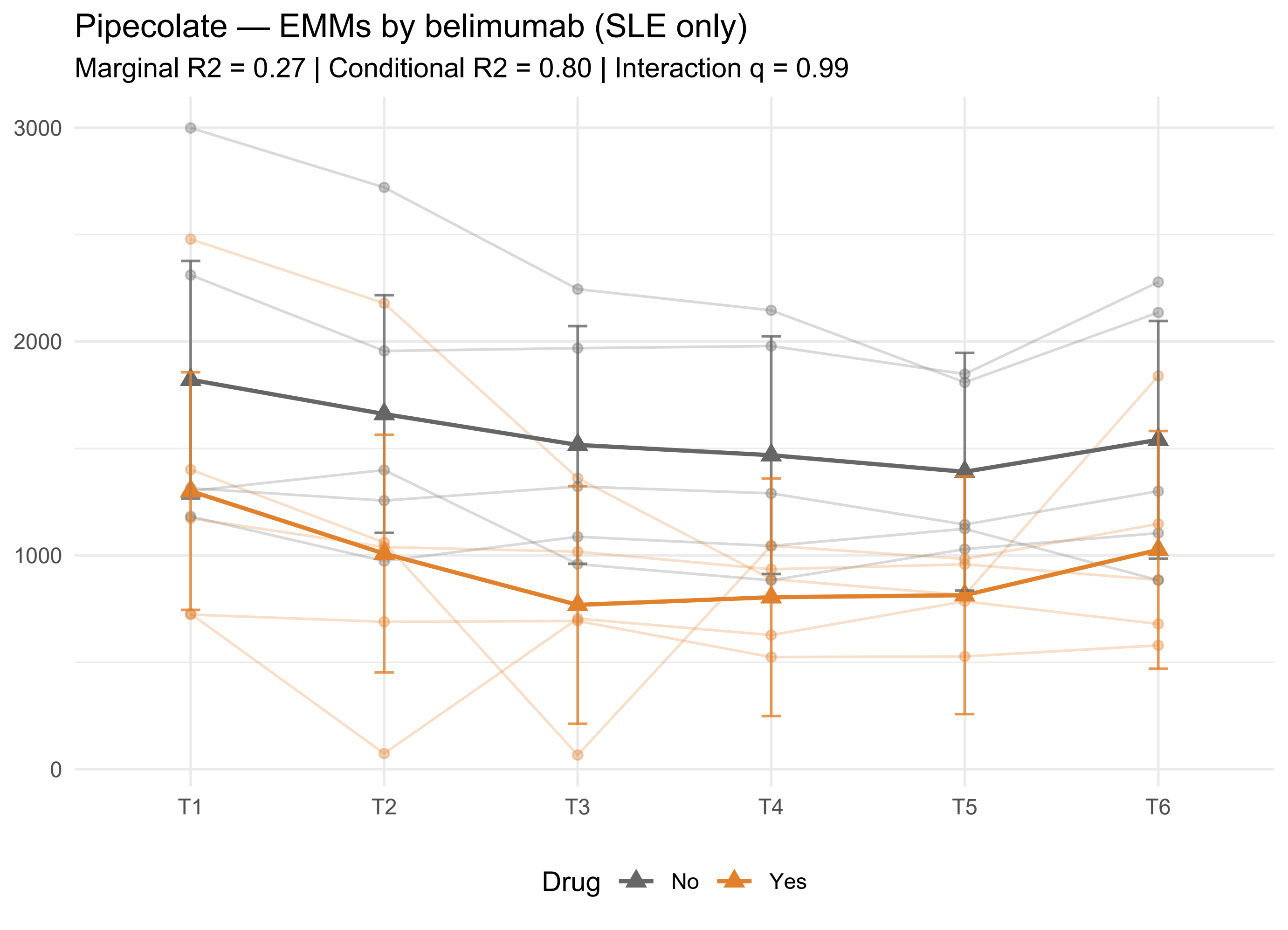

Pipecolate — EMMs by belimumab (SLE only)
Marginal R2 = 0.27 | Conditional R2 = 0.80 | Interaction q = 0.99
3000
2000
1000
0
T1
T2
T3
T4
T5
T6
Drug
No
Yes

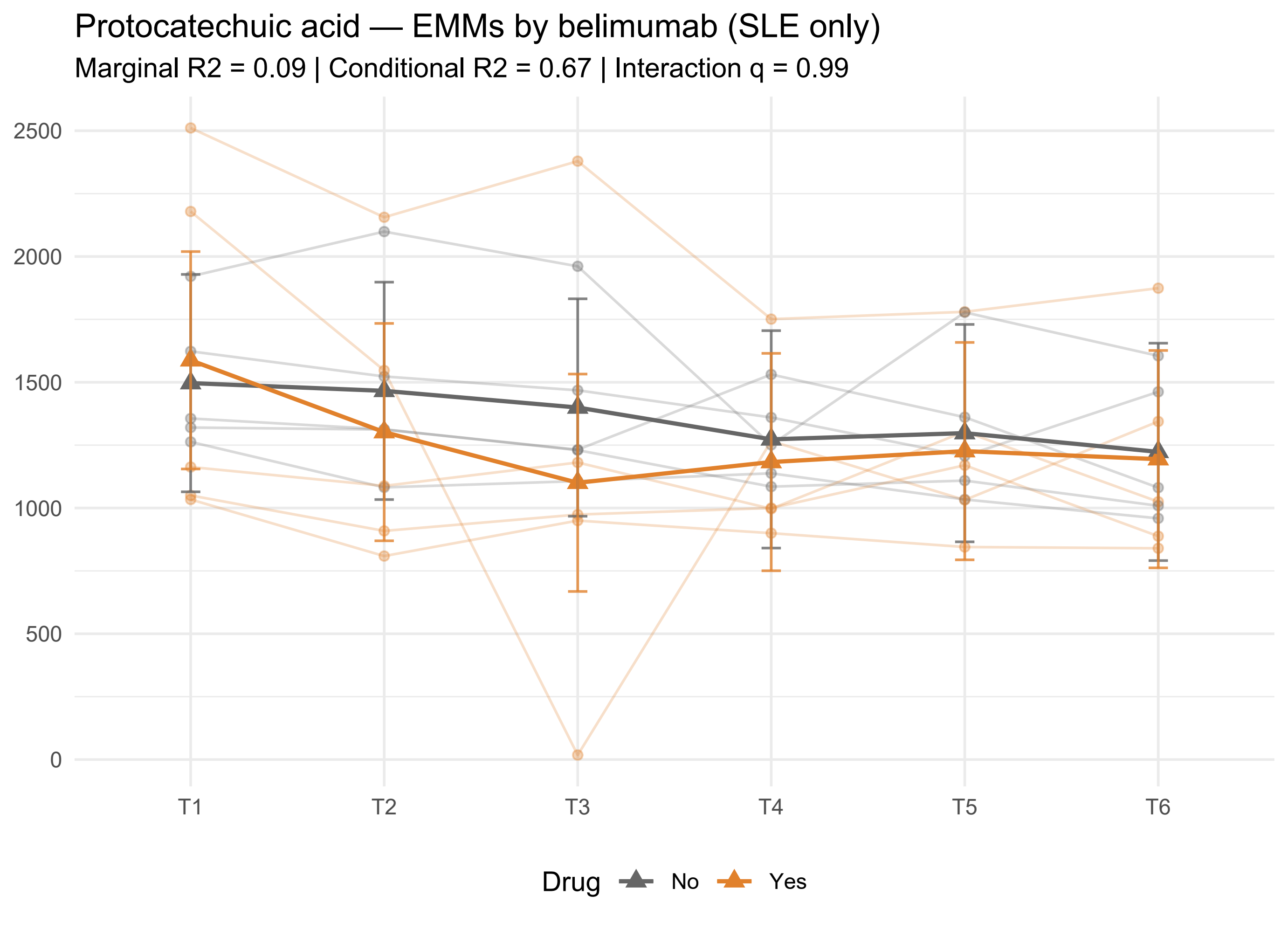
Protocatechuic acid — EMMs by belimumab (SLE only)
Marginal R2 = 0.09 | Conditional R2 = 0.67 | Interaction q = 0.99
2500
2000
1500
1000
500
0
T1
T2
T3
T4
T5
T6
Drug
No
Yes

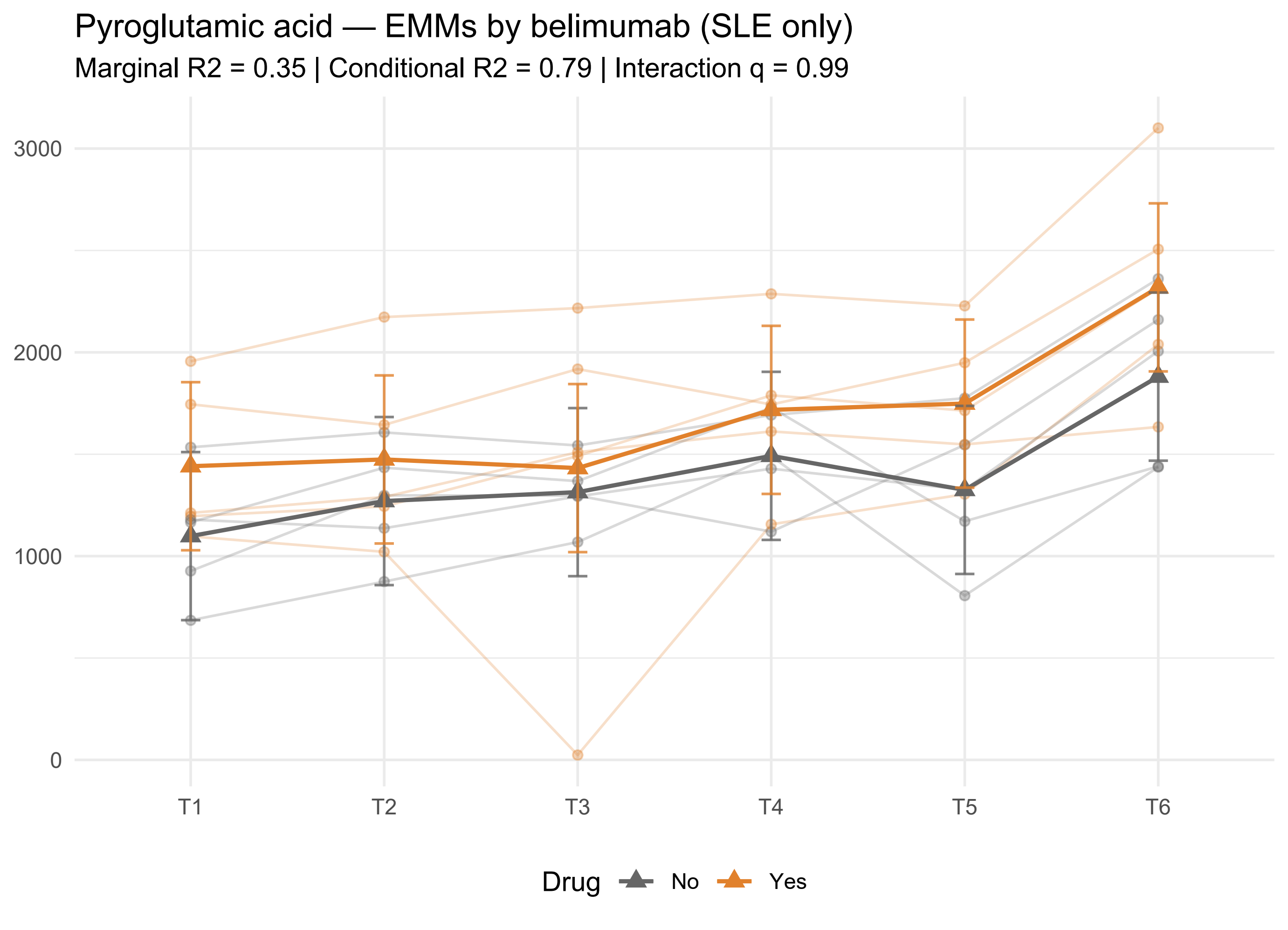
Pyroglutamic acid — EMMs by belimumab (SLE only)
Marginal R2 = 0.35 | Conditional R2 = 0.79 | Interaction q = 0.99
3000
2000
1000
0
T1
T2
T3
T4
T5
T6
Drug
No
Yes

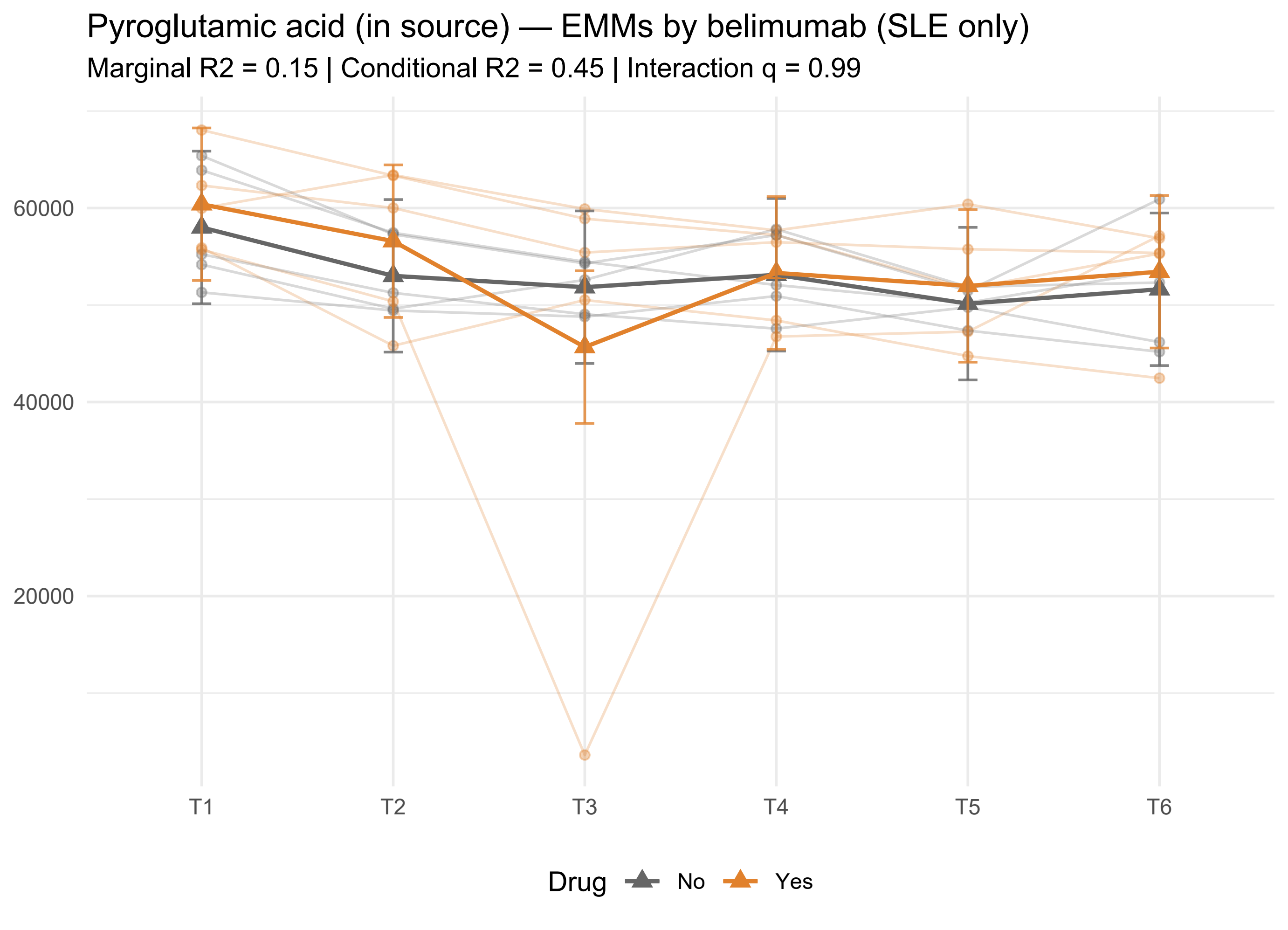
Pyroglutamic acid (in source) — EMMs by belimumab (SLE only)
Marginal R2 = 0.15 | Conditional R2 = 0.45 | Interaction q = 0.99
60000
40000
20000
T1
T2
T3
T4
T5
T6
Drug
No
Yes

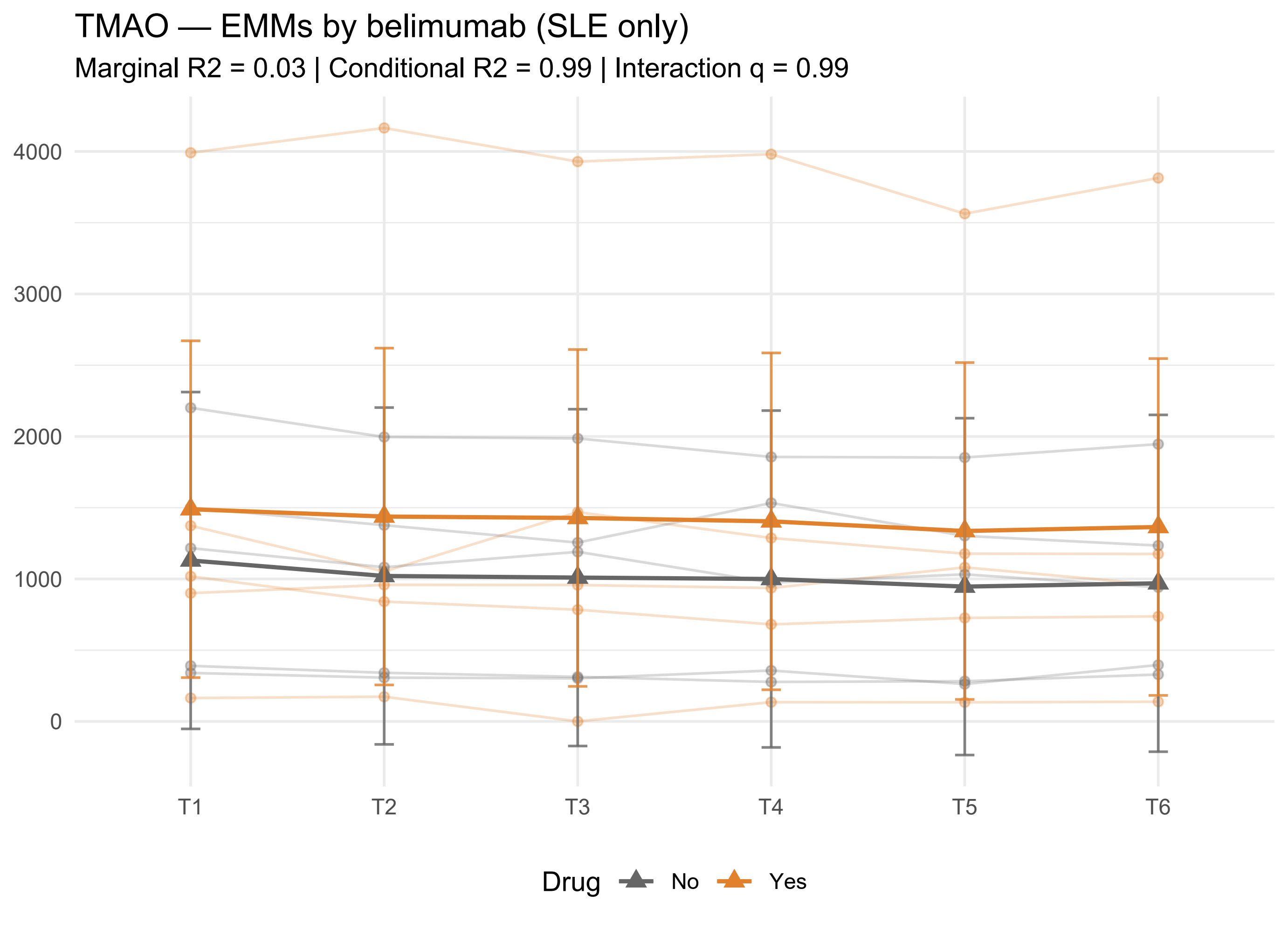
TMAO — EMMs by belimumab (SLE only)
Marginal R2 = 0.03 | Conditional R2 = 0.99 | Interaction q = 0.99
4000
3000
2000
1000
0
T1
T2
T3
T4
T5
T6
Drug
No
Yes

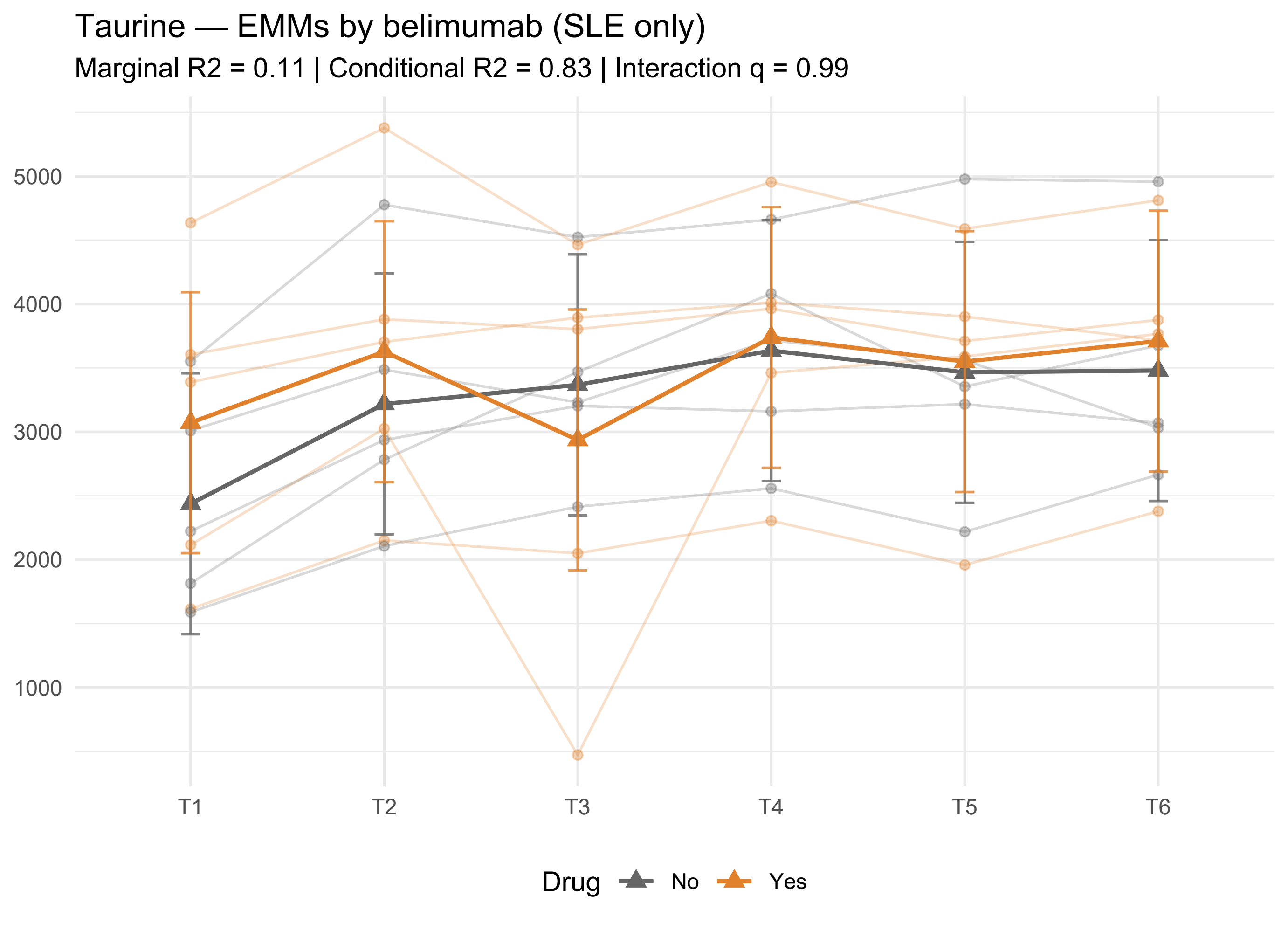
Taurine — EMMs by belimumab (SLE only)
Marginal R2 = 0.11 | Conditional R2 = 0.83 | Interaction q = 0.99
5000
4000
3000
2000
1000
T1
T2
T3
T4
T5
T6
Drug
No
Yes

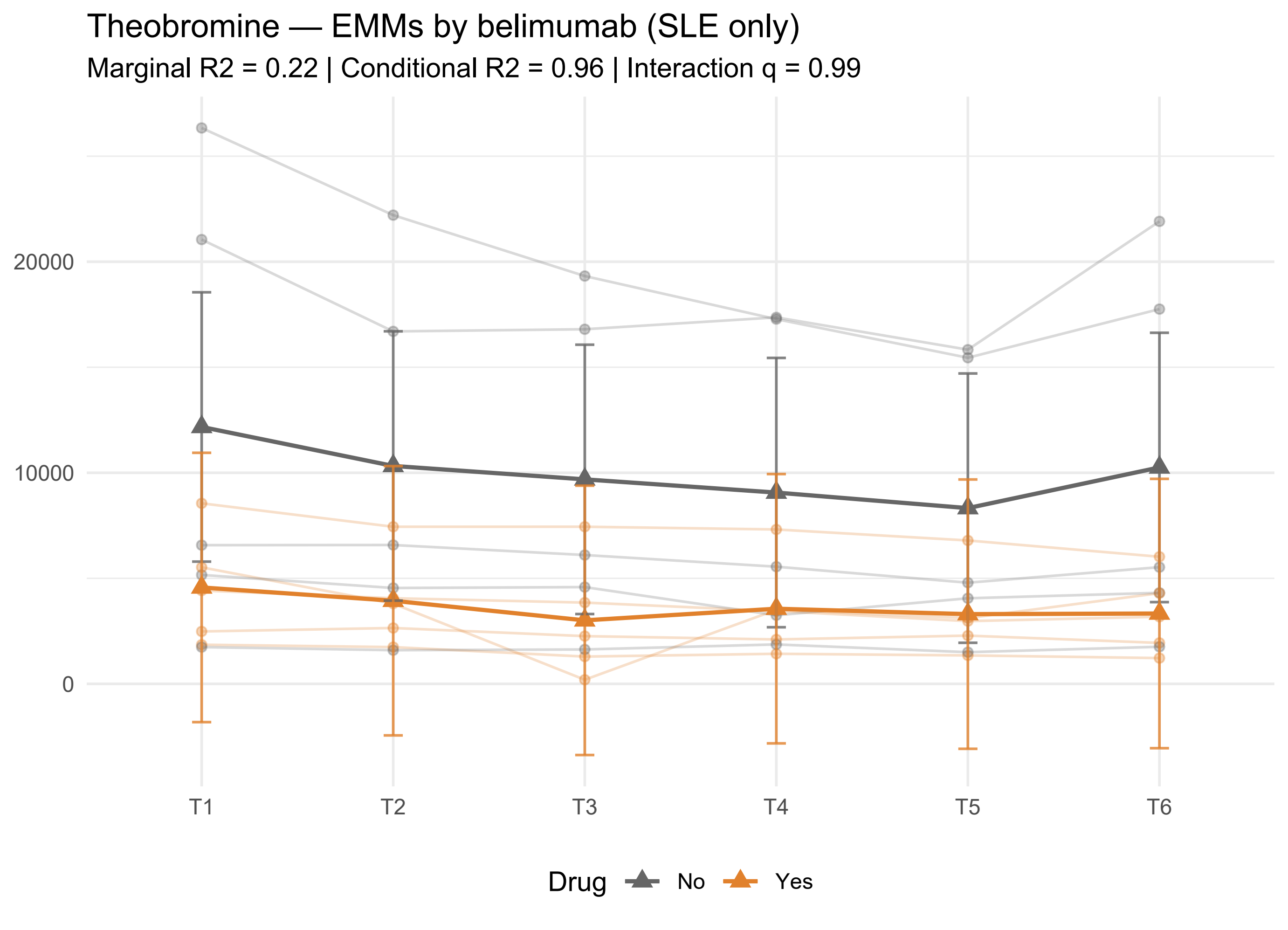
Theobromine — EMMs by belimumab (SLE only)
Marginal R2 = 0.22 | Conditional R2 = 0.96 | Interaction q = 0.99
20000
10000
0
T1
T2
T3
T4
T5
T6
Drug
No
Yes

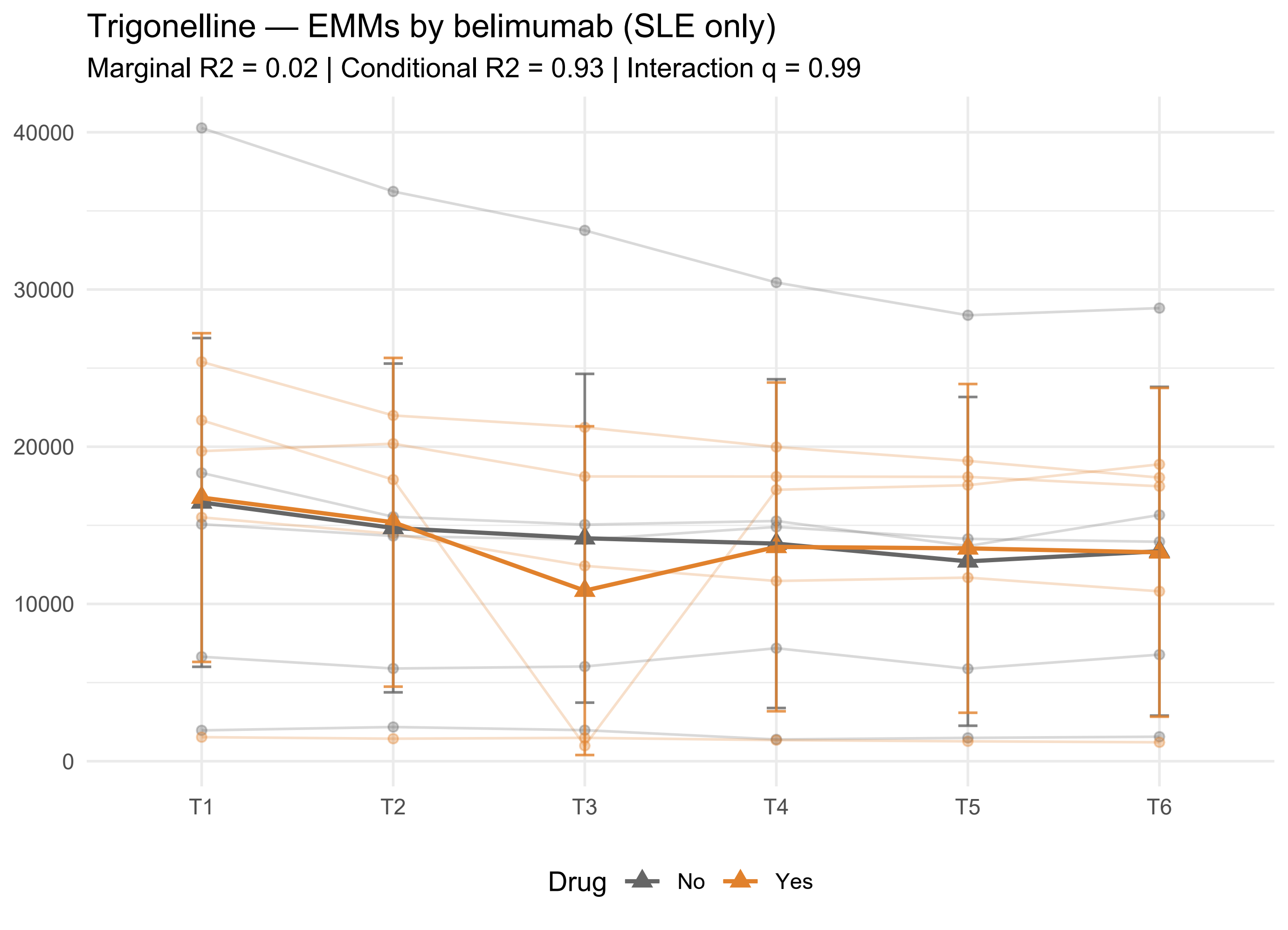

Trigonelline — EMMs by belimumab (SLE only)
Marginal R2 = 0.02 | Conditional R2 = 0.93 | Interaction q = 0.99
40000
30000
20000
10000
0
T1
T2
T3
T4
T5
T6
Drug
No
Yes

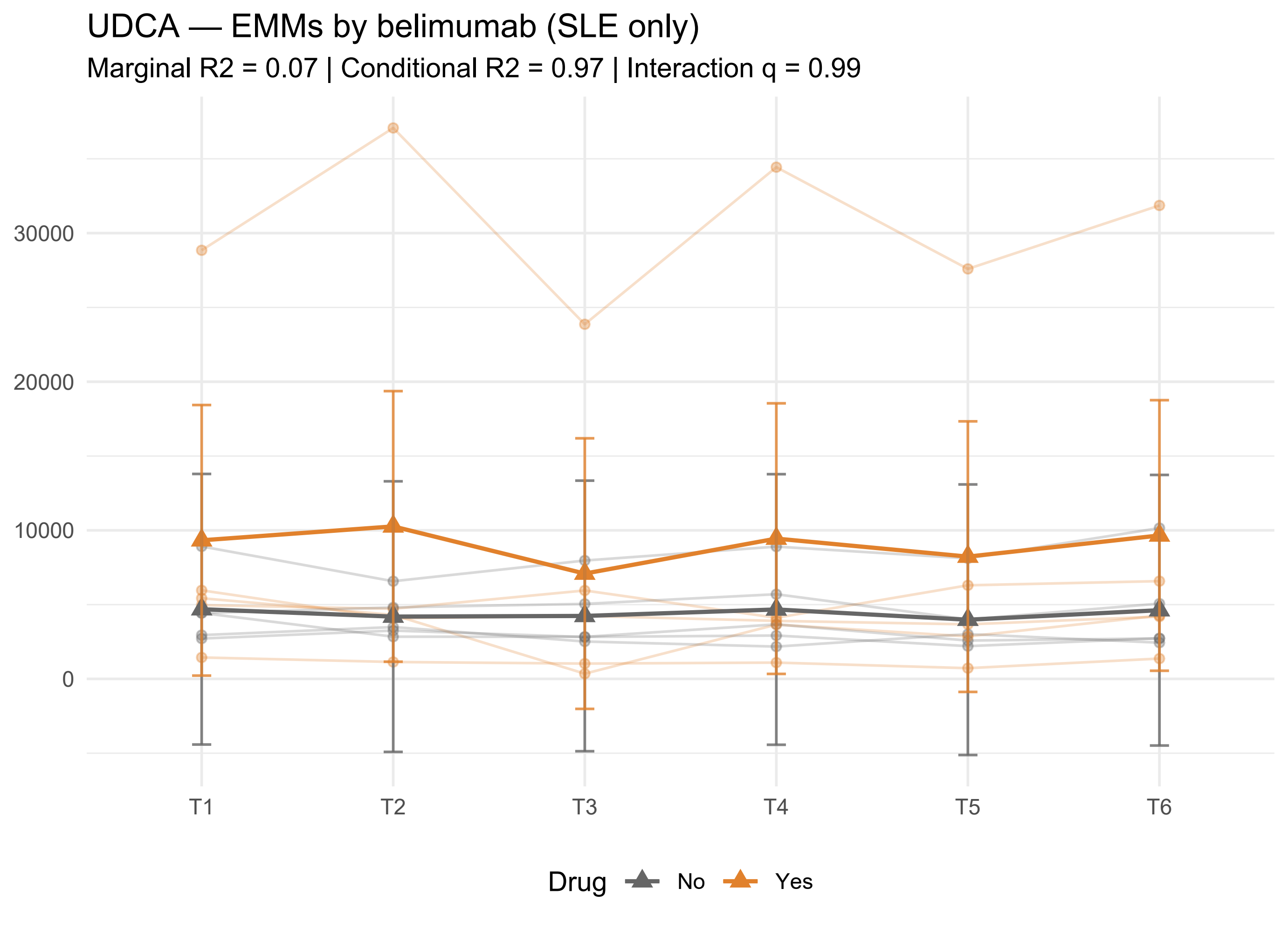

UDCA — EMMs by belimumab (SLE only)
Marginal R2 = 0.07 | Conditional R2 = 0.97 | Interaction q = 0.99
30000
20000
10000
0
T1
T2
T3
T4
T5
T6
Drug
No
Yes

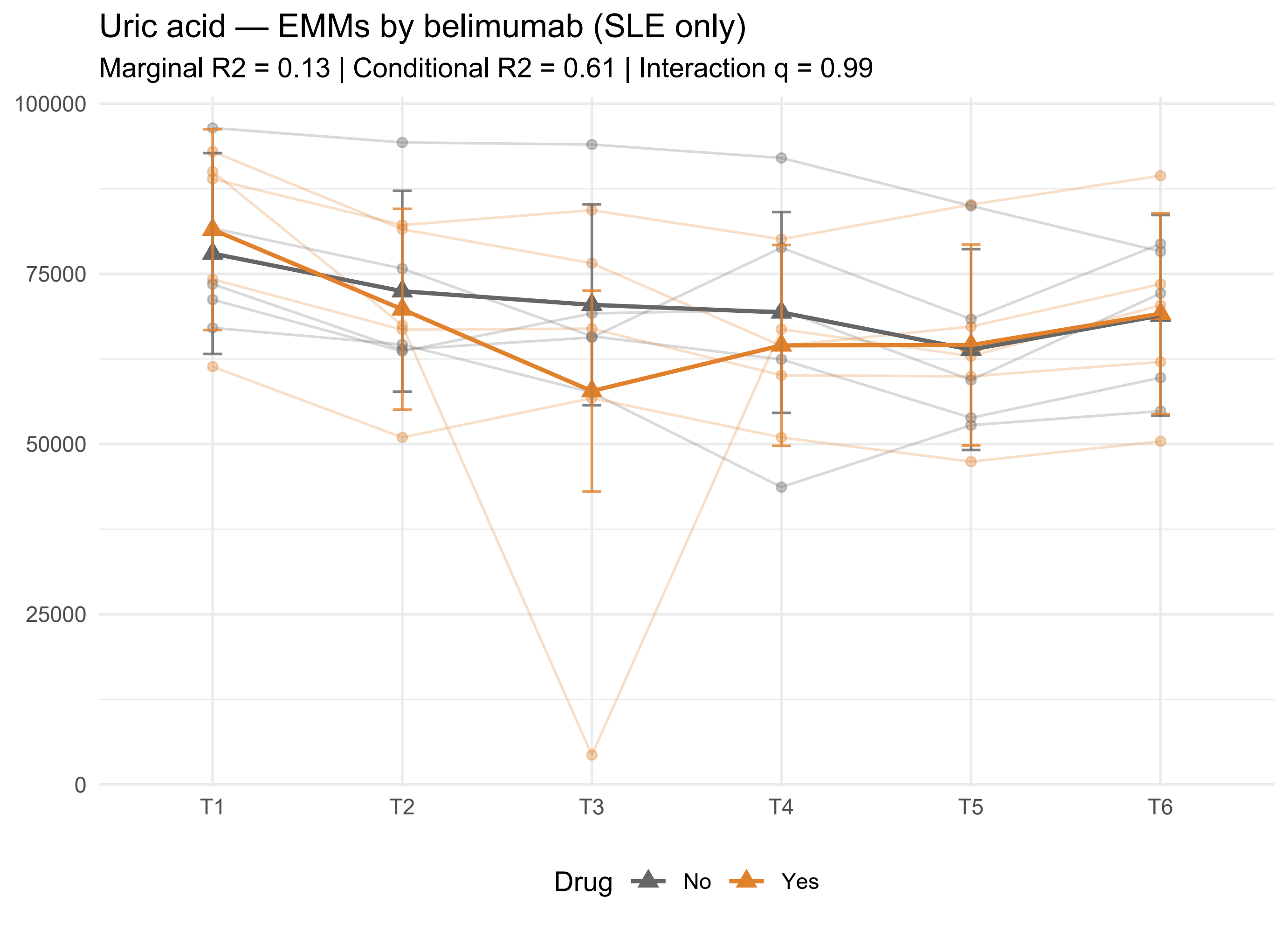

Uric acid — EMMs by belimumab (SLE only)
Marginal R2 = 0.13 | Conditional R2 = 0.61 | Interaction q = 0.99
100000
75000
50000
25000
0
T1
T2
T3
T4
T5
T6
Drug
No
Yes

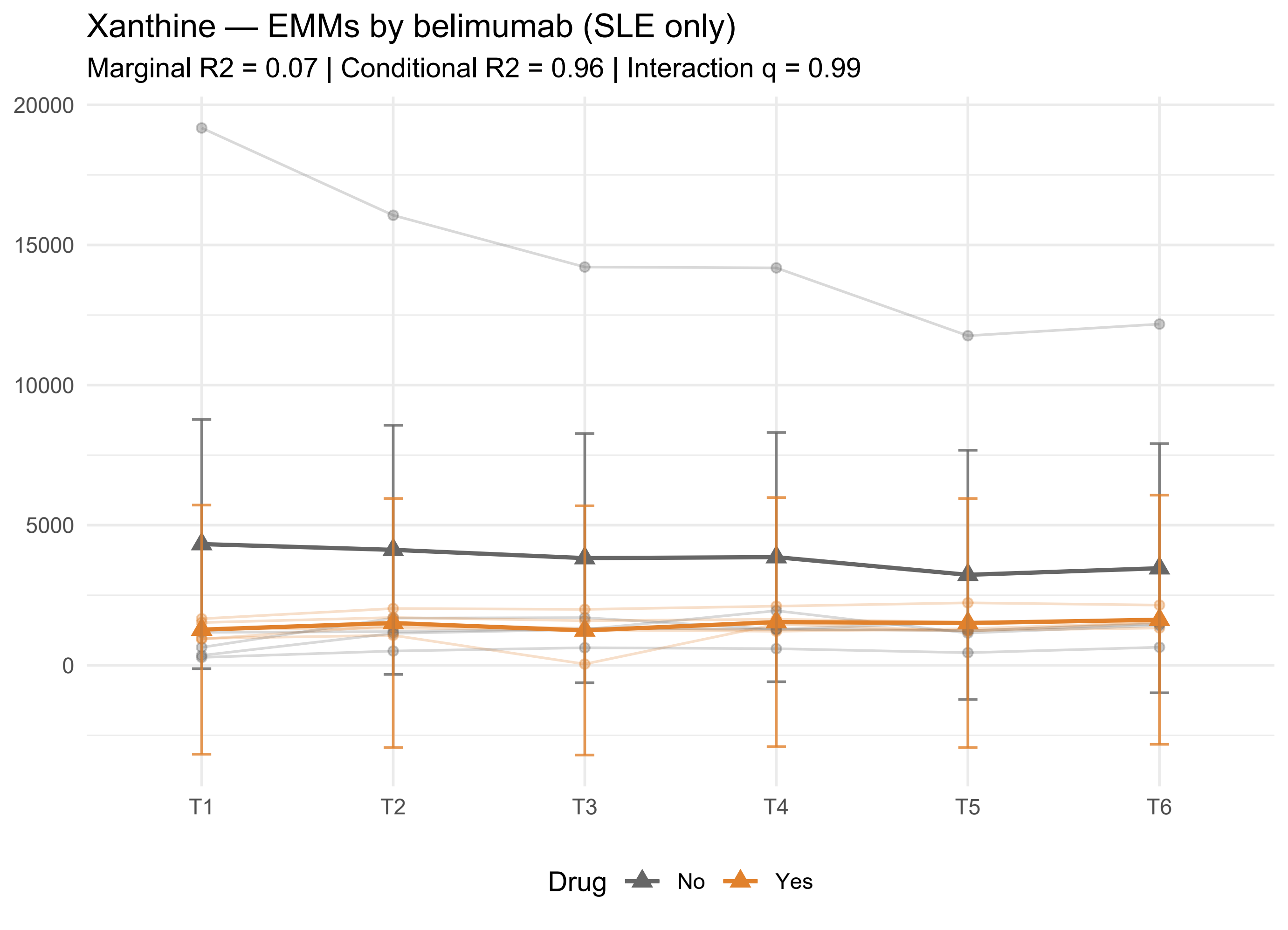
Xanthine — EMMs by belimumab (SLE only)
Marginal R2 = 0.07 | Conditional R2 = 0.96 | Interaction q = 0.99
20000
15000
10000
5000
0
T1
T2
T3
T4
T5
T6
Drug
No
Yes

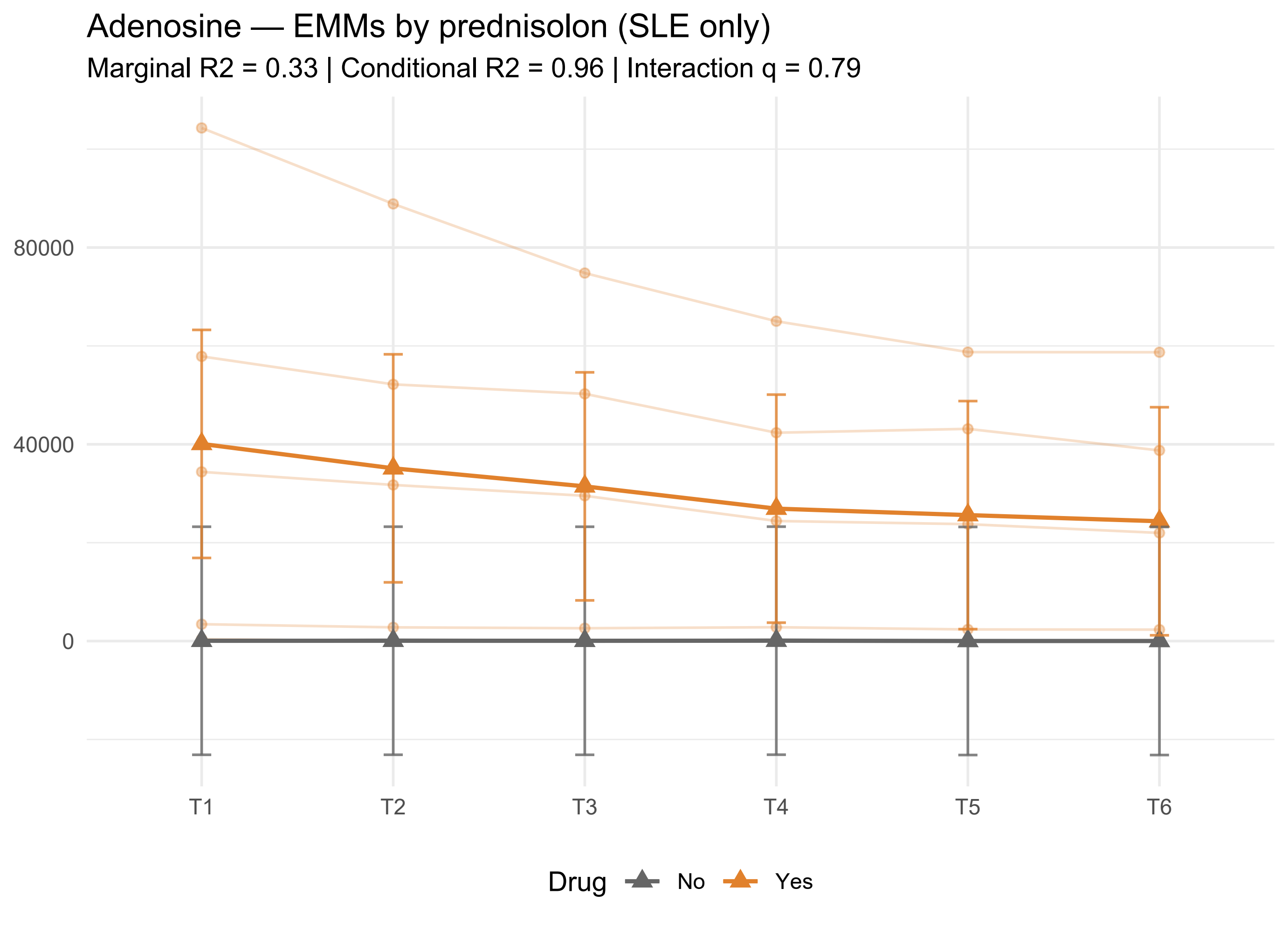

Adenosine — EMMs by prednisolon (SLE only)
Marginal R2 = 0.33 | Conditional R2 = 0.96 | Interaction q = 0.79
80000
40000
0
T1
T2
T3
T4
T5
T6
Drug
No
Yes

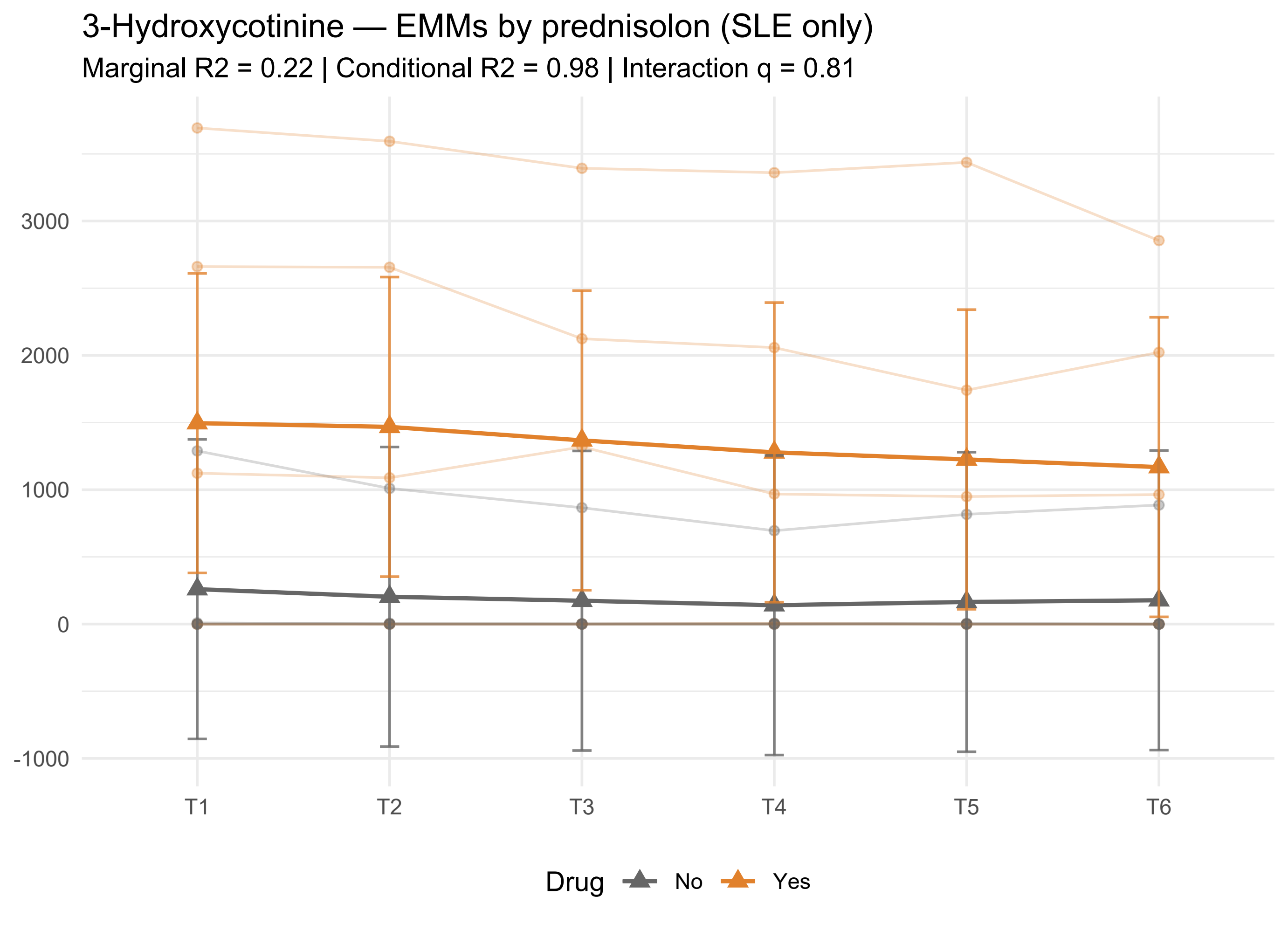

3-Hydroxycotinine — EMMs by prednisolon (SLE only)
Marginal R2 = 0.22 | Conditional R2 = 0.98 | Interaction q = 0.81
3000
2000
1000
0
-1000
T1
T2
T3
T4
T5
T6
Drug
No
Yes

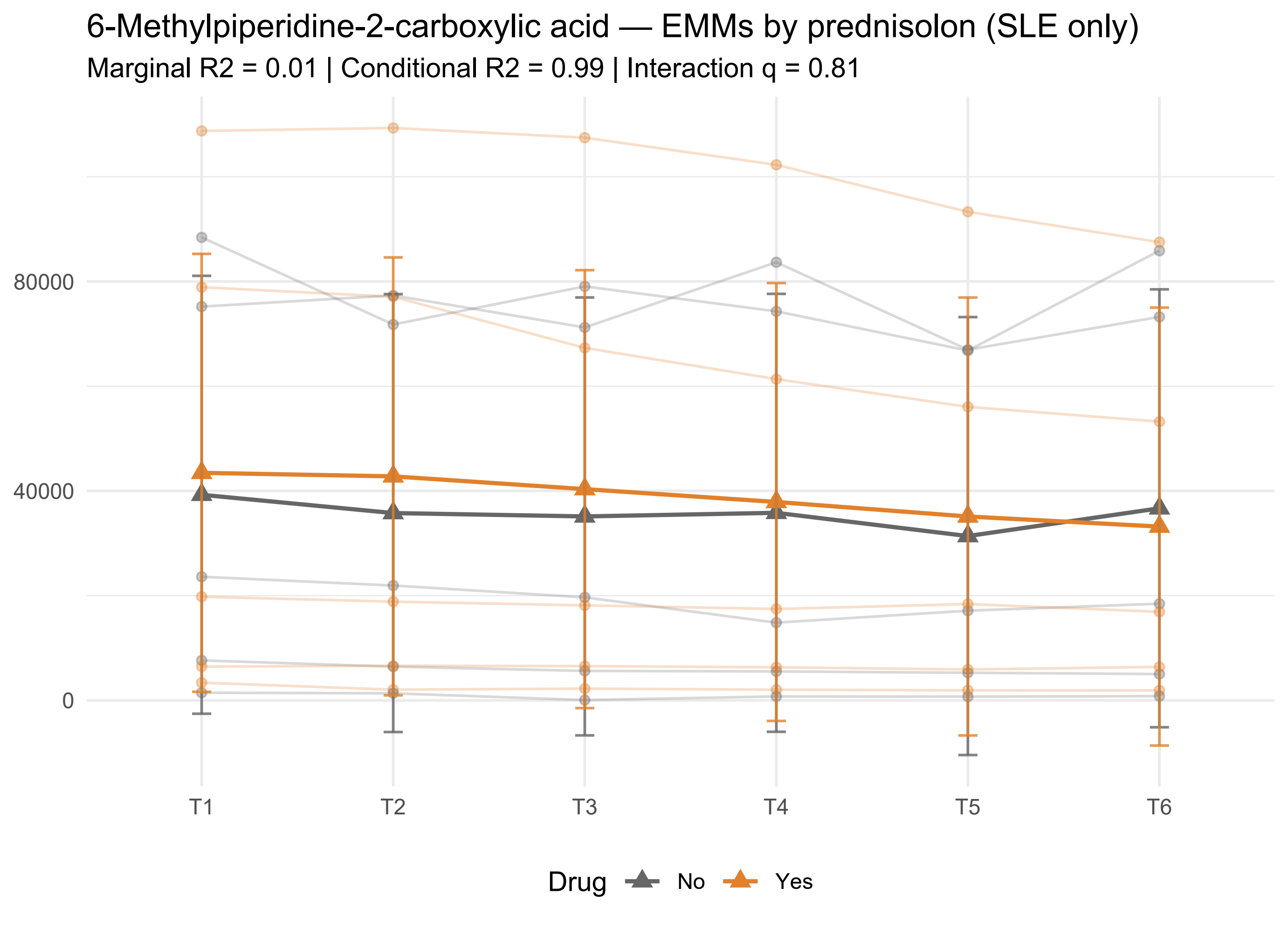
6-Methylpiperidine-2-carboxylic acid — EMMs by prednisolon (SLE only)
Marginal R2 = 0.01 | Conditional R2 = 0.99 | Interaction q = 0.81
80000
40000
0
T1
T2
T3
T4
T5
T6
Drug
No
Yes

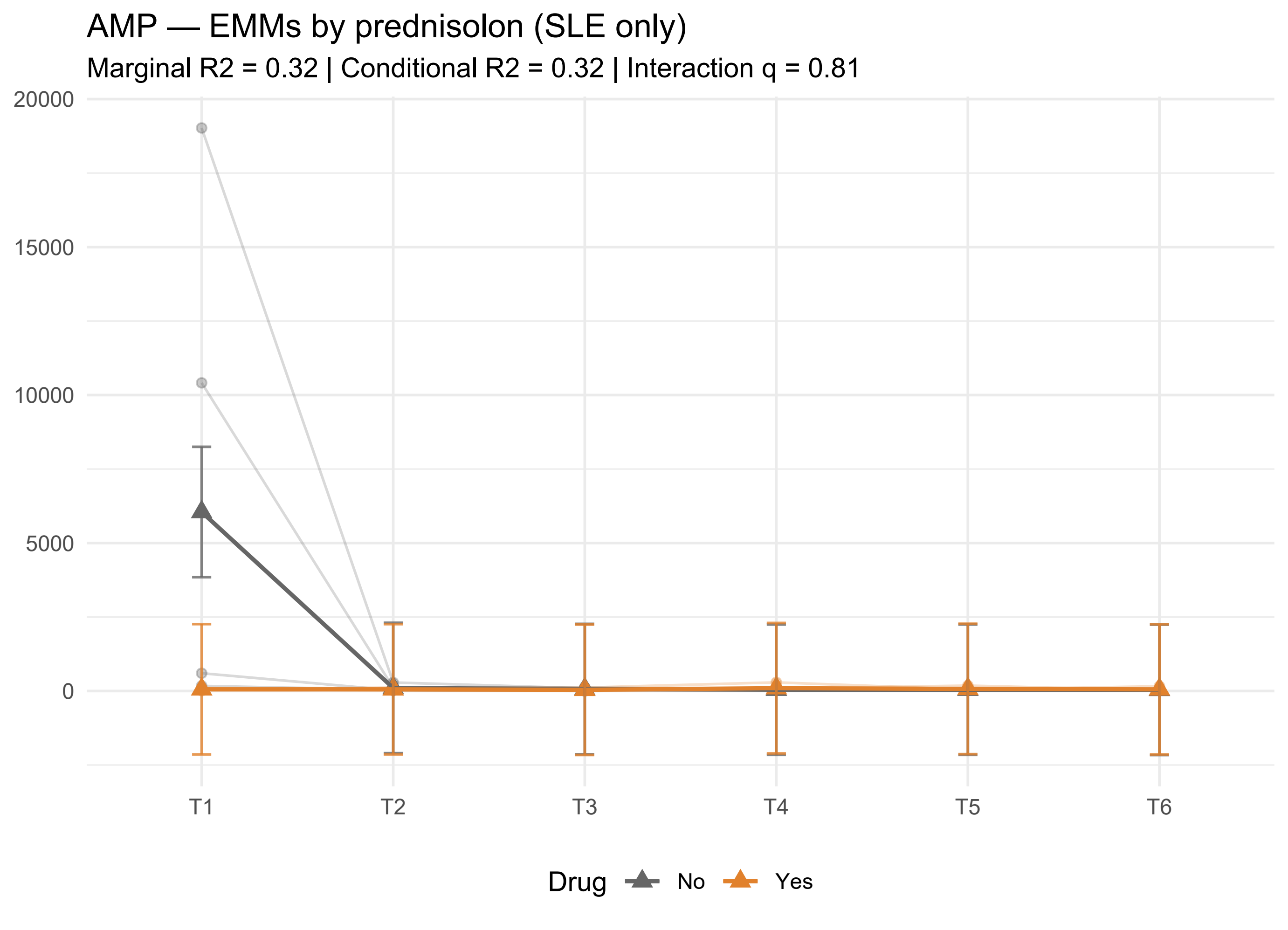

AMP — EMMs by prednisolon (SLE only)
Marginal R2 = 0.32 | Conditional R2 = 0.32 | Interaction q = 0.81
20000
15000
10000
5000
0
T1
T2
T3
T4
T5
T6
Drug
No
Yes

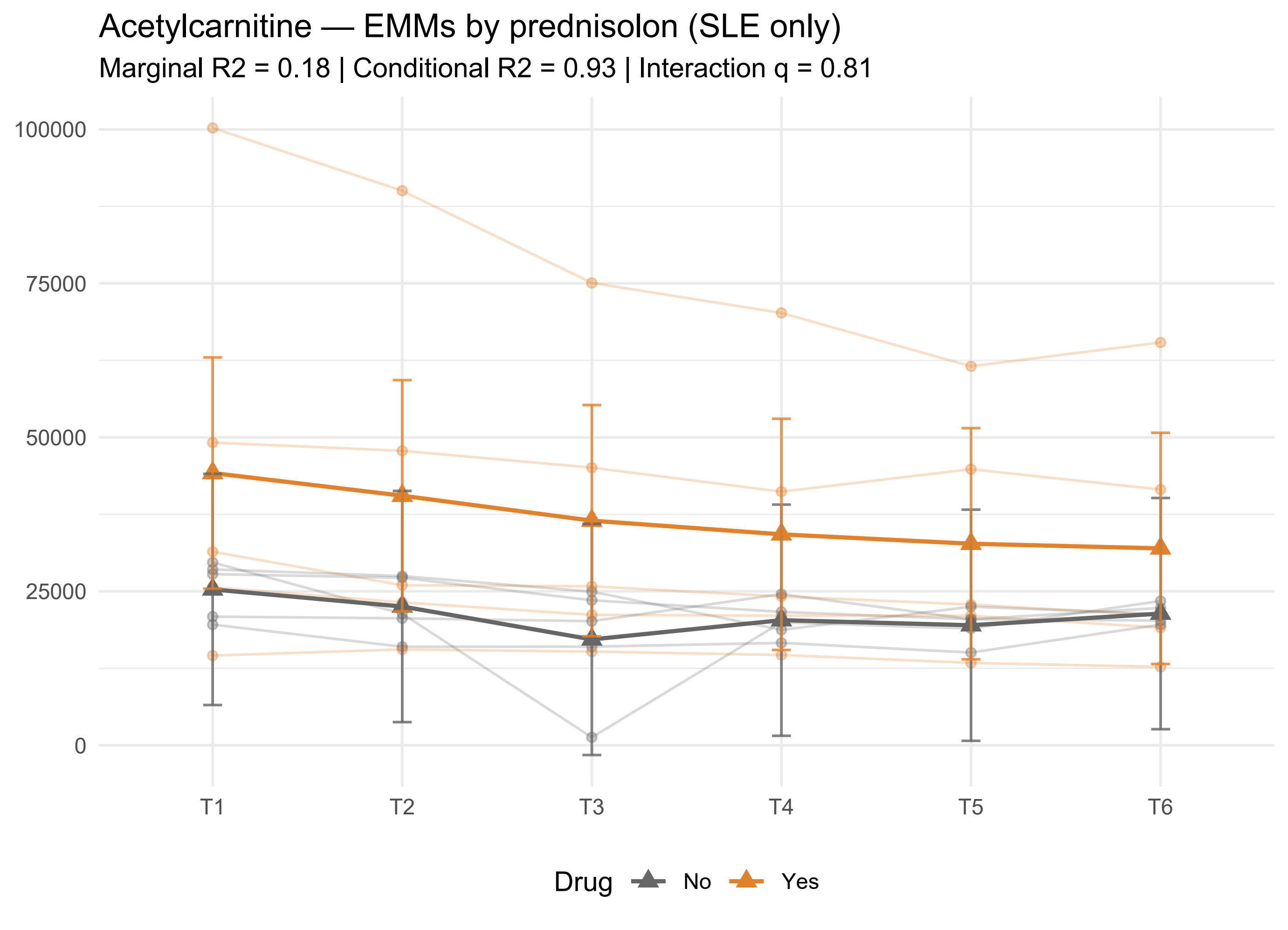
Acetylcarnitine — EMMs by prednisolon (SLE only)
Marginal R2 = 0.18 | Conditional R2 = 0.93 | Interaction q = 0.81
100000
75000
50000
25000
0
T1
T2
T3
T4
T5
T6
Drug
No
Yes

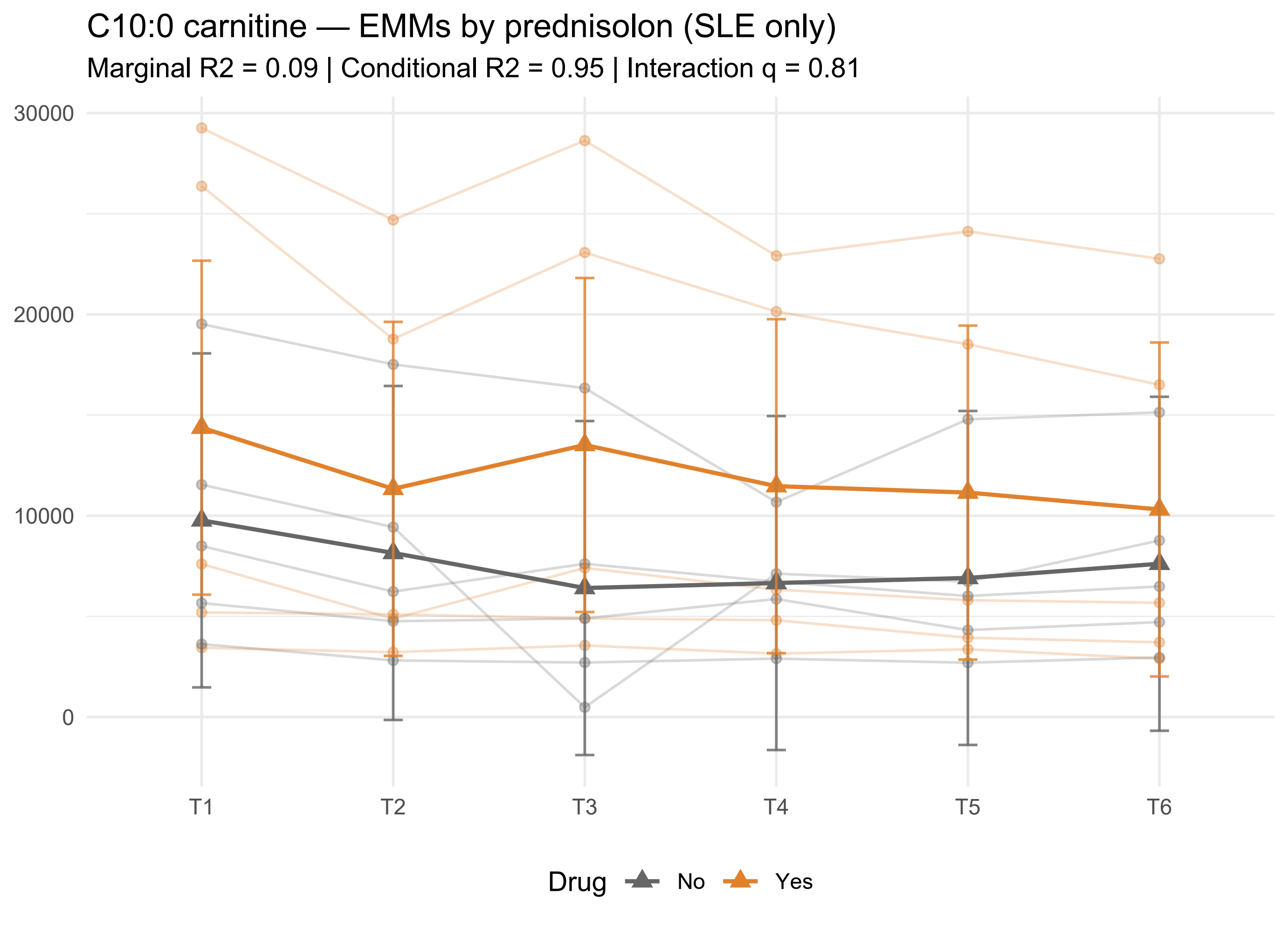

C10:0 carnitine — EMMs by prednisolon (SLE only)
Marginal R2 = 0.09 | Conditional R2 = 0.95 | Interaction q = 0.81
30000
20000
10000
0
T1
T2
T3
T4
T5
T6
Drug
No
Yes

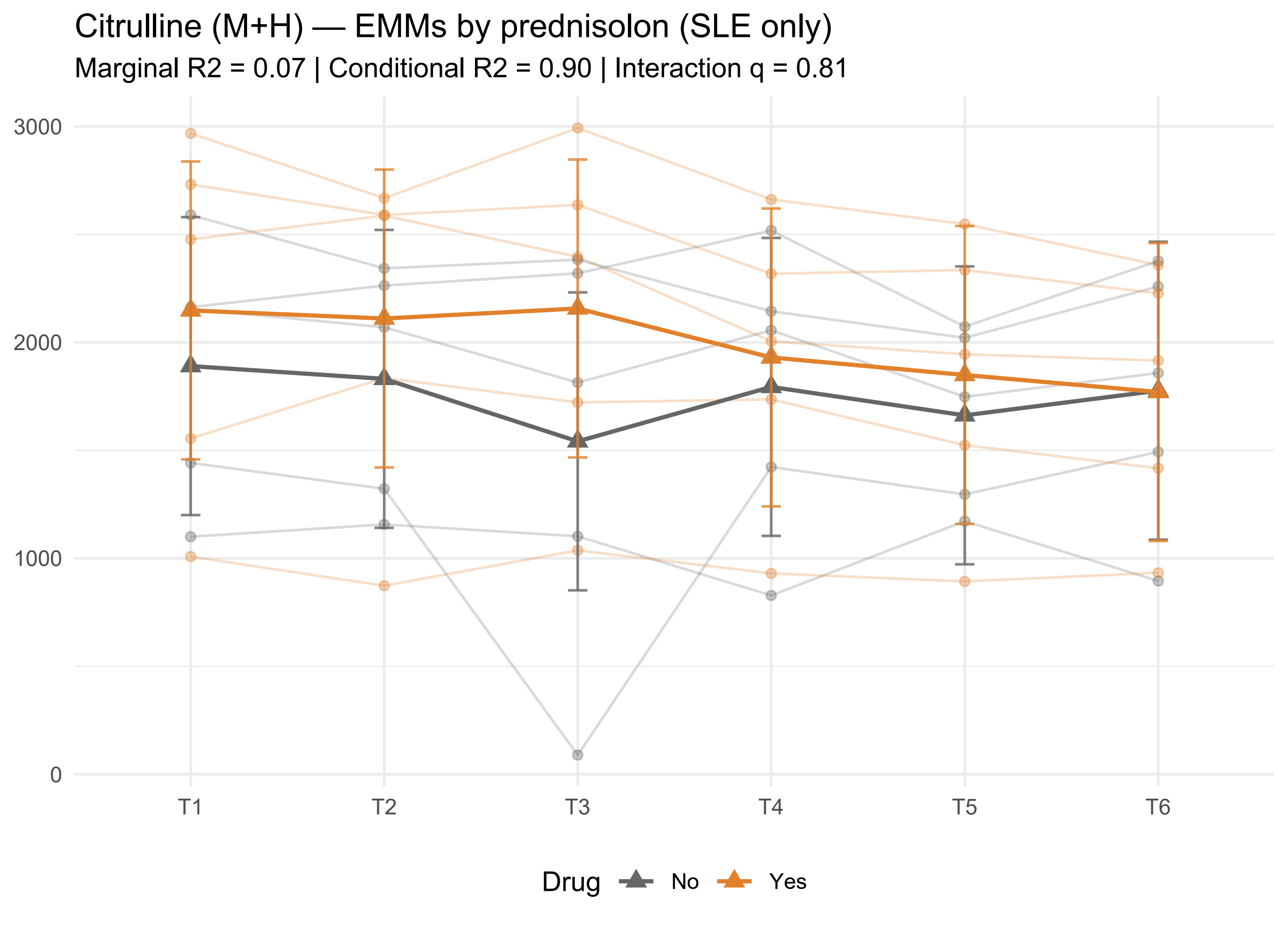
Citrulline (M+H) — EMMs by prednisolon (SLE only)
Marginal R2 = 0.07 | Conditional R2 = 0.90 | Interaction q = 0.81
3000
2000
1000
0
T1
T2
T3
T4
T5
T6
Drug
No
Yes

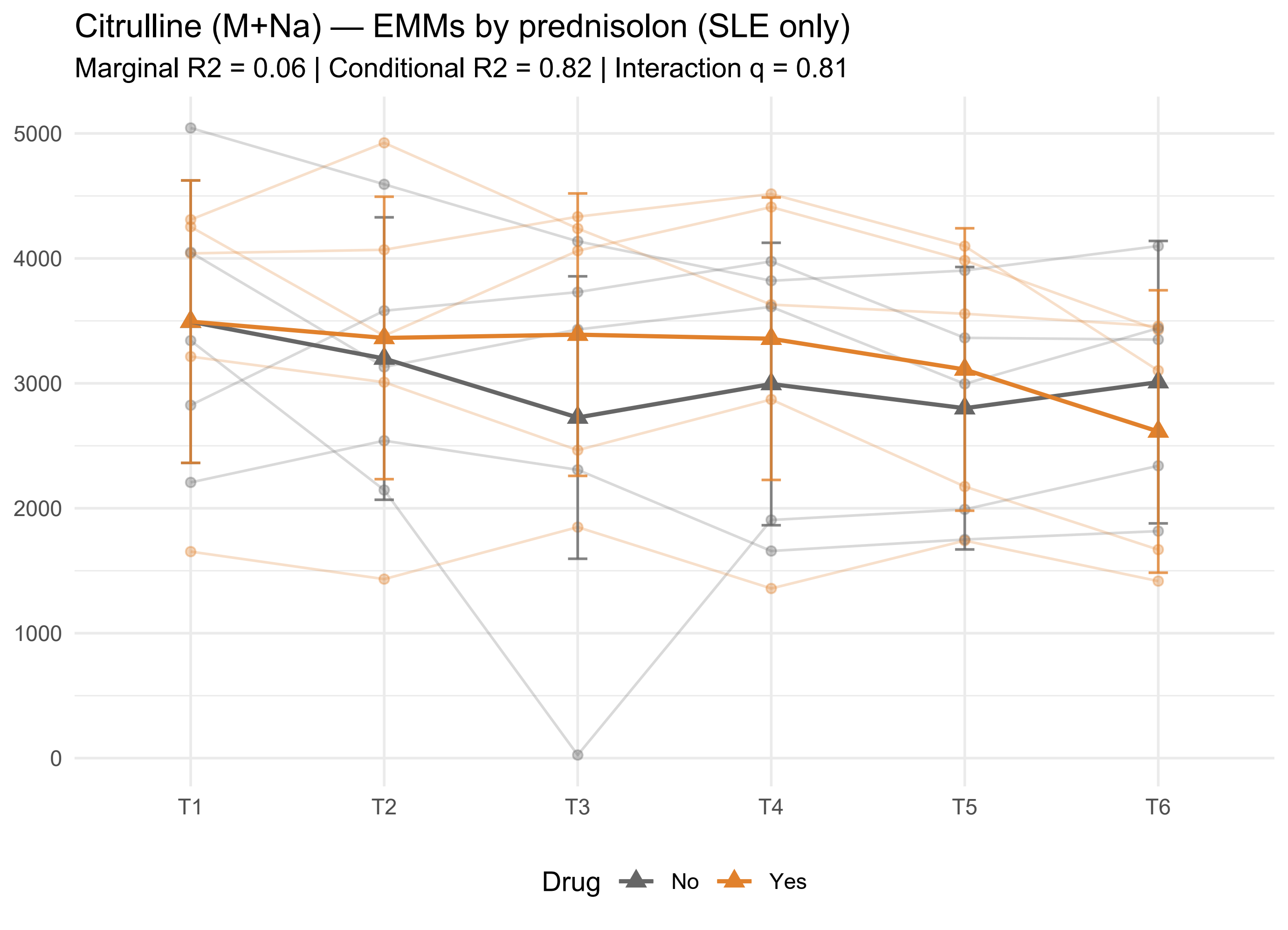

Citrulline (M+Na) — EMMs by prednisolon (SLE only)
Marginal R2 = 0.06 | Conditional R2 = 0.82 | Interaction q = 0.81
5000
4000
3000
2000
1000
0
T1
T2
T3
T4
T5
T6
Drug
No
Yes

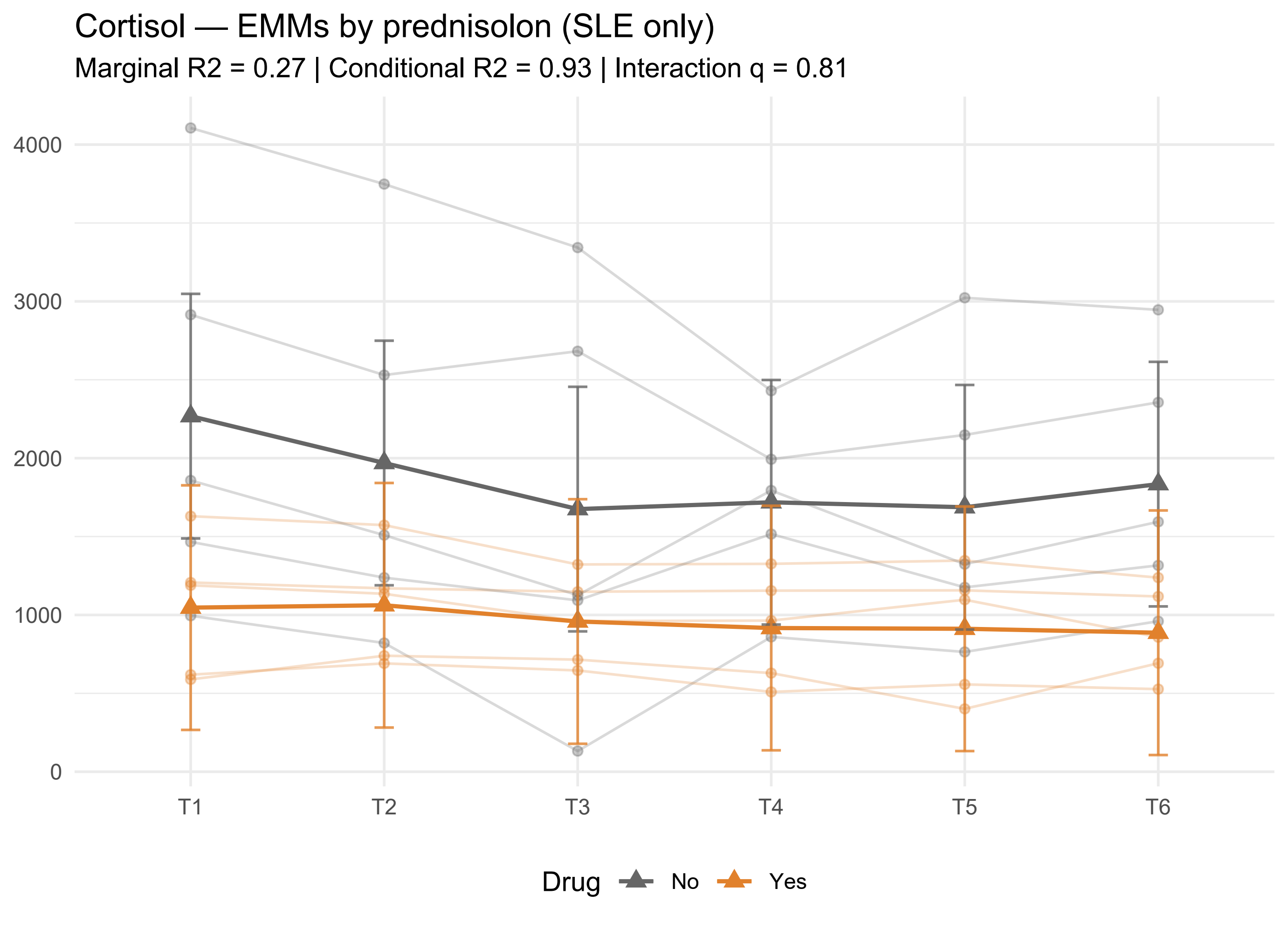
Cortisol — EMMs by prednisolon (SLE only)
Marginal R2 = 0.27 | Conditional R2 = 0.93 | Interaction q = 0.81
4000
3000
2000
1000
0
T1
T2
T3
T4
T5
T6
Drug
No
Yes

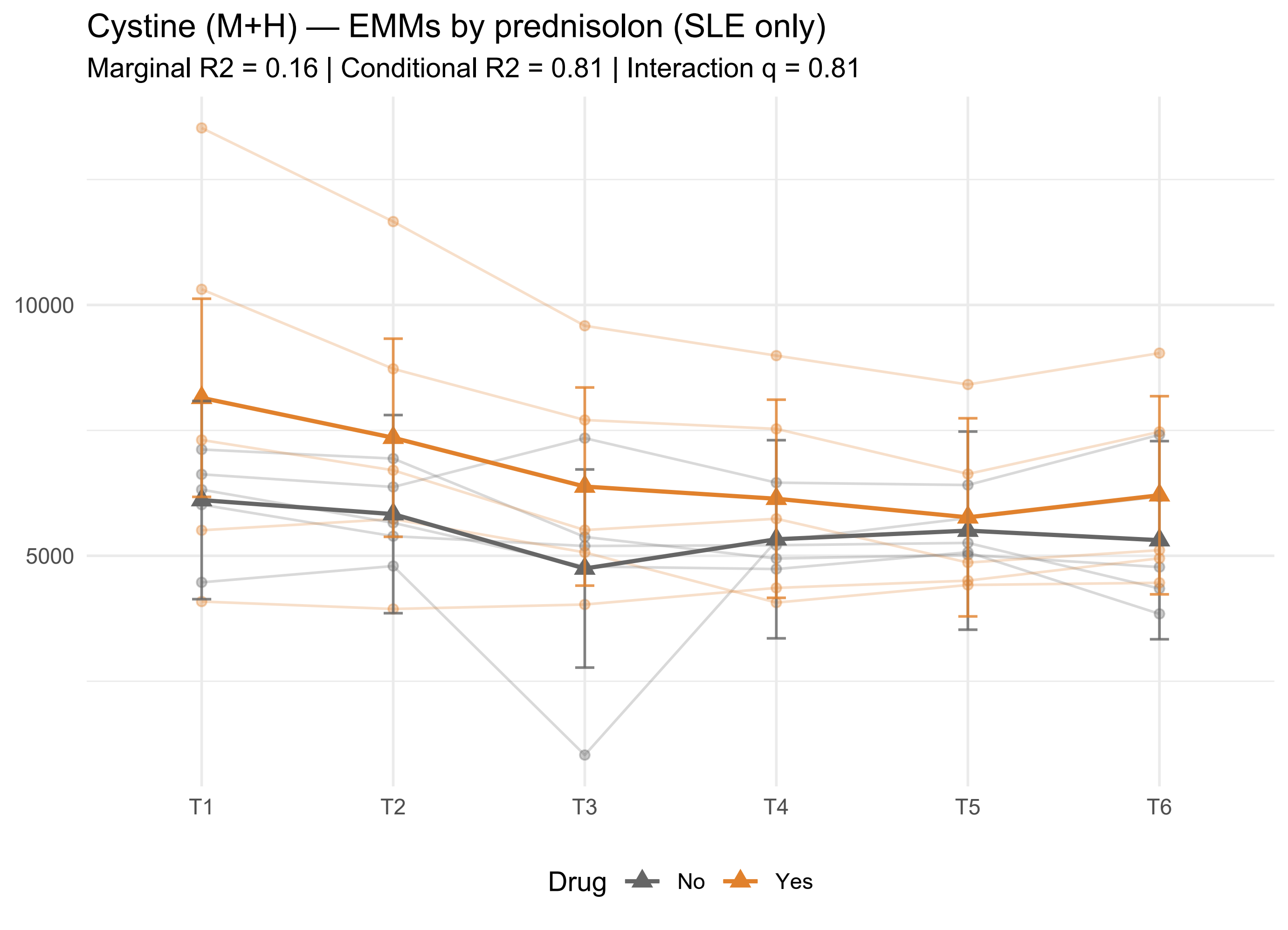
Cystine (M+H) — EMMs by prednisolon (SLE only)
Marginal R2 = 0.16 | Conditional R2 = 0.81 | Interaction q = 0.81
10000
5000
T1
T2
T3
T4
T5
T6
Drug
No
Yes

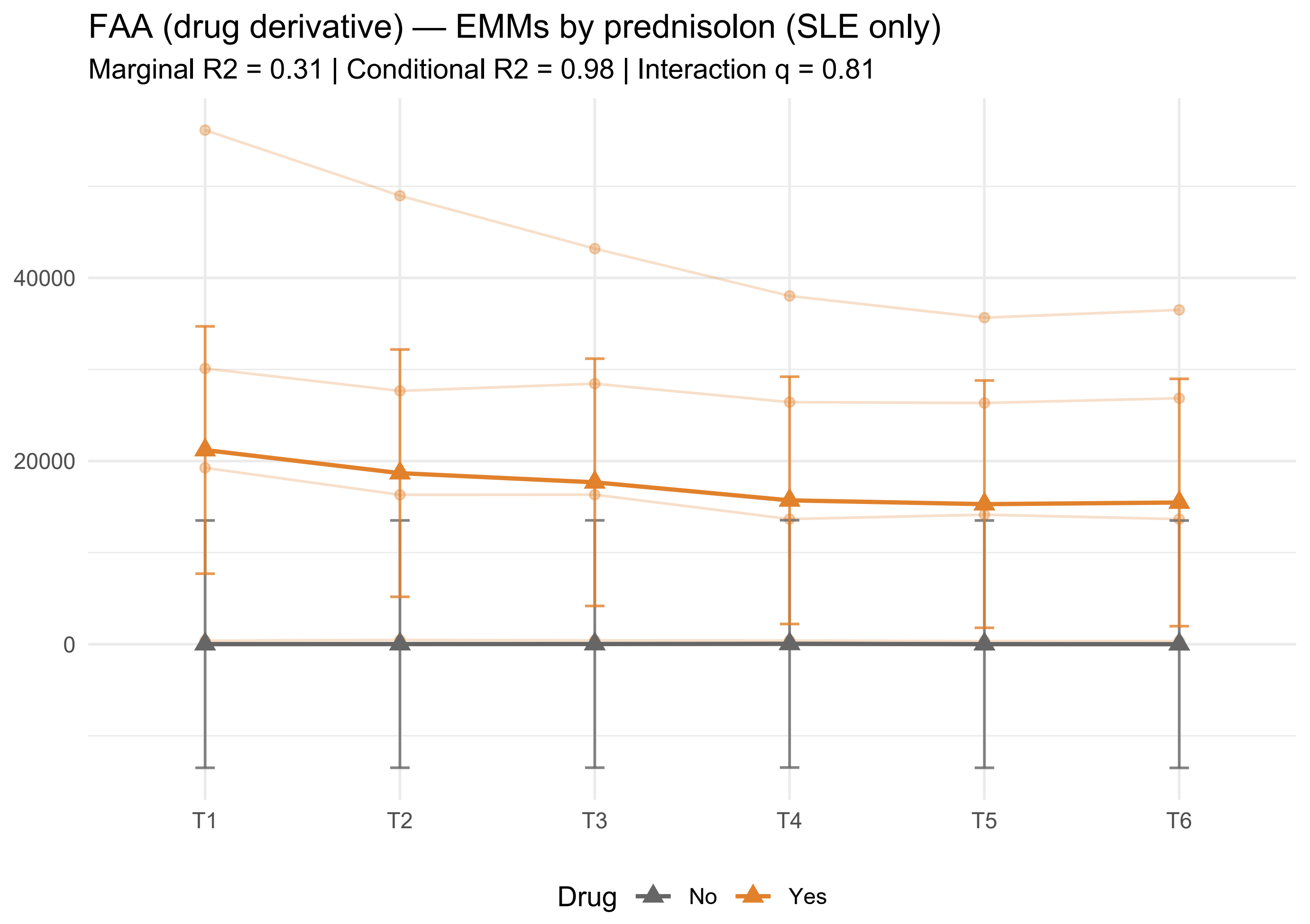
FAA (drug derivative) — EMMs by prednisolon (SLE only)
Marginal R2 = 0.31 | Conditional R2 = 0.98 | Interaction q = 0.81
40000
20000
0
T1
T2
T3
T4
T5
T6
Drug
No
Yes

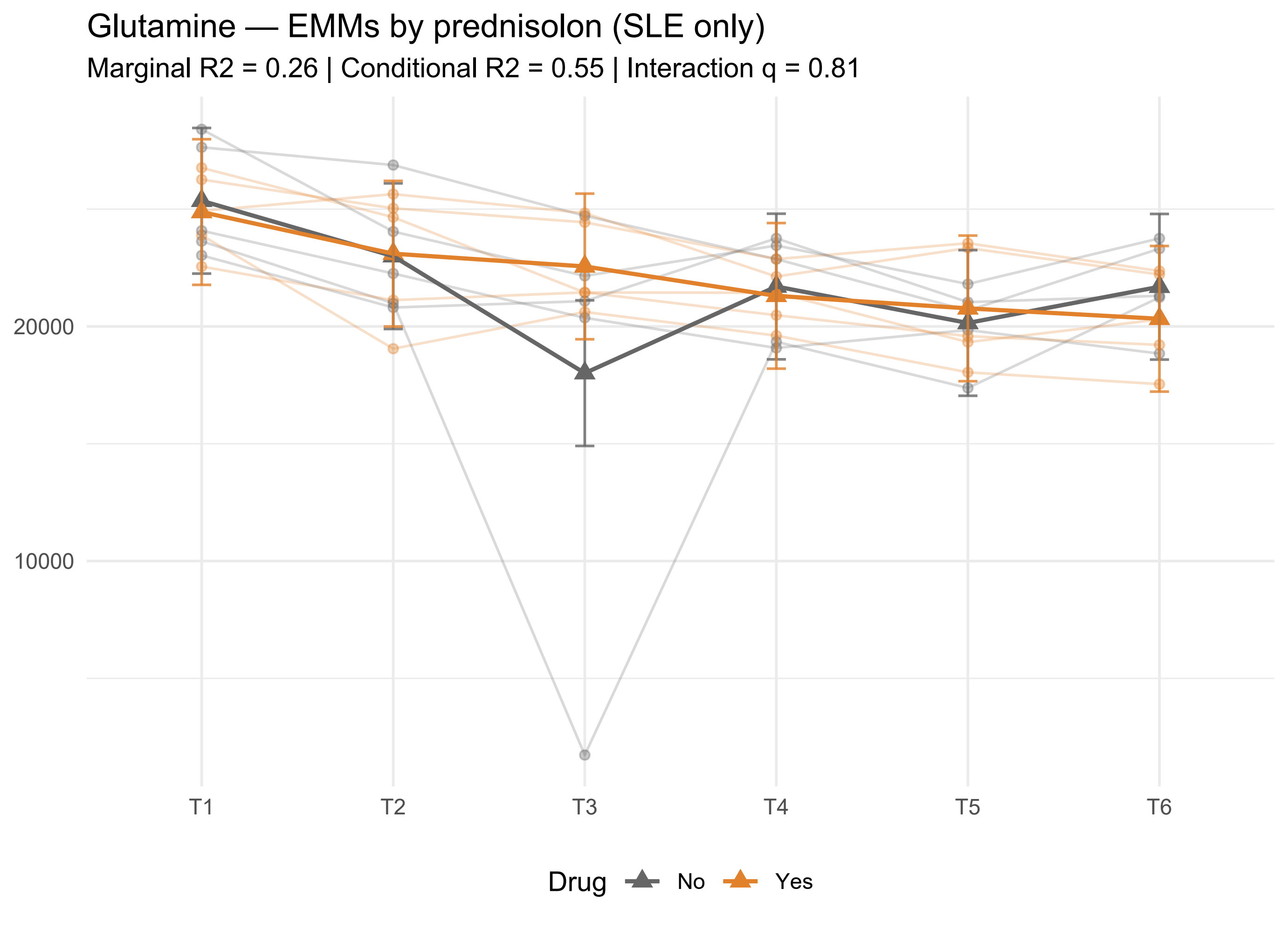

Glutamine — EMMs by prednisolon (SLE only)
Marginal R2 = 0.26 | Conditional R2 = 0.55 | Interaction q = 0.81
20000
10000
T1
T2
T3
T4
T5
T6
Drug
No
Yes

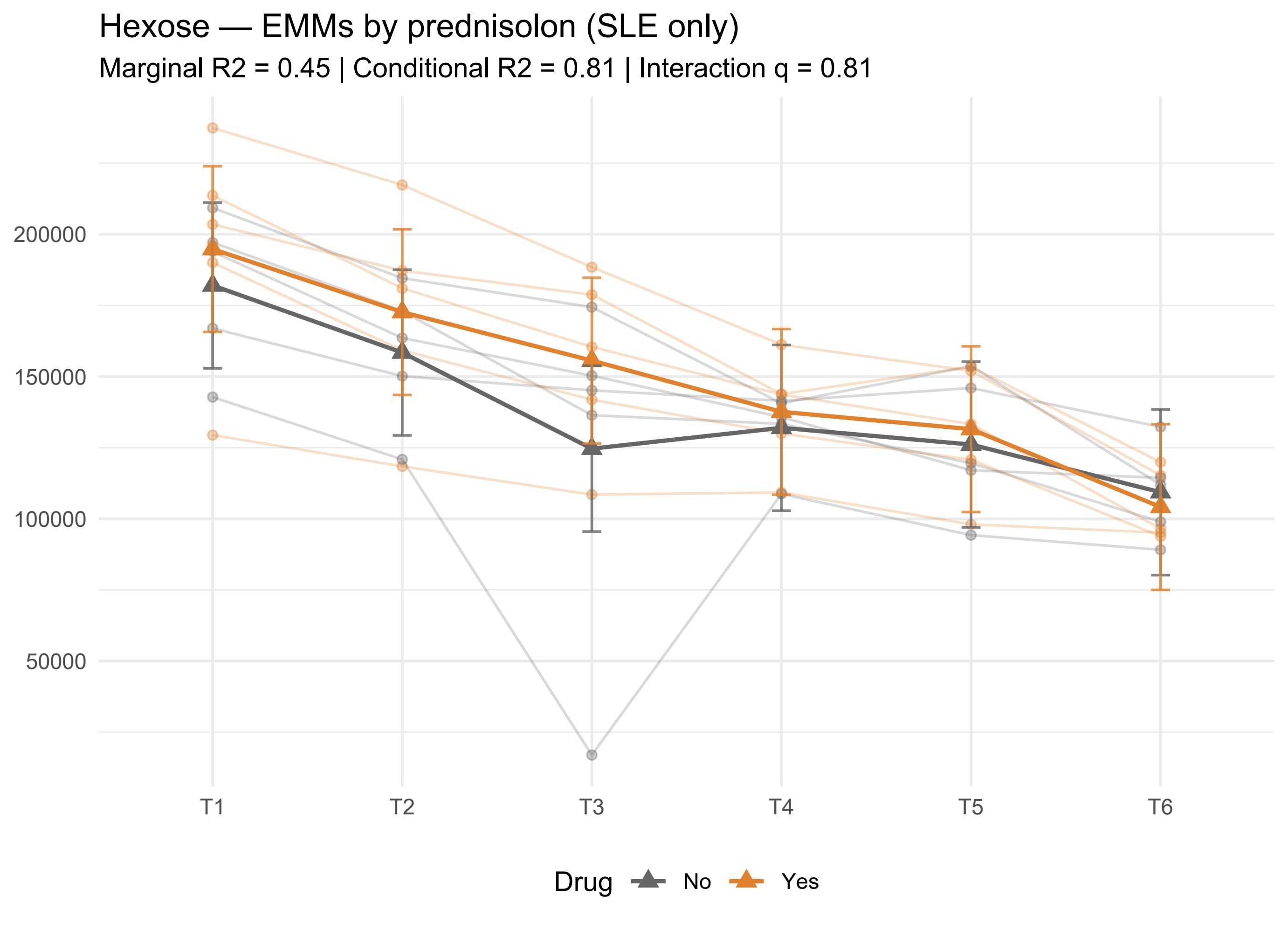
Hexose — EMMs by prednisolon (SLE only)
Marginal R2 = 0.45 | Conditional R2 = 0.81 | Interaction q = 0.81
200000
150000
100000
50000
T1
T2
T3
T4
T5
T6
Drug
No
Yes

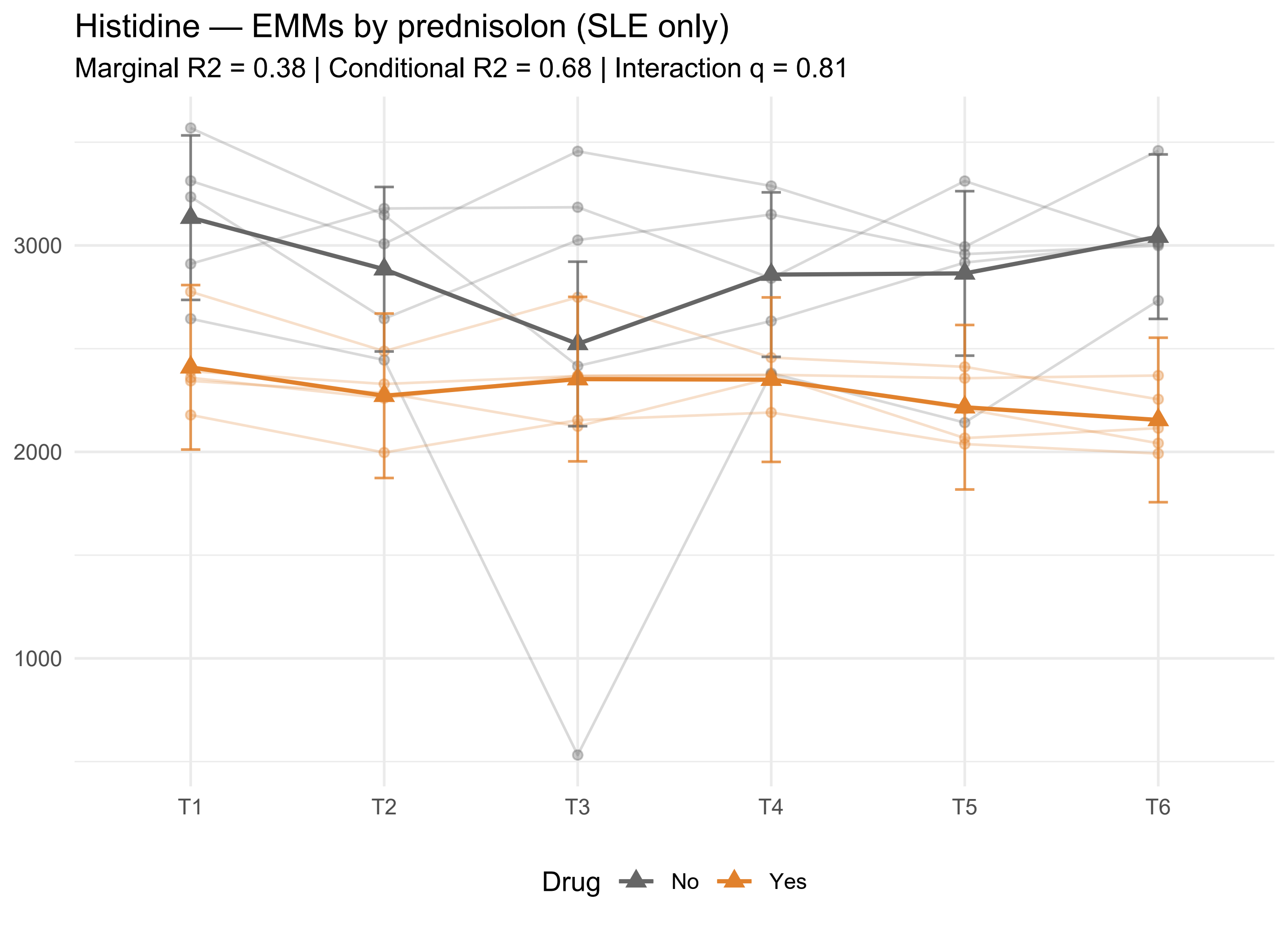
Histidine — EMMs by prednisolon (SLE only)
Marginal R2 = 0.38 | Conditional R2 = 0.68 | Interaction q = 0.81
3000
2000
1000
T1
T2
T3
T4
T5
T6
Drug
No
Yes

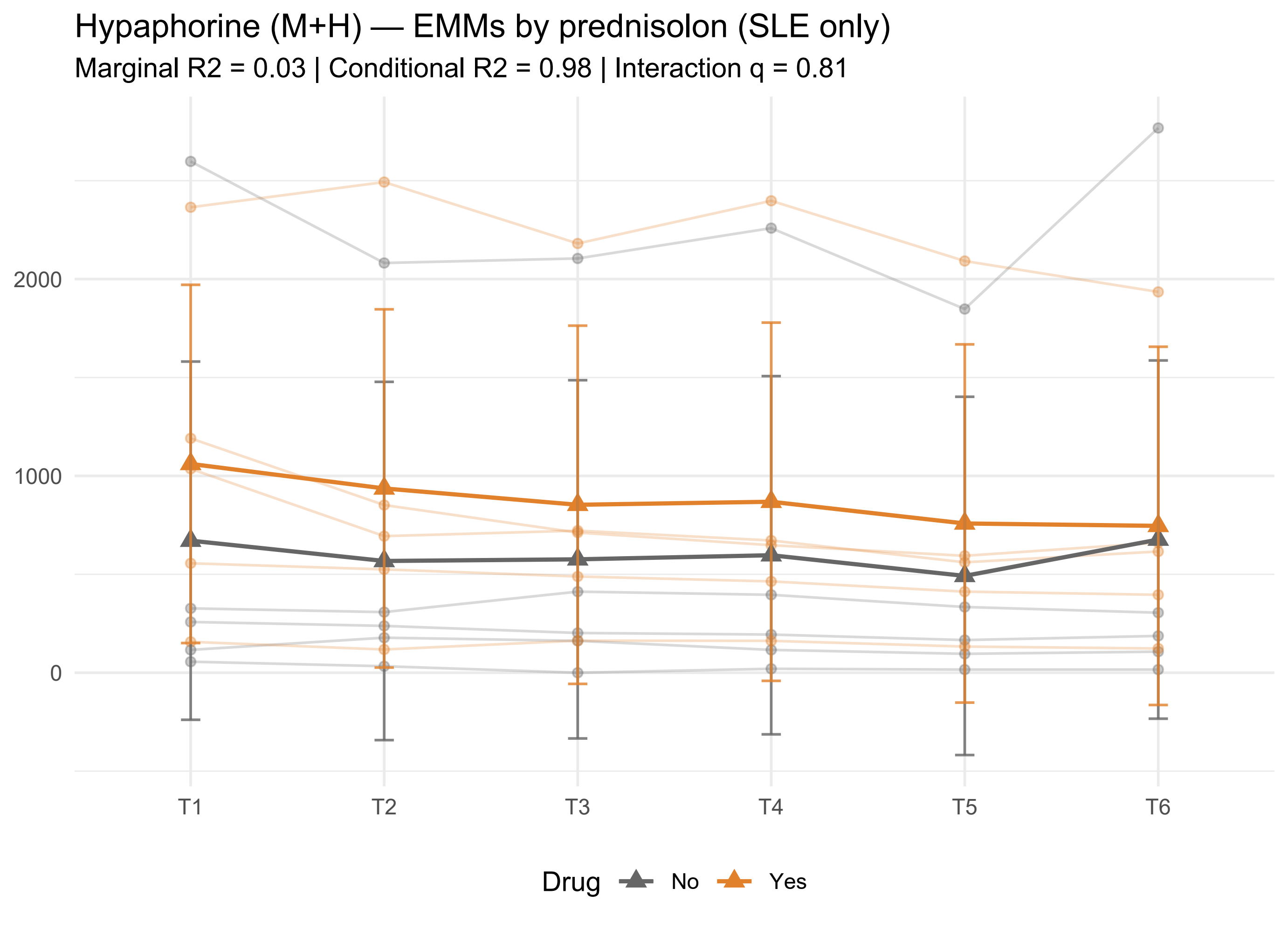
Hypaphorine (M+H) — EMMs by prednisolon (SLE only)
Marginal R2 = 0.03 | Conditional R2 = 0.98 | Interaction q = 0.81
2000
1000
0
T1
T2
T3
T4
T5
T6
Drug
No
Yes

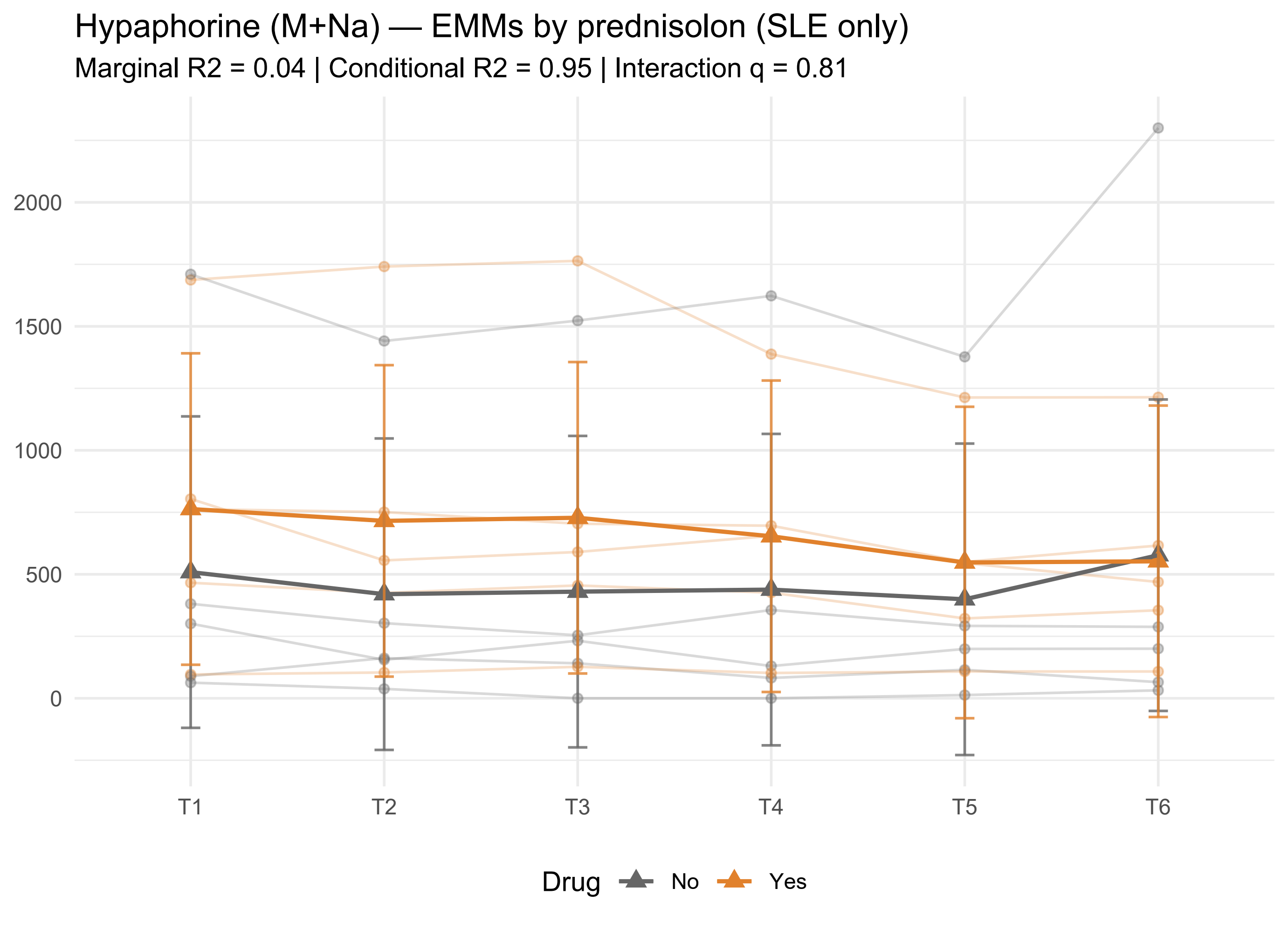

Hypaphorine (M+Na) — EMMs by prednisolon (SLE only)
Marginal R2 = 0.04 | Conditional R2 = 0.95 | Interaction q = 0.81
2000
1500
1000
500
0
T1
T2
T3
T4
T5
T6
Drug
No
Yes

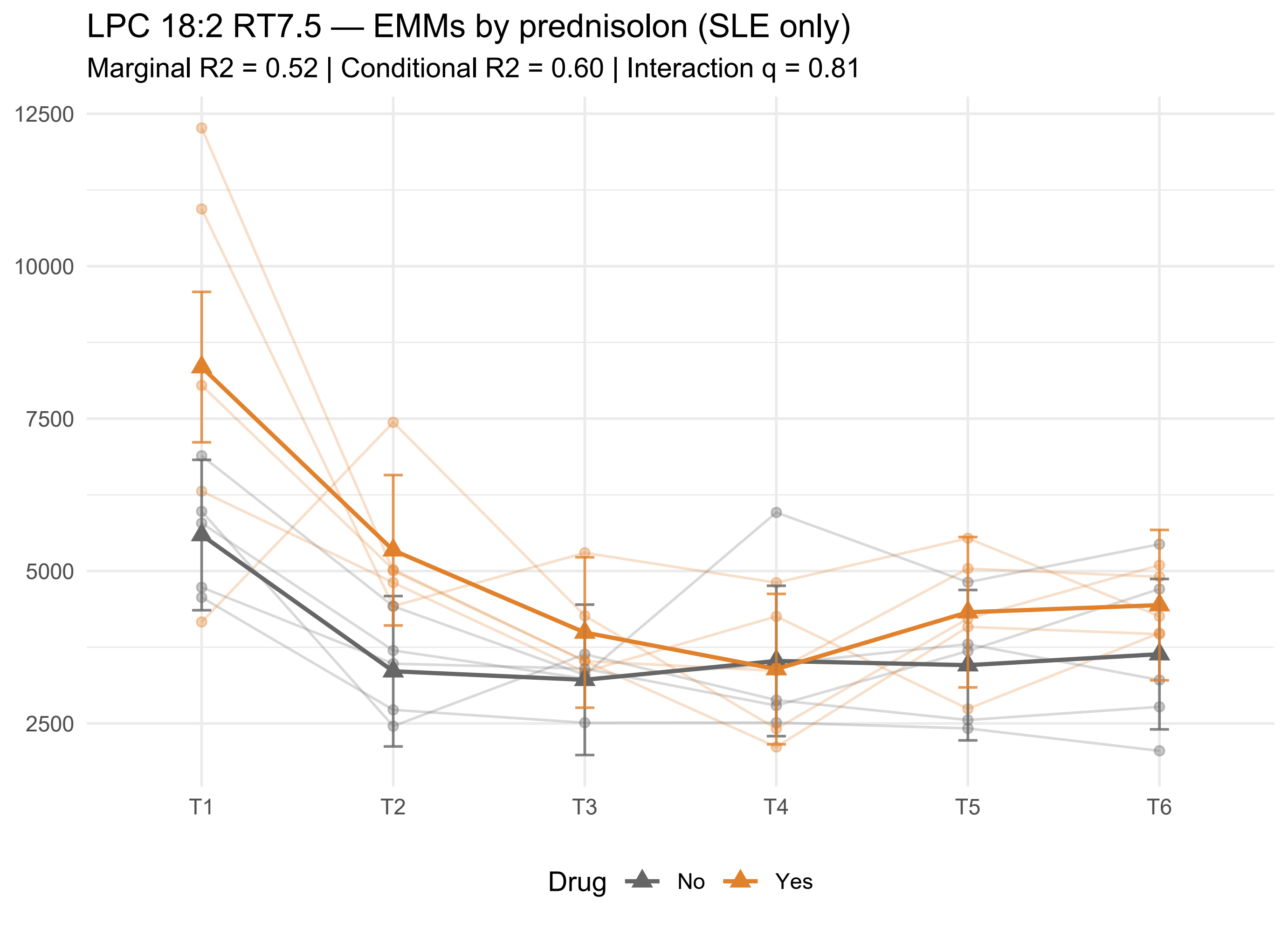
LPC 18:2 RT7.5 — EMMs by prednisolon (SLE only)
Marginal R2 = 0.52 | Conditional R2 = 0.60 | Interaction q = 0.81
12500
10000
7500
5000
2500
T1
T2
T3
T4
T5
T6
Drug
No
Yes

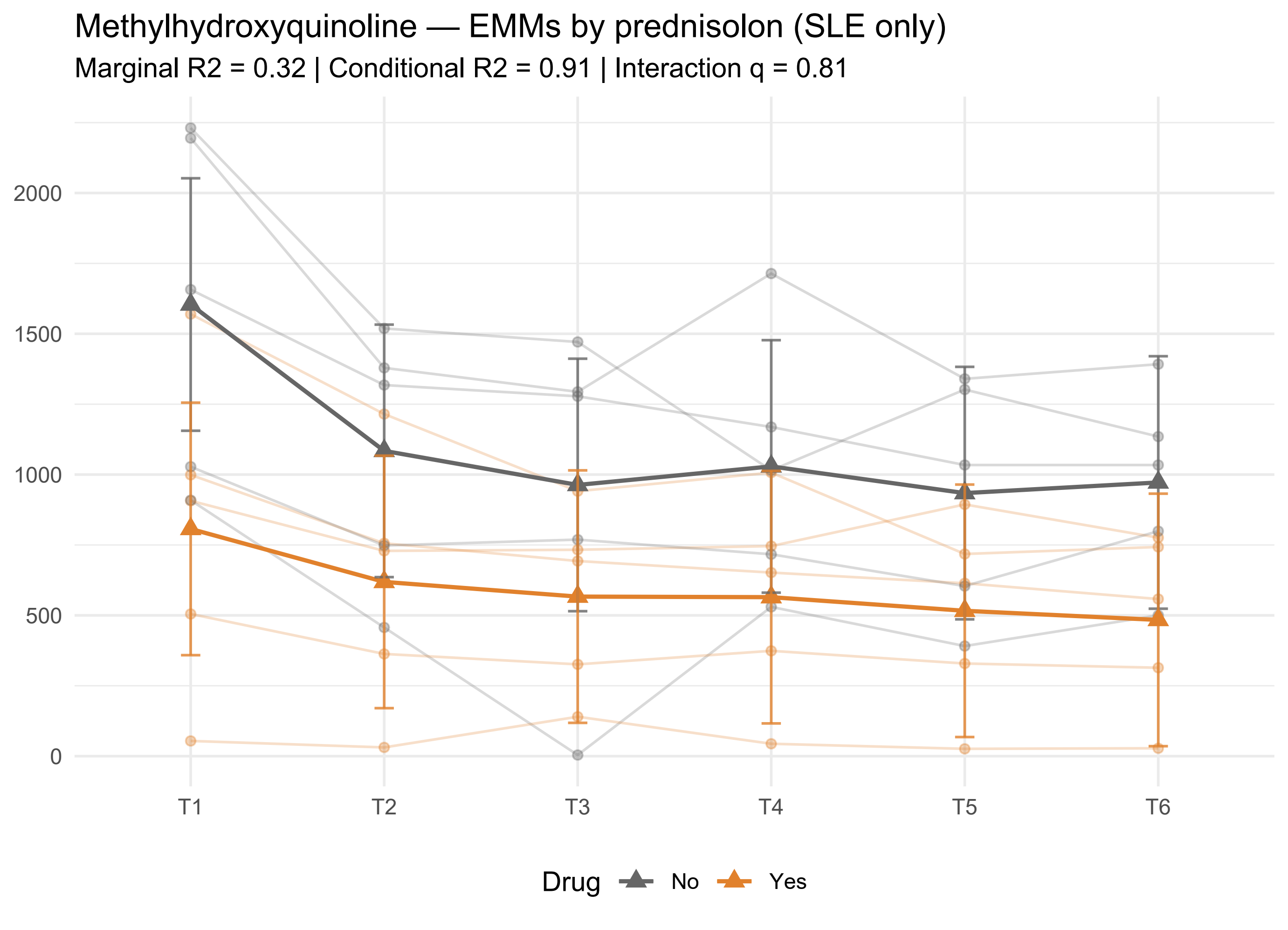
Methylhydroxyquinoline — EMMs by prednisolon (SLE only)
Marginal R2 = 0.32 | Conditional R2 = 0.91 | Interaction q = 0.81
2000
1500
1000
500
0
T1
T2
T3
T4
T5
T6
Drug
No
Yes

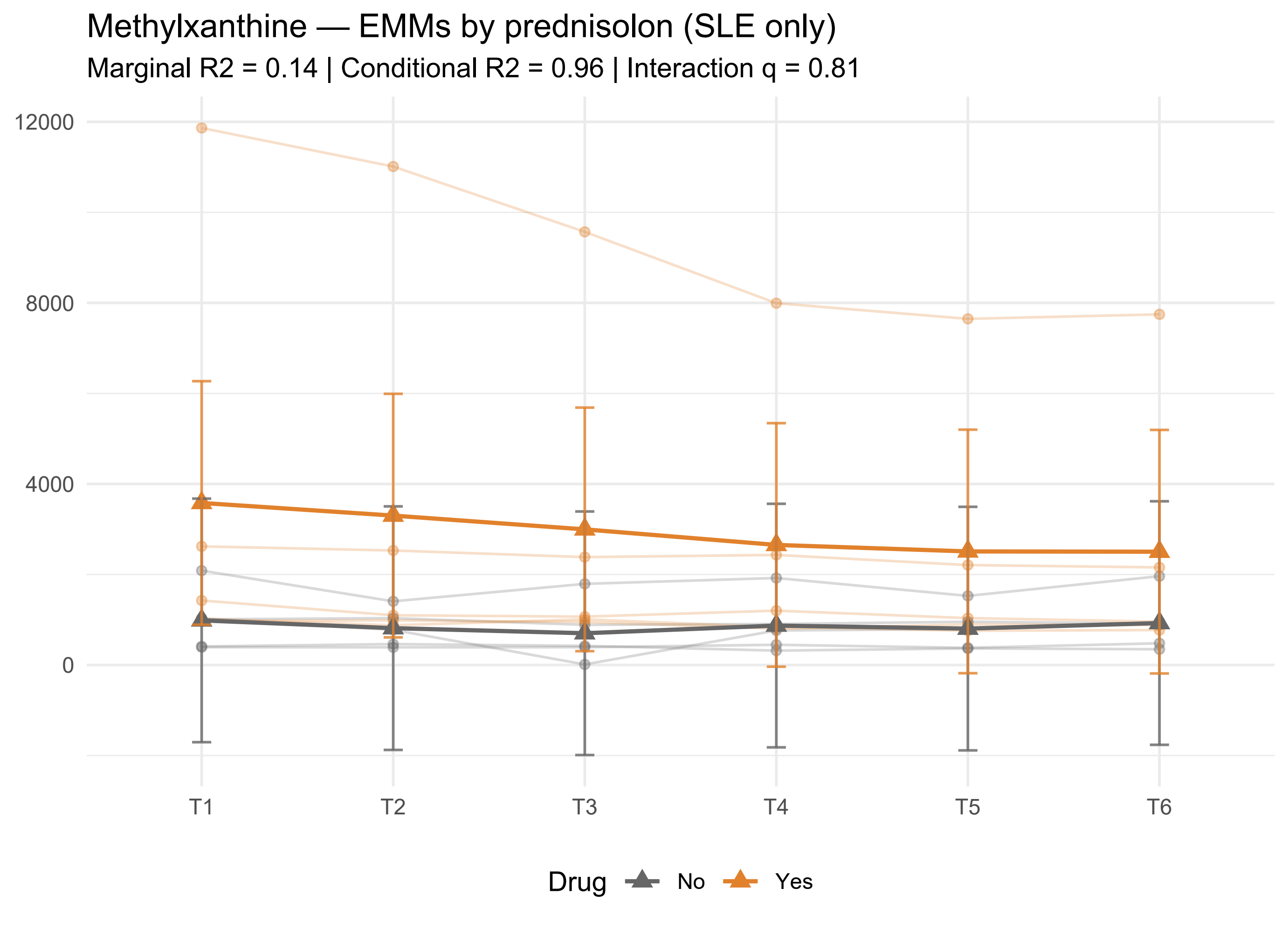

Methylxanthine — EMMs by prednisolon (SLE only)
Marginal R2 = 0.14 | Conditional R2 = 0.96 | Interaction q = 0.81
12000
8000
4000
0
T1
T2
T3
T4
T5
T6
Drug
No
Yes

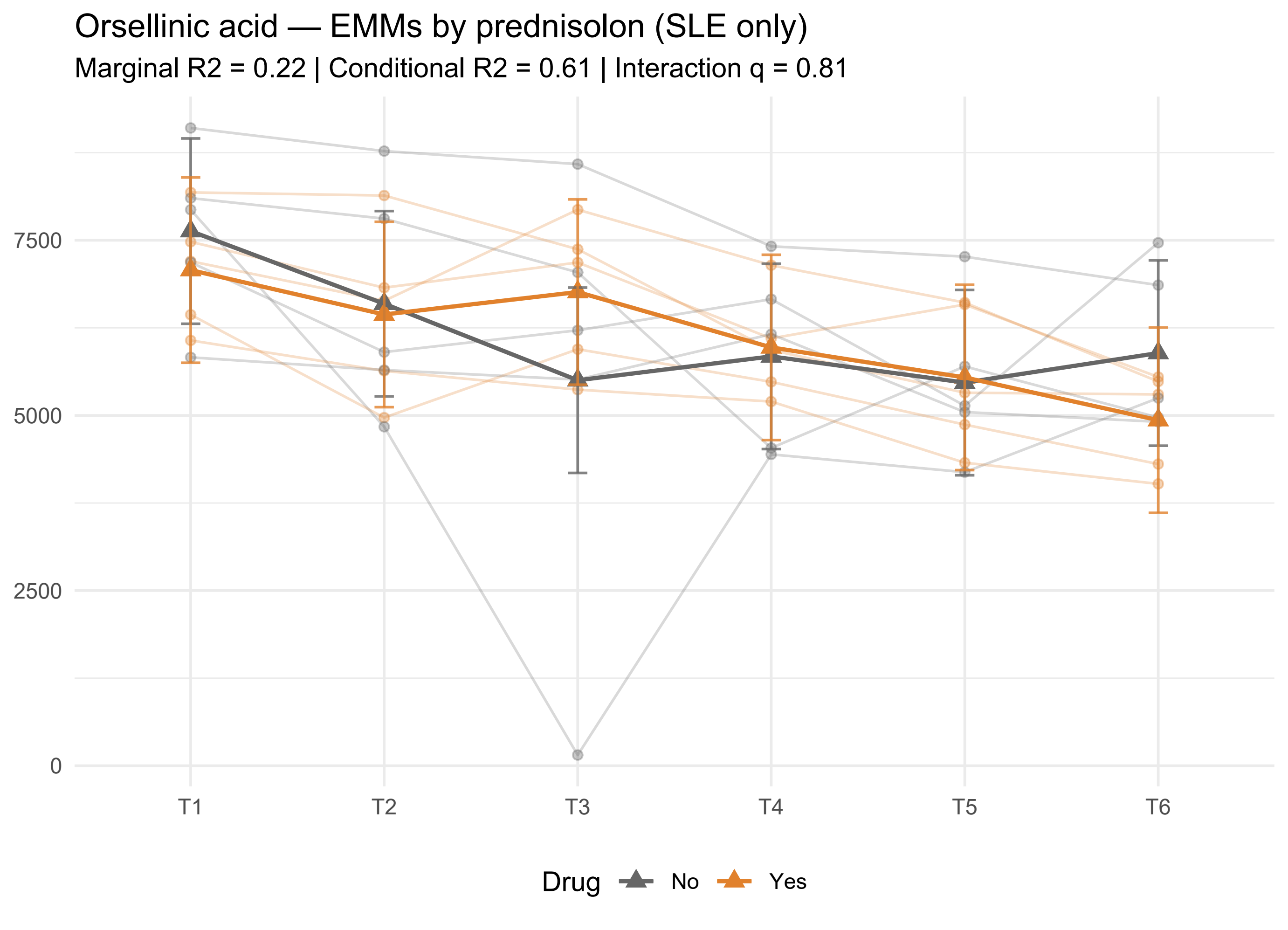

Orsellinic acid — EMMs by prednisolon (SLE only)
Marginal R2 = 0.22 | Conditional R2 = 0.61 | Interaction q = 0.81
7500
5000
2500
0
T1
T2
T3
T4
T5
T6
Drug
No
Yes

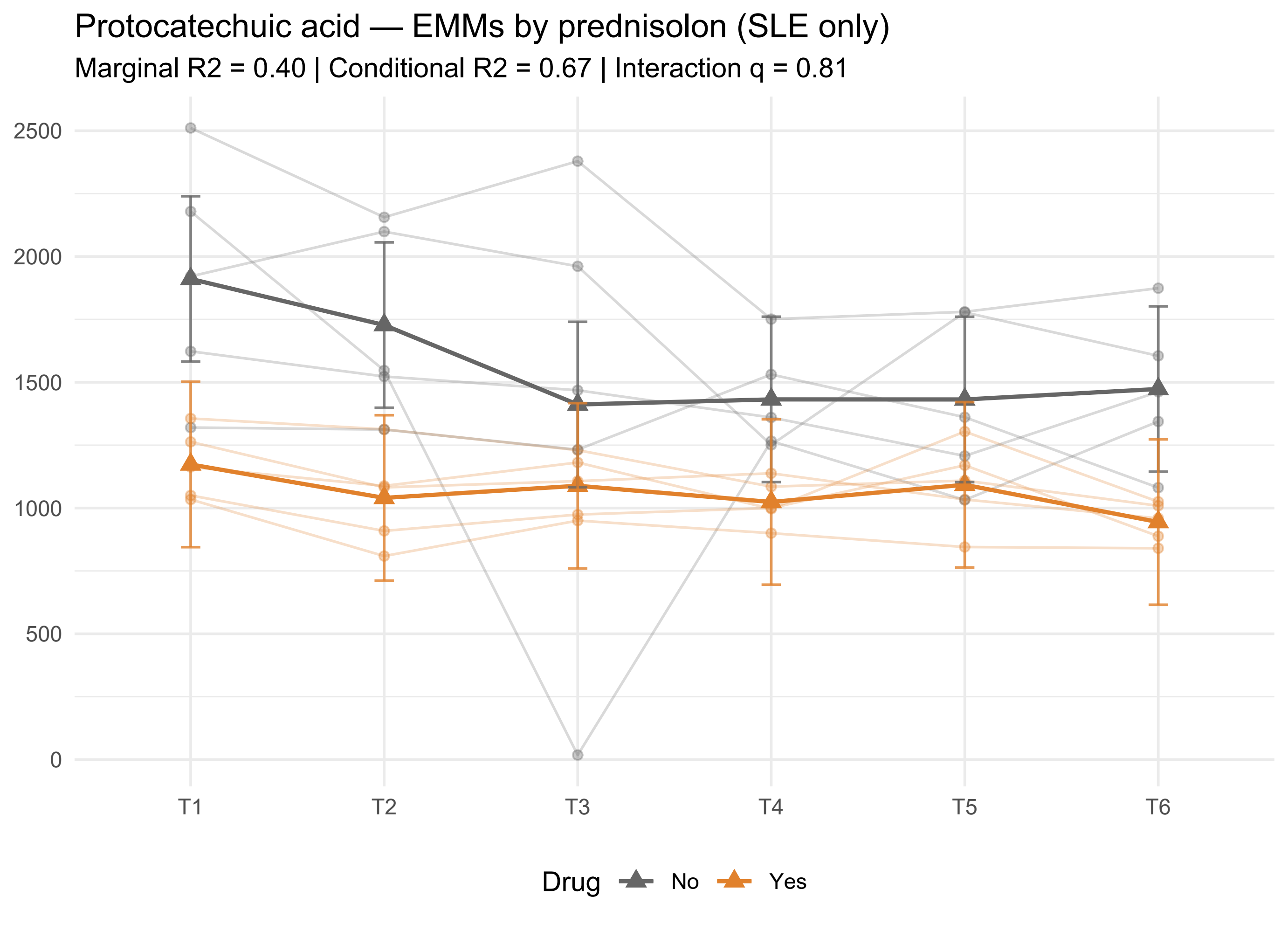

Protocatechuic acid — EMMs by prednisolon (SLE only)
Marginal R2 = 0.40 | Conditional R2 = 0.67 | Interaction q = 0.81
2500
2000
1500
1000
500
0
T1
T2
T3
T4
T5
T6
Drug
No
Yes

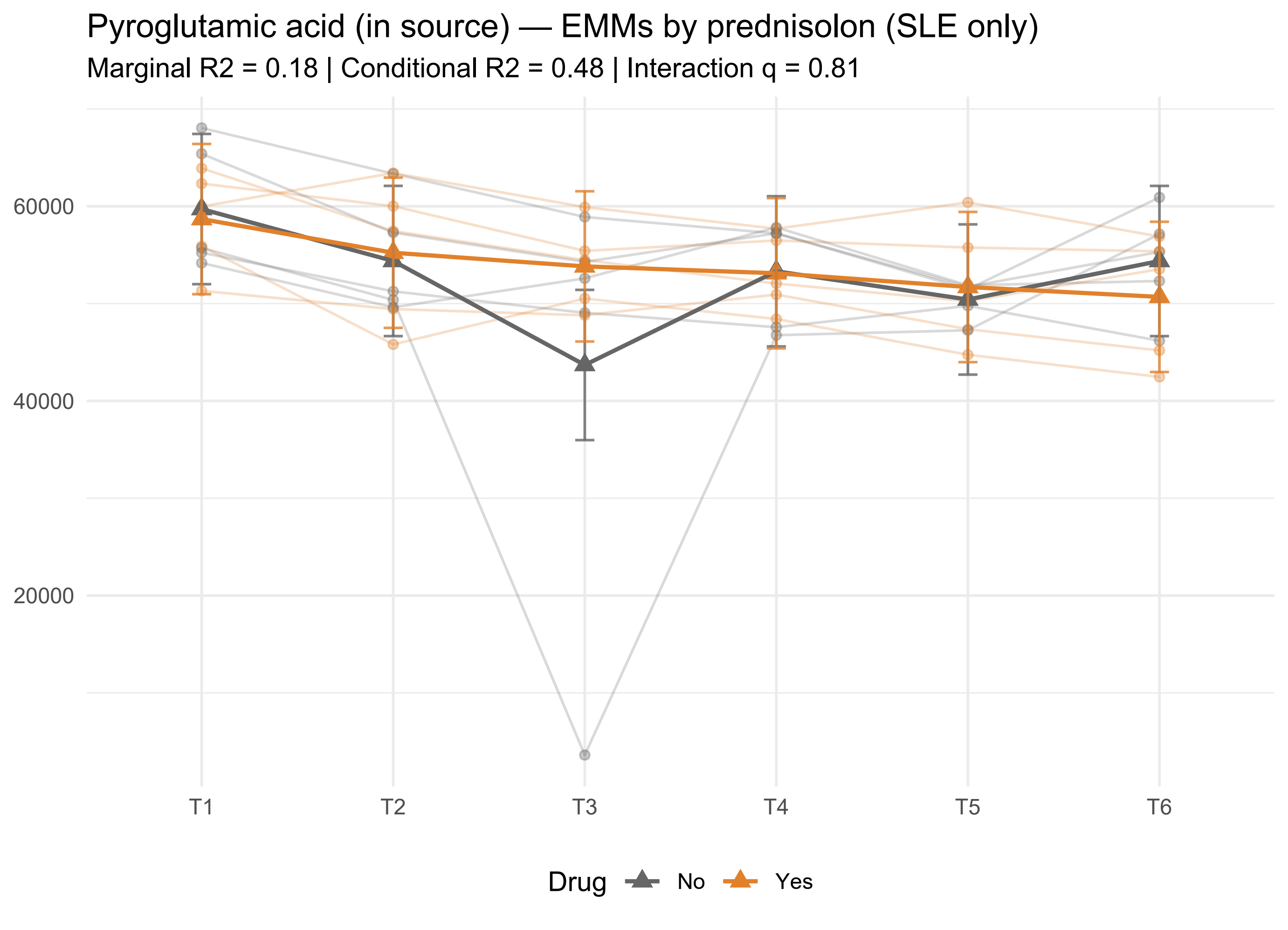
Pyroglutamic acid (in source) — EMMs by prednisolon (SLE only)
Marginal R2 = 0.18 | Conditional R2 = 0.48 | Interaction q = 0.81
60000
40000
20000
T1
T2
T3
T4
T5
T6
Drug
No
Yes

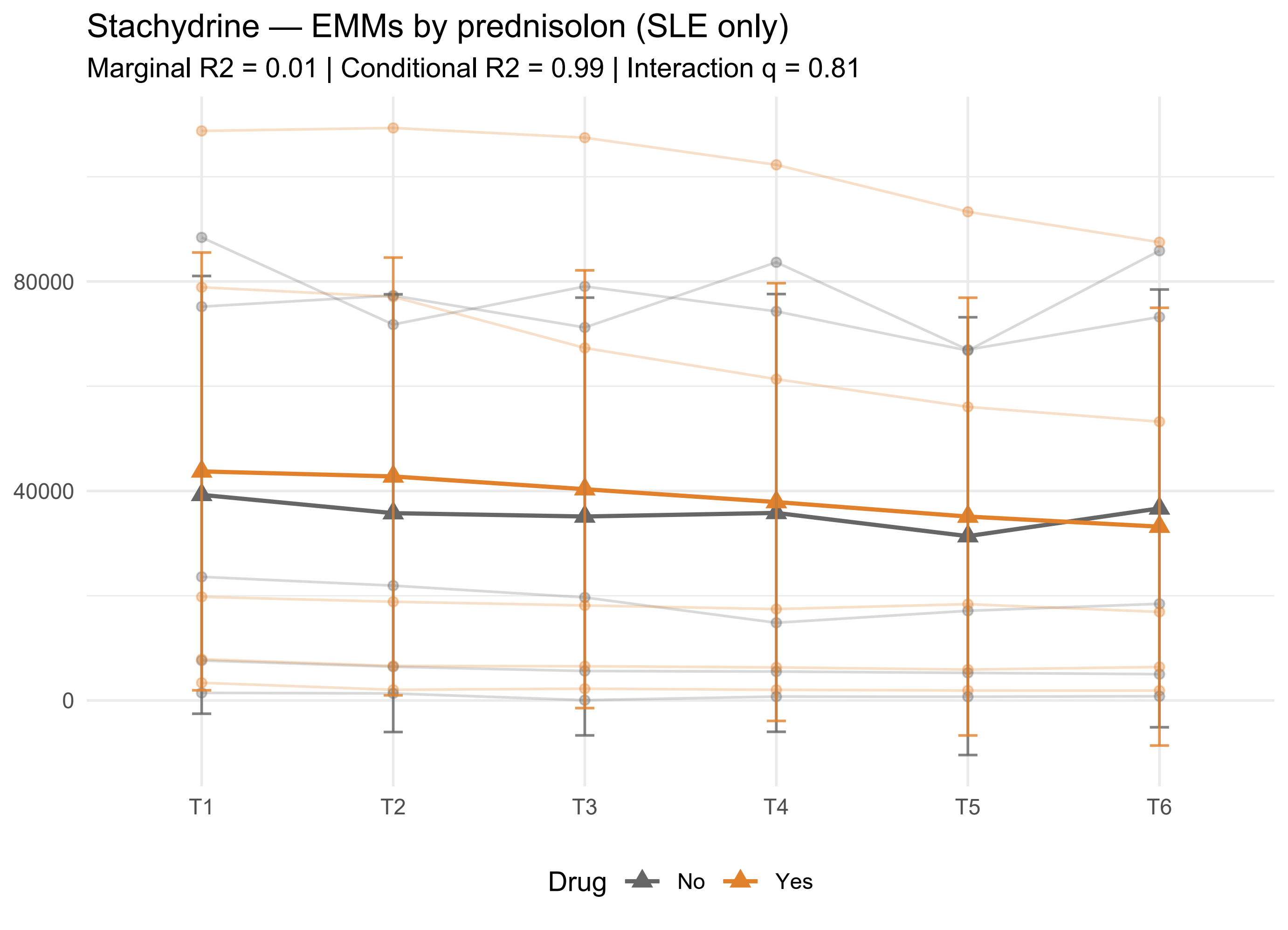

Stachydrine — EMMs by prednisolon (SLE only)
Marginal R2 = 0.01 | Conditional R2 = 0.99 | Interaction q = 0.81
80000
40000
0
T1
T2
T3
T4
T5
T6
Drug
No
Yes

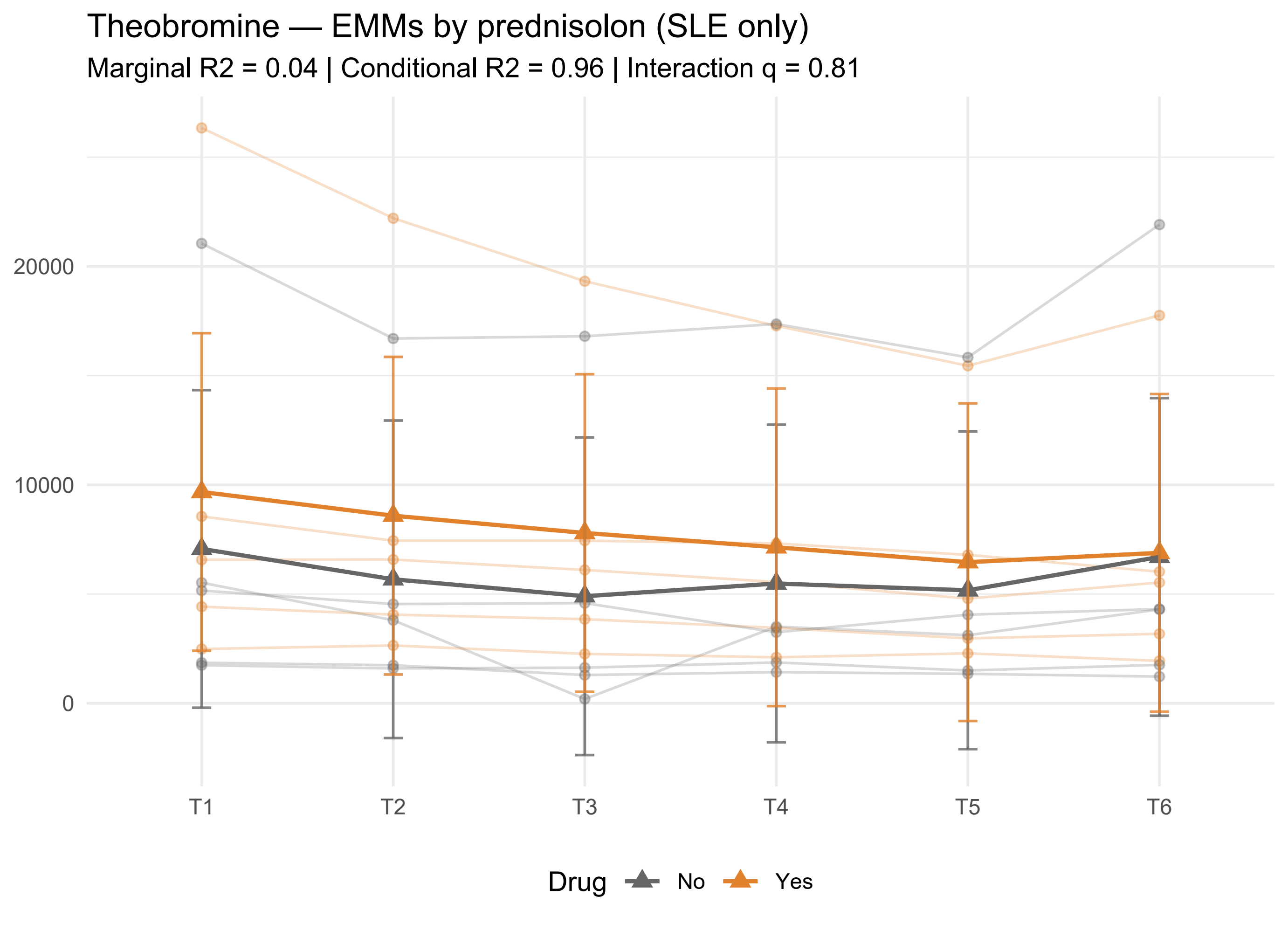

Theobromine — EMMs by prednisolon (SLE only)
Marginal R2 = 0.04 | Conditional R2 = 0.96 | Interaction q = 0.81
20000
10000
0
T1
T2
T3
T4
T5
T6
Drug
No
Yes

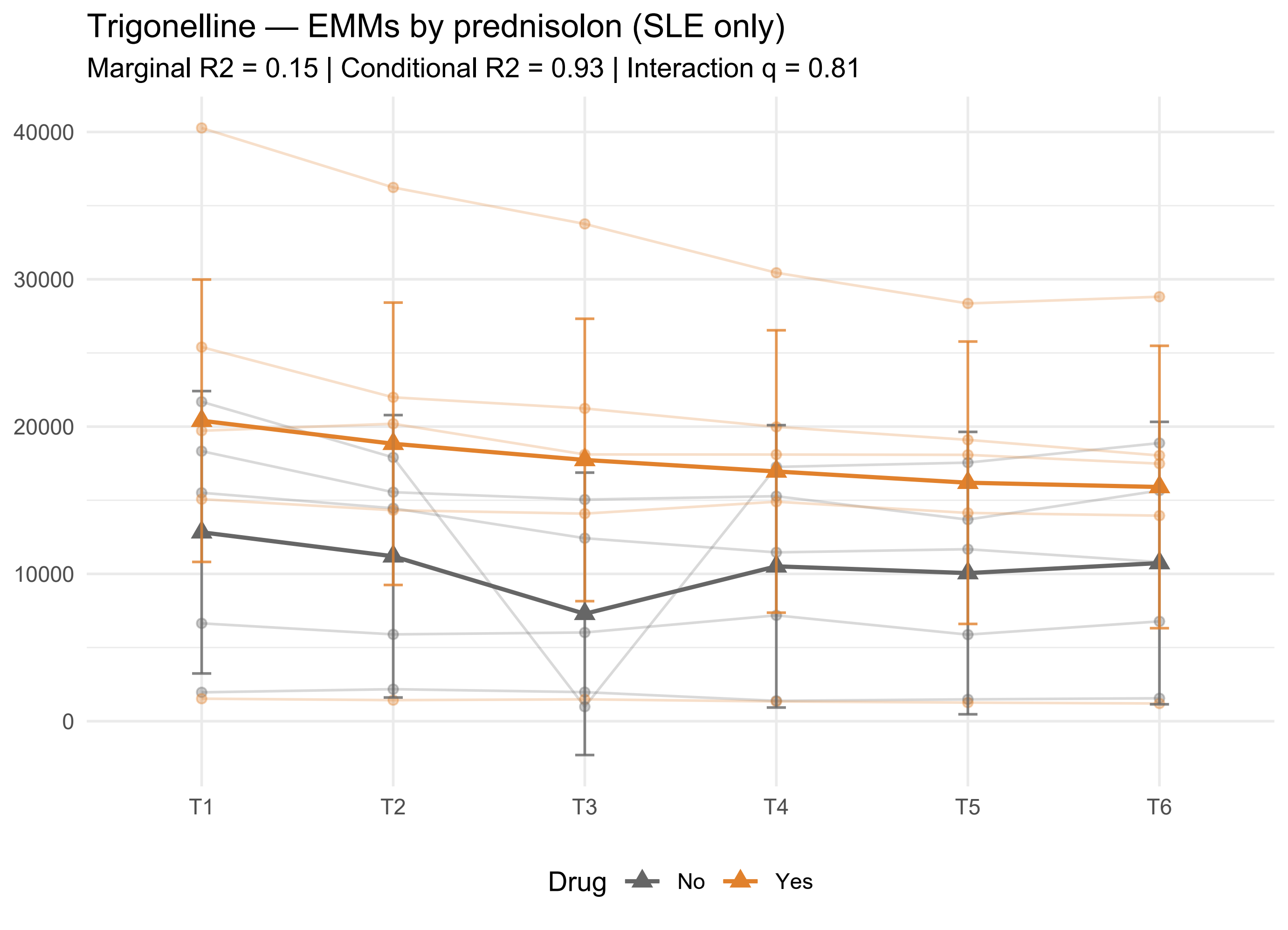

Trigonelline — EMMs by prednisolon (SLE only)
Marginal R2 = 0.15 | Conditional R2 = 0.93 | Interaction q = 0.81
40000
30000
20000
10000
0
T1
T2
T3
T4
T5
T6
Drug
No
Yes

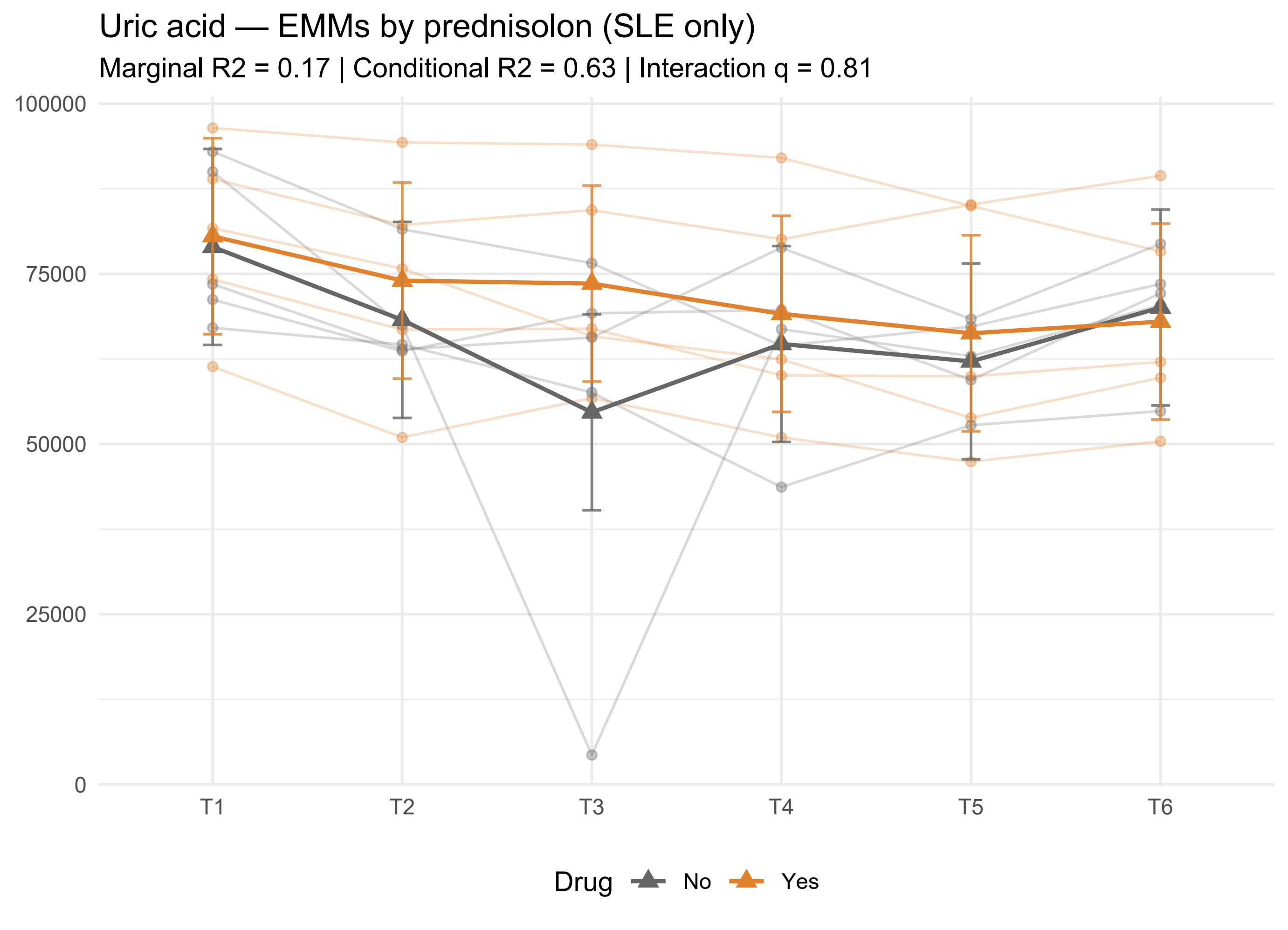

Uric acid — EMMs by prednisolon (SLE only)
Marginal R2 = 0.17 | Conditional R2 = 0.63 | Interaction q = 0.81
100000
75000
50000
25000
0
T1
T2
T3
T4
T5
T6
Drug
No
Yes

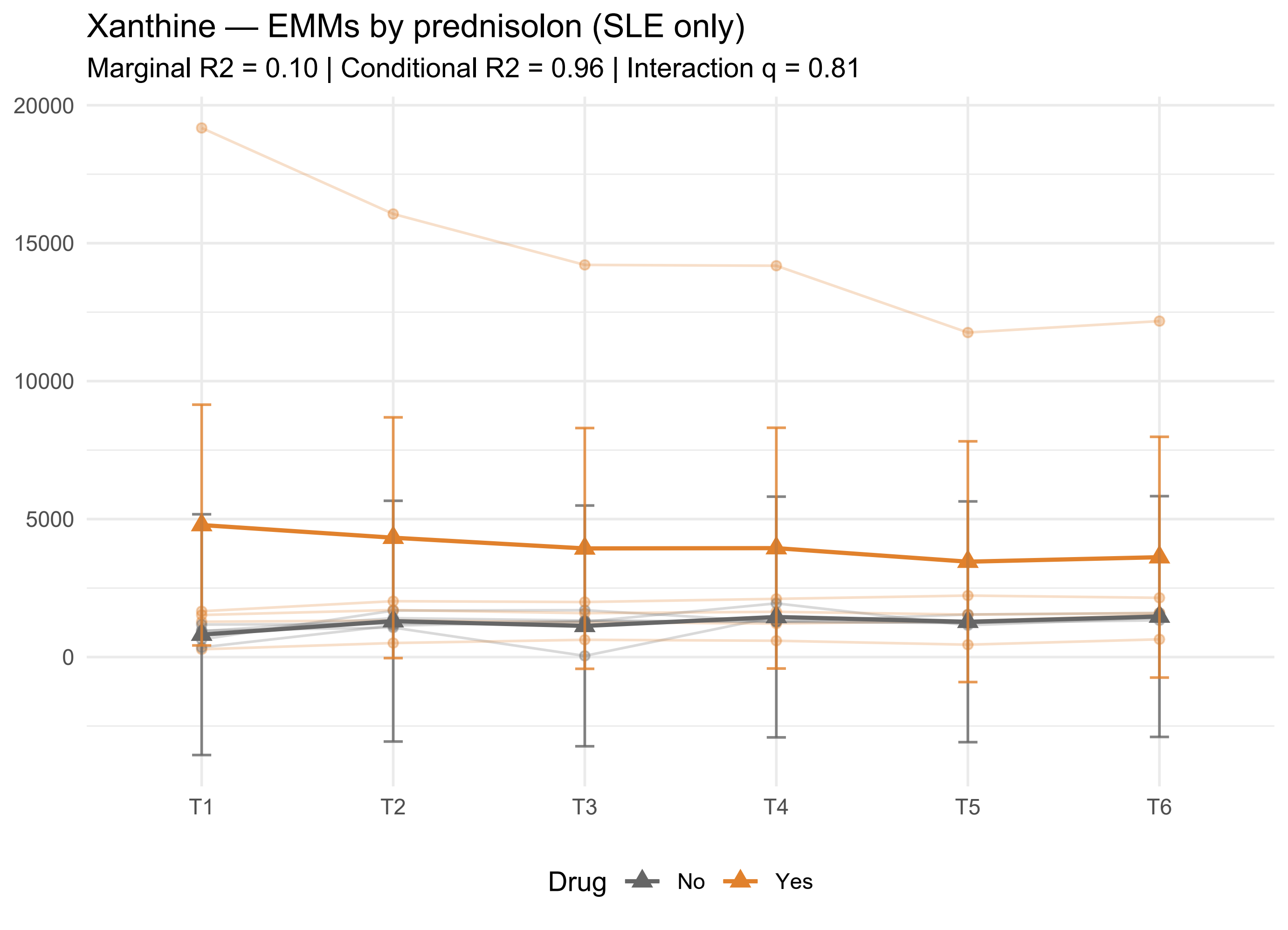

Xanthine — EMMs by prednisolon (SLE only)
Marginal R2 = 0.10 | Conditional R2 = 0.96 | Interaction q = 0.81
20000
15000
10000
5000
0
T1
T2
T3
T4
T5
T6
Drug
No
Yes

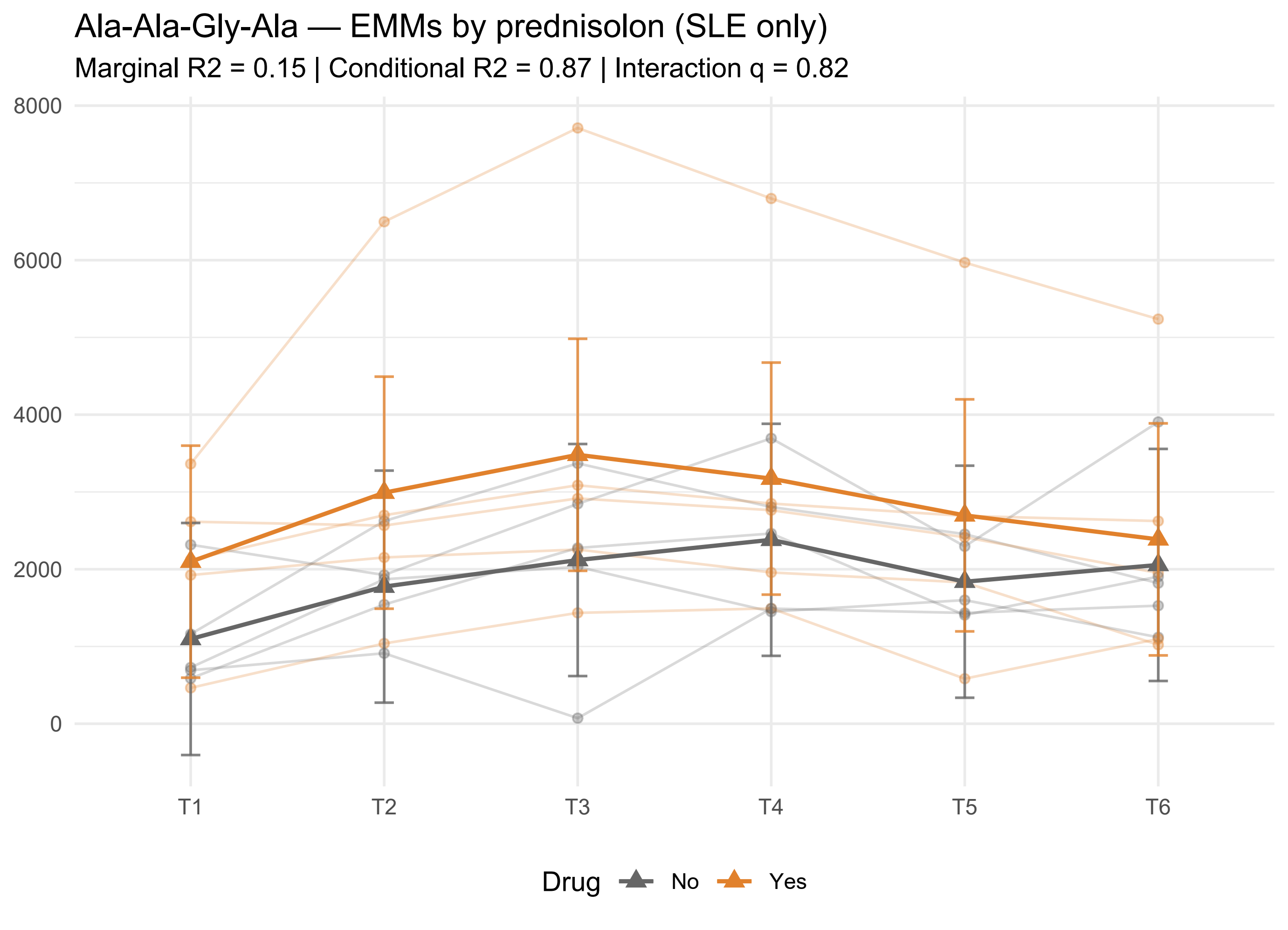

Ala-Ala-Gly-Ala — EMMs by prednisolon (SLE only)
Marginal R2 = 0.15 | Conditional R2 = 0.87 | Interaction q = 0.82
8000
6000
4000
2000
0
T1
T2
T3
T4
T5
T6
Drug
No
Yes

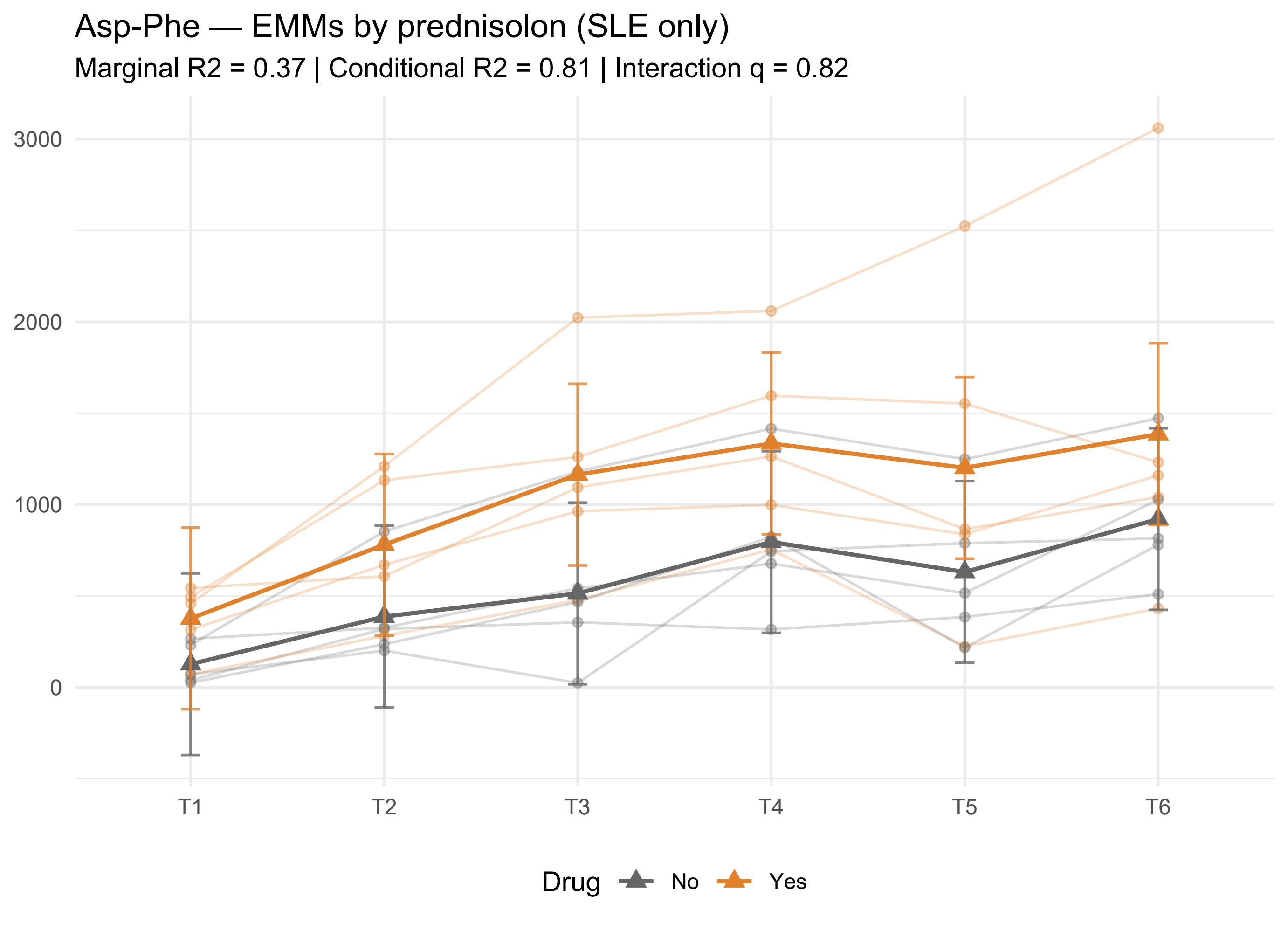

Asp-Phe — EMMs by prednisolon (SLE only)
Marginal R2 = 0.37 | Conditional R2 = 0.81 | Interaction q = 0.82
3000
2000
1000
0
T1
T2
T3
T4
T5
T6
Drug
No
Yes

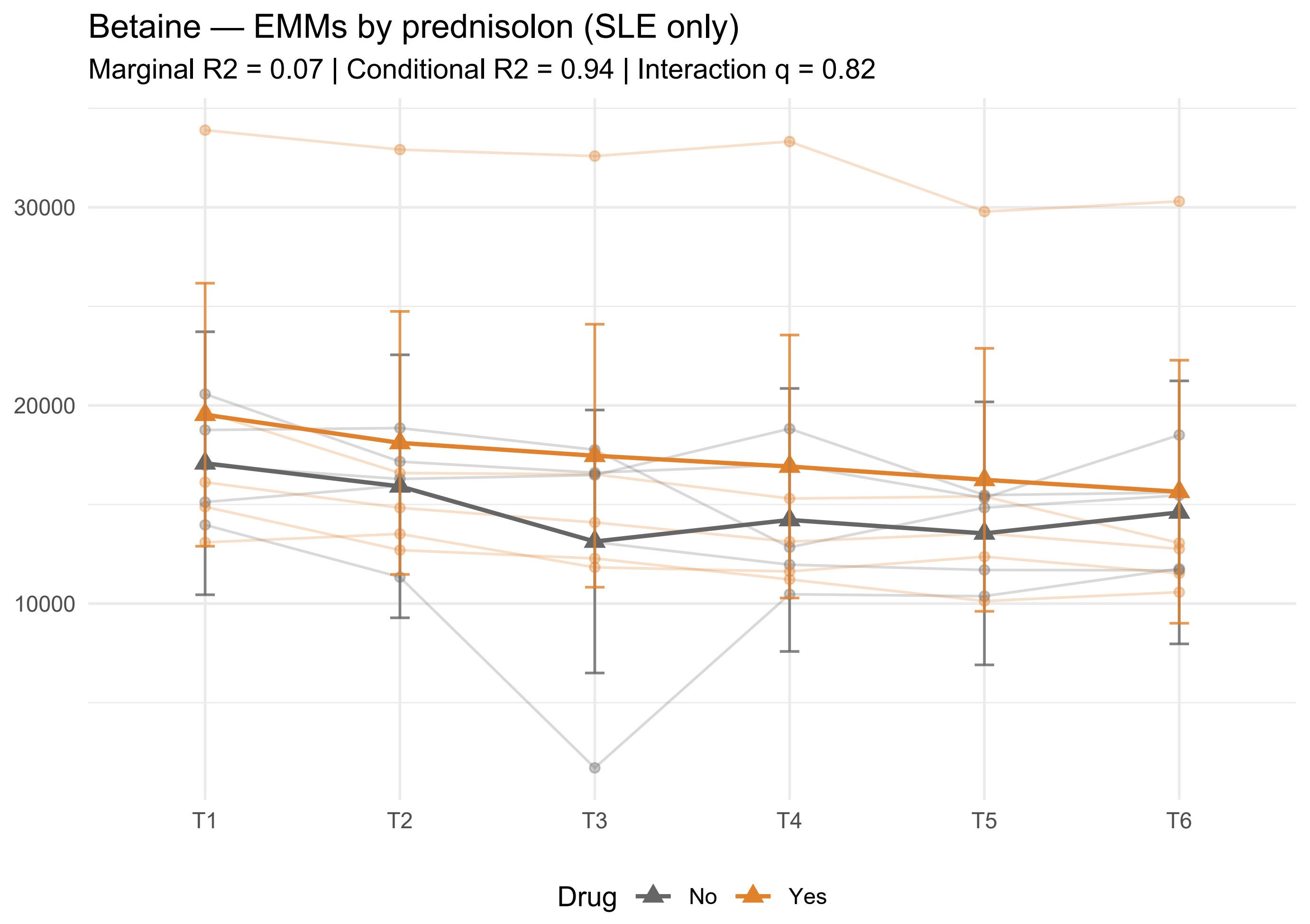
Betaine — EMMs by prednisolon (SLE only)
Marginal R2 = 0.07 | Conditional R2 = 0.94 | Interaction q = 0.82
30000
20000
10000
T1
T2
T3
T4
T5
T6
Drug
No
Yes

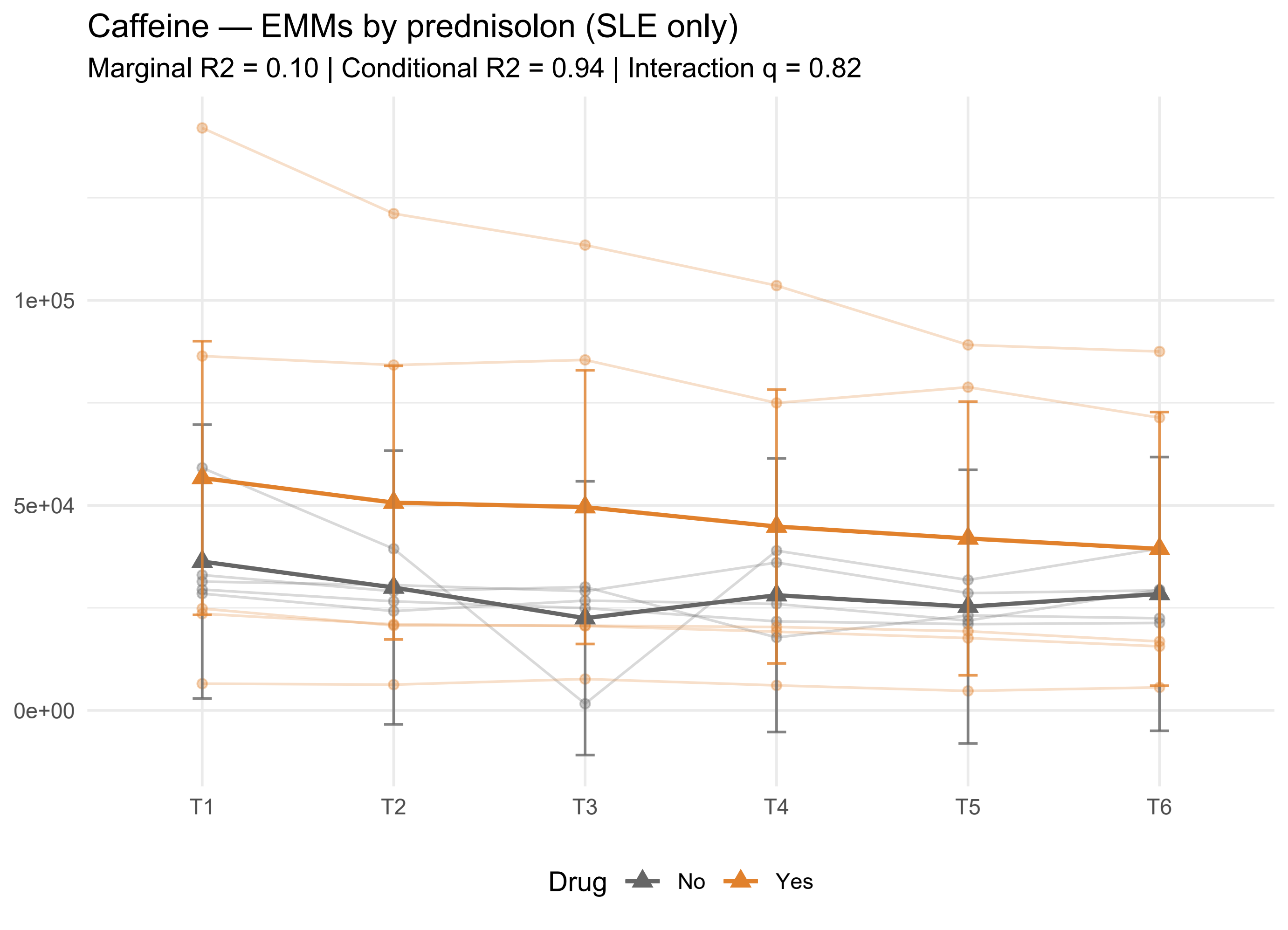

Caffeine — EMMs by prednisolon (SLE only)
Marginal R2 = 0.10 | Conditional R2 = 0.94 | Interaction q = 0.82
1e+05
5e+04
0e+00
T1
T2
T3
T4
T5
T6
Drug
No
Yes

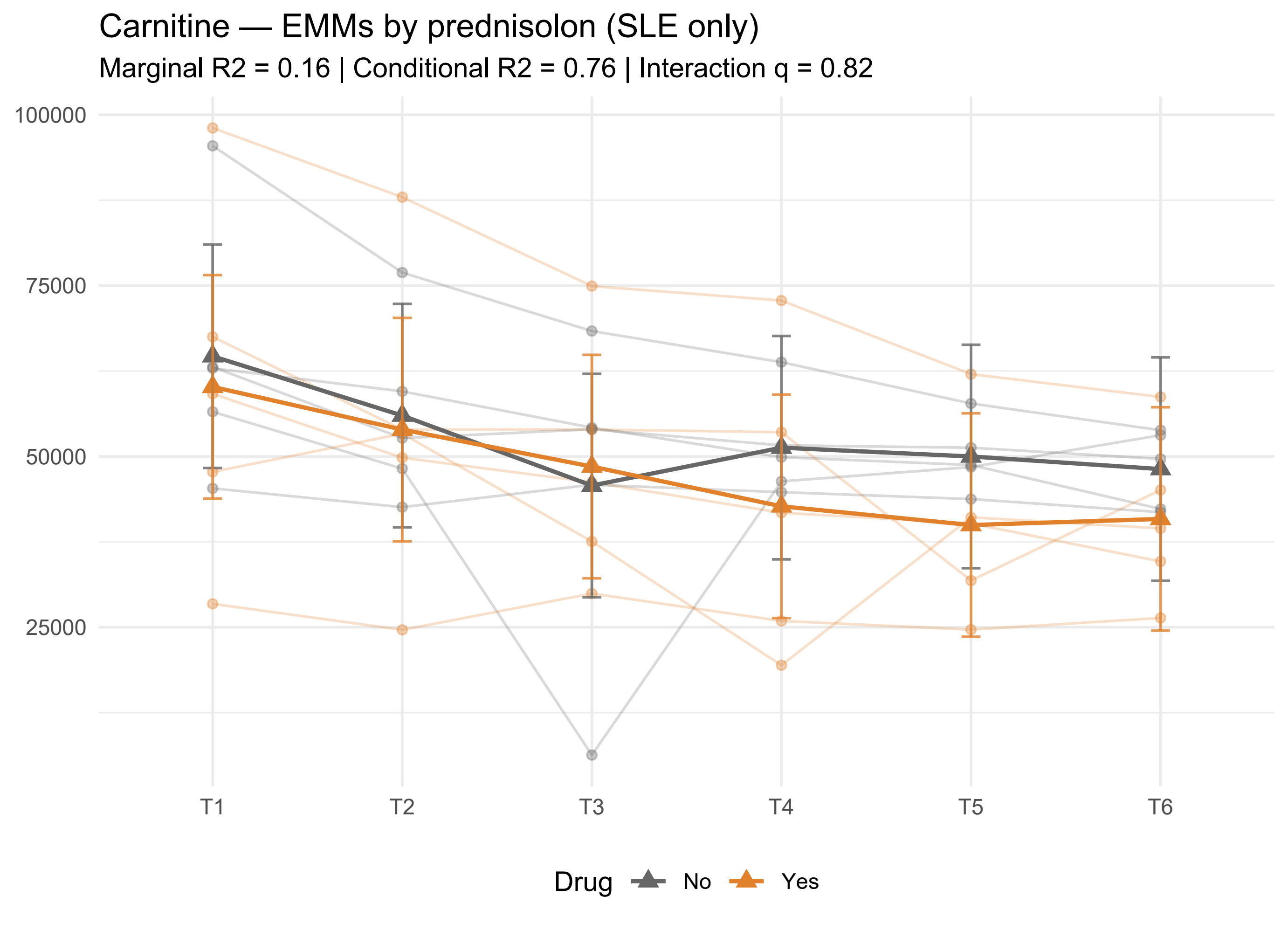

Carnitine — EMMs by prednisolon (SLE only)
Marginal R2 = 0.16 | Conditional R2 = 0.76 | Interaction q = 0.82
100000
75000
50000
25000
T1
T2
T3
T4
T5
T6
Drug
No
Yes

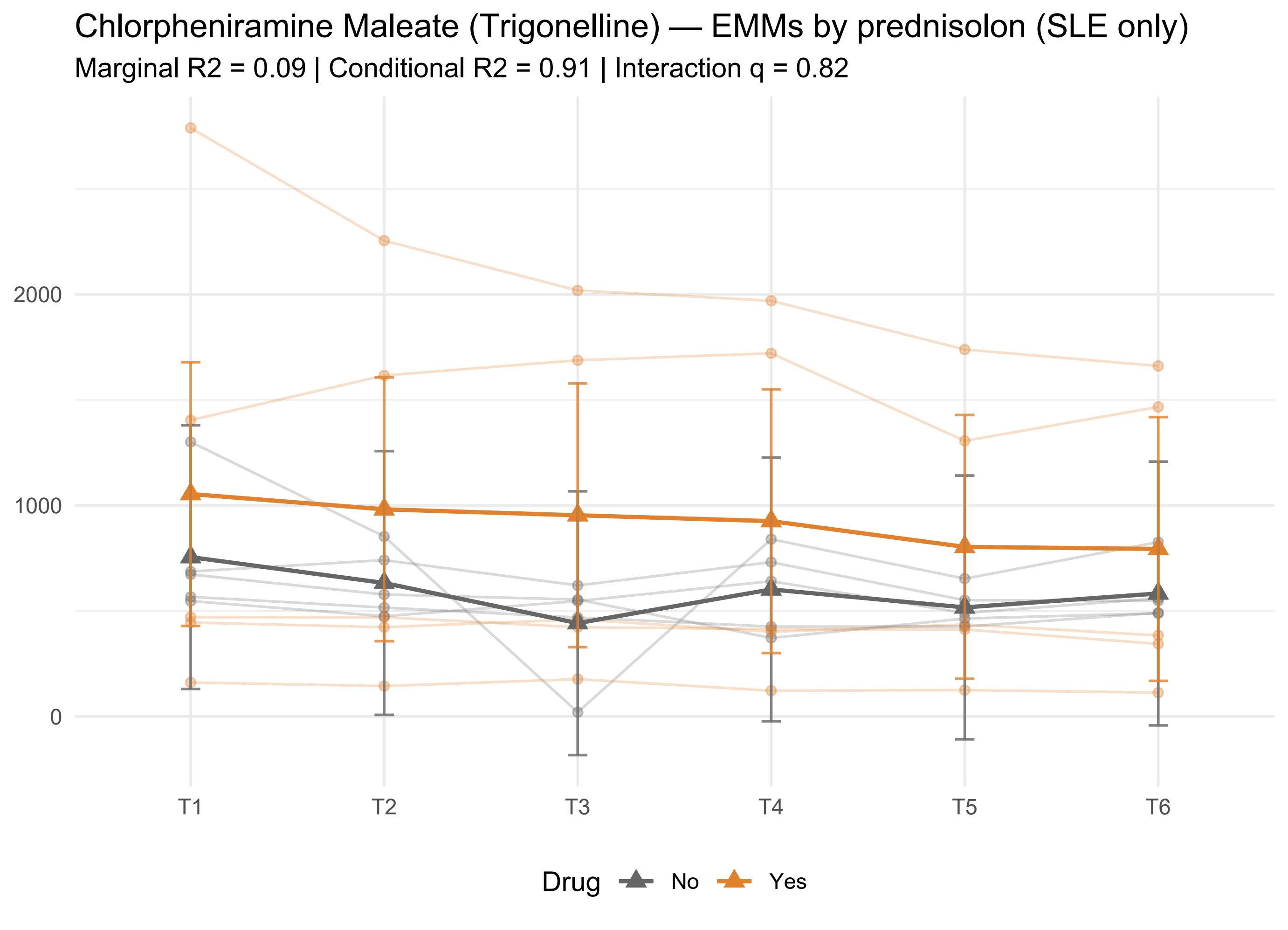

Chlorpheniramine Maleate (Trigonelline) — EMMs by prednisolon (SLE only)
Marginal R2 = 0.09 | Conditional R2 = 0.91 | Interaction q = 0.82
2000
1000
0
T1
T2
T3
T4
T5
T6
Drug
No
Yes

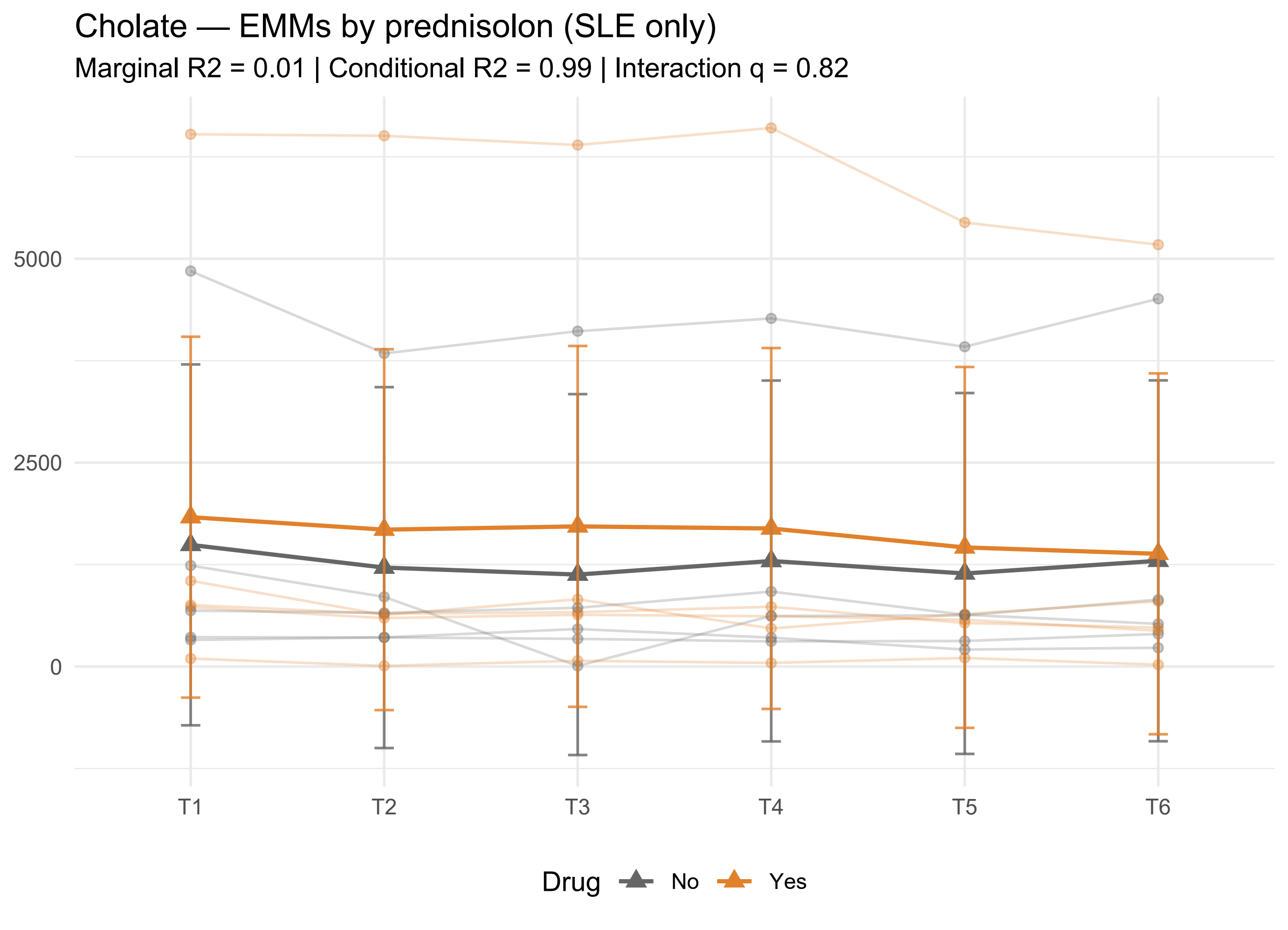
Cholate — EMMs by prednisolon (SLE only)
Marginal R2 = 0.01 | Conditional R2 = 0.99 | Interaction q = 0.82
5000
2500
0
T1
T2
T3
T4
T5
T6
Drug
No
Yes

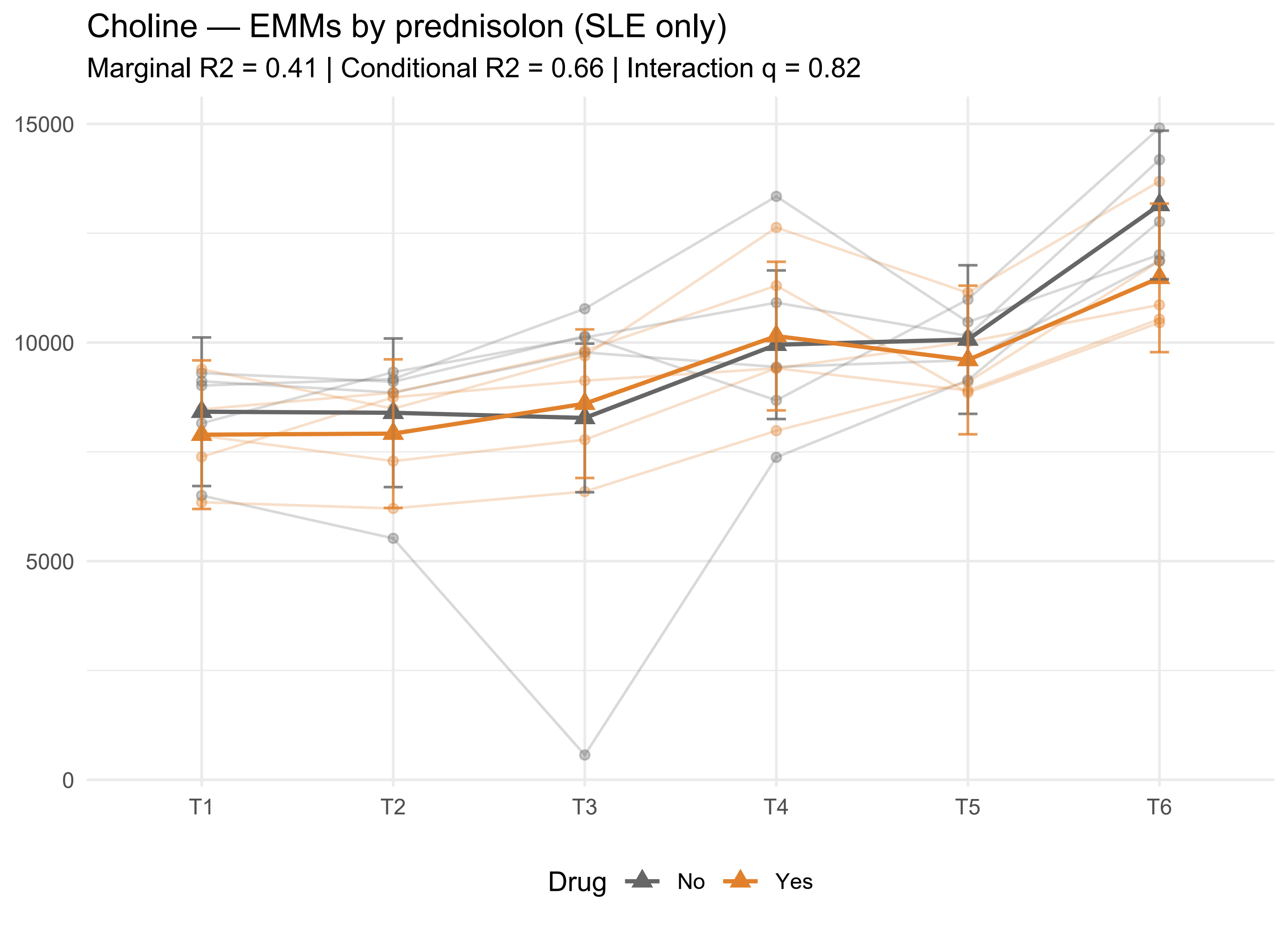

Choline — EMMs by prednisolon (SLE only)
Marginal R2 = 0.41 | Conditional R2 = 0.66 | Interaction q = 0.82
15000
10000
5000
0
T1
T2
T3
T4
T5
T6
Drug
No
Yes

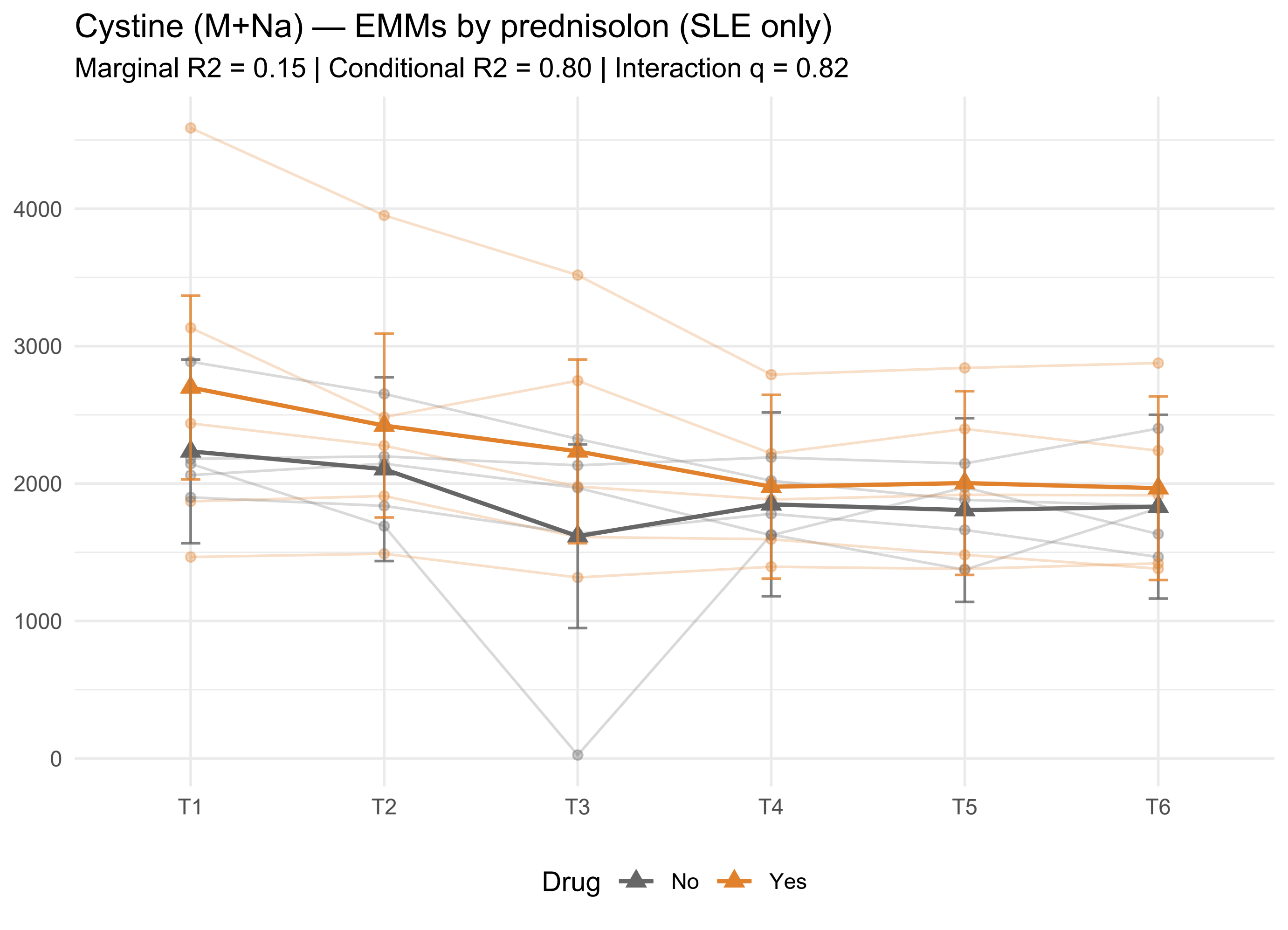

Cystine (M+Na) — EMMs by prednisolon (SLE only)
Marginal R2 = 0.15 | Conditional R2 = 0.80 | Interaction q = 0.82
4000
3000
2000
1000
0
T1
T2
T3
T4
T5
T6
Drug
No
Yes

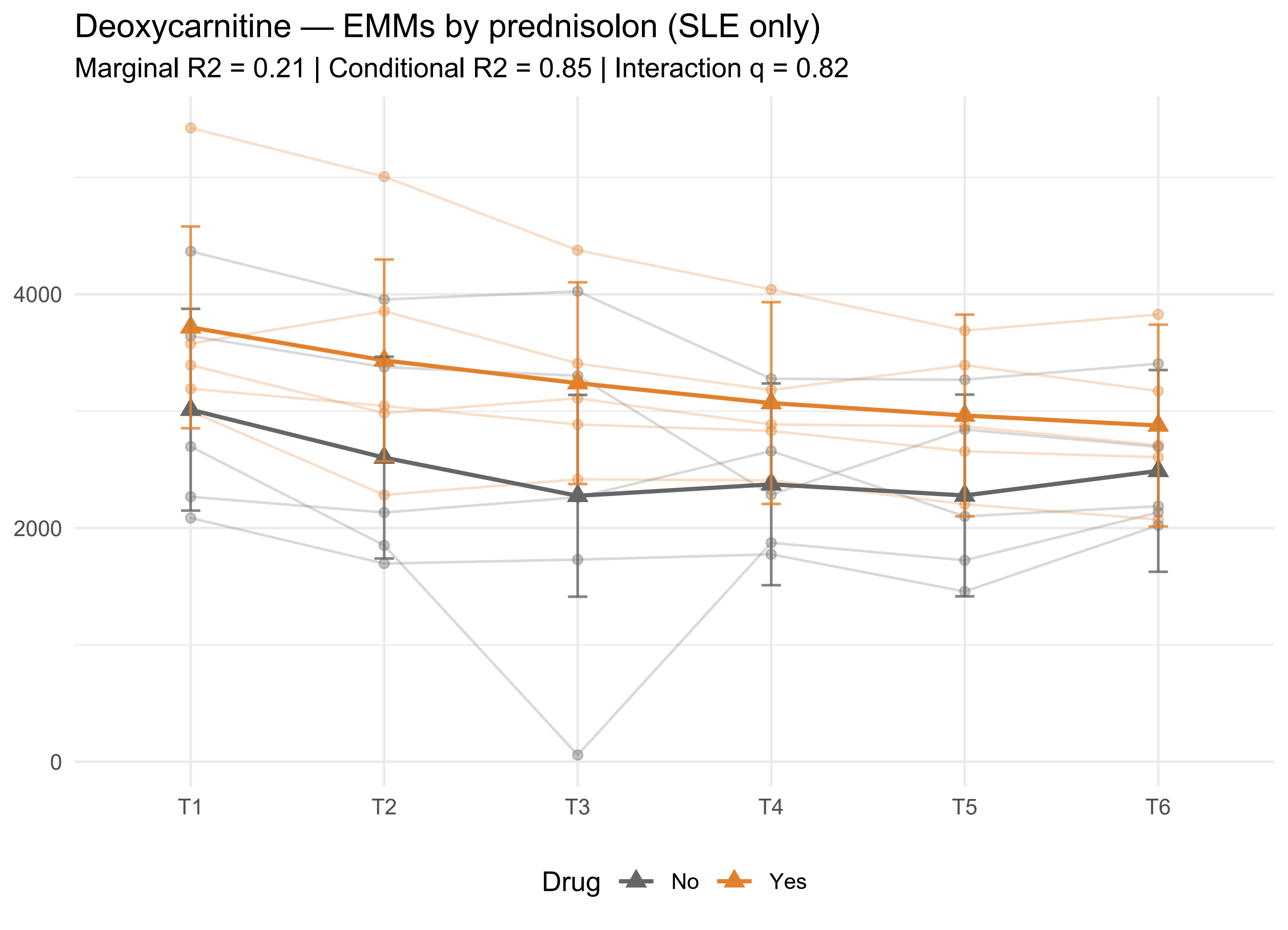
Deoxycarnitine — EMMs by prednisolon (SLE only)
Marginal R2 = 0.21 | Conditional R2 = 0.85 | Interaction q = 0.82
4000
2000
0
T1
T2
T3
T4
T5
T6
Drug
No
Yes

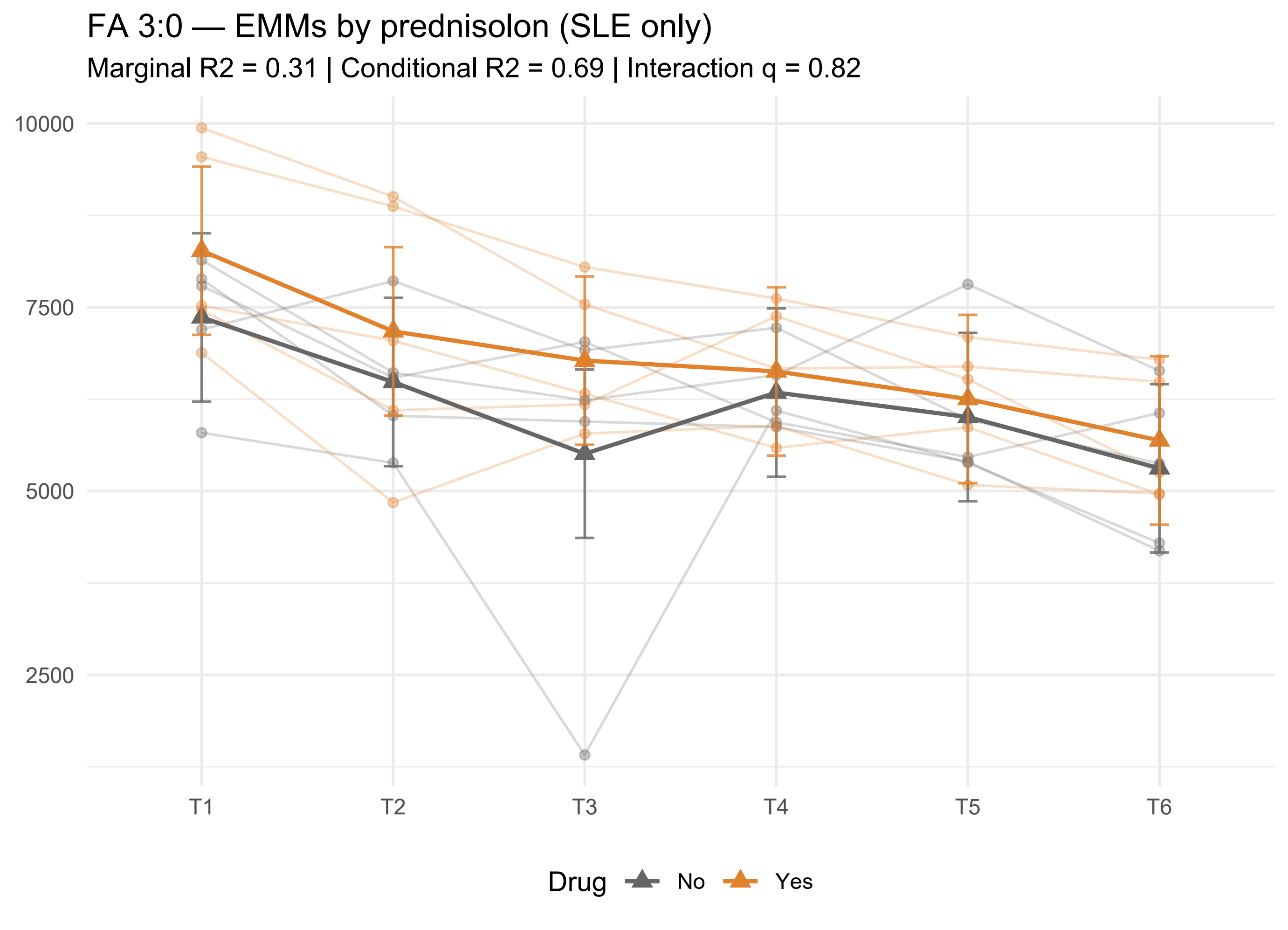

FA 3:0 — EMMs by prednisolon (SLE only)
Marginal R2 = 0.31 | Conditional R2 = 0.69 | Interaction q = 0.82
10000
7500
5000
2500
T1
T2
T3
T4
T5
T6
Drug
No
Yes

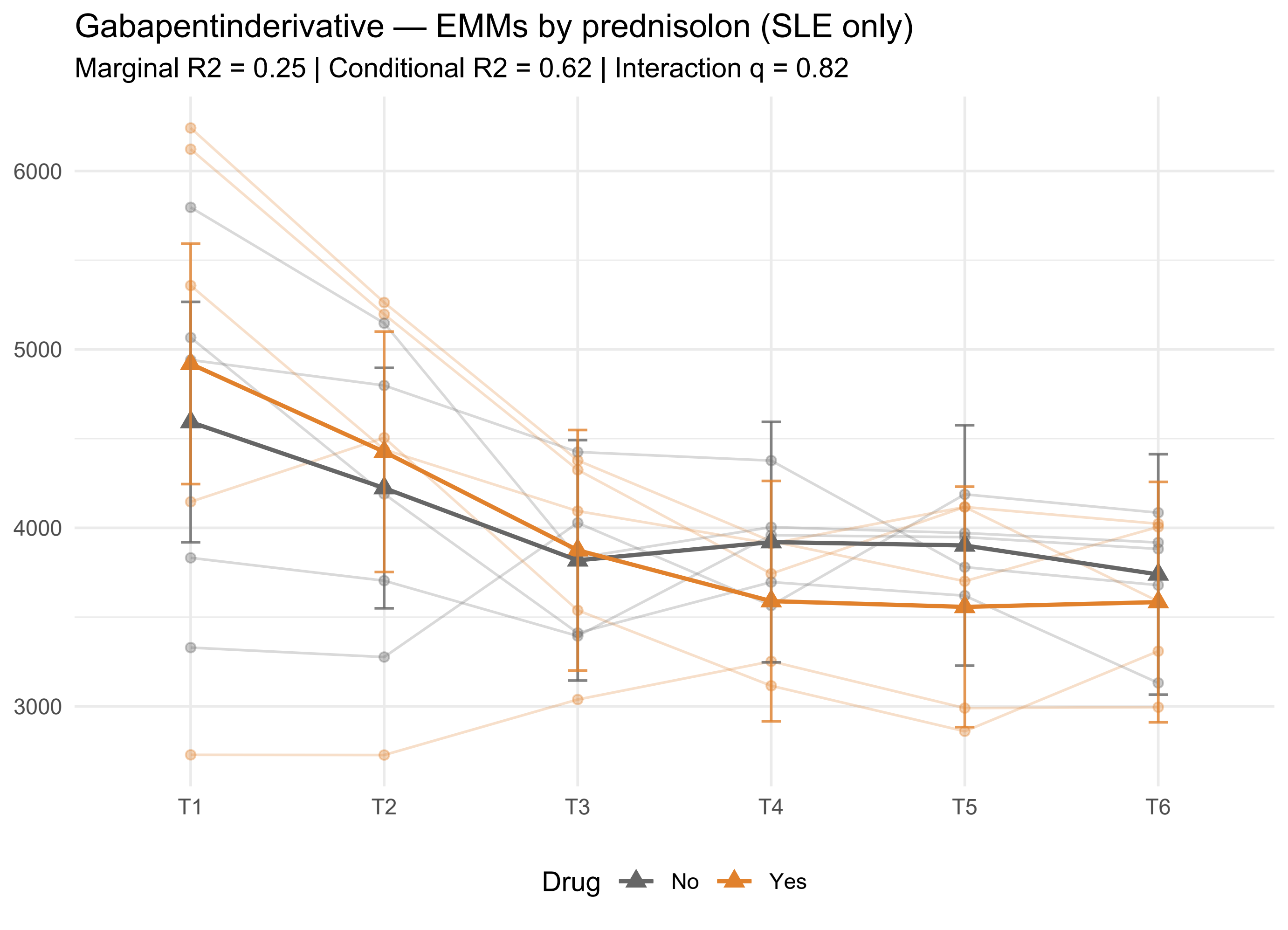

Gabapentinderivative — EMMs by prednisolon (SLE only)
Marginal R2 = 0.25 | Conditional R2 = 0.62 | Interaction q = 0.82
6000
5000
4000
3000
T1
T2
T3
T4
T5
T6
Drug
No
Yes

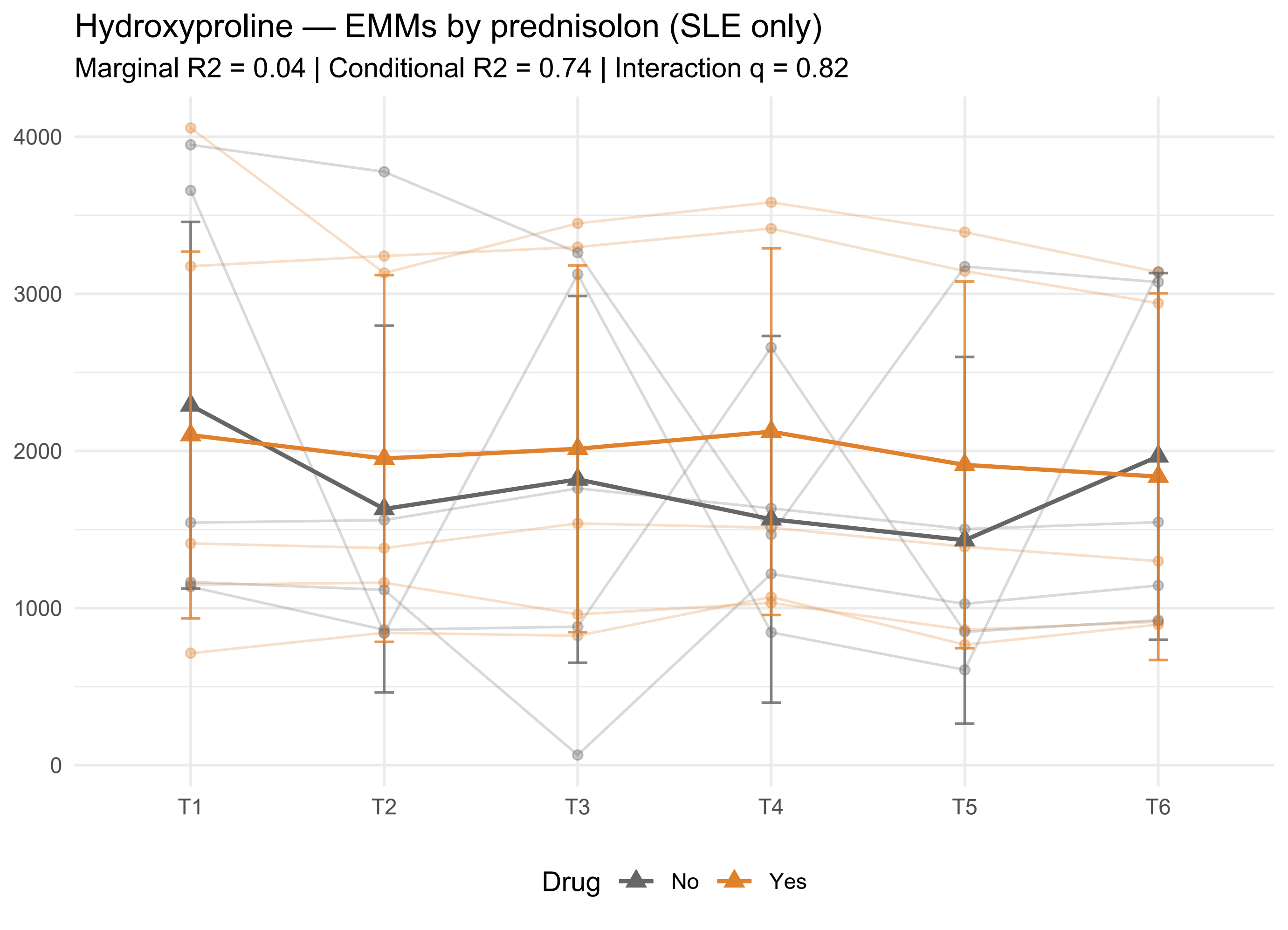

Hydroxyproline — EMMs by prednisolon (SLE only)
Marginal R2 = 0.04 | Conditional R2 = 0.74 | Interaction q = 0.82
4000
3000
2000
1000
0
T1
T2
T3
T4
T5
T6
Drug
No
Yes

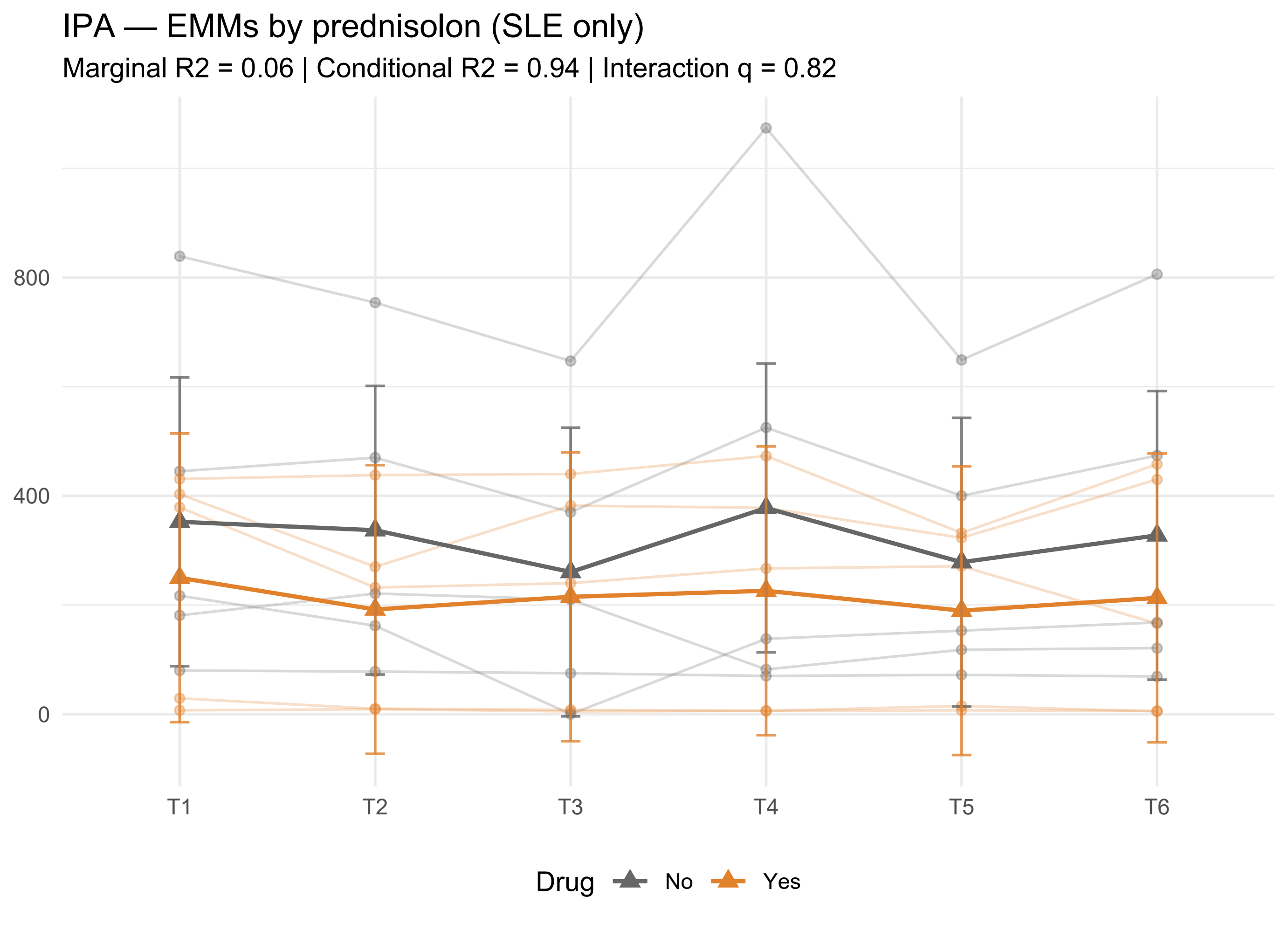
IPA — EMMs by prednisolon (SLE only)
Marginal R2 = 0.06 | Conditional R2 = 0.94 | Interaction q = 0.82
800
400
0
T1
T2
T3
T4
T5
T6
Drug
No
Yes

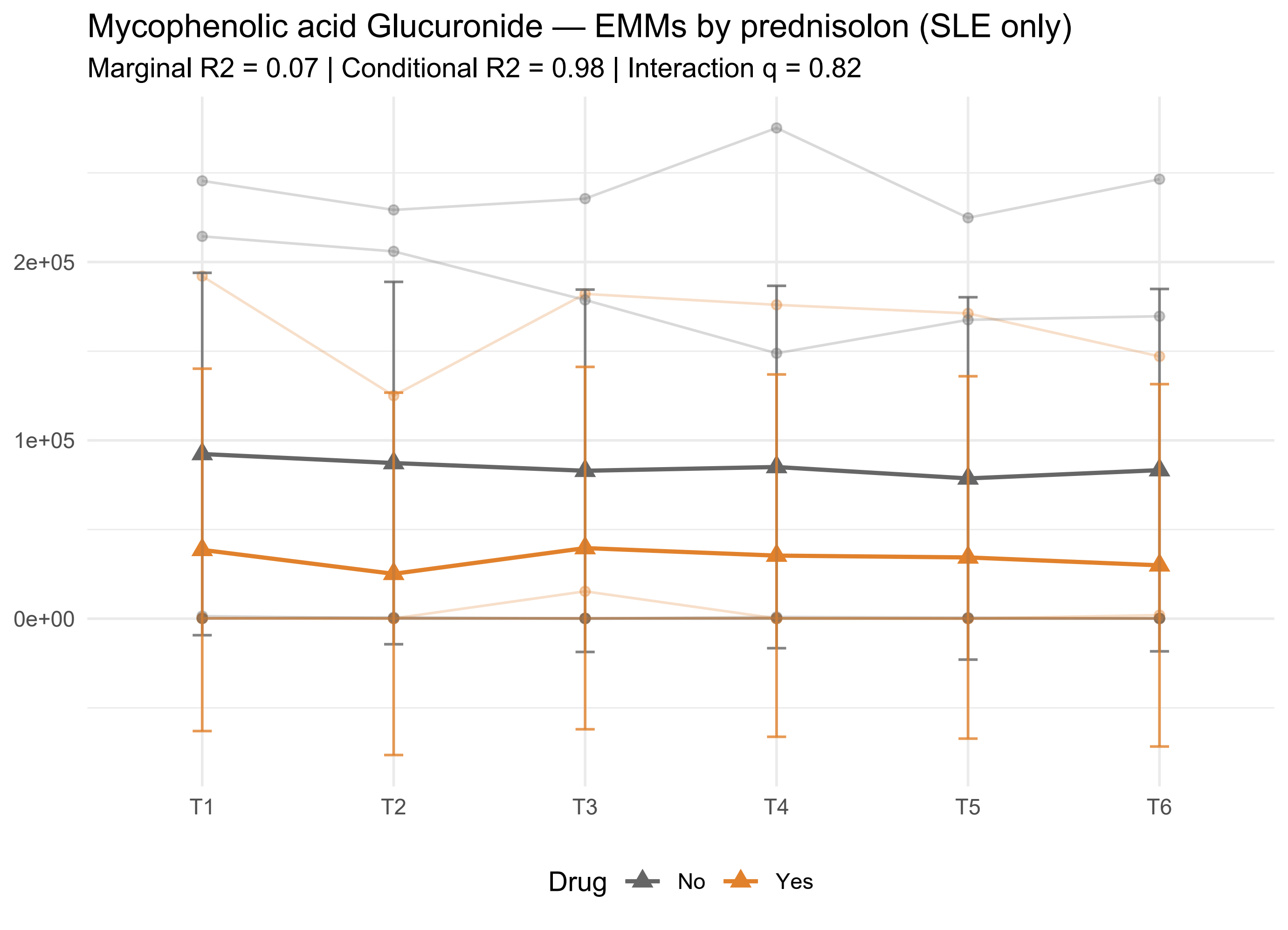

Mycophenolic acid Glucuronide — EMMs by prednisolon (SLE only)
Marginal R2 = 0.07 | Conditional R2 = 0.98 | Interaction q = 0.82
2e+05
1e+05
0e+00
T1
T2
T3
T4
T5
T6
Drug
No
Yes

Paraxanthine — EMMs by prednisolon (SLE only)

Marginal R2 = 0.10 | Conditional R2 = 0.91 | Interaction q = 0.82

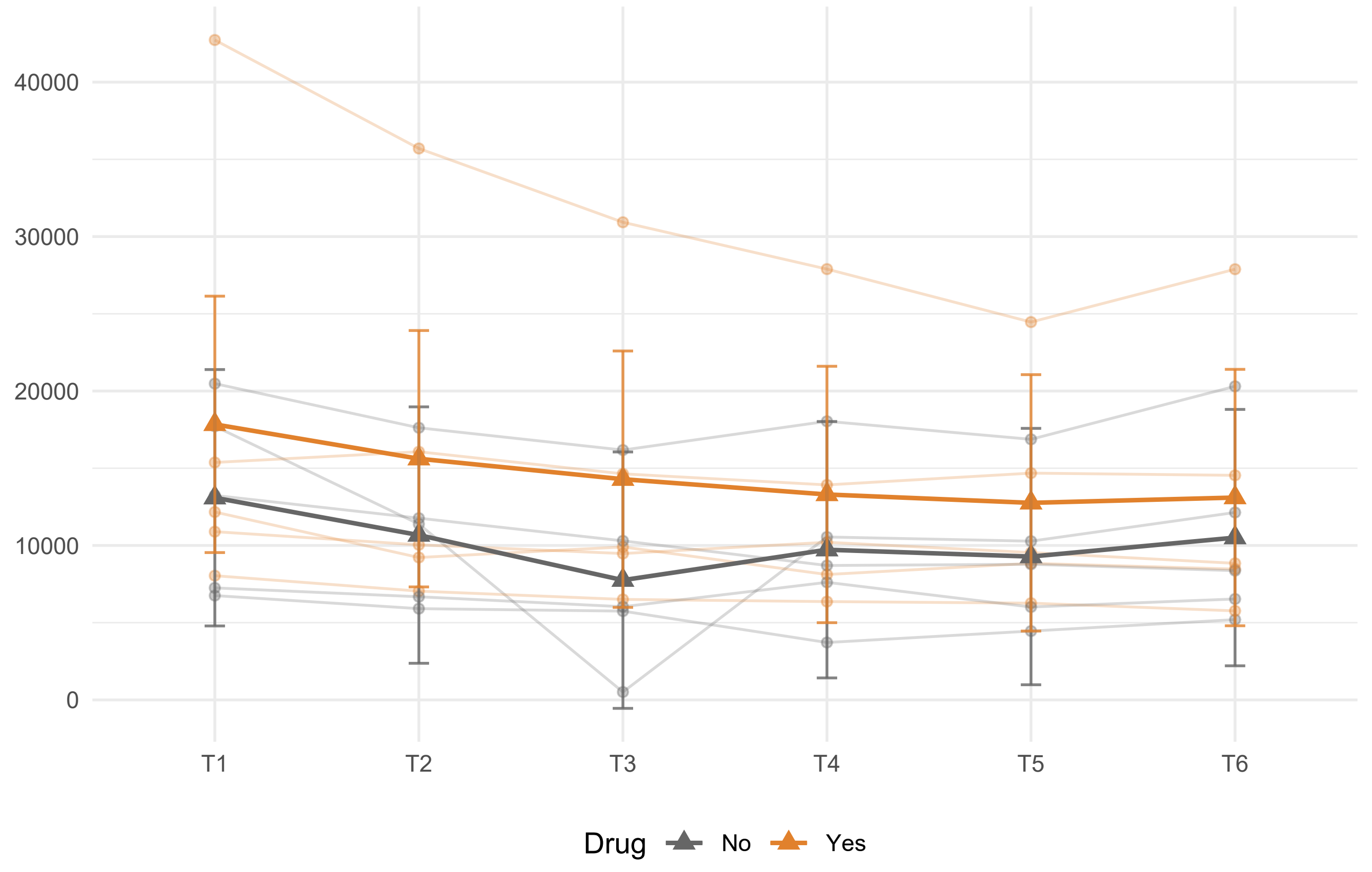

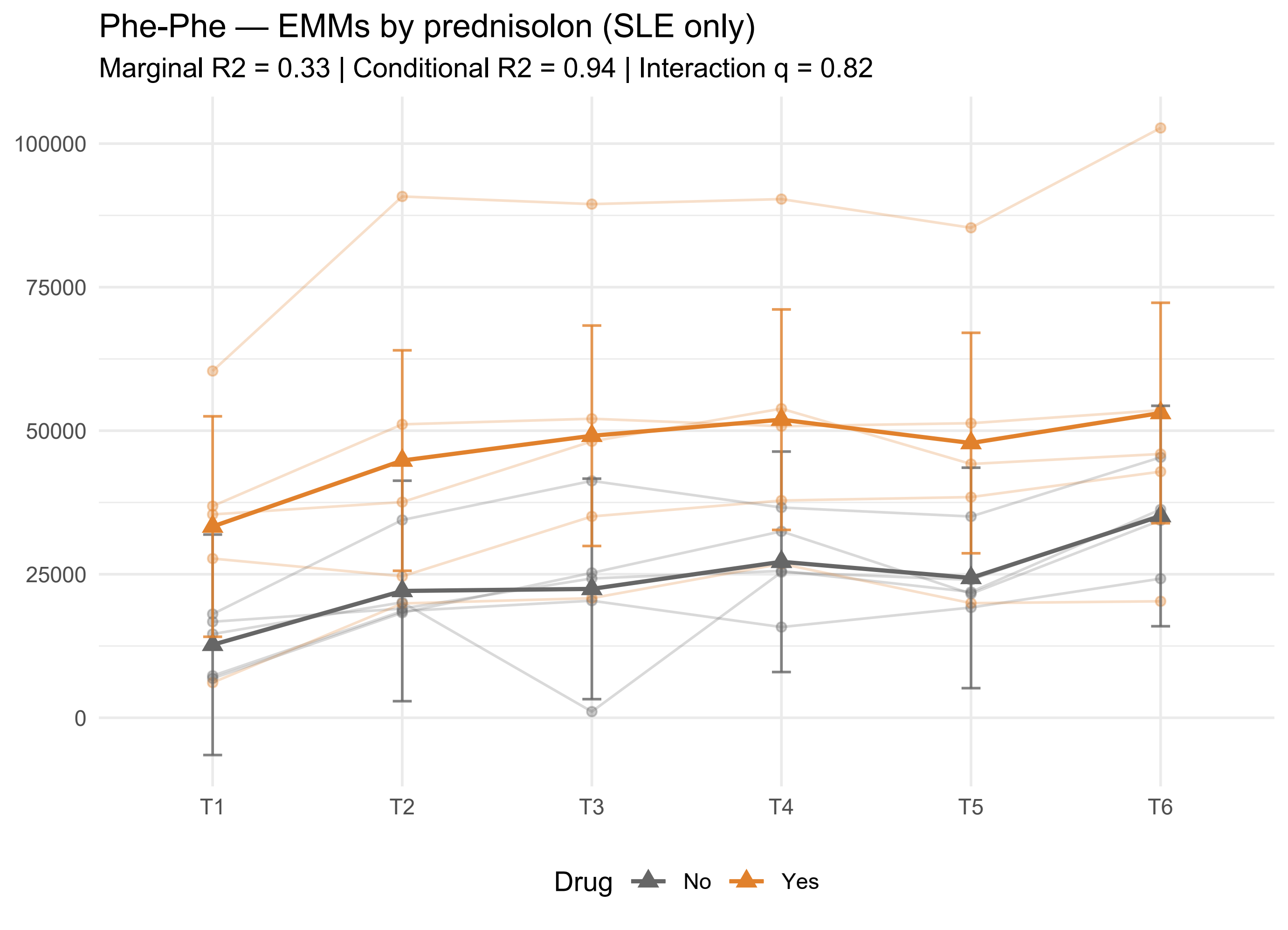

Phe-Phe — EMMs by prednisolon (SLE only)
Marginal R2 = 0.33 | Conditional R2 = 0.94 | Interaction q = 0.82
100000
75000
50000
25000
0
T1
T2
T3
T4
T5
T6
Drug
No
Yes

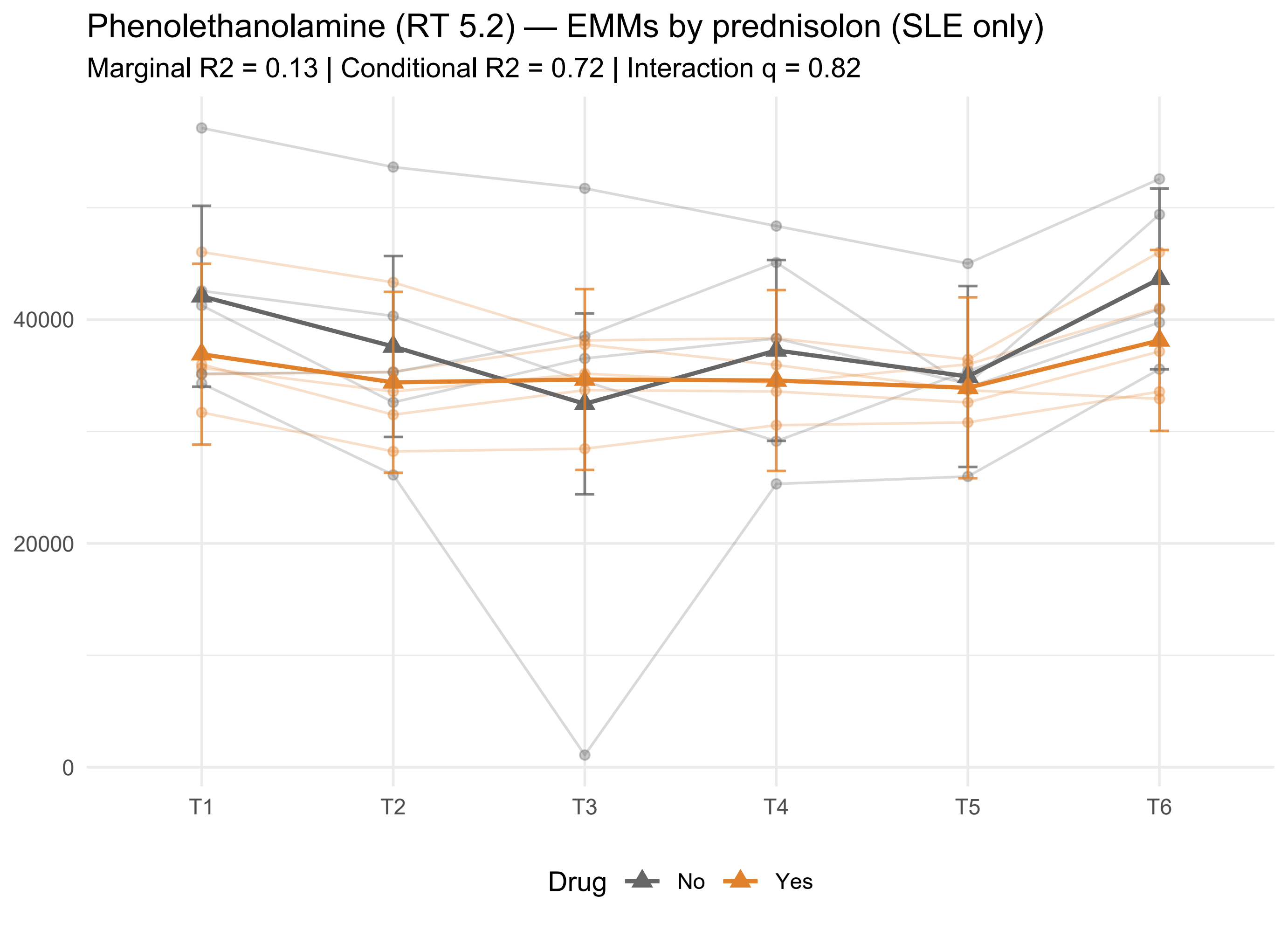

Phenolethanolamine (RT 5.2) — EMMs by prednisolon (SLE only)
Marginal R2 = 0.13 | Conditional R2 = 0.72 | Interaction q = 0.82
40000
20000
0
T1
T2
T3
T4
T5
T6
Drug
No
Yes

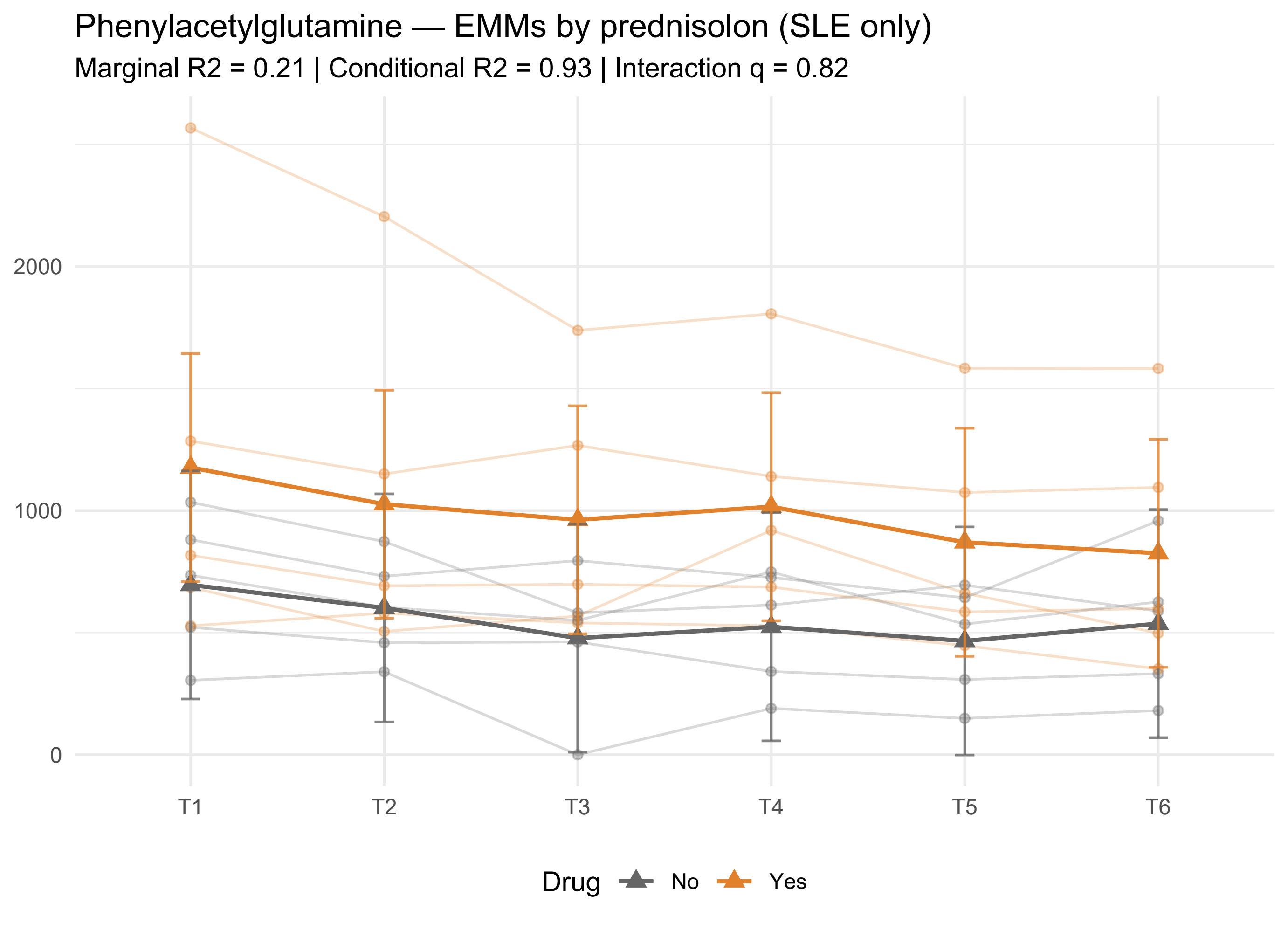
Phenylacetylglutamine — EMMs by prednisolon (SLE only)
Marginal R2 = 0.21 | Conditional R2 = 0.93 | Interaction q = 0.82
2000
1000
0
T1
T2
T3
T4
T5
T6
Drug
No
Yes

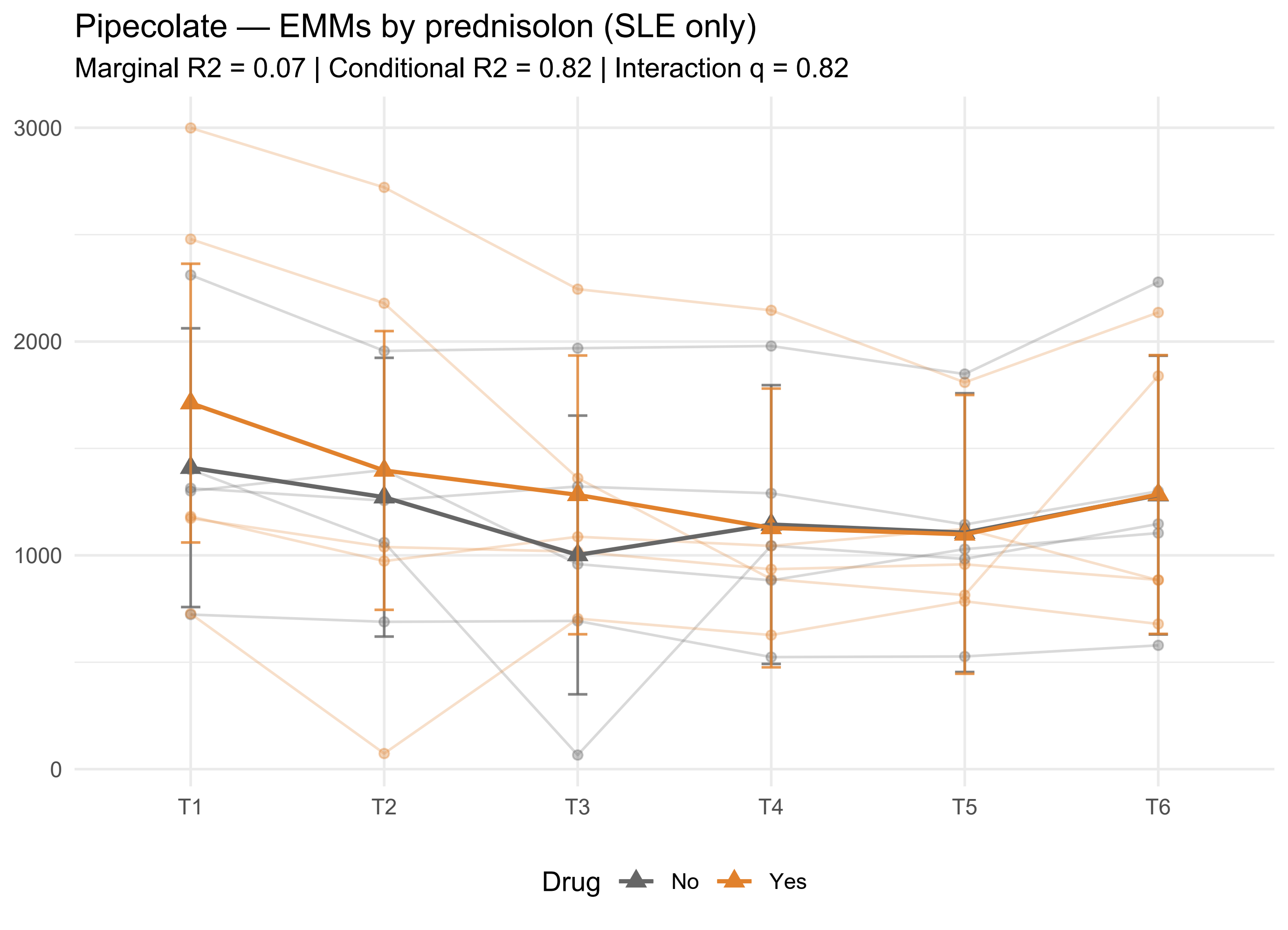

Pipecolate — EMMs by prednisolon (SLE only)
Marginal R2 = 0.07 | Conditional R2 = 0.82 | Interaction q = 0.82
3000
2000
1000
0
T1
T2
T3
T4
T5
T6
Drug
No
Yes

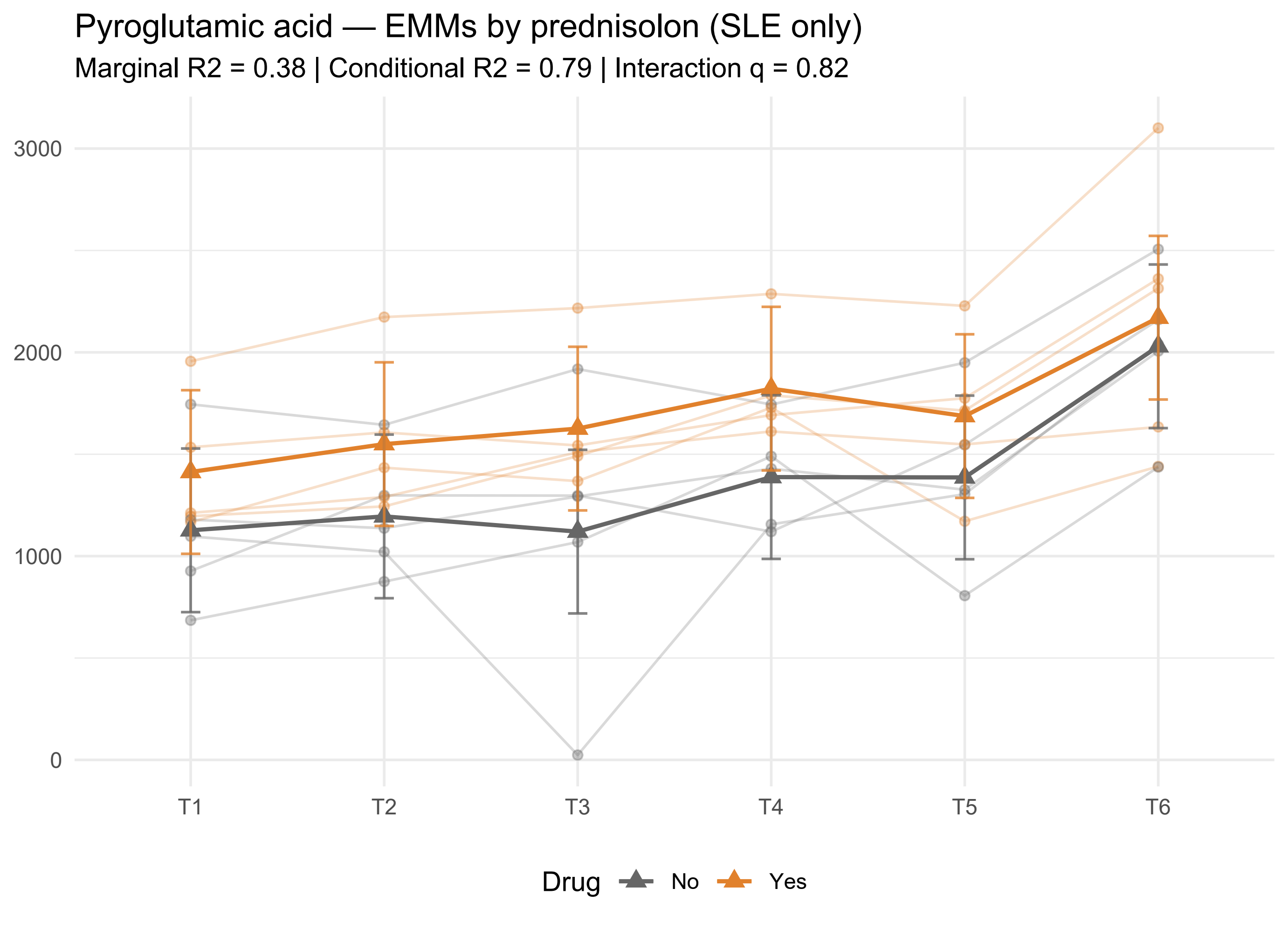
Pyroglutamic acid — EMMs by prednisolon (SLE only)
Marginal R2 = 0.38 | Conditional R2 = 0.79 | Interaction q = 0.82
3000
2000
1000
0
T1
T2
T3
T4
T5
T6
Drug
No
Yes

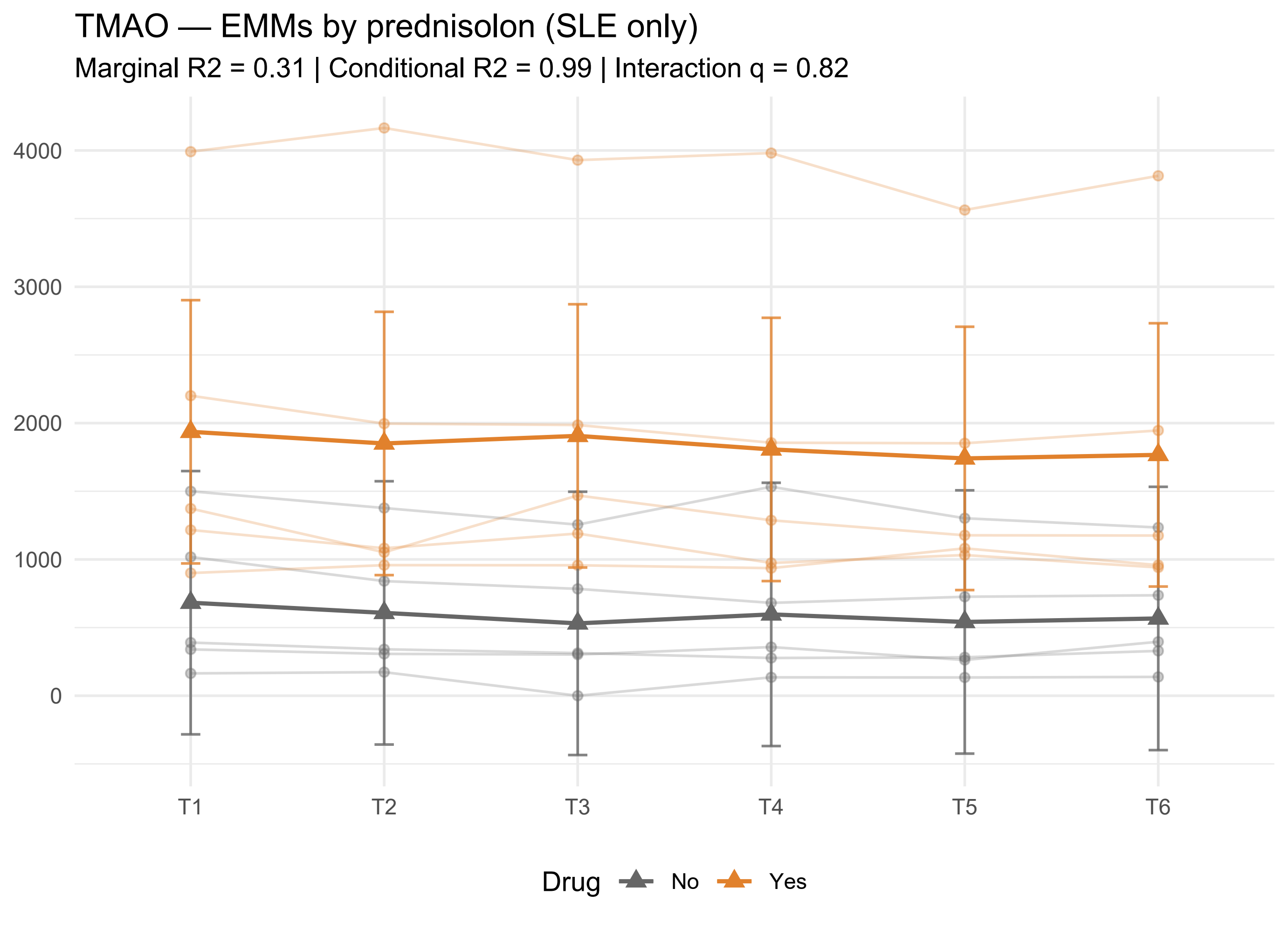

TMAO — EMMs by prednisolon (SLE only)
Marginal R2 = 0.31 | Conditional R2 = 0.99 | Interaction q = 0.82
4000
3000
2000
1000
0
T1
T2
T3
T4
T5
T6
Drug
No
Yes

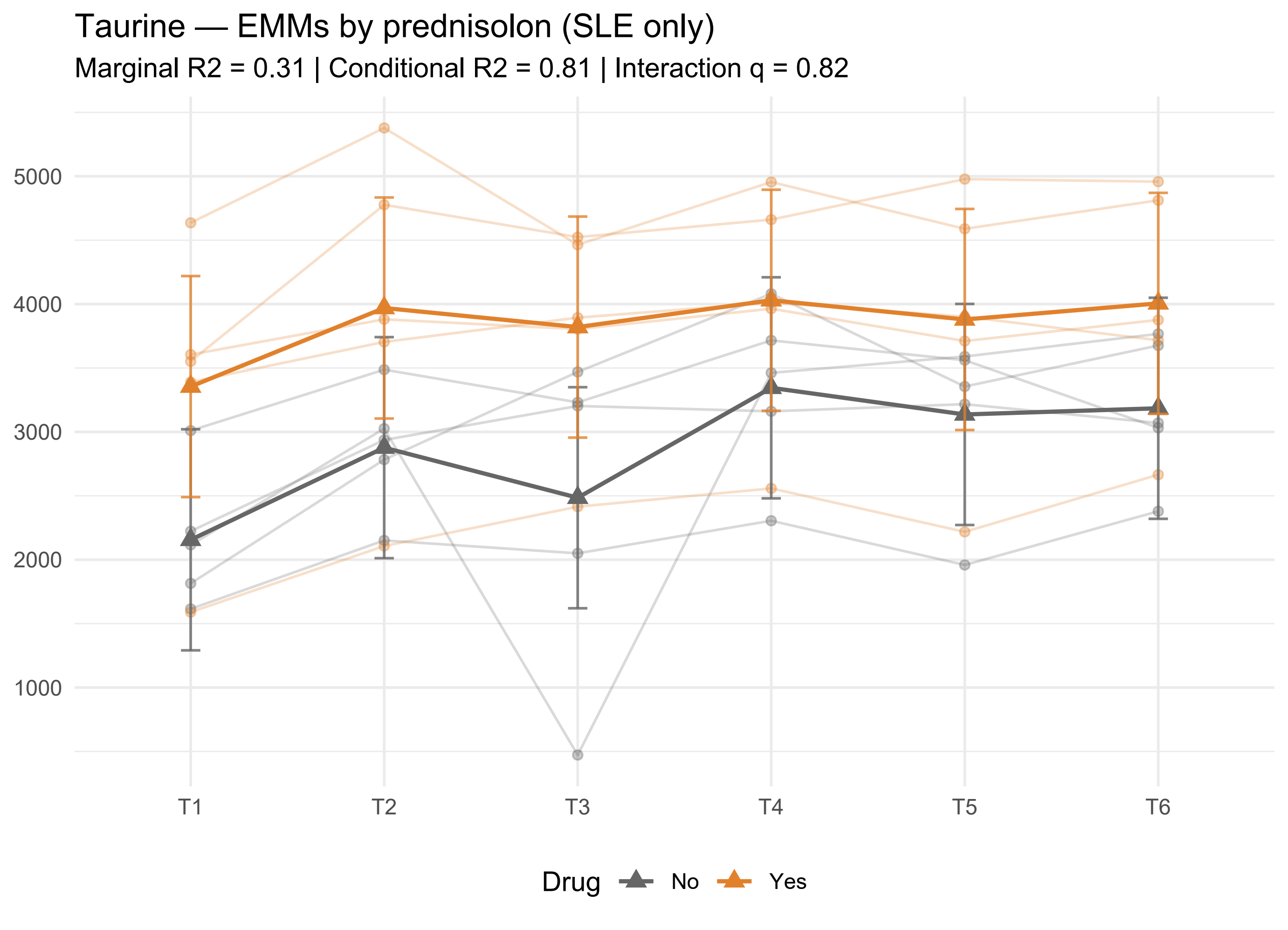

Taurine — EMMs by prednisolon (SLE only)
Marginal R2 = 0.31 | Conditional R2 = 0.81 | Interaction q = 0.82
5000
4000
3000
2000
1000
T1
T2
T3
T4
T5
T6
Drug
No
Yes

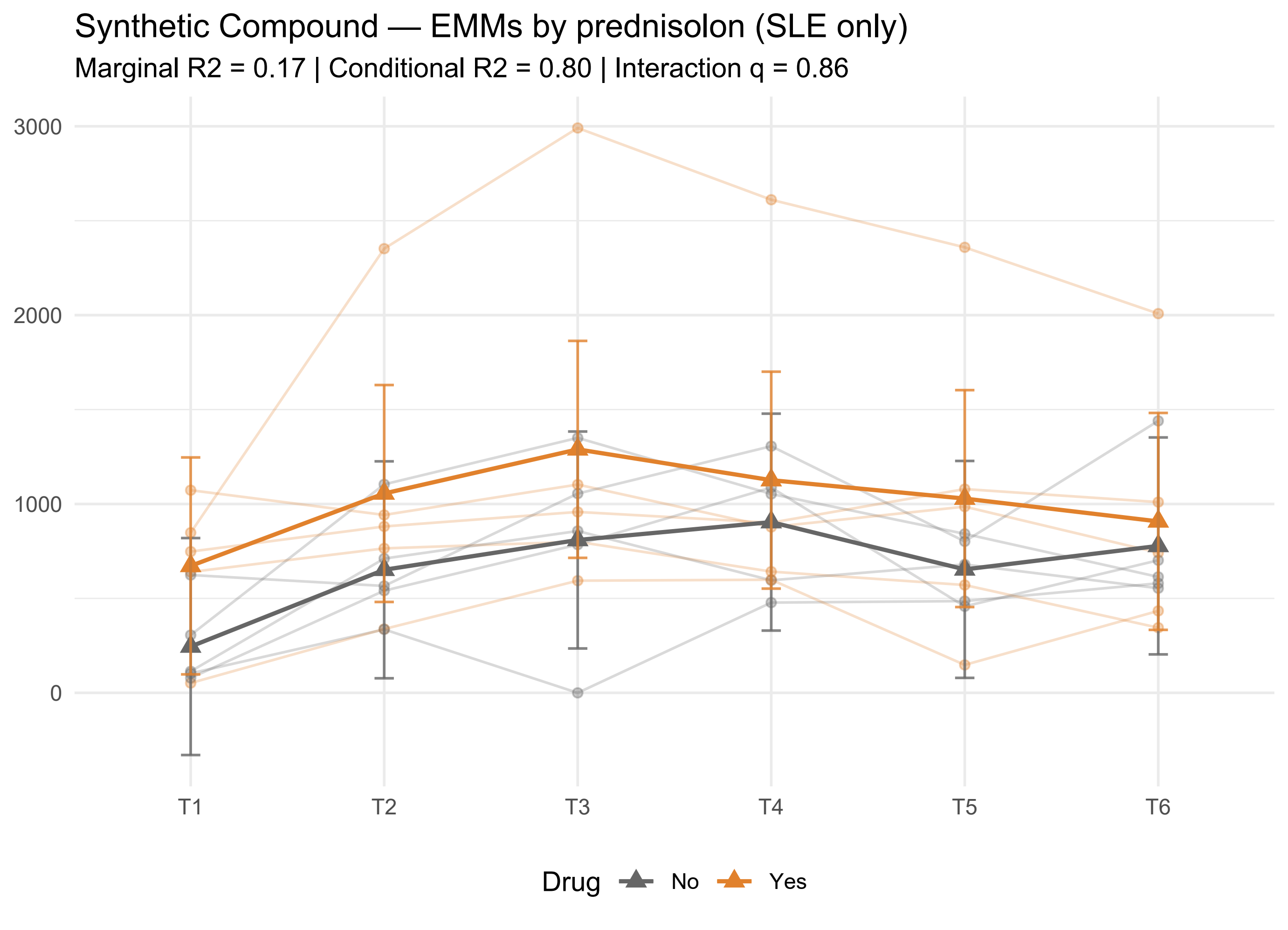

Synthetic Compound — EMMs by prednisolon (SLE only)
Marginal R2 = 0.17 | Conditional R2 = 0.80 | Interaction q = 0.86
3000
2000
1000
0
T1
T2
T3
T4
T5
T6
Drug
No
Yes

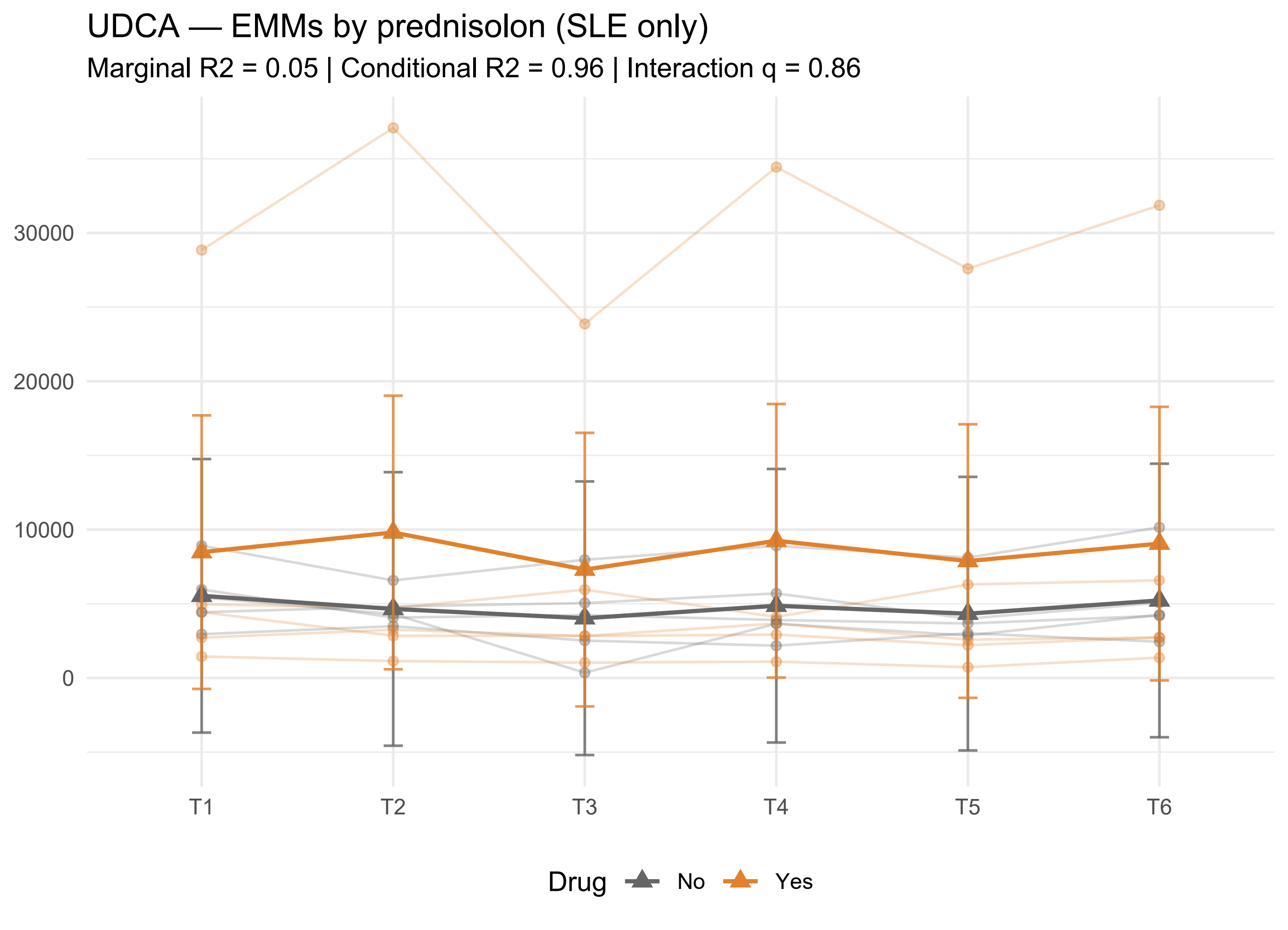

UDCA — EMMs by prednisolon (SLE only)
Marginal R2 = 0.05 | Conditional R2 = 0.96 | Interaction q = 0.86
30000
20000
10000
0
T1
T2
T3
T4
T5
T6
Drug
No
Yes

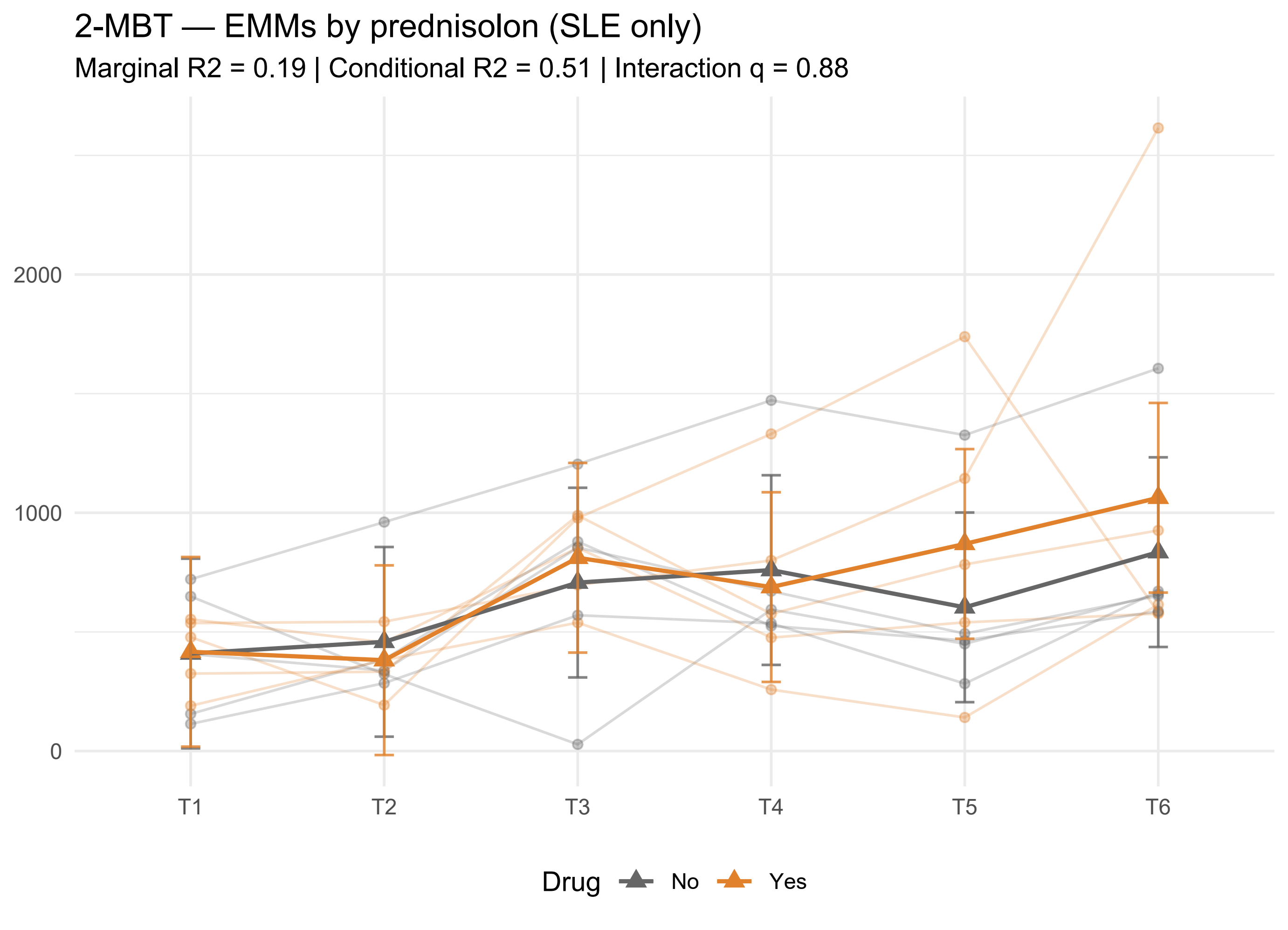

2-MBT — EMMs by prednisolon (SLE only)
Marginal R2 = 0.19 | Conditional R2 = 0.51 | Interaction q = 0.88
2000
1000
0
T1
T2
T3
T4
T5
T6
Drug
No
Yes

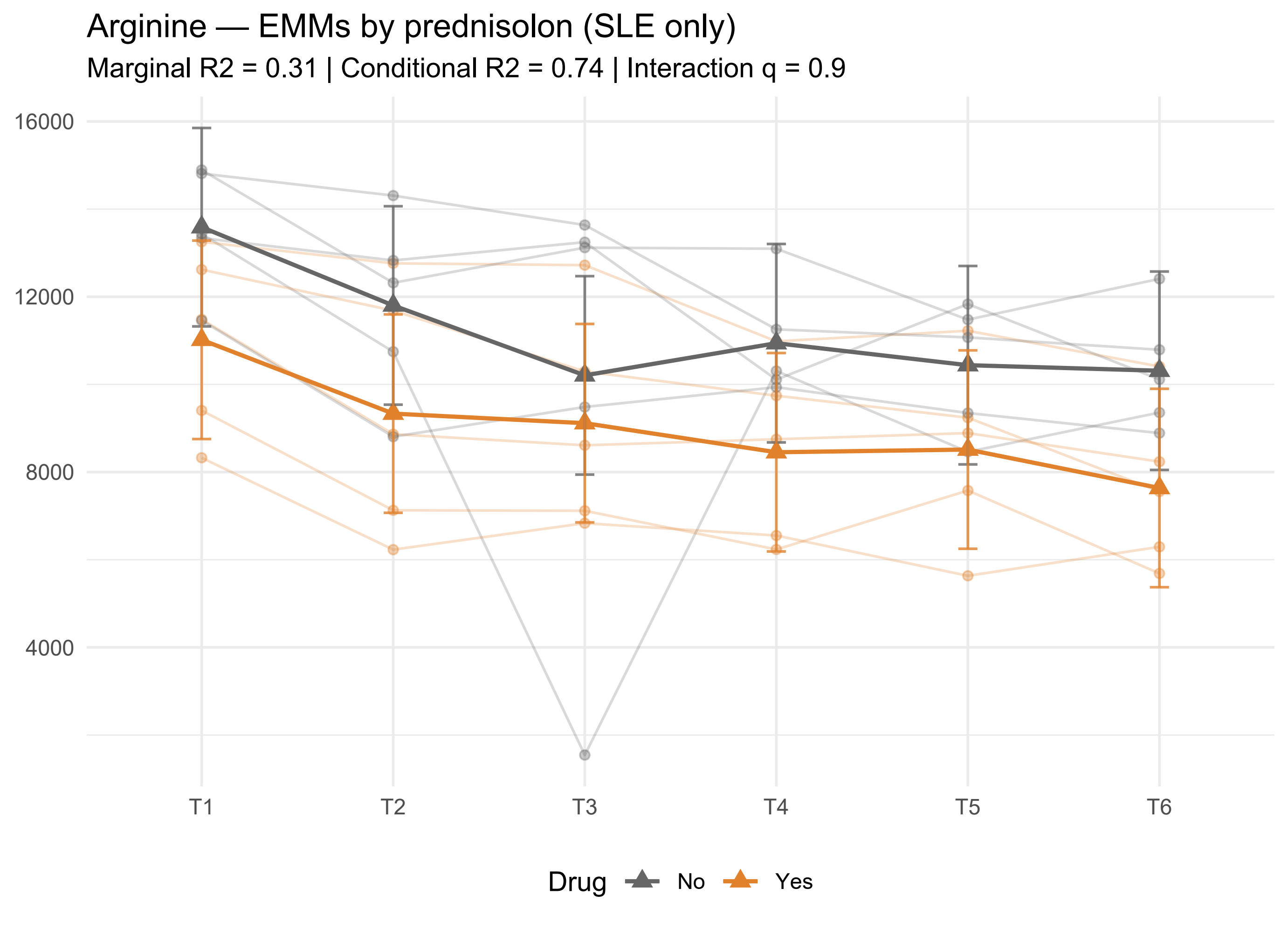

Arginine — EMMs by prednisolon (SLE only)
Marginal R2 = 0.31 | Conditional R2 = 0.74 | Interaction q = 0.9
16000
12000
8000
4000
T1
T2
T3
T4
T5
T6
Drug
No
Yes

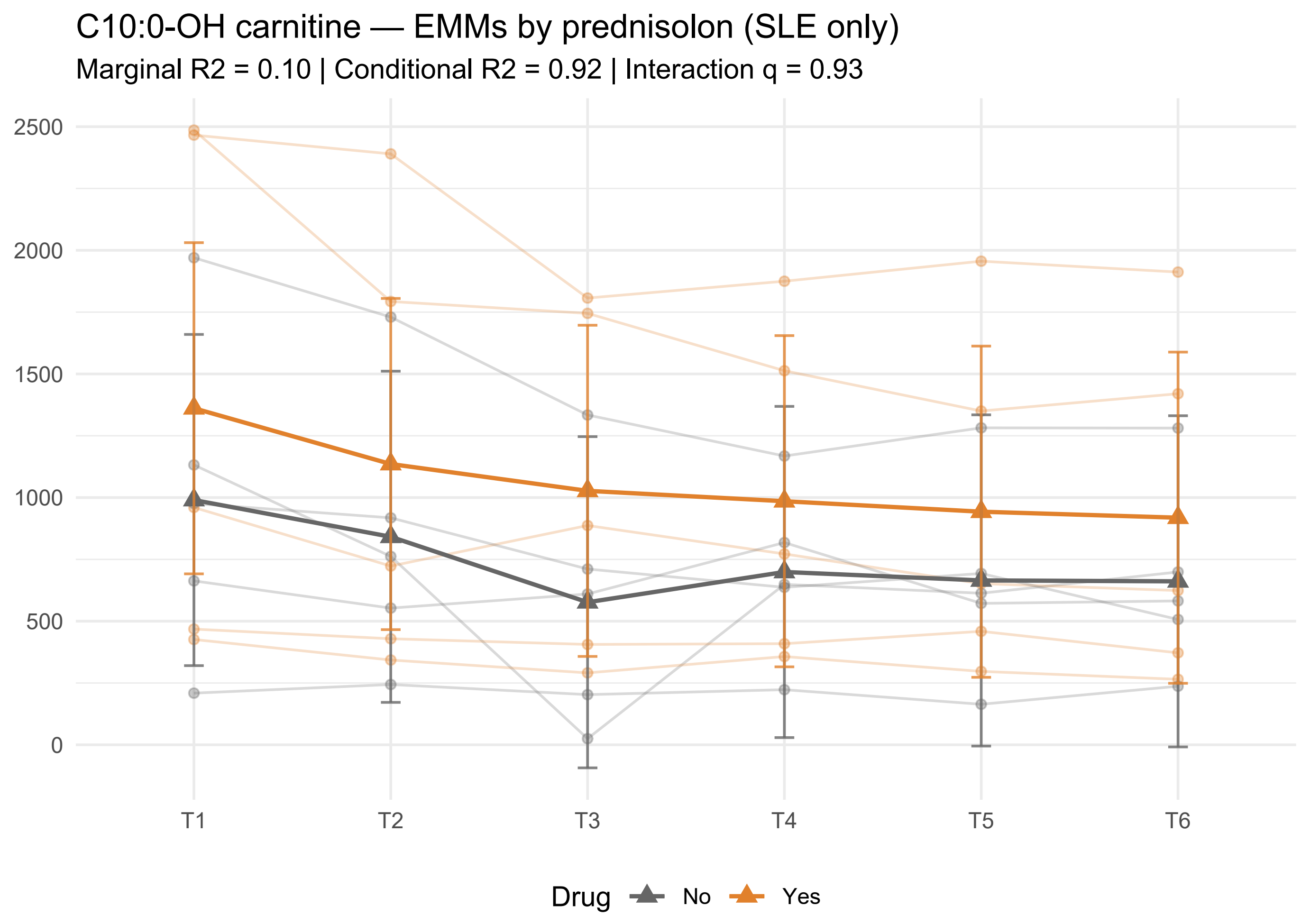
C10:0-OH carnitine — EMMs by prednisolon (SLE only)
Marginal R2 = 0.10 | Conditional R2 = 0.92 | Interaction q = 0.93
2500
2000
1500
1000
500
0
T1
T2
T3
T4
T5
T6
Drug
No
Yes

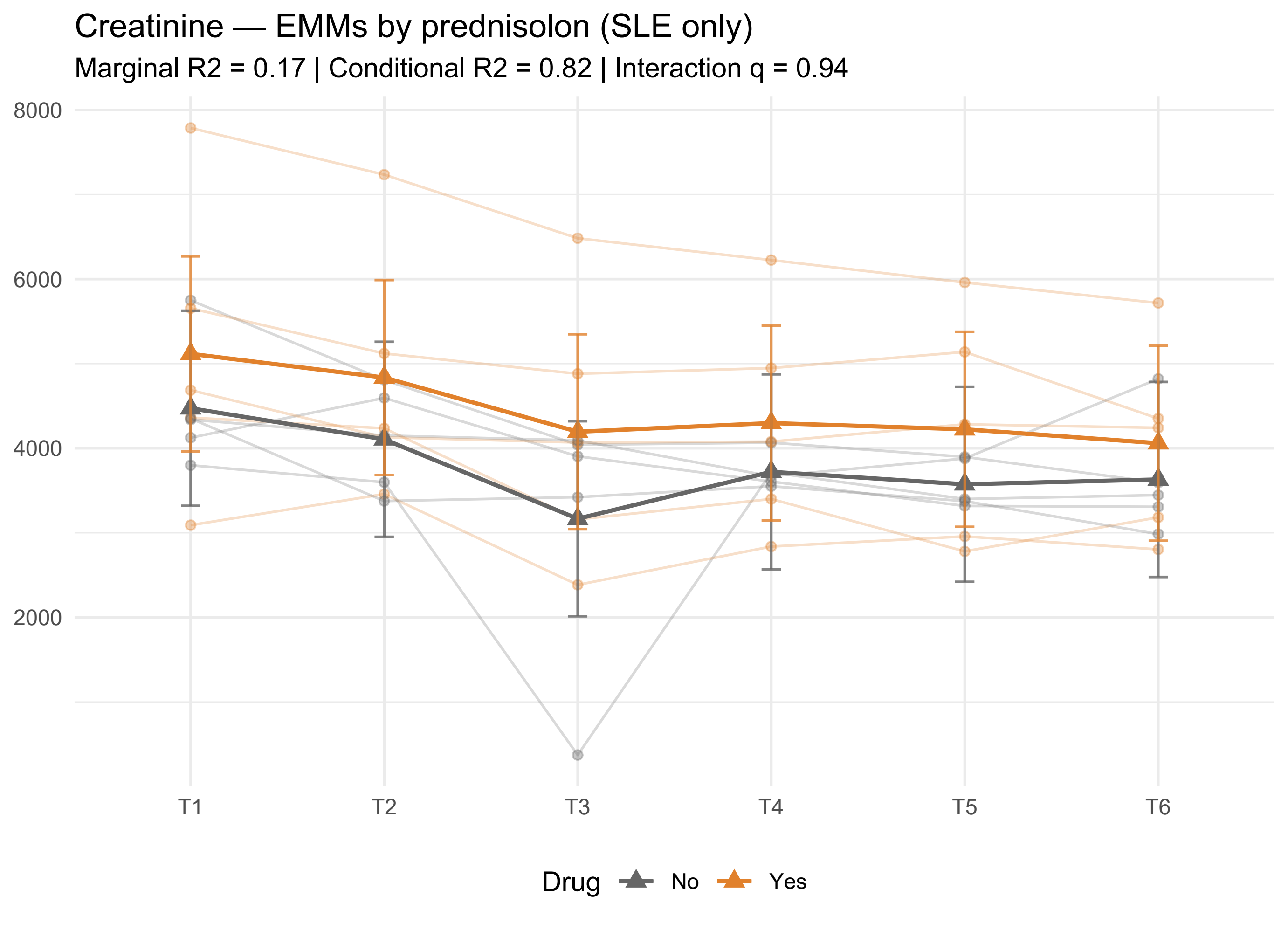
Creatinine — EMMs by prednisolon (SLE only)
Marginal R2 = 0.17 | Conditional R2 = 0.82 | Interaction q = 0.94
8000
6000
4000
2000
T1
T2
T3
T4
T5
T6
Drug
No
Yes

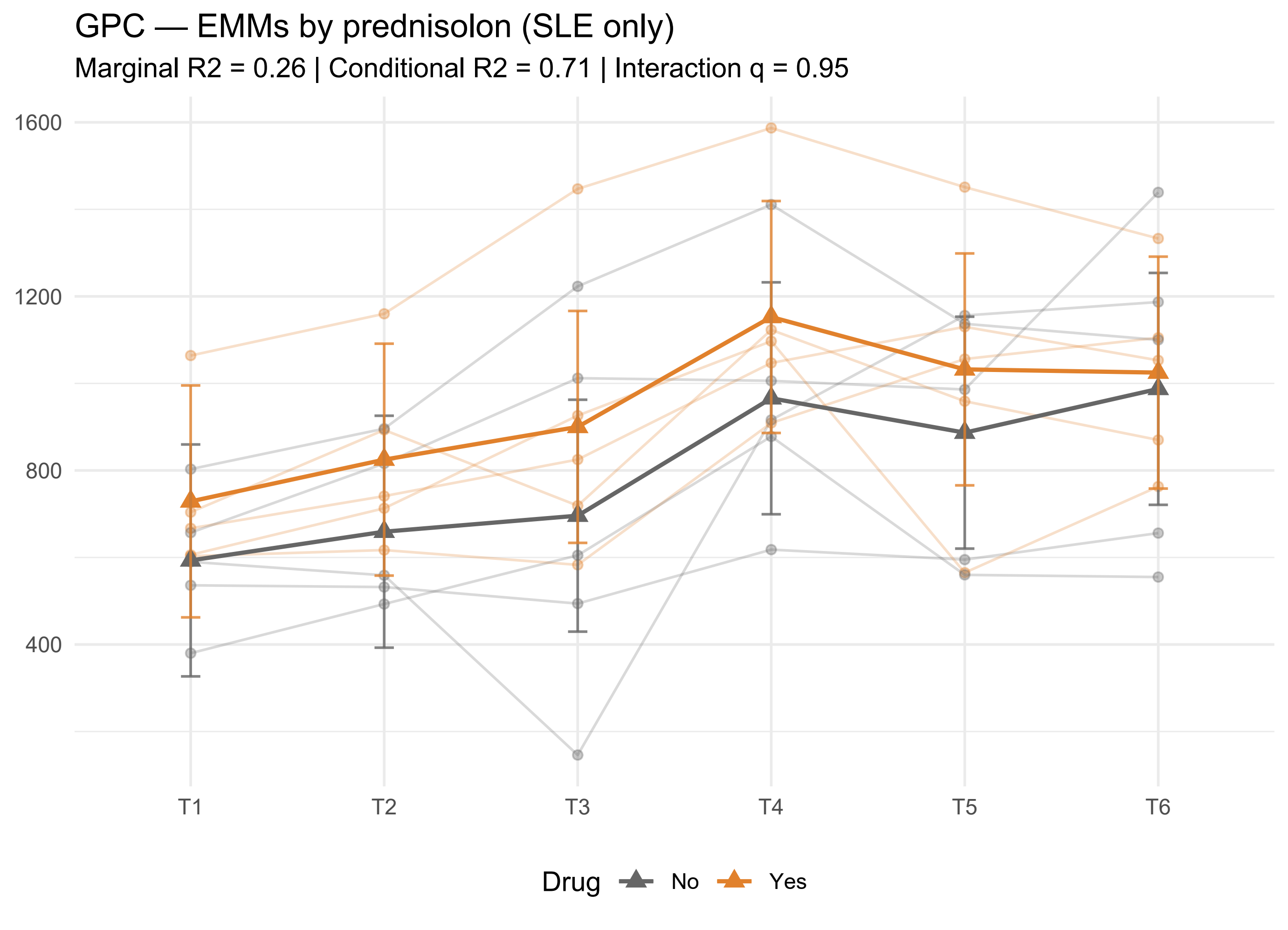
GPC — EMMs by prednisolon (SLE only)
Marginal R2 = 0.26 | Conditional R2 = 0.71 | Interaction q = 0.95
1600
1200
800
400
T1
T2
T3
T4
T5
T6
Drug
No
Yes

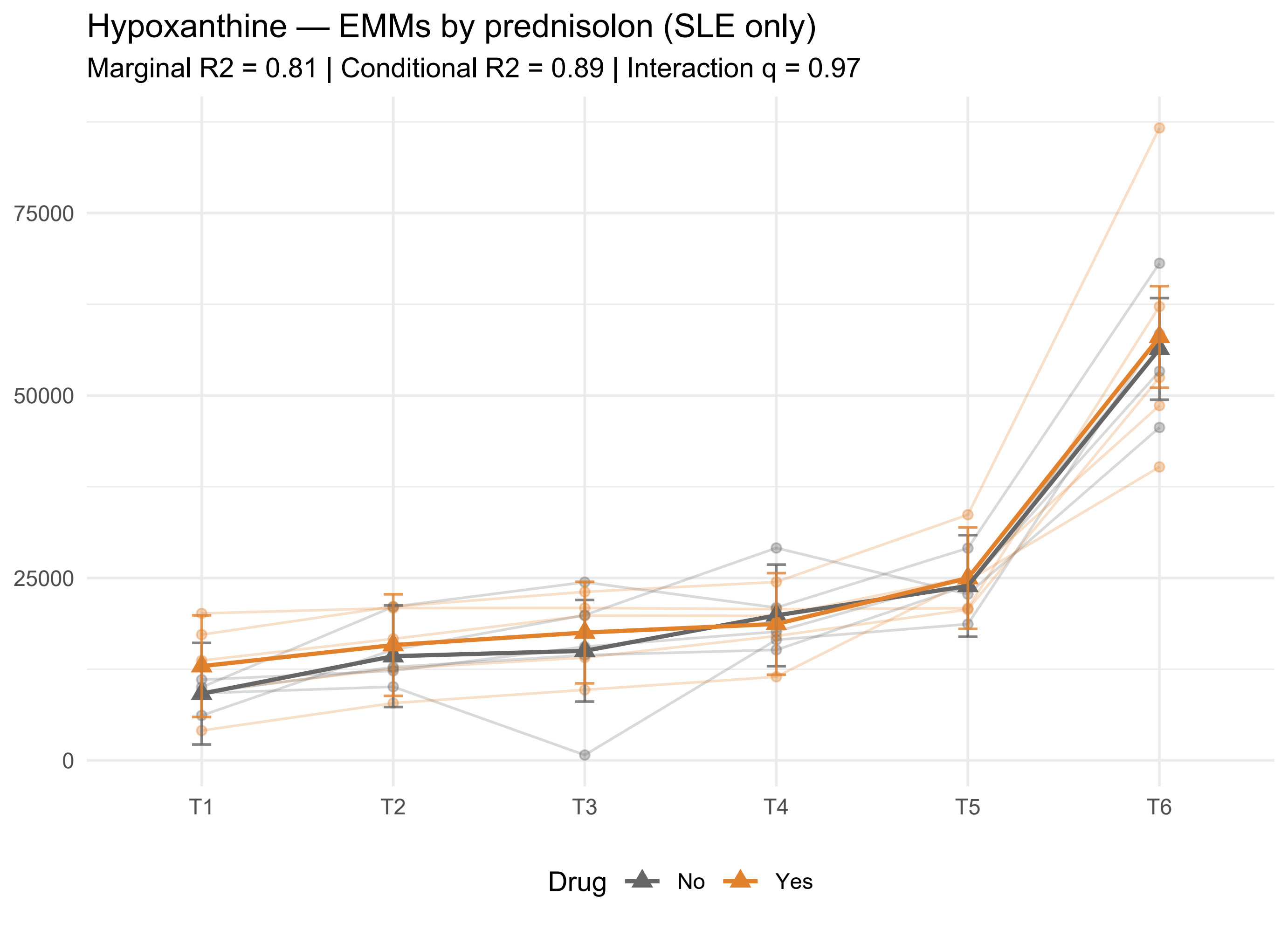

Hypoxanthine — EMMs by prednisolon (SLE only)
Marginal R2 = 0.81 | Conditional R2 = 0.89 | Interaction q = 0.97
75000
50000
25000
0
T1
T2
T3
T4
T5
T6
Drug
No
Yes

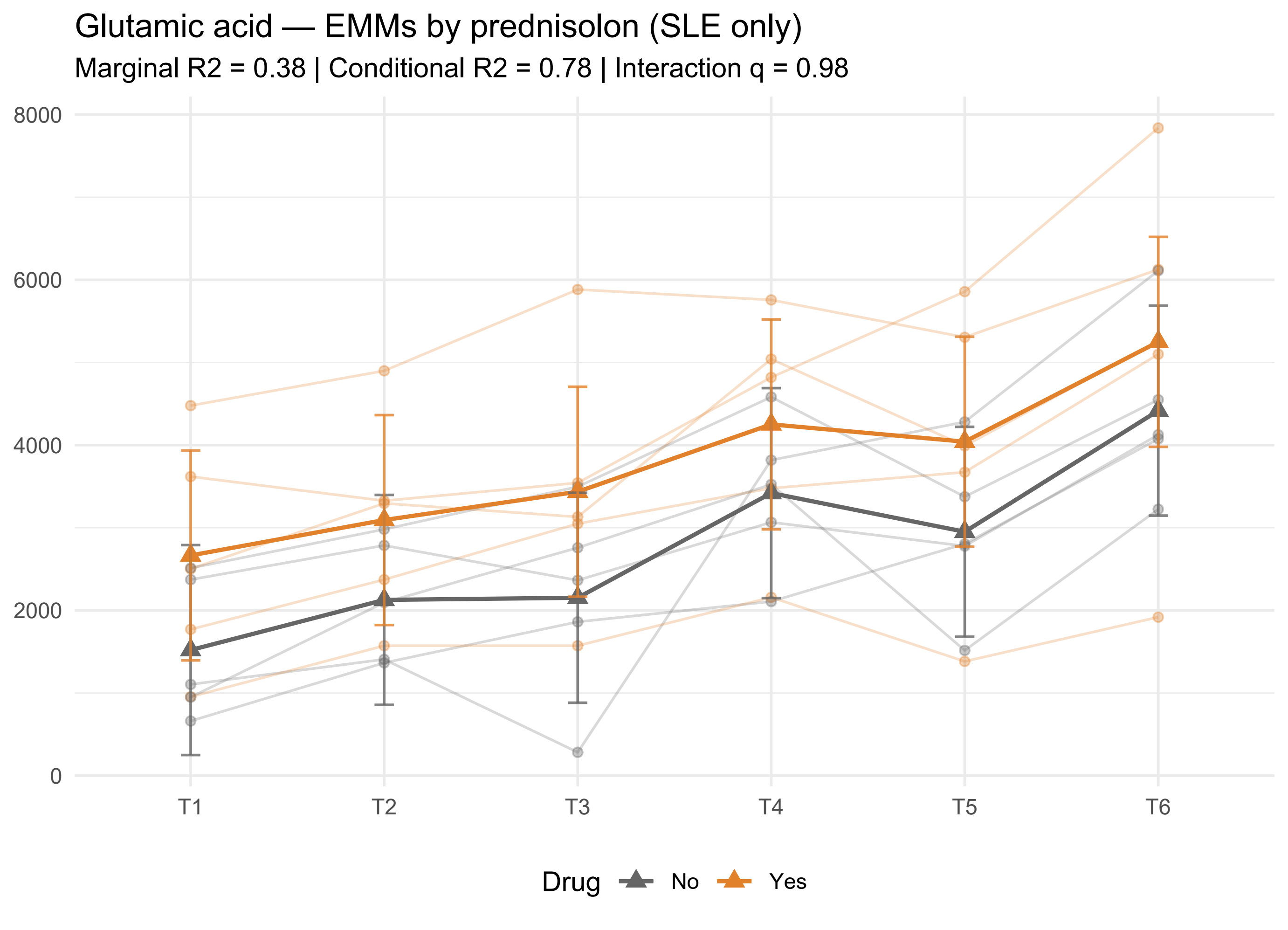

Glutamic acid — EMMs by prednisolon (SLE only)
Marginal R2 = 0.38 | Conditional R2 = 0.78 | Interaction q = 0.98
8000
6000
4000
2000
0
T1
T2
T3
T4
T5
T6
Drug
No
Yes

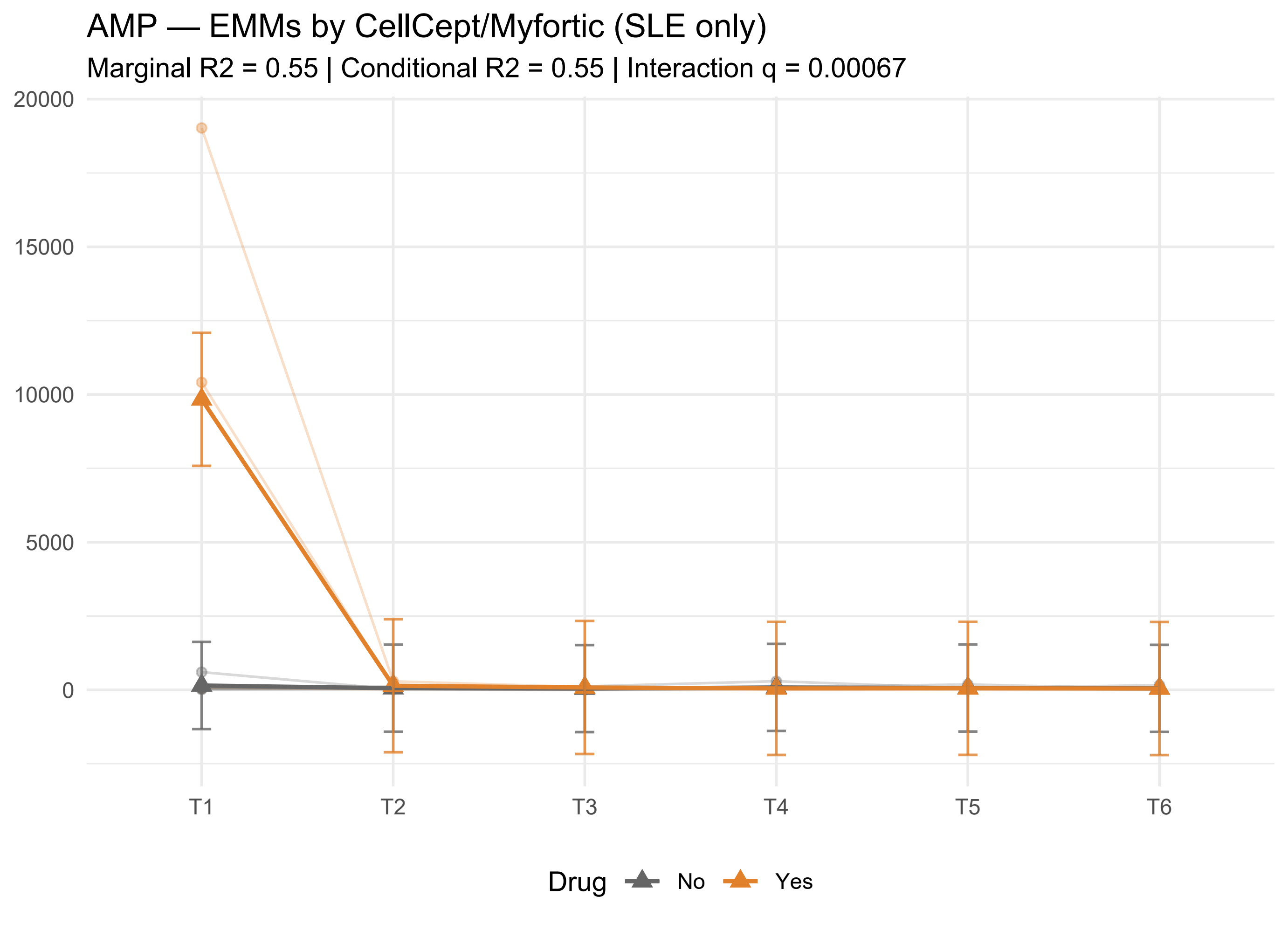

AMP — EMMs by CellCept/Myfortic (SLE only)
Marginal R2 = 0.55 | Conditional R2 = 0.55 | Interaction q = 0.00067
20000
15000
10000
5000
0
T1
T2
T3
T4
T5
T6
Drug
No
Yes

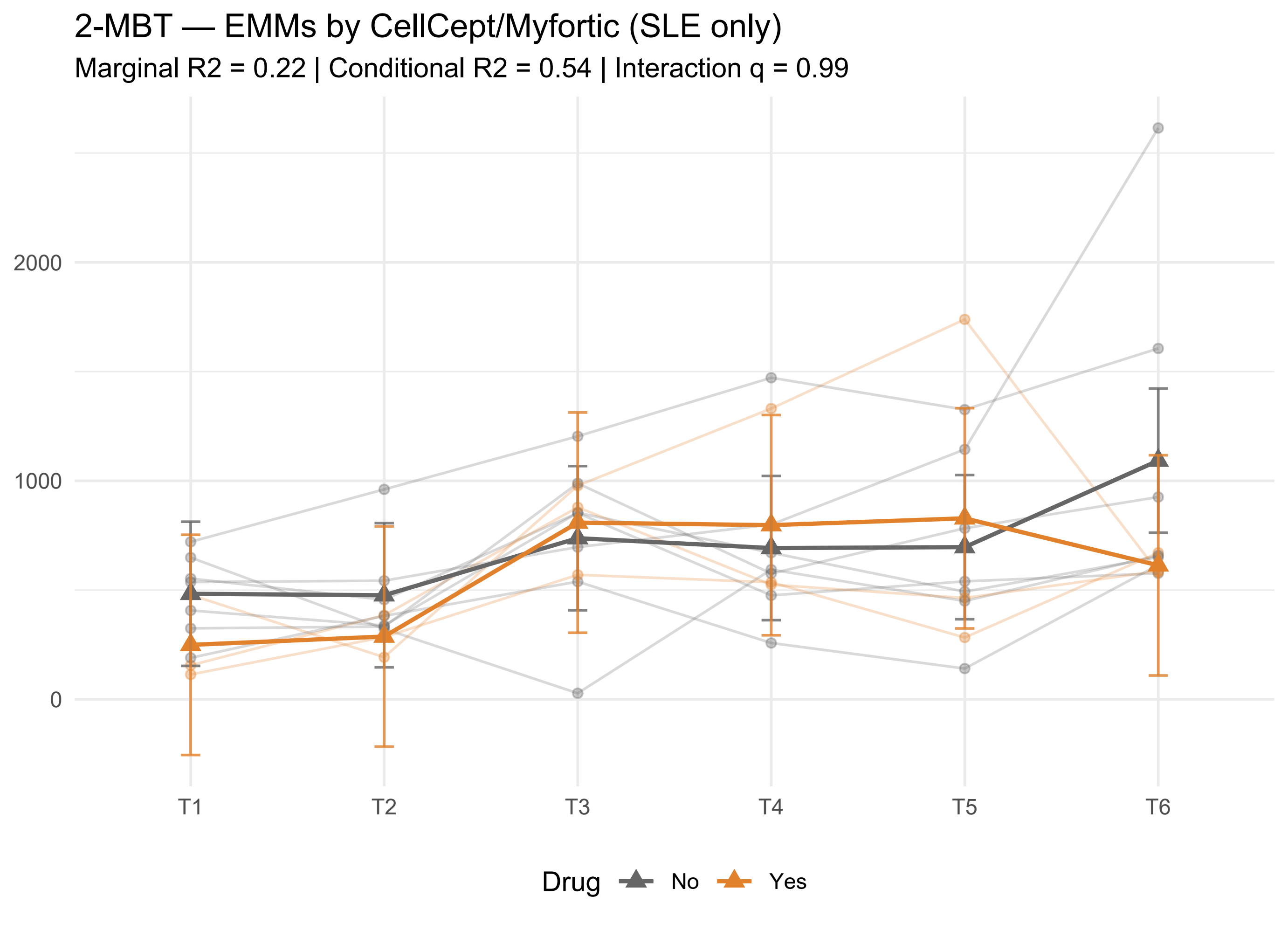
2-MBT — EMMs by CellCept/Myfortic (SLE only)
Marginal R2 = 0.22 | Conditional R2 = 0.54 | Interaction q = 0.99
2000
1000
0
T1
T2
T3
T4
T5
T6
Drug
No
Yes

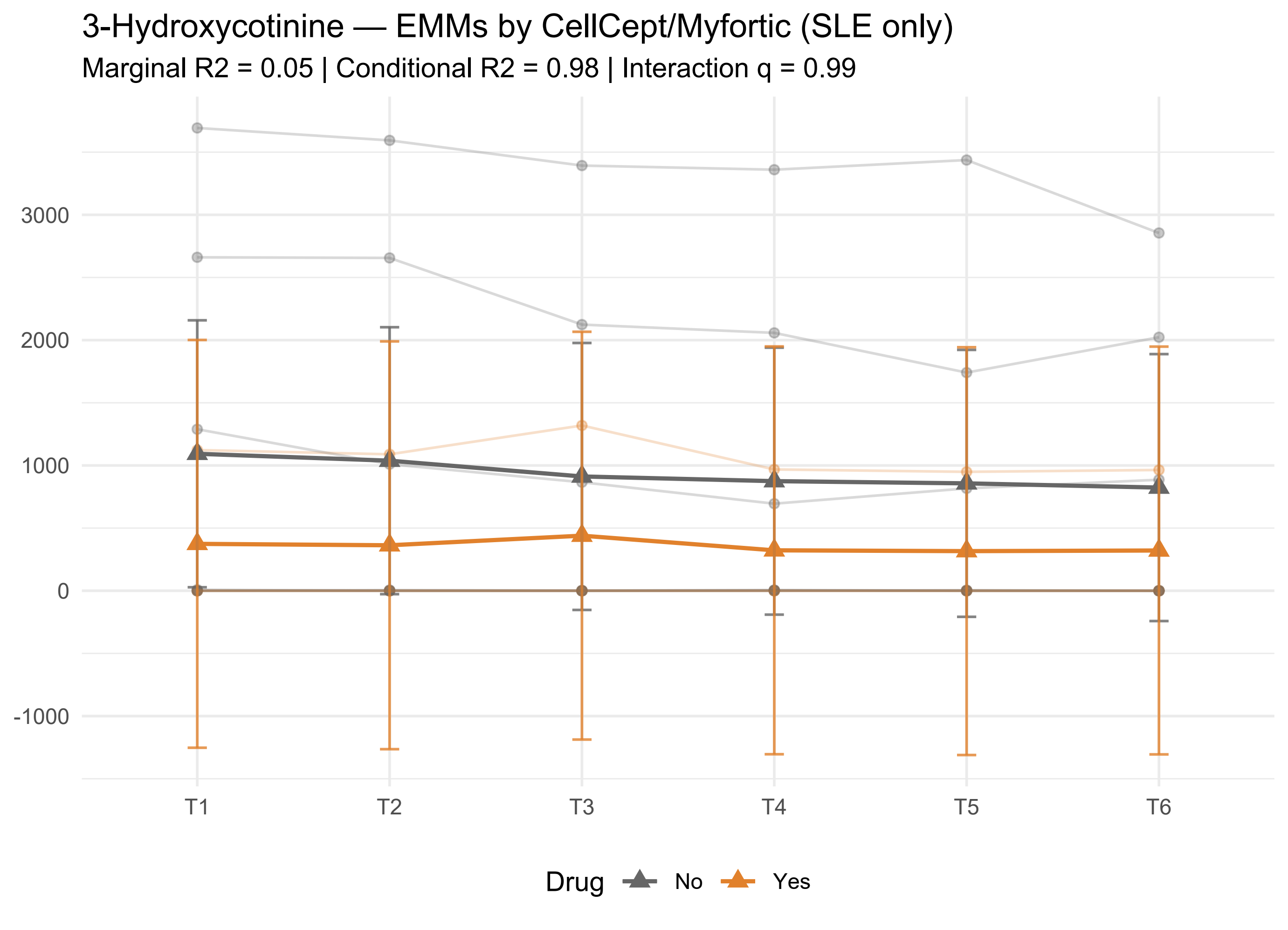

3-Hydroxycotinine — EMMs by CellCept/Myfortic (SLE only)
Marginal R2 = 0.05 | Conditional R2 = 0.98 | Interaction q = 0.99
3000
2000
1000
0
-1000
T1
T2
T3
T4
T5
T6
Drug
No
Yes

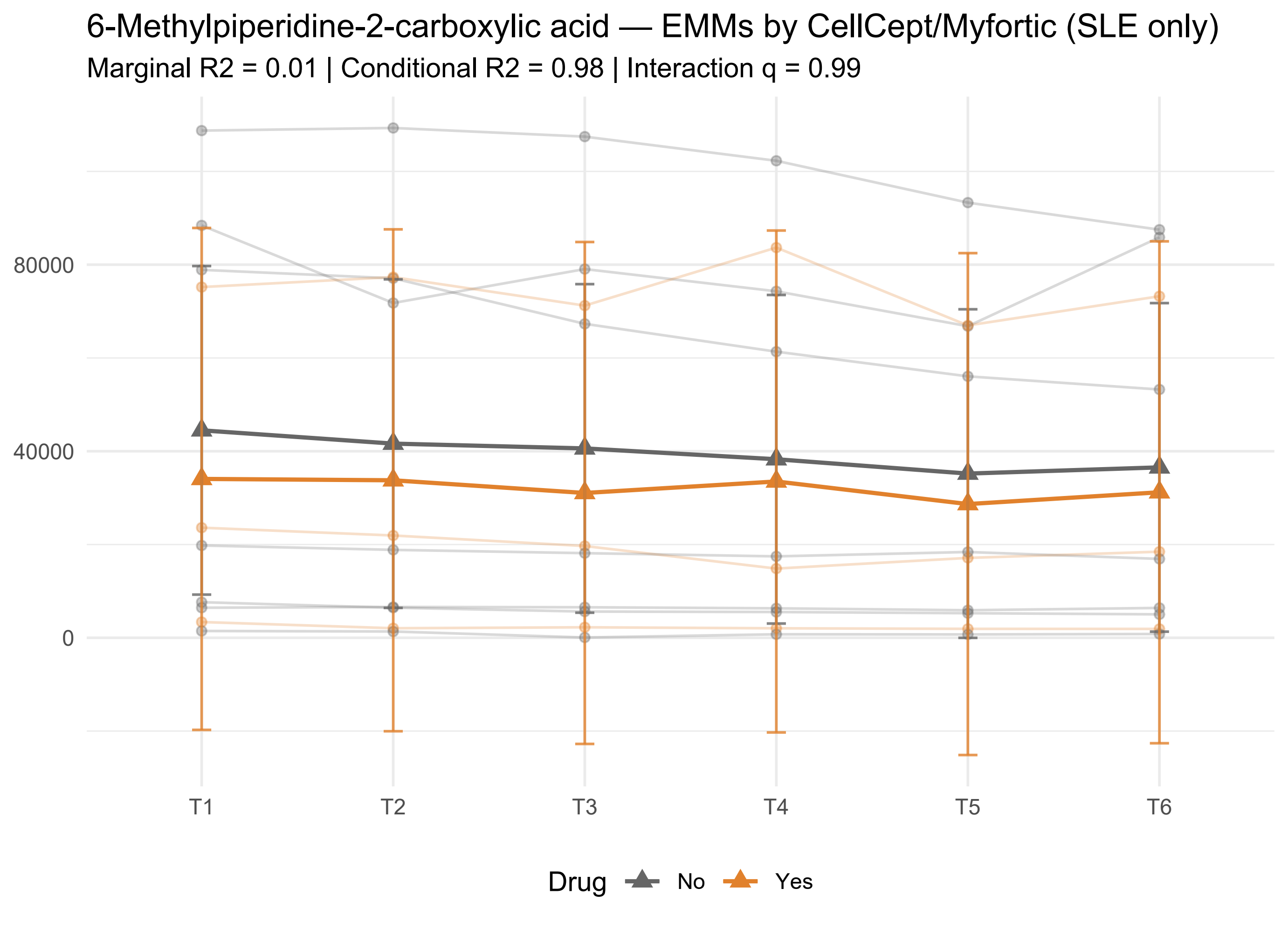

6-Methylpiperidine-2-carboxylic acid — EMMs by CellCept/Myfortic (SLE only)
Marginal R2 = 0.01 | Conditional R2 = 0.98 | Interaction q = 0.99
80000
40000
0
T1
T2
T3
T4
T5
T6
Drug
No
Yes

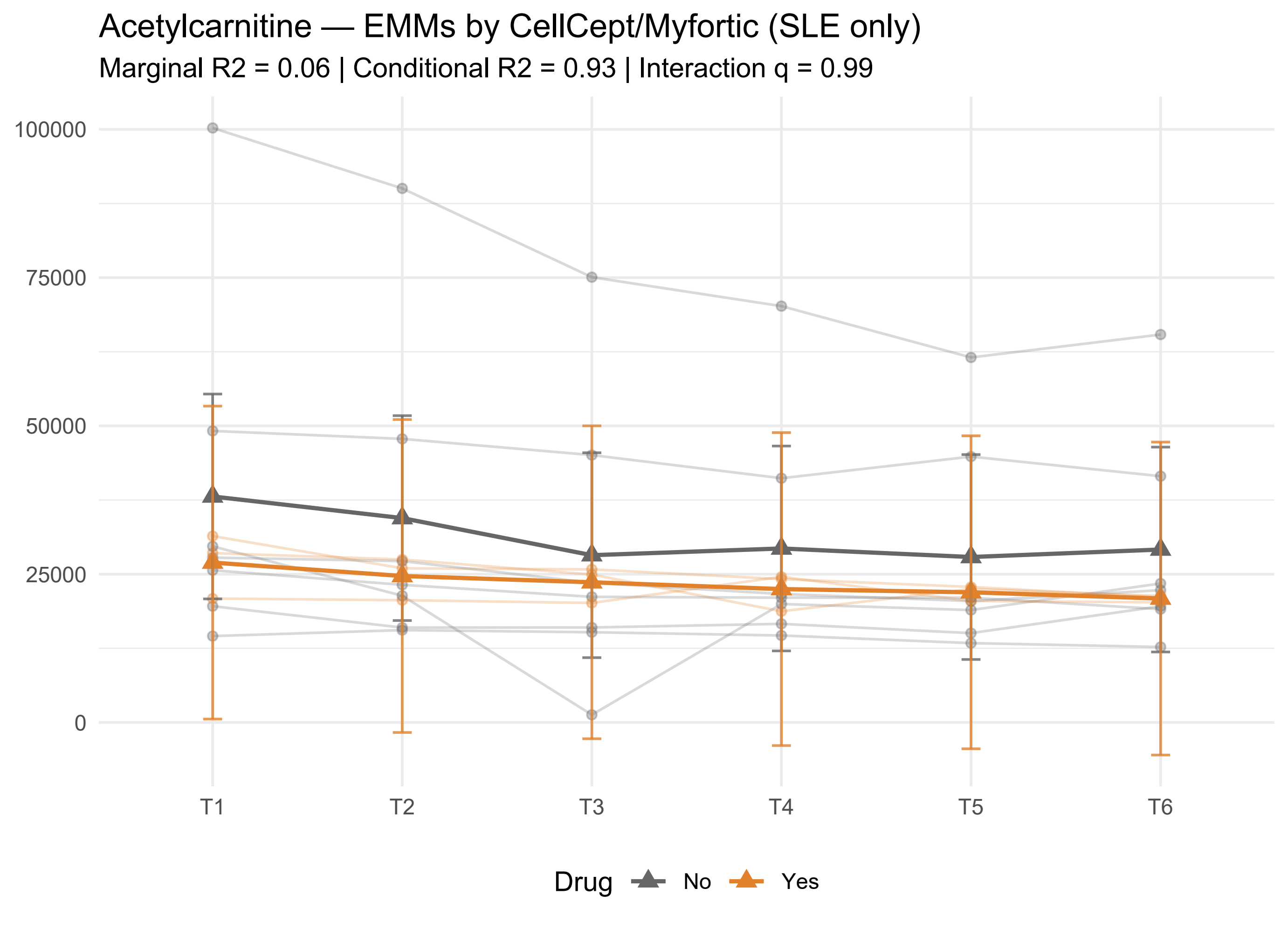
Acetylcarnitine — EMMs by CellCept/Myfortic (SLE only)
Marginal R2 = 0.06 | Conditional R2 = 0.93 | Interaction q = 0.99
100000
75000
50000
25000
0
T1
T2
T3
T4
T5
T6
Drug
No
Yes

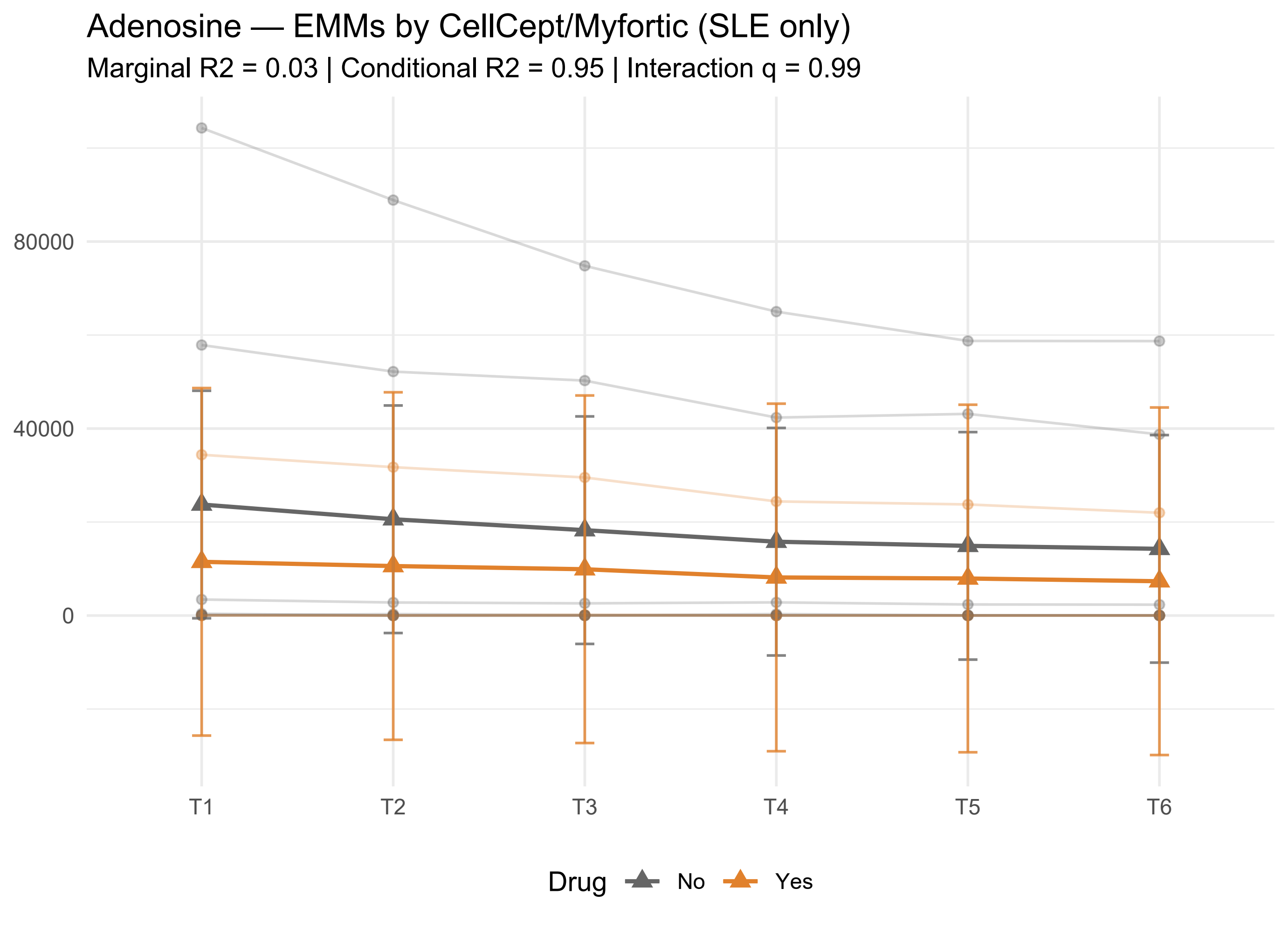

Adenosine — EMMs by CellCept/Myfortic (SLE only)
Marginal R2 = 0.03 | Conditional R2 = 0.95 | Interaction q = 0.99
80000
40000
0
T1
T2
T3
T4
T5
T6
Drug
No
Yes

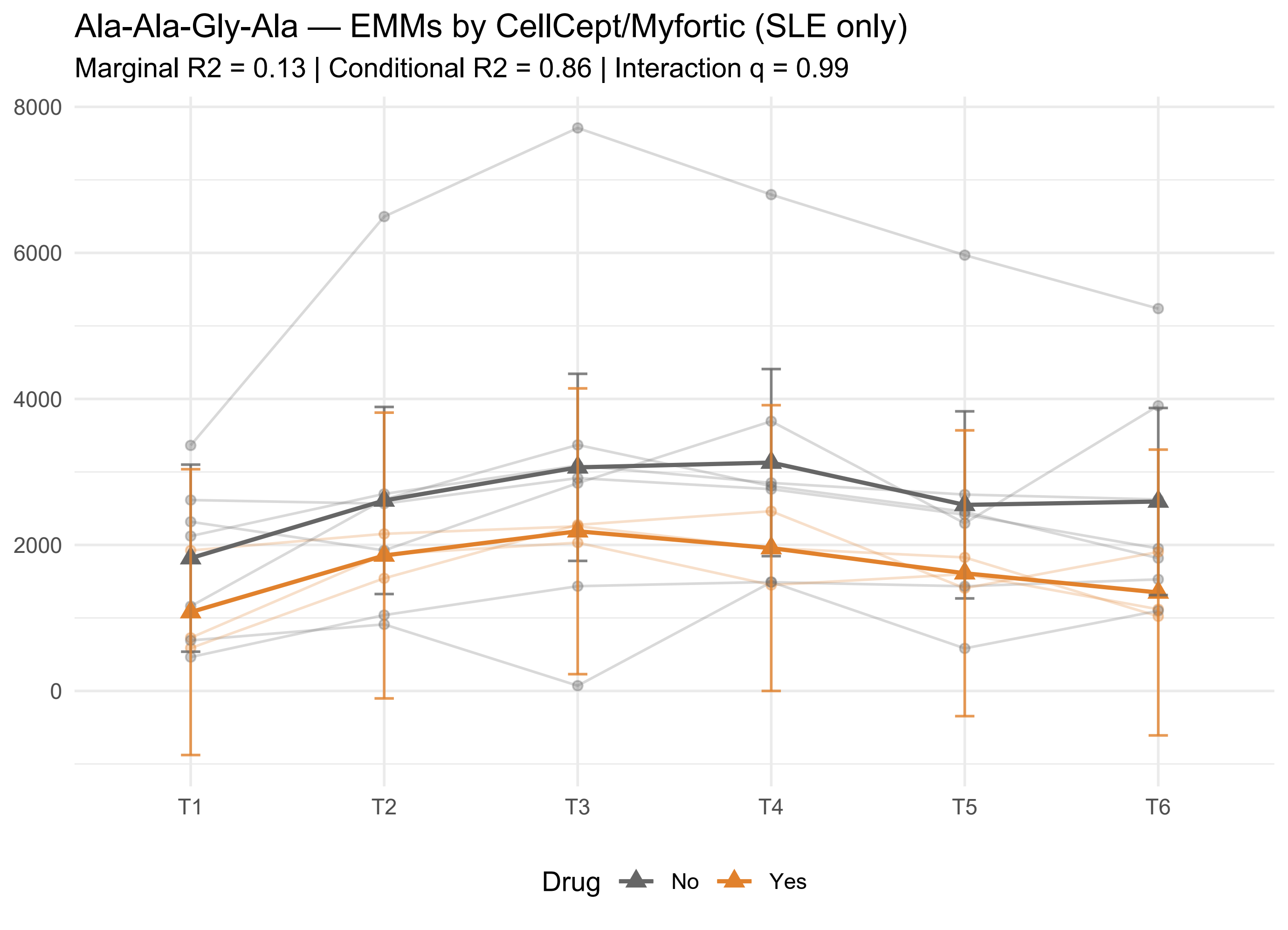
Ala-Ala-Gly-Ala — EMMs by CellCept/Myfortic (SLE only)
Marginal R2 = 0.13 | Conditional R2 = 0.86 | Interaction q = 0.99
8000
6000
4000
2000
0
T1
T2
T3
T4
T5
T6
Drug
No
Yes

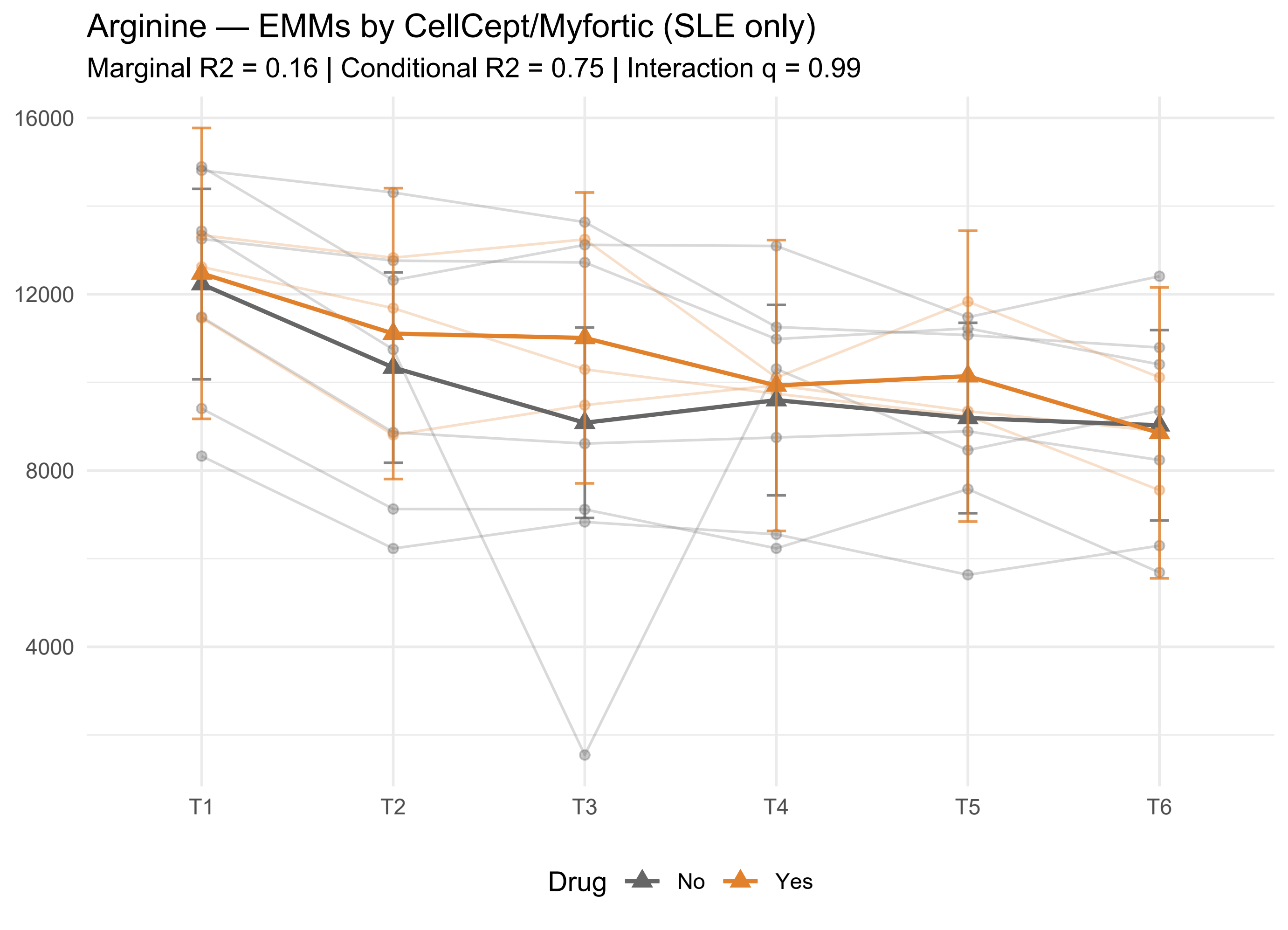

Arginine — EMMs by CellCept/Myfortic (SLE only)
Marginal R2 = 0.16 | Conditional R2 = 0.75 | Interaction q = 0.99
16000
12000
8000
4000
T1
T2
T3
T4
T5
T6
Drug
No
Yes

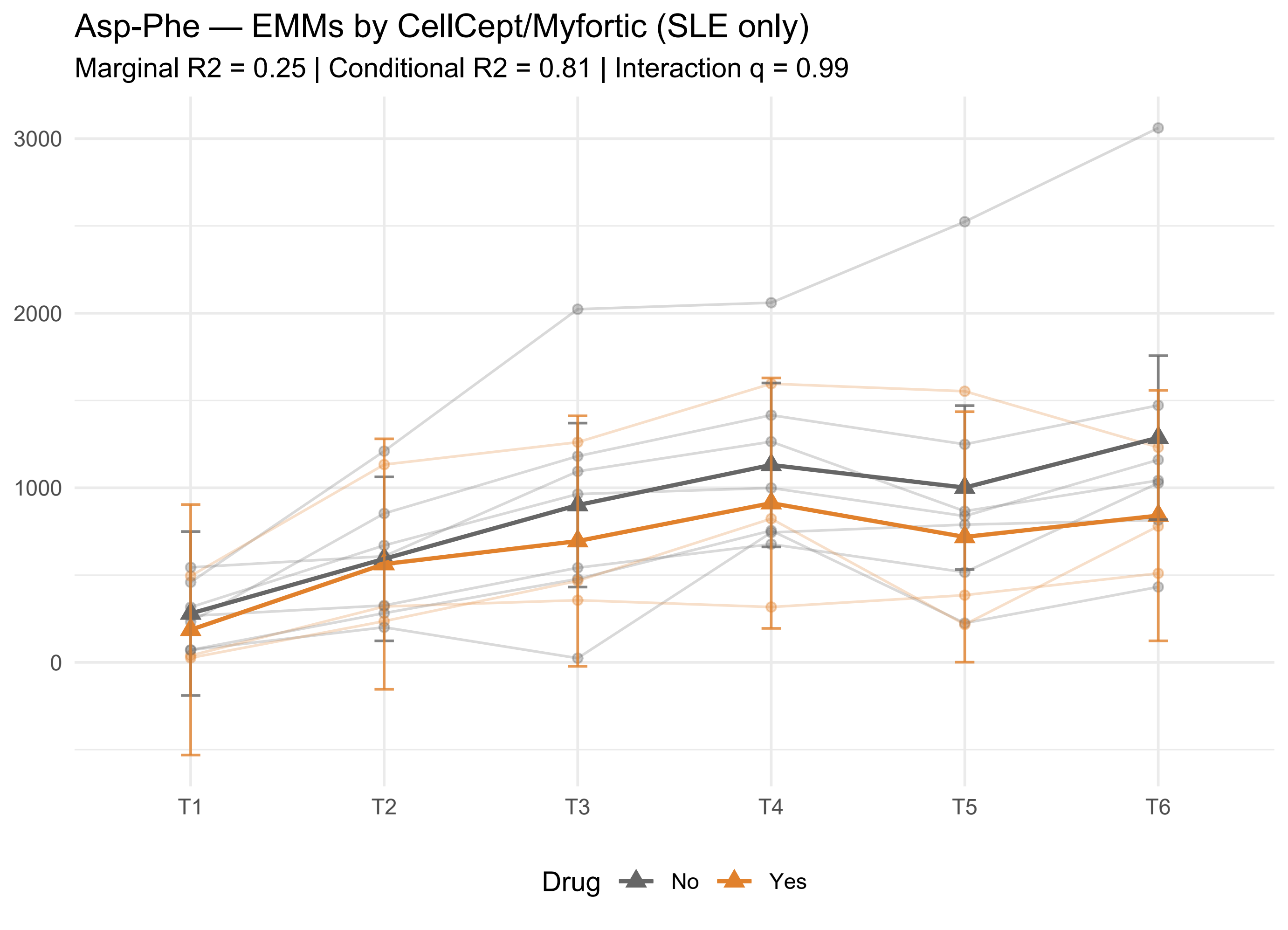

Asp-Phe — EMMs by CellCept/Myfortic (SLE only)
Marginal R2 = 0.25 | Conditional R2 = 0.81 | Interaction q = 0.99
3000
2000
1000
0
T1
T2
T3
T4
T5
T6
Drug
No
Yes

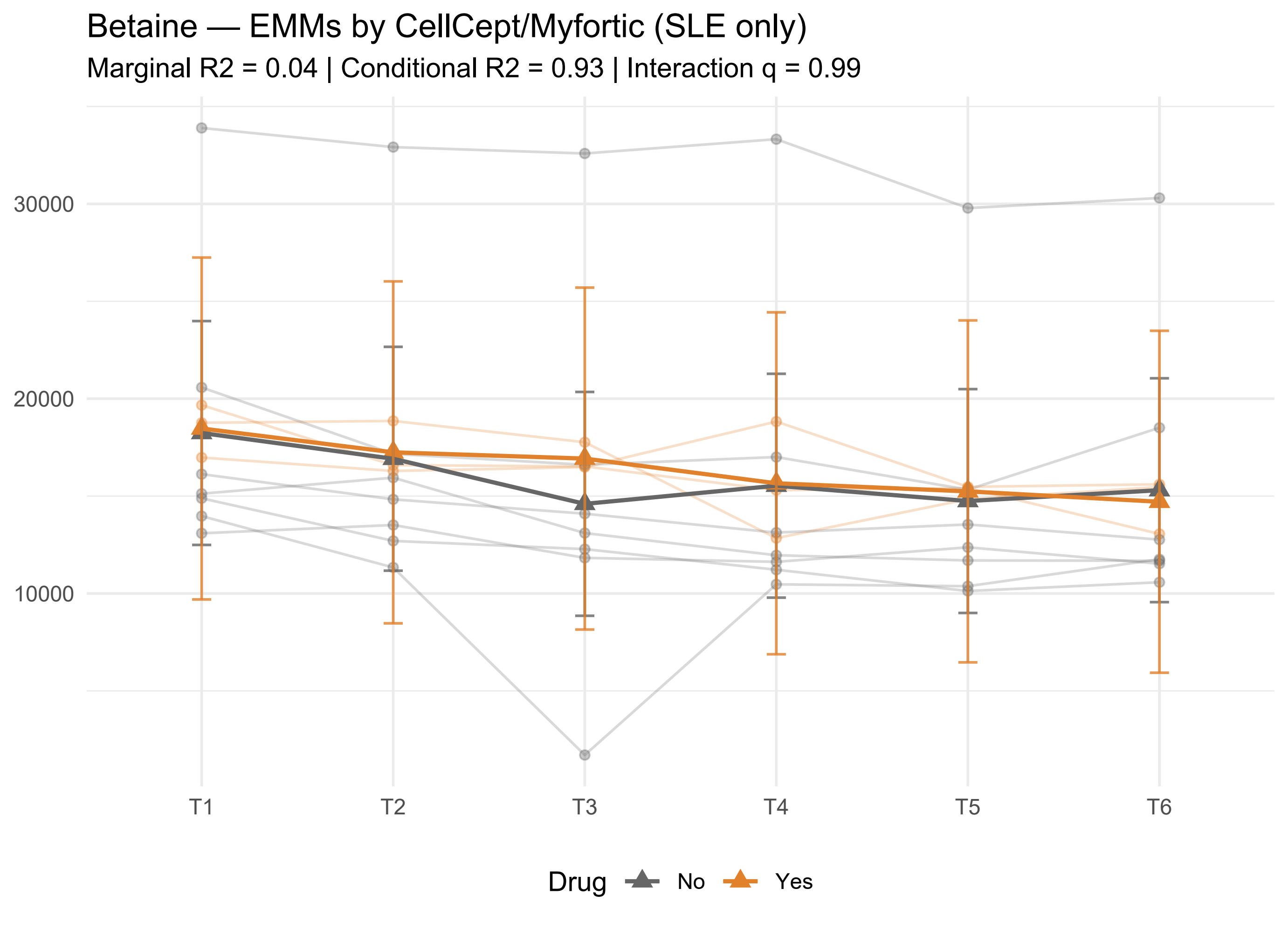

Betaine — EMMs by CellCept/Myfortic (SLE only)
Marginal R2 = 0.04 | Conditional R2 = 0.93 | Interaction q = 0.99
30000
20000
10000
T1
T2
T3
T4
T5
T6
Drug
No
Yes

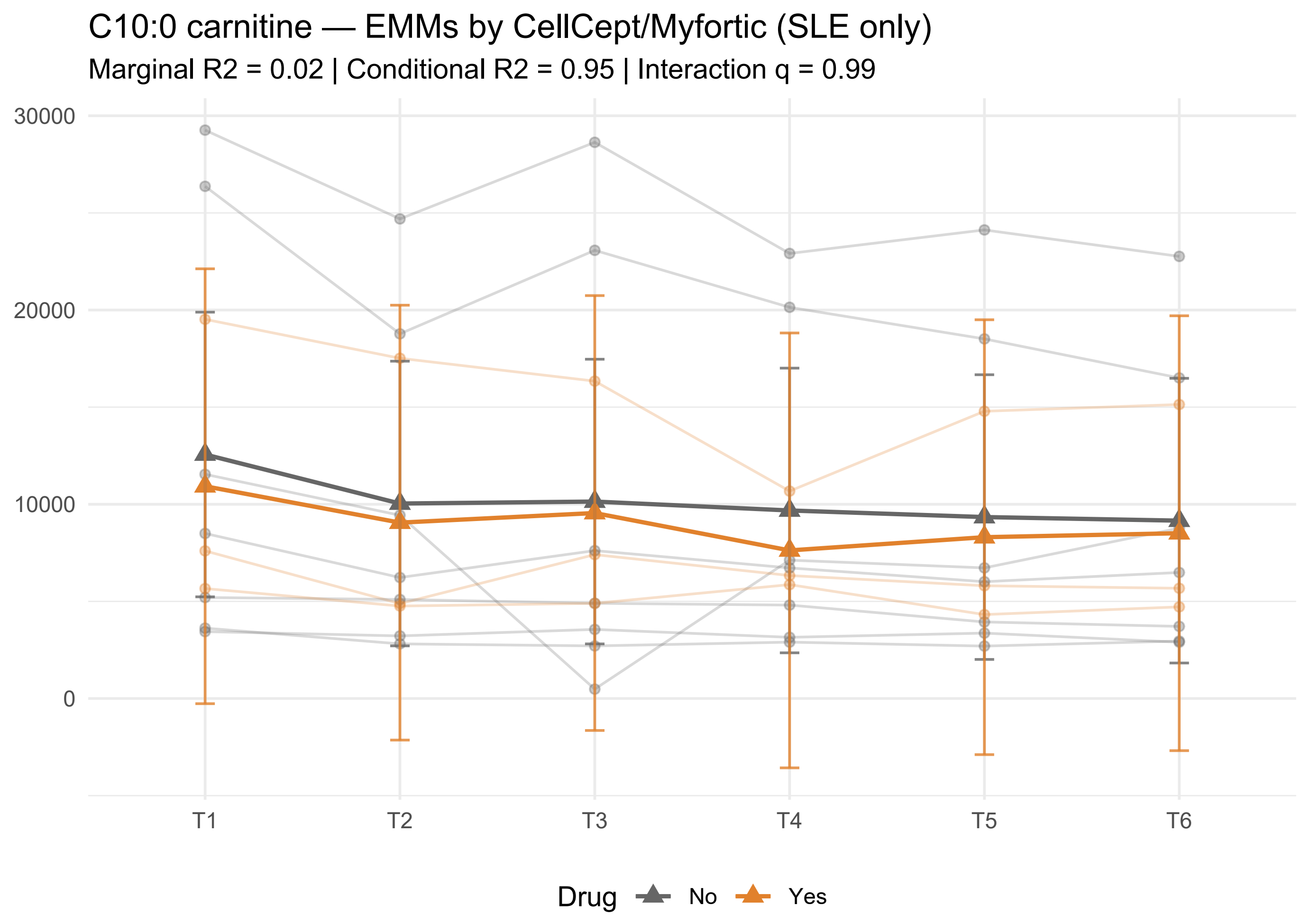
C10:0 carnitine — EMMs by CellCept/Myfortic (SLE only)
Marginal R2 = 0.02 | Conditional R2 = 0.95 | Interaction q = 0.99
30000
20000
10000
0
T1
T2
T3
T4
T5
T6
Drug
No
Yes

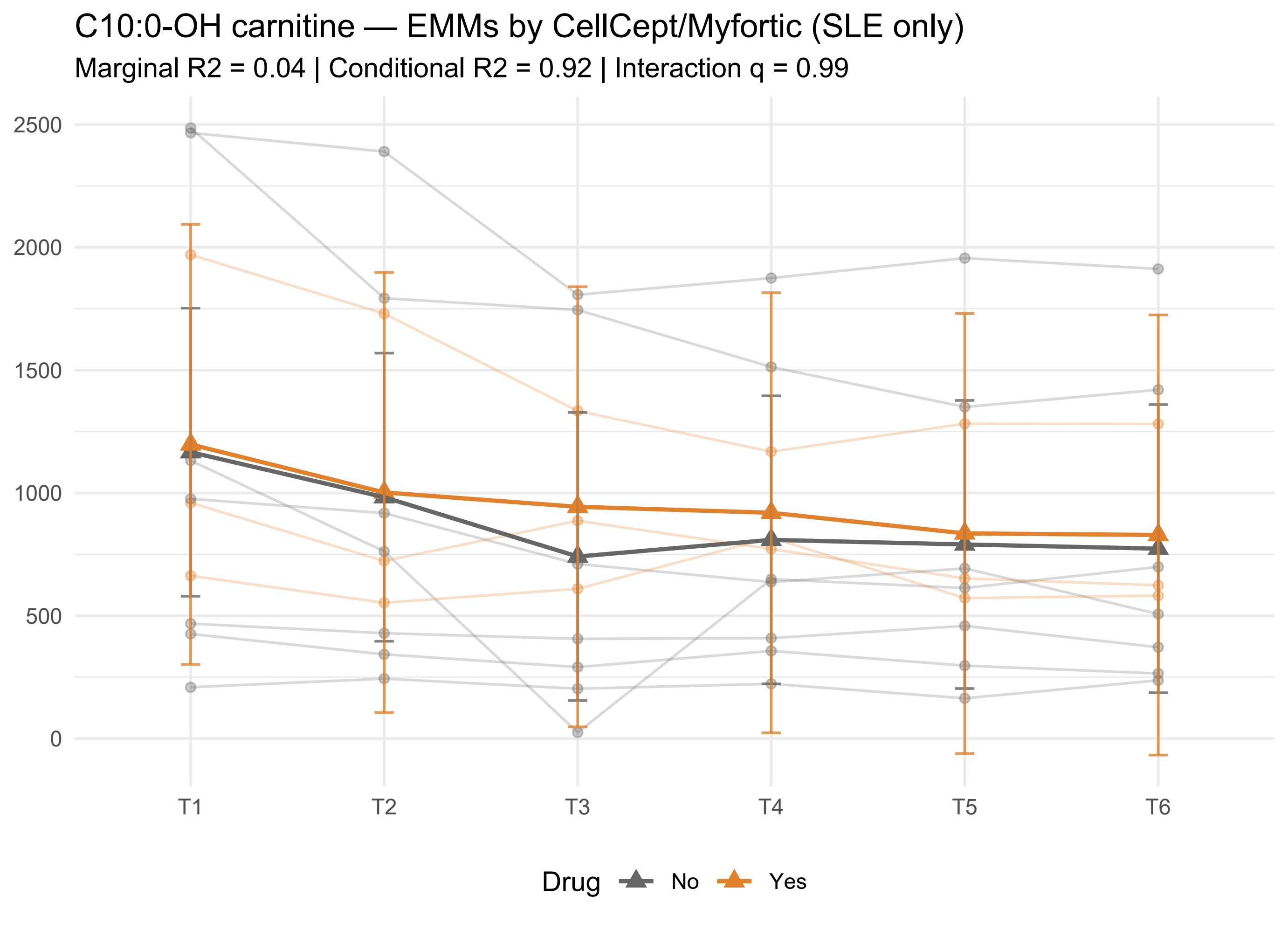
C10:0-OH carnitine — EMMs by CellCept/Myfortic (SLE only)
Marginal R2 = 0.04 | Conditional R2 = 0.92 | Interaction q = 0.99
2500
2000
1500
1000
500
0
T1
T2
T3
T4
T5
T6
Drug
No
Yes

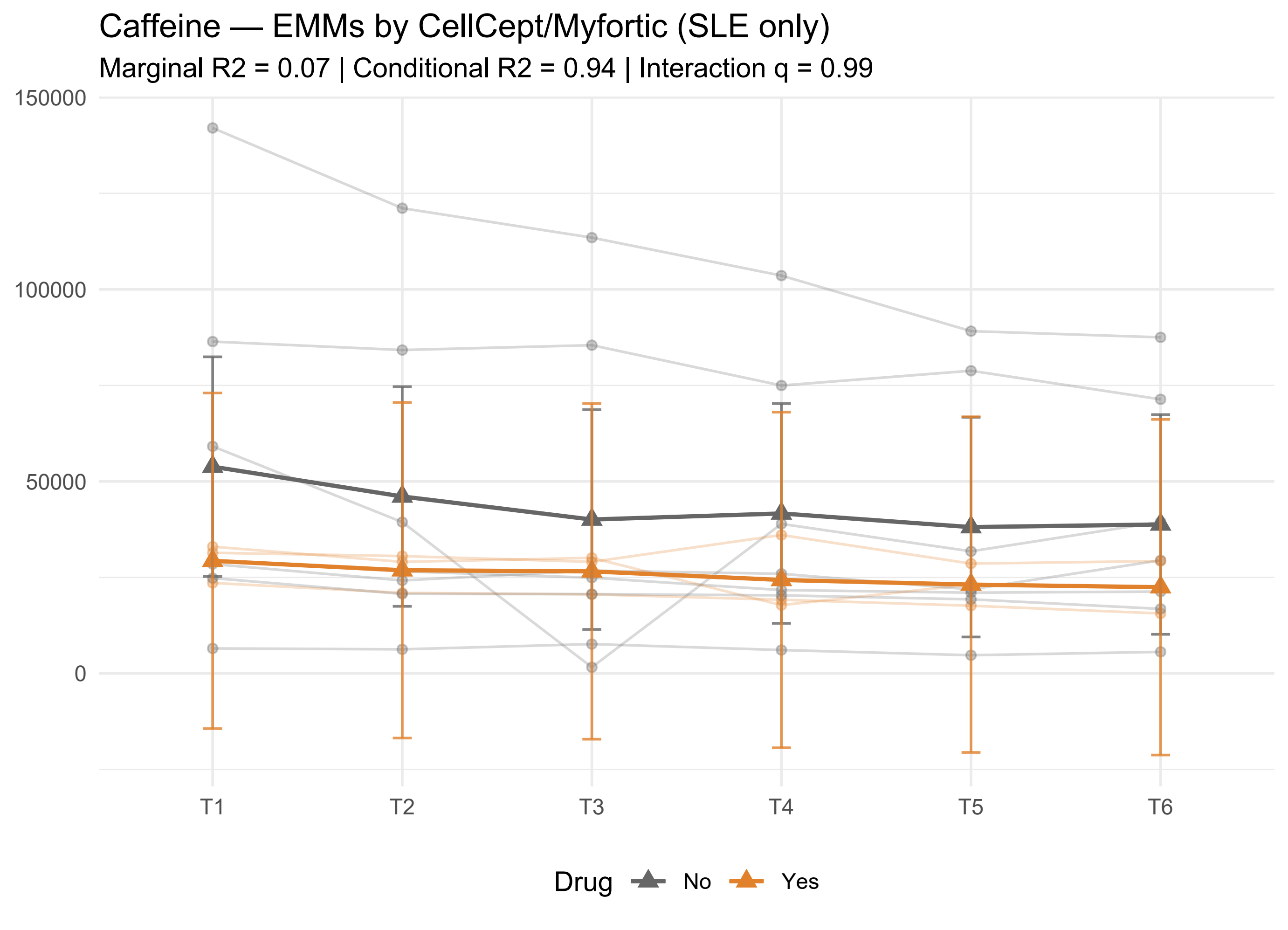
Caffeine — EMMs by CellCept/Myfortic (SLE only)
Marginal R2 = 0.07 | Conditional R2 = 0.94 | Interaction q = 0.99
150000
100000
50000
0
T1
T2
T3
T4
T5
T6
Drug
No
Yes

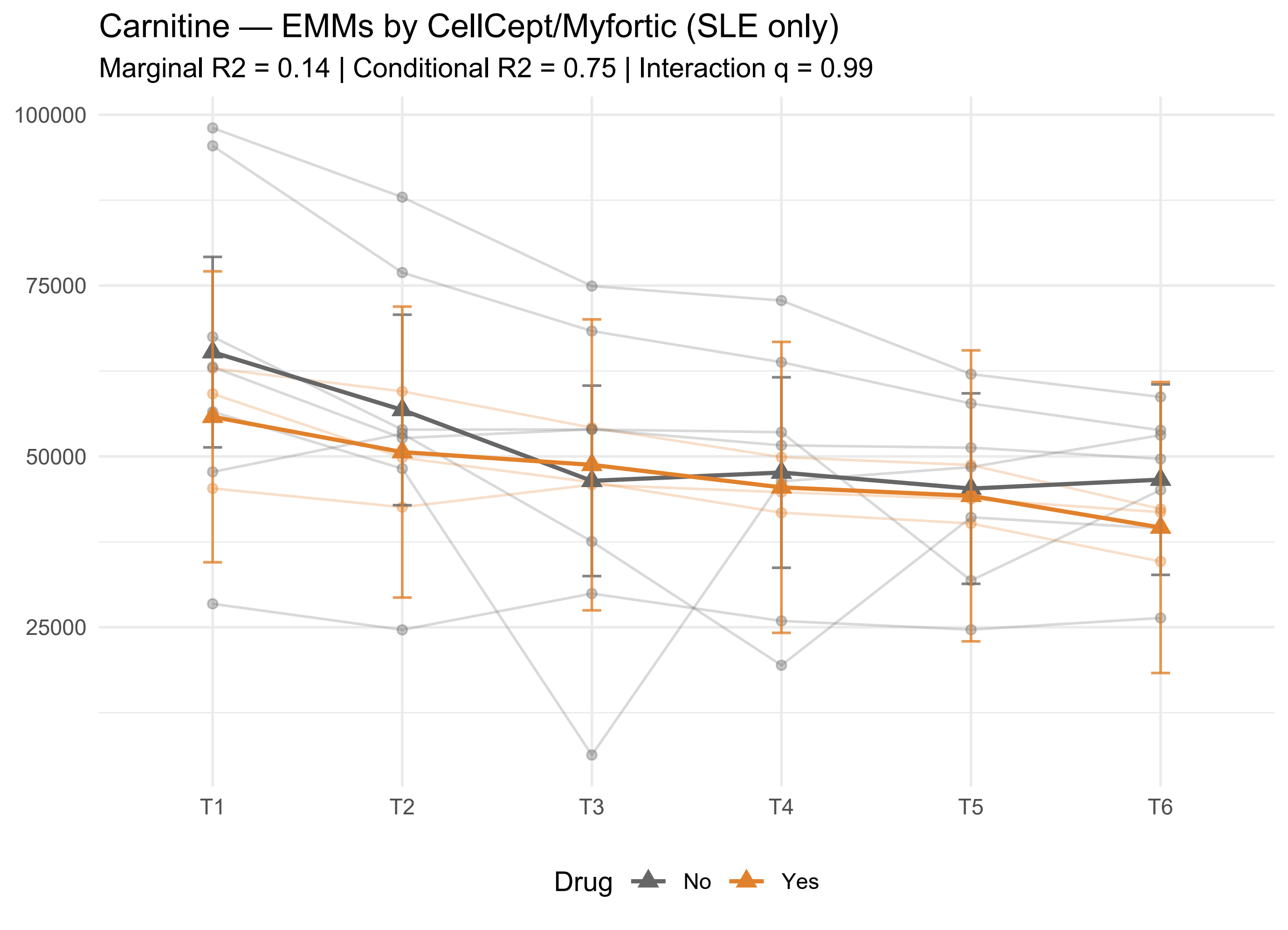

Carnitine — EMMs by CellCept/Myfortic (SLE only)
Marginal R2 = 0.14 | Conditional R2 = 0.75 | Interaction q = 0.99
100000
75000
50000
25000
T1
T2
T3
T4
T5
T6
Drug
No
Yes

Chlorpheniramine Maleate (Trigonelline) — EMMs by CellCept/Myfortic (SLE only)

Marginal R2 = 0.07 | Conditional R2 = 0.91 | Interaction q = 0.99

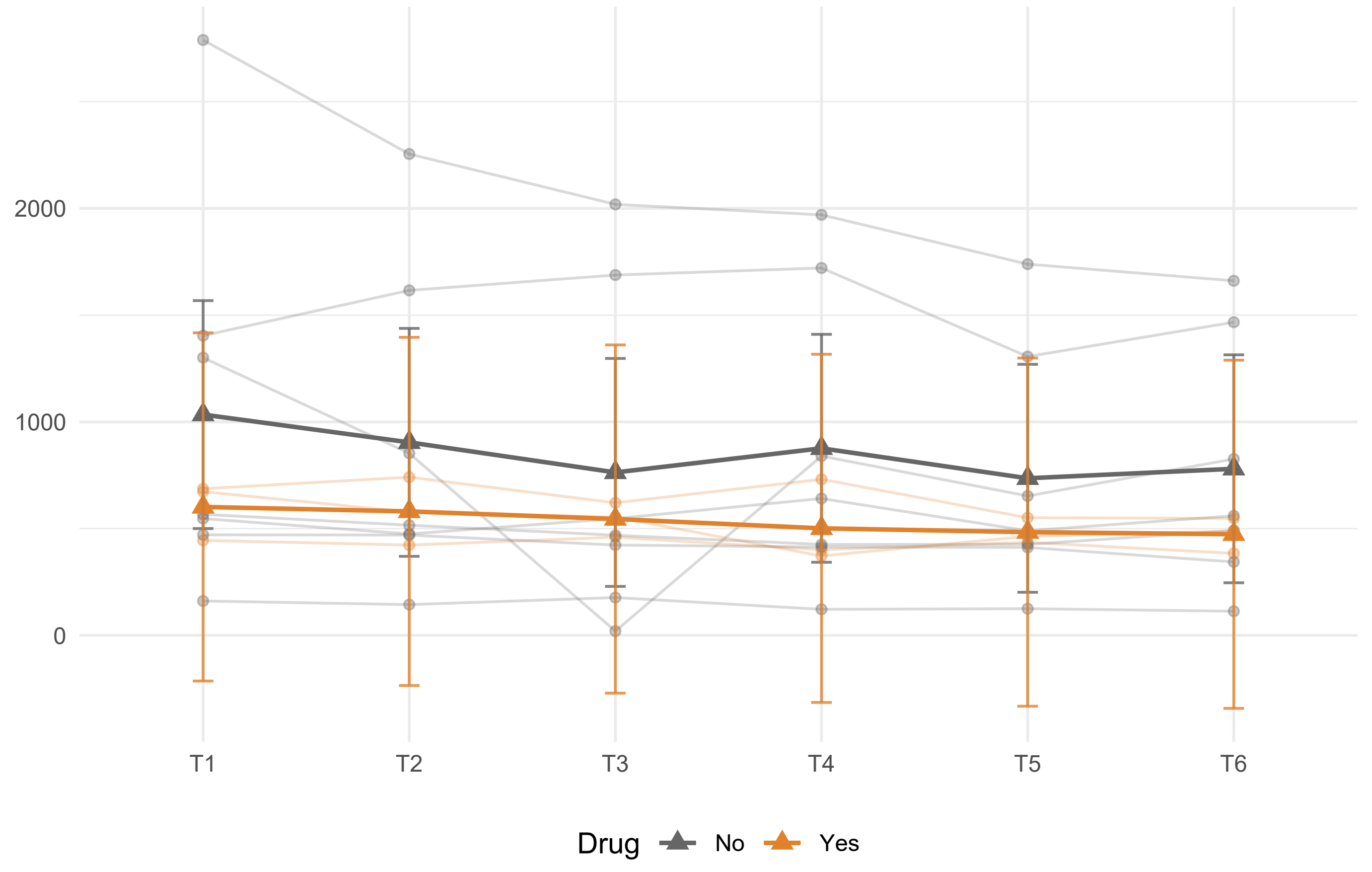

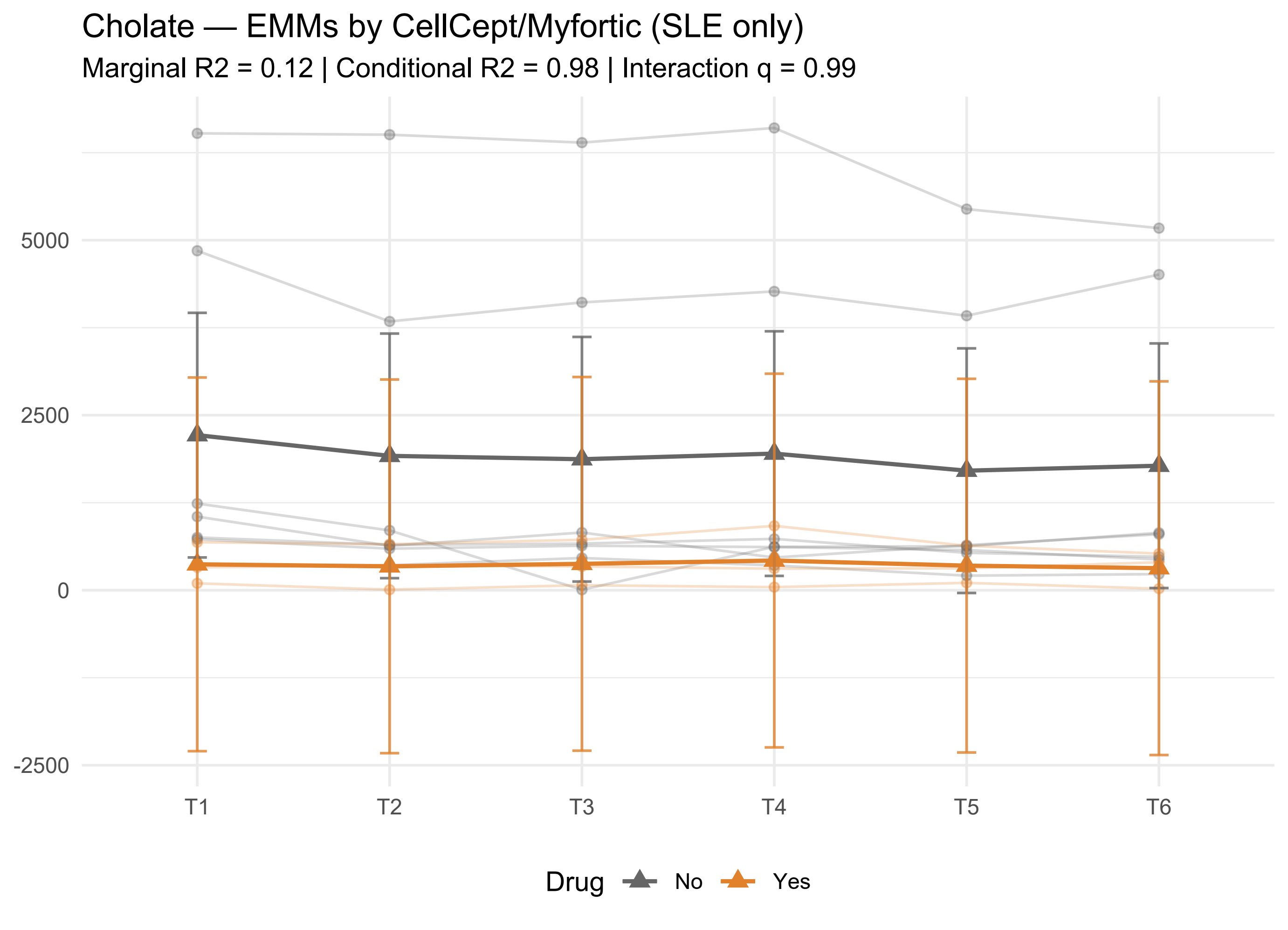

Cholate — EMMs by CellCept/Myfortic (SLE only)
Marginal R2 = 0.12 | Conditional R2 = 0.98 | Interaction q = 0.99
5000
2500
0
-2500
T1
T2
T3
T4
T5
T6
Drug
No
Yes

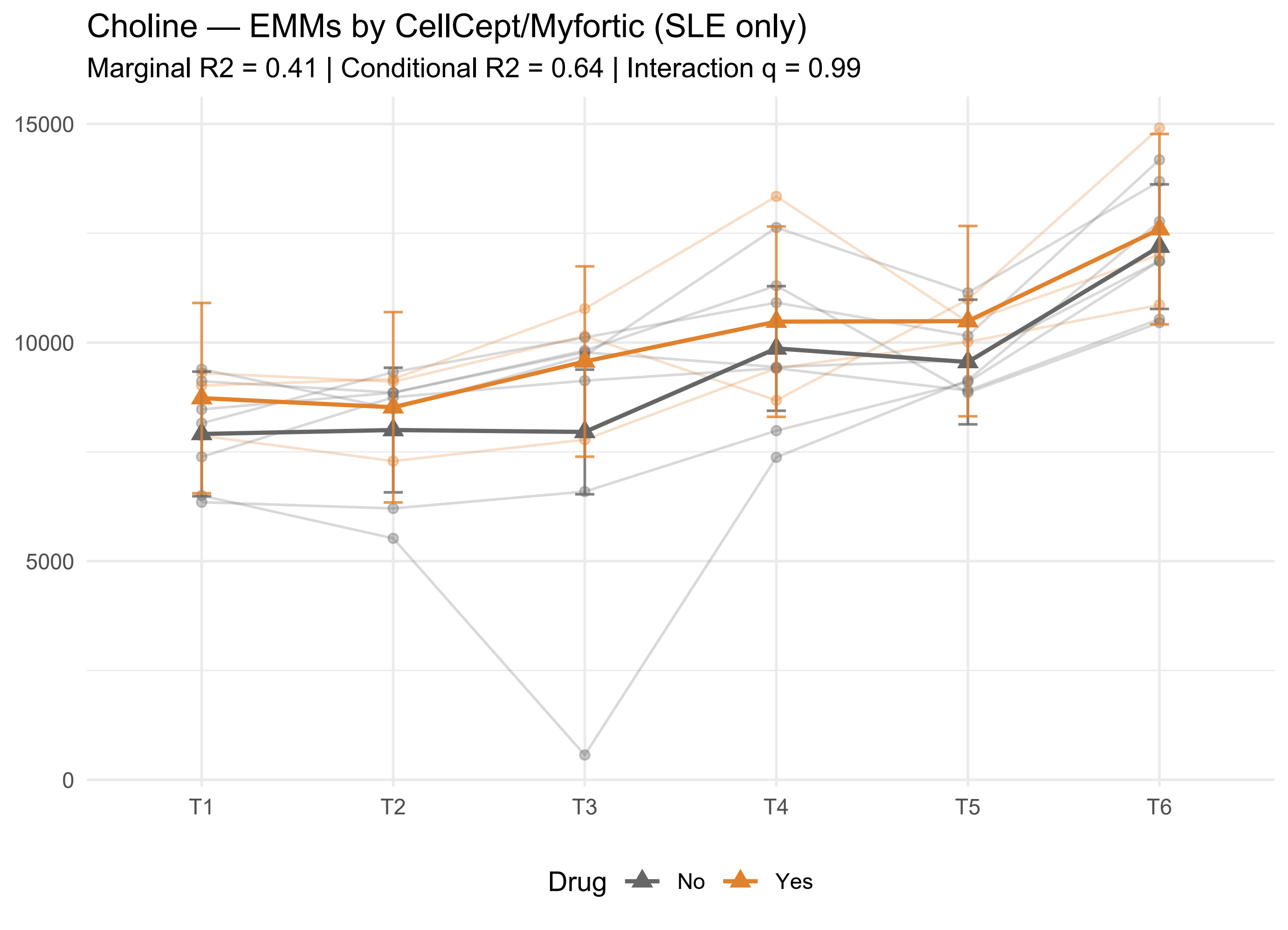

Choline — EMMs by CellCept/Myfortic (SLE only)
Marginal R2 = 0.41 | Conditional R2 = 0.64 | Interaction q = 0.99
15000
10000
5000
0
T1
T2
T3
T4
T5
T6
Drug
No
Yes

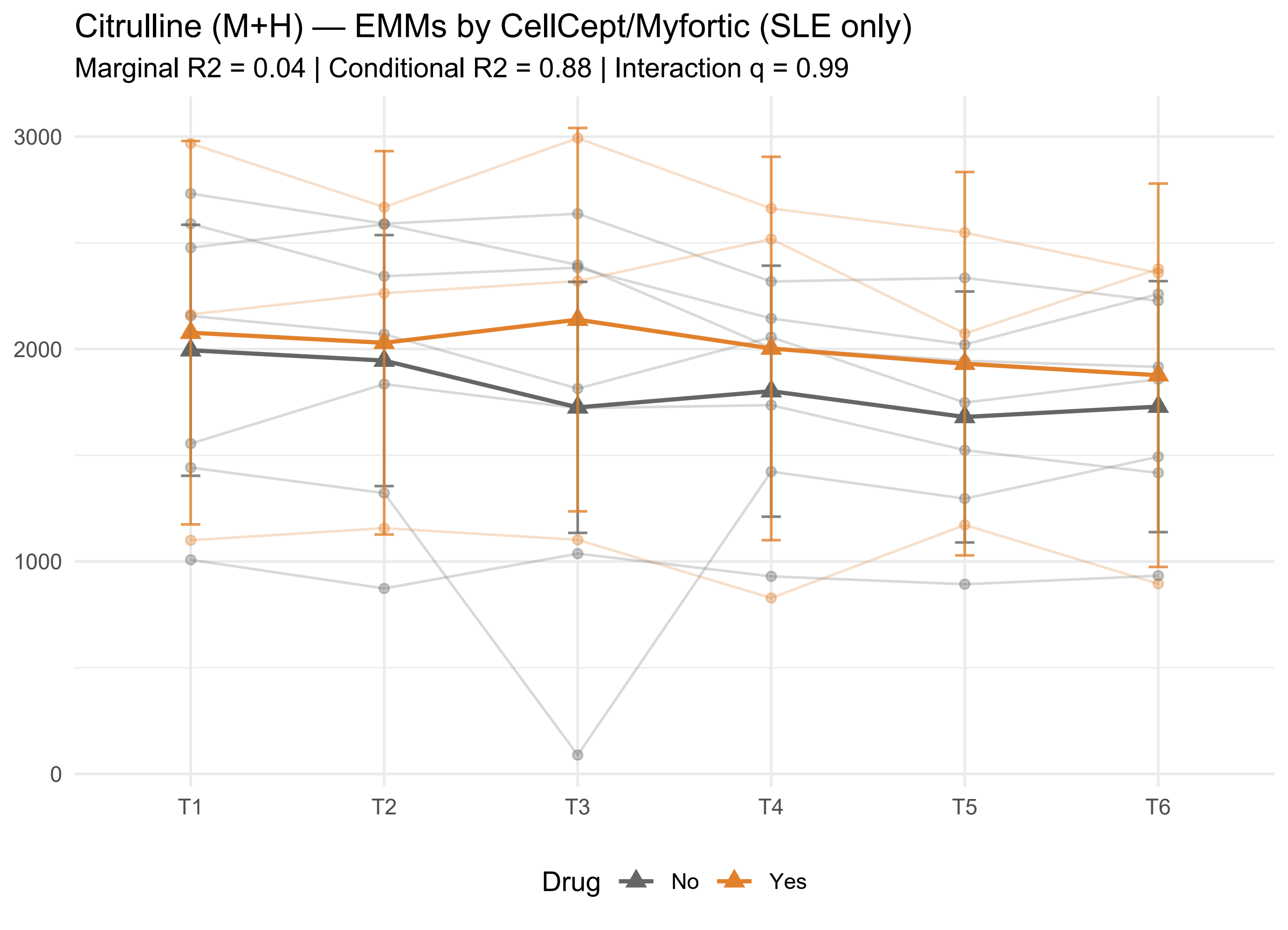

Citrulline (M+H) — EMMs by CellCept/Myfortic (SLE only)
Marginal R2 = 0.04 | Conditional R2 = 0.88 | Interaction q = 0.99
3000
2000
1000
0
T1
T2
T3
T4
T5
T6
Drug
No
Yes

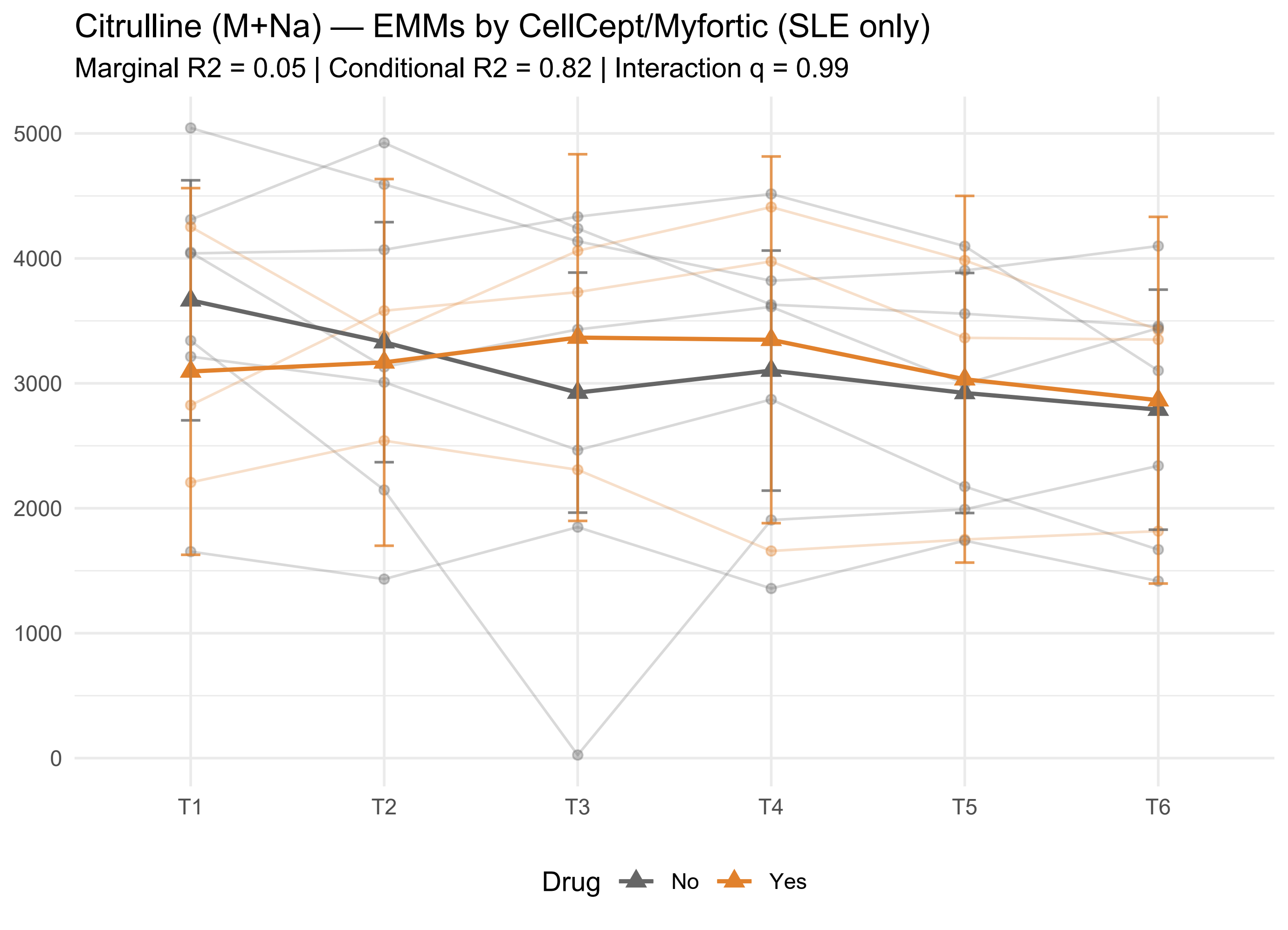
Citrulline (M+Na) — EMMs by CellCept/Myfortic (SLE only)
Marginal R2 = 0.05 | Conditional R2 = 0.82 | Interaction q = 0.99
5000
4000
3000
2000
1000
0
T1
T2
T3
T4
T5
T6
Drug
No
Yes

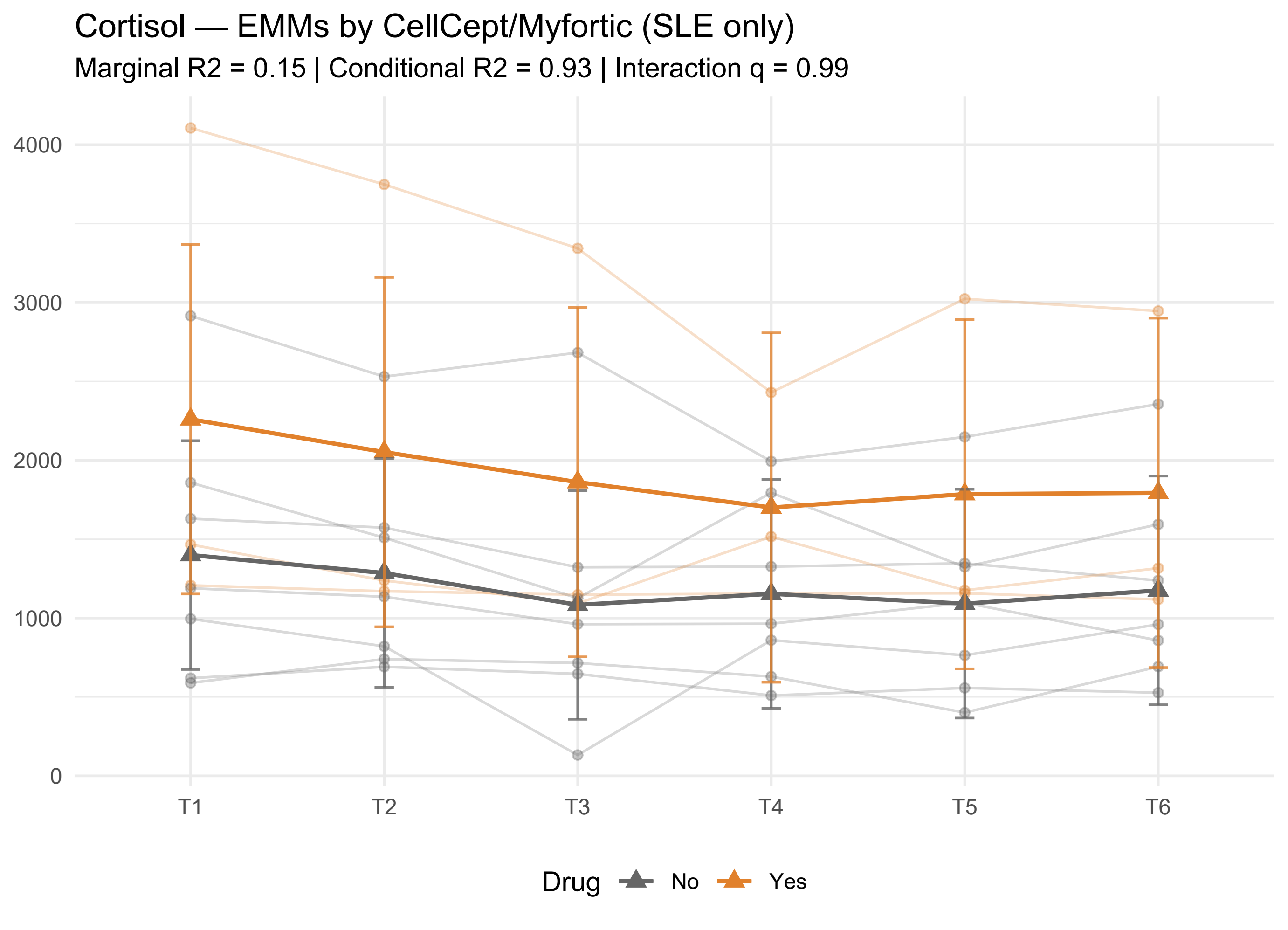

Cortisol — EMMs by CellCept/Myfortic (SLE only)
Marginal R2 = 0.15 | Conditional R2 = 0.93 | Interaction q = 0.99
4000
3000
2000
1000
0
T1
T2
T3
T4
T5
T6
Drug
No
Yes

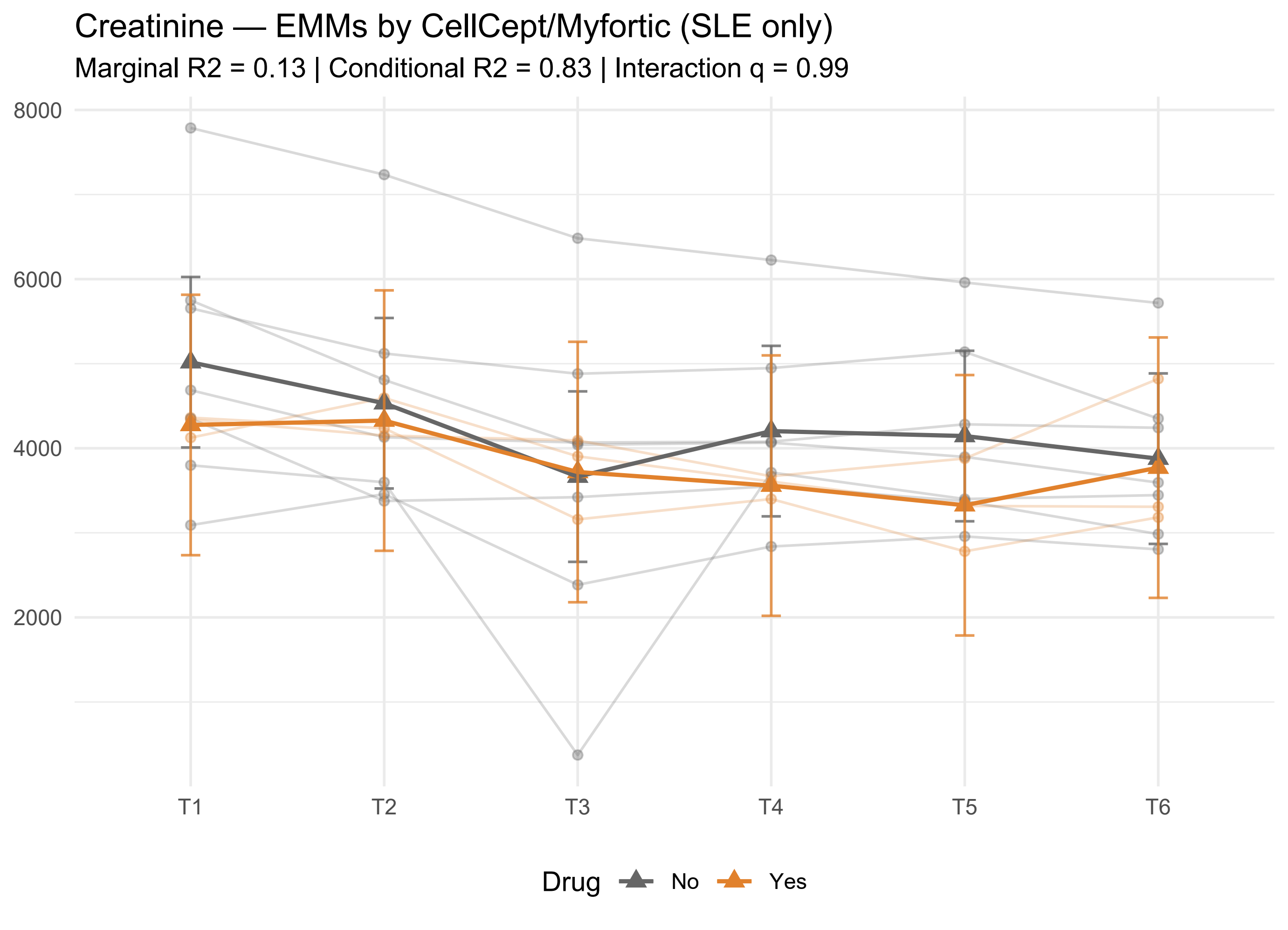

Creatinine — EMMs by CellCept/Myfortic (SLE only)
Marginal R2 = 0.13 | Conditional R2 = 0.83 | Interaction q = 0.99
8000
6000
4000
2000
T1
T2
T3
T4
T5
T6
Drug
No
Yes

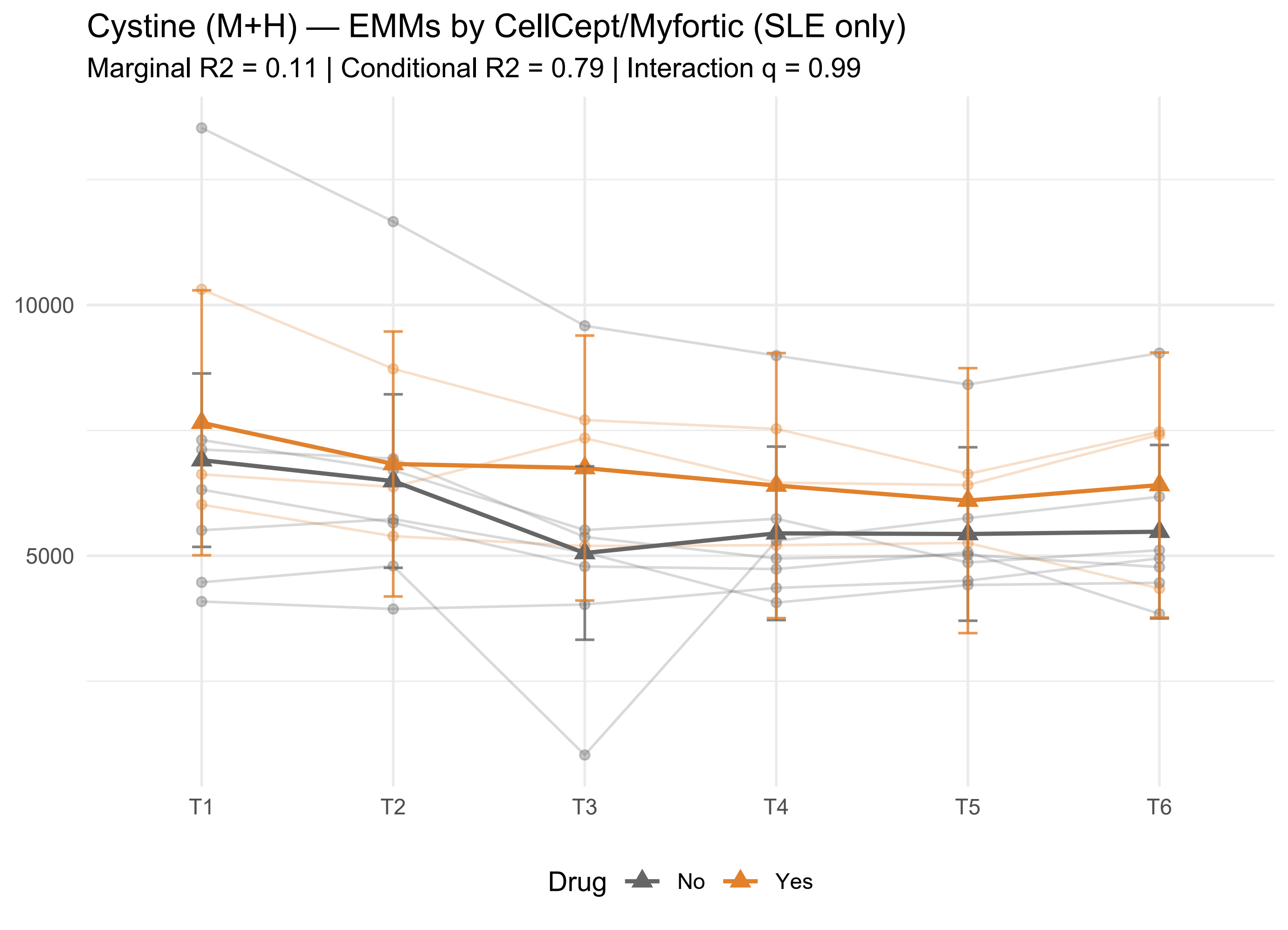

Cystine (M+H) — EMMs by CellCept/Myfortic (SLE only)
Marginal R2 = 0.11 | Conditional R2 = 0.79 | Interaction q = 0.99
10000
5000
T1
T2
T3
T4
T5
T6
Drug
No
Yes

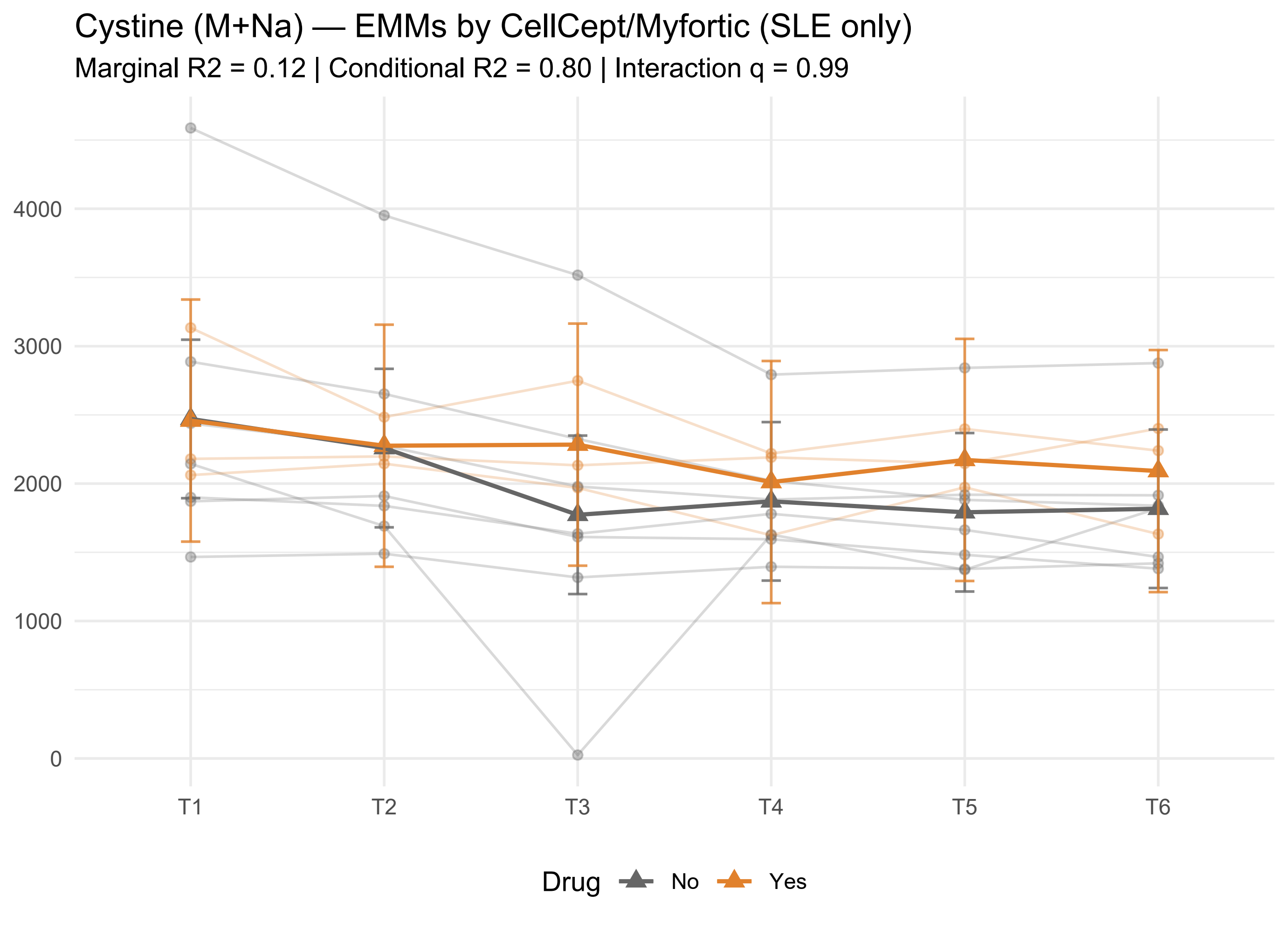

Cystine (M+Na) — EMMs by CellCept/Myfortic (SLE only)
Marginal R2 = 0.12 | Conditional R2 = 0.80 | Interaction q = 0.99
4000
3000
2000
1000
0
T1
T2
T3
T4
T5
T6
Drug
No
Yes

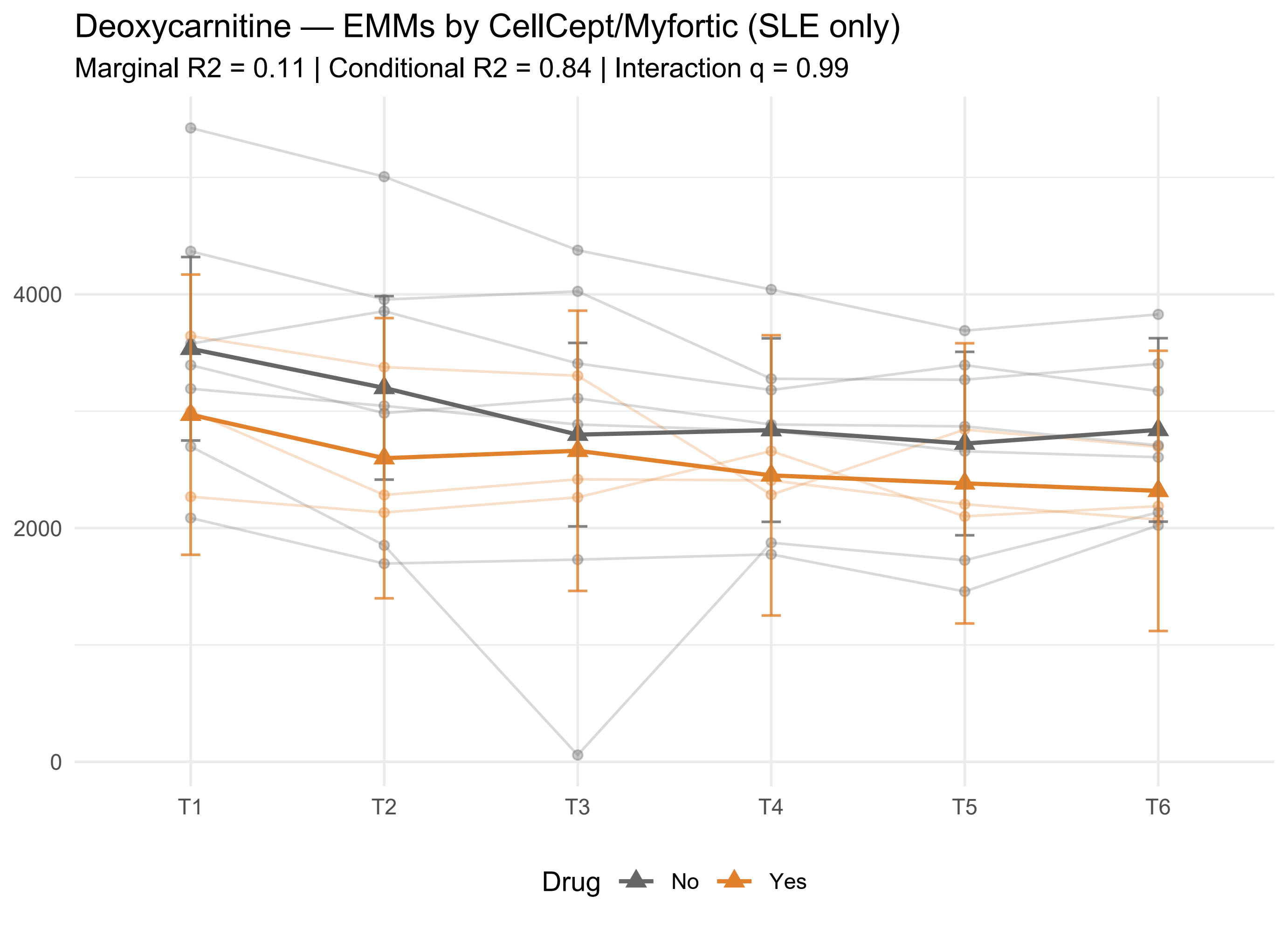
Deoxycarnitine — EMMs by CellCept/Myfortic (SLE only)
Marginal R2 = 0.11 | Conditional R2 = 0.84 | Interaction q = 0.99
4000
2000
0
T1
T2
T3
T4
T5
T6
Drug
No
Yes

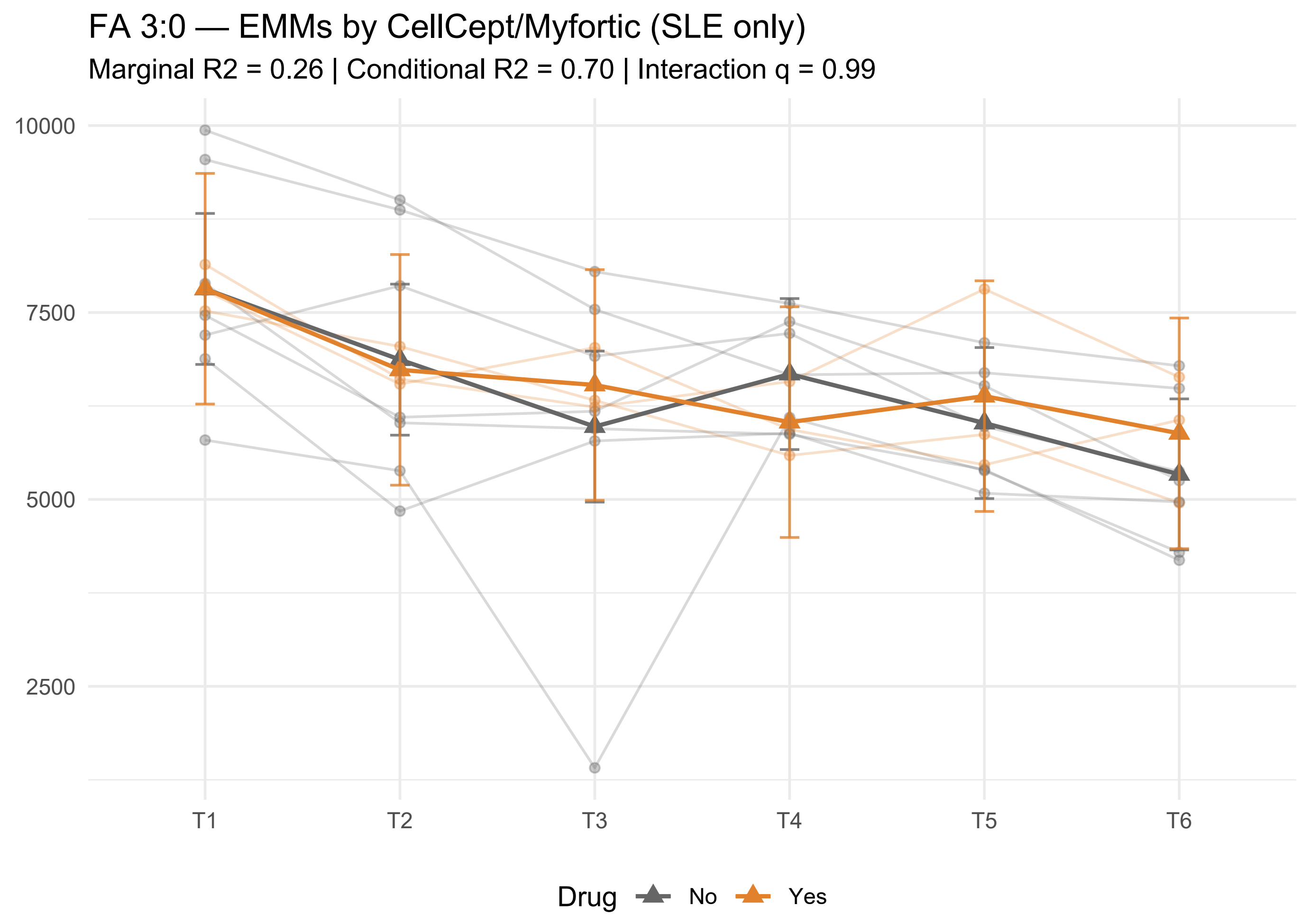
FA 3:0 — EMMs by CellCept/Myfortic (SLE only)
Marginal R2 = 0.26 | Conditional R2 = 0.70 | Interaction q = 0.99
10000
7500
5000
2500
T1
T2
T3
T4
T5
T6
Drug
No
Yes

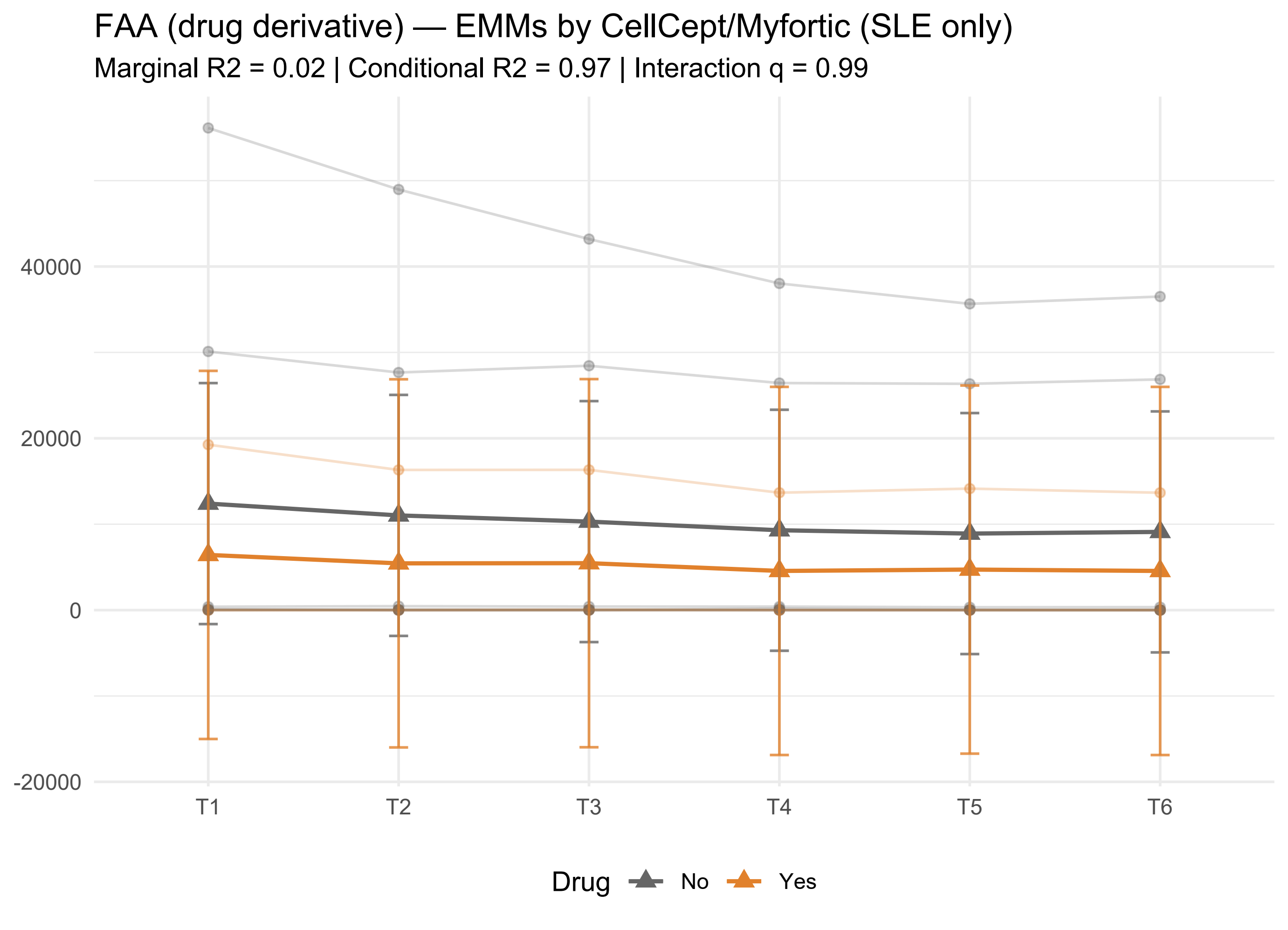
FAA (drug derivative) — EMMs by CellCept/Myfortic (SLE only)
Marginal R2 = 0.02 | Conditional R2 = 0.97 | Interaction q = 0.99
40000
20000
0
-20000
T1
T2
T3
T4
T5
T6
Drug
No
Yes

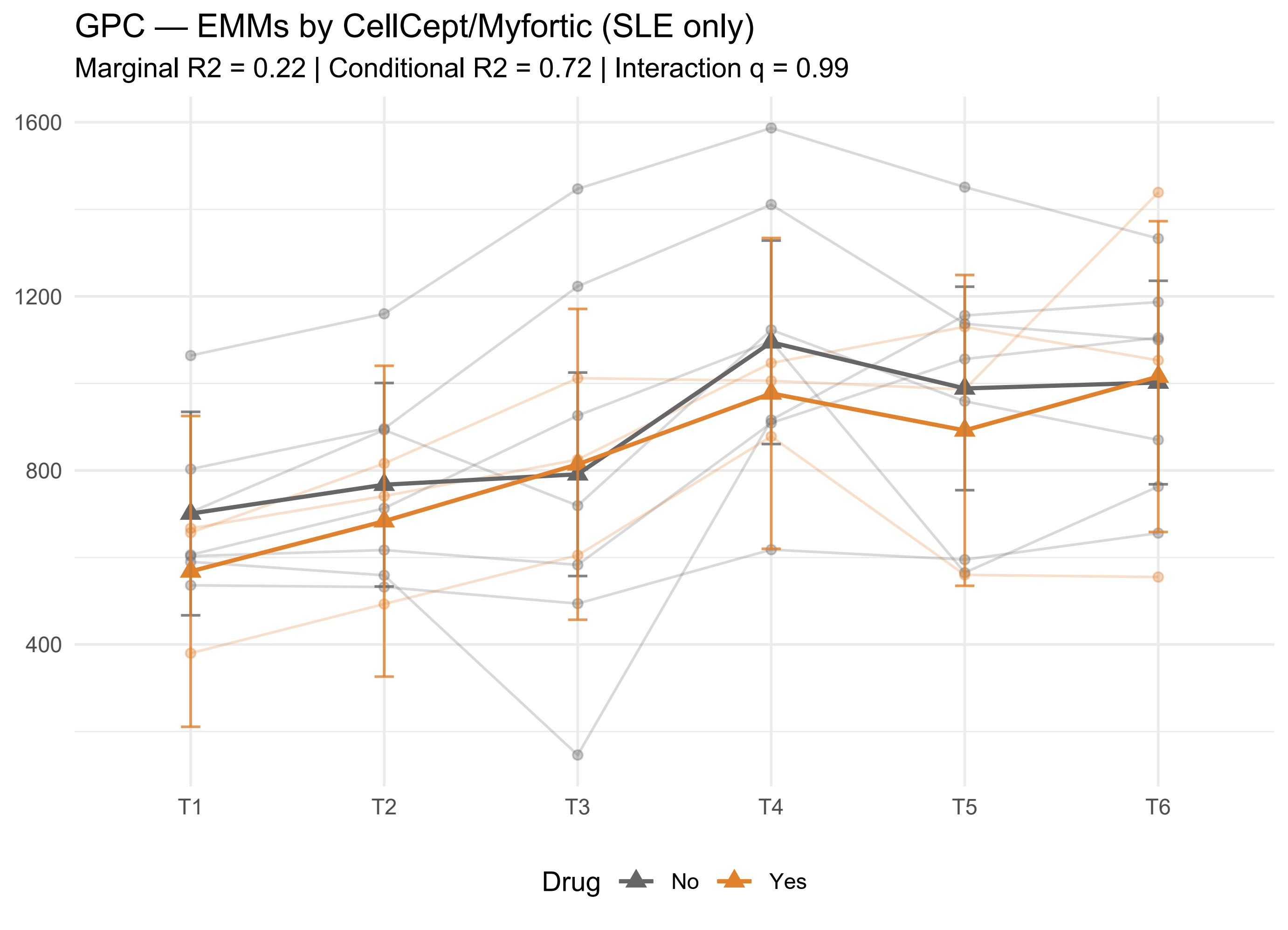
GPC — EMMs by CellCept/Myfortic (SLE only)
Marginal R2 = 0.22 | Conditional R2 = 0.72 | Interaction q = 0.99
1600
1200
800
400
T1
T2
T3
T4
T5
T6
Drug
No
Yes

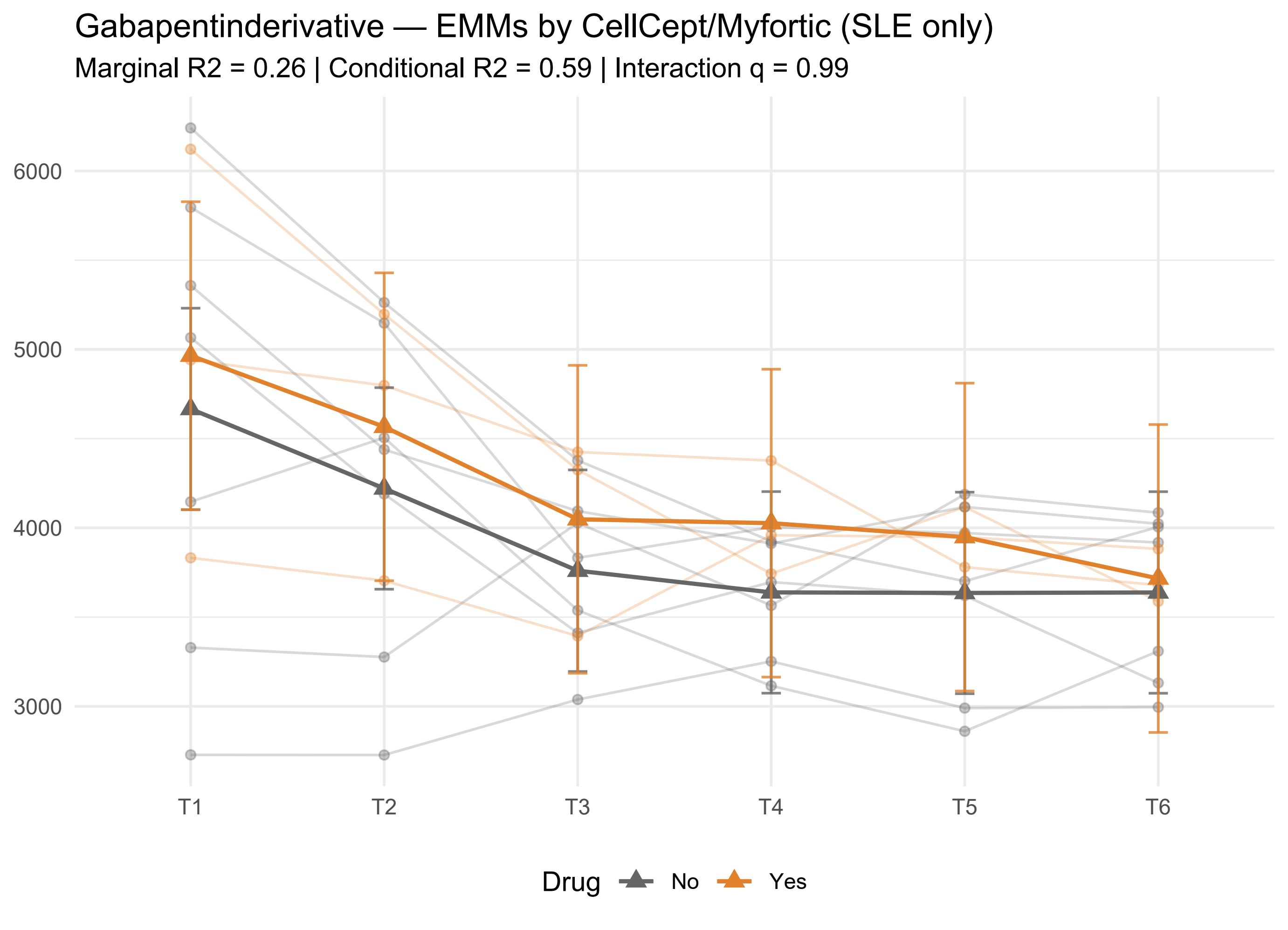

Gabapentinderivative — EMMs by CellCept/Myfortic (SLE only)
Marginal R2 = 0.26 | Conditional R2 = 0.59 | Interaction q = 0.99
6000
5000
4000
3000
T1
T2
T3
T4
T5
T6
Drug
No
Yes

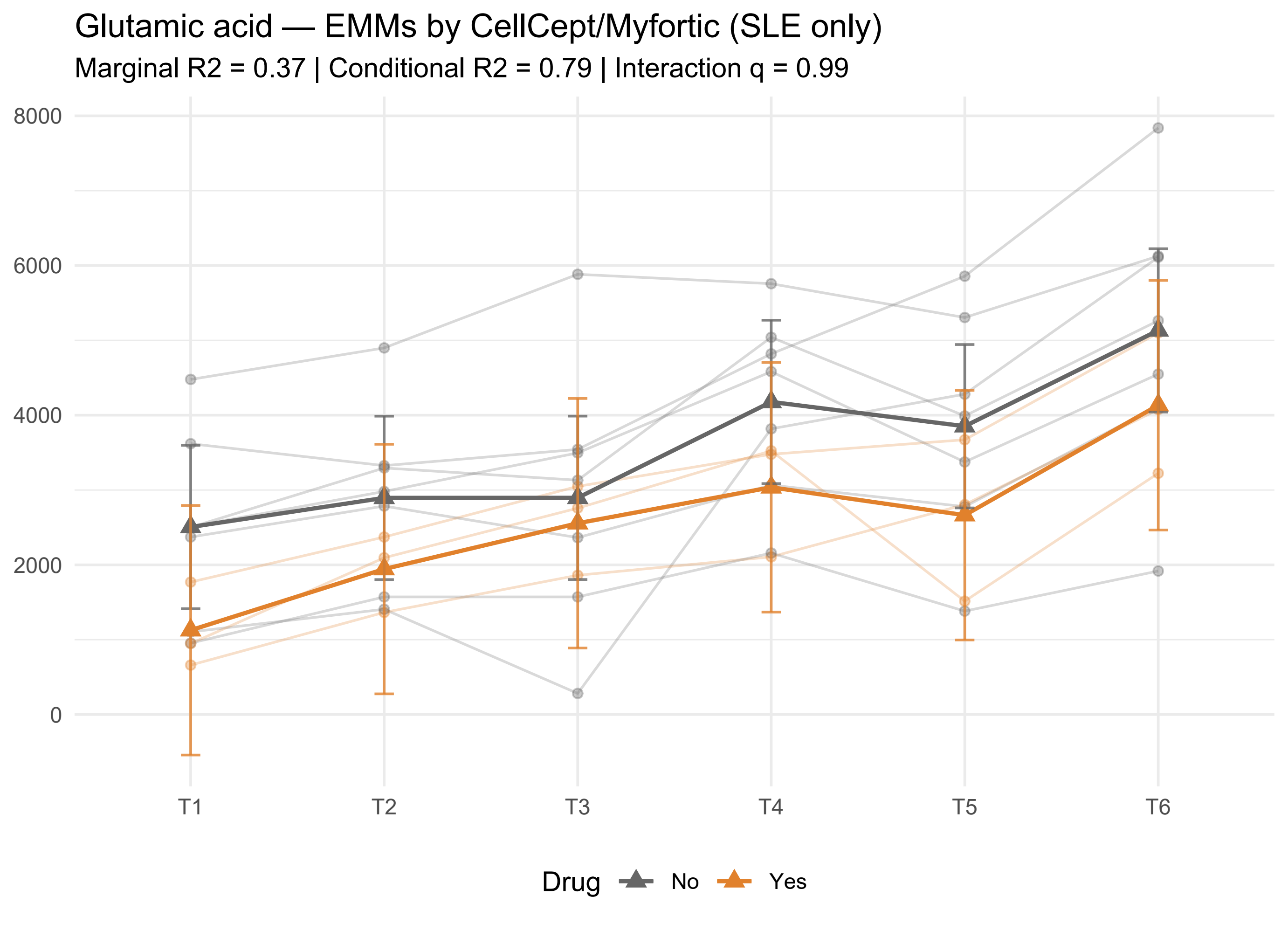

Glutamic acid — EMMs by CellCept/Myfortic (SLE only)
Marginal R2 = 0.37 | Conditional R2 = 0.79 | Interaction q = 0.99
8000
6000
4000
2000
0
T1
T2
T3
T4
T5
T6
Drug
No
Yes

Glutamine — EMMs by CellCept/Myfortic (SLE only)

Marginal R2 = 0.25 | Conditional R2 = 0.50 | Interaction q = 0.99

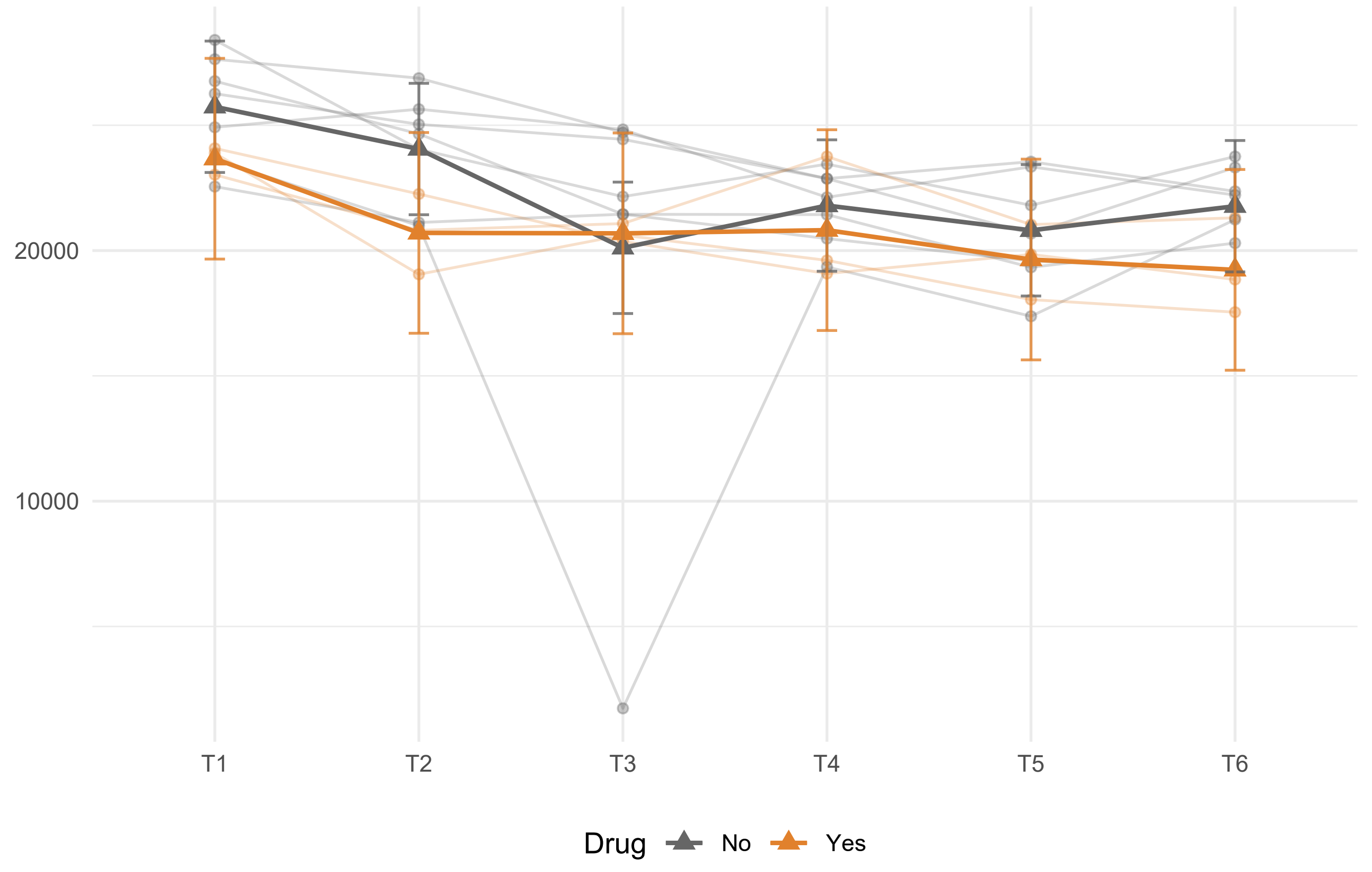

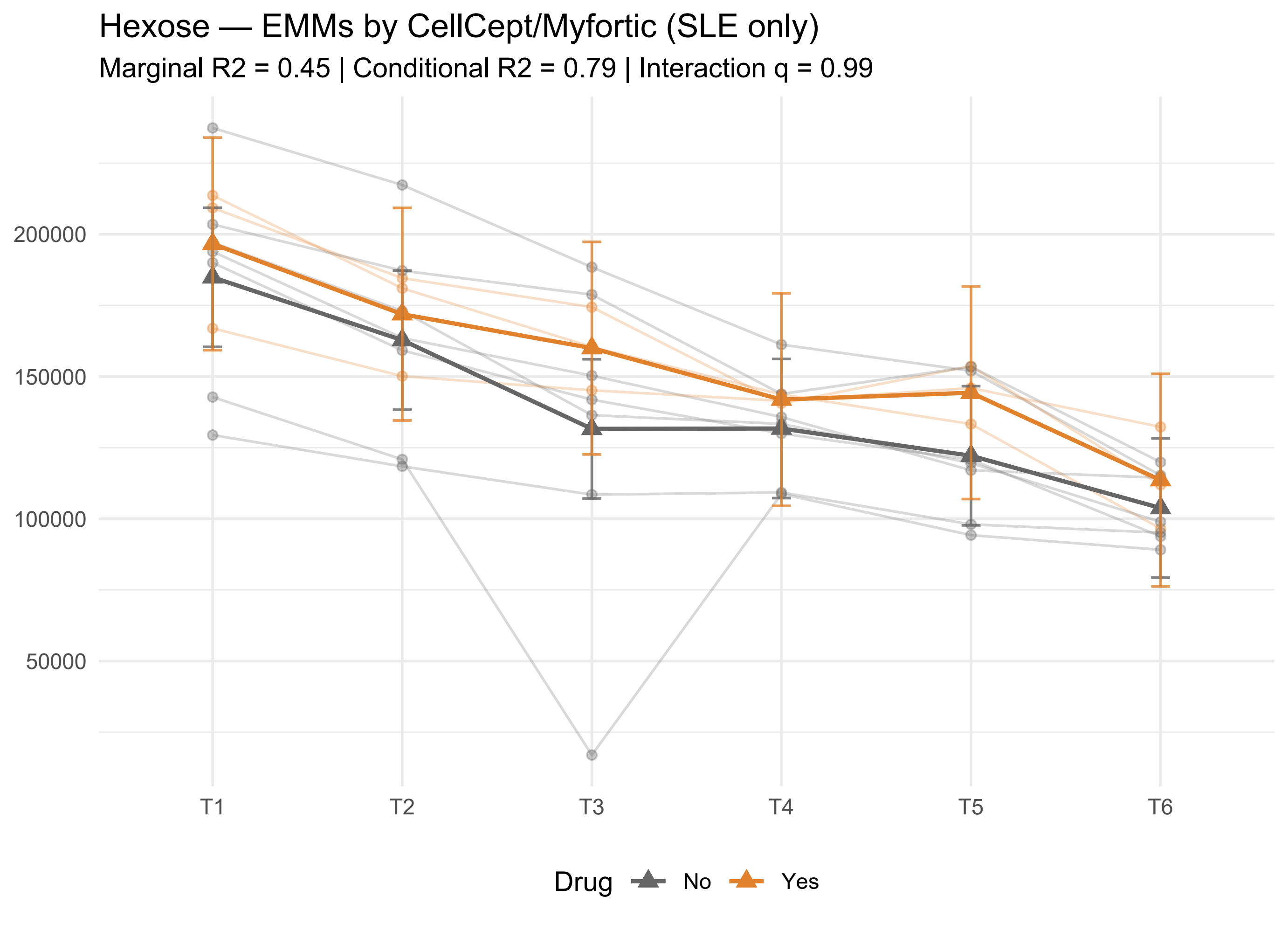
Hexose — EMMs by CellCept/Myfortic (SLE only)
Marginal R2 = 0.45 | Conditional R2 = 0.79 | Interaction q = 0.99
200000
150000
100000
50000
T1
T2
T3
T4
T5
T6
Drug
No
Yes

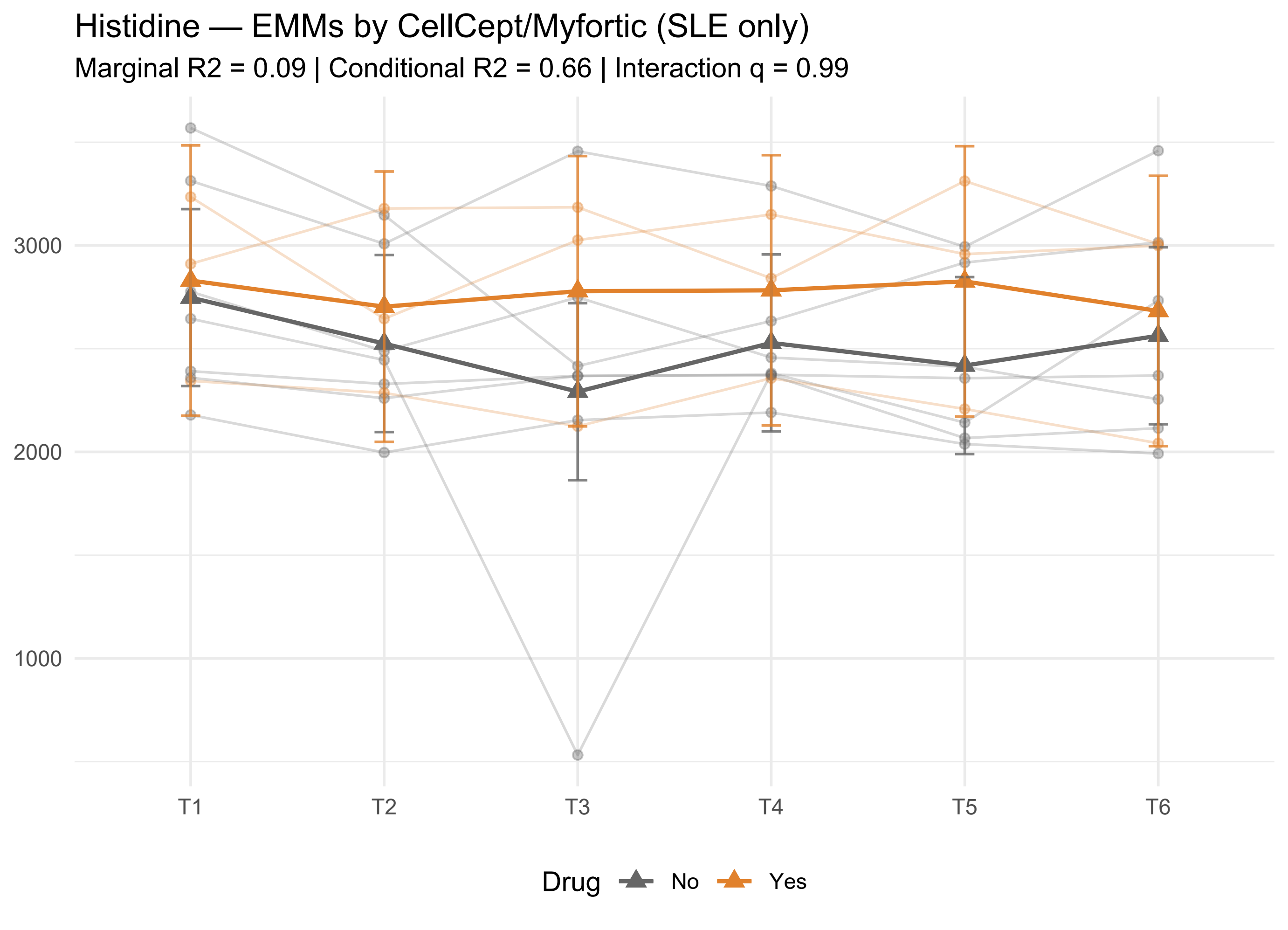

Histidine — EMMs by CellCept/Myfortic (SLE only)
Marginal R2 = 0.09 | Conditional R2 = 0.66 | Interaction q = 0.99
3000
2000
1000
T1
T2
T3
T4
T5
T6
Drug
No
Yes

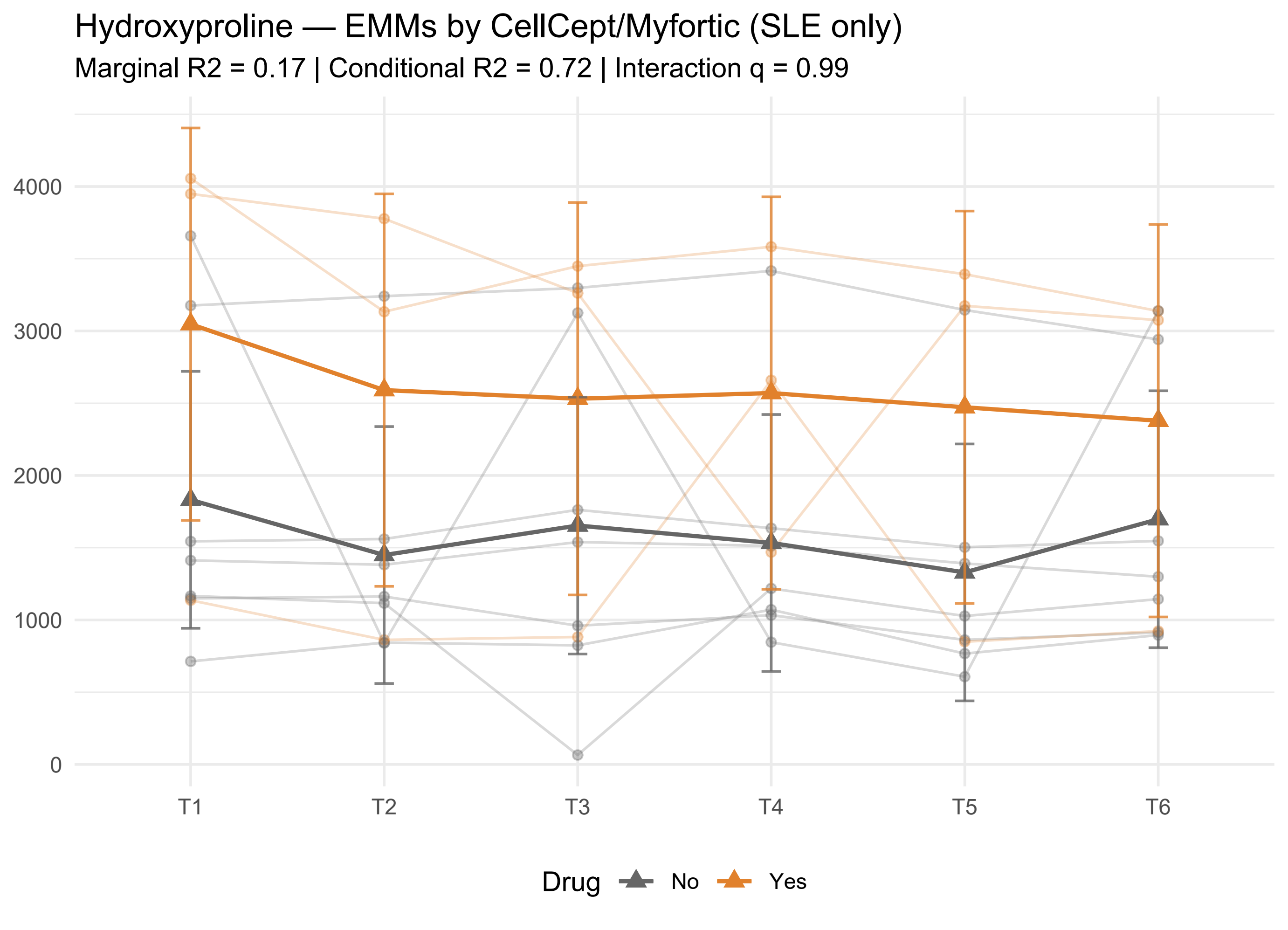
Hydroxyproline — EMMs by CellCept/Myfortic (SLE only)
Marginal R2 = 0.17 | Conditional R2 = 0.72 | Interaction q = 0.99
4000
3000
2000
1000
0
T1
T2
T3
T4
T5
T6
Drug
No
Yes

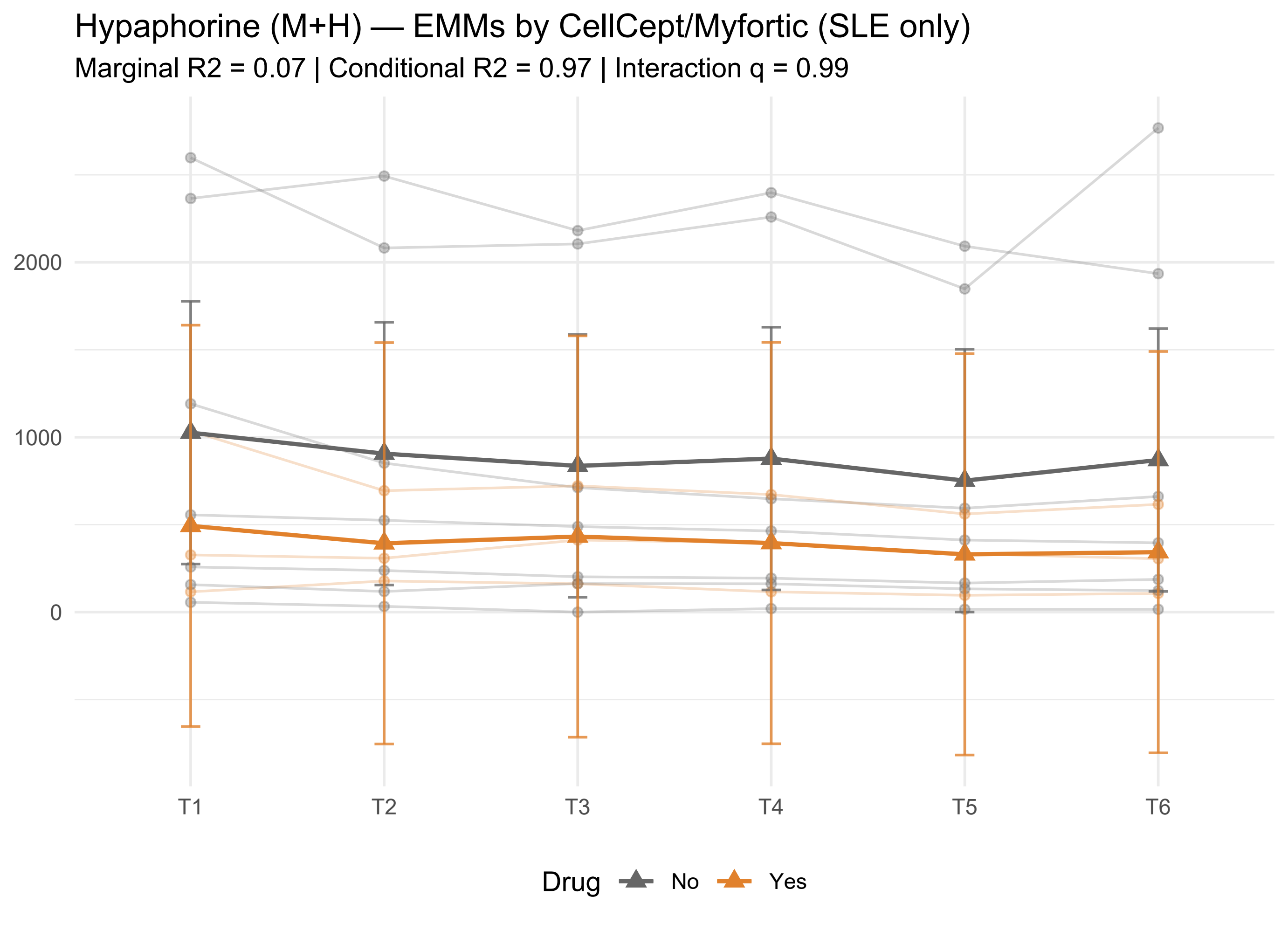

Hypaphorine (M+H) — EMMs by CellCept/Myfortic (SLE only)
Marginal R2 = 0.07 | Conditional R2 = 0.97 | Interaction q = 0.99
2000
1000
0
T1
T2
T3
T4
T5
T6
Drug
No
Yes

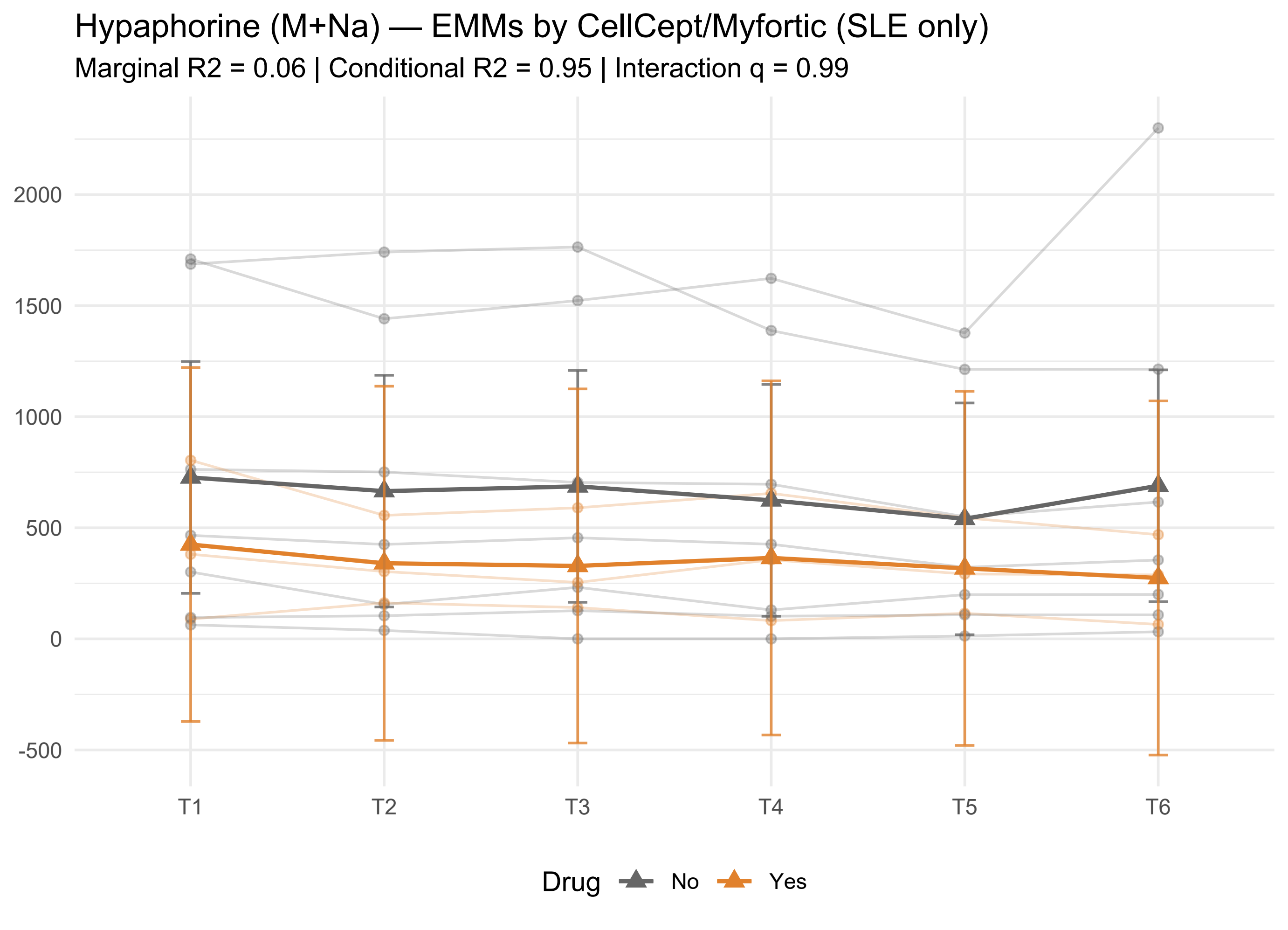

Hypaphorine (M+Na) — EMMs by CellCept/Myfortic (SLE only)
Marginal R2 = 0.06 | Conditional R2 = 0.95 | Interaction q = 0.99
2000
1500
1000
500
0
-500
T1
T2
T3
T4
T5
T6
Drug
No
Yes

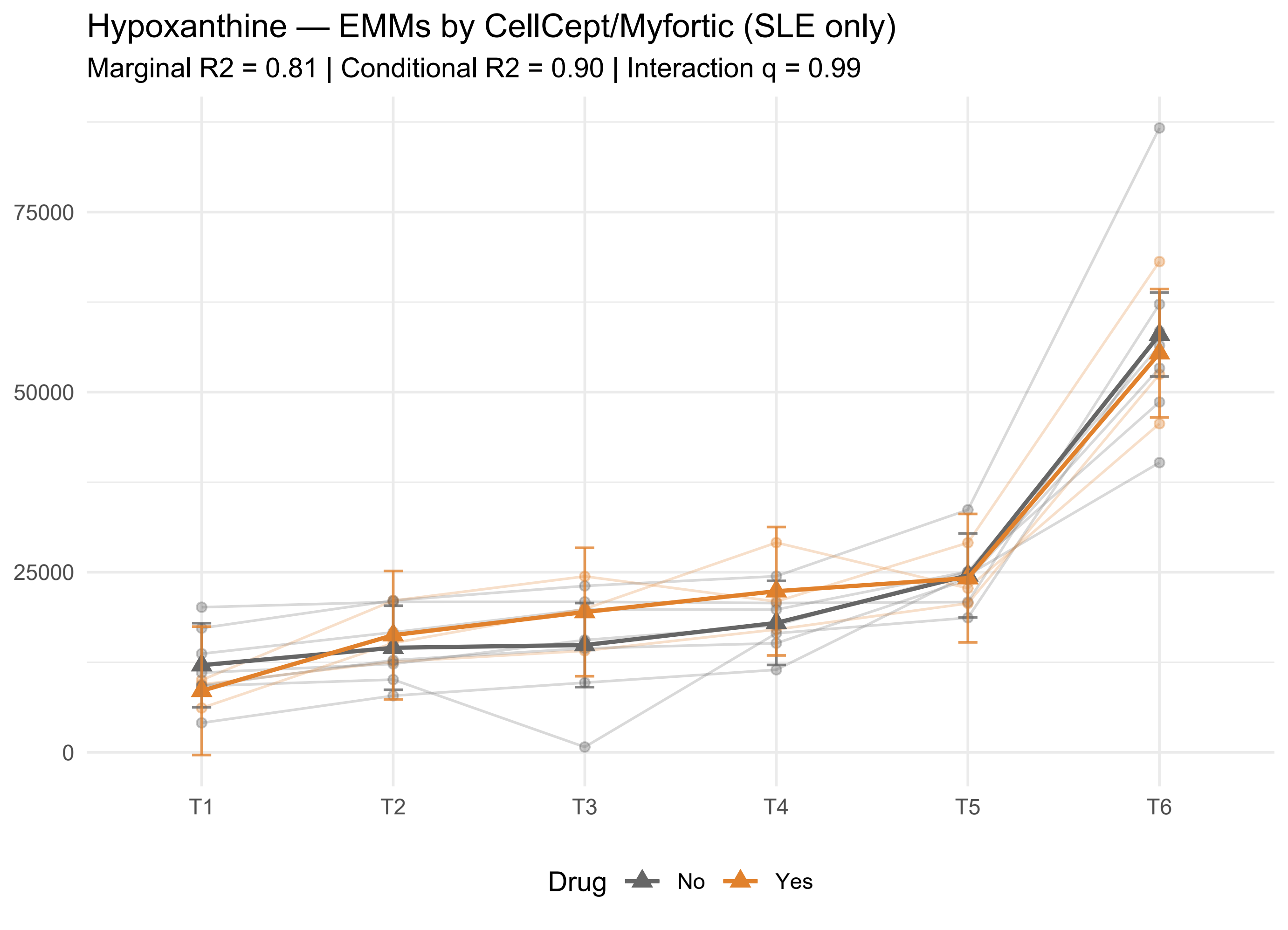

Hypoxanthine — EMMs by CellCept/Myfortic (SLE only)
Marginal R2 = 0.81 | Conditional R2 = 0.90 | Interaction q = 0.99
75000
50000
25000
0
T1
T2
T3
T4
T5
T6
Drug
No
Yes

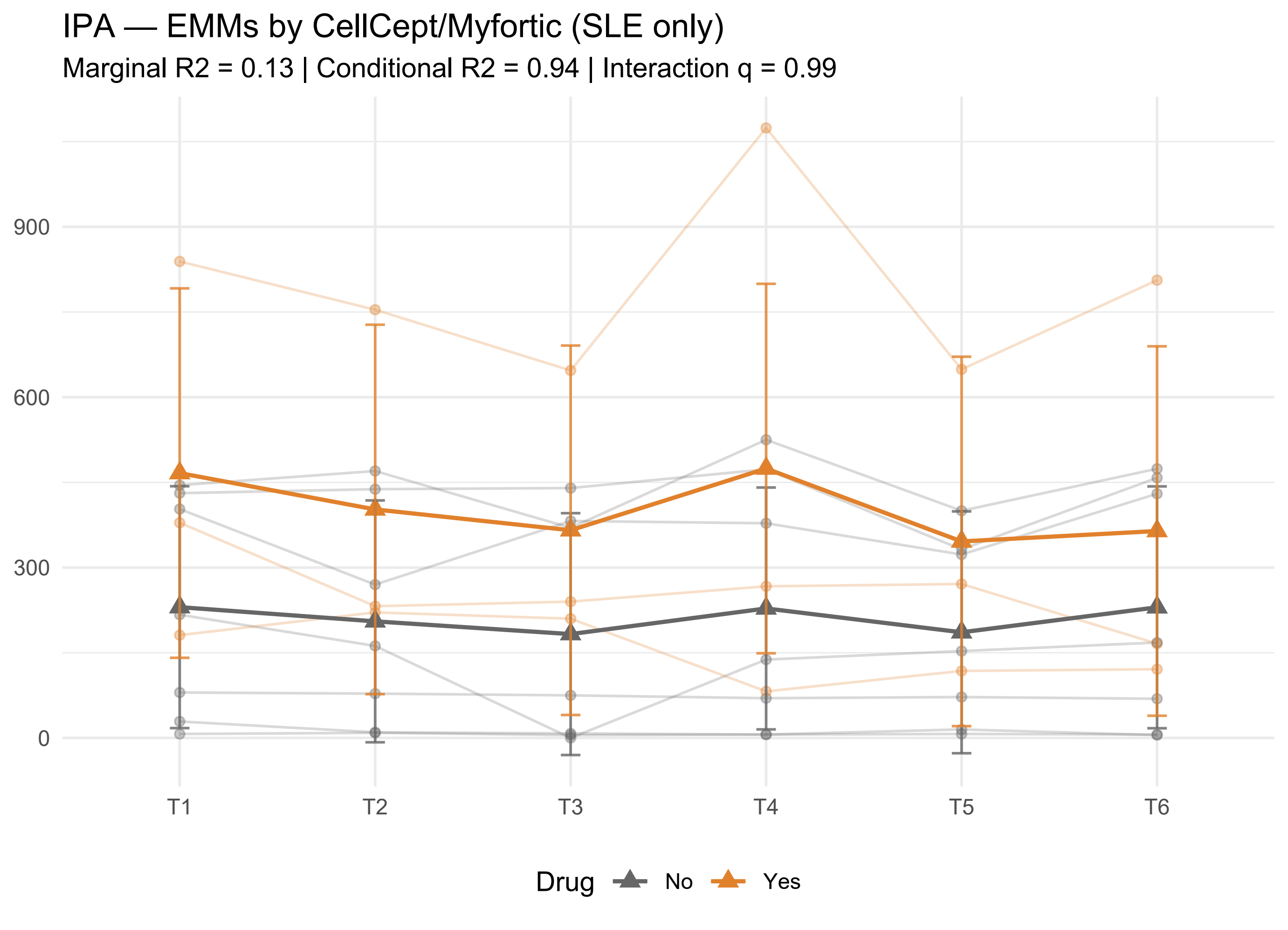

IPA — EMMs by CellCept/Myfortic (SLE only)
Marginal R2 = 0.13 | Conditional R2 = 0.94 | Interaction q = 0.99
900
600
300
0
T1
T2
T3
T4
T5
T6
Drug
No
Yes

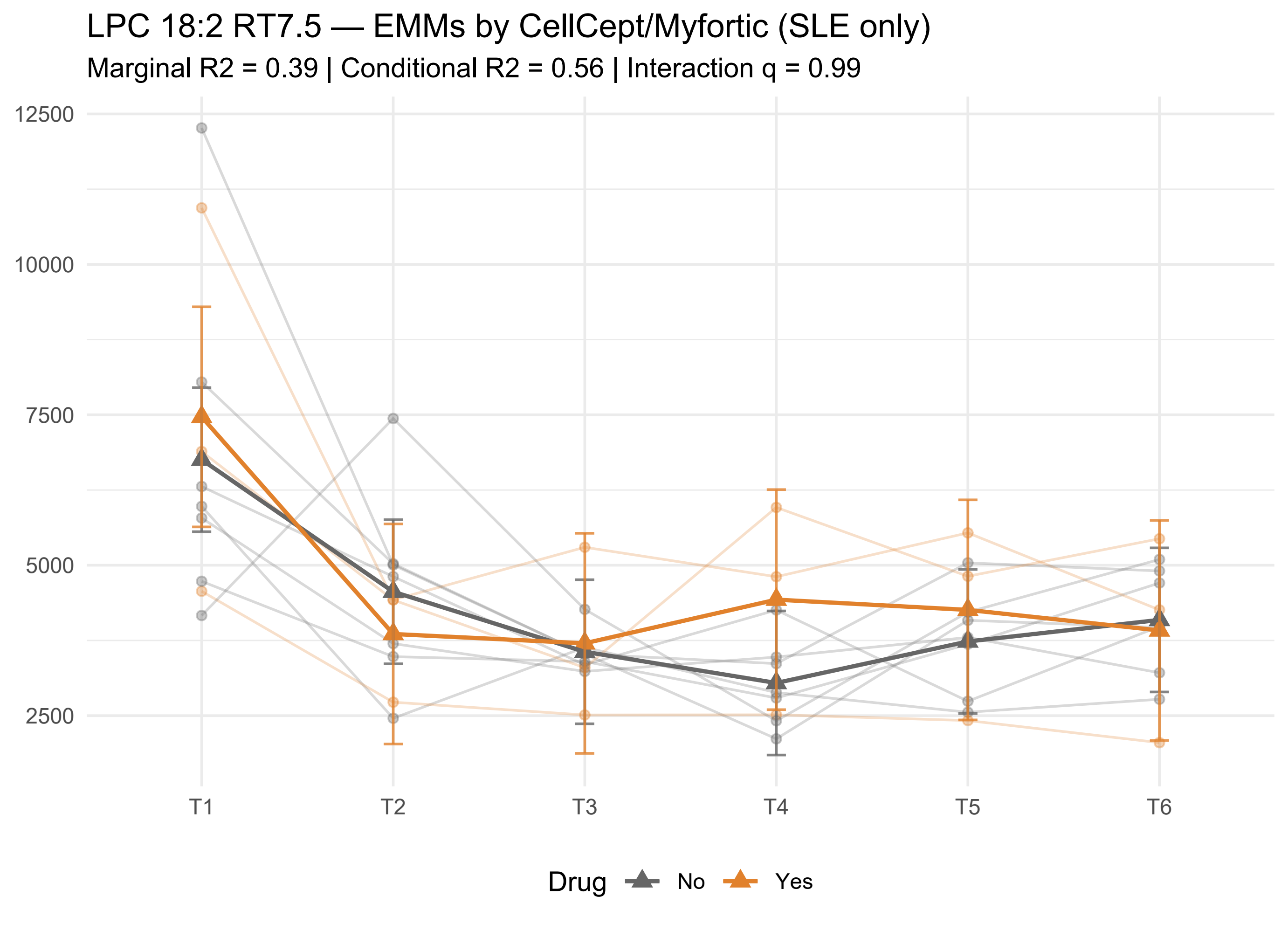

LPC 18:2 RT7.5 — EMMs by CellCept/Myfortic (SLE only)
Marginal R2 = 0.39 | Conditional R2 = 0.56 | Interaction q = 0.99
12500
10000
7500
5000
2500
T1
T2
T3
T4
T5
T6
Drug
No
Yes

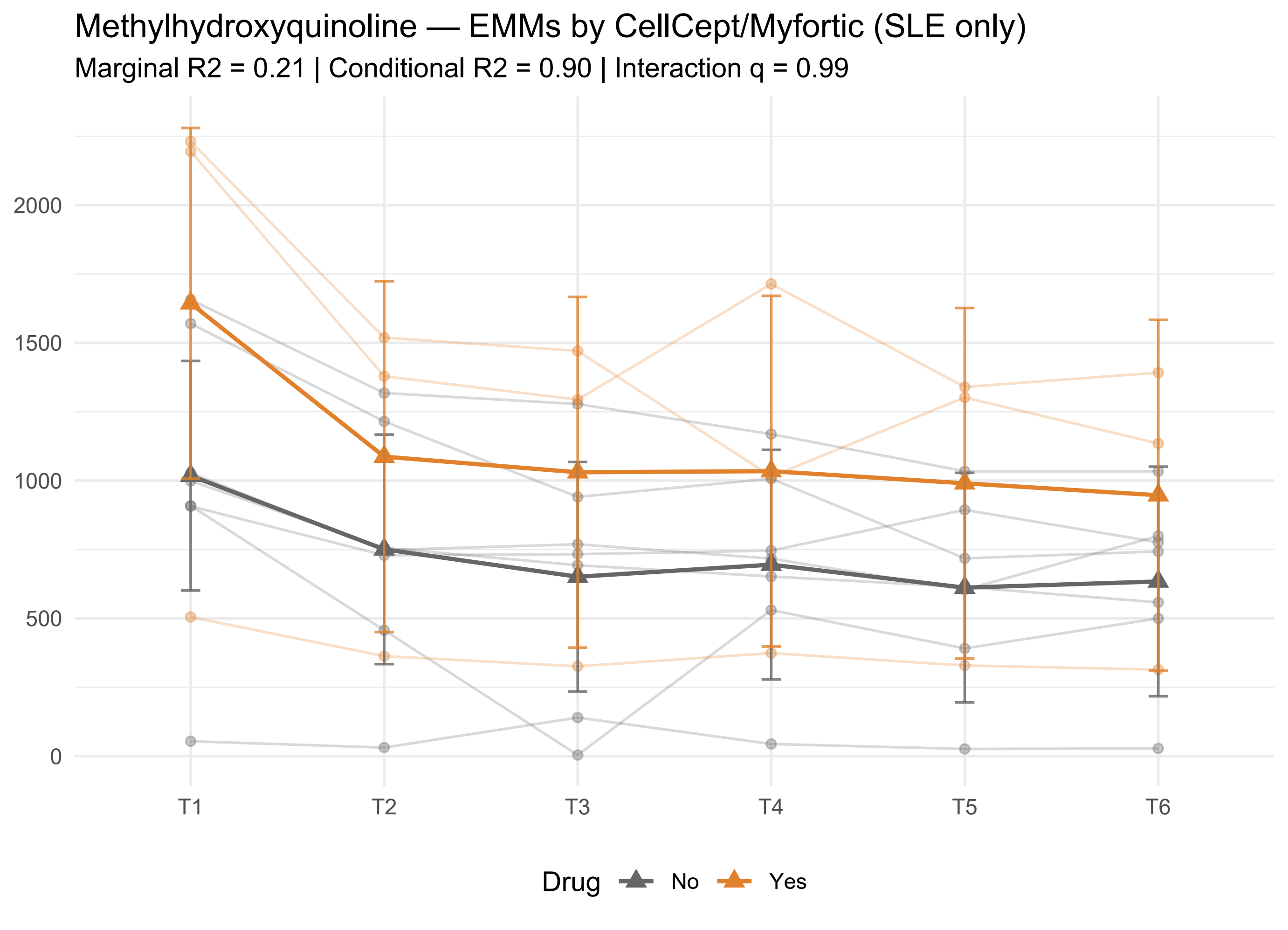
Methylhydroxyquinoline — EMMs by CellCept/Myfortic (SLE only)
Marginal R2 = 0.21 | Conditional R2 = 0.90 | Interaction q = 0.99
2000
1500
1000
500
0
T1
T2
T3
T4
T5
T6
Drug
No
Yes

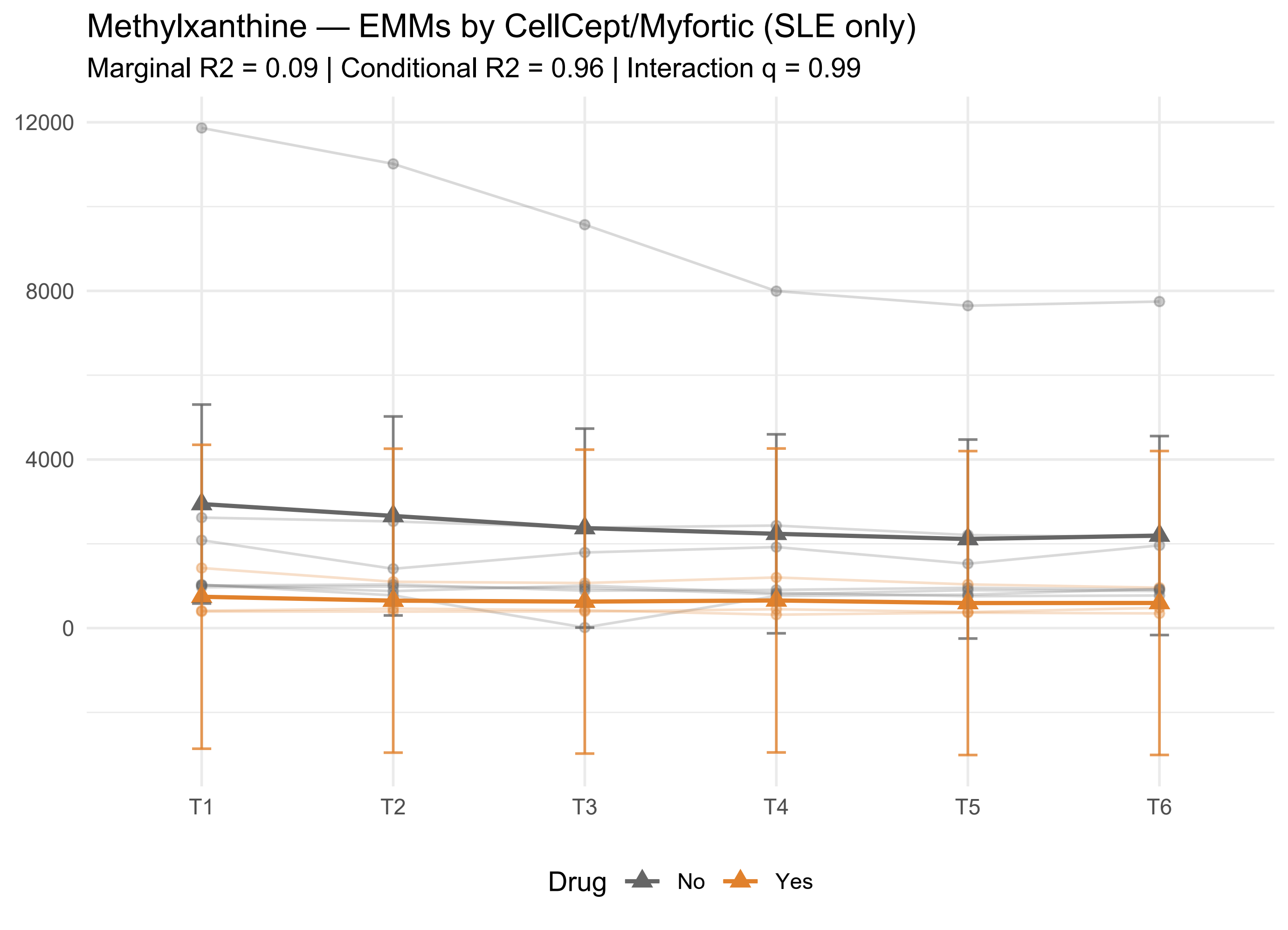
Methylxanthine — EMMs by CellCept/Myfortic (SLE only)
Marginal R2 = 0.09 | Conditional R2 = 0.96 | Interaction q = 0.99
12000
8000
4000
0
T1
T2
T3
T4
T5
T6
Drug
No
Yes

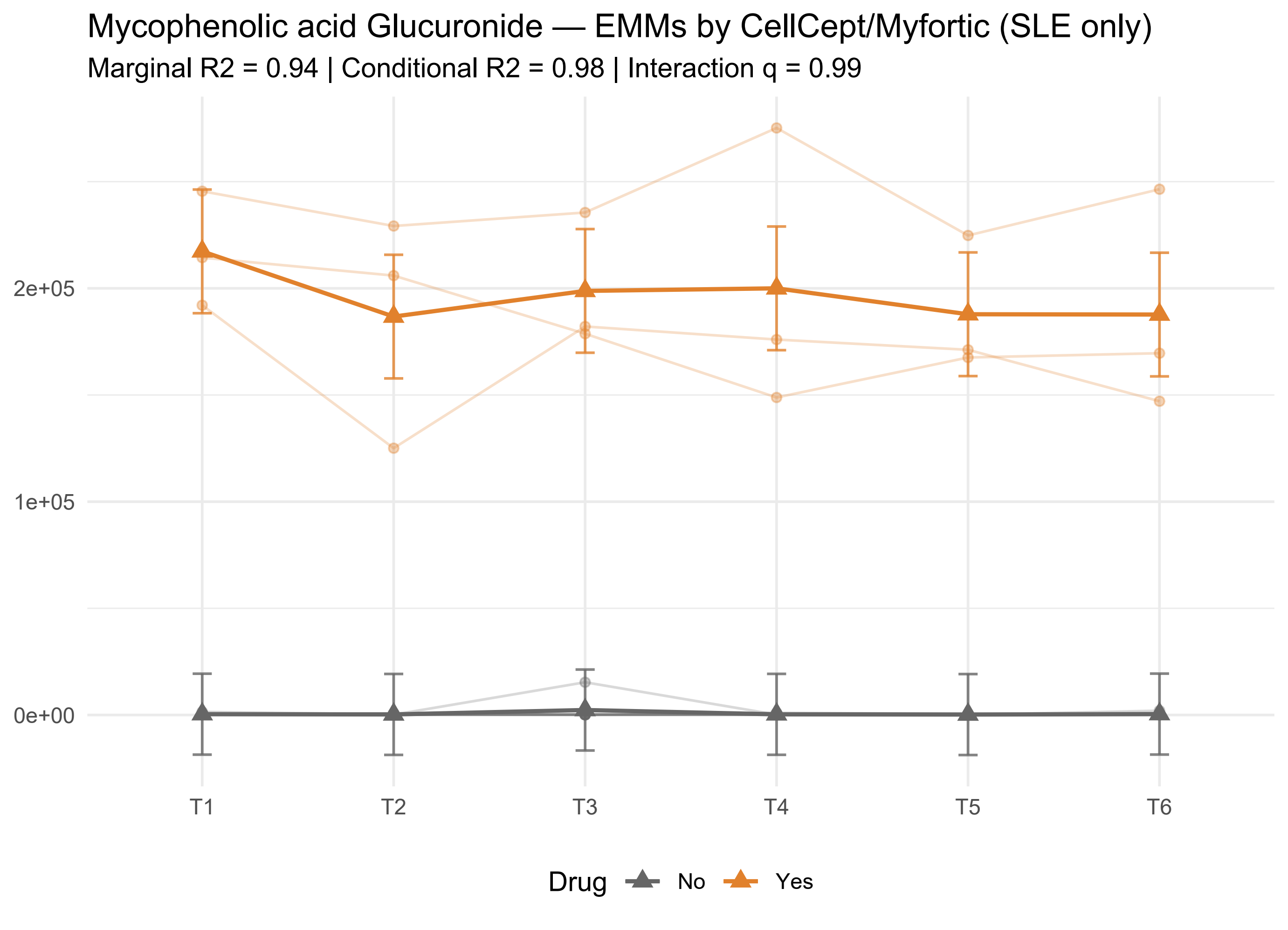

Mycophenolic acid Glucuronide — EMMs by CellCept/Myfortic (SLE only)
Marginal R2 = 0.94 | Conditional R2 = 0.98 | Interaction q = 0.99
2e+05
1e+05
0e+00
T1
T2
T3
T4
T5
T6
Drug
No
Yes

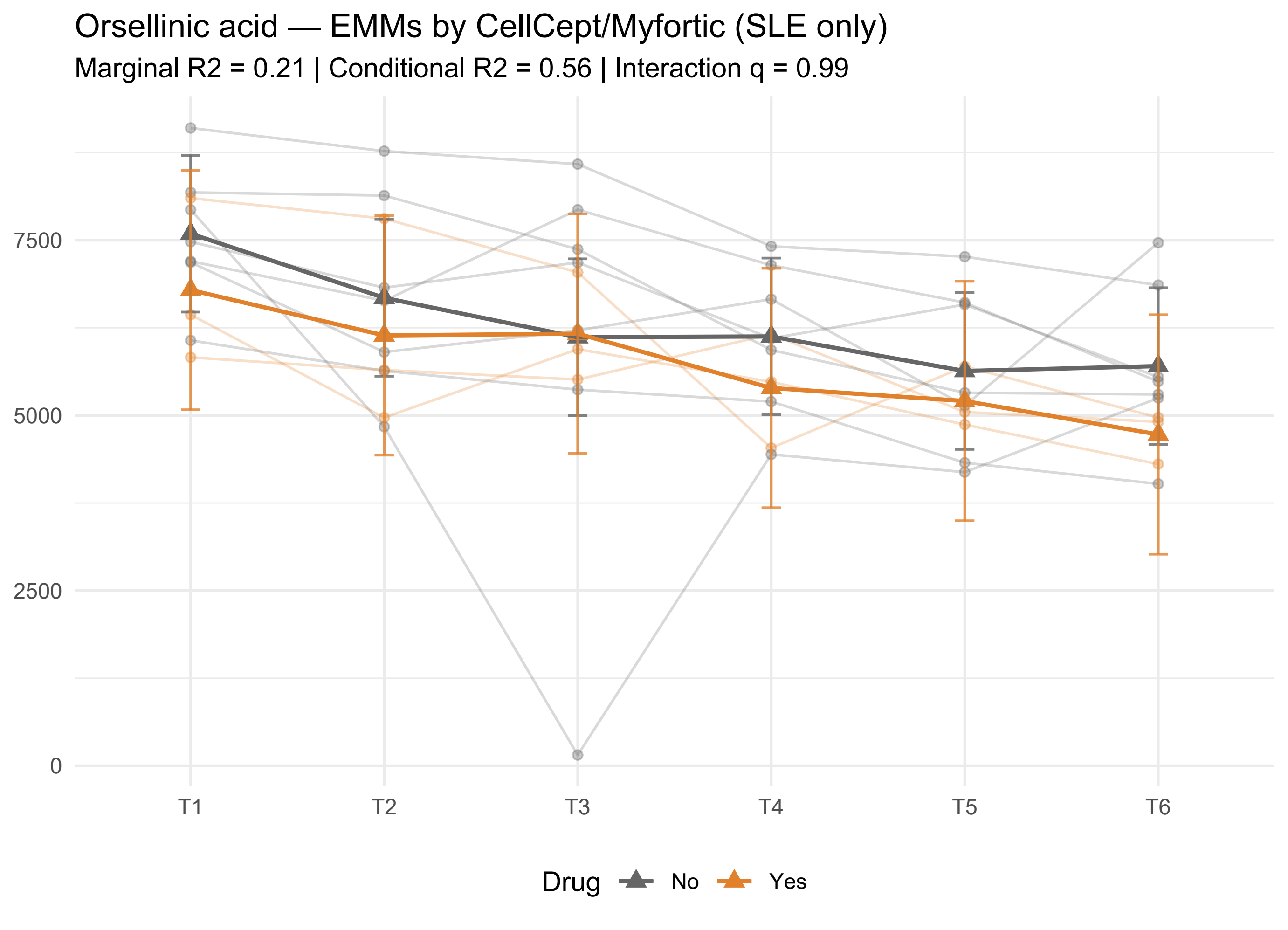
Orsellinic acid — EMMs by CellCept/Myfortic (SLE only)
Marginal R2 = 0.21 | Conditional R2 = 0.56 | Interaction q = 0.99
7500
5000
2500
0
T1
T2
T3
T4
T5
T6
Drug
No
Yes

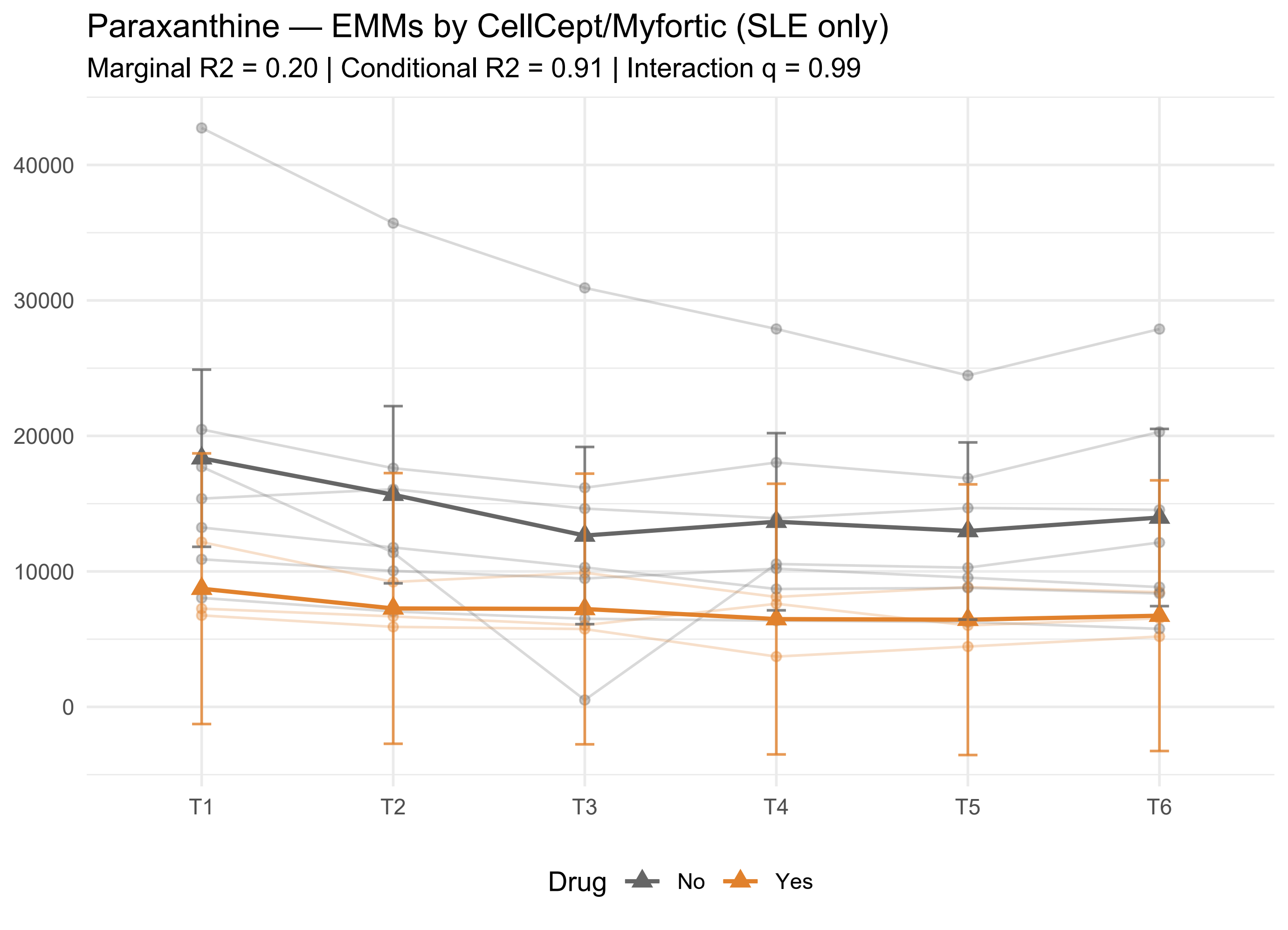
Paraxanthine — EMMs by CellCept/Myfortic (SLE only)
Marginal R2 = 0.20 | Conditional R2 = 0.91 | Interaction q = 0.99
40000
30000
20000
10000
0
T1
T2
T3
T4
T5
T6
Drug
No
Yes

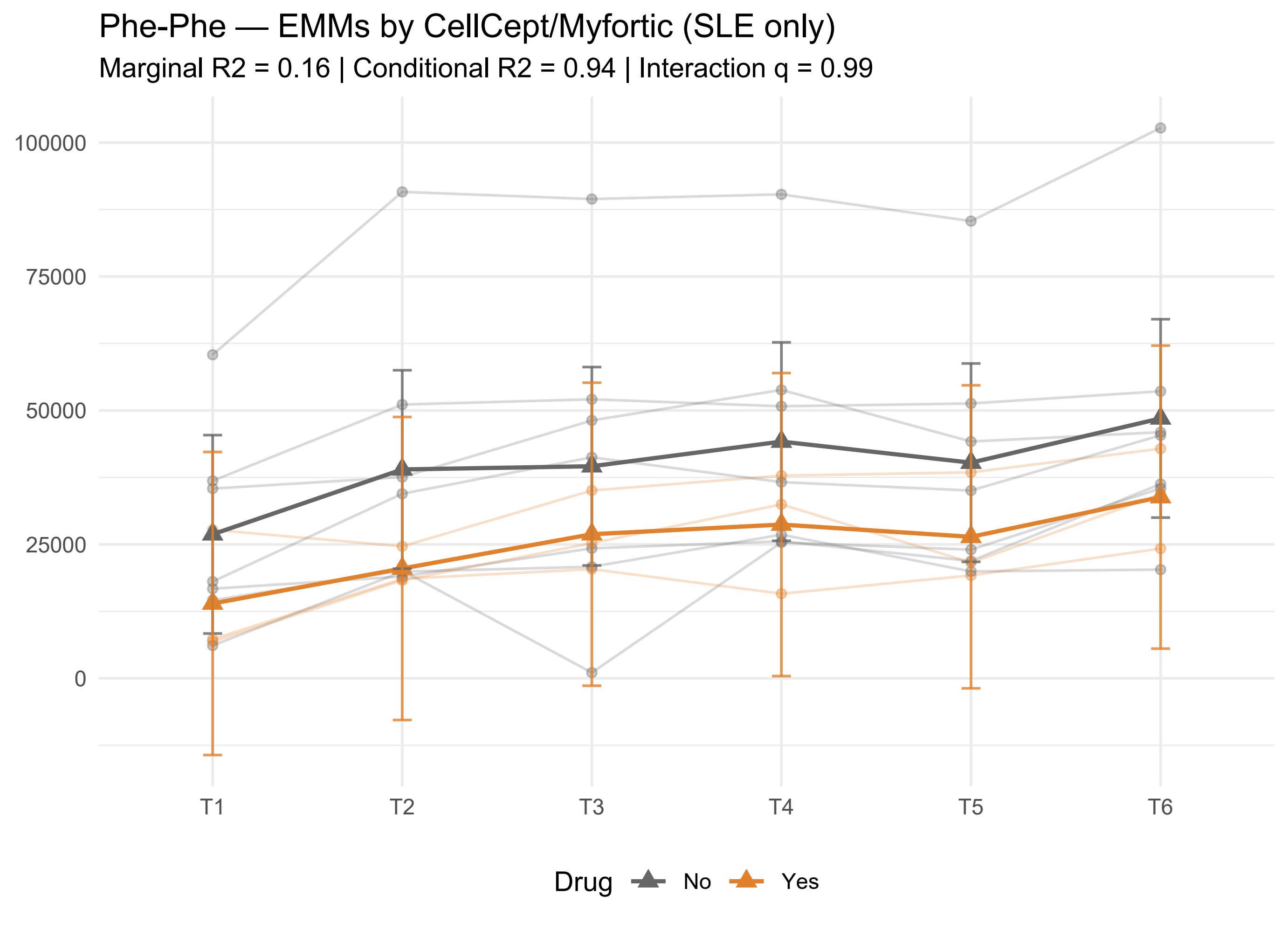

Phe-Phe — EMMs by CellCept/Myfortic (SLE only)
Marginal R2 = 0.16 | Conditional R2 = 0.94 | Interaction q = 0.99
100000
75000
50000
25000
0
T1
T2
T3
T4
T5
T6
Drug
No
Yes

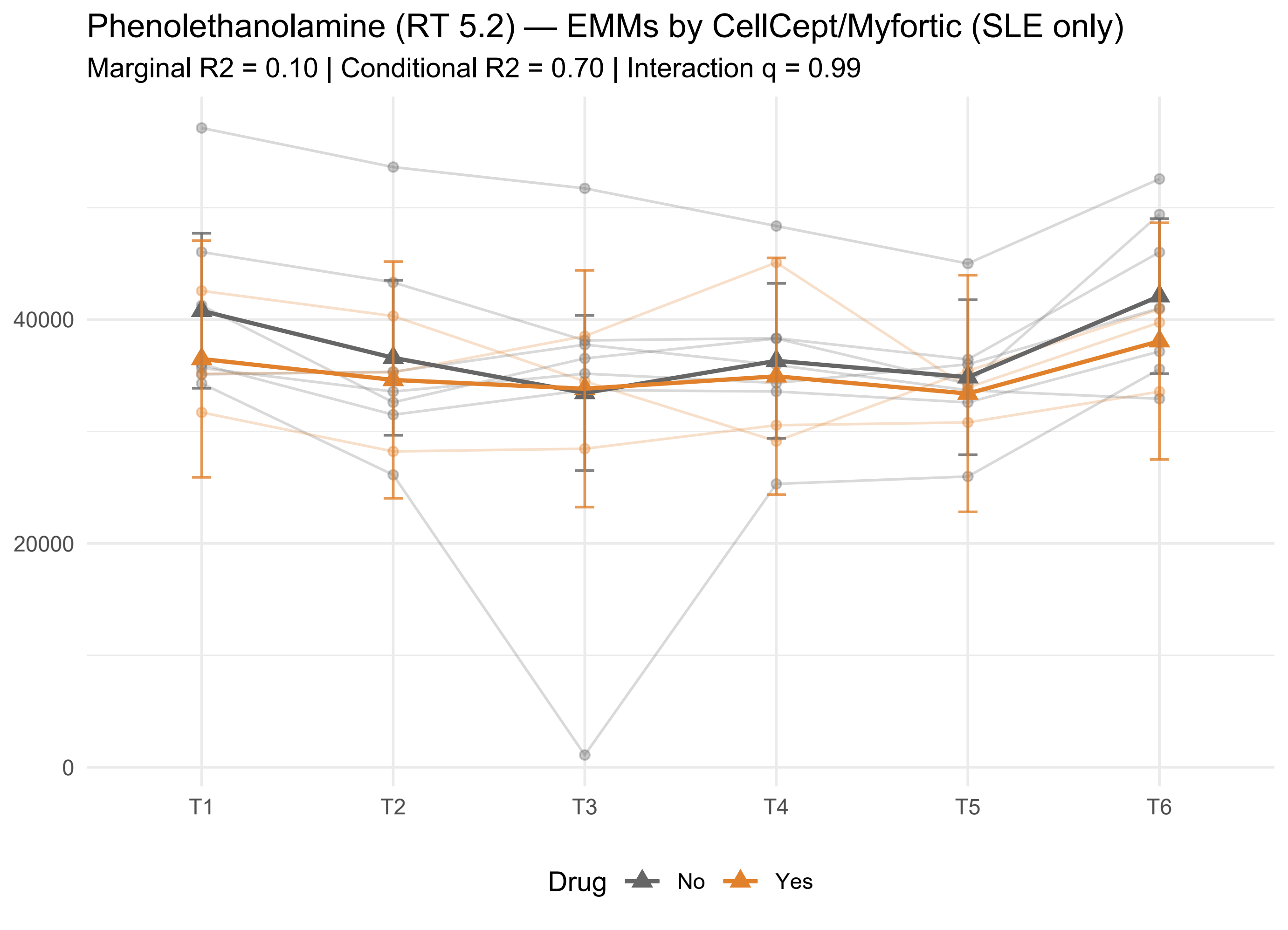

Phenolethanolamine (RT 5.2) — EMMs by CellCept/Myfortic (SLE only)
Marginal R2 = 0.10 | Conditional R2 = 0.70 | Interaction q = 0.99
0
20000
40000
T1
T2
T3
T4
T5
T6
Drug
No
Yes

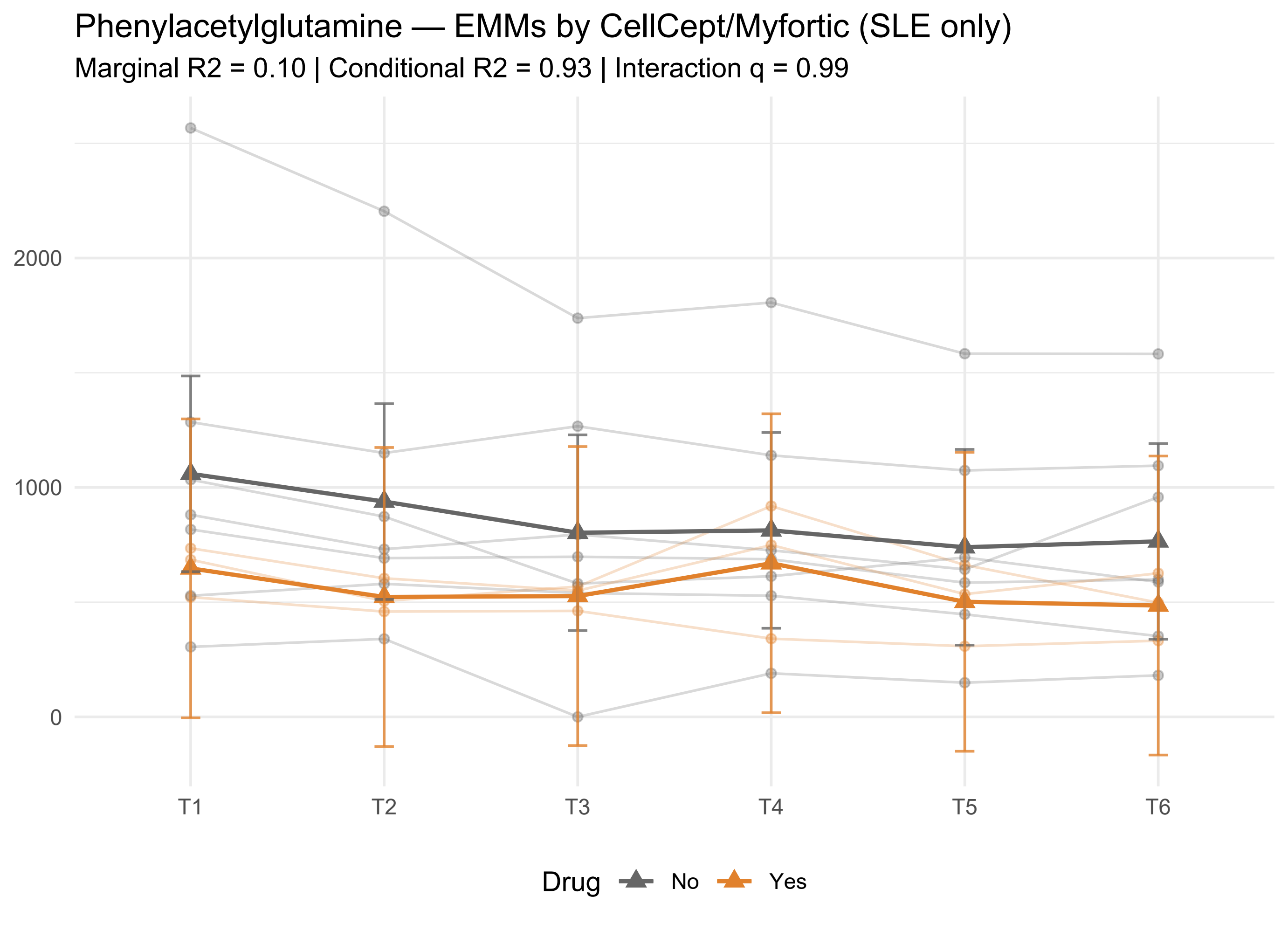

Phenylacetylglutamine — EMMs by CellCept/Myfortic (SLE only)
Marginal R2 = 0.10 | Conditional R2 = 0.93 | Interaction q = 0.99
2000
1000
0
T1
T2
T3
T4
T5
T6
Drug
No
Yes

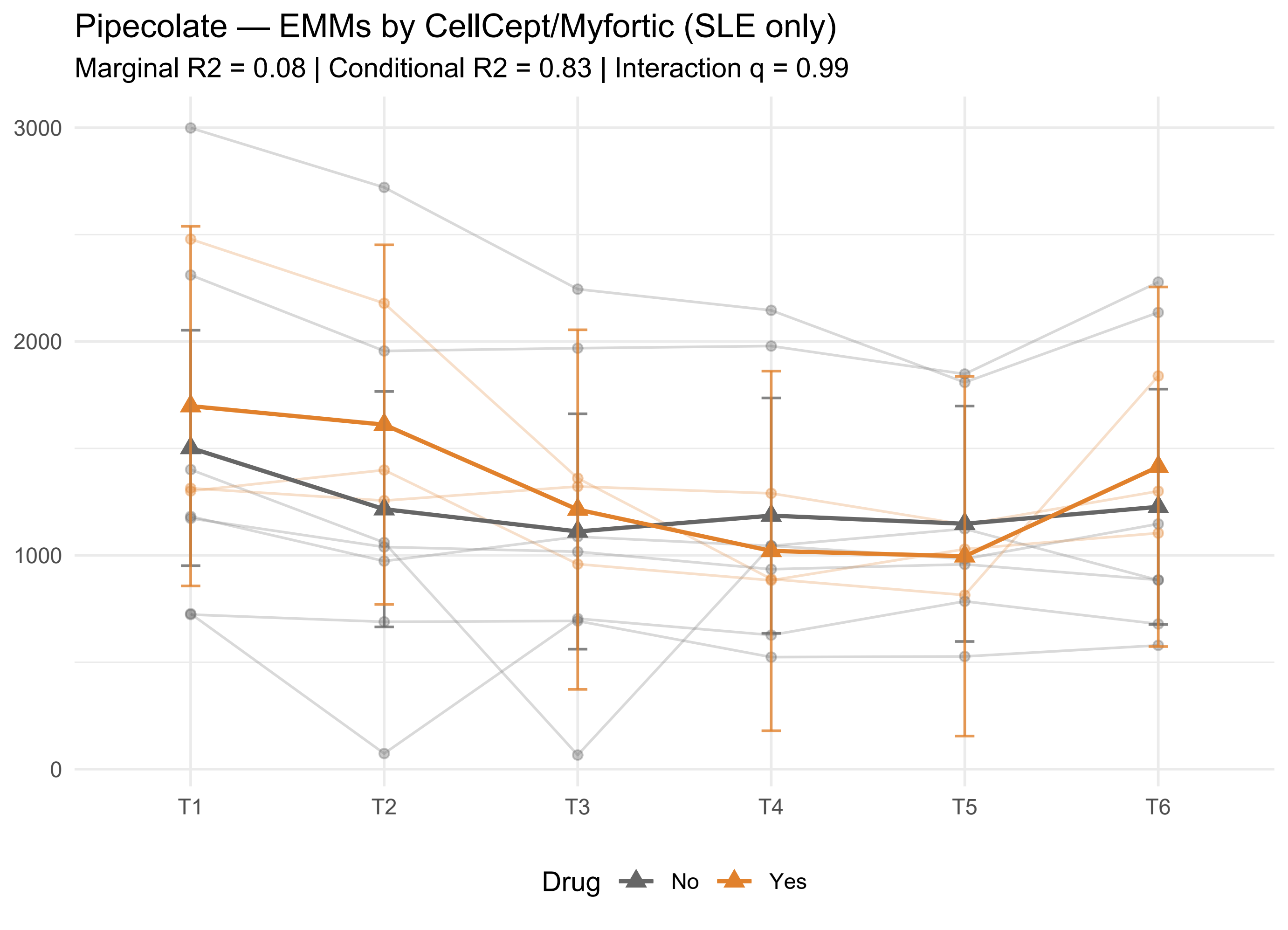

Pipecolate — EMMs by CellCept/Myfortic (SLE only)
Marginal R2 = 0.08 | Conditional R2 = 0.83 | Interaction q = 0.99
3000
2000
1000
0
T1
T2
T3
T4
T5
T6
Drug
No
Yes

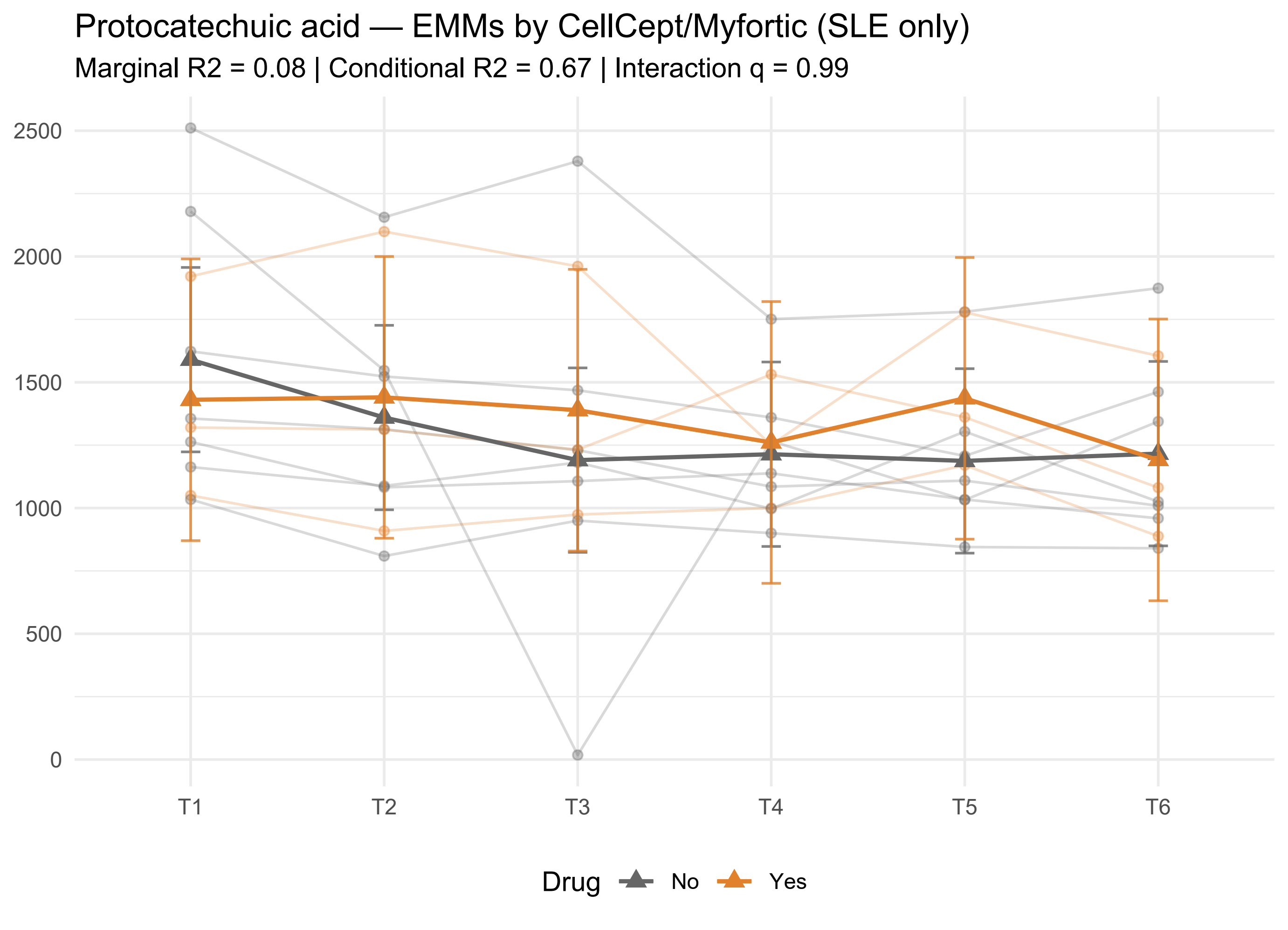
Protocatechuic acid — EMMs by CellCept/Myfortic (SLE only)
Marginal R2 = 0.08 | Conditional R2 = 0.67 | Interaction q = 0.99
2500
2000
1500
1000
500
0
T1
T2
T3
T4
T5
T6
Drug
No
Yes

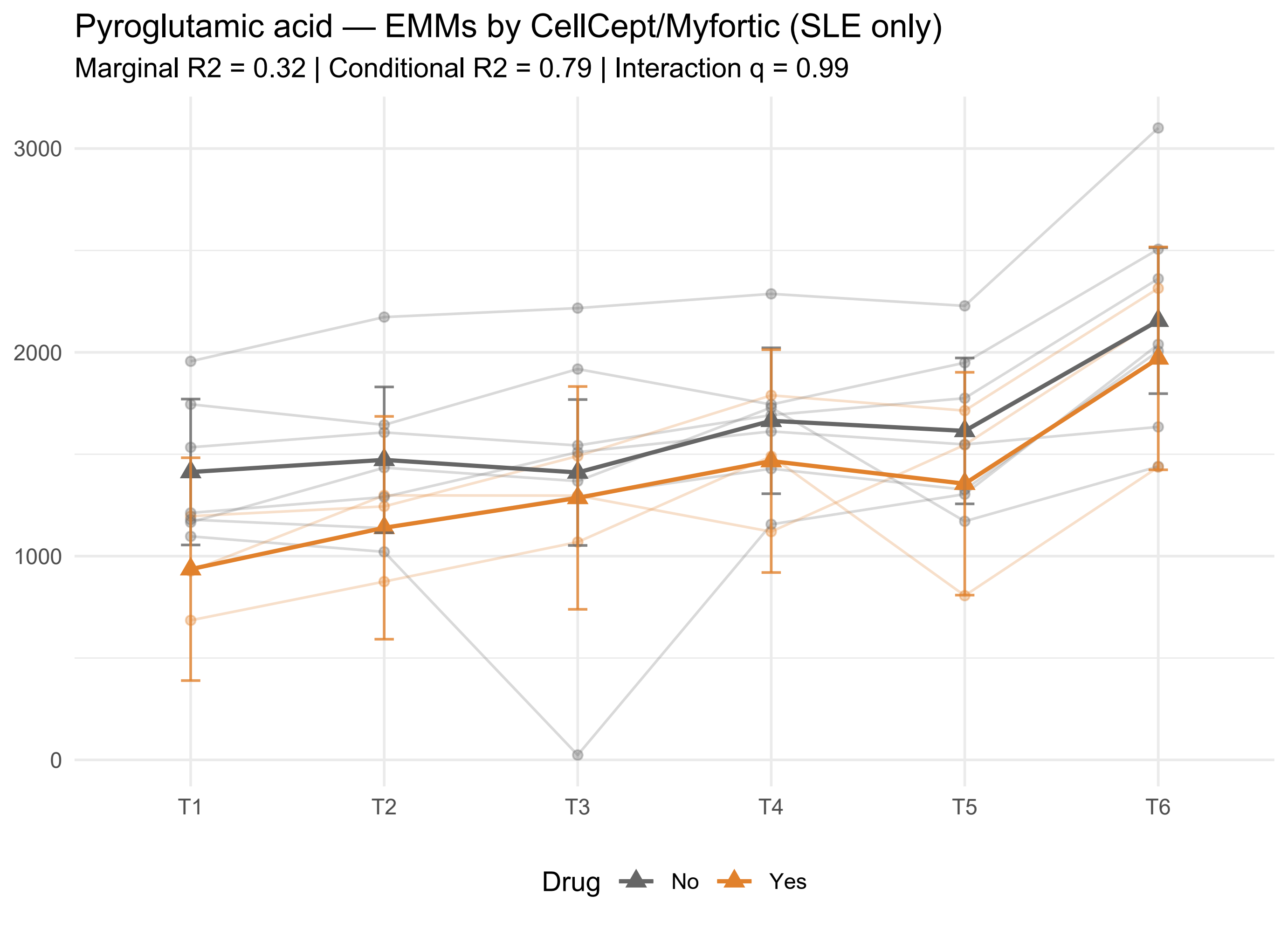
Pyroglutamic acid — EMMs by CellCept/Myfortic (SLE only)
Marginal R2 = 0.32 | Conditional R2 = 0.79 | Interaction q = 0.99
3000
2000
1000
0
T1
T2
T3
T4
T5
T6
Drug
No
Yes

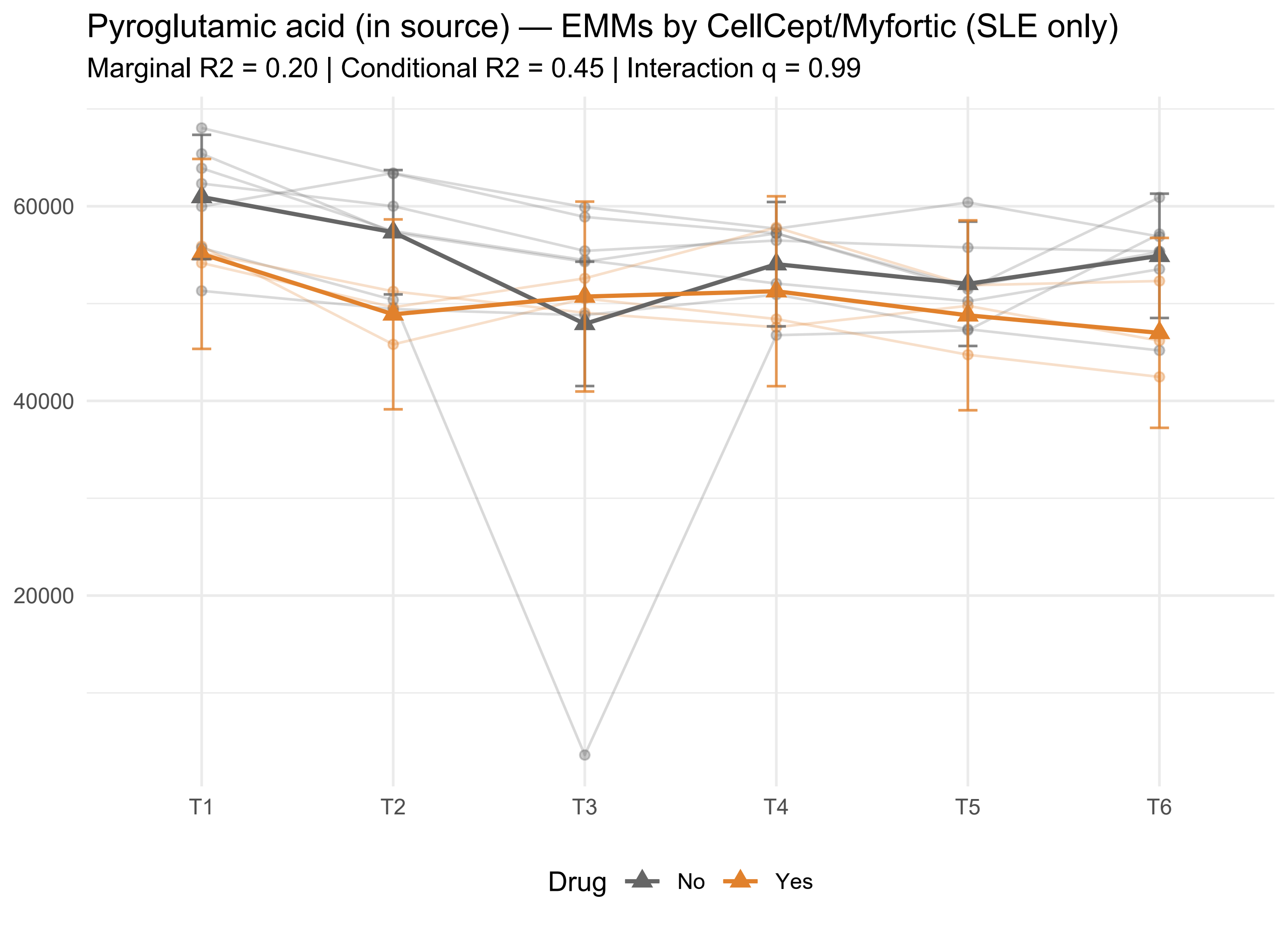
Pyroglutamic acid (in source) — EMMs by CellCept/Myfortic (SLE only)
Marginal R2 = 0.20 | Conditional R2 = 0.45 | Interaction q = 0.99
60000
40000
20000
T1
T2
T3
T4
T5
T6
Drug
No
Yes

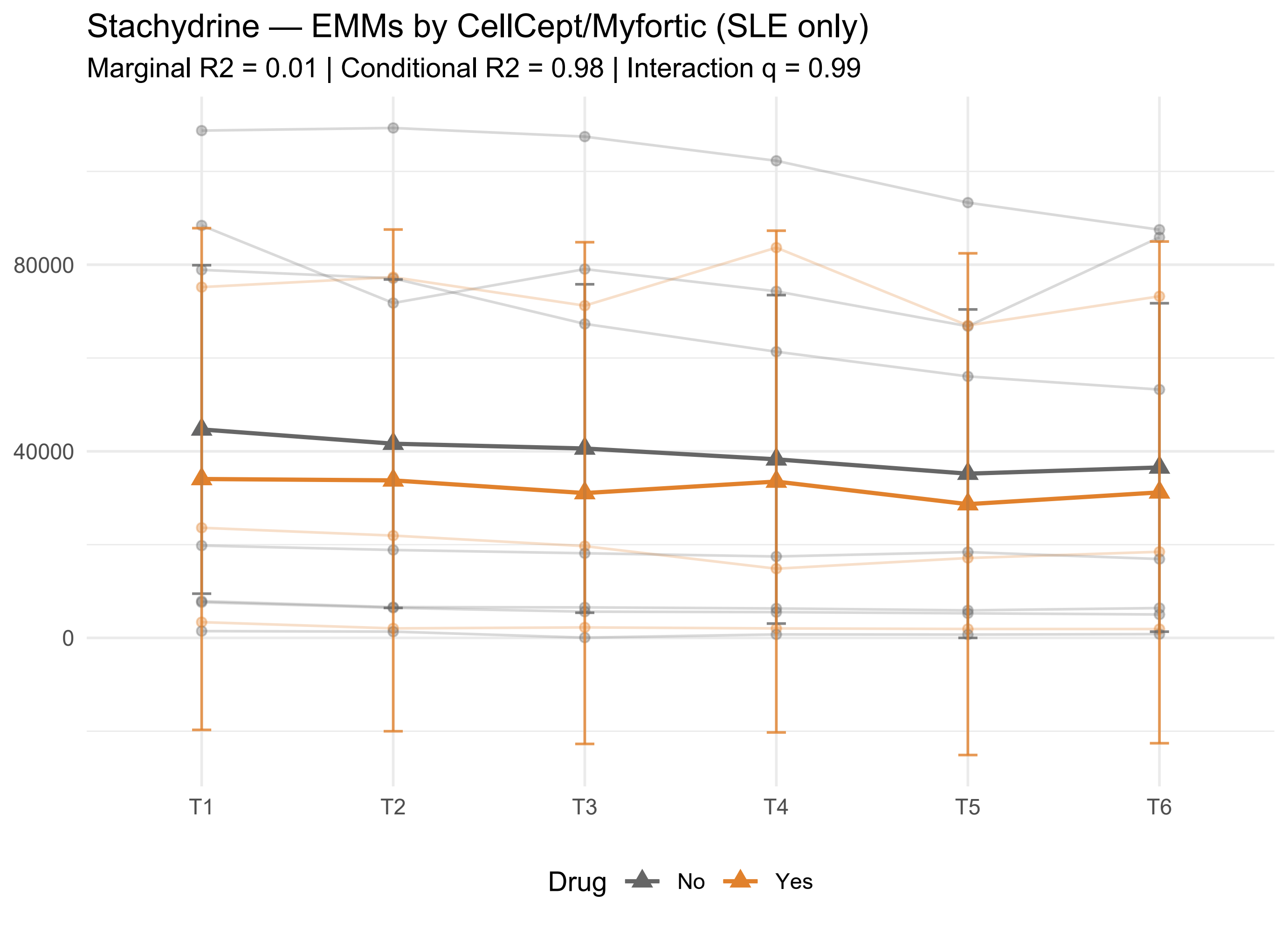
Stachydrine — EMMs by CellCept/Myfortic (SLE only)
Marginal R2 = 0.01 | Conditional R2 = 0.98 | Interaction q = 0.99
80000
40000
0
T1
T2
T3
T4
T5
T6
Drug
No
Yes

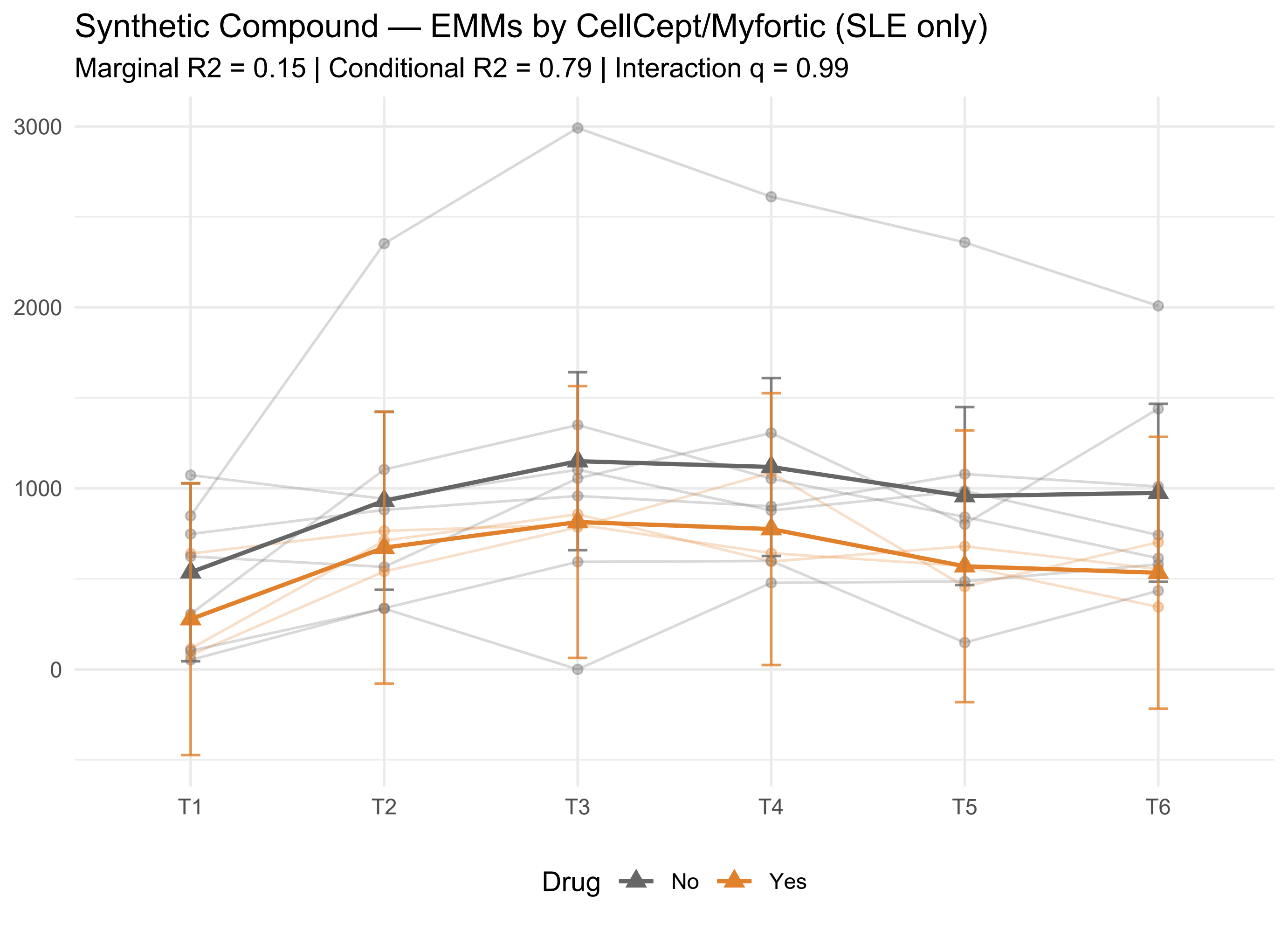

Synthetic Compound — EMMs by CellCept/Myfortic (SLE only)
Marginal R2 = 0.15 | Conditional R2 = 0.79 | Interaction q = 0.99
3000
2000
1000
0
T1
T2
T3
T4
T5
T6
Drug
No
Yes

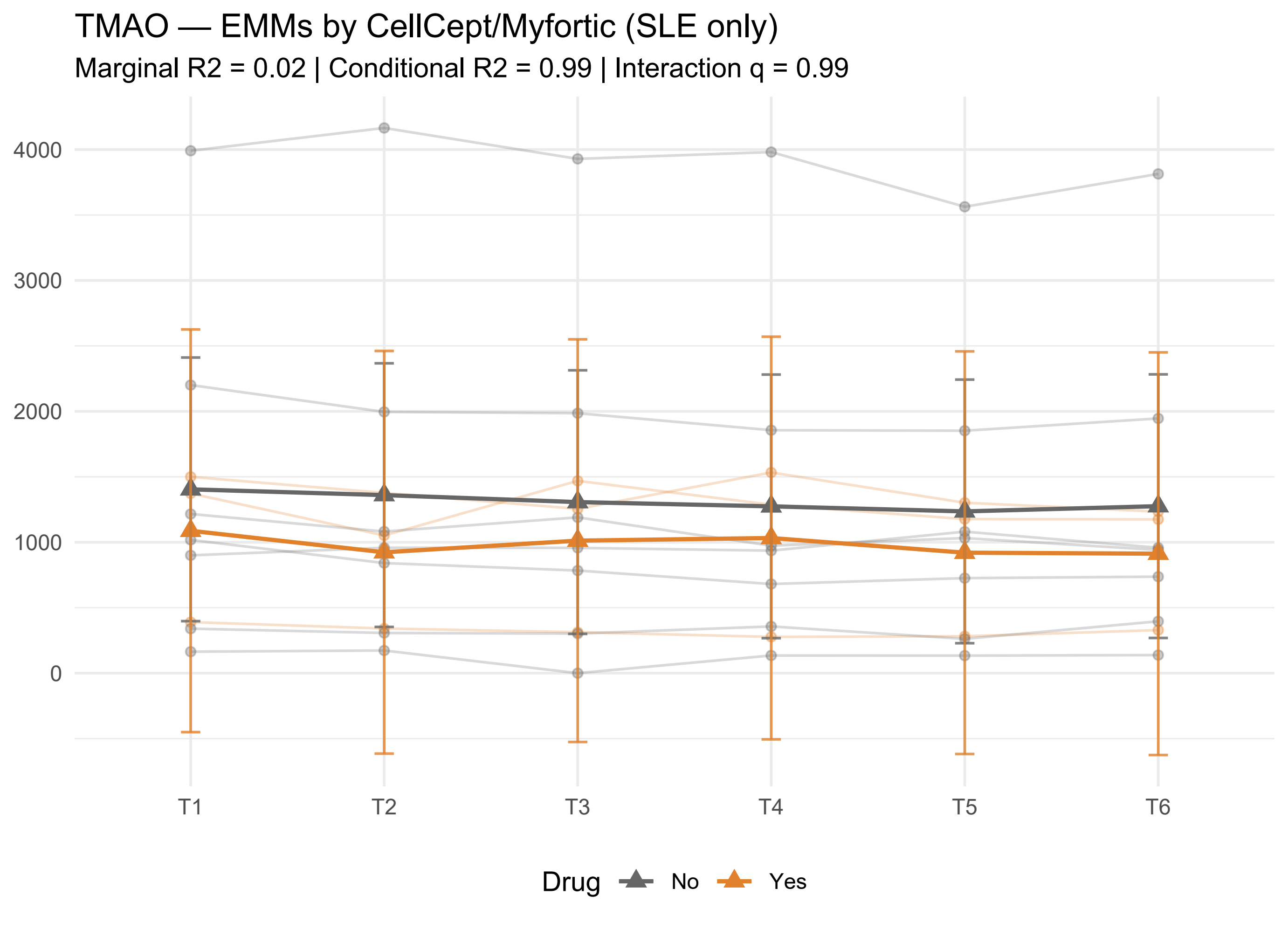
TMAO — EMMs by CellCept/Myfortic (SLE only)
Marginal R2 = 0.02 | Conditional R2 = 0.99 | Interaction q = 0.99
4000
3000
2000
1000
0
T1
T2
T3
T4
T5
T6
Drug
No
Yes

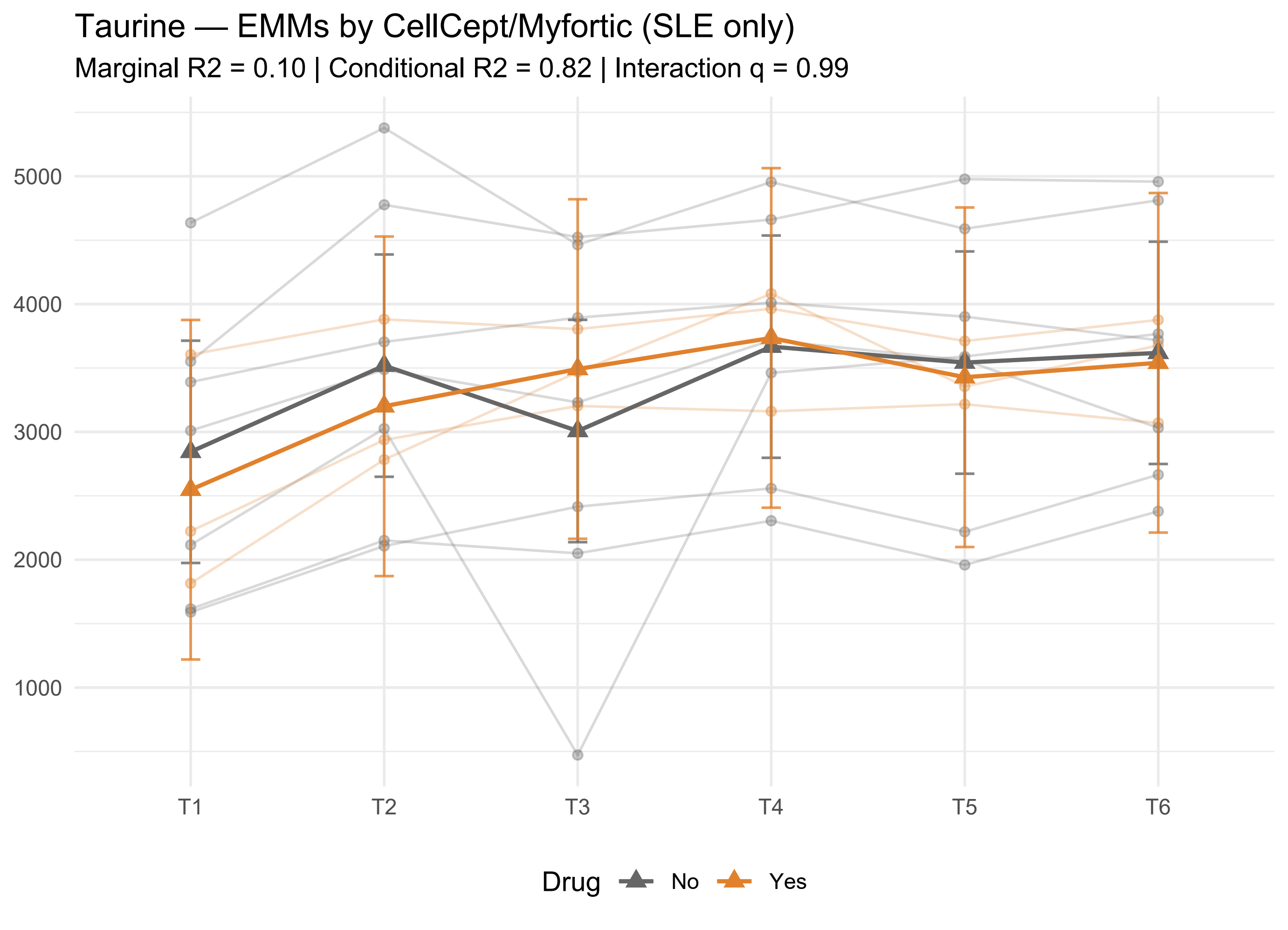

Taurine — EMMs by CellCept/Myfortic (SLE only)
Marginal R2 = 0.10 | Conditional R2 = 0.82 | Interaction q = 0.99
5000
4000
3000
2000
1000
T1
T2
T3
T4
T5
T6
Drug
No
Yes

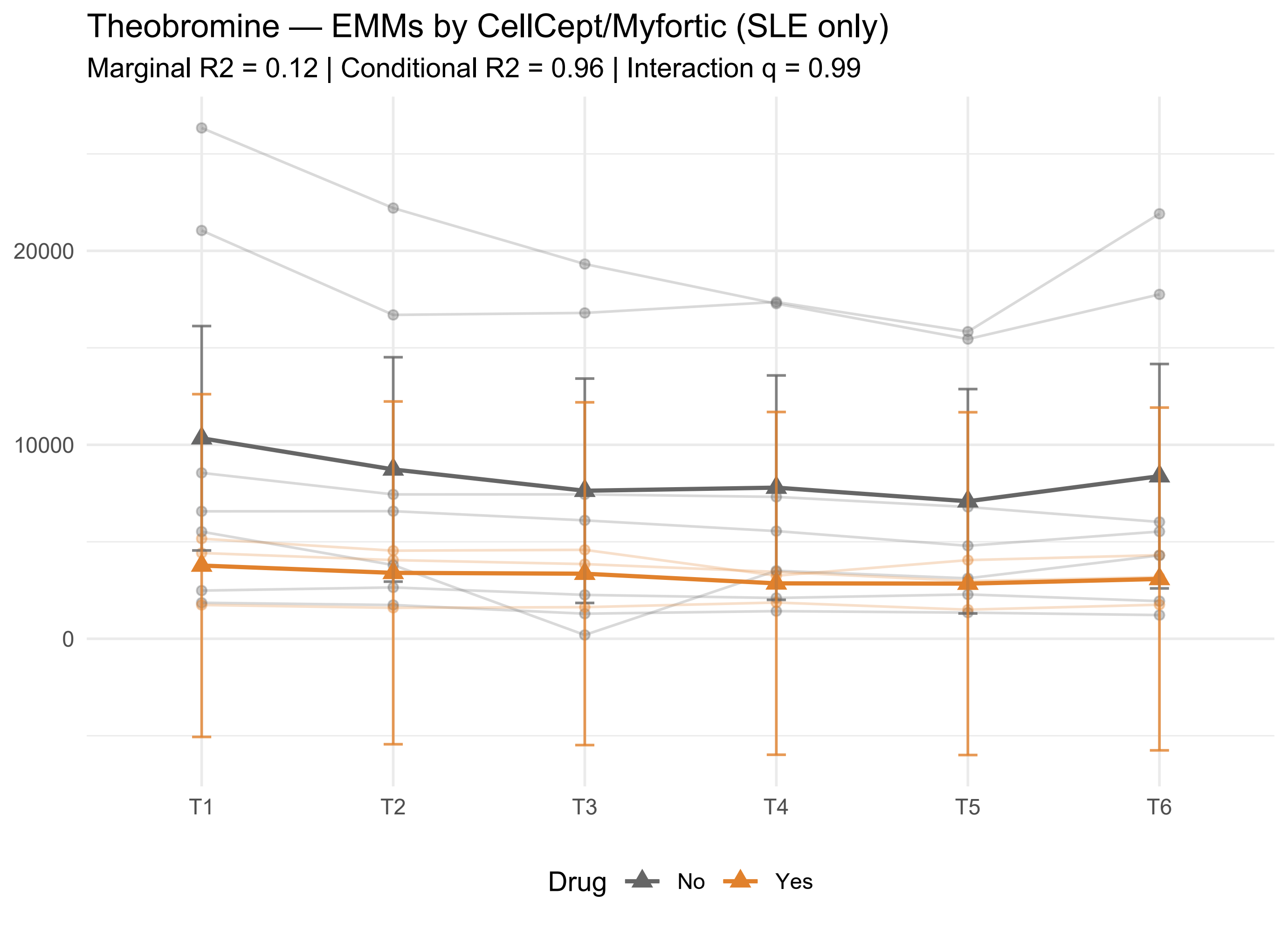

Theobromine — EMMs by CellCept/Myfortic (SLE only)
Marginal R2 = 0.12 | Conditional R2 = 0.96 | Interaction q = 0.99
20000
10000
0
T1
T2
T3
T4
T5
T6
Drug
No
Yes

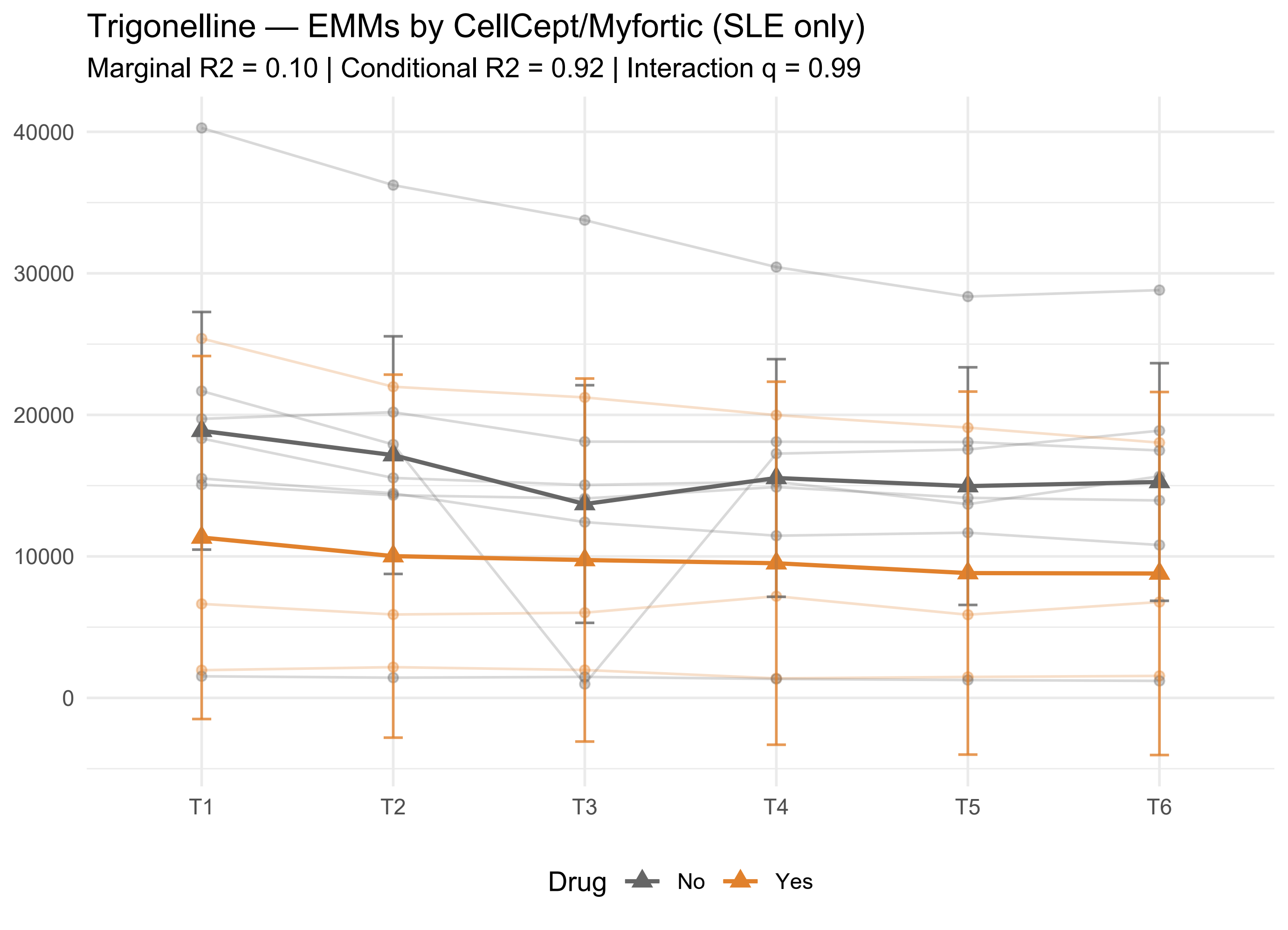

Trigonelline — EMMs by CellCept/Myfortic (SLE only)
Marginal R2 = 0.10 | Conditional R2 = 0.92 | Interaction q = 0.99
40000
30000
20000
10000
0
T1
T2
T3
T4
T5
T6
Drug
No
Yes

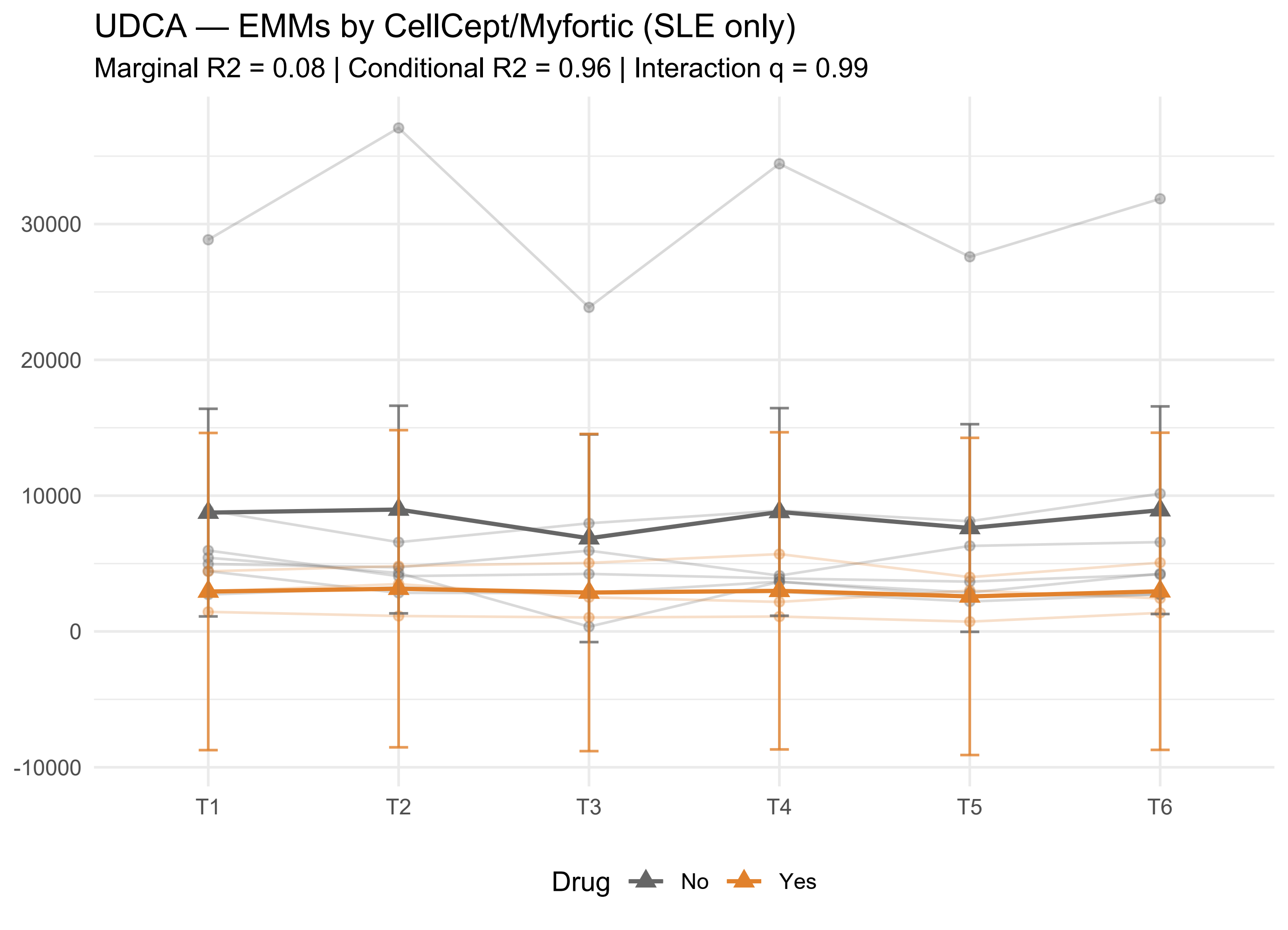

UDCA — EMMs by CellCept/Myfortic (SLE only)
Marginal R2 = 0.08 | Conditional R2 = 0.96 | Interaction q = 0.99
30000
20000
10000
0
-10000
T1
T2
T3
T4
T5
T6
Drug
No
Yes

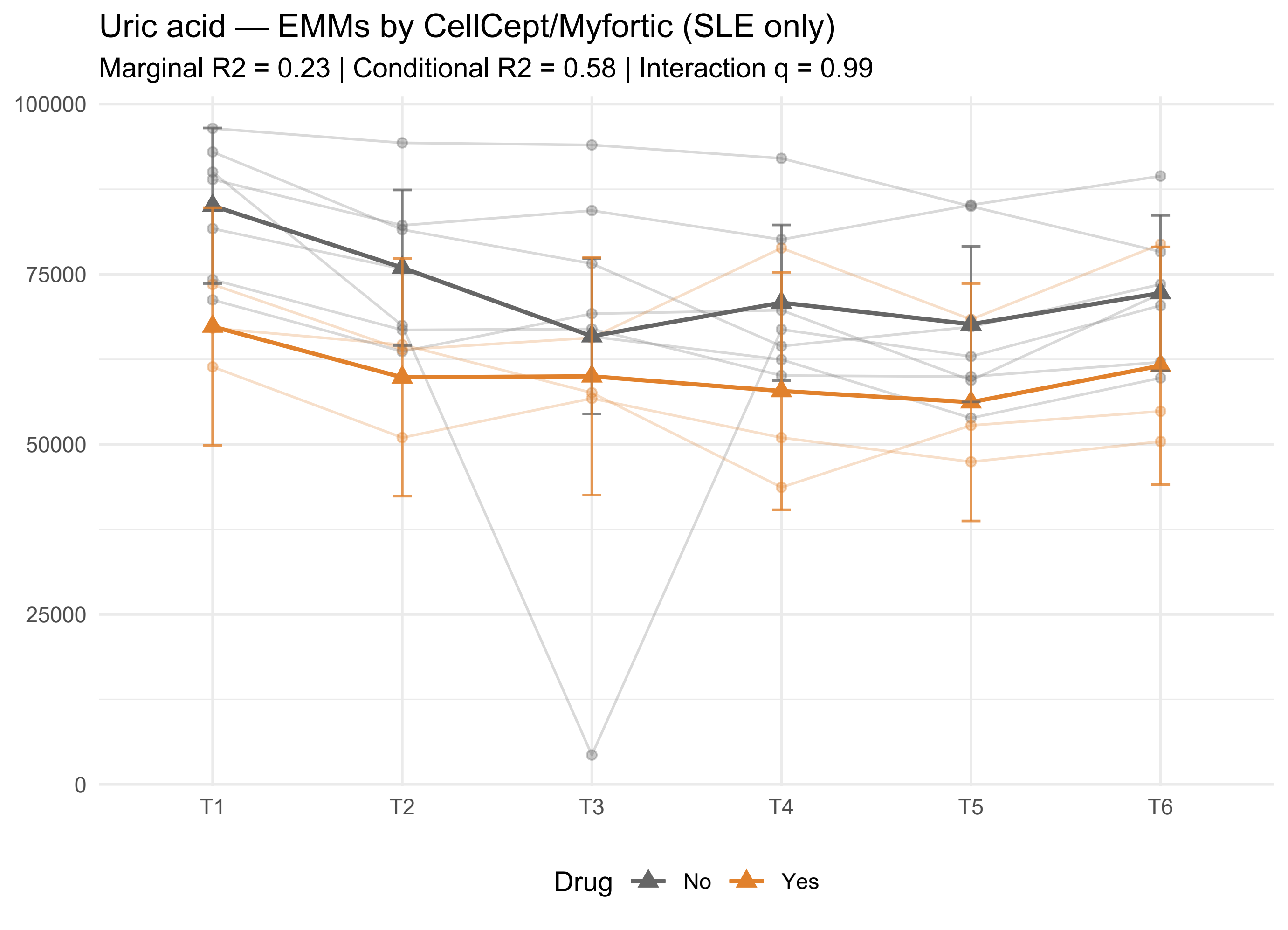

Uric acid — EMMs by CellCept/Myfortic (SLE only)
Marginal R2 = 0.23 | Conditional R2 = 0.58 | Interaction q = 0.99
100000
75000
50000
25000
0
T1
T2
T3
T4
T5
T6
Drug
No
Yes

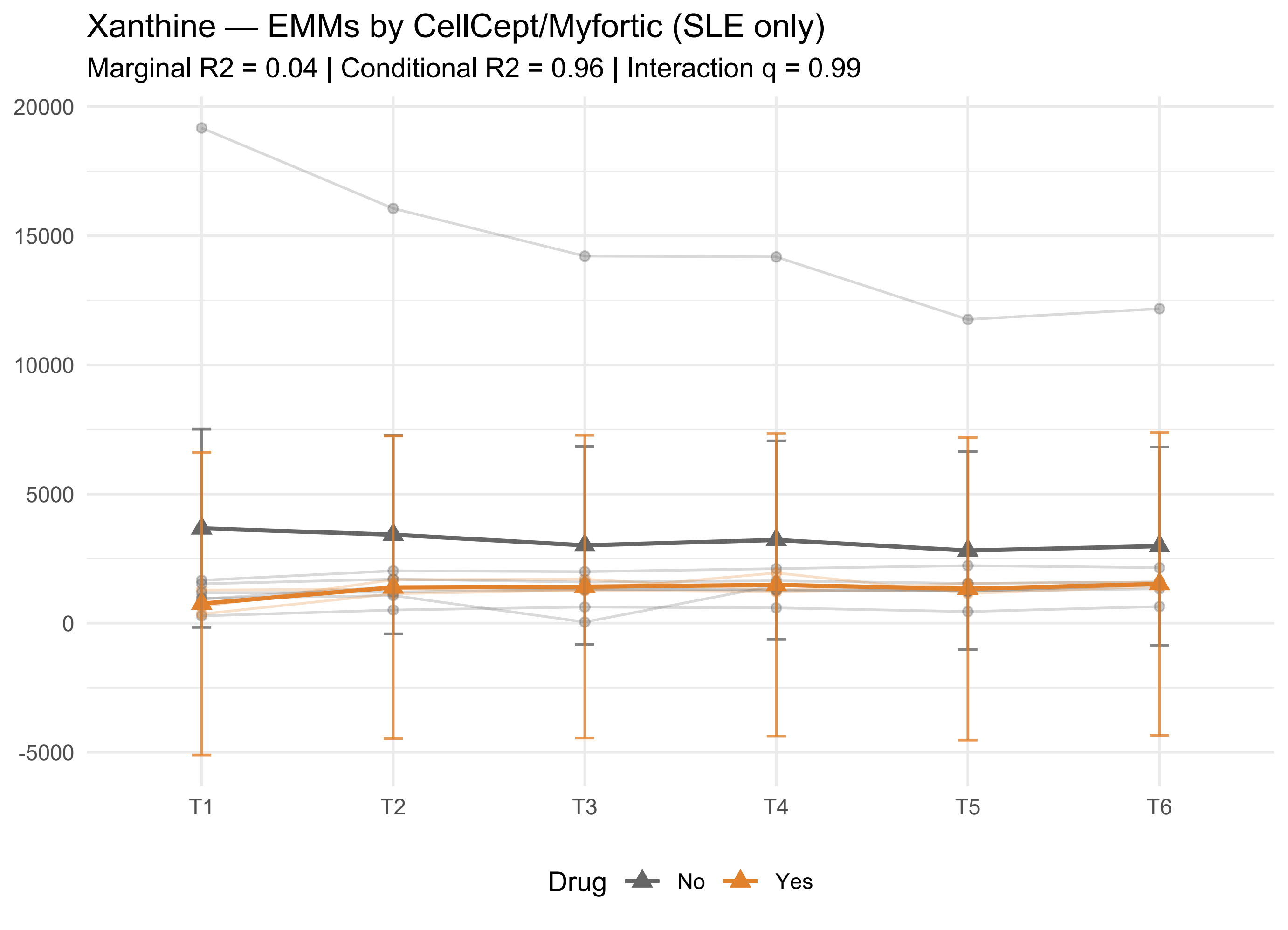

Xanthine — EMMs by CellCept/Myfortic (SLE only)
Marginal R2 = 0.04 | Conditional R2 = 0.96 | Interaction q = 0.99
20000
15000
10000
5000
0
-5000
T1
T2
T3
T4
T5
T6
Drug
No
Yes

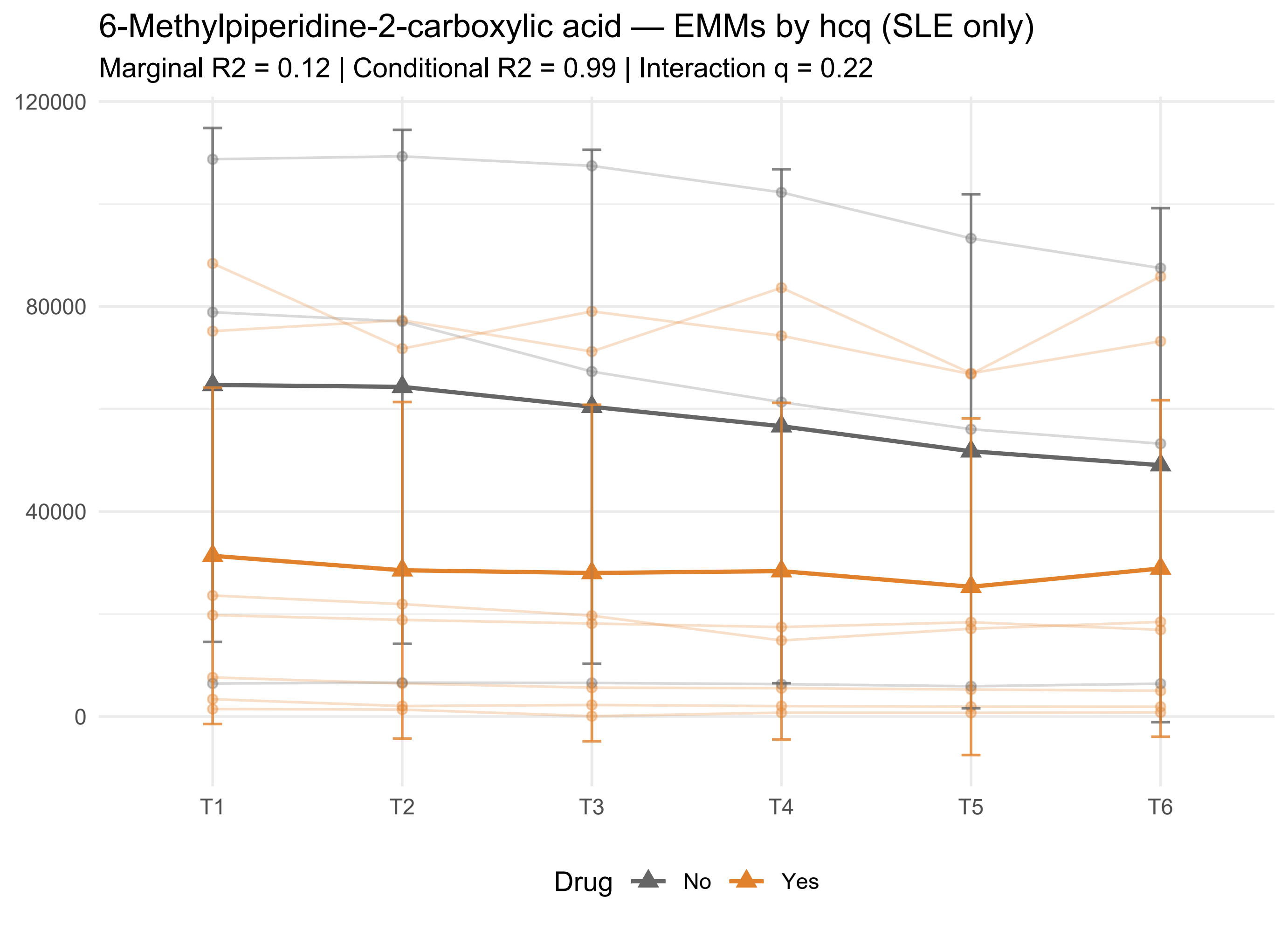

6-Methylpiperidine-2-carboxylic acid — EMMs by hcq (SLE only)
Marginal R2 = 0.12 | Conditional R2 = 0.99 | Interaction q = 0.22
120000
80000
40000
0
T1
T2
T3
T4
T5
T6
Drug
No
Yes

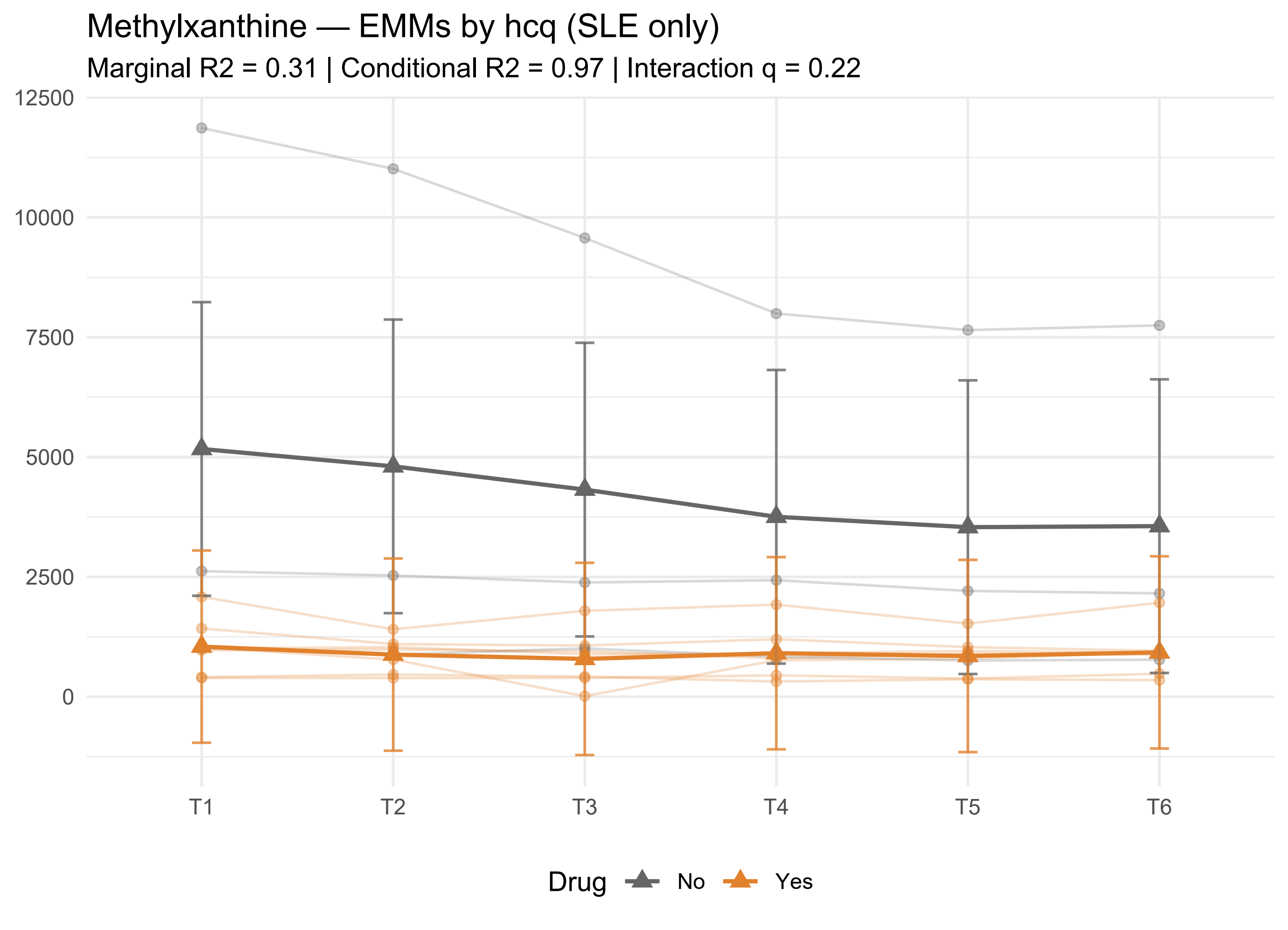

Methylxanthine — EMMs by hcq (SLE only)
Marginal R2 = 0.31 | Conditional R2 = 0.97 | Interaction q = 0.22
12500
10000
7500
5000
2500
0
T1
T2
T3
T4
T5
T6
Drug
No
Yes

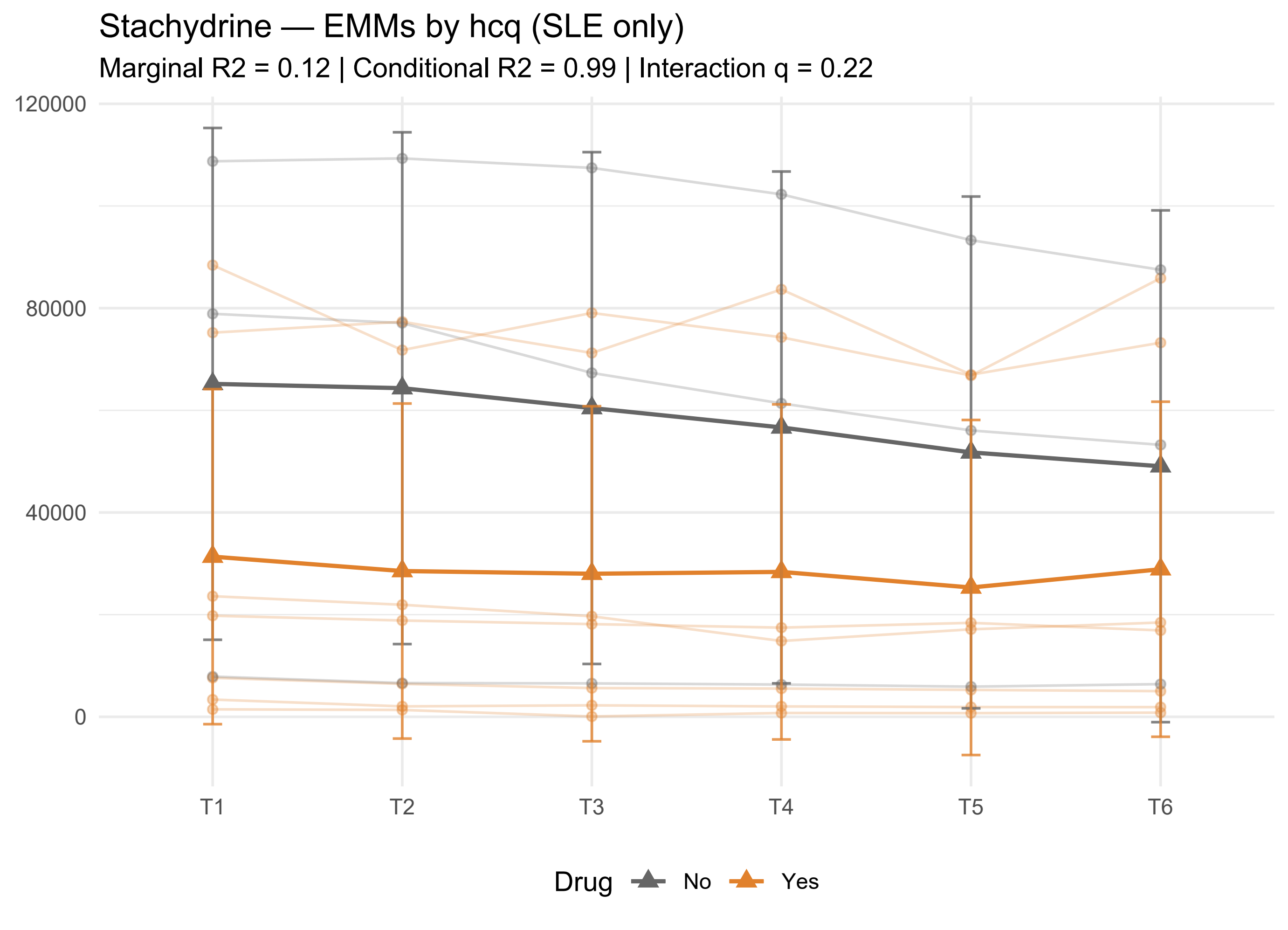

Stachydrine — EMMs by hcq (SLE only)
Marginal R2 = 0.12 | Conditional R2 = 0.99 | Interaction q = 0.22
120000
80000
40000
0
T1
T2
T3
T4
T5
T6
Drug
No
Yes

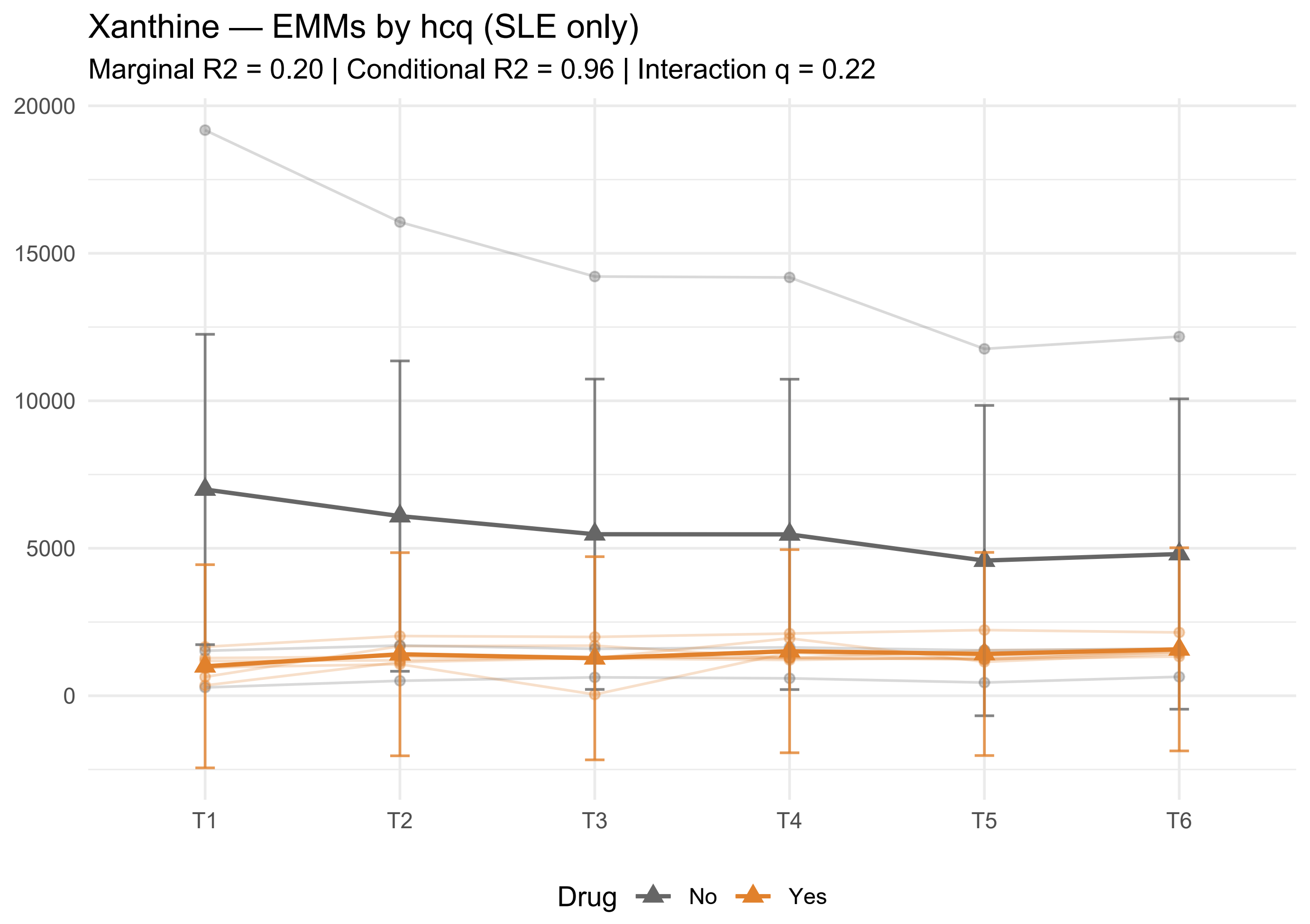
Xanthine — EMMs by hcq (SLE only)
Marginal R2 = 0.20 | Conditional R2 = 0.96 | Interaction q = 0.22
20000
15000
10000
5000
0
T1
T2
T3
T4
T5
T6
Drug
No
Yes

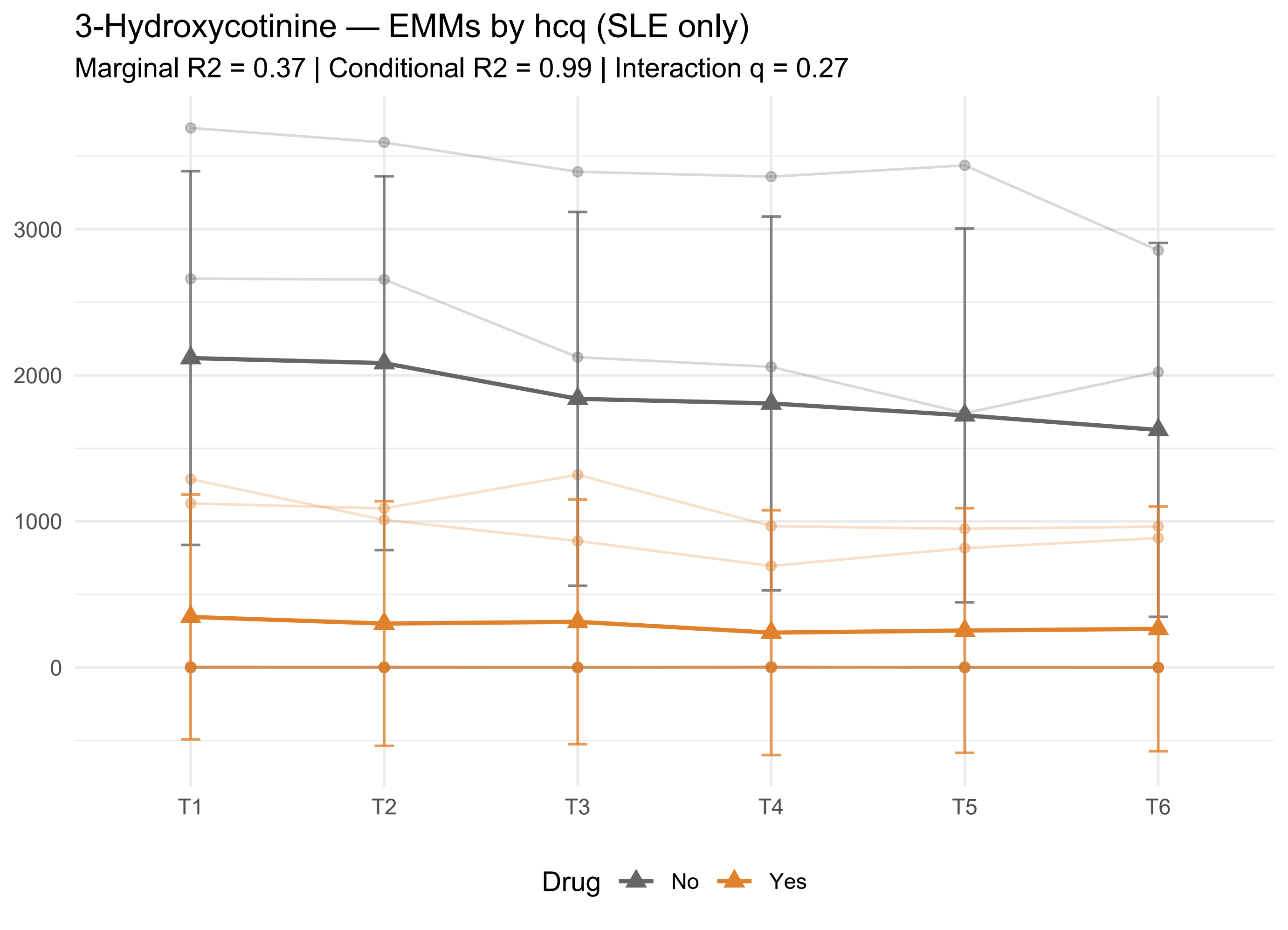
3-Hydroxycotinine — EMMs by hcq (SLE only)
Marginal R2 = 0.37 | Conditional R2 = 0.99 | Interaction q = 0.27
3000
2000
1000
0
T1
T2
T3
T4
T5
T6
Drug
No
Yes

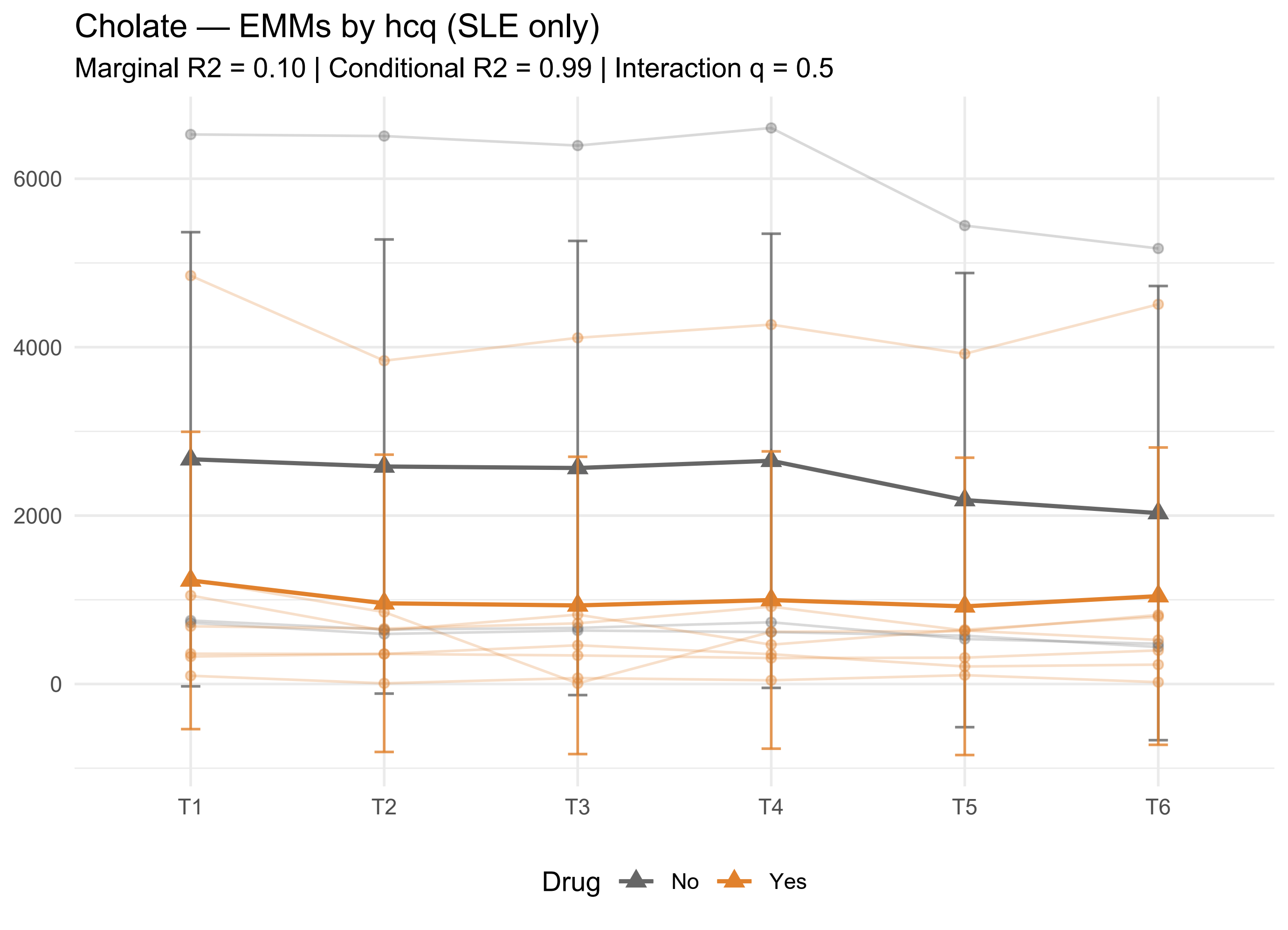

Cholate — EMMs by hcq (SLE only)
Marginal R2 = 0.10 | Conditional R2 = 0.99 | Interaction q = 0.5
6000
4000
2000
0
T1
T2
T3
T4
T5
T6
Drug
No
Yes

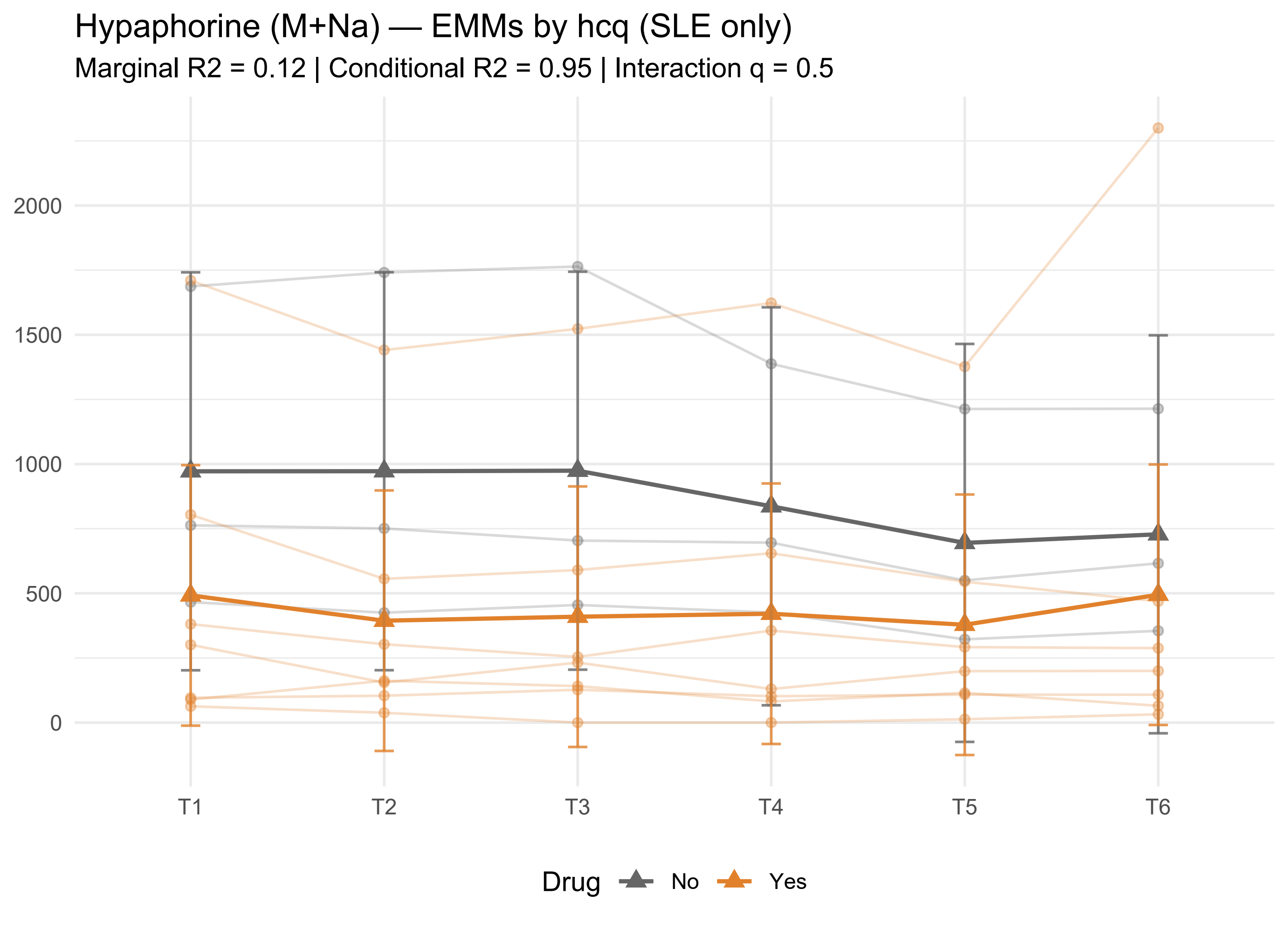

Hypaphorine (M+Na) — EMMs by hcq (SLE only)
Marginal R2 = 0.12 | Conditional R2 = 0.95 | Interaction q = 0.5
2000
1500
1000
500
0
T1
T2
T3
T4
T5
T6
Drug
No
Yes

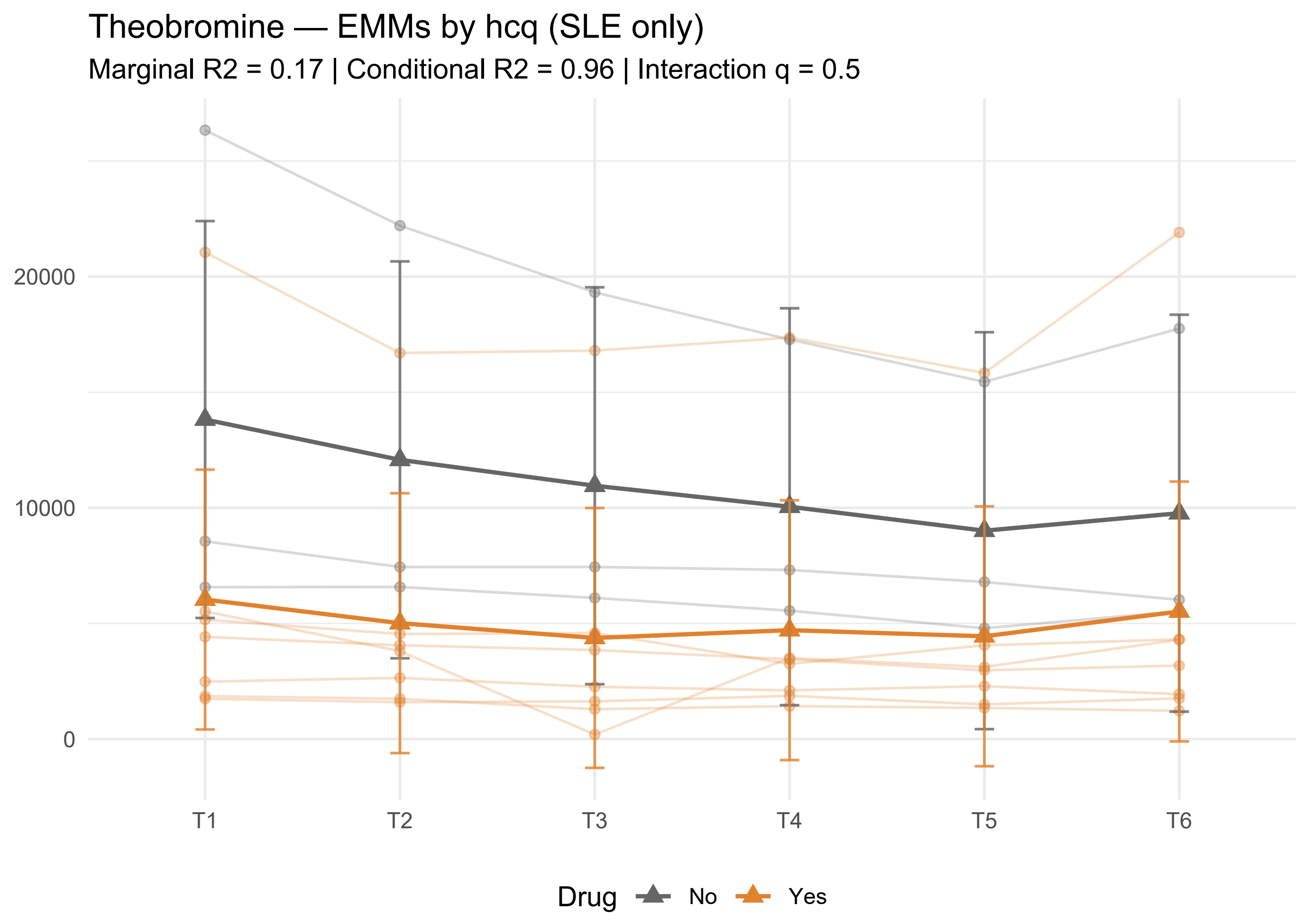

Theobromine — EMMs by hcq (SLE only)
Marginal R2 = 0.17 | Conditional R2 = 0.96 | Interaction q = 0.5
20000
10000
0
T1
T2
T3
T4
T5
T6
Drug
No
Yes

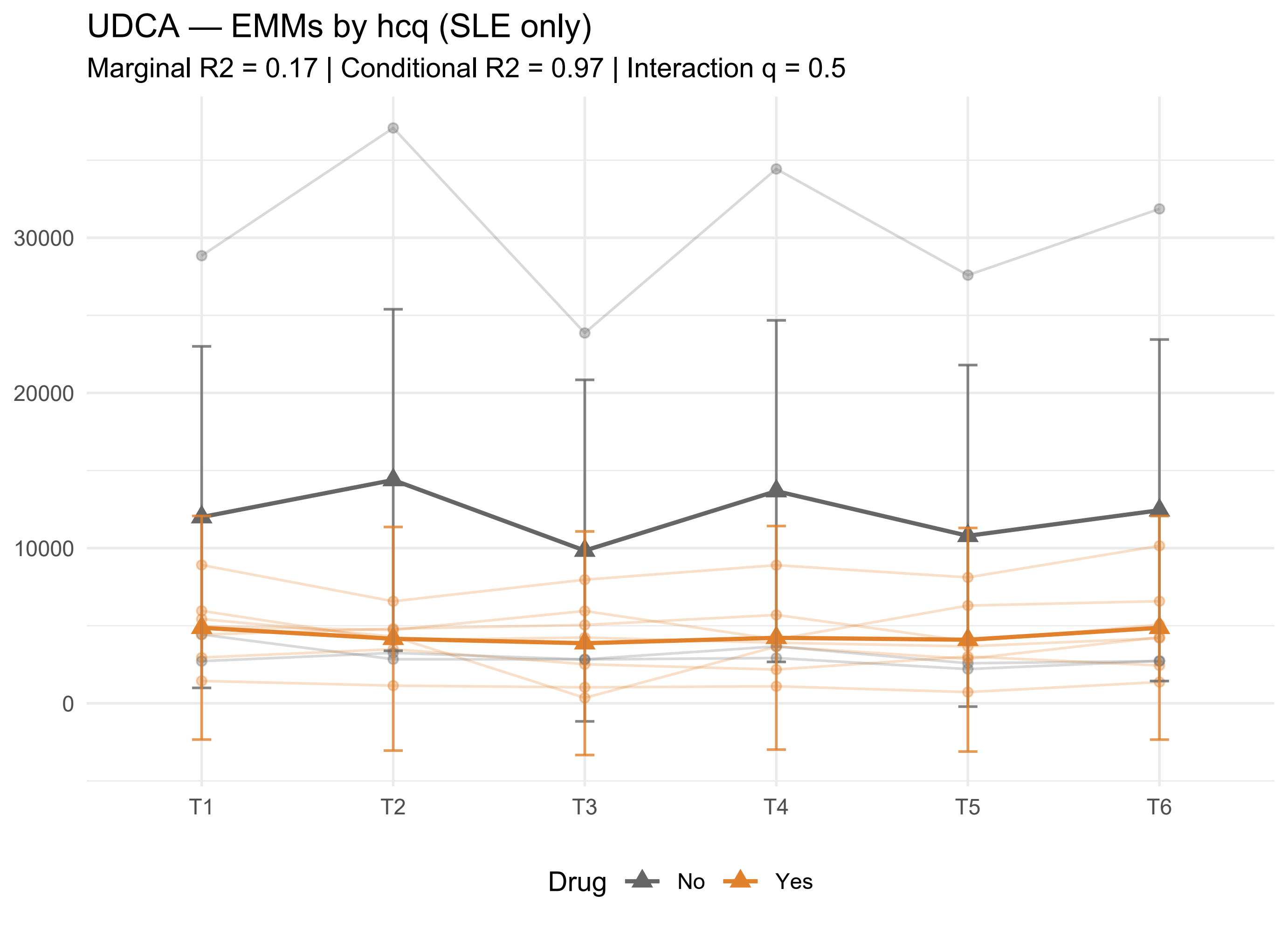
UDCA — EMMs by hcq (SLE only)
Marginal R2 = 0.17 | Conditional R2 = 0.97 | Interaction q = 0.5
30000
20000
10000
0
T1
T2
T3
T4
T5
T6
Drug
No
Yes

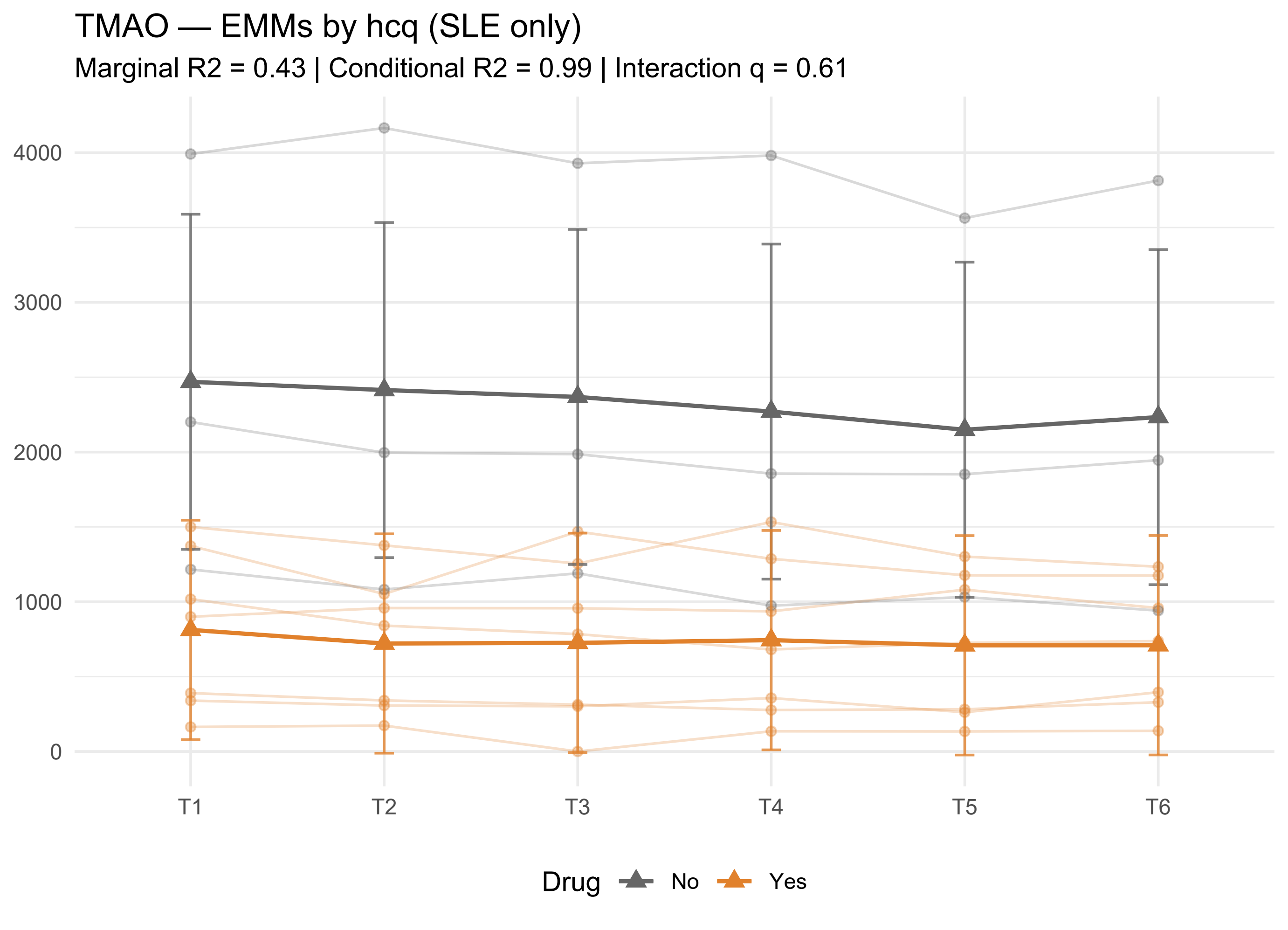
TMAO — EMMs by hcq (SLE only)
Marginal R2 = 0.43 | Conditional R2 = 0.99 | Interaction q = 0.61
4000
3000
2000
1000
0
T1
T2
T3
T4
T5
T6
Drug
No
Yes

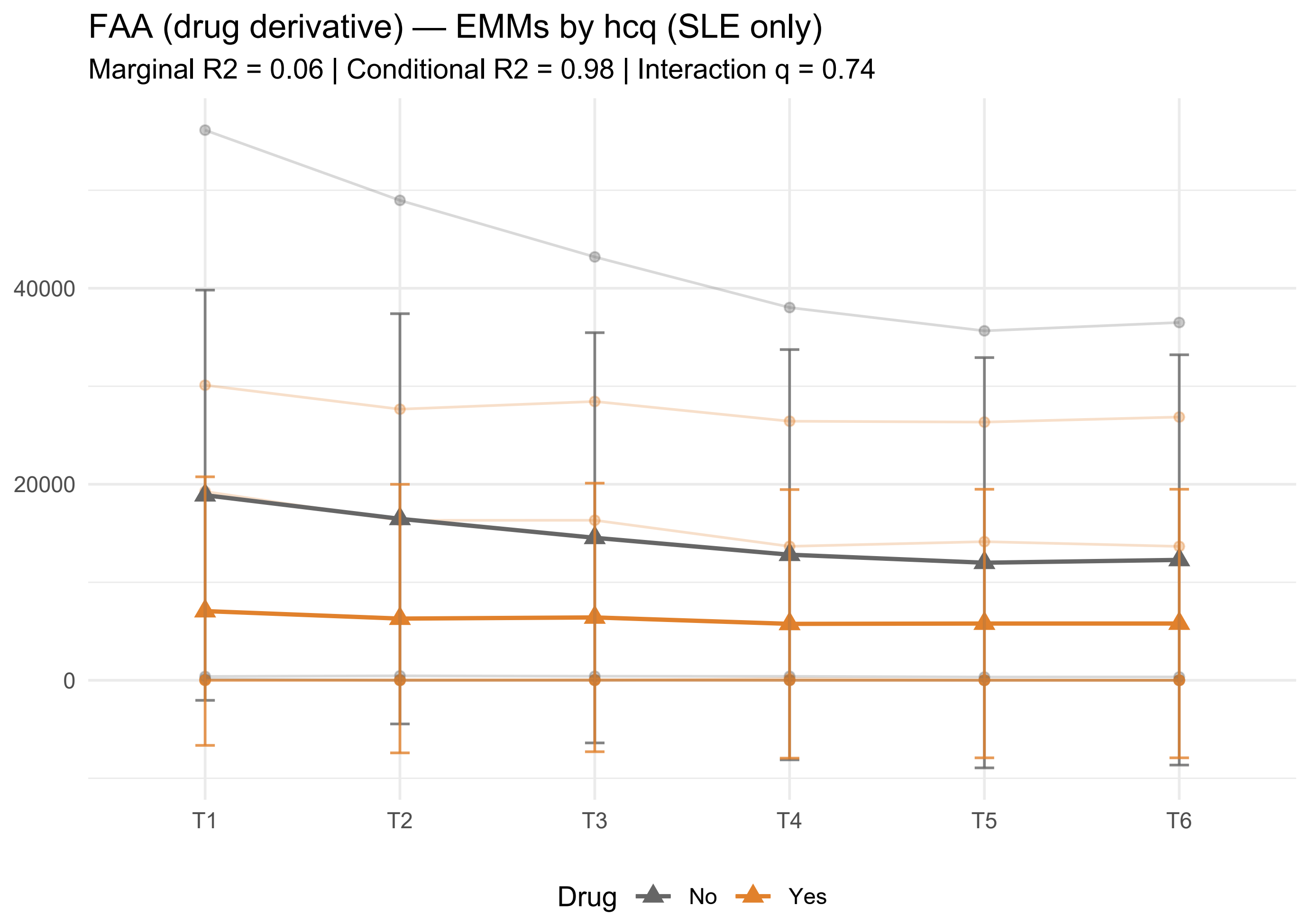
FAA (drug derivative) — EMMs by hcq (SLE only)
Marginal R2 = 0.06 | Conditional R2 = 0.98 | Interaction q = 0.74
40000
20000
0
T1
T2
T3
T4
T5
T6
Drug
No
Yes

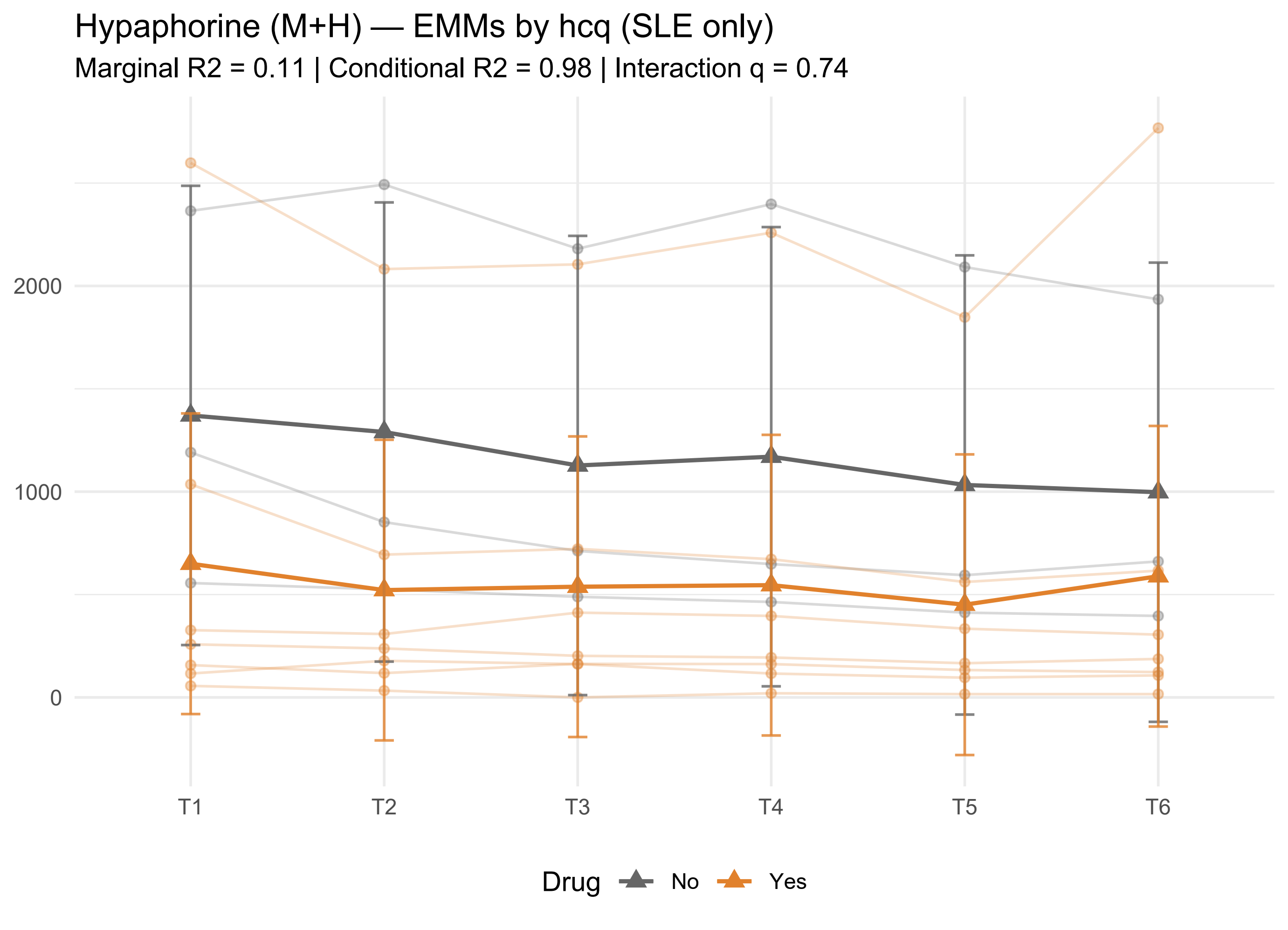

Hypaphorine (M+H) — EMMs by hcq (SLE only)
Marginal R2 = 0.11 | Conditional R2 = 0.98 | Interaction q = 0.74
2000
1000
0
T1
T2
T3
T4
T5
T6
Drug
No
Yes

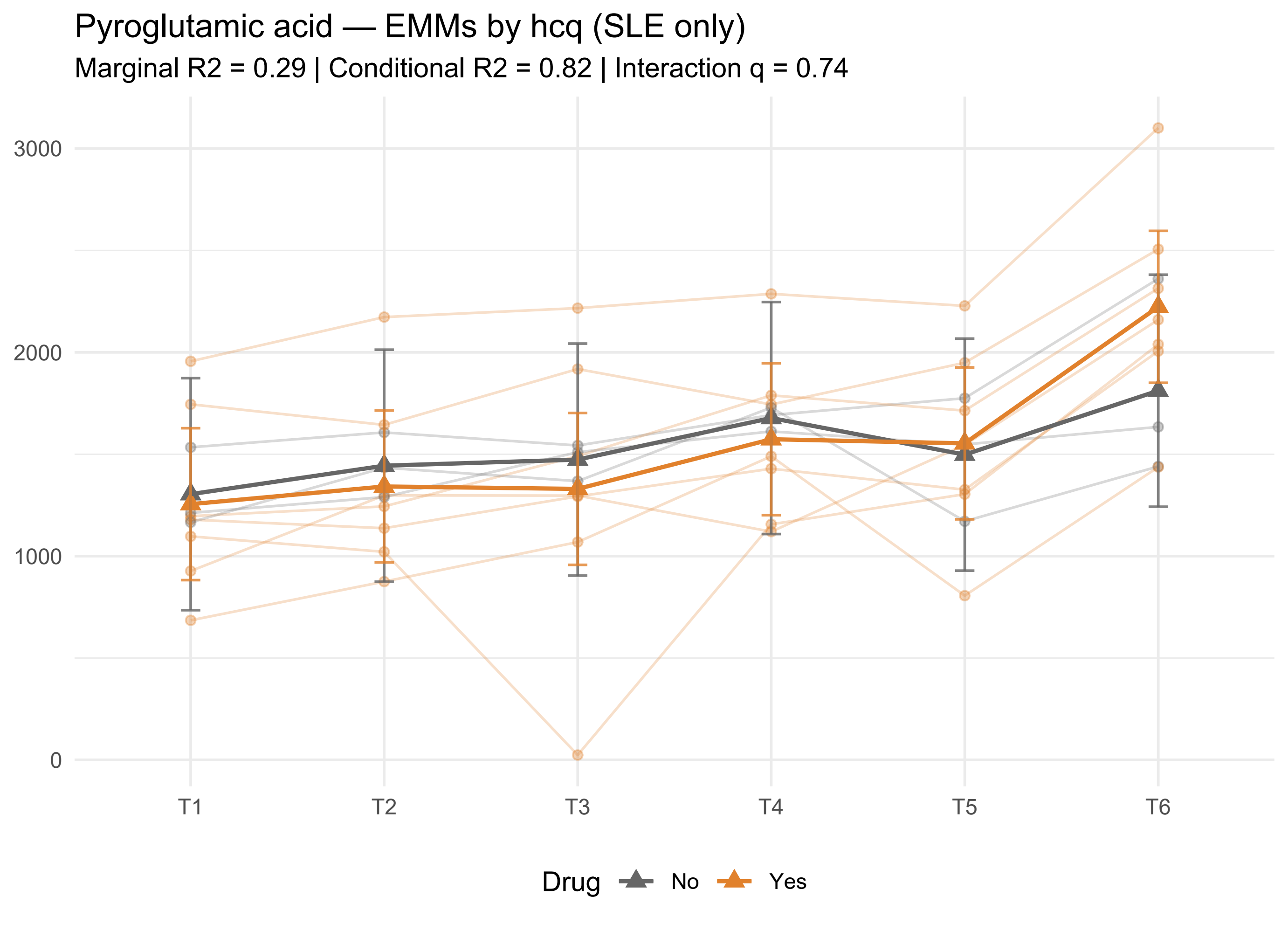

Pyroglutamic acid — EMMs by hcq (SLE only)
Marginal R2 = 0.29 | Conditional R2 = 0.82 | Interaction q = 0.74
3000
2000
1000
0
T1
T2
T3
T4
T5
T6
Drug
No
Yes

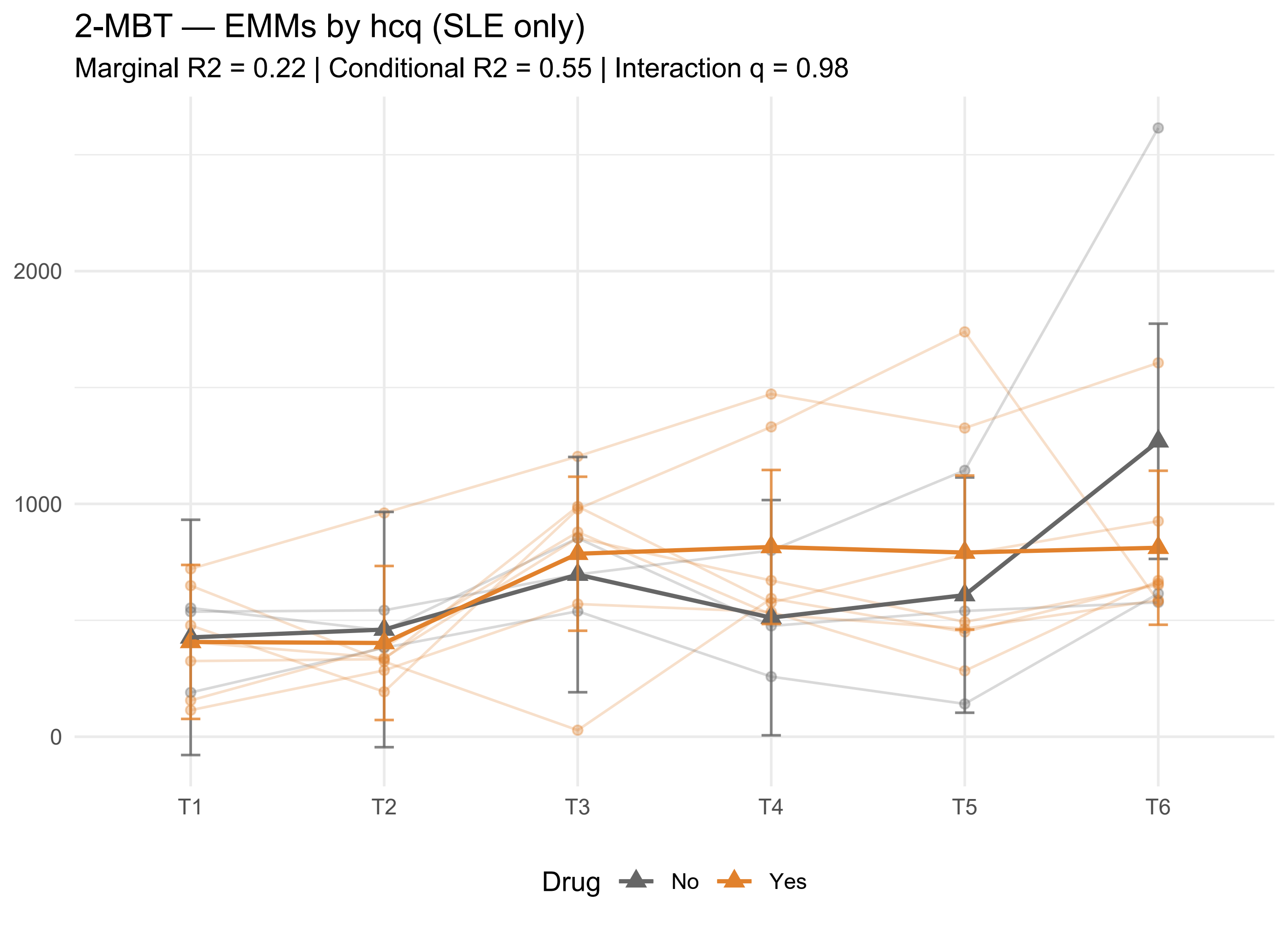
2-MBT — EMMs by hcq (SLE only)
Marginal R2 = 0.22 | Conditional R2 = 0.55 | Interaction q = 0.98
2000
1000
0
T1
T2
T3
T4
T5
T6
Drug
No
Yes

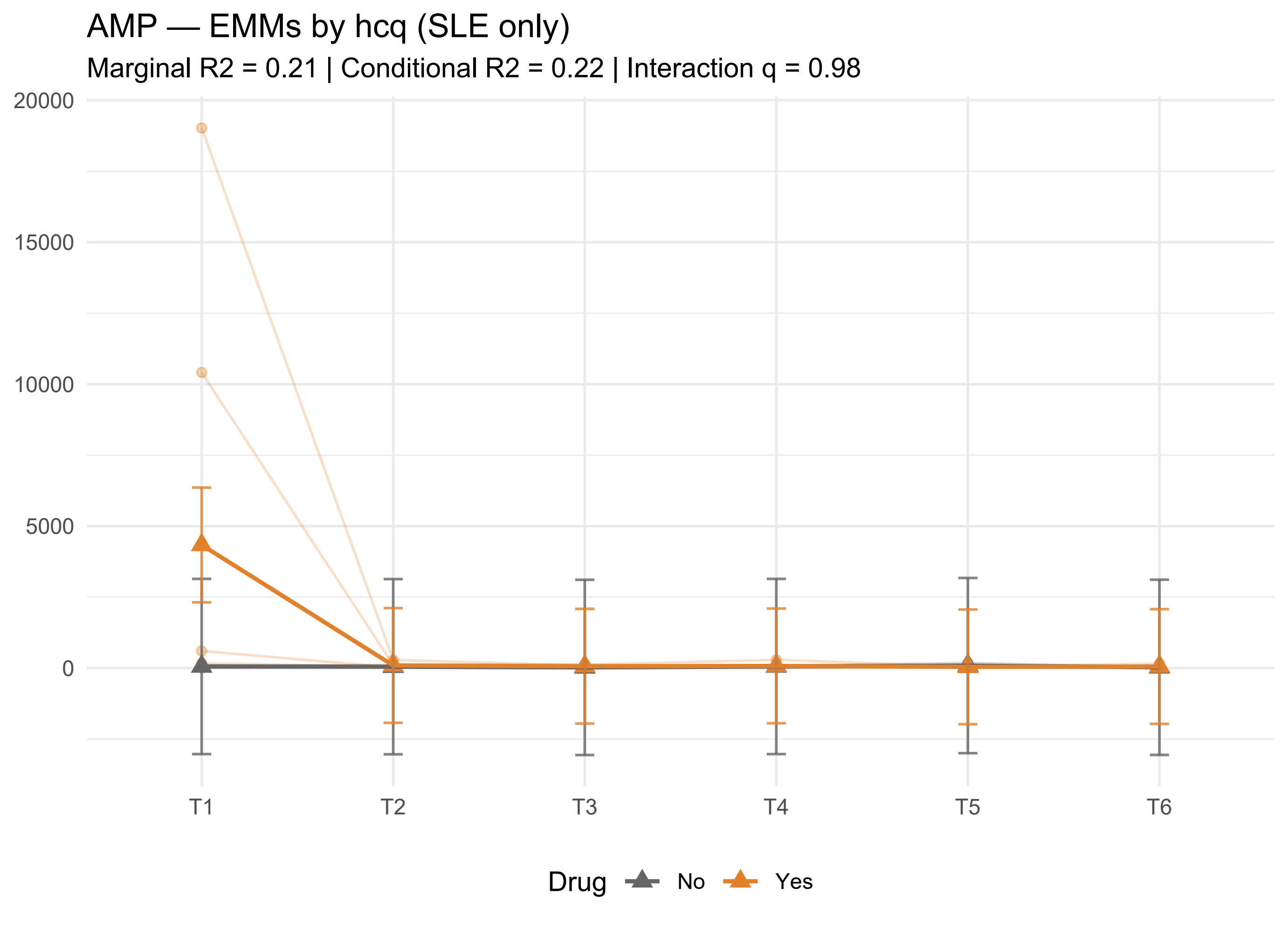

AMP — EMMs by hcq (SLE only)
Marginal R2 = 0.21 | Conditional R2 = 0.22 | Interaction q = 0.98
20000
15000
10000
5000
0
T1
T2
T3
T4
T5
T6
Drug
No
Yes

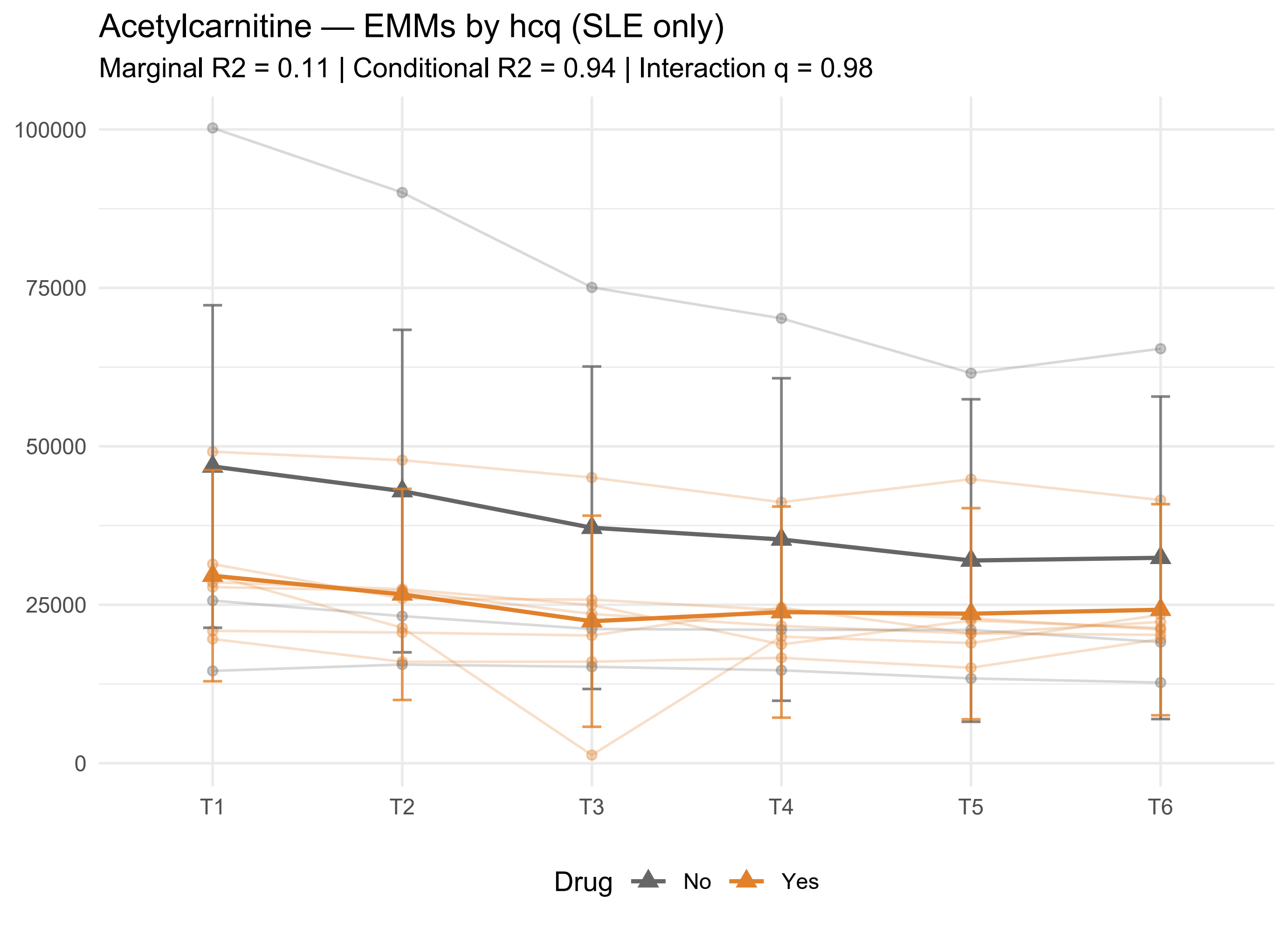

Acetylcarnitine — EMMs by hcq (SLE only)
Marginal R2 = 0.11 | Conditional R2 = 0.94 | Interaction q = 0.98
100000
75000
50000
25000
0
T1
T2
T3
T4
T5
T6
Drug
No
Yes

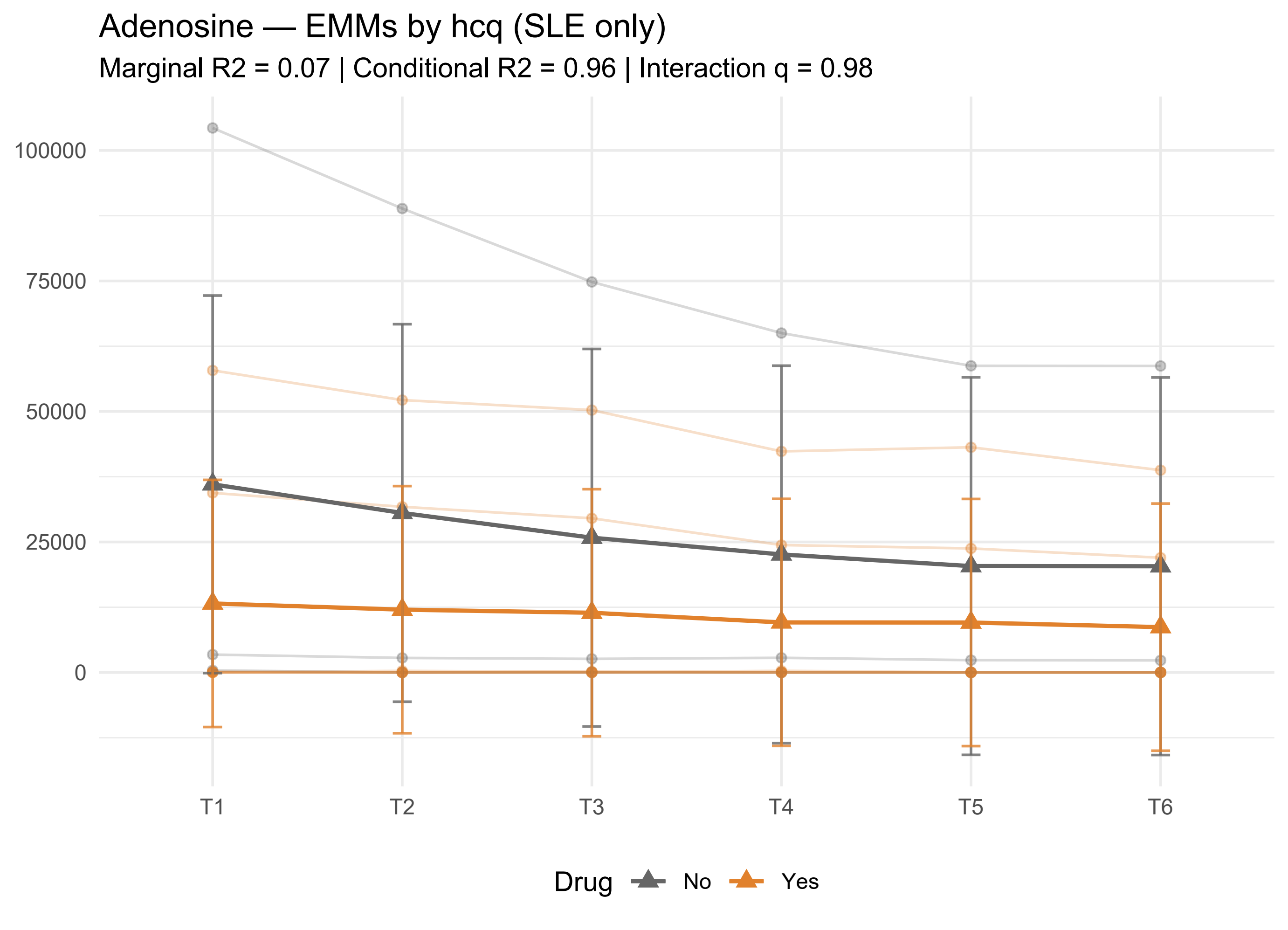

Adenosine — EMMs by hcq (SLE only)
Marginal R2 = 0.07 | Conditional R2 = 0.96 | Interaction q = 0.98
100000
75000
50000
25000
0
T1
T2
T3
T4
T5
T6
Drug
No
Yes

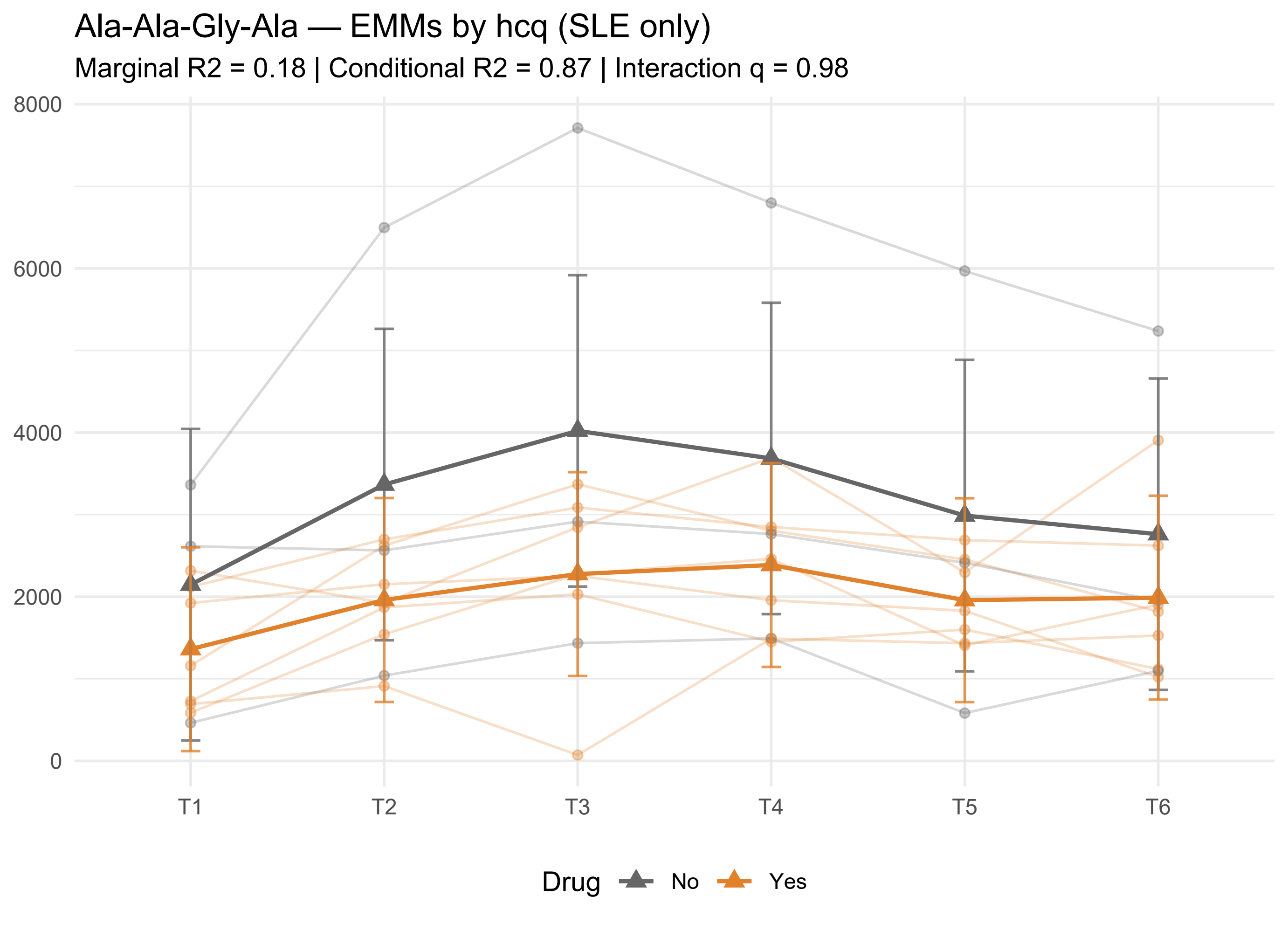

Ala-Ala-Gly-Ala — EMMs by hcq (SLE only)
Marginal R2 = 0.18 | Conditional R2 = 0.87 | Interaction q = 0.98
8000
6000
4000
2000
0
T1
T2
T3
T4
T5
T6
Drug
No
Yes

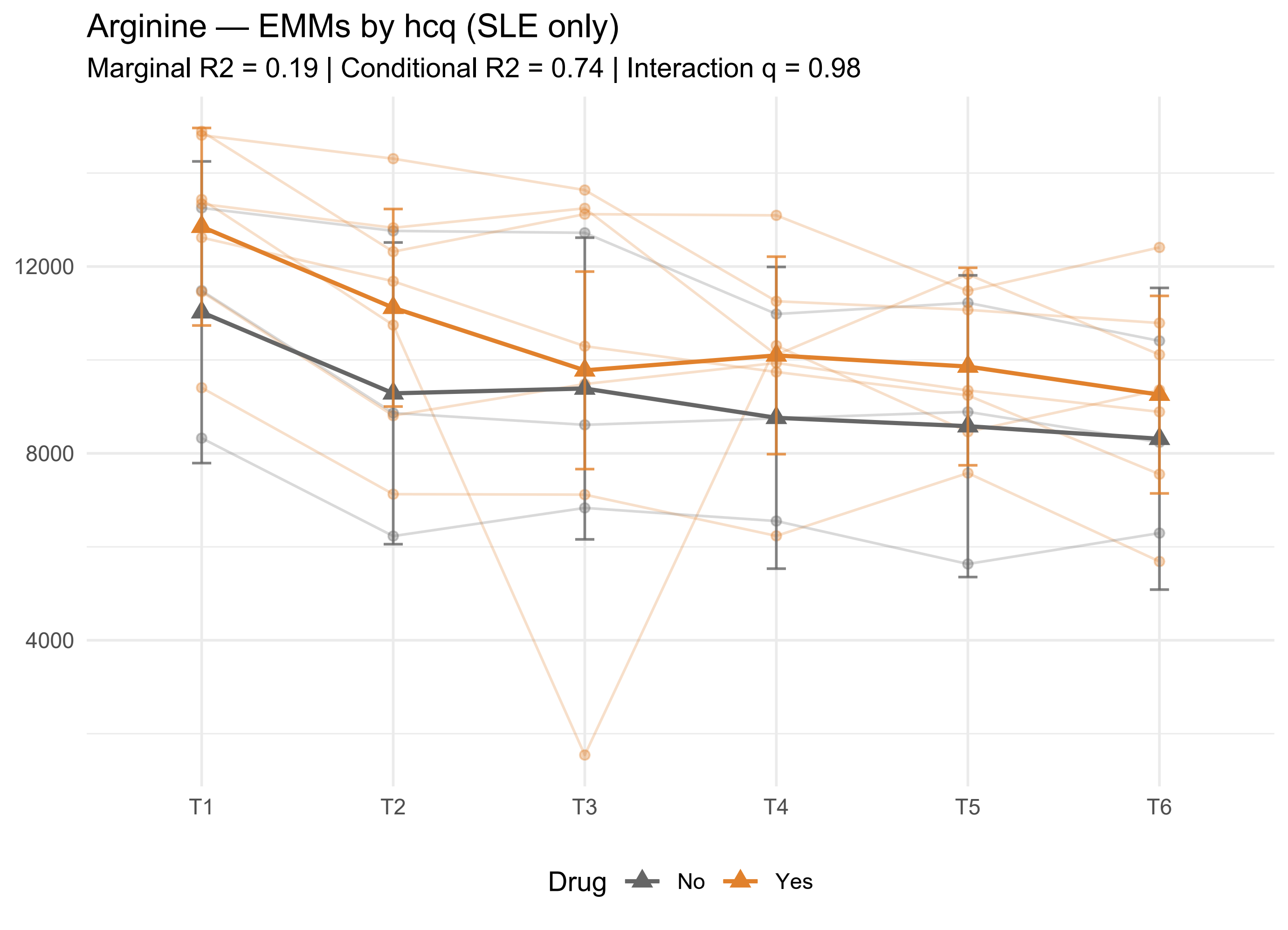

Arginine — EMMs by hcq (SLE only)
Marginal R2 = 0.19 | Conditional R2 = 0.74 | Interaction q = 0.98
12000
8000
4000
T1
T2
T3
T4
T5
T6
Drug
No
Yes

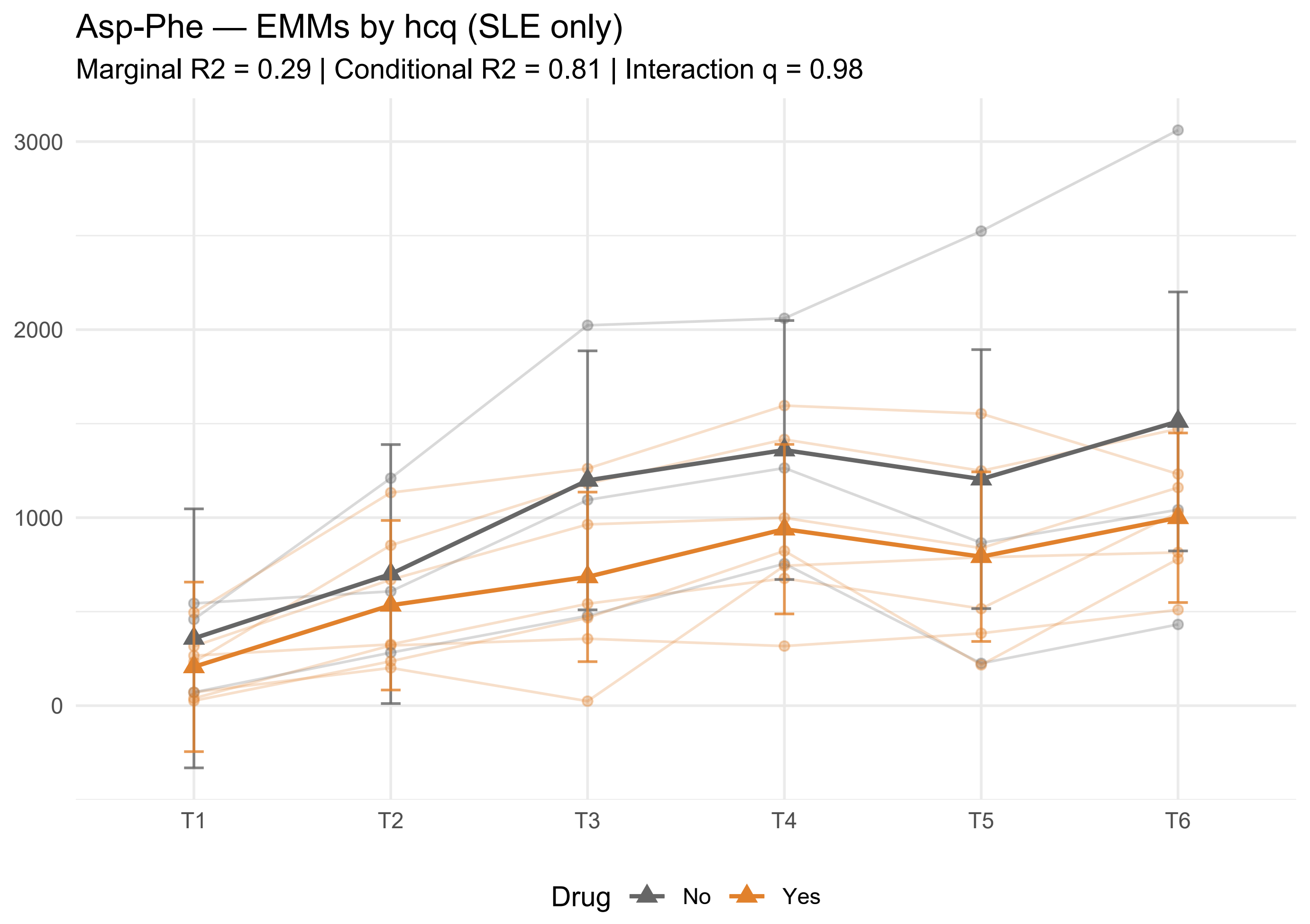

Asp-Phe — EMMs by hcq (SLE only)
Marginal R2 = 0.29 | Conditional R2 = 0.81 | Interaction q = 0.98
3000
2000
1000
0
T1
T2
T3
T4
T5
T6
Drug
No
Yes

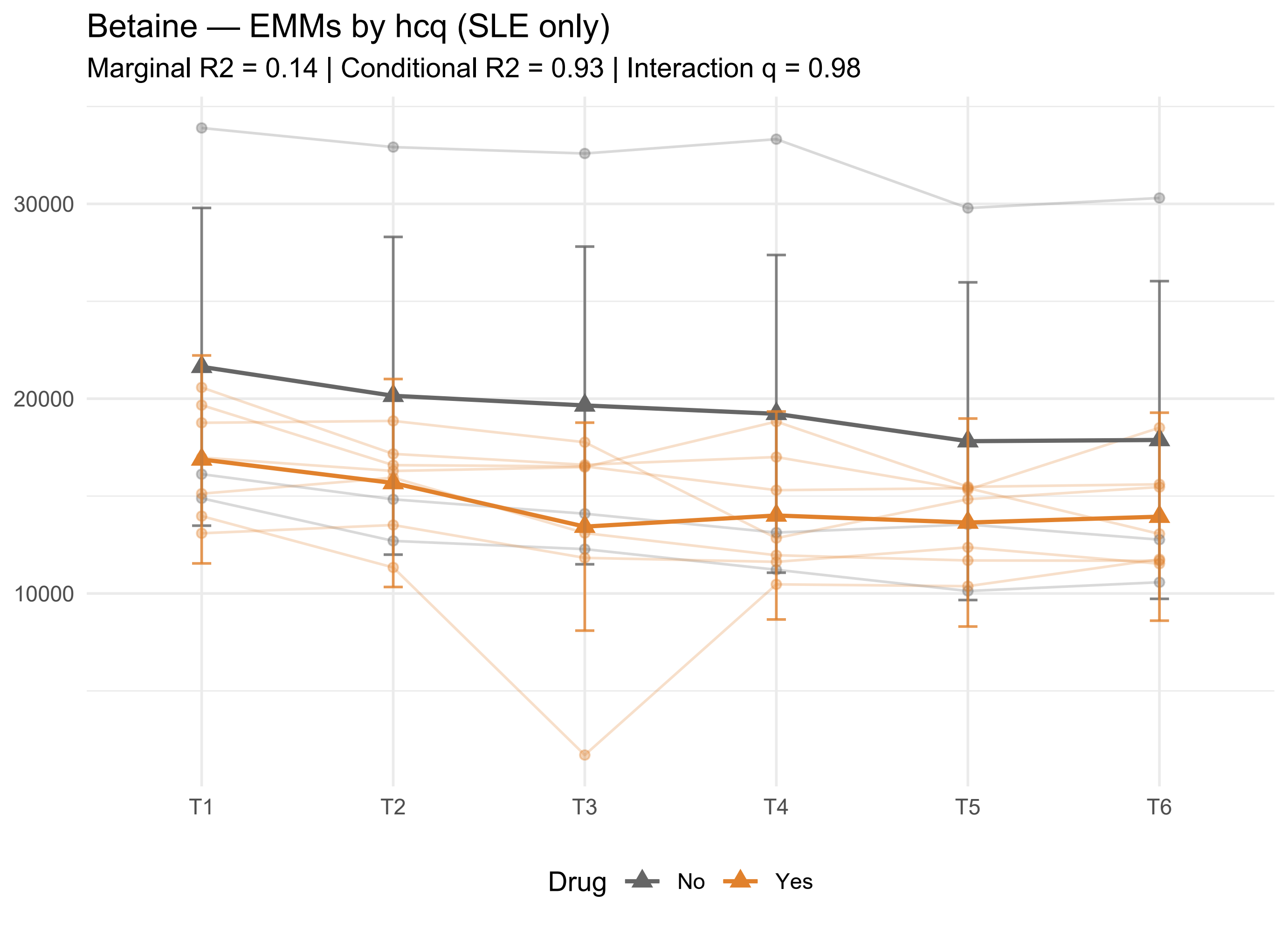

Betaine — EMMs by hcq (SLE only)
Marginal R2 = 0.14 | Conditional R2 = 0.93 | Interaction q = 0.98
30000
20000
10000
T1
T2
T3
T4
T5
T6
Drug
No
Yes

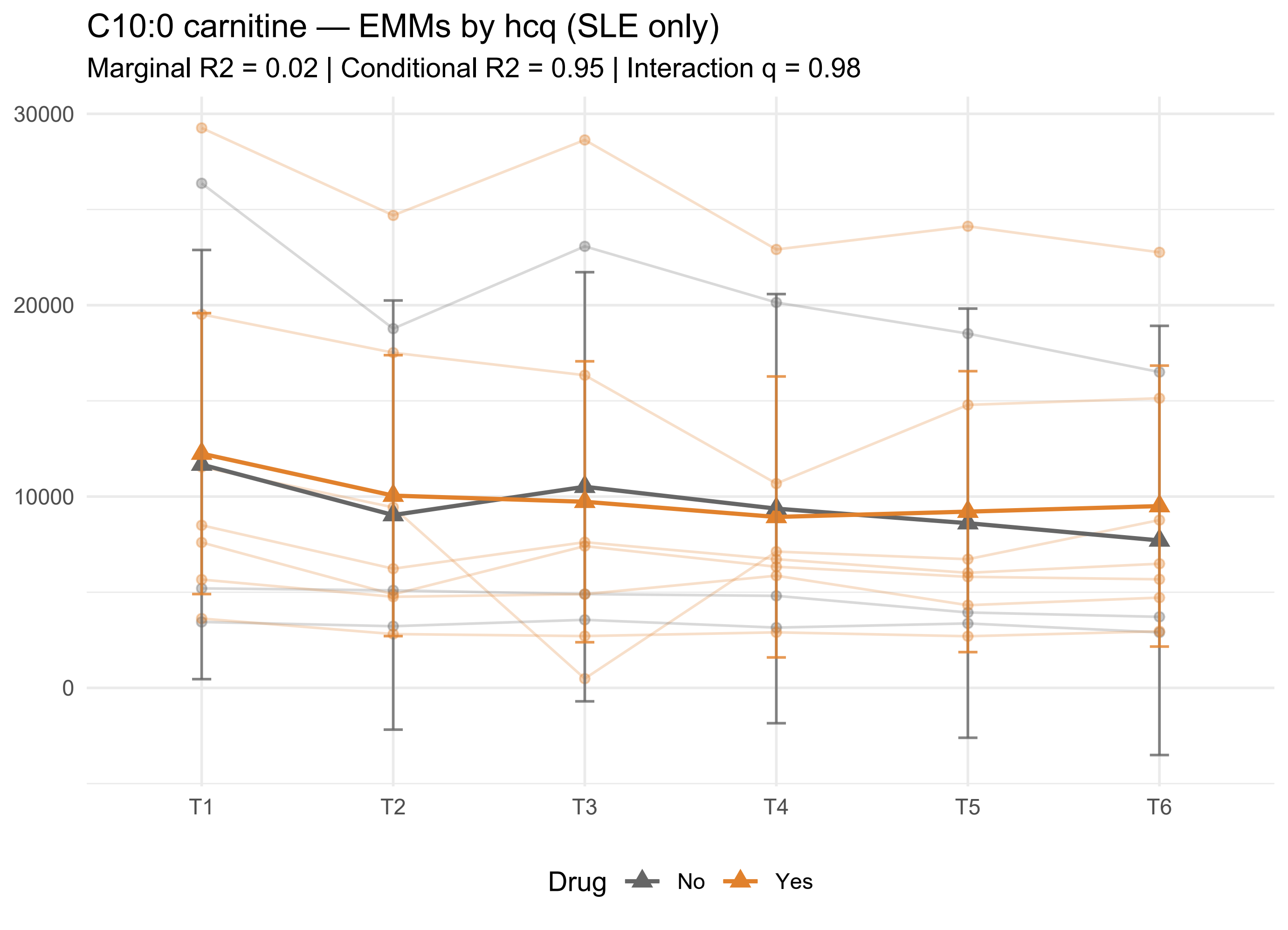
C10:0 carnitine — EMMs by hcq (SLE only)
Marginal R2 = 0.02 | Conditional R2 = 0.95 | Interaction q = 0.98
30000
20000
10000
0
T1
T2
T3
T4
T5
T6
Drug
No
Yes

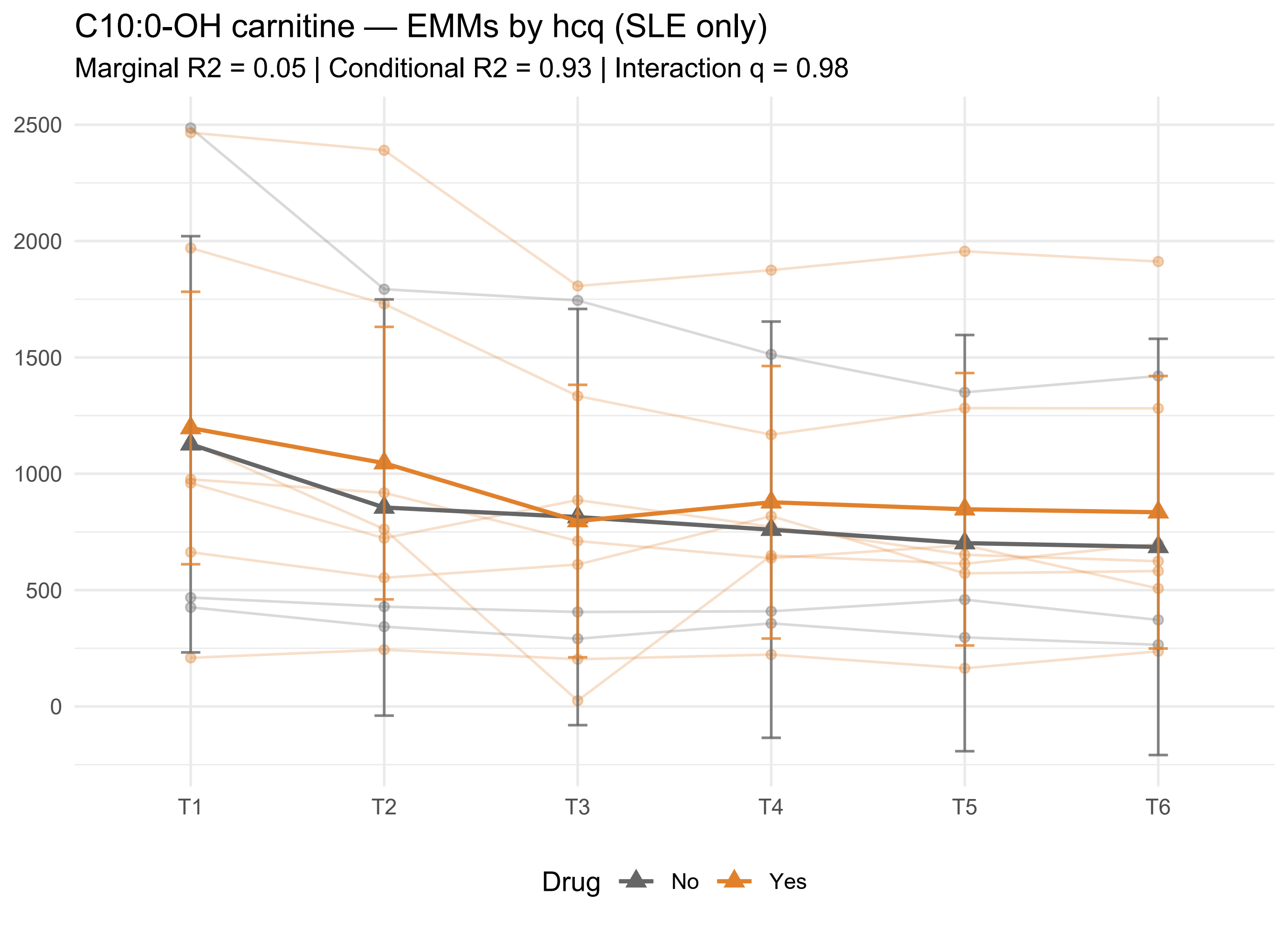

C10:0-OH carnitine — EMMs by hcq (SLE only)
Marginal R2 = 0.05 | Conditional R2 = 0.93 | Interaction q = 0.98
2500
2000
1500
1000
500
0
T1
T2
T3
T4
T5
T6
Drug
No
Yes

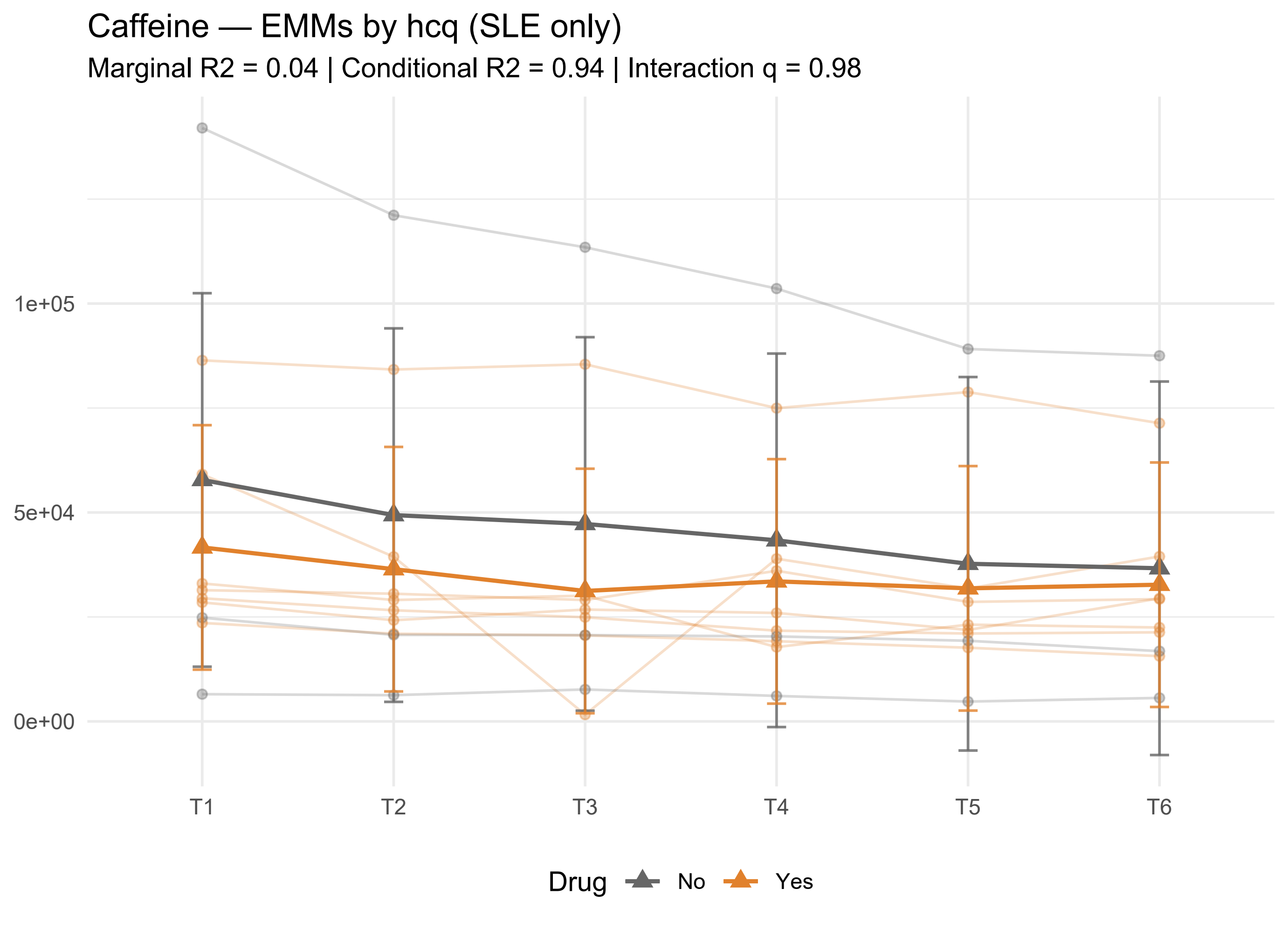
Caffeine — EMMs by hcq (SLE only)
Marginal R2 = 0.04 | Conditional R2 = 0.94 | Interaction q = 0.98
1e+05
5e+04
0e+00
T1
T2
T3
T4
T5
T6
Drug
No
Yes

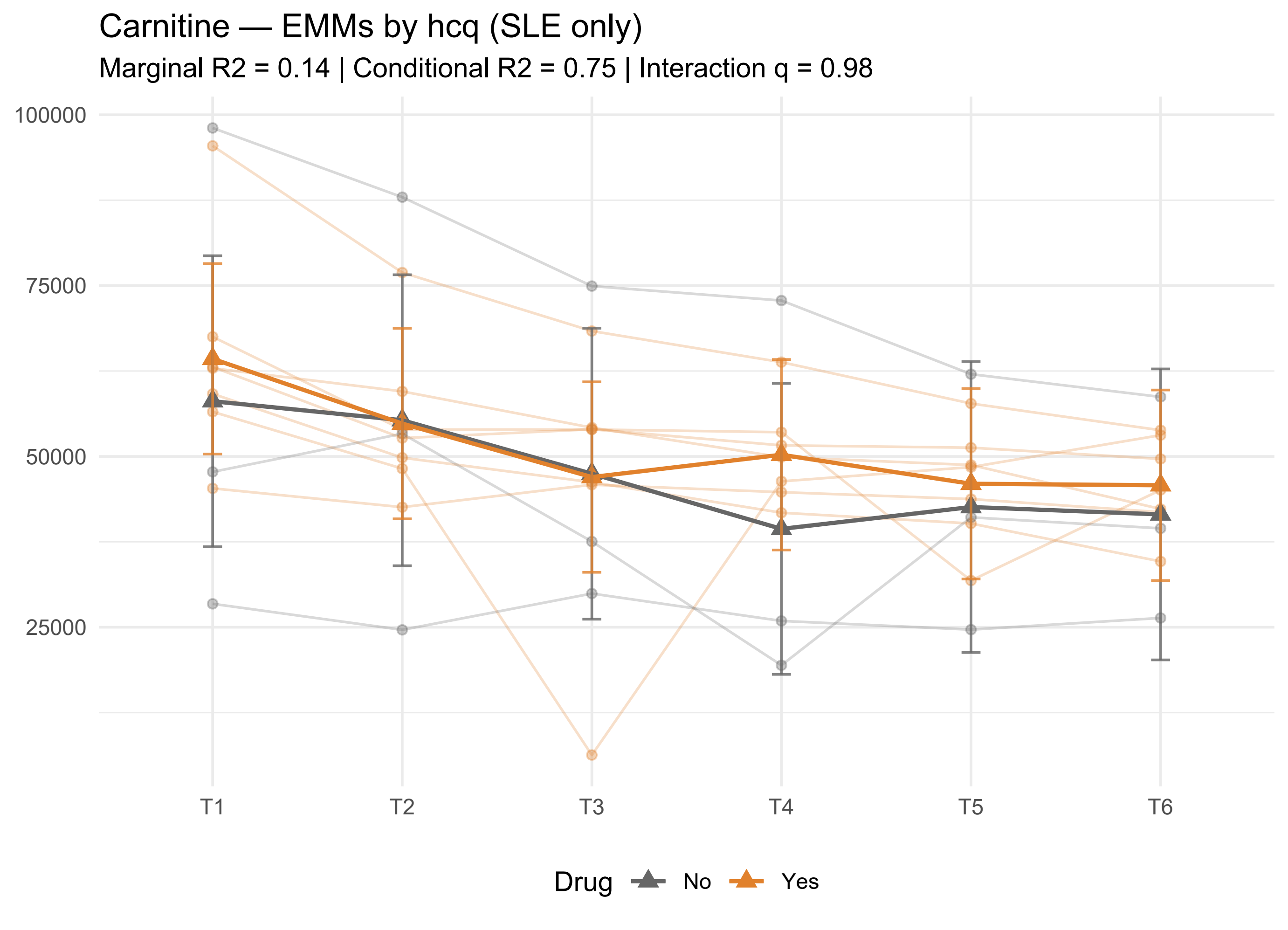

Carnitine — EMMs by hcq (SLE only)
Marginal R2 = 0.14 | Conditional R2 = 0.75 | Interaction q = 0.98
100000
75000
50000
25000
T1
T2
T3
T4
T5
T6
Drug
No
Yes

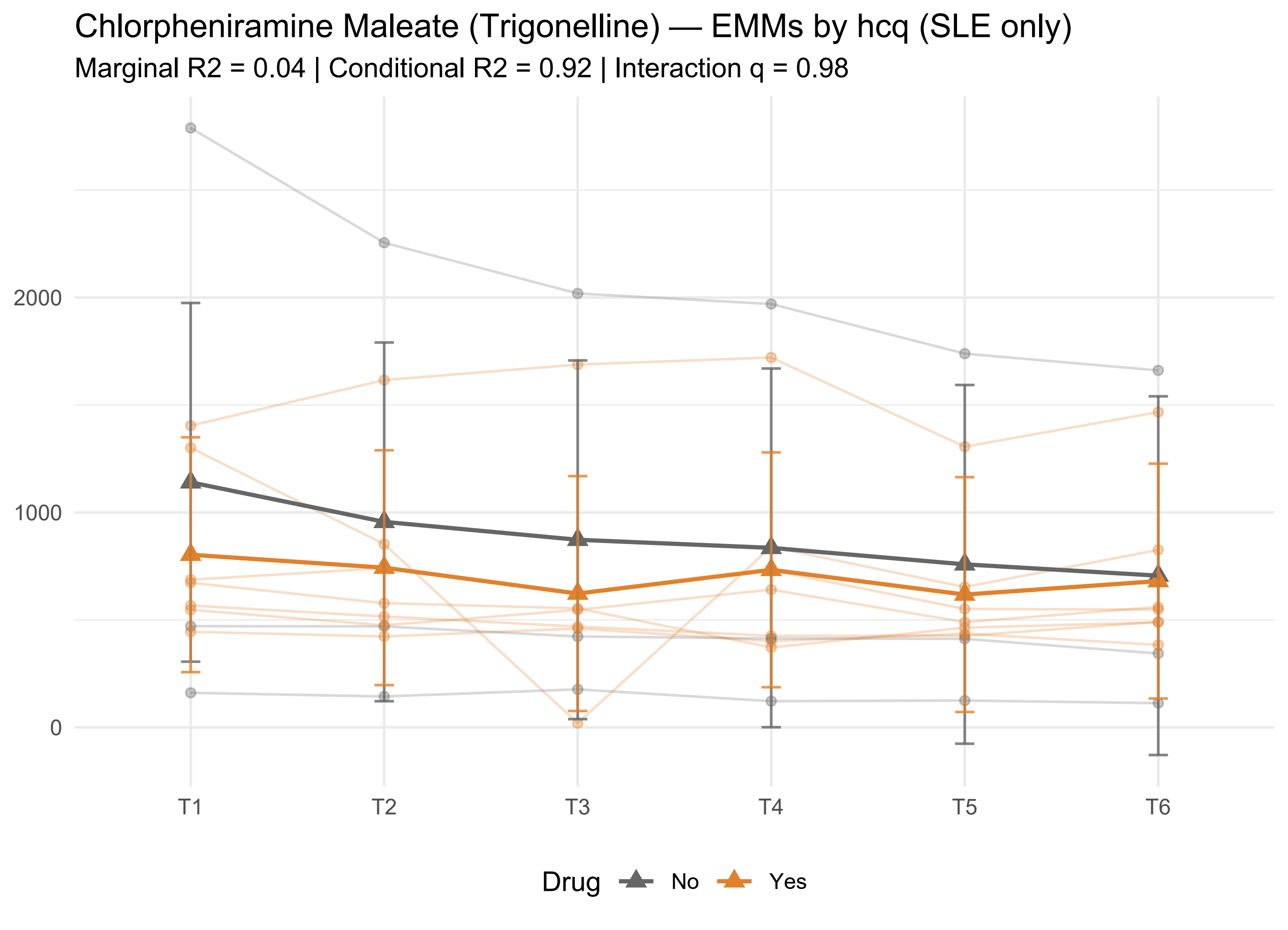
Chlorpheniramine Maleate (Trigonelline) — EMMs by hcq (SLE only)
Marginal R2 = 0.04 | Conditional R2 = 0.92 | Interaction q = 0.98
2000
1000
0
T1
T2
T3
T4
T5
T6
Drug
No
Yes

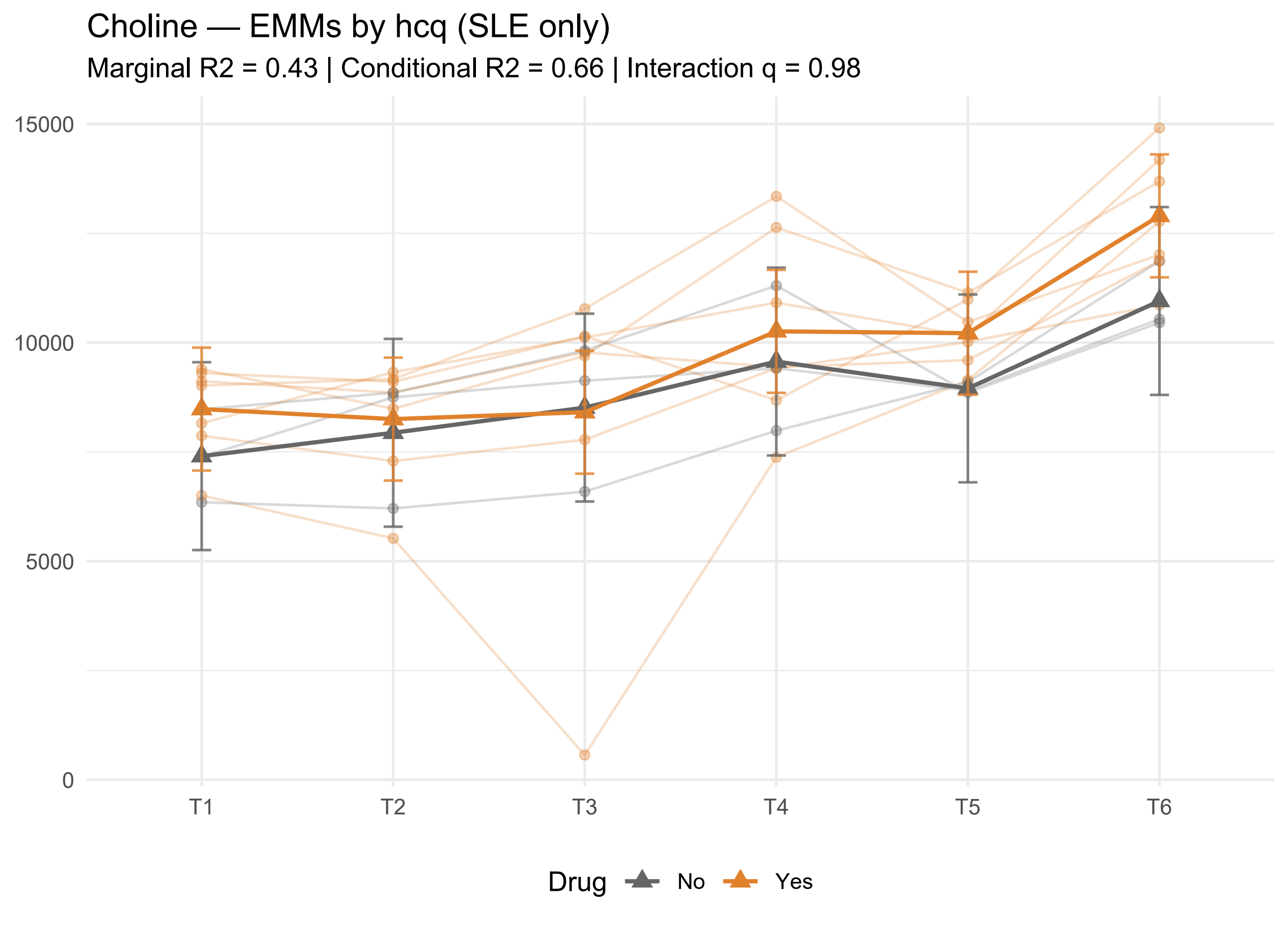
Choline — EMMs by hcq (SLE only)
Marginal R2 = 0.43 | Conditional R2 = 0.66 | Interaction q = 0.98
15000
10000
5000
0
T1
T2
T3
T4
T5
T6
Drug
No
Yes

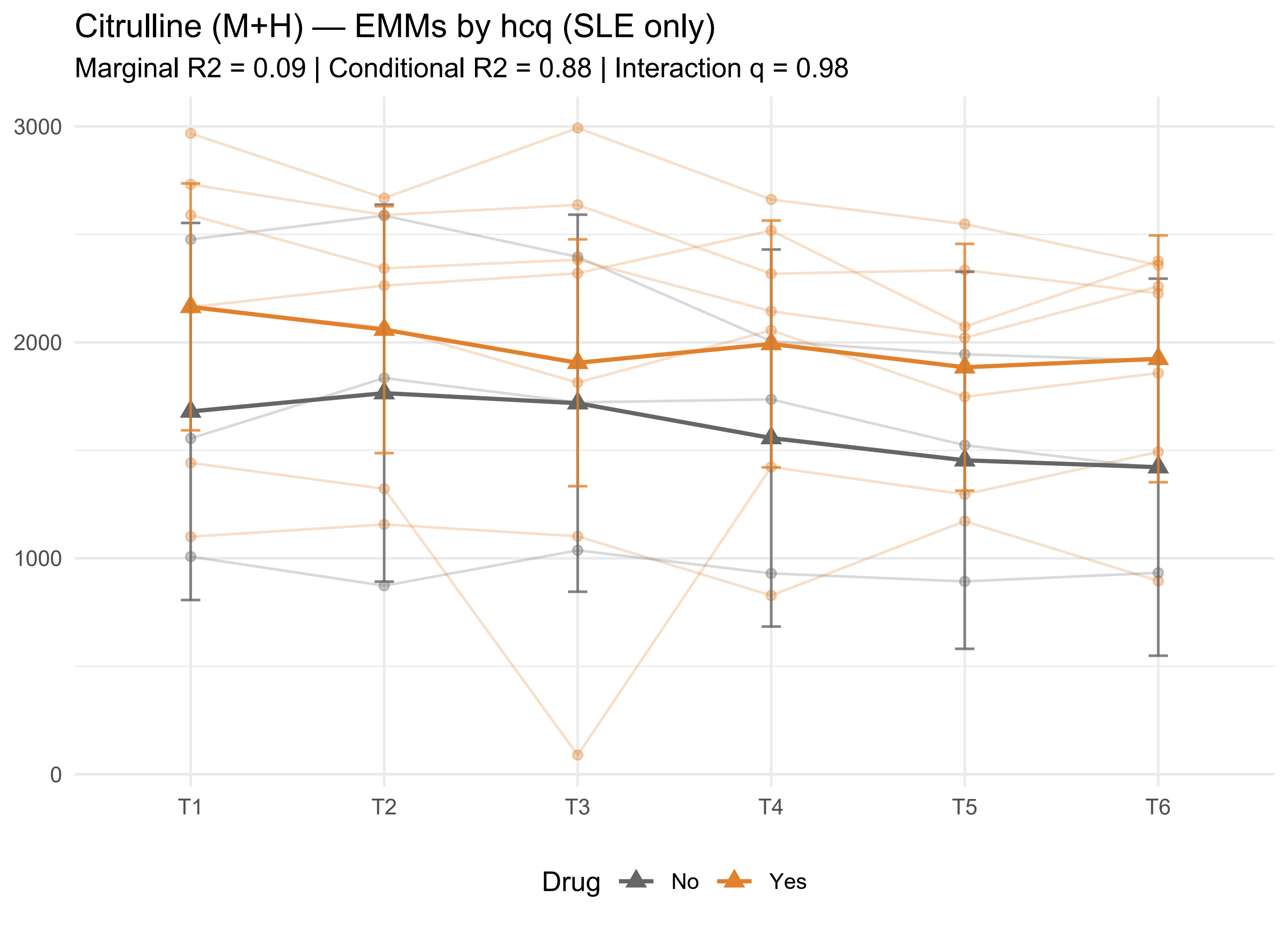

Citrulline (M+H) — EMMs by hcq (SLE only)
Marginal R2 = 0.09 | Conditional R2 = 0.88 | Interaction q = 0.98
3000
2000
1000
0
T1
T2
T3
T4
T5
T6
Drug
No
Yes

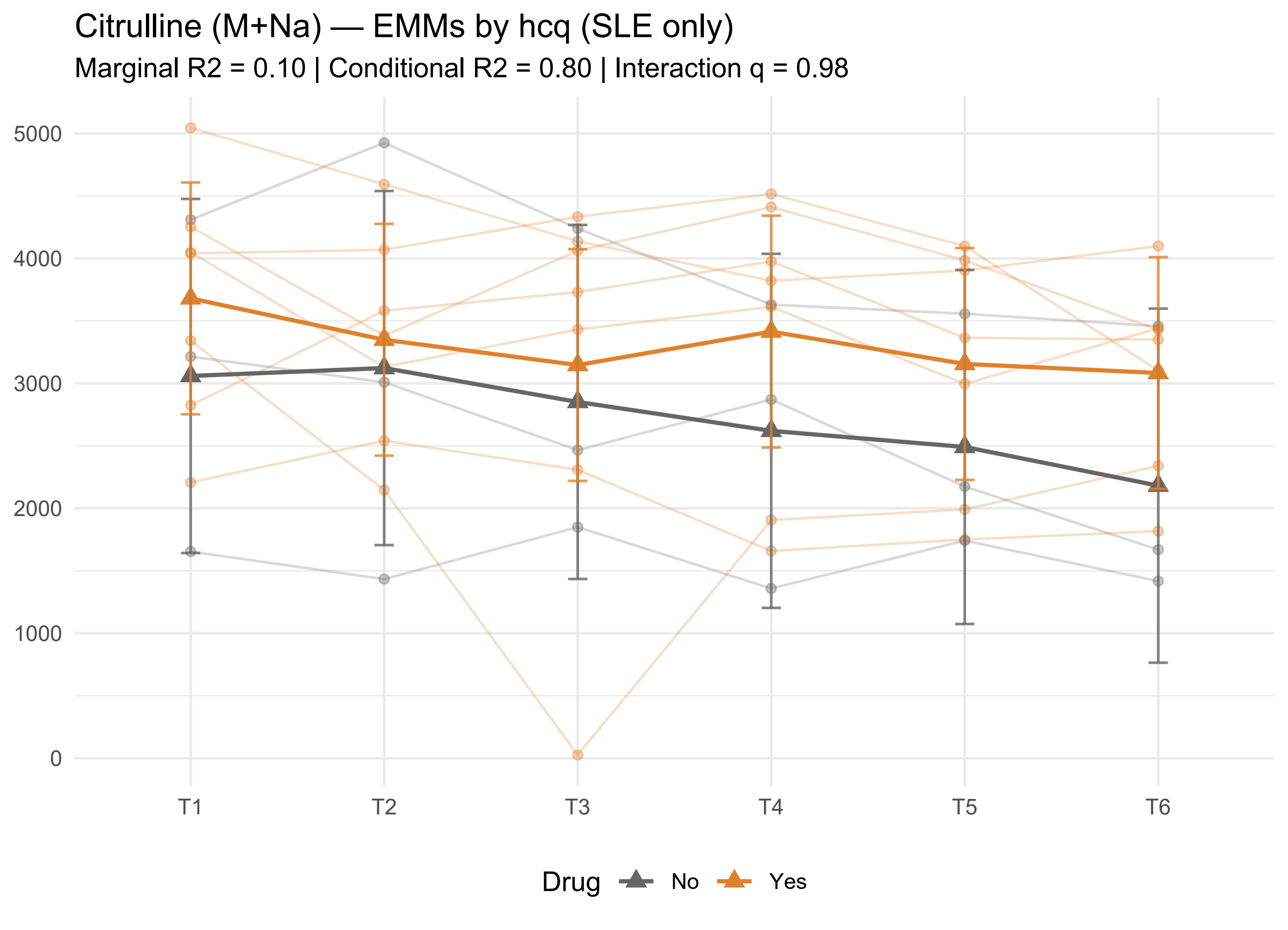

Citrulline (M+Na) — EMMs by hcq (SLE only)
Marginal R2 = 0.10 | Conditional R2 = 0.80 | Interaction q = 0.98
5000
4000
3000
2000
1000
0
T1
T2
T3
T4
T5
T6
Drug
No
Yes

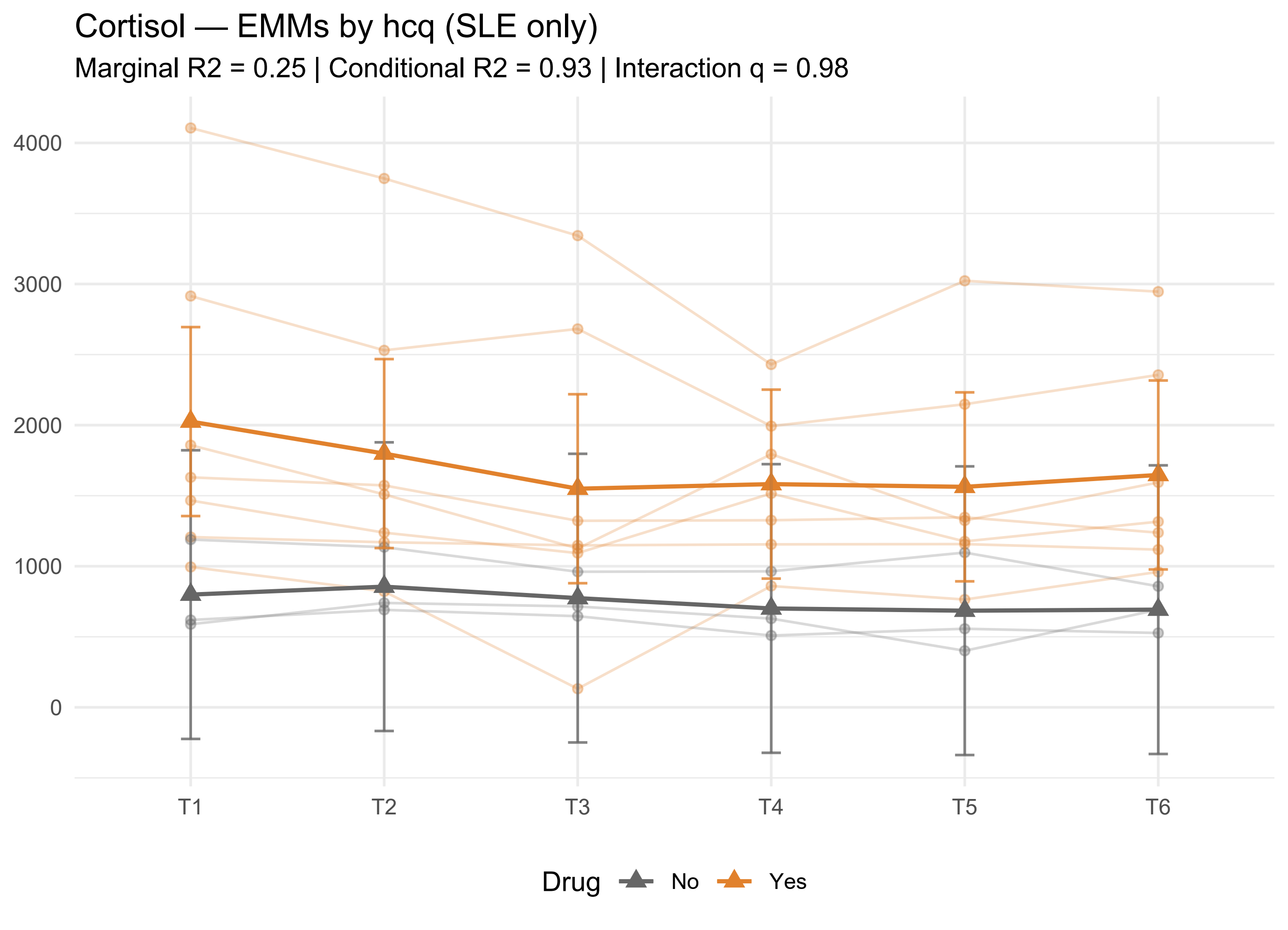

Cortisol — EMMs by hcq (SLE only)
Marginal R2 = 0.25 | Conditional R2 = 0.93 | Interaction q = 0.98
4000
3000
2000
1000
0
T1
T2
T3
T4
T5
T6
Drug
No
Yes

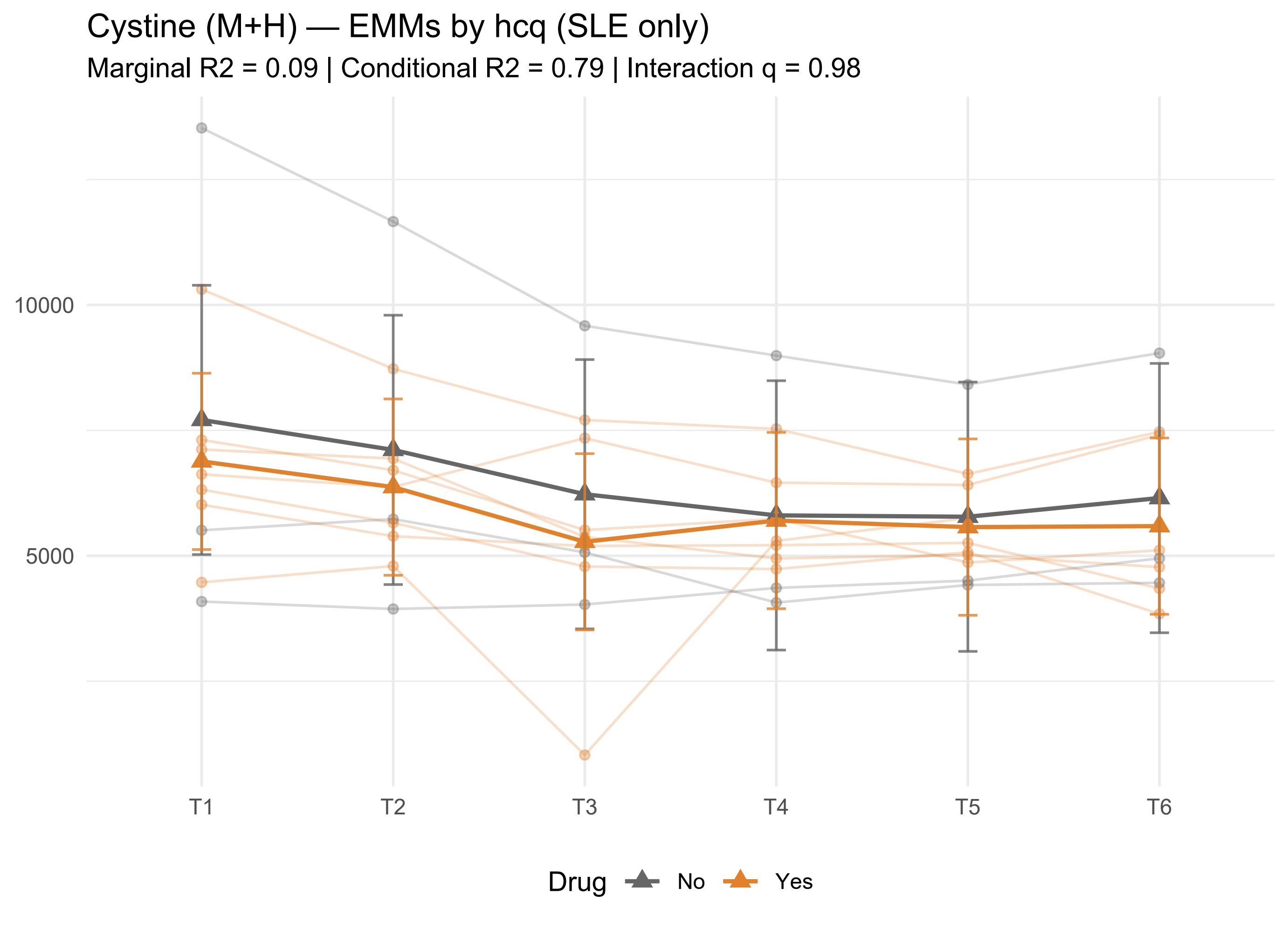

Cystine (M+H) — EMMs by hcq (SLE only)
Marginal R2 = 0.09 | Conditional R2 = 0.79 | Interaction q = 0.98
10000
5000
T1
T2
T3
T4
T5
T6
Drug
No
Yes

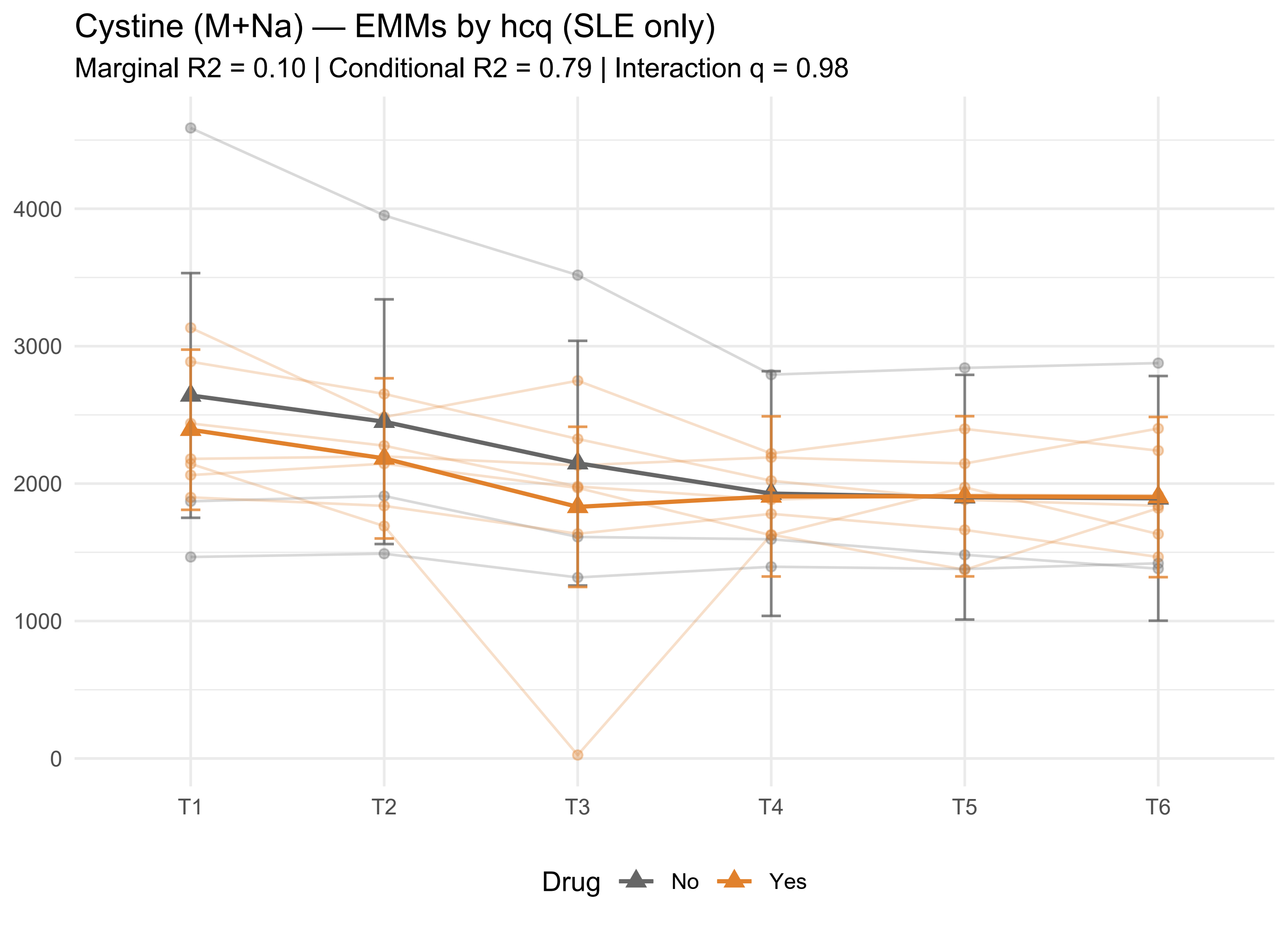

Cystine (M+Na) — EMMs by hcq (SLE only)
Marginal R2 = 0.10 | Conditional R2 = 0.79 | Interaction q = 0.98
4000
3000
2000
1000
0
T1
T2
T3
T4
T5
T6
Drug
No
Yes

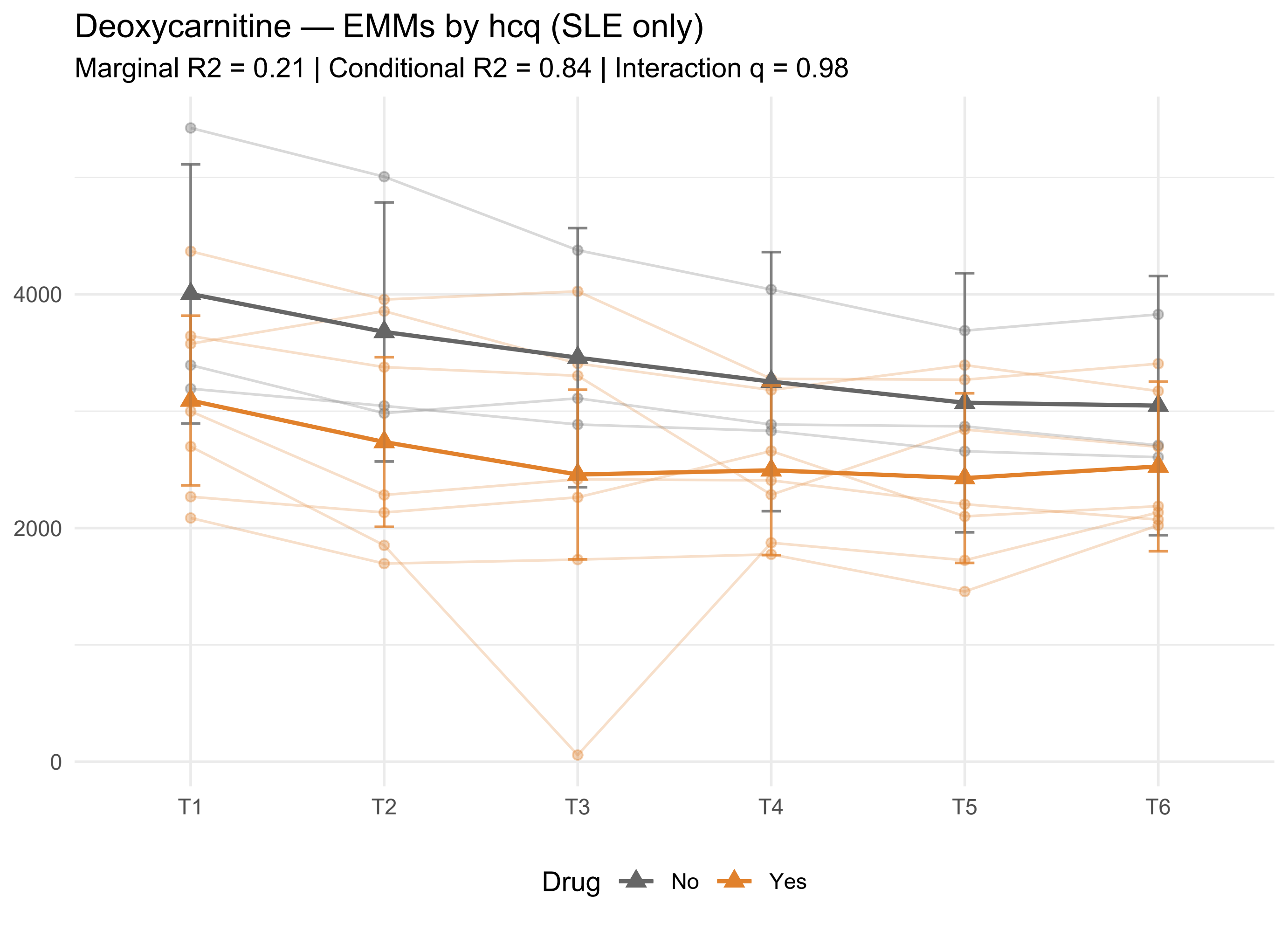
Deoxycarnitine — EMMs by hcq (SLE only)
Marginal R2 = 0.21 | Conditional R2 = 0.84 | Interaction q = 0.98
4000
2000
0
T1
T2
T3
T4
T5
T6
Drug
No
Yes

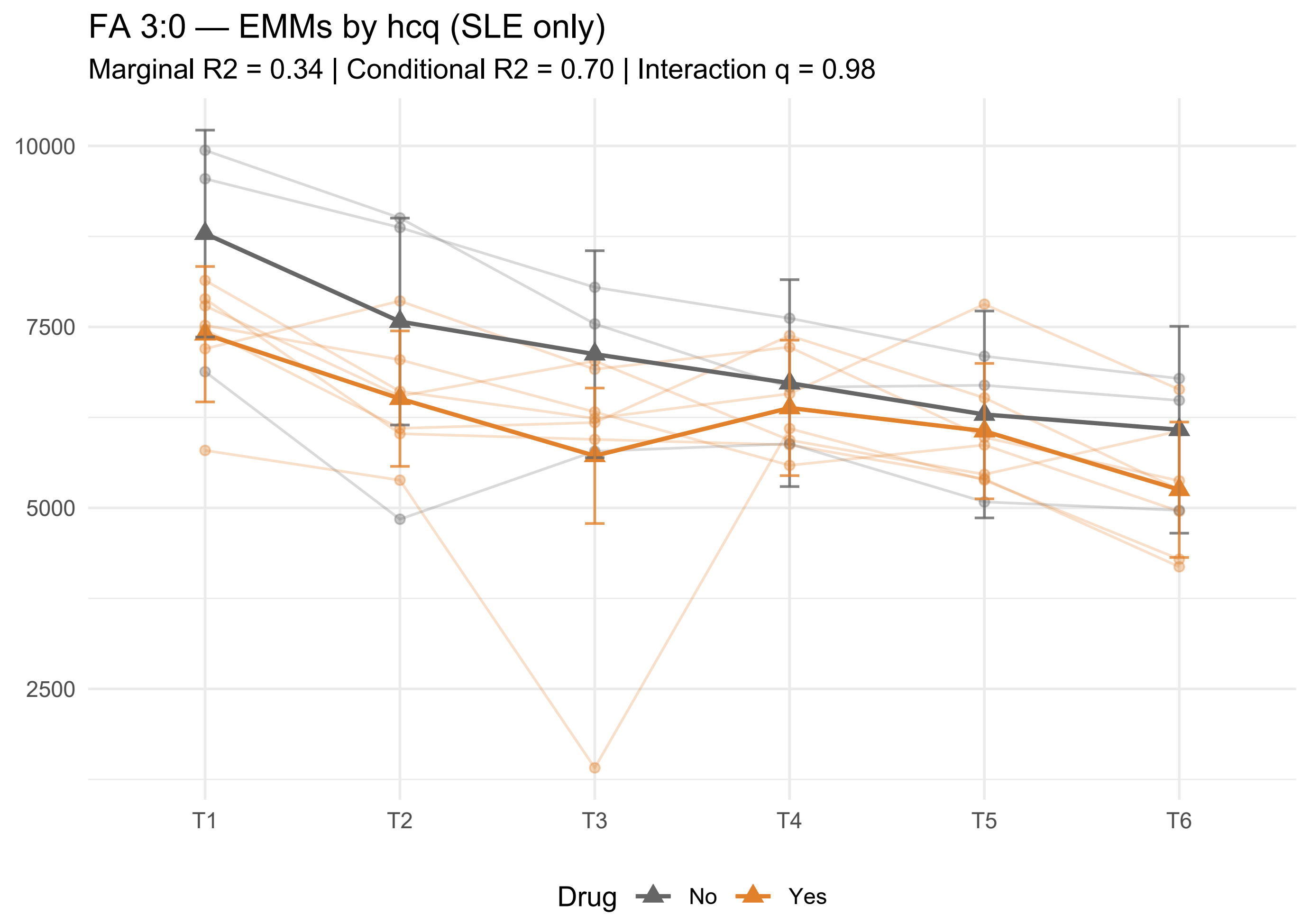

FA 3:0 — EMMs by hcq (SLE only)
Marginal R2 = 0.34 | Conditional R2 = 0.70 | Interaction q = 0.98
10000
7500
5000
2500
T1
T2
T3
T4
T5
T6
Drug
No
Yes

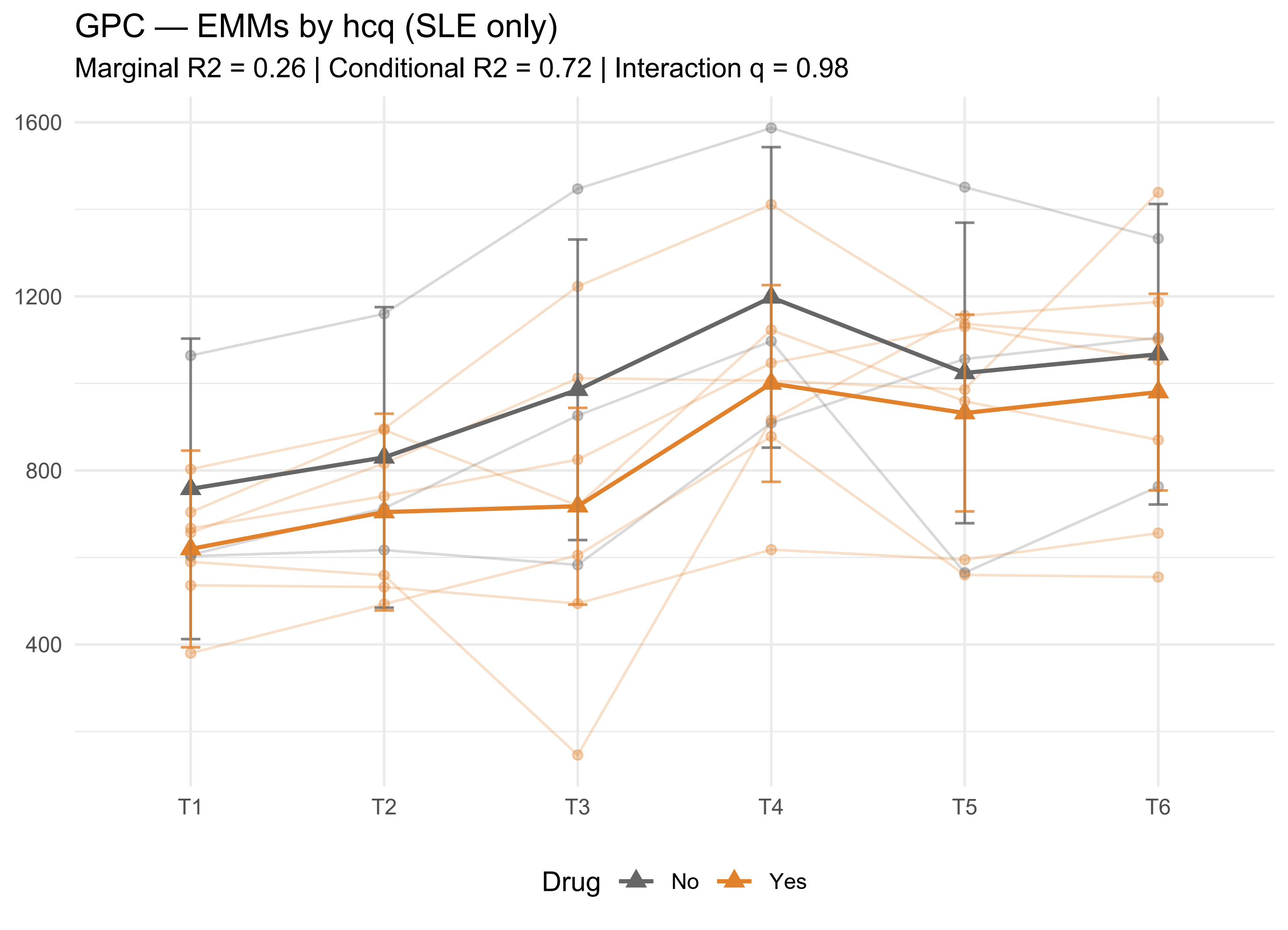
GPC — EMMs by hcq (SLE only)
Marginal R2 = 0.26 | Conditional R2 = 0.72 | Interaction q = 0.98
1600
1200
800
400
T1
T2
T3
T4
T5
T6
Drug
No
Yes

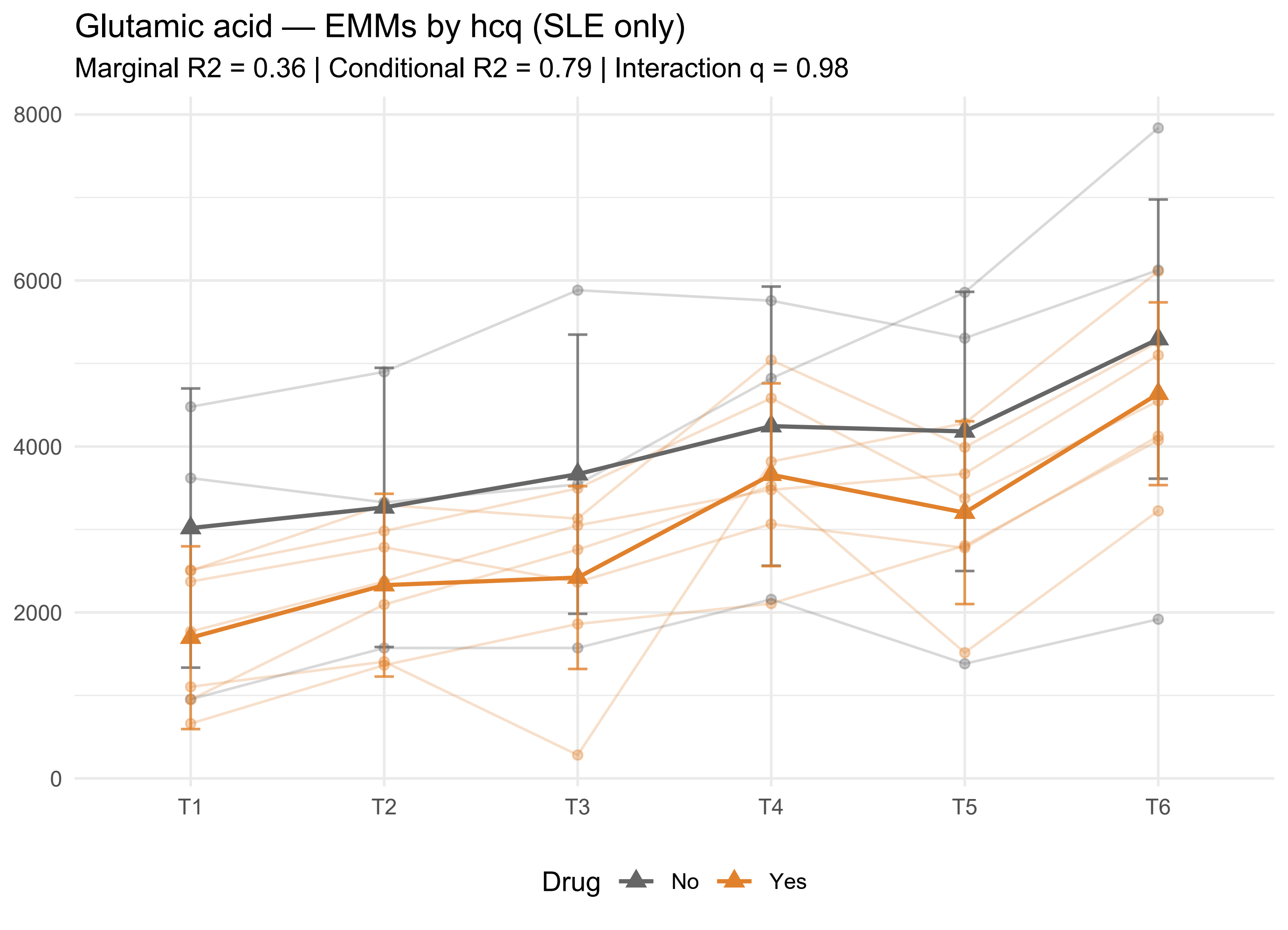

Glutamic acid — EMMs by hcq (SLE only)
Marginal R2 = 0.36 | Conditional R2 = 0.79 | Interaction q = 0.98
8000
6000
4000
2000
0
T1
T2
T3
T4
T5
T6
Drug
No
Yes

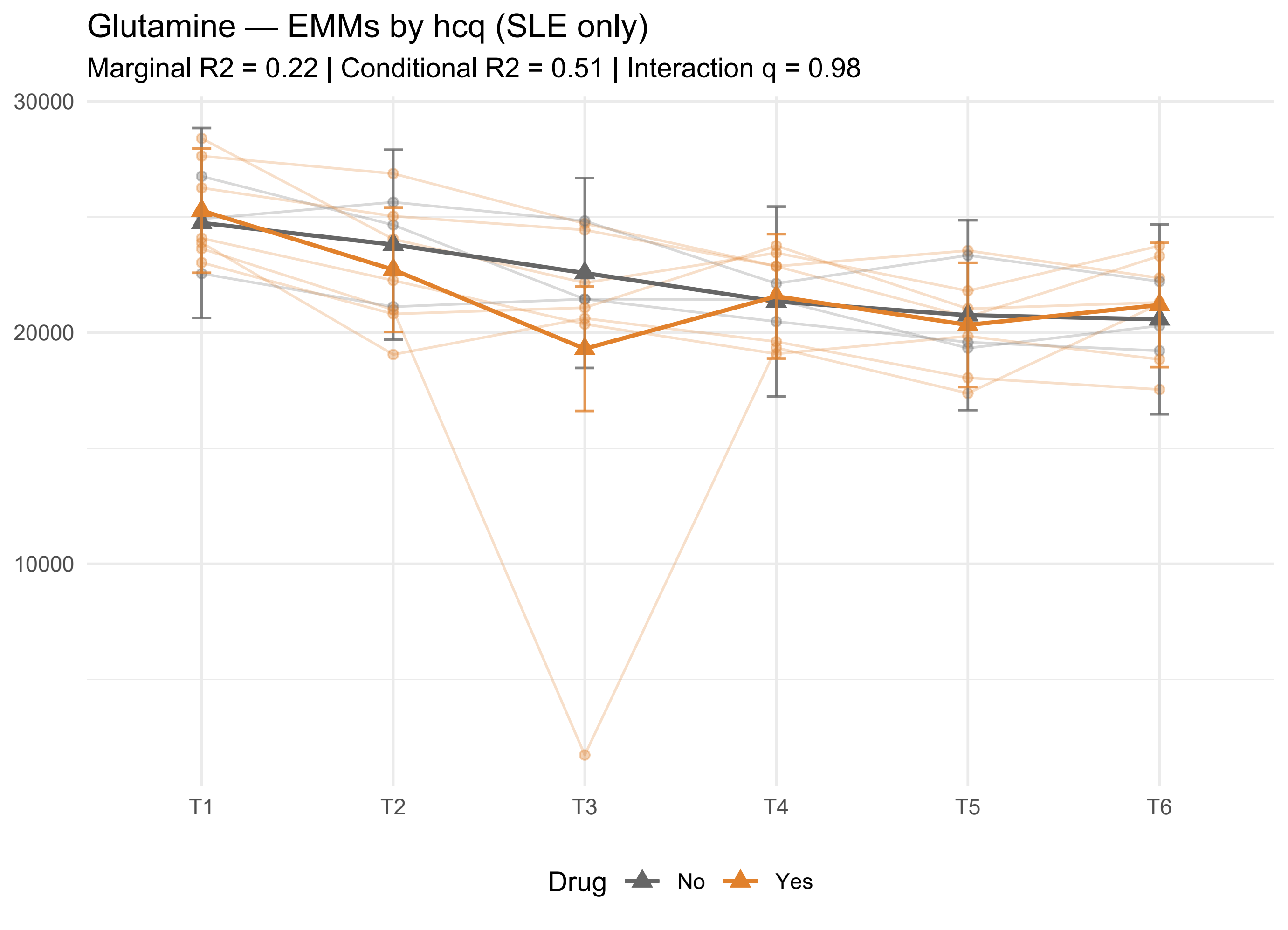
Glutamine — EMMs by hcq (SLE only)
Marginal R2 = 0.22 | Conditional R2 = 0.51 | Interaction q = 0.98
30000
20000
10000
T1
T2
T3
T4
T5
T6
Drug
No
Yes

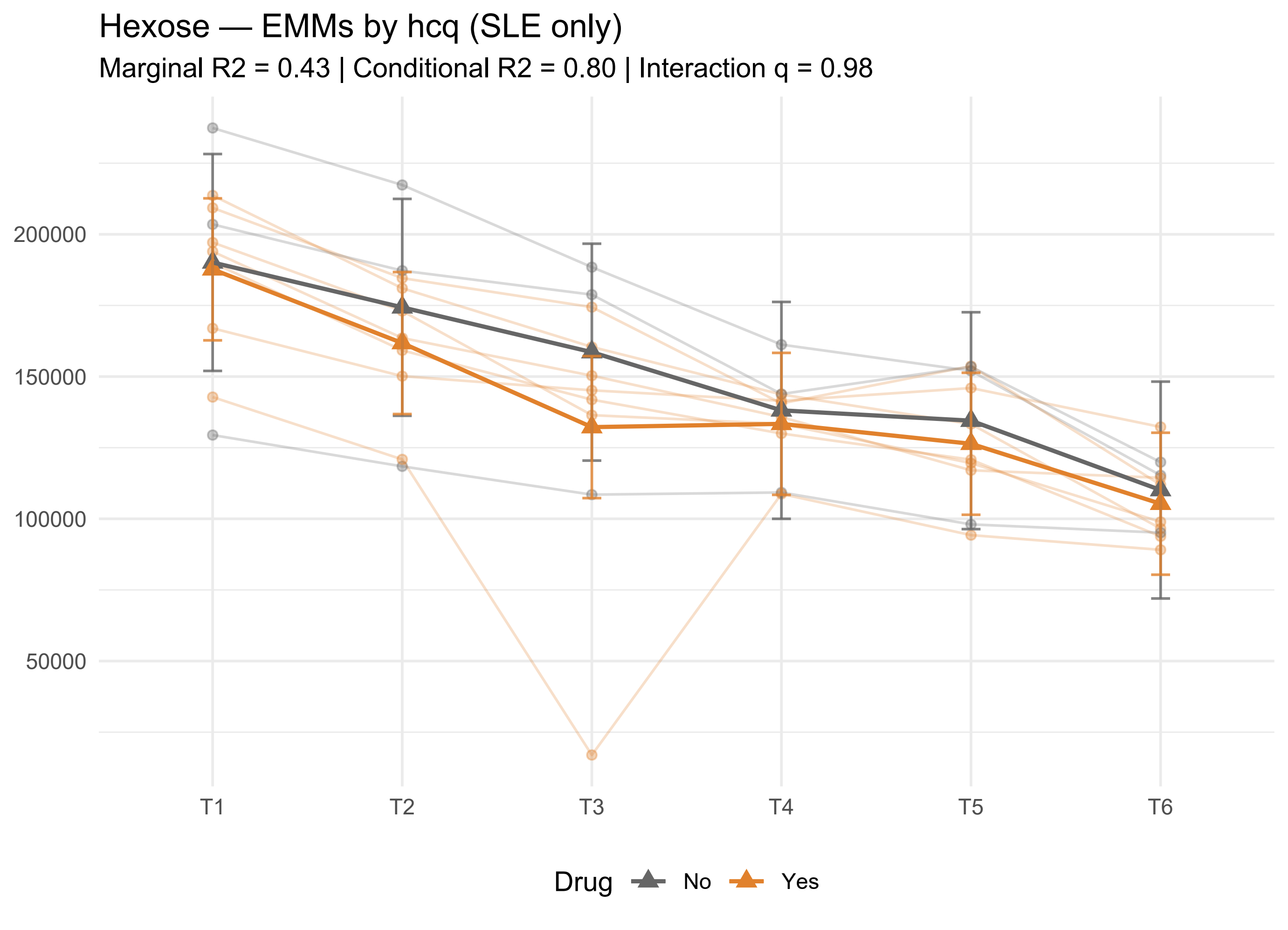

Hexose — EMMs by hcq (SLE only)
Marginal R2 = 0.43 | Conditional R2 = 0.80 | Interaction q = 0.98
200000
150000
100000
50000
T1
T2
T3
T4
T5
T6
Drug
No
Yes

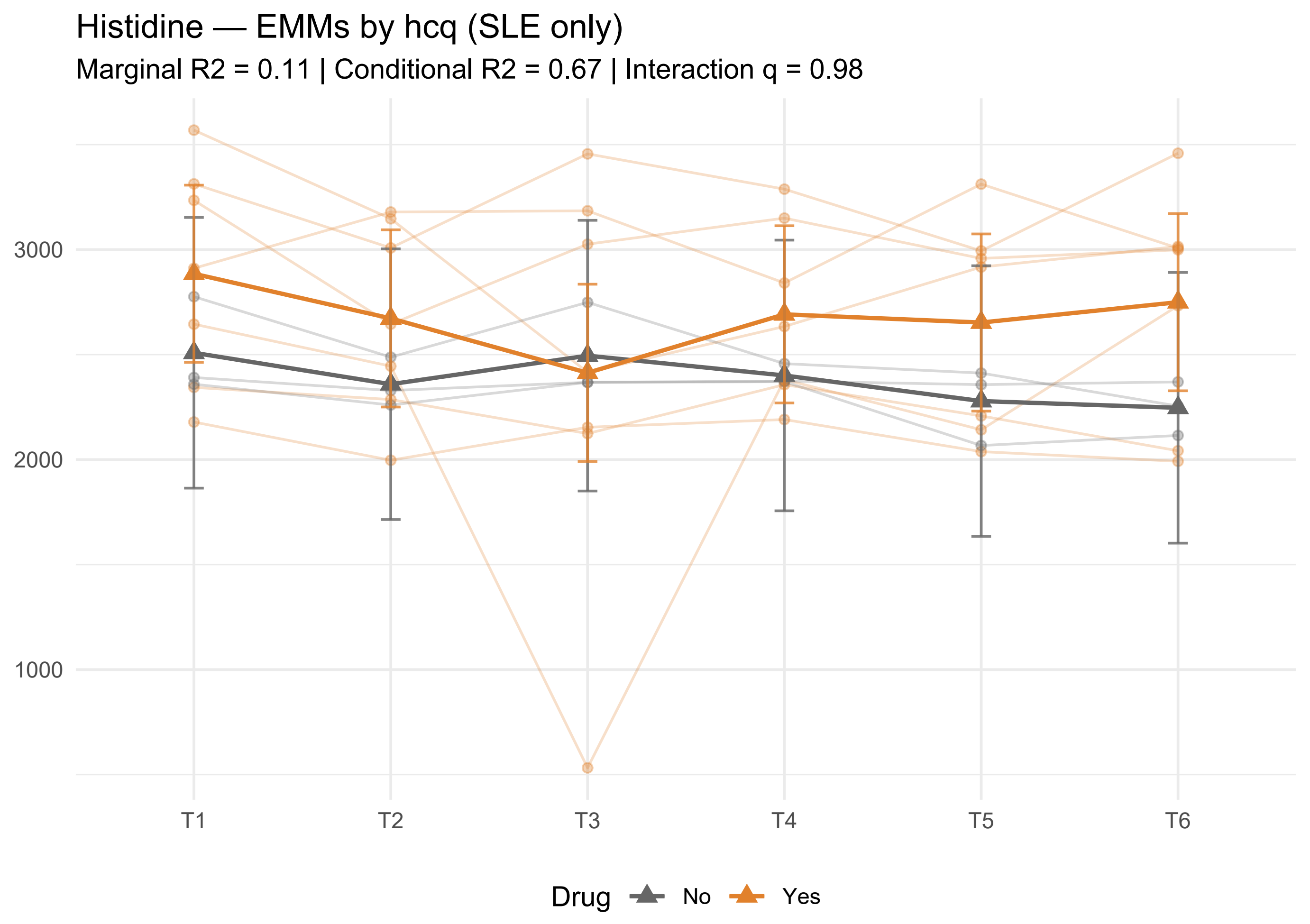

Histidine — EMMs by hcq (SLE only)
Marginal R2 = 0.11 | Conditional R2 = 0.67 | Interaction q = 0.98
3000
2000
1000
T1
T2
T3
T4
T5
T6
Drug
No
Yes

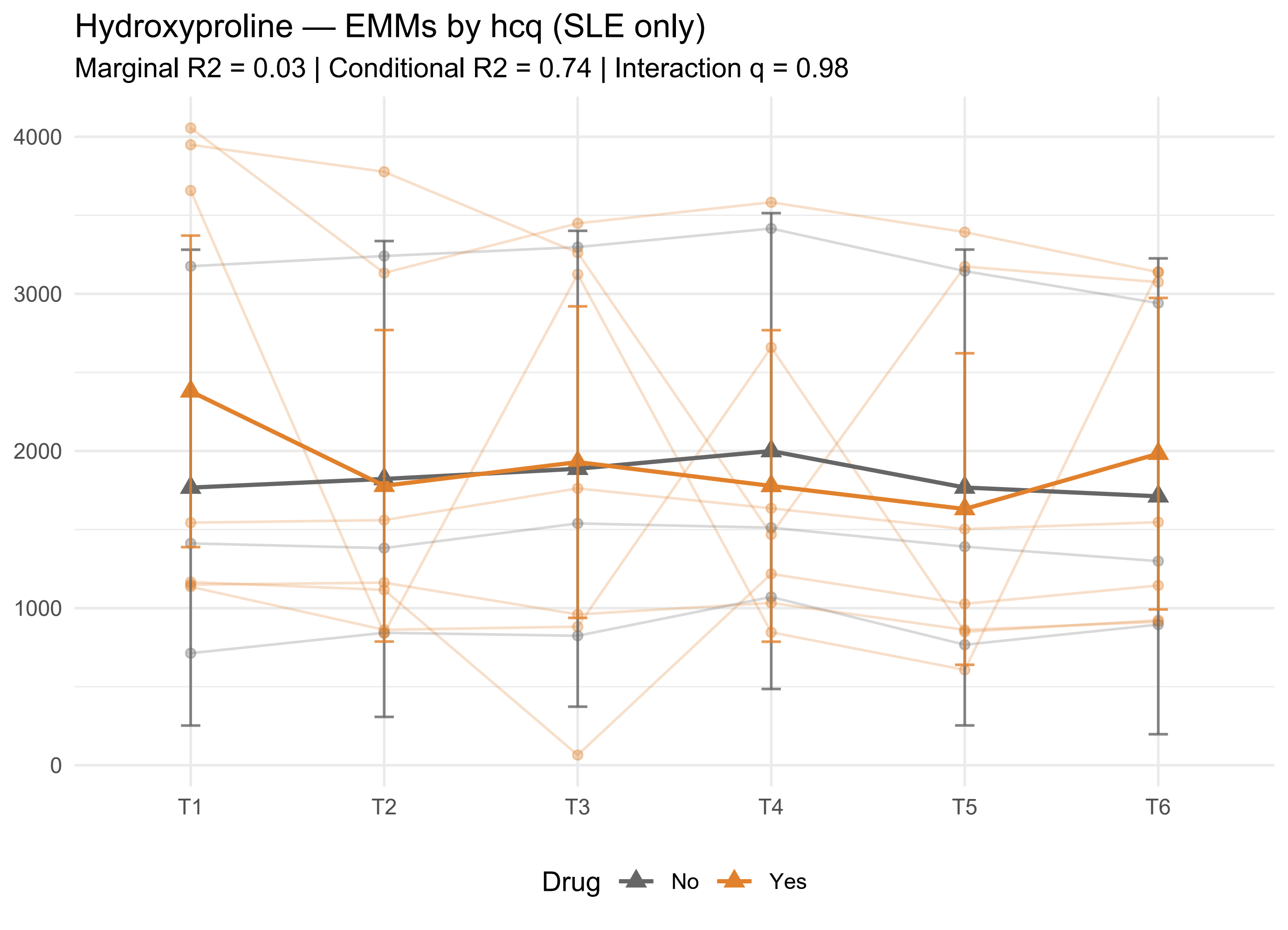

Hydroxyproline — EMMs by hcq (SLE only)
Marginal R2 = 0.03 | Conditional R2 = 0.74 | Interaction q = 0.98
4000
3000
2000
1000
0
T1
T2
T3
T4
T5
T6
Drug
No
Yes

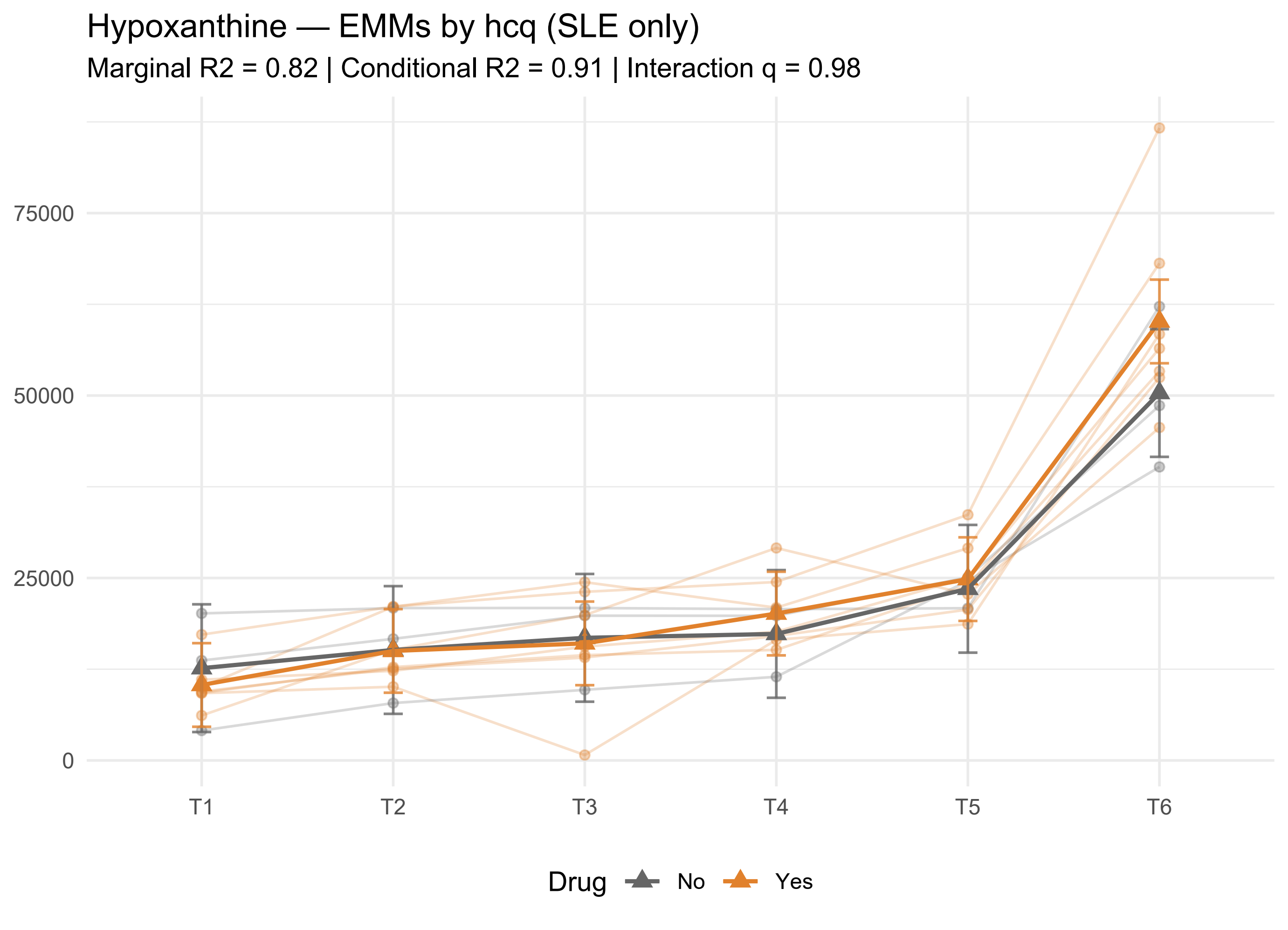

Hypoxanthine — EMMs by hcq (SLE only)
Marginal R2 = 0.82 | Conditional R2 = 0.91 | Interaction q = 0.98
75000
50000
25000
0
T1
T2
T3
T4
T5
T6
Drug
No
Yes

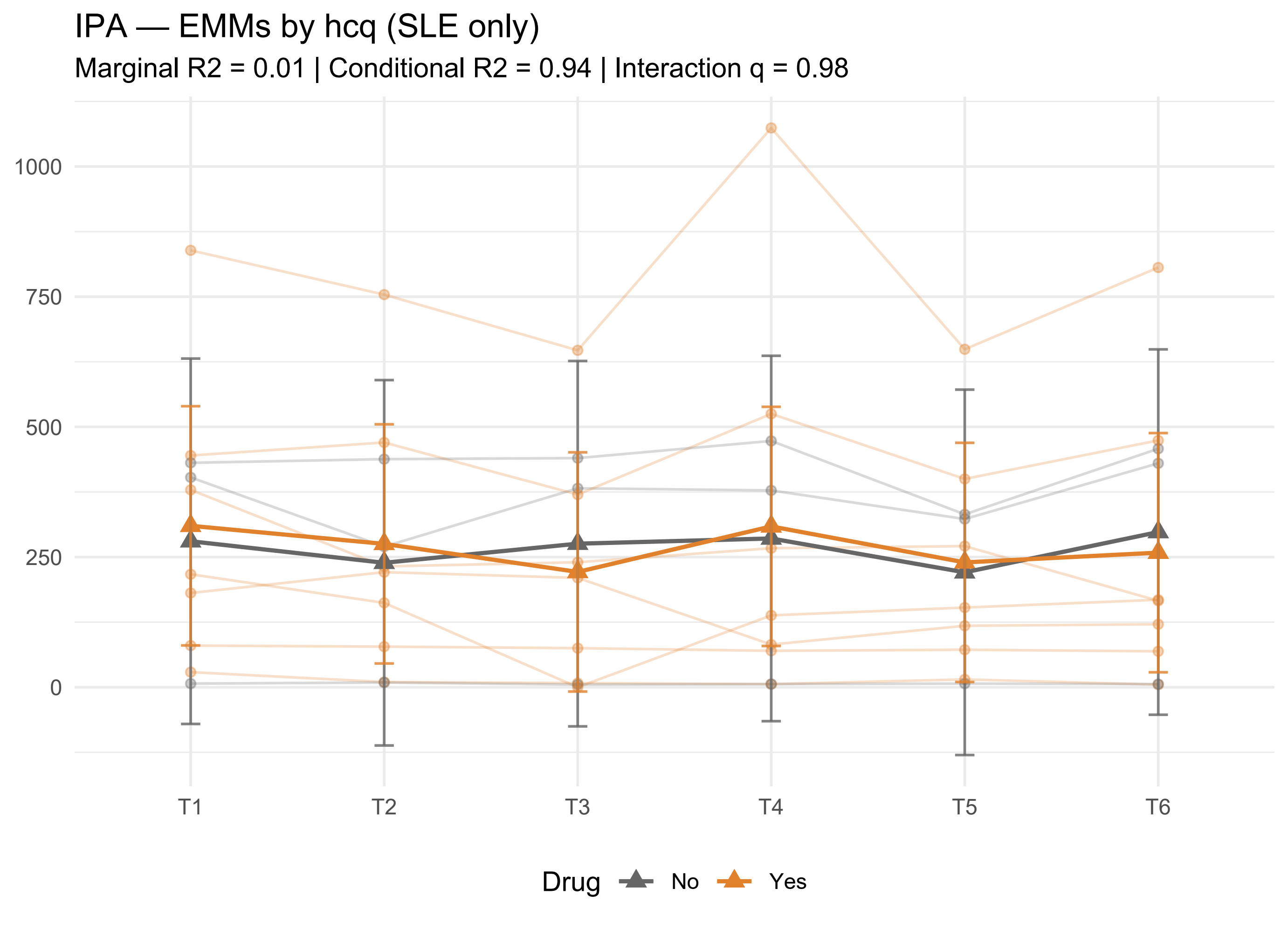
IPA — EMMs by hcq (SLE only)
Marginal R2 = 0.01 | Conditional R2 = 0.94 | Interaction q = 0.98
1000
750
500
250
0
T1
T2
T3
T4
T5
T6
Drug
No
Yes

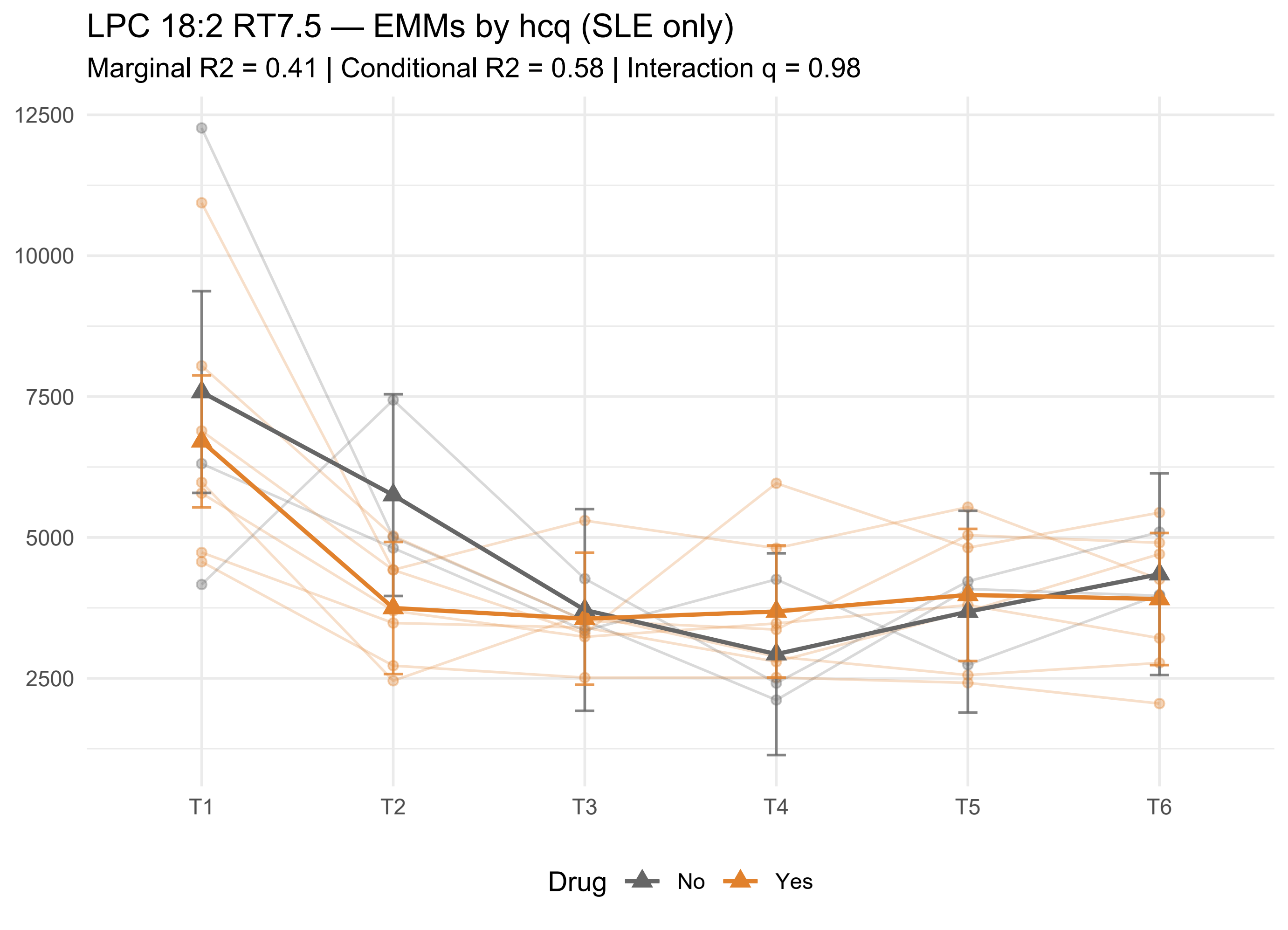

LPC 18:2 RT7.5 — EMMs by hcq (SLE only)
Marginal R2 = 0.41 | Conditional R2 = 0.58 | Interaction q = 0.98
12500
10000
7500
5000
2500
T1
T2
T3
T4
T5
T6
Drug
No
Yes

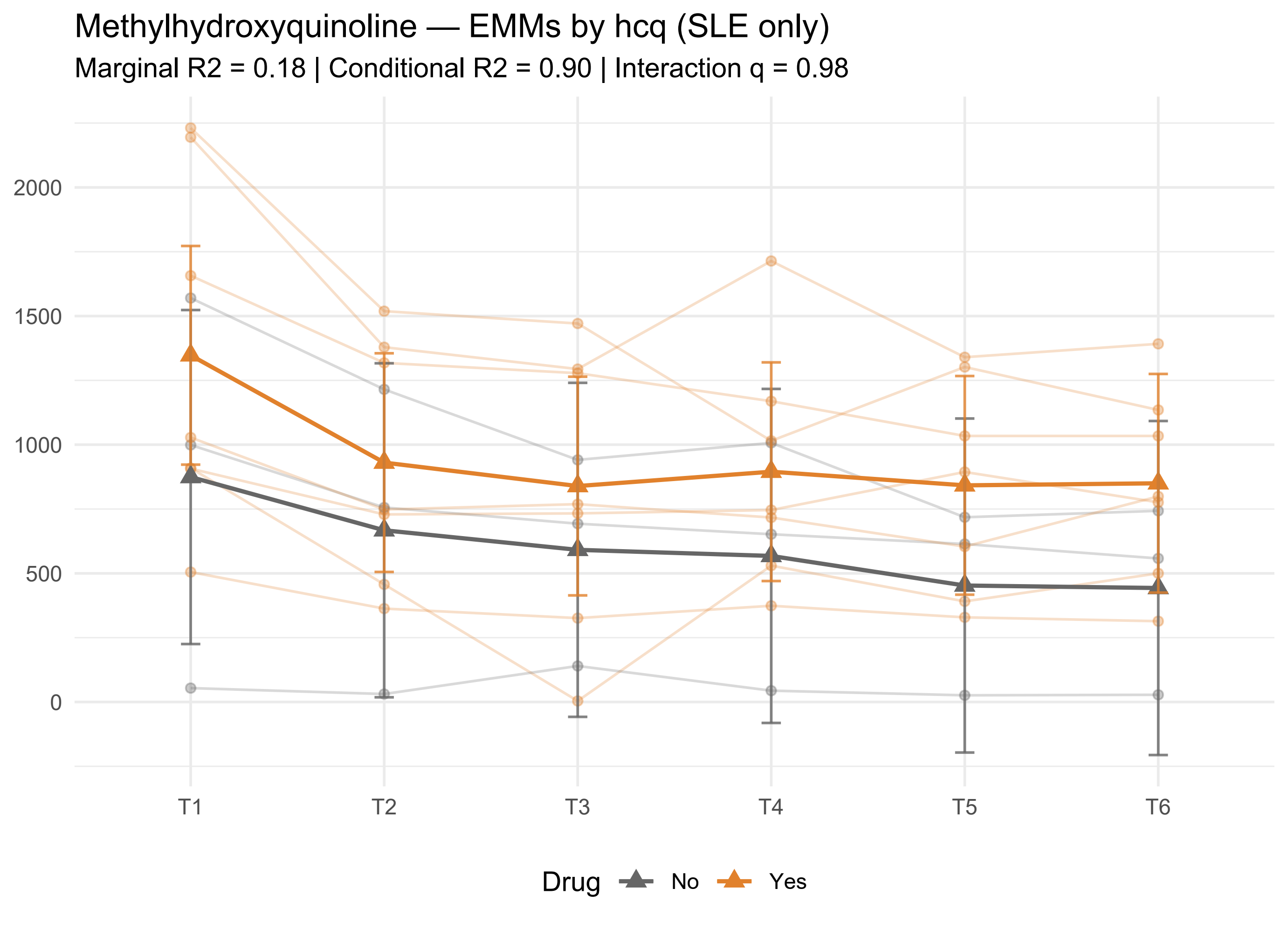

Methylhydroxyquinoline — EMMs by hcq (SLE only)
Marginal R2 = 0.18 | Conditional R2 = 0.90 | Interaction q = 0.98
2000
1500
1000
500
0
T1
T2
T3
T4
T5
T6
Drug
No
Yes

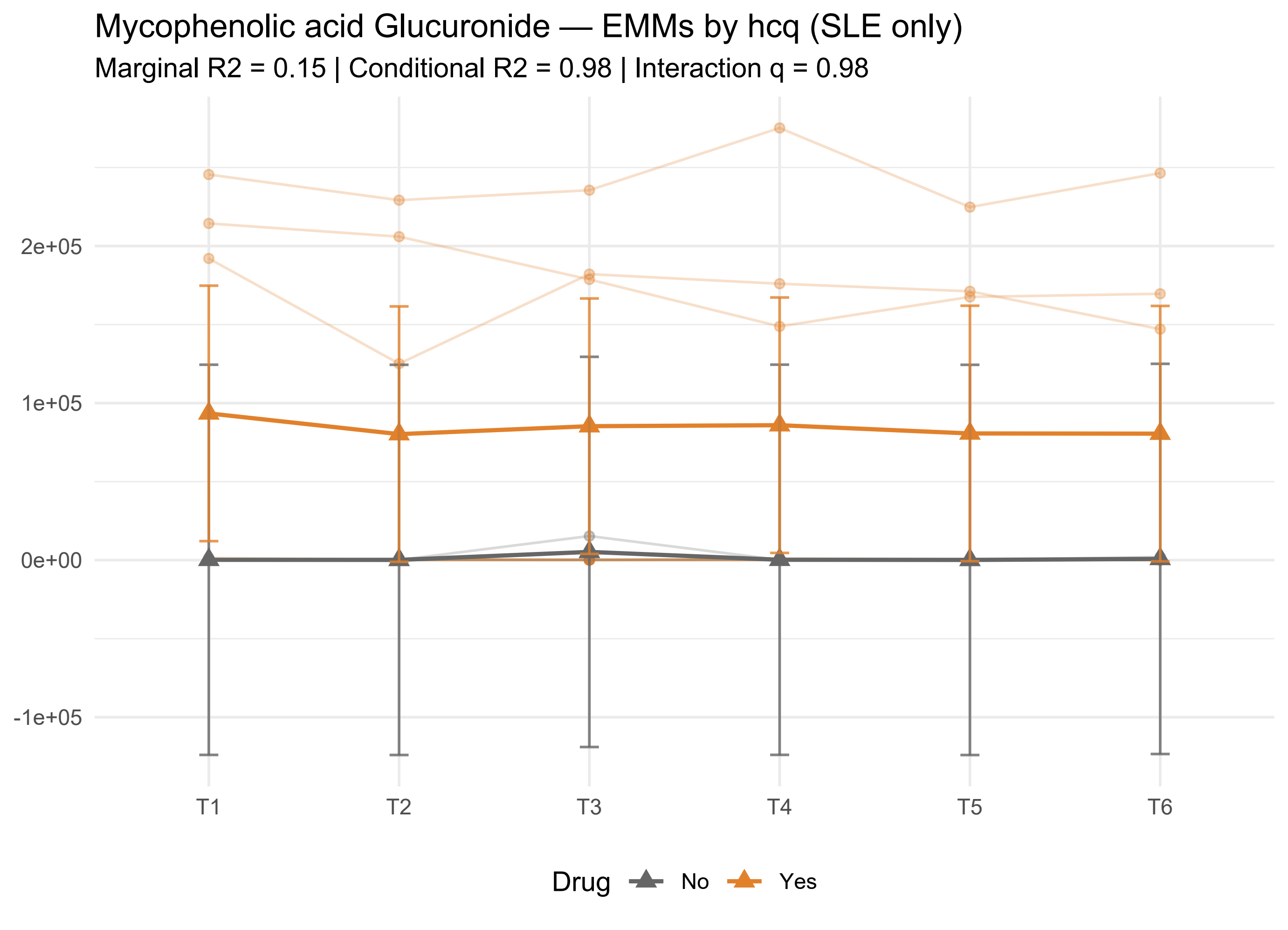

Mycophenolic acid Glucuronide — EMMs by hcq (SLE only)
Marginal R2 = 0.15 | Conditional R2 = 0.98 | Interaction q = 0.98
2e+05
1e+05
0e+00
-1e+05
T1
T2
T3
T4
T5
T6
Drug
No
Yes

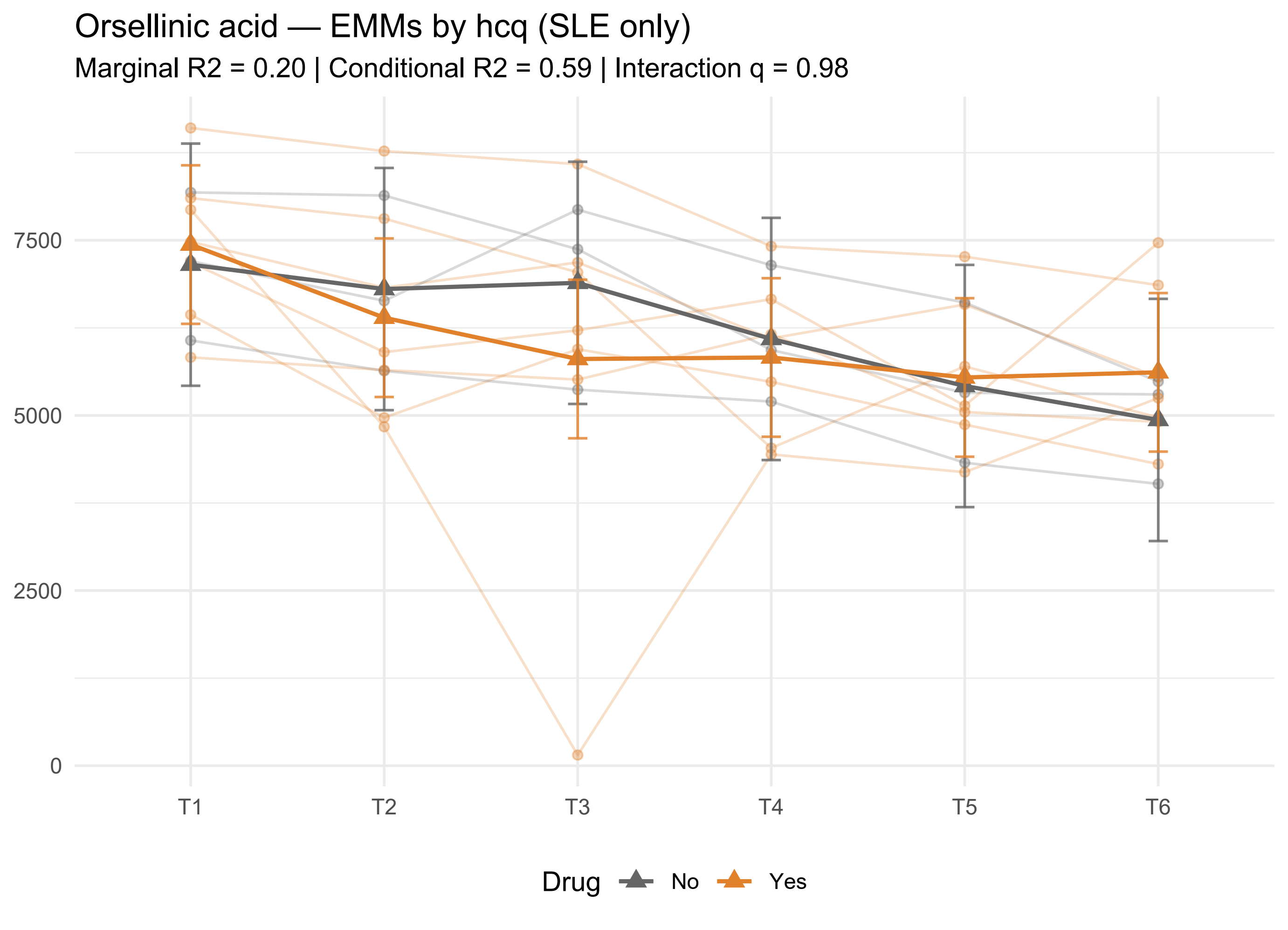

Orsellinic acid — EMMs by hcq (SLE only)
Marginal R2 = 0.20 | Conditional R2 = 0.59 | Interaction q = 0.98
7500
5000
2500
0
T1
T2
T3
T4
T5
T6
Drug
No
Yes

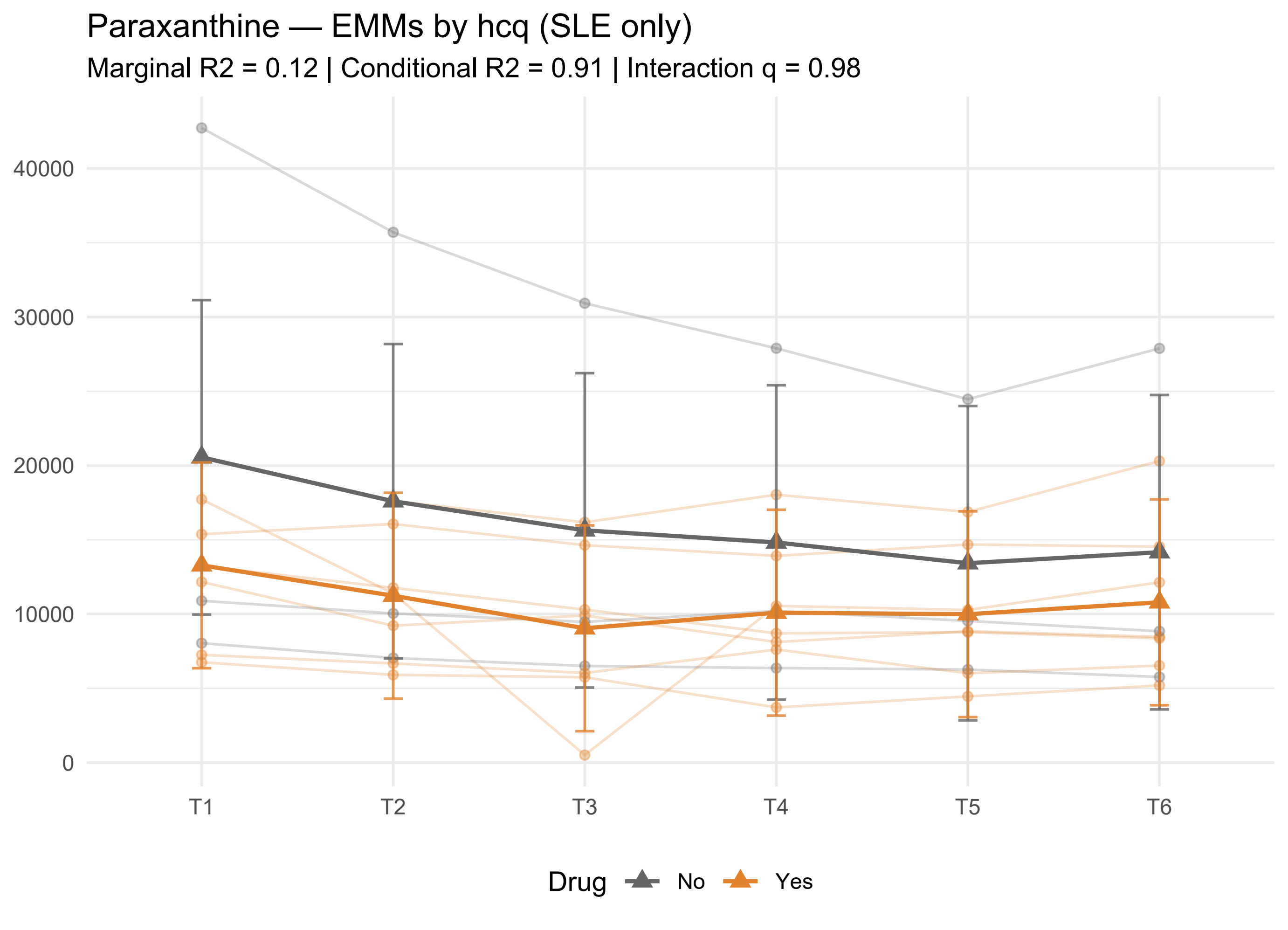

Paraxanthine — EMMs by hcq (SLE only)
Marginal R2 = 0.12 | Conditional R2 = 0.91 | Interaction q = 0.98
40000
30000
20000
10000
0
T1
T2
T3
T4
T5
T6
Drug
No
Yes

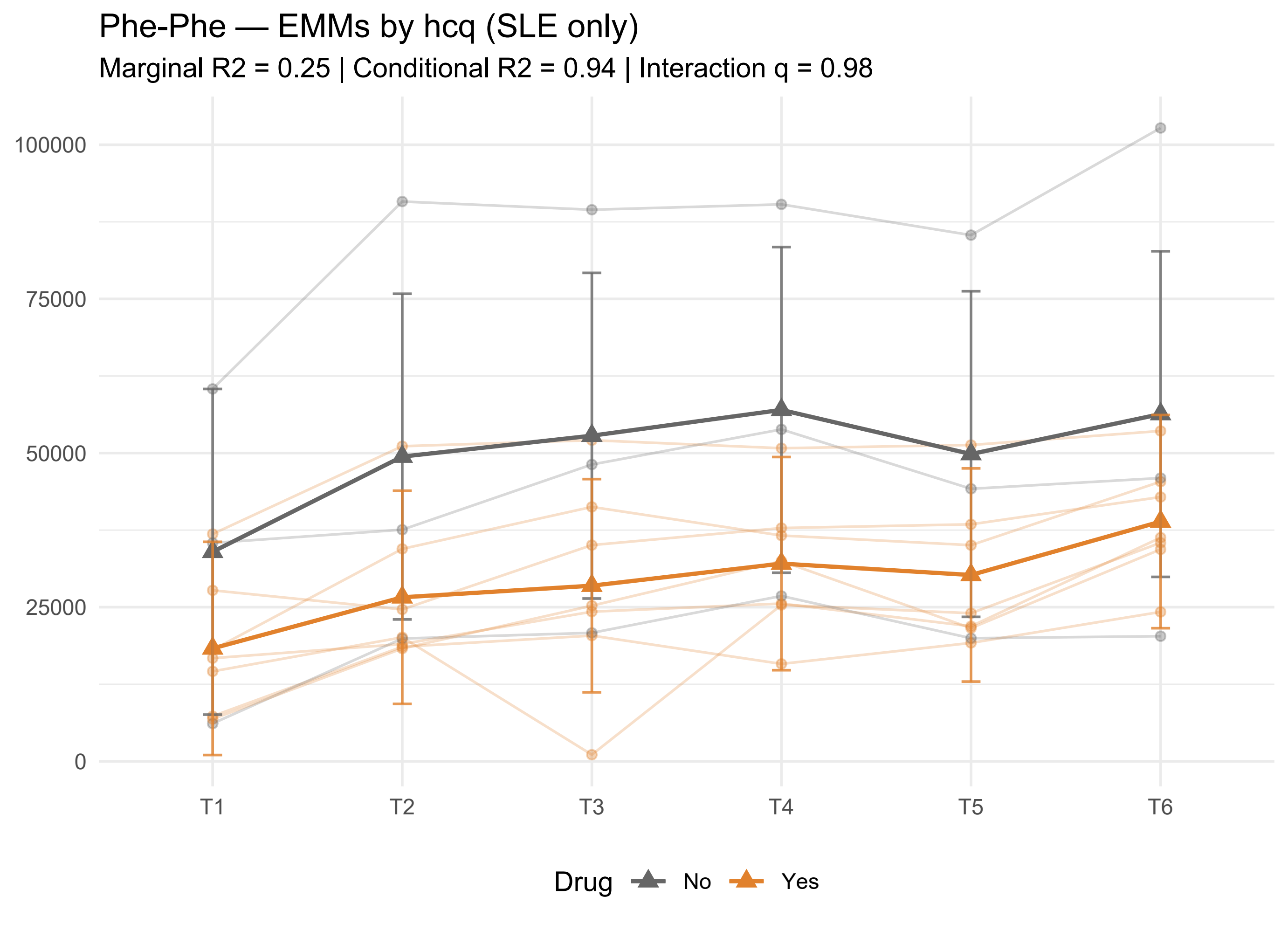

Phe-Phe — EMMs by hcq (SLE only)
Marginal R2 = 0.25 | Conditional R2 = 0.94 | Interaction q = 0.98
100000
75000
50000
25000
0
T1
T2
T3
T4
T5
T6
Drug
No
Yes

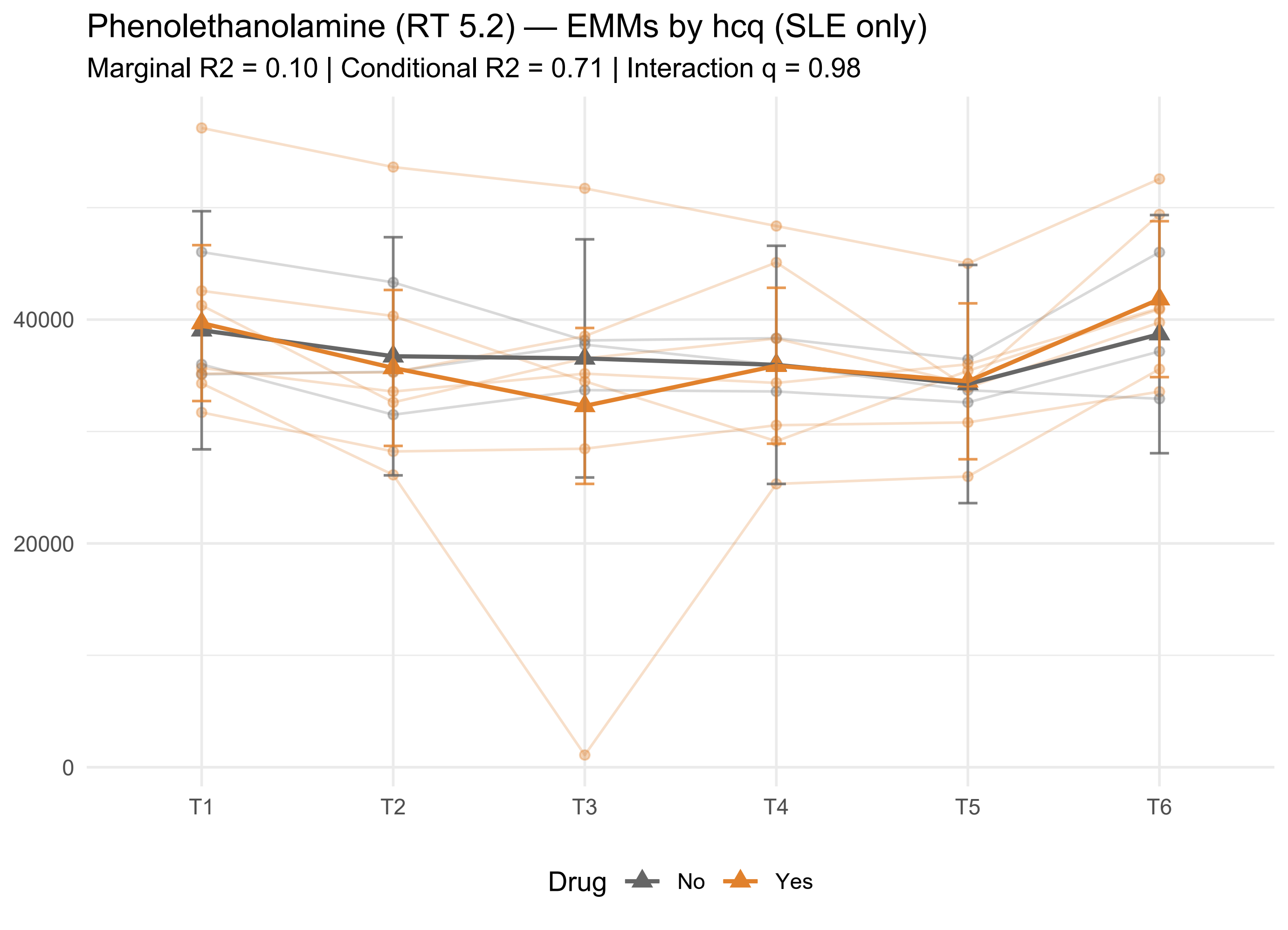

Phenolethanolamine (RT 5.2) — EMMs by hcq (SLE only)
Marginal R2 = 0.10 | Conditional R2 = 0.71 | Interaction q = 0.98
40000
20000
0
T1
T2
T3
T4
T5
T6
Drug
No
Yes

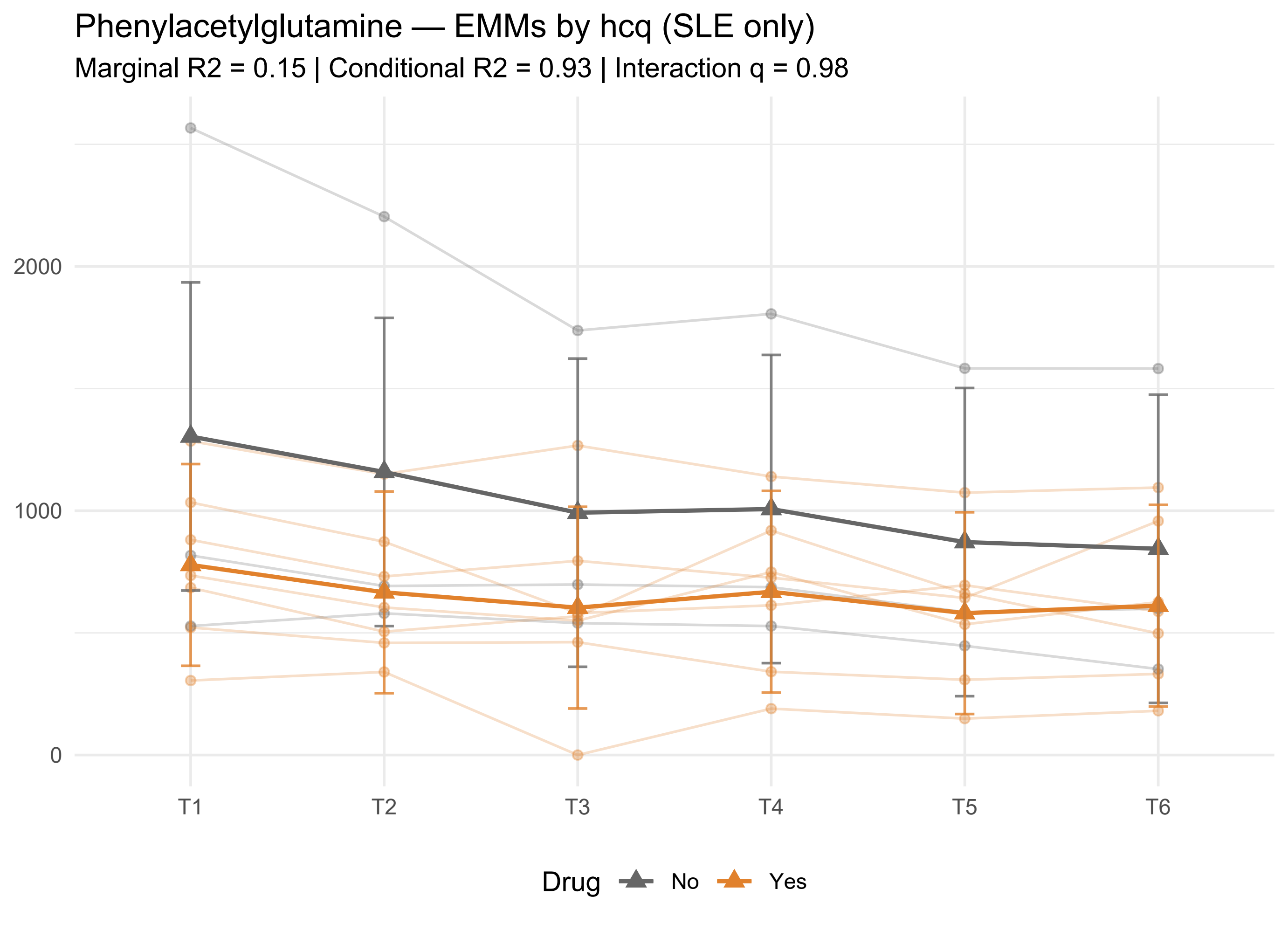

Phenylacetylglutamine — EMMs by hcq (SLE only)
Marginal R2 = 0.15 | Conditional R2 = 0.93 | Interaction q = 0.98
2000
1000
0
T1
T2
T3
T4
T5
T6
Drug
No
Yes

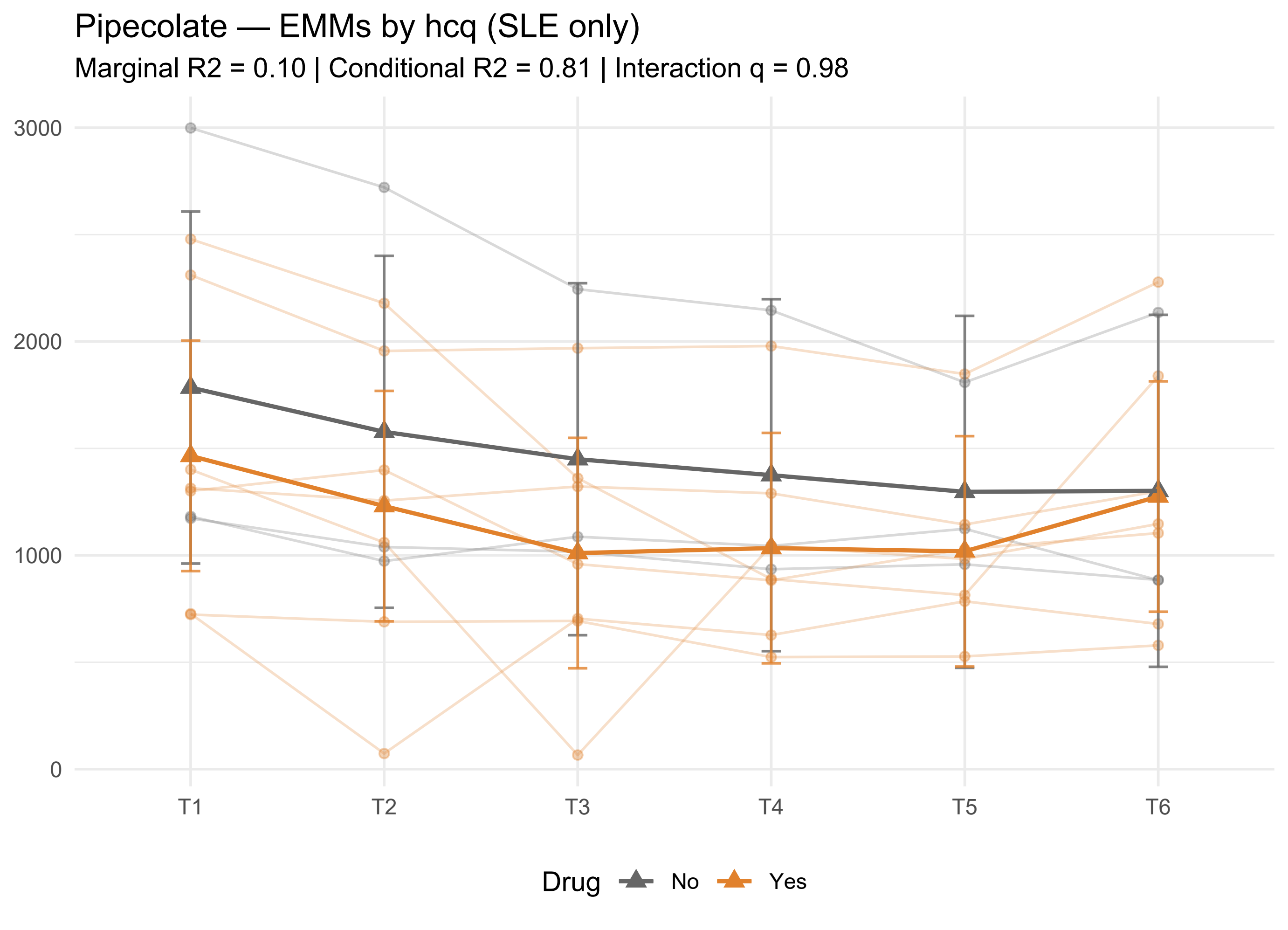

Pipecolate — EMMs by hcq (SLE only)
Marginal R2 = 0.10 | Conditional R2 = 0.81 | Interaction q = 0.98
3000
2000
1000
0
T1
T2
T3
T4
T5
T6
Drug
No
Yes

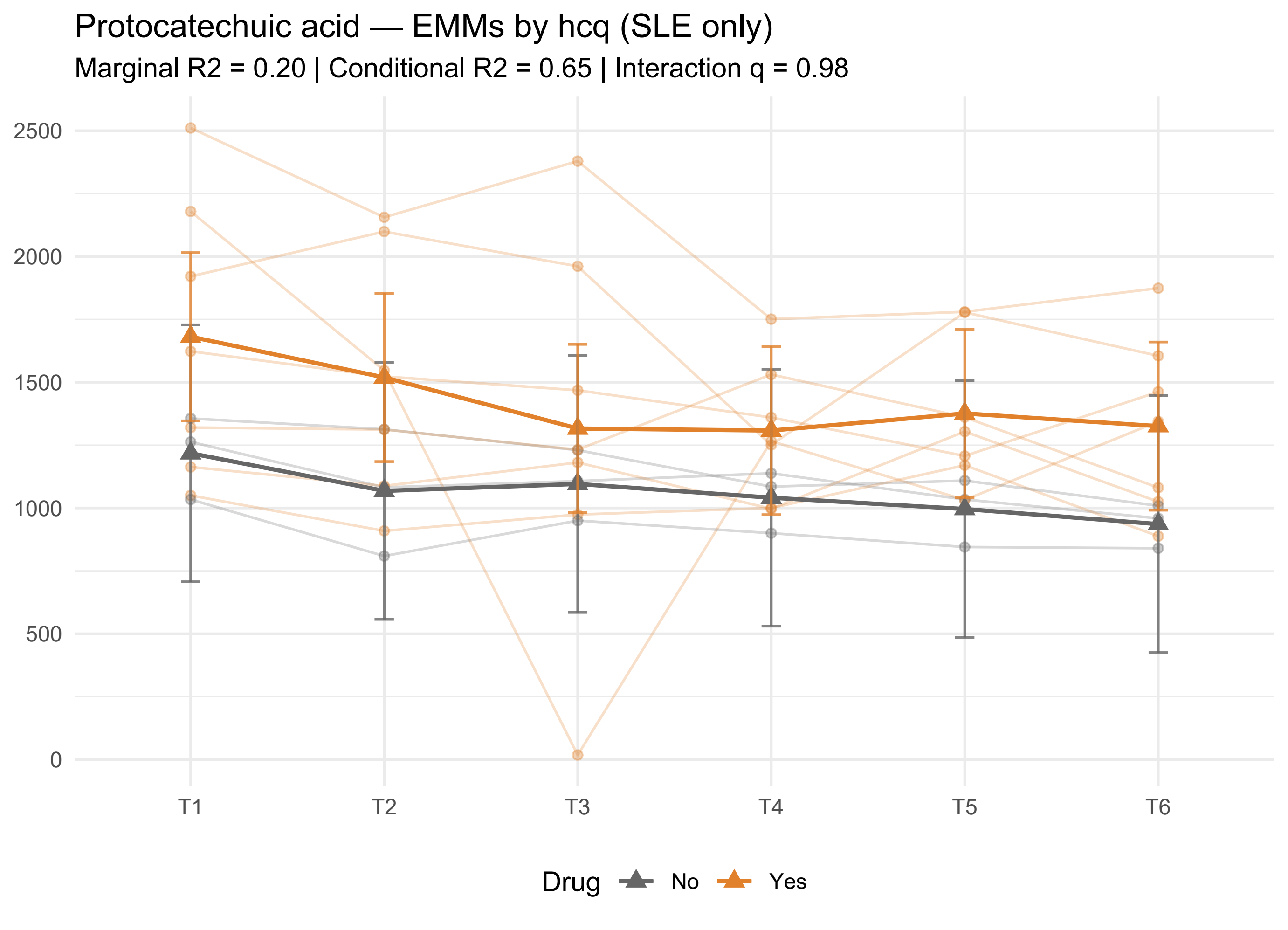

Protocatechuic acid — EMMs by hcq (SLE only)
Marginal R2 = 0.20 | Conditional R2 = 0.65 | Interaction q = 0.98
2500
2000
1500
1000
500
0
T1
T2
T3
T4
T5
T6
Drug
No
Yes

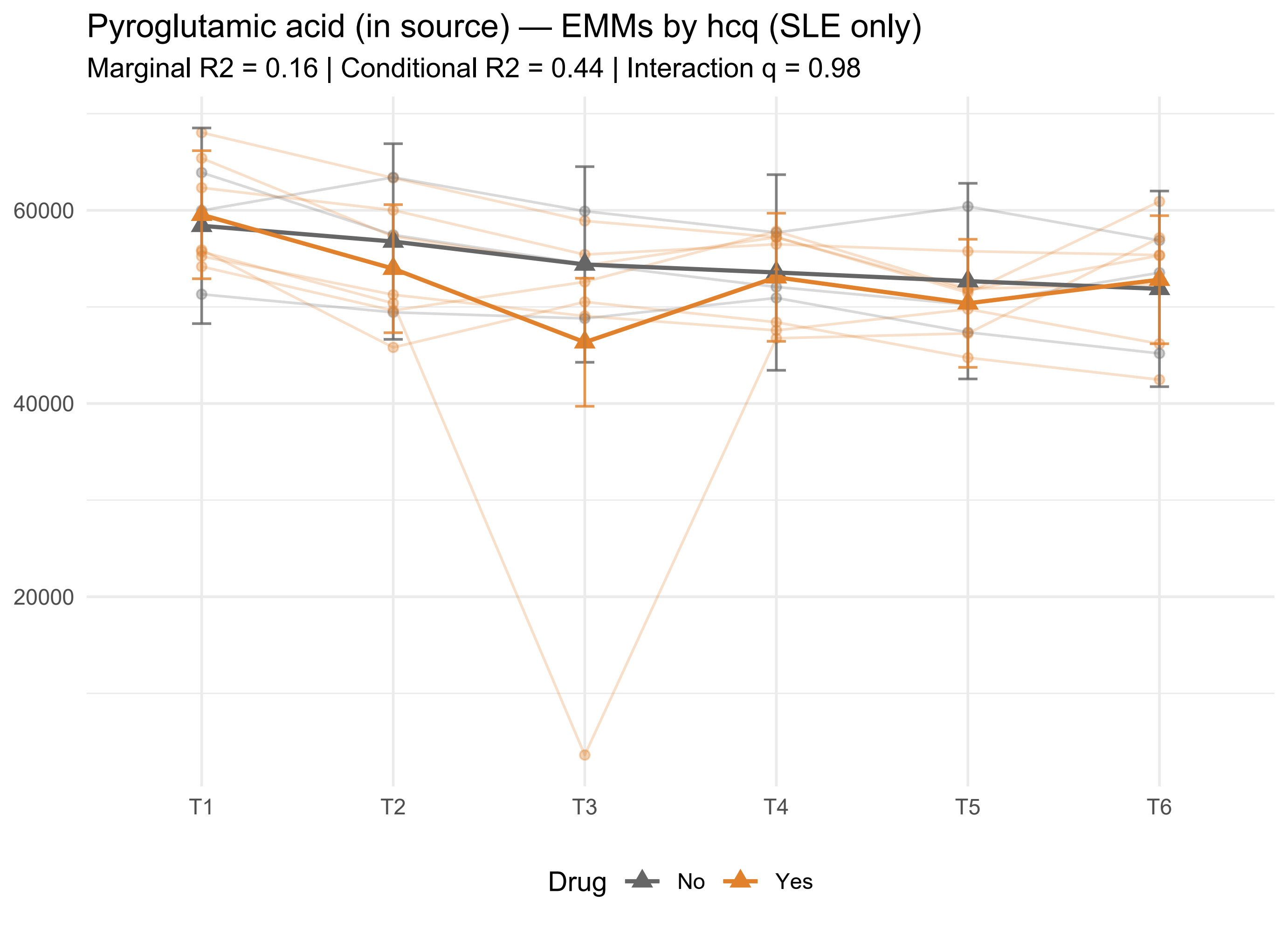

Pyroglutamic acid (in source) — EMMs by hcq (SLE only)
Marginal R2 = 0.16 | Conditional R2 = 0.44 | Interaction q = 0.98
60000
40000
20000
T1
T2
T3
T4
T5
T6
Drug
No
Yes

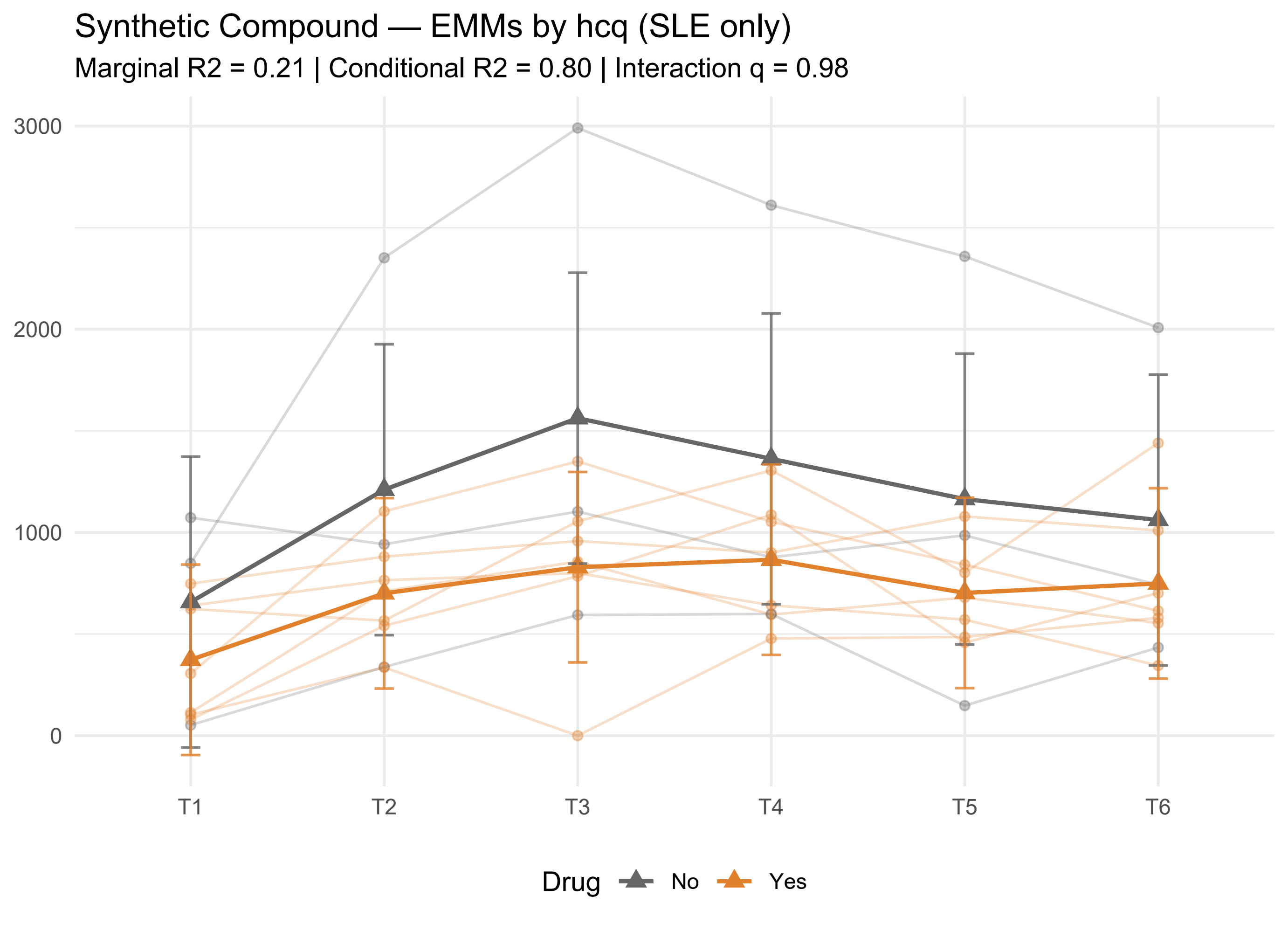

Synthetic Compound — EMMs by hcq (SLE only)
Marginal R2 = 0.21 | Conditional R2 = 0.80 | Interaction q = 0.98
3000
2000
1000
0
T1
T2
T3
T4
T5
T6
Drug
No
Yes

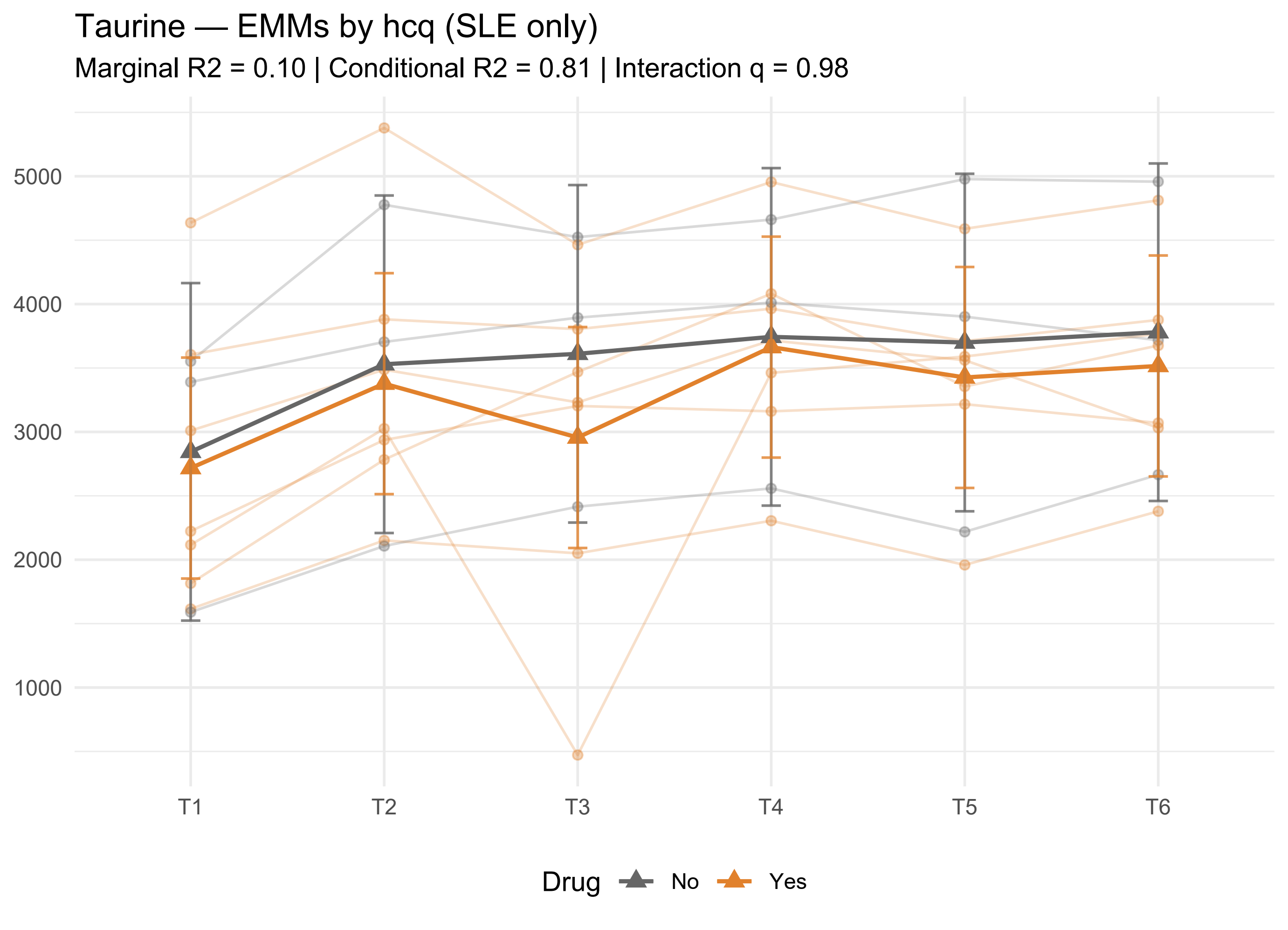
Taurine — EMMs by hcq (SLE only)
Marginal R2 = 0.10 | Conditional R2 = 0.81 | Interaction q = 0.98
5000
4000
3000
2000
1000
T1
T2
T3
T4
T5
T6
Drug
No
Yes

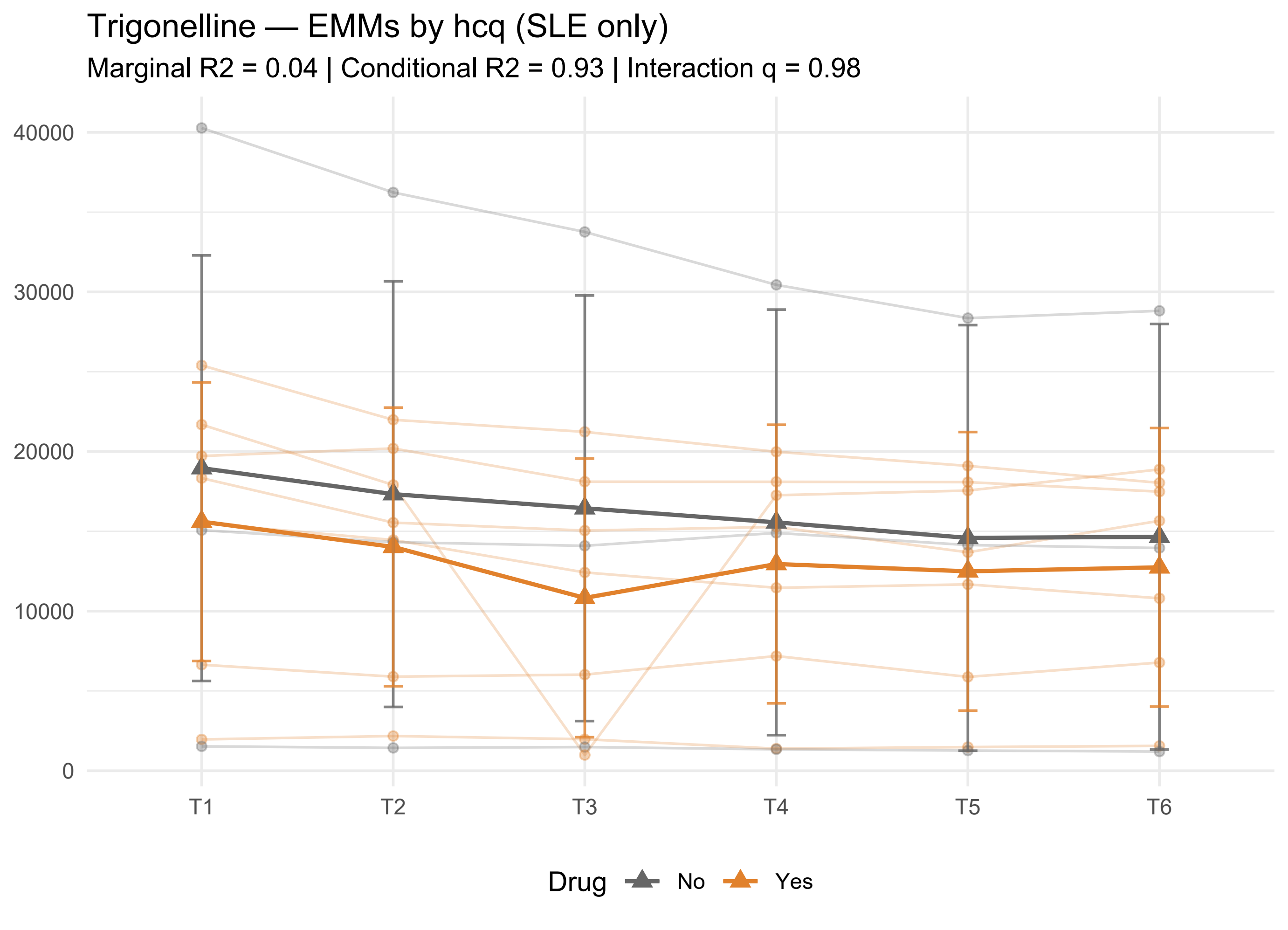
Trigonelline — EMMs by hcq (SLE only)
Marginal R2 = 0.04 | Conditional R2 = 0.93 | Interaction q = 0.98
40000
30000
20000
10000
0
T1
T2
T3
T4
T5
T6
Drug
No
Yes

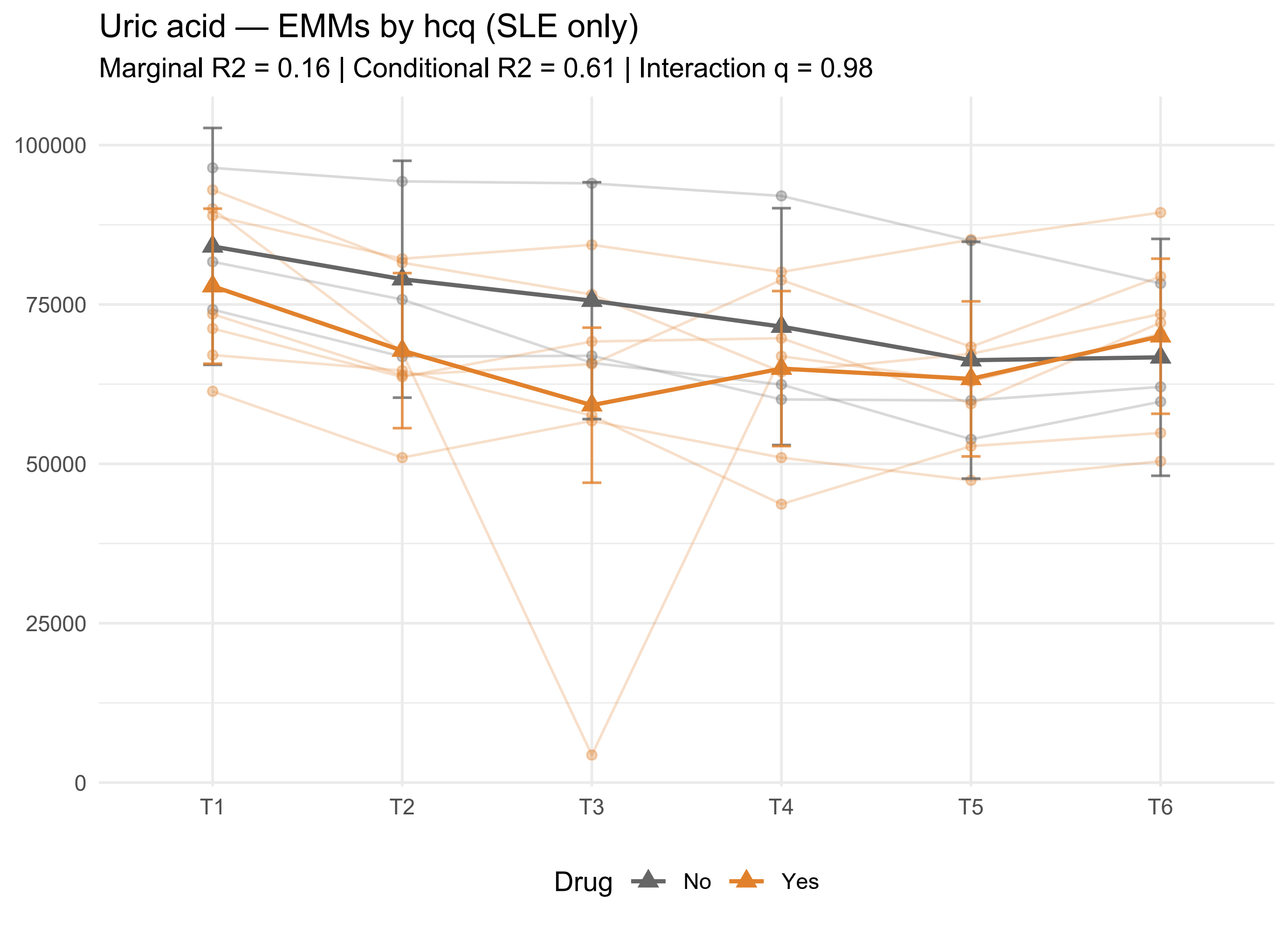

Uric acid — EMMs by hcq (SLE only)
Marginal R2 = 0.16 | Conditional R2 = 0.61 | Interaction q = 0.98
100000
75000
50000
25000
0
T1
T2
T3
T4
T5
T6
Drug
No
Yes

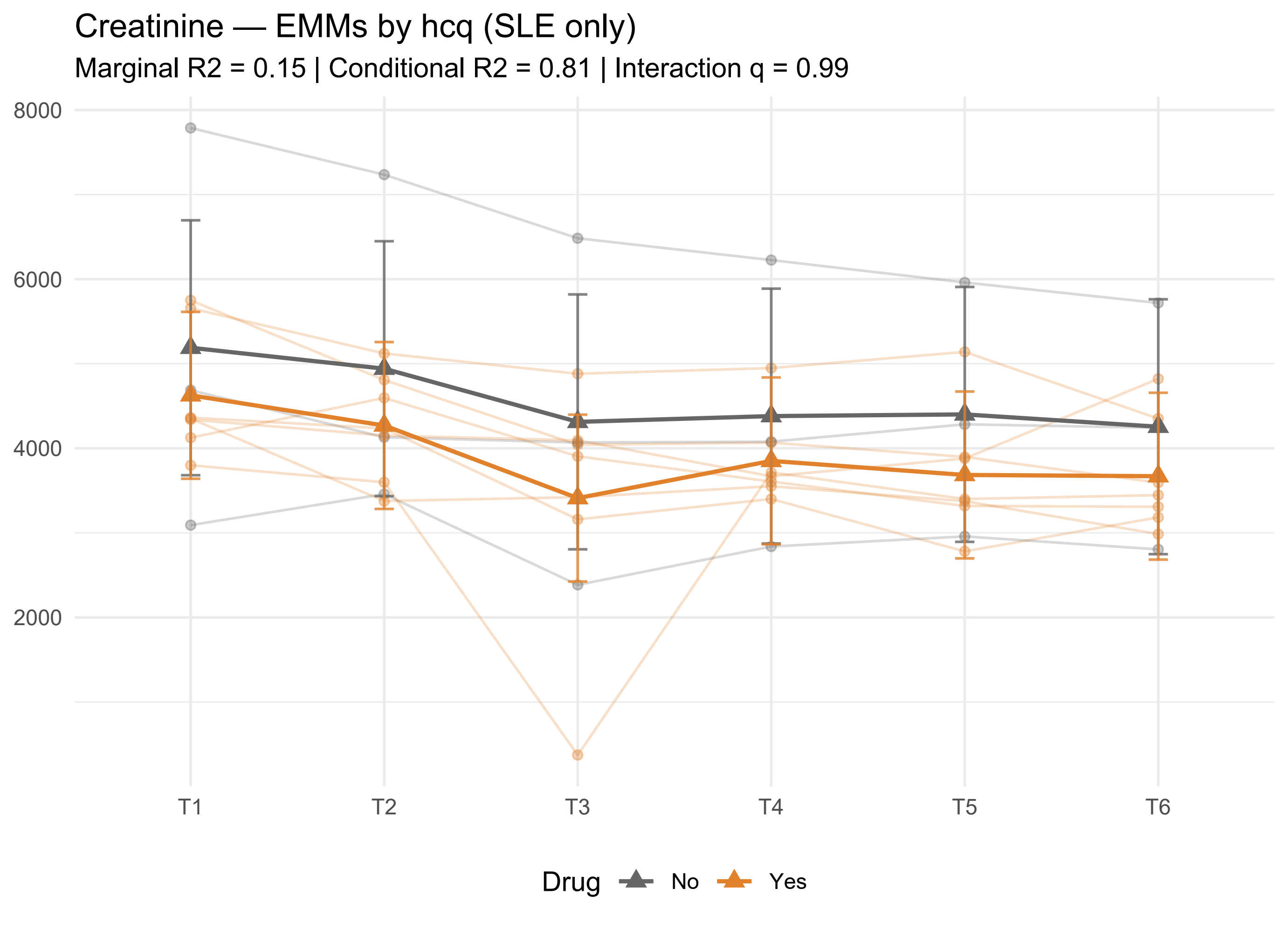

Creatinine — EMMs by hcq (SLE only)
Marginal R2 = 0.15 | Conditional R2 = 0.81 | Interaction q = 0.99
8000
6000
4000
2000
T1
T2
T3
T4
T5
T6
Drug
No
Yes

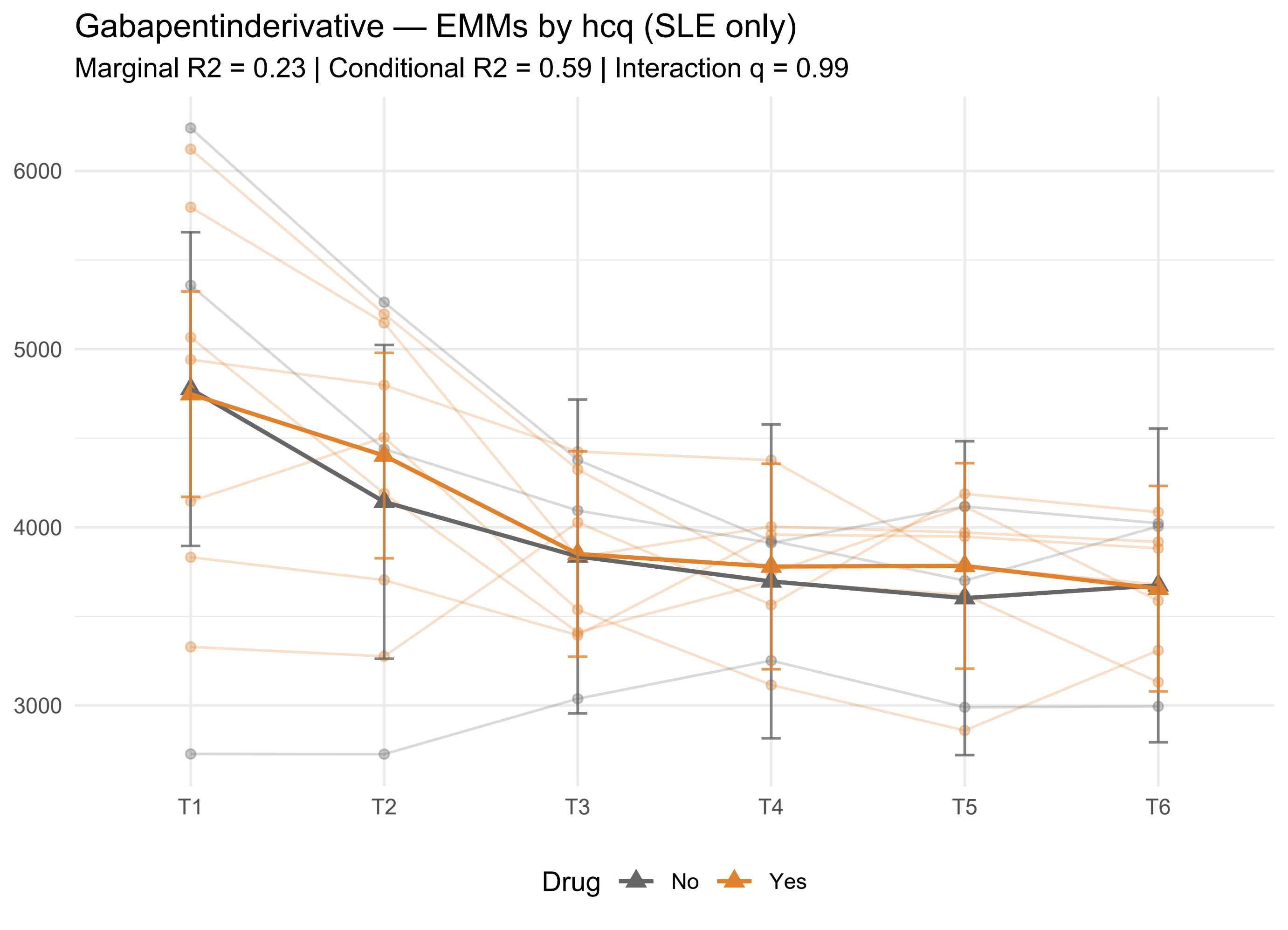

Gabapentinderivative — EMMs by hcq (SLE only)
Marginal R2 = 0.23 | Conditional R2 = 0.59 | Interaction q = 0.99
6000
5000
4000
3000
T1
T2
T3
T4
T5
T6
Drug
No
Yes

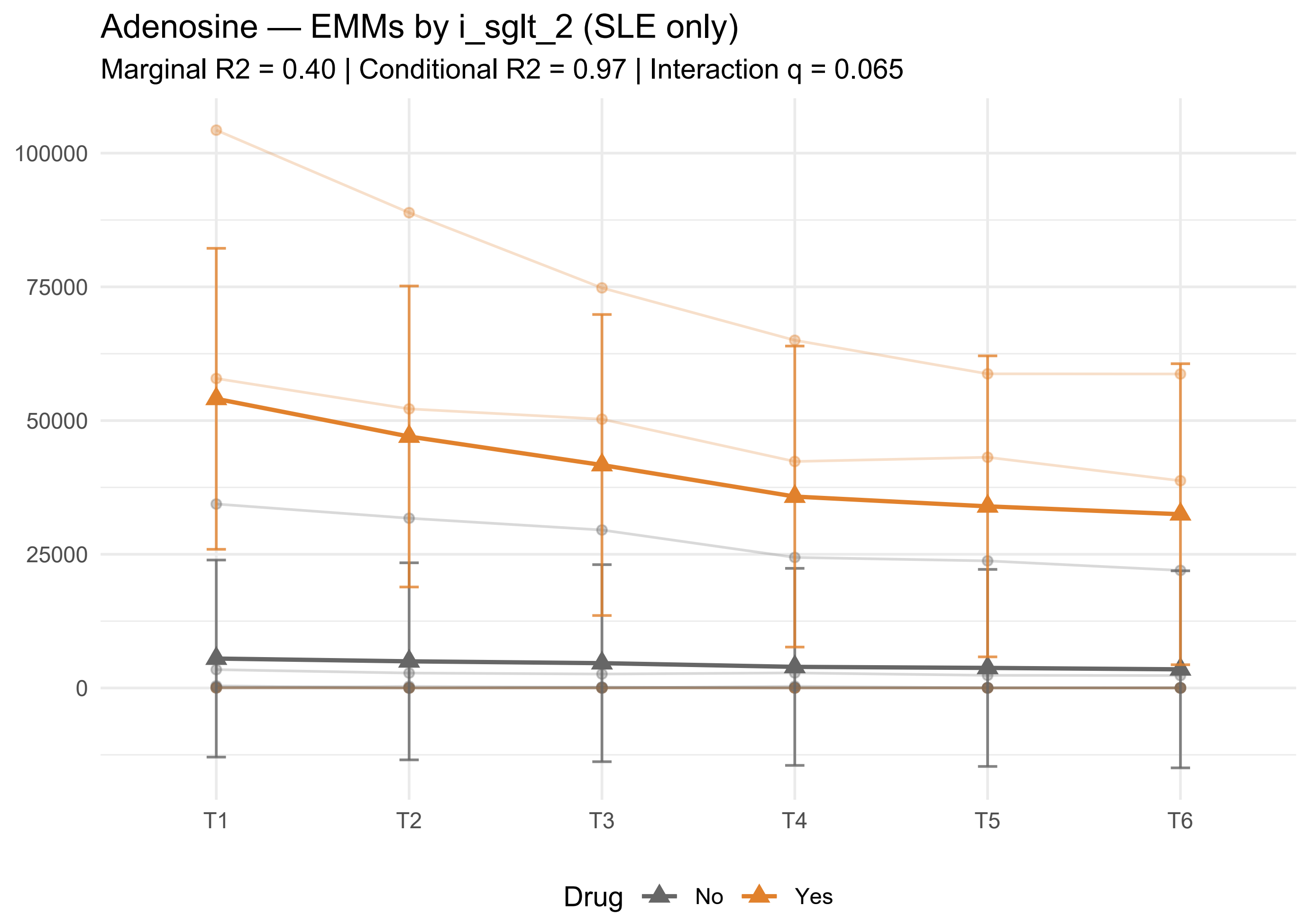
Adenosine — EMMs by i_sglt_2 (SLE only)
Marginal R2 = 0.40 | Conditional R2 = 0.97 | Interaction q = 0.065
100000
75000
50000
25000
0
T1
T2
T3
T4
T5
T6
Drug
No
Yes

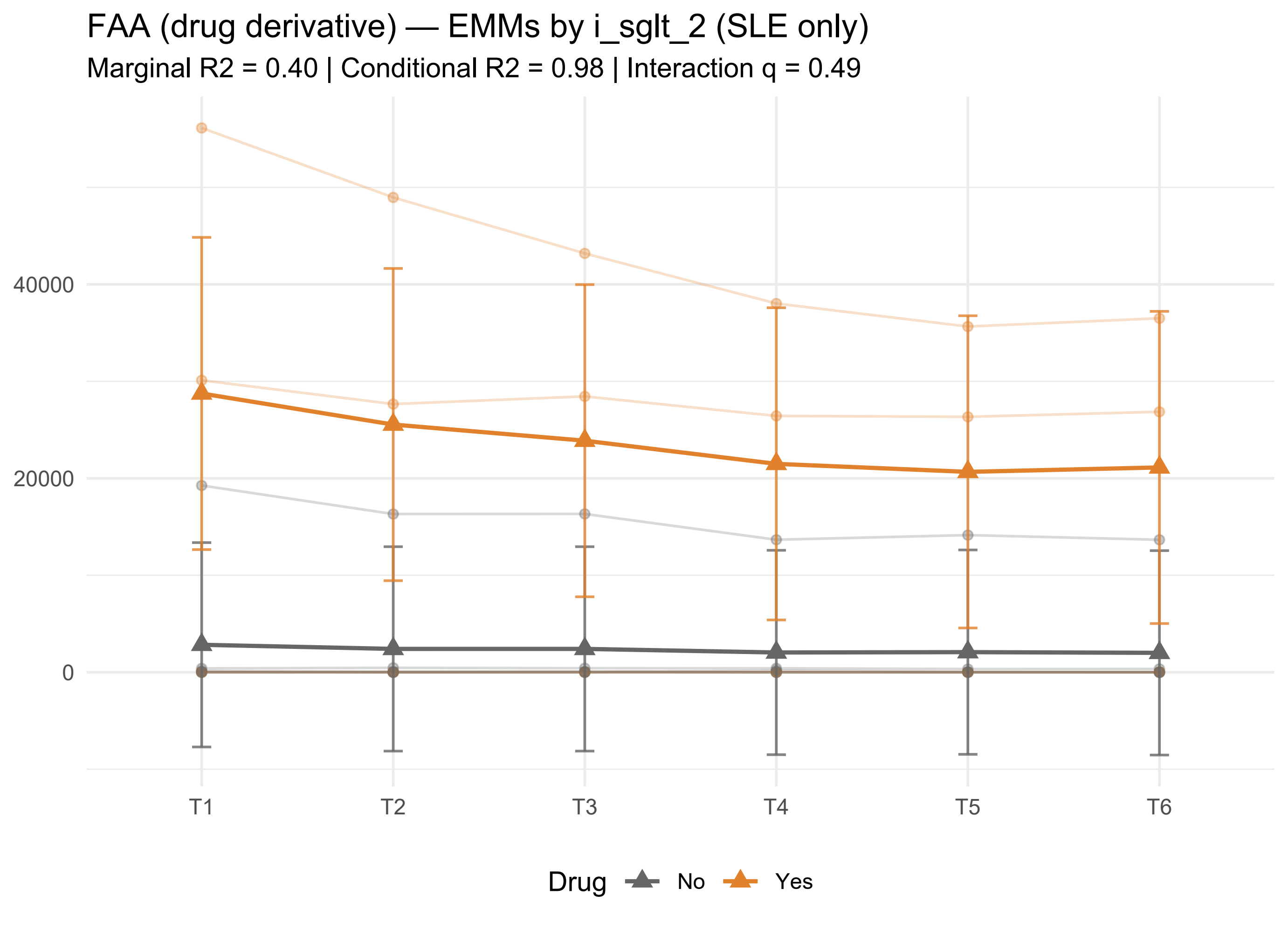
FAA (drug derivative) — EMMs by i_sglt_2 (SLE only)
Marginal R2 = 0.40 | Conditional R2 = 0.98 | Interaction q = 0.49
40000
20000
0
T1
T2
T3
T4
T5
T6
Drug
No
Yes

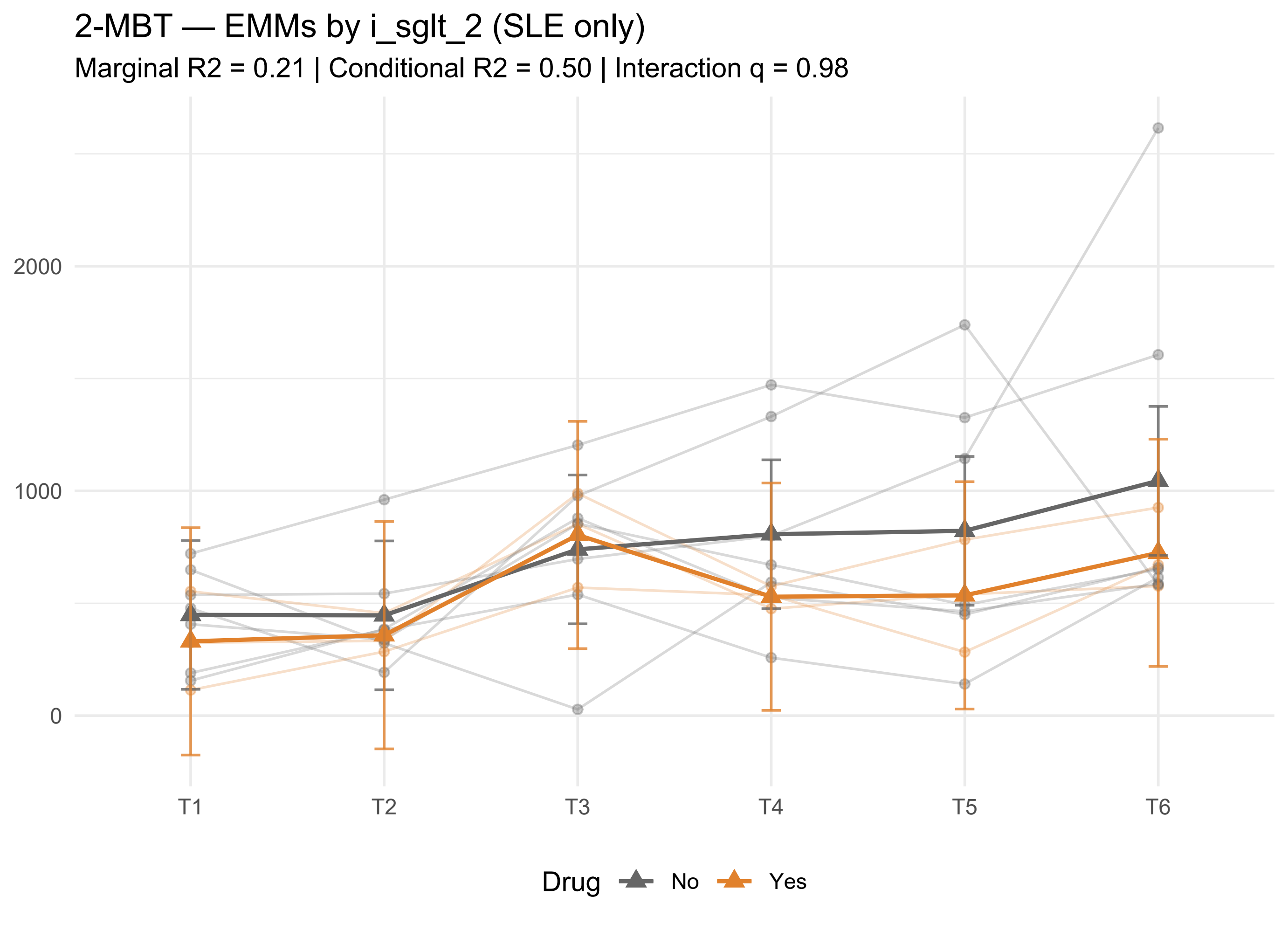

2-MBT — EMMs by i_sglt_2 (SLE only)
Marginal R2 = 0.21 | Conditional R2 = 0.50 | Interaction q = 0.98
2000
1000
0
T1
T2
T3
T4
T5
T6
Drug
No
Yes

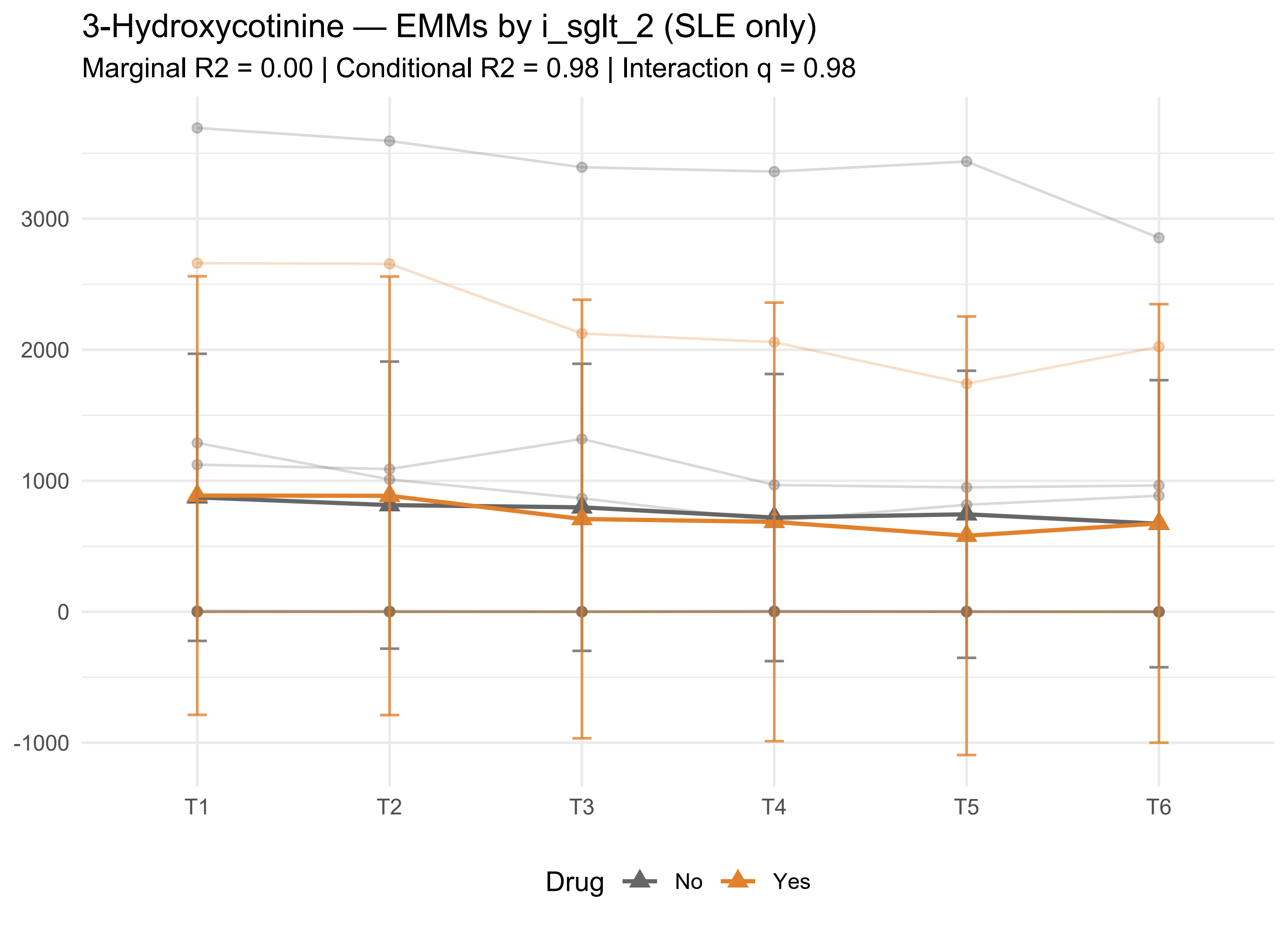

3-Hydroxycotinine — EMMs by i_sglt_2 (SLE only)
Marginal R2 = 0.00 | Conditional R2 = 0.98 | Interaction q = 0.98
3000
2000
1000
0
-1000
T1
T2
T3
T4
T5
T6
Drug
No
Yes

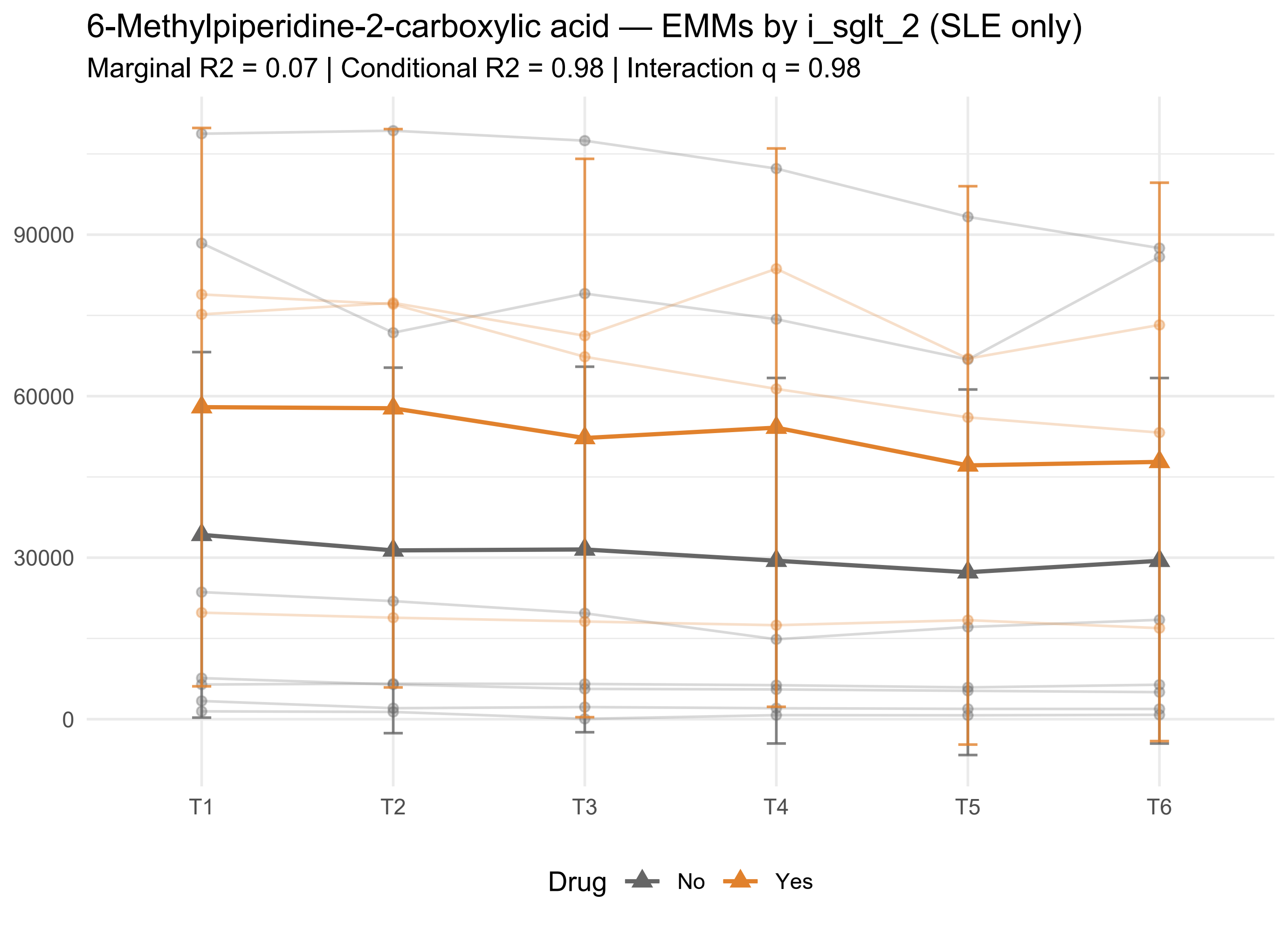

6-Methylpiperidine-2-carboxylic acid — EMMs by i_sglt_2 (SLE only)
Marginal R2 = 0.07 | Conditional R2 = 0.98 | Interaction q = 0.98
90000
60000
30000
0
T1
T2
T3
T4
T5
T6
Drug
No
Yes

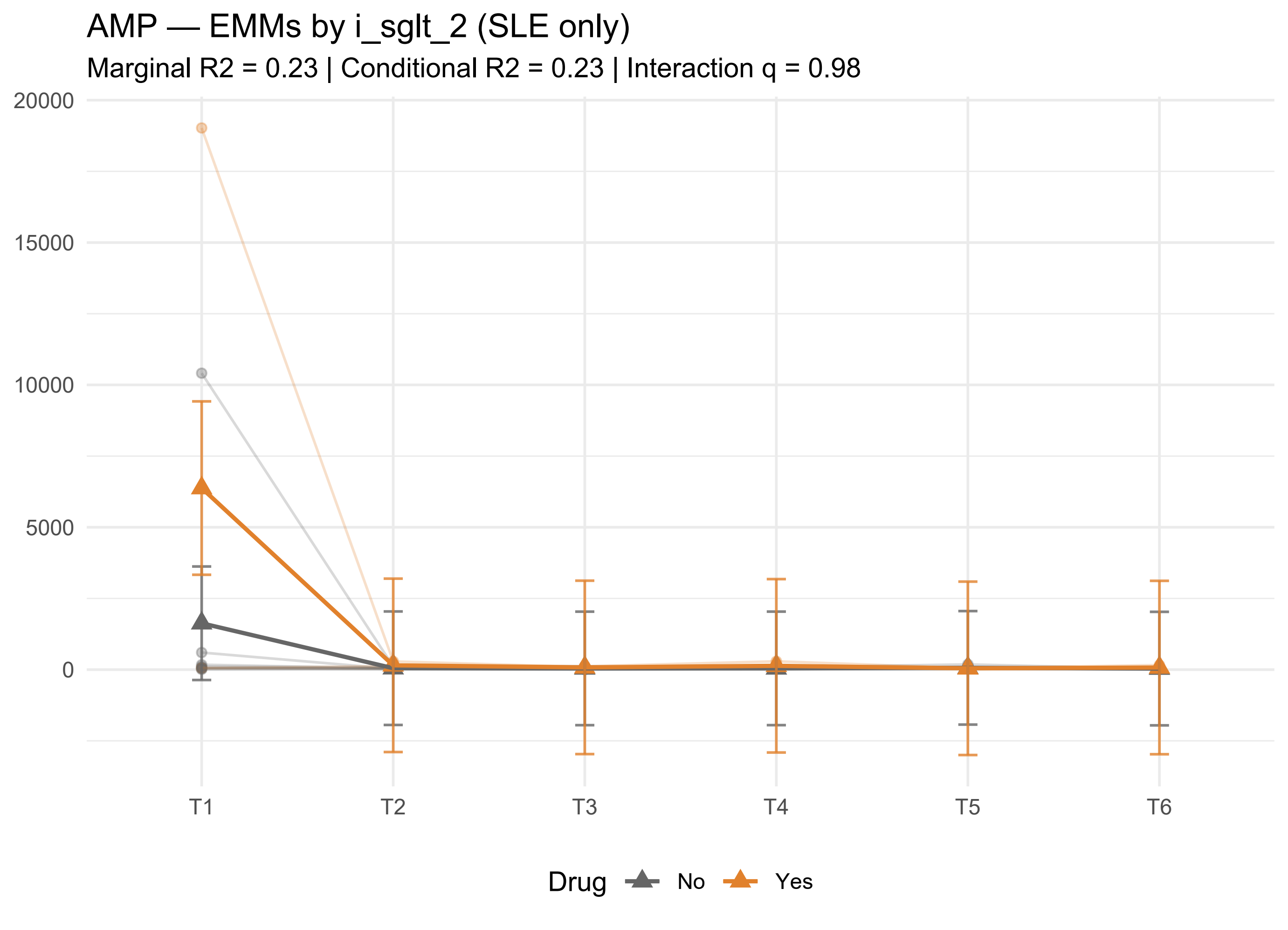
AMP — EMMs by i_sglt_2 (SLE only)
Marginal R2 = 0.23 | Conditional R2 = 0.23 | Interaction q = 0.98
20000
15000
10000
5000
0
T1
T2
T3
T4
T5
T6
Drug
No
Yes

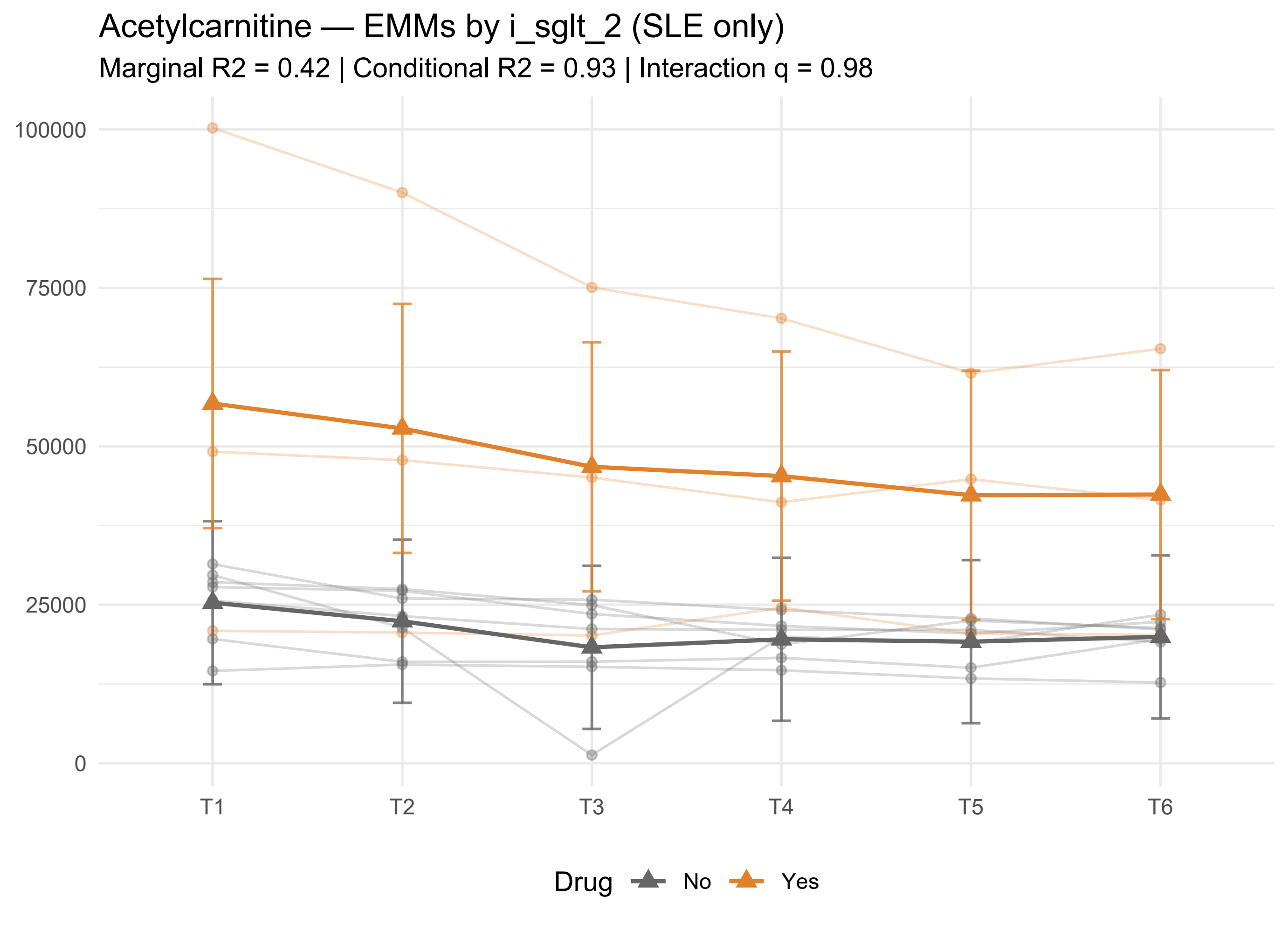

Acetylcarnitine — EMMs by i_sglt_2 (SLE only)
Marginal R2 = 0.42 | Conditional R2 = 0.93 | Interaction q = 0.98
100000
75000
50000
25000
0
T1
T2
T3
T4
T5
T6
Drug
No
Yes

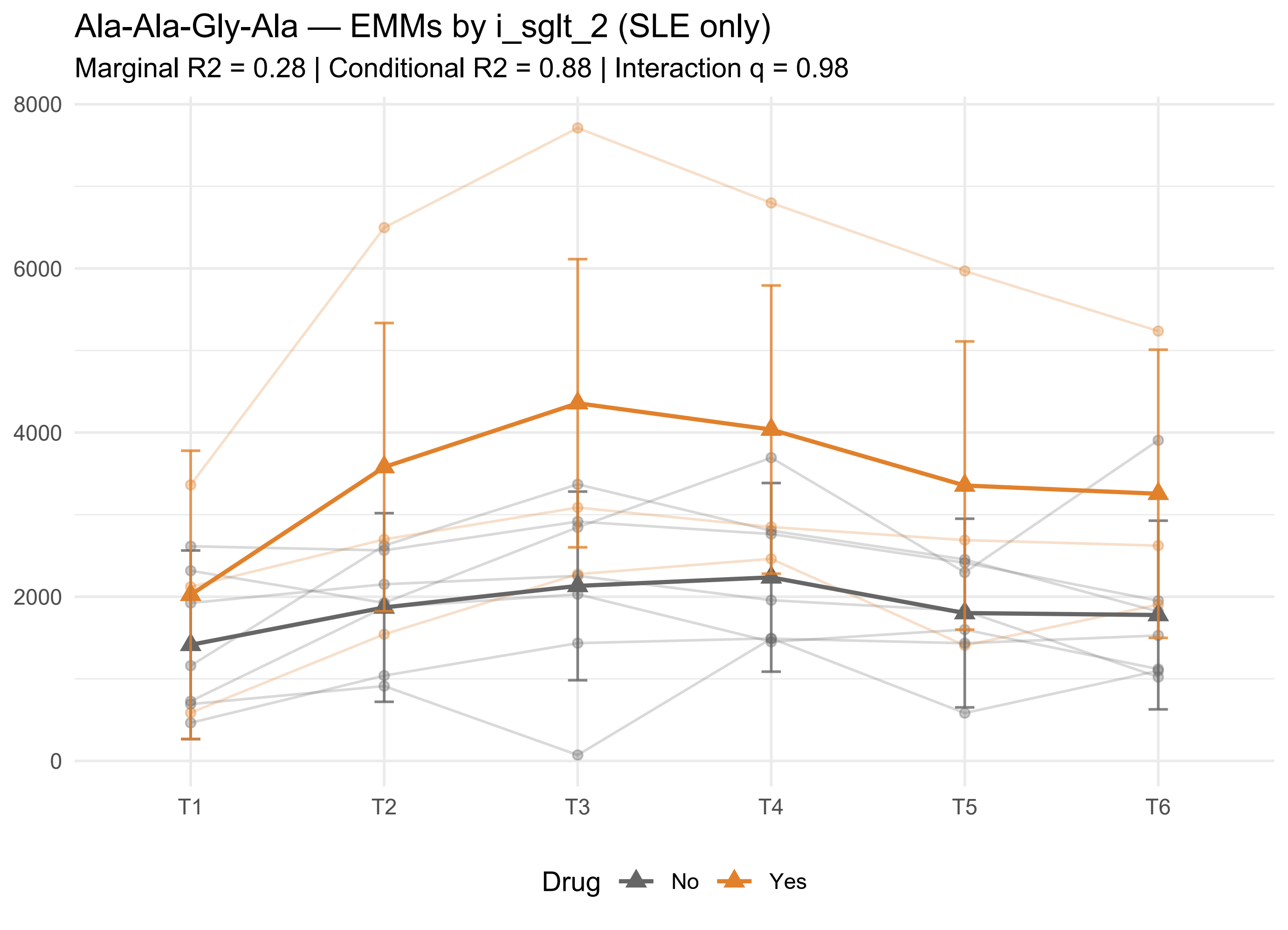

Ala-Ala-Gly-Ala — EMMs by i_sglt_2 (SLE only)
Marginal R2 = 0.28 | Conditional R2 = 0.88 | Interaction q = 0.98
8000
6000
4000
2000
0
T1
T2
T3
T4
T5
T6
Drug
No
Yes

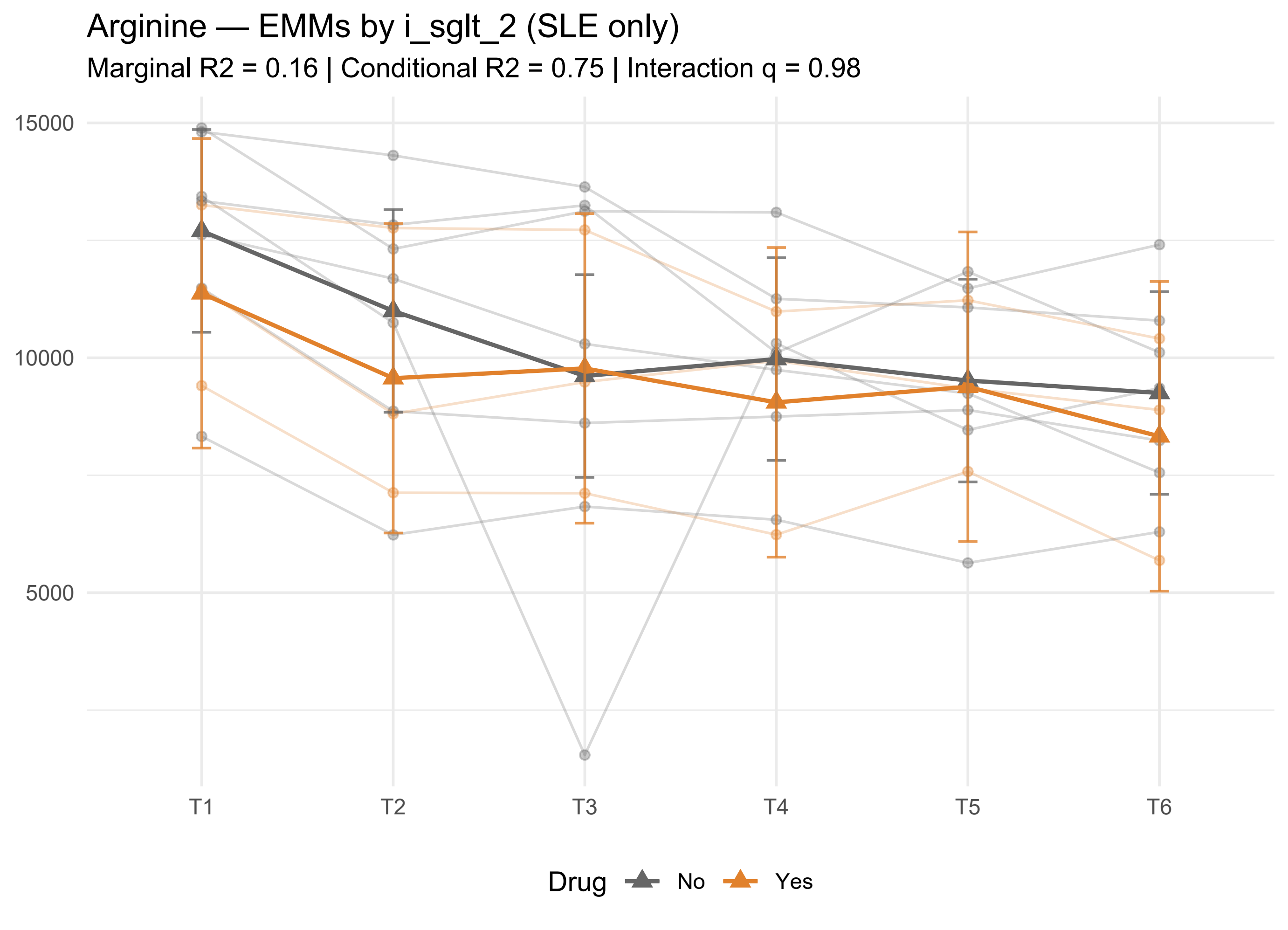

Arginine — EMMs by i_sglt_2 (SLE only)
Marginal R2 = 0.16 | Conditional R2 = 0.75 | Interaction q = 0.98
15000
10000
5000
T1
T2
T3
T4
T5
T6
Drug
No
Yes

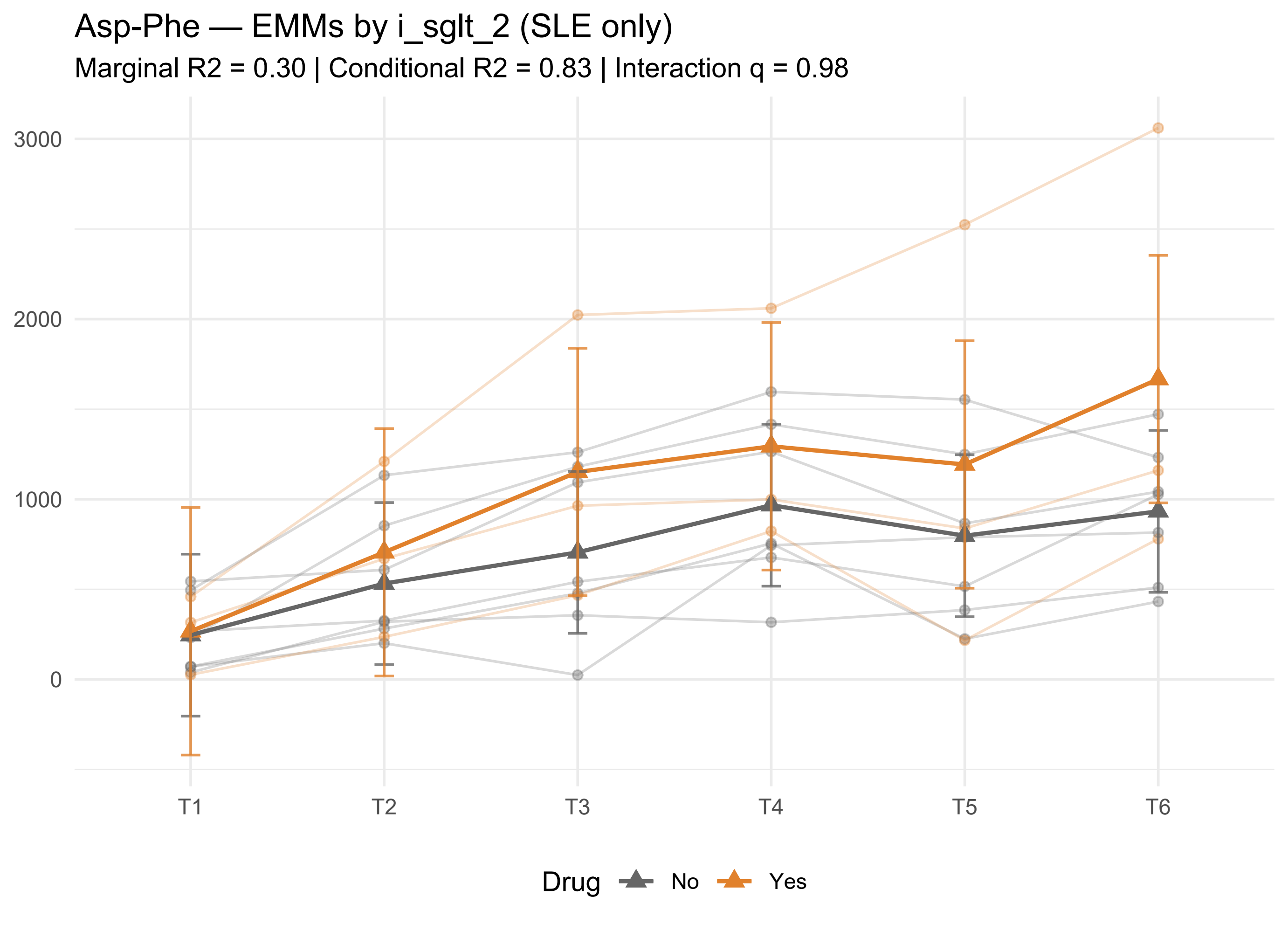

Asp-Phe — EMMs by i_sglt_2 (SLE only)
Marginal R2 = 0.30 | Conditional R2 = 0.83 | Interaction q = 0.98
3000
2000
1000
0
T1
T2
T3
T4
T5
T6
Drug
No
Yes

Betaine — EMMs by i_sglt_2 (SLE only)

Marginal R2 = 0.09 | Conditional R2 = 0.93 | Interaction q = 0.98

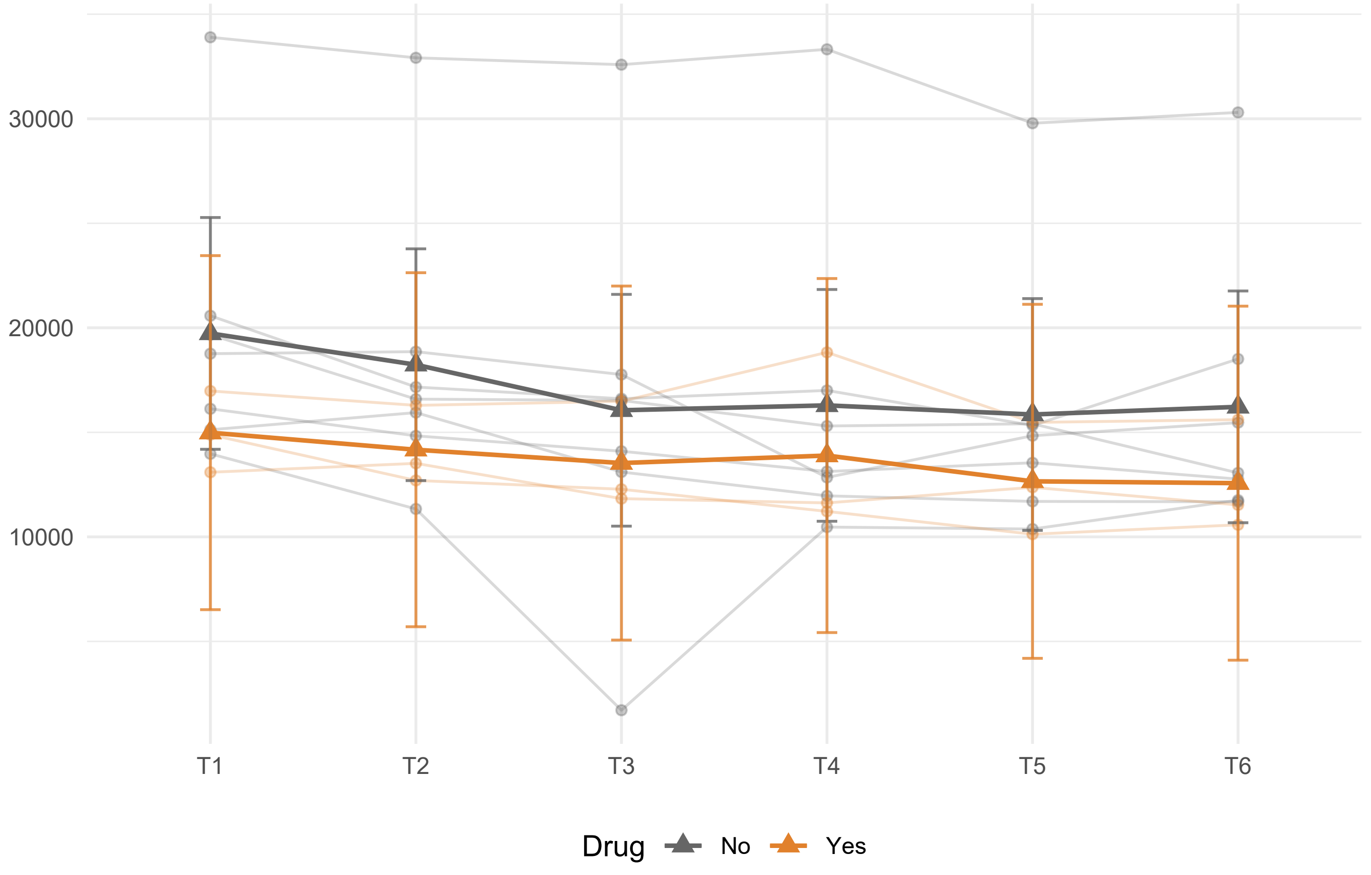

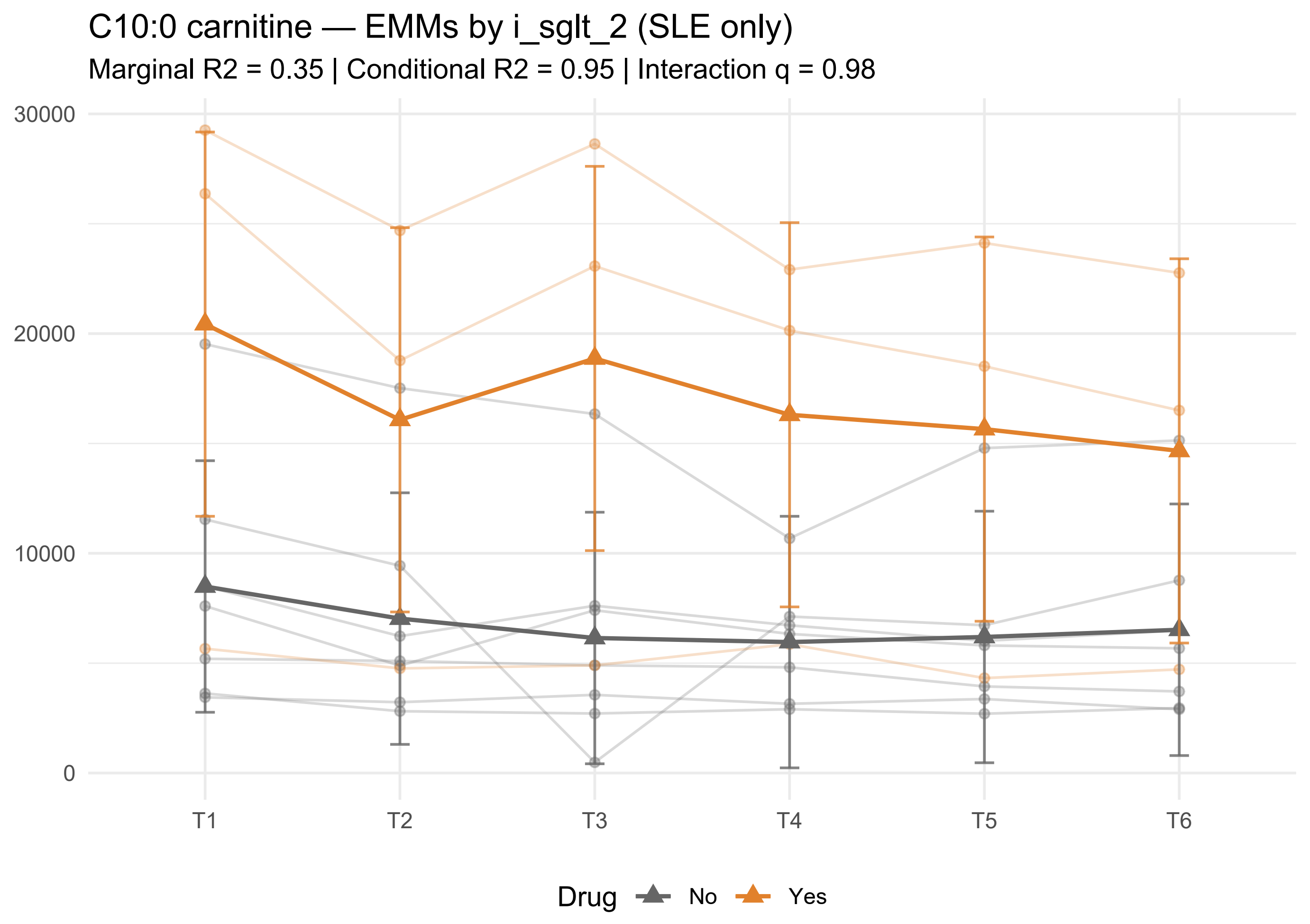

C10:0 carnitine — EMMs by i_sglt_2 (SLE only)
Marginal R2 = 0.35 | Conditional R2 = 0.95 | Interaction q = 0.98
30000
20000
10000
0
T1
T2
T3
T4
T5
T6
Drug
No
Yes

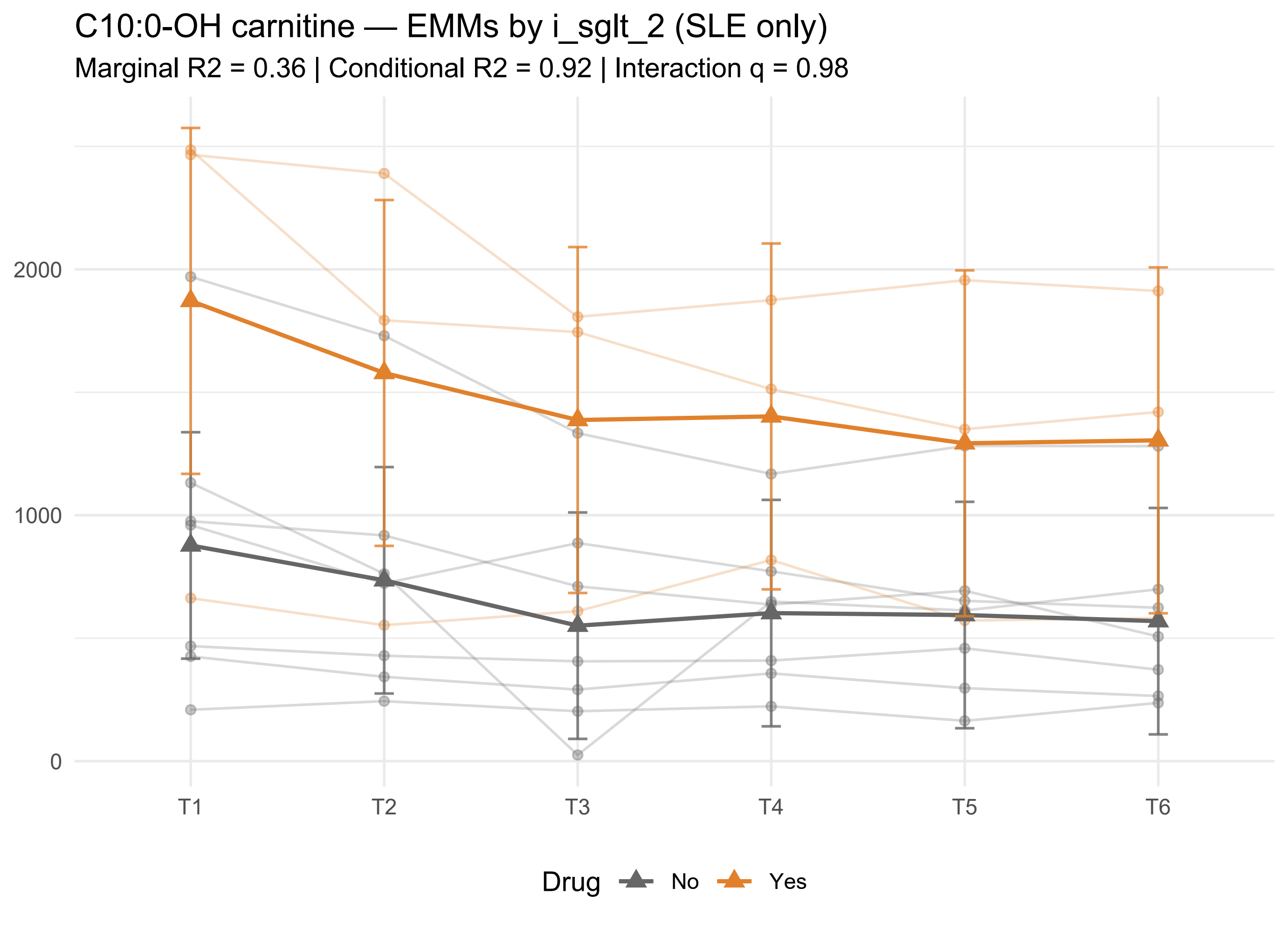

C10:0-OH carnitine — EMMs by i_sglt_2 (SLE only)
Marginal R2 = 0.36 | Conditional R2 = 0.92 | Interaction q = 0.98
2000
1000
0
T1
T2
T3
T4
T5
T6
Drug
No
Yes

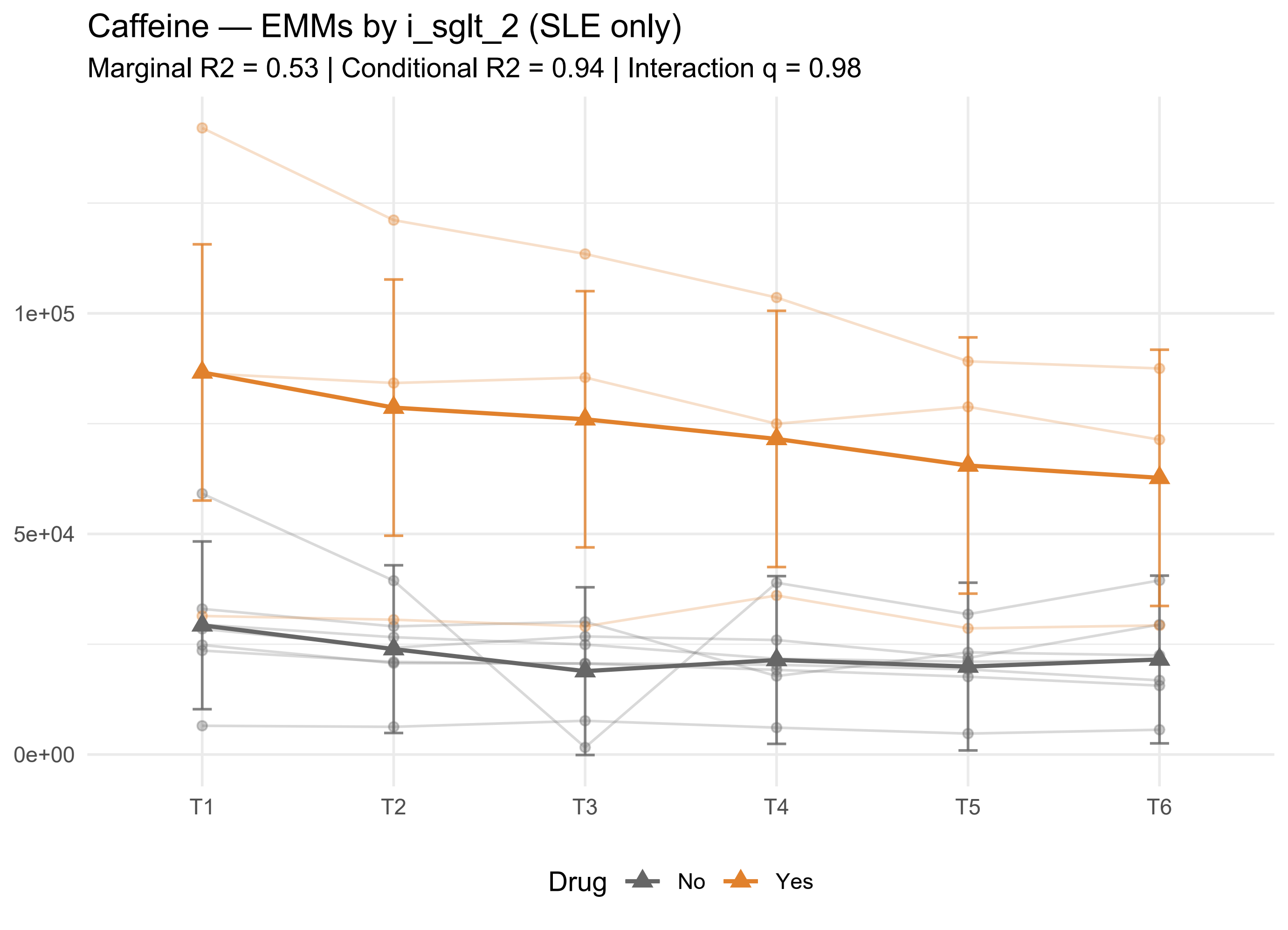

Caffeine — EMMs by i_sglt_2 (SLE only)
Marginal R2 = 0.53 | Conditional R2 = 0.94 | Interaction q = 0.98
1e+05
5e+04
0e+00
T1
T2
T3
T4
T5
T6
Drug
No
Yes

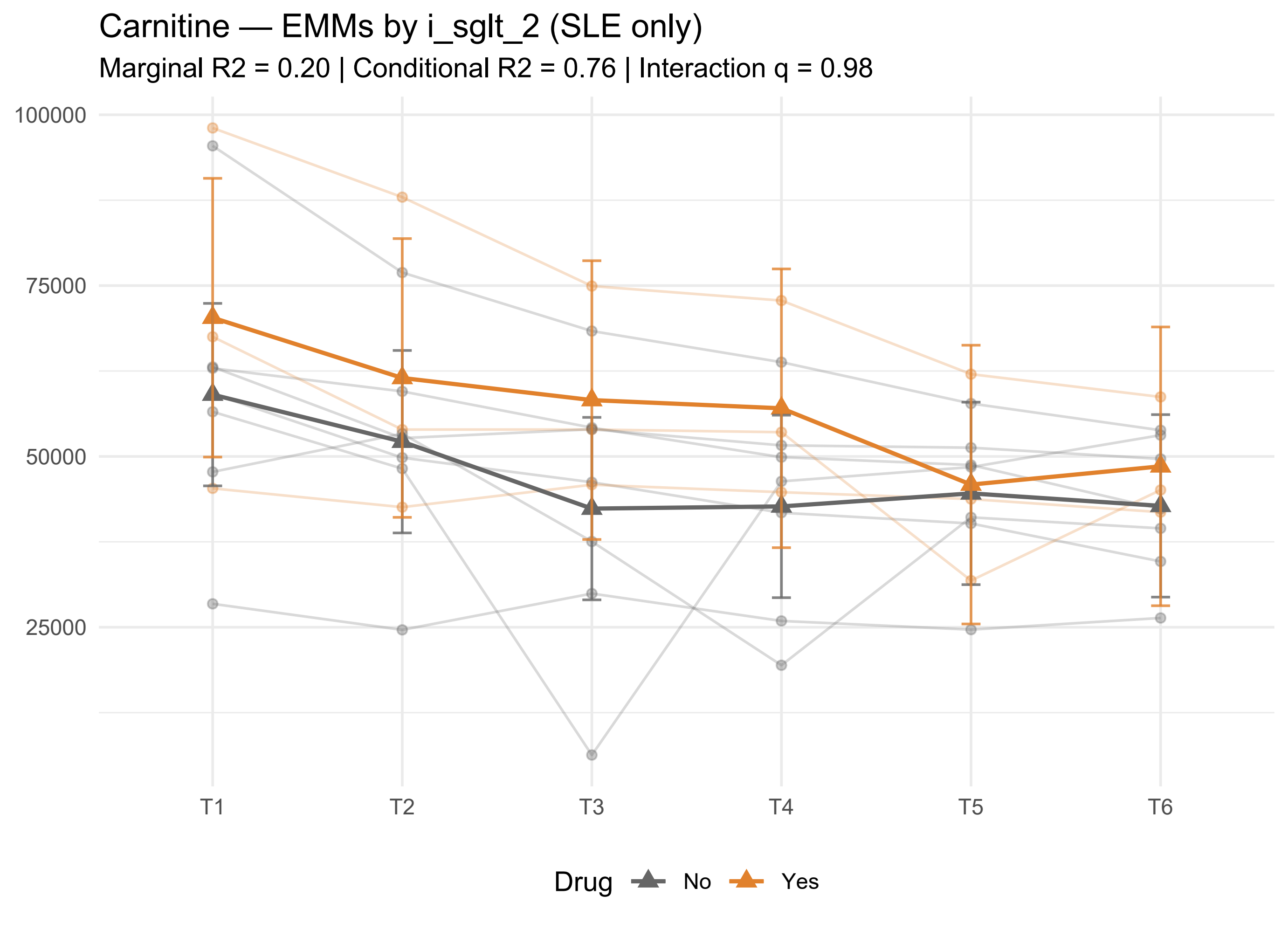
Carnitine — EMMs by i_sglt_2 (SLE only)
Marginal R2 = 0.20 | Conditional R2 = 0.76 | Interaction q = 0.98
100000
75000
50000
25000
T1
T2
T3
T4
T5
T6
Drug
No
Yes

Chlorpheniramine Maleate (Trigonelline) — EMMs by i_sglt_2 (SLE only)

Marginal R2 = 0.53 | Conditional R2 = 0.91 | Interaction q = 0.98

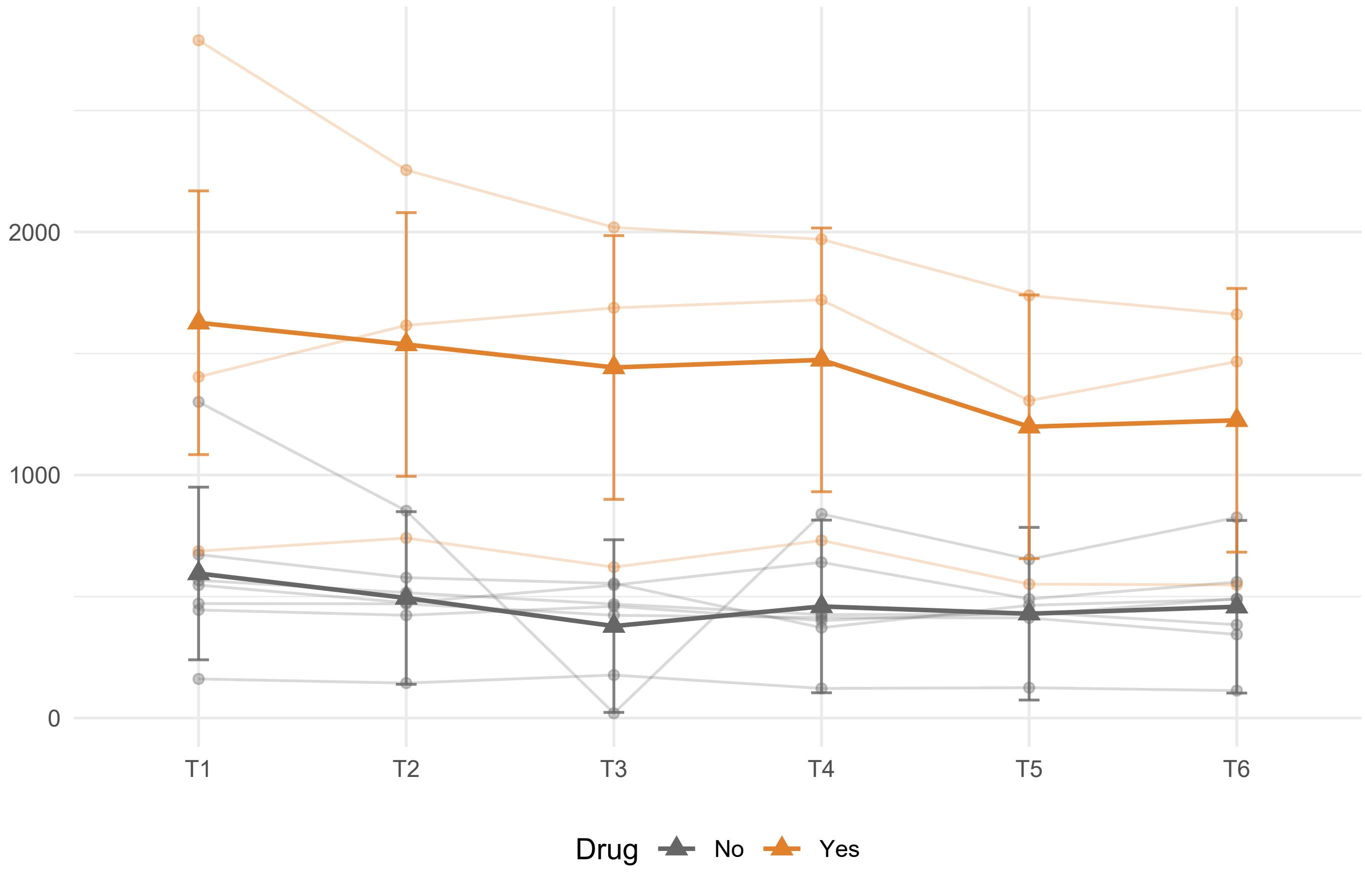

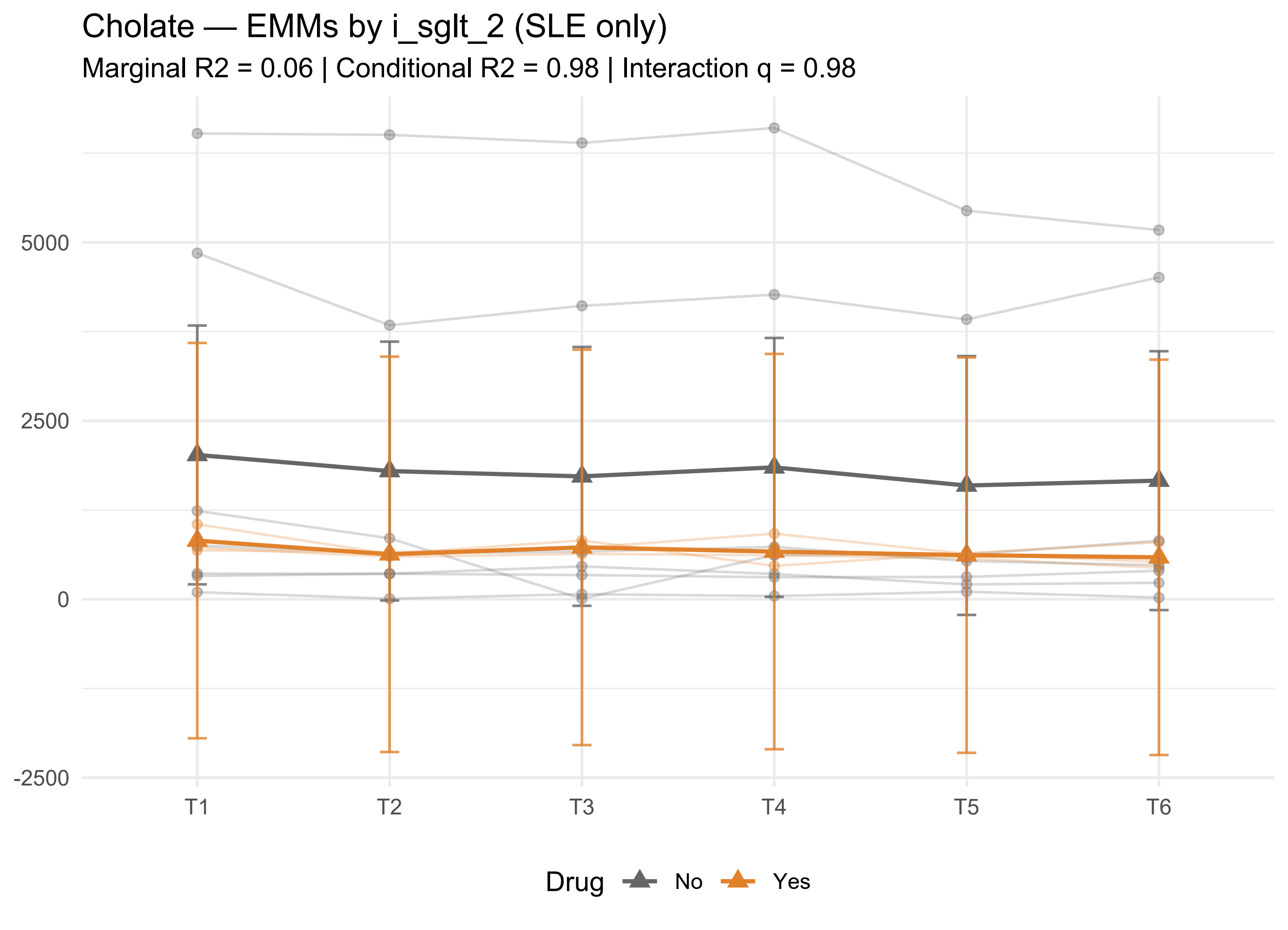

Cholate — EMMs by i_sglt_2 (SLE only)
Marginal R2 = 0.06 | Conditional R2 = 0.98 | Interaction q = 0.98
5000
2500
0
-2500
T1
T2
T3
T4
T5
T6
Drug
No
Yes

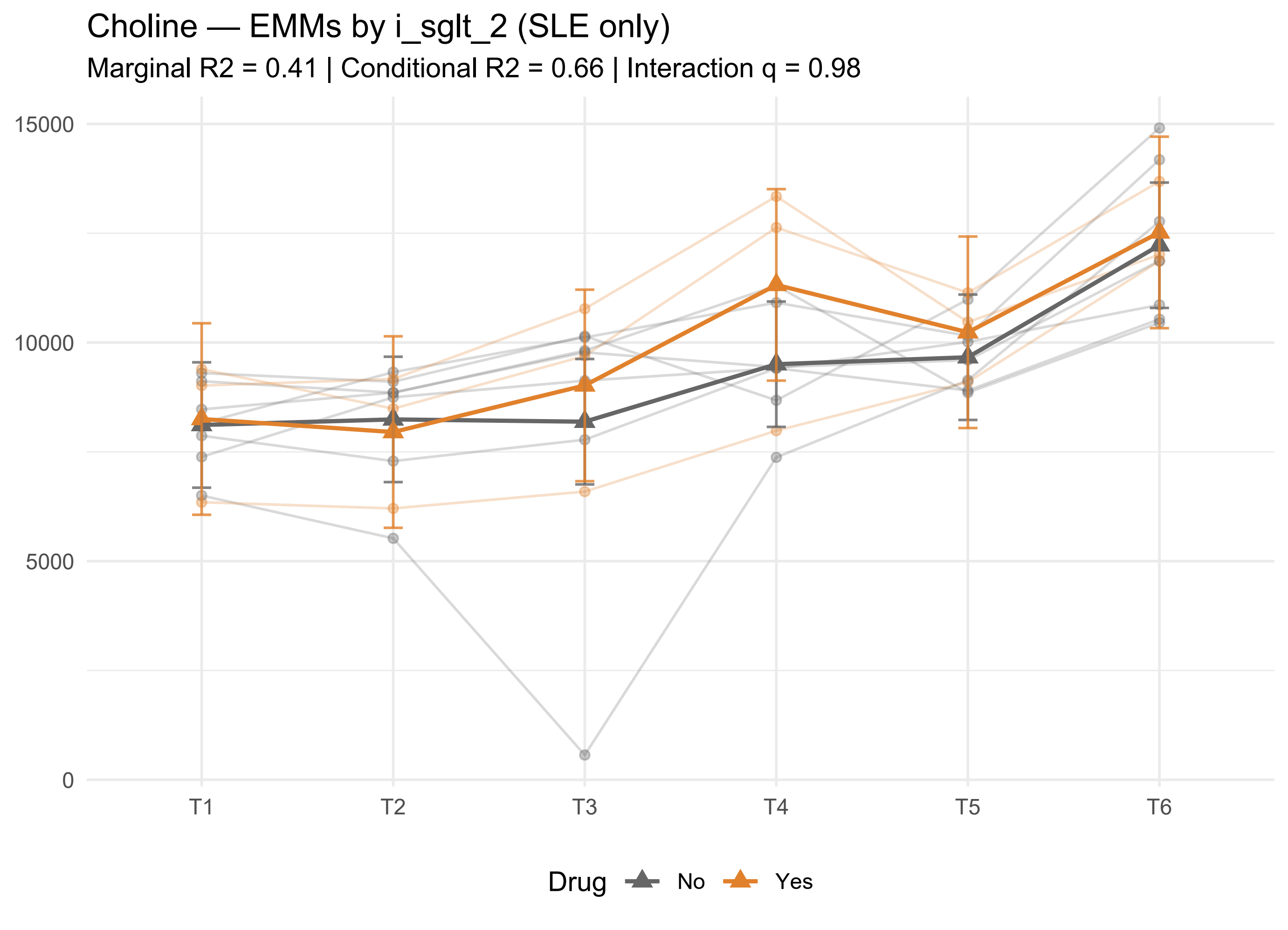

Choline — EMMs by i_sglt_2 (SLE only)
Marginal R2 = 0.41 | Conditional R2 = 0.66 | Interaction q = 0.98
15000
10000
5000
0
T1
T2
T3
T4
T5
T6
Drug
No
Yes

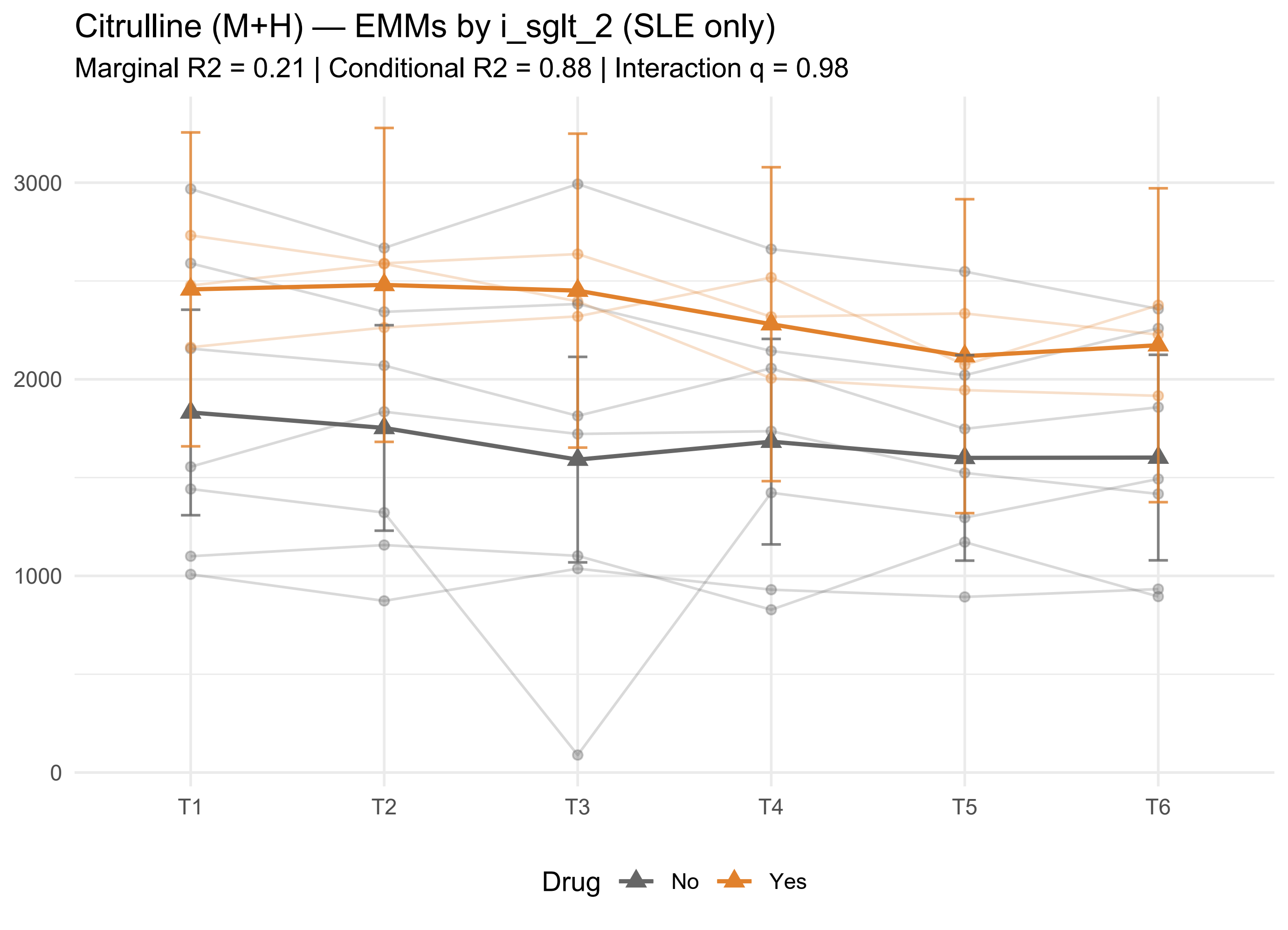

Citrulline (M+H) — EMMs by i_sglt_2 (SLE only)
Marginal R2 = 0.21 | Conditional R2 = 0.88 | Interaction q = 0.98
3000
2000
1000
0
T1
T2
T3
T4
T5
T6
Drug
No
Yes

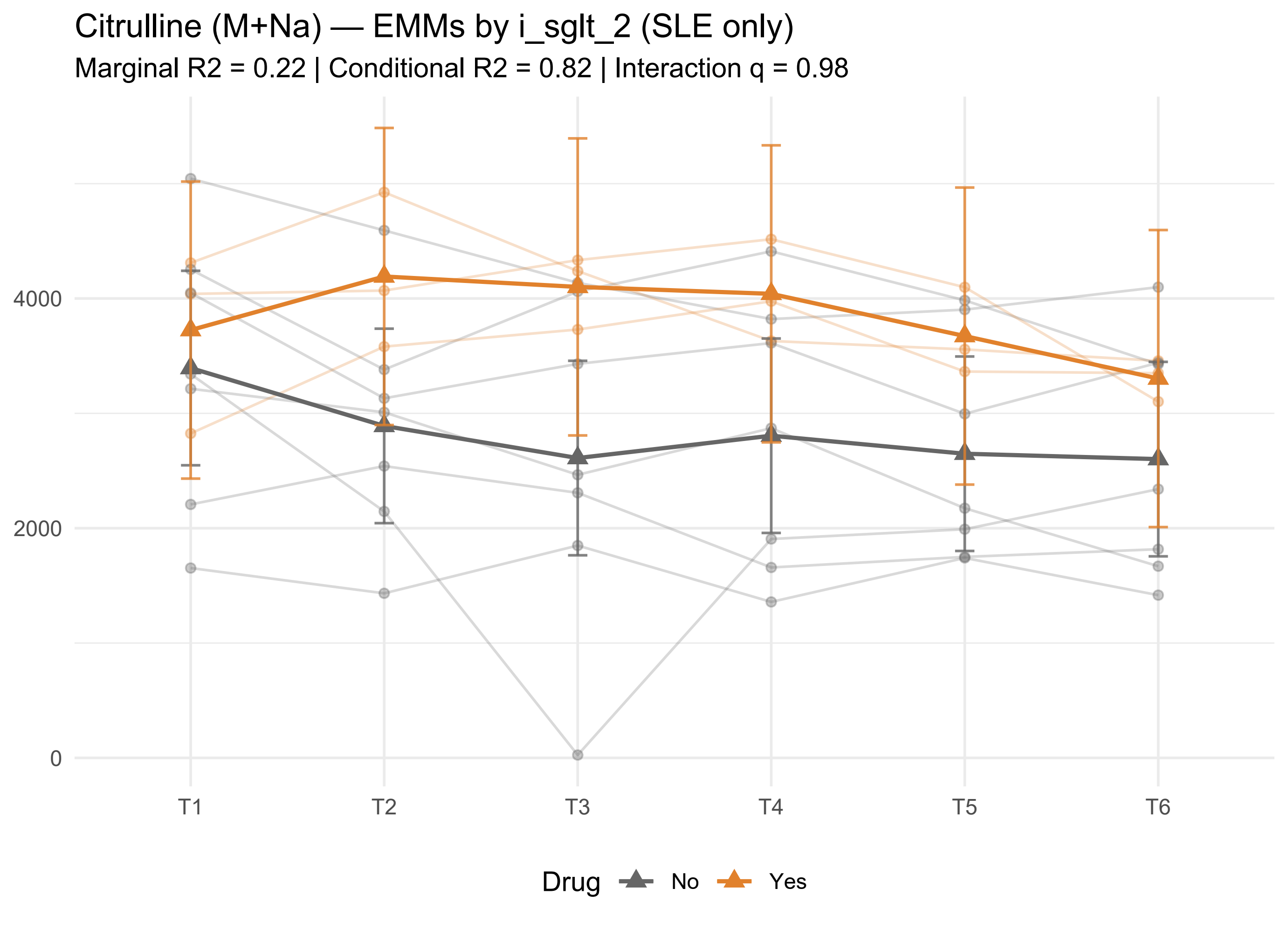

Citrulline (M+Na) — EMMs by i_sglt_2 (SLE only)
Marginal R2 = 0.22 | Conditional R2 = 0.82 | Interaction q = 0.98
4000
2000
0
T1
T2
T3
T4
T5
T6
Drug
No
Yes

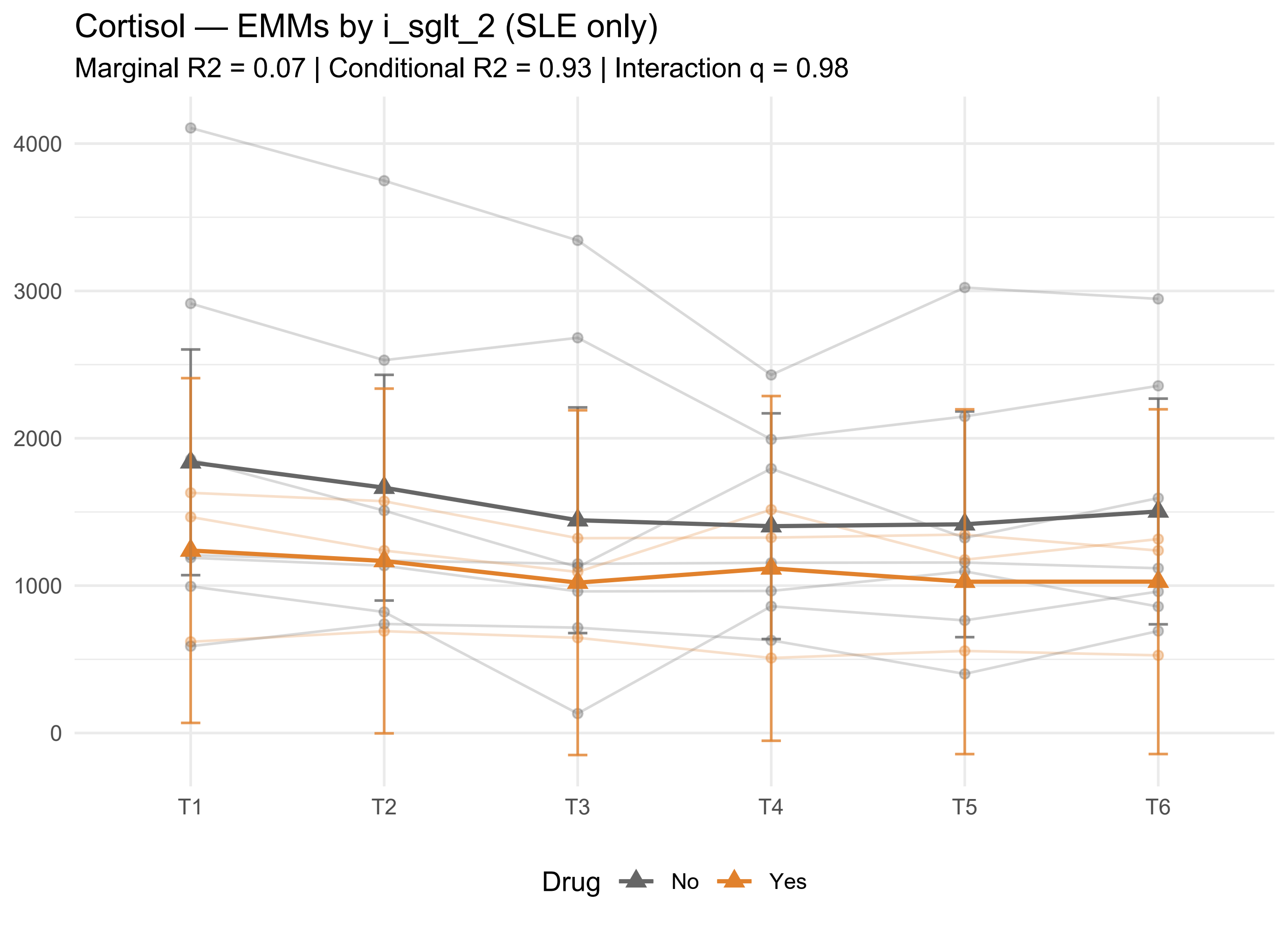

Cortisol — EMMs by i_sglt_2 (SLE only)
Marginal R2 = 0.07 | Conditional R2 = 0.93 | Interaction q = 0.98
4000
3000
2000
1000
0
T1
T2
T3
T4
T5
T6
Drug
No
Yes

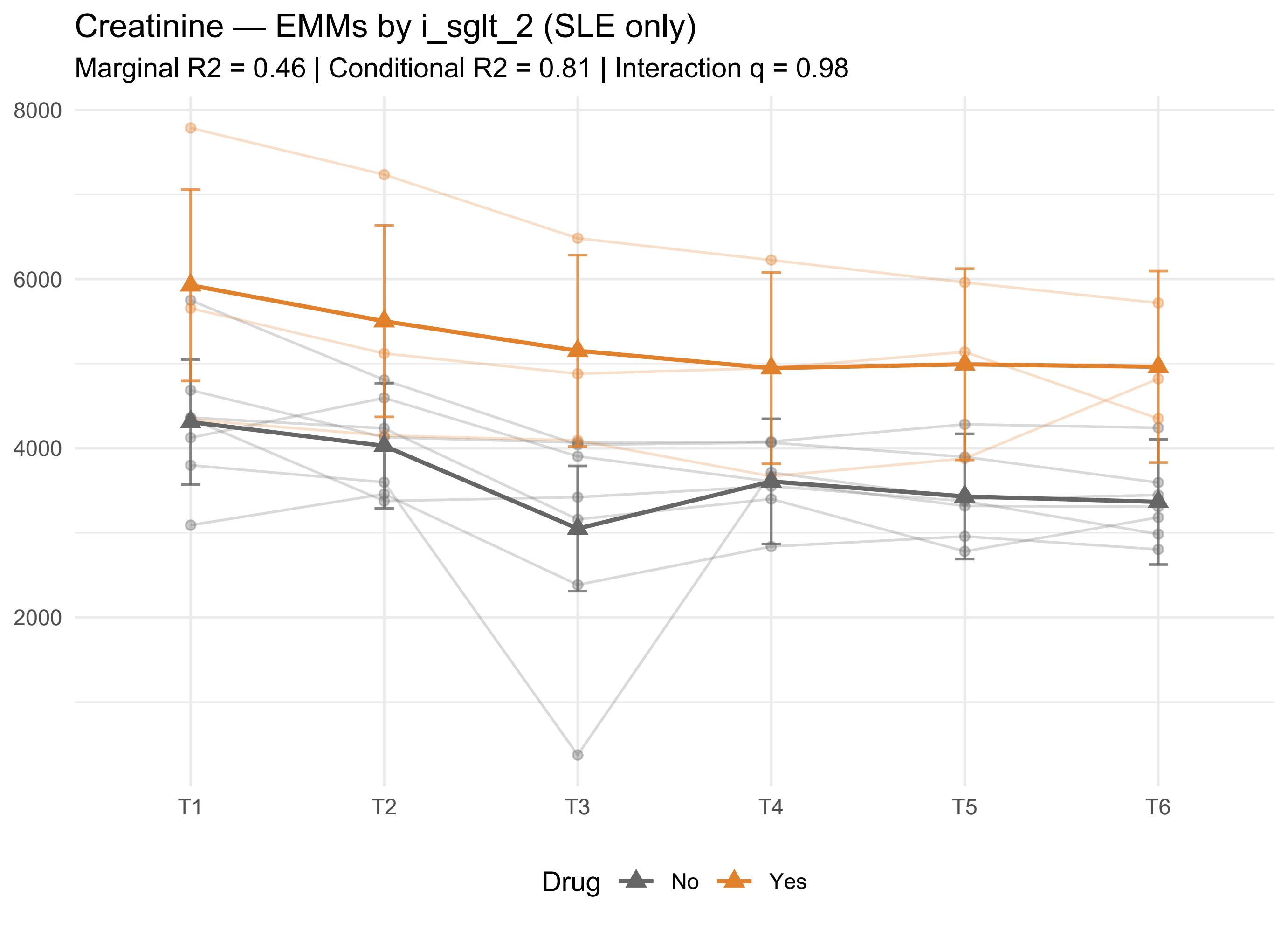

Creatinine — EMMs by i_sglt_2 (SLE only)
Marginal R2 = 0.46 | Conditional R2 = 0.81 | Interaction q = 0.98
8000
6000
4000
2000
T1
T2
T3
T4
T5
T6
Drug
No
Yes

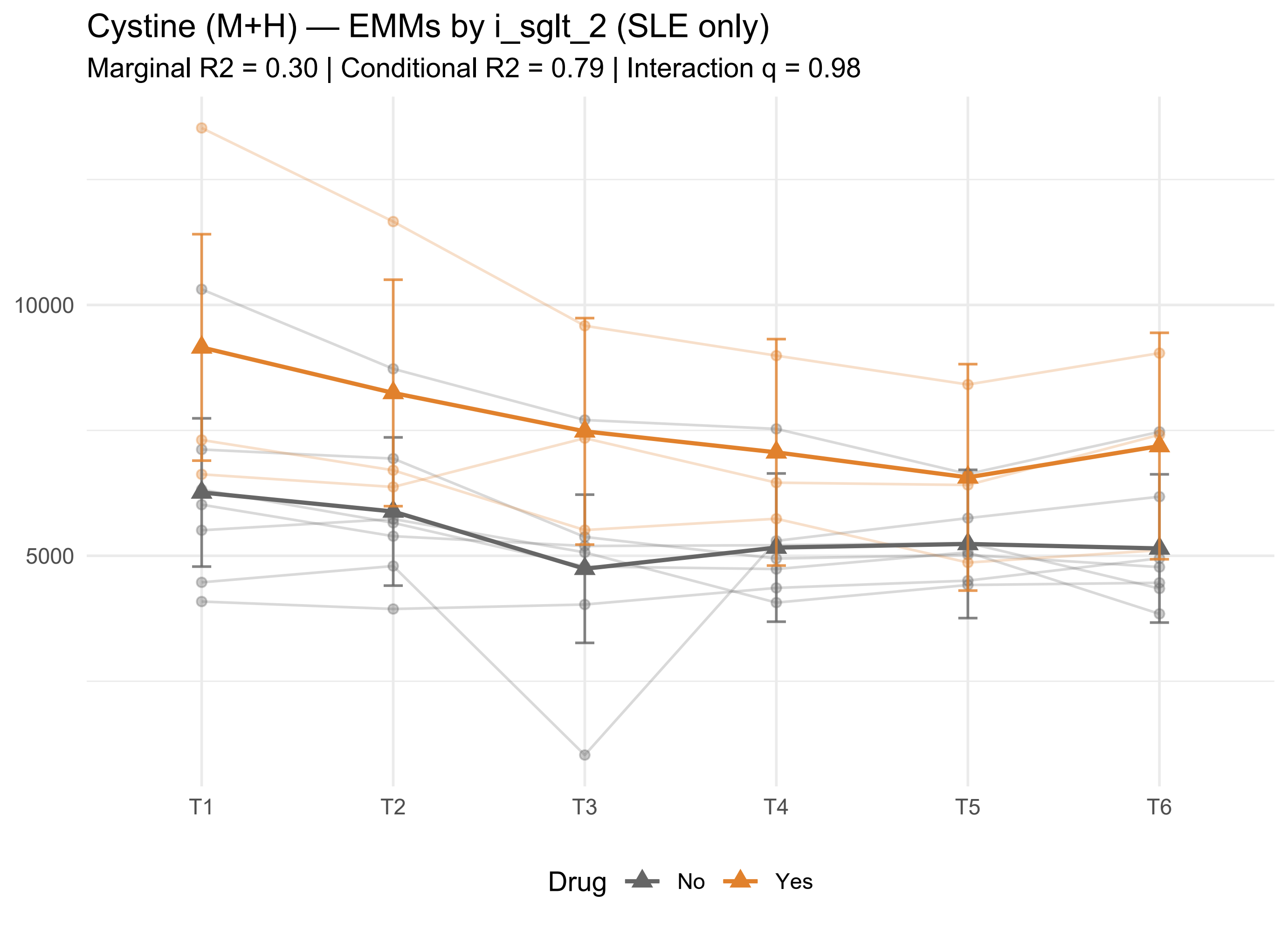
Cystine (M+H) — EMMs by i_sglt_2 (SLE only)
Marginal R2 = 0.30 | Conditional R2 = 0.79 | Interaction q = 0.98
10000
5000
T1
T2
T3
T4
T5
T6
Drug
No
Yes

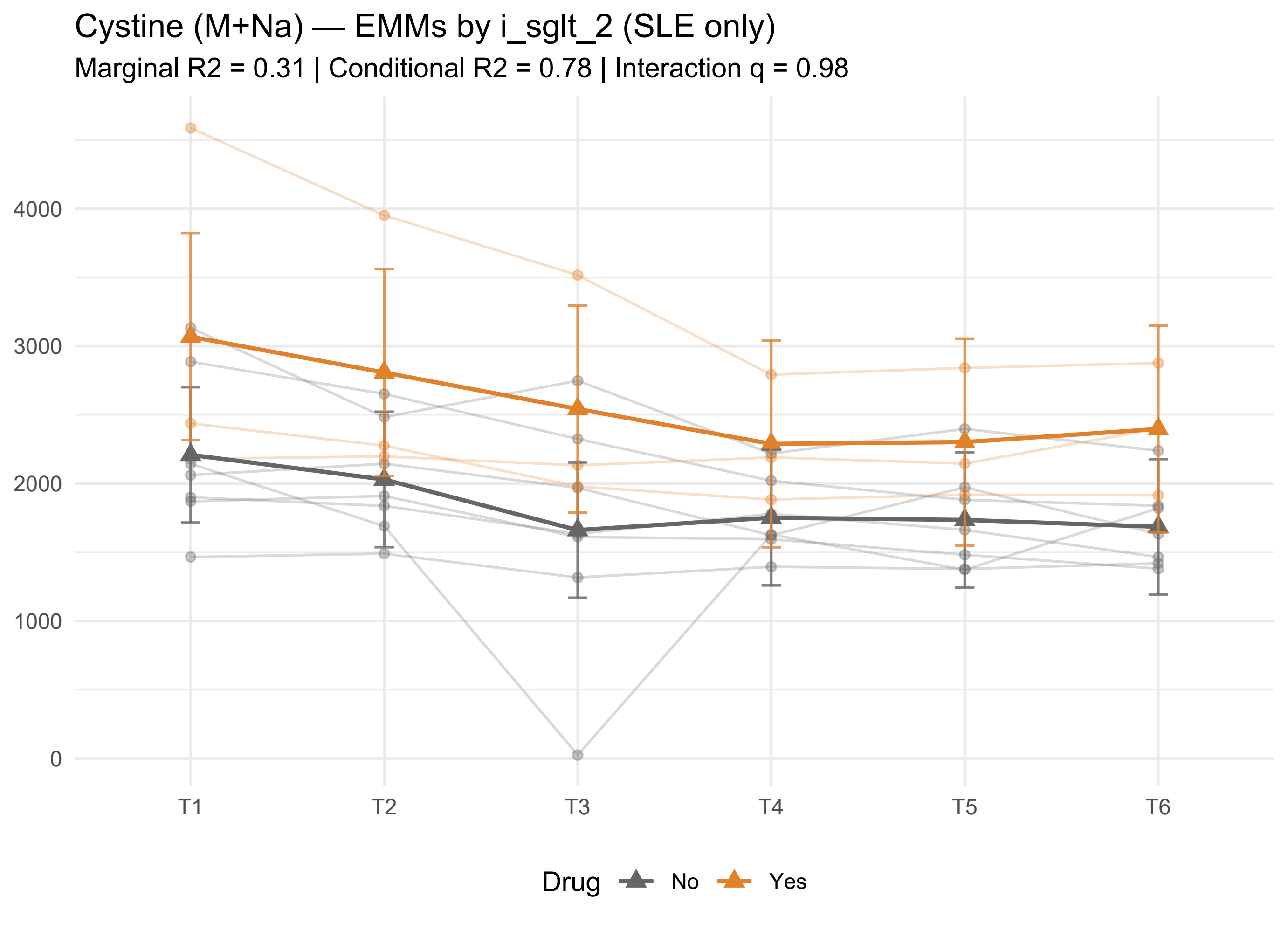

Cystine (M+Na) — EMMs by i_sglt_2 (SLE only)
Marginal R2 = 0.31 | Conditional R2 = 0.78 | Interaction q = 0.98
4000
3000
2000
1000
0
T1
T2
T3
T4
T5
T6
Drug
No
Yes

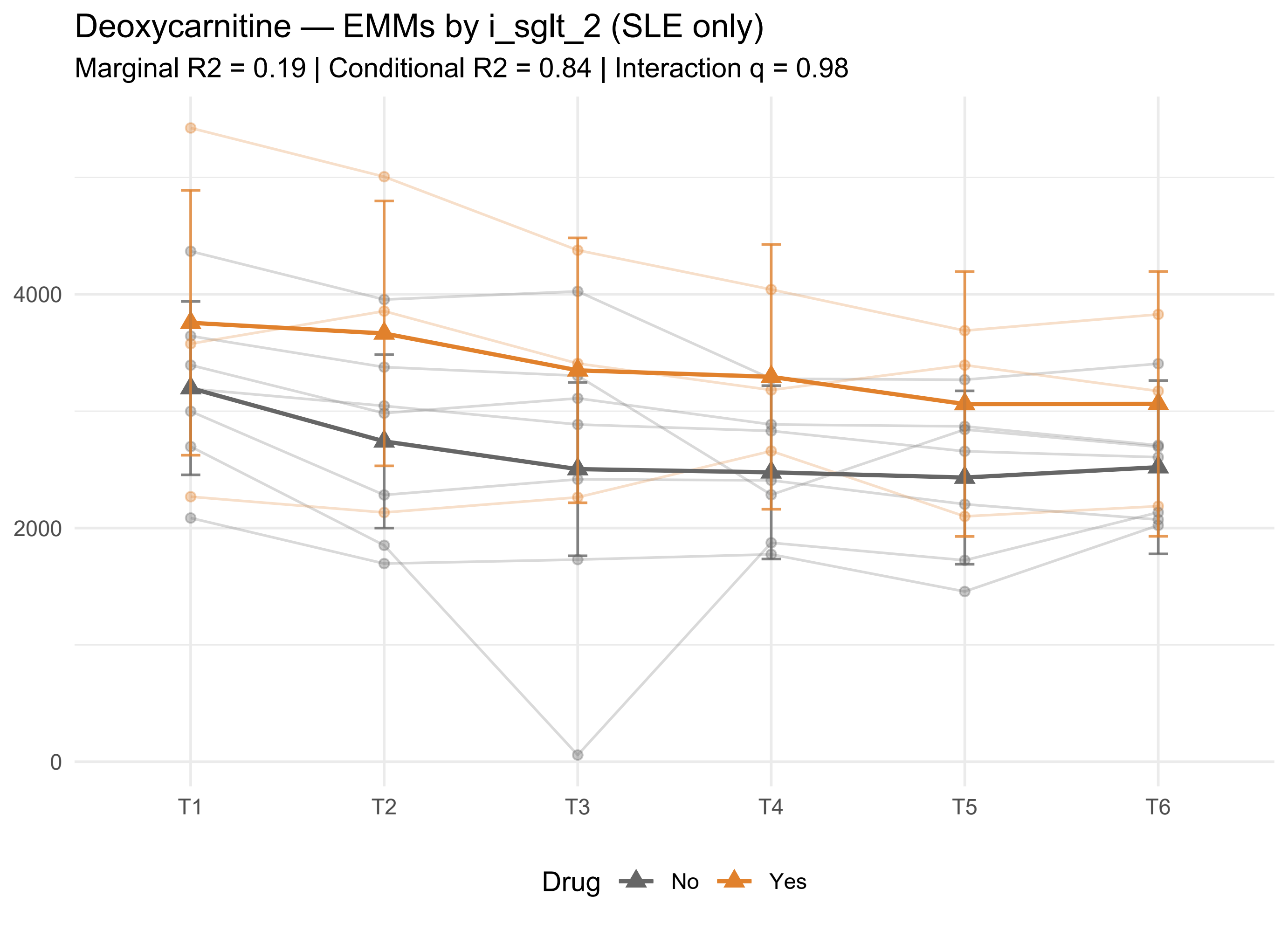
Deoxycarnitine — EMMs by i_sglt_2 (SLE only)
Marginal R2 = 0.19 | Conditional R2 = 0.84 | Interaction q = 0.98
4000
2000
0
T1
T2
T3
T4
T5
T6
Drug
No
Yes

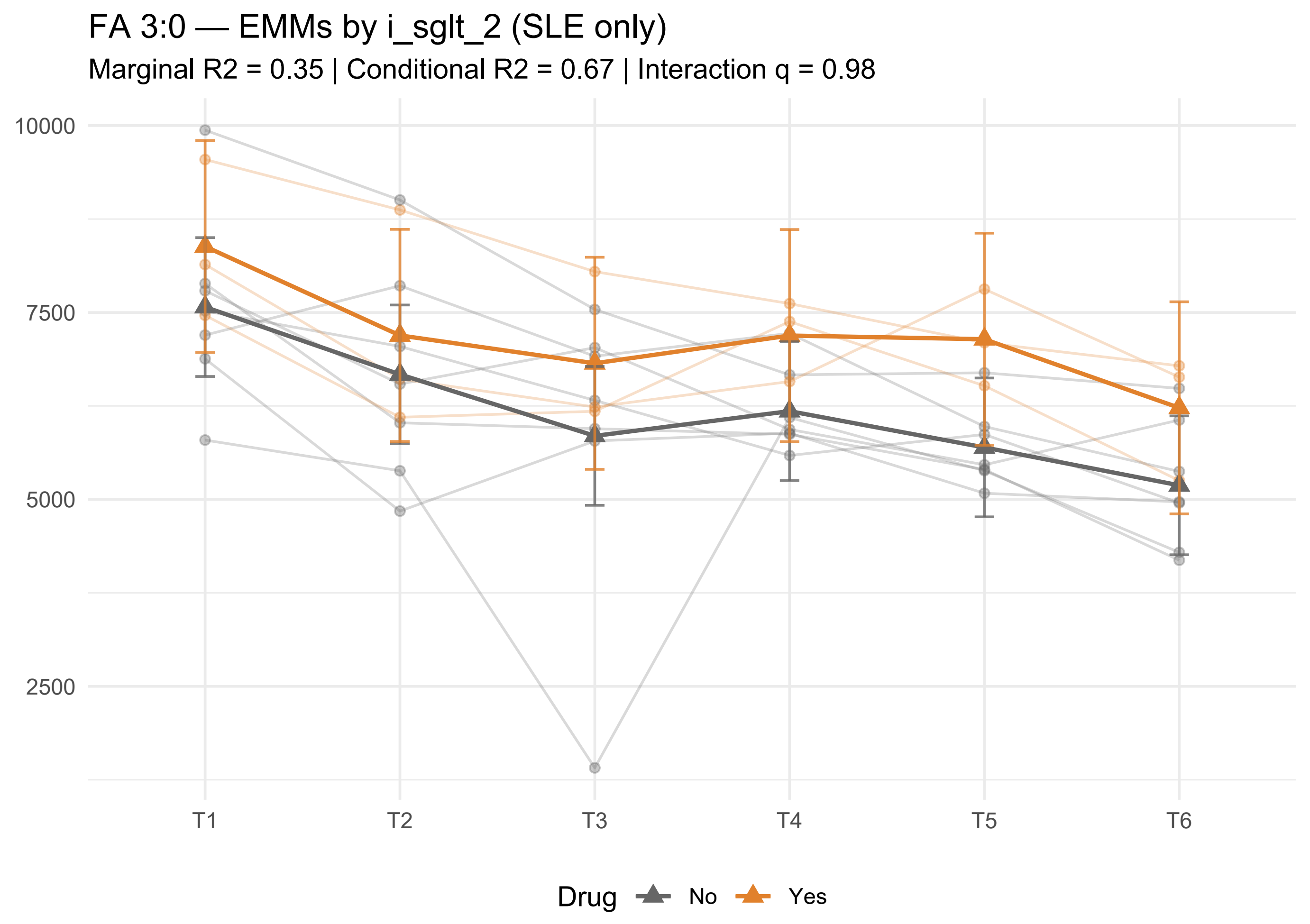
FA 3:0 — EMMs by i_sglt_2 (SLE only)
Marginal R2 = 0.35 | Conditional R2 = 0.67 | Interaction q = 0.98
10000
7500
5000
2500
T1
T2
T3
T4
T5
T6
Drug
No
Yes

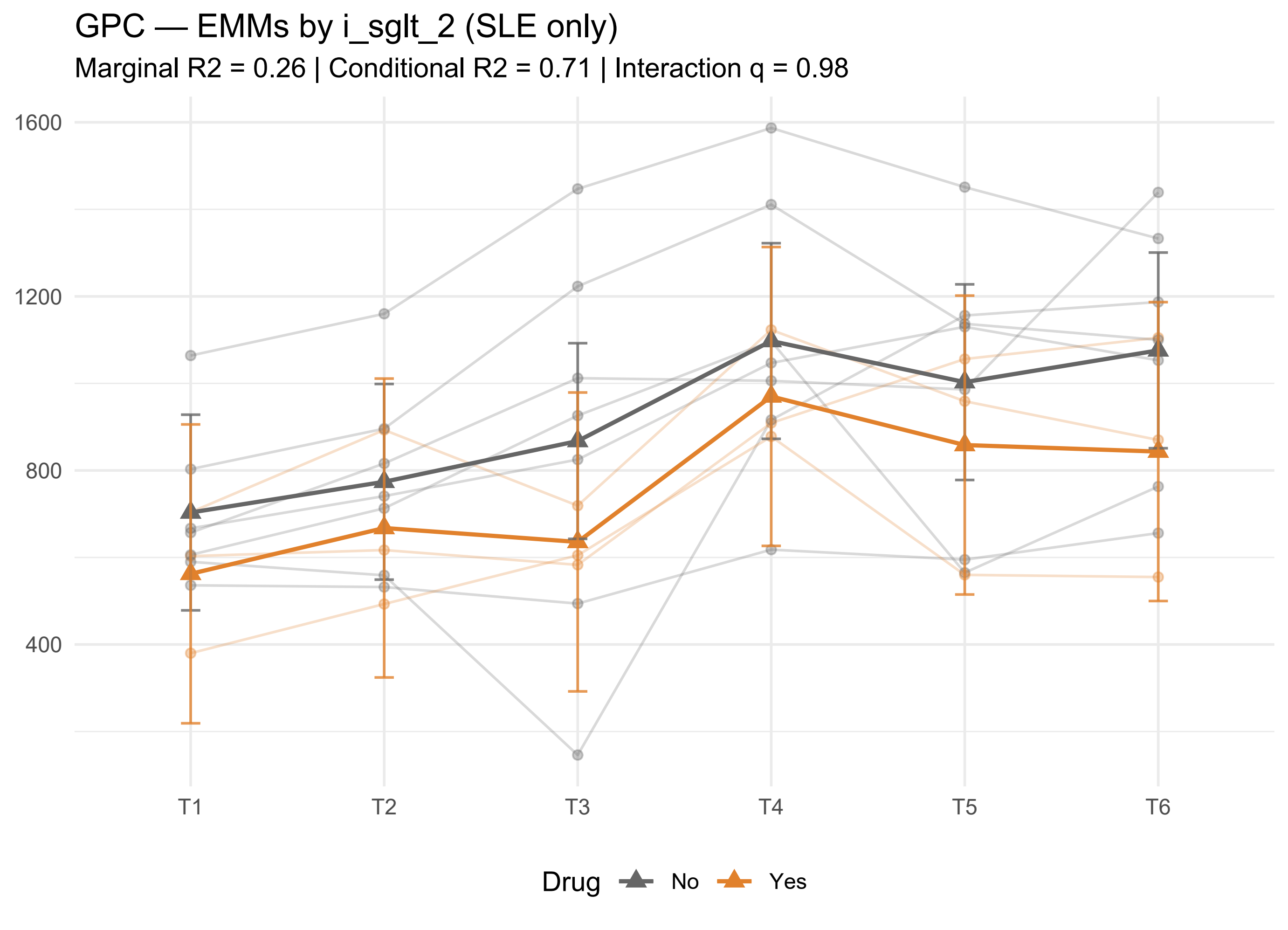

GPC — EMMs by i_sglt_2 (SLE only)
Marginal R2 = 0.26 | Conditional R2 = 0.71 | Interaction q = 0.98
1600
1200
800
400
T1
T2
T3
T4
T5
T6
Drug
No
Yes

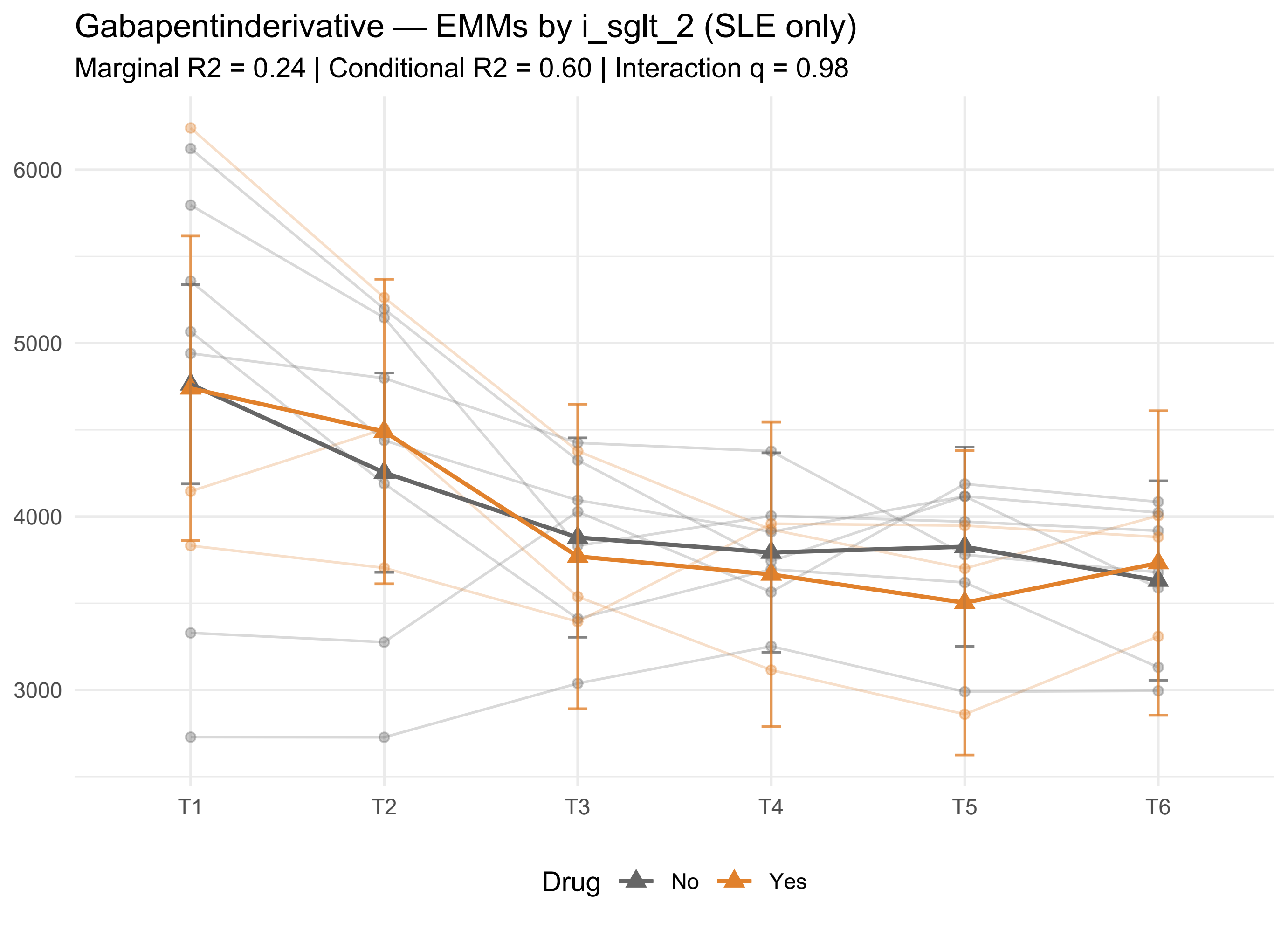
Gabapentinderivative — EMMs by i_sglt_2 (SLE only)
Marginal R2 = 0.24 | Conditional R2 = 0.60 | Interaction q = 0.98
6000
5000
4000
3000
T1
T2
T3
T4
T5
T6
Drug
No
Yes

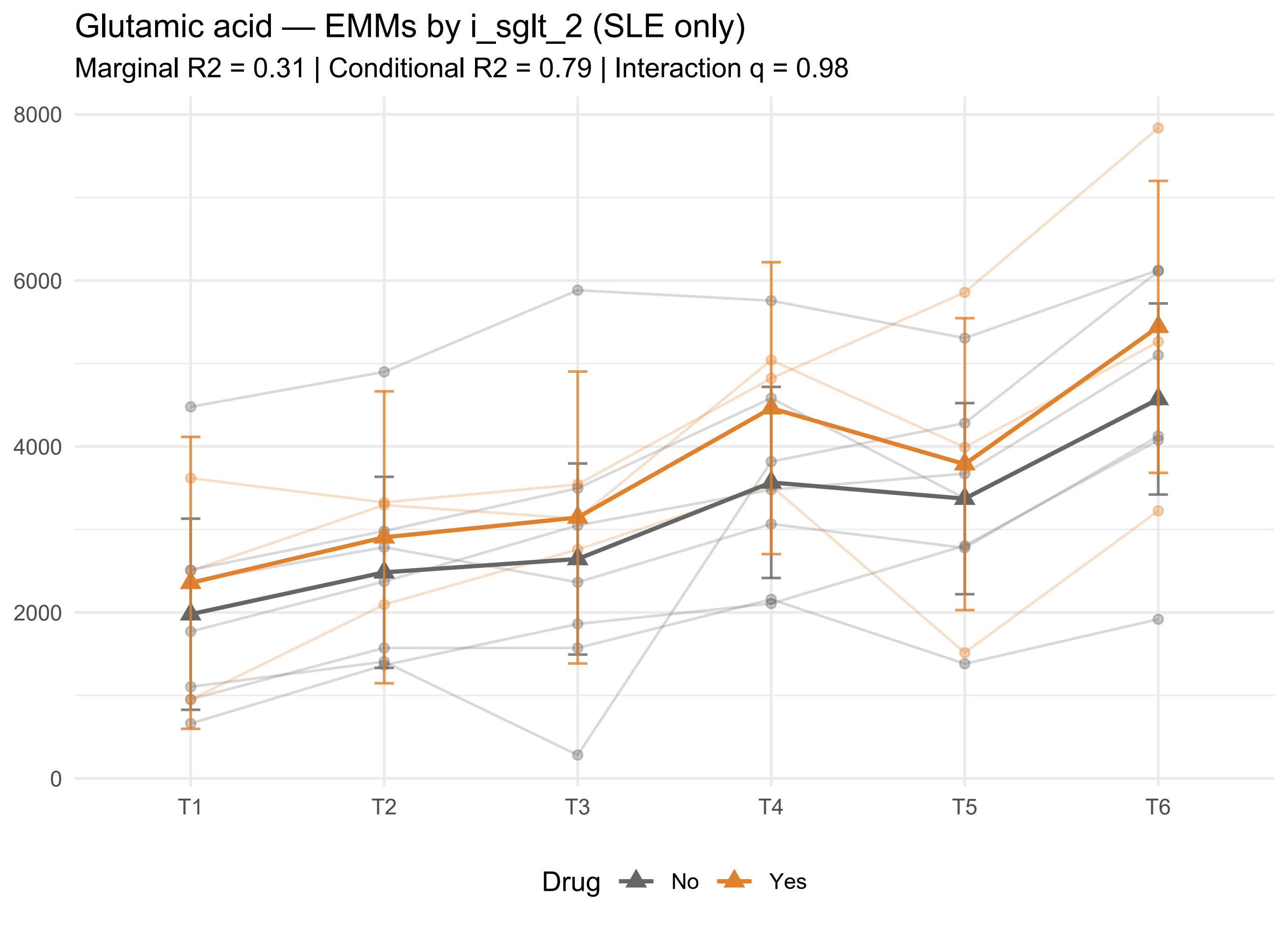

Glutamic acid — EMMs by i_sglt_2 (SLE only)
Marginal R2 = 0.31 | Conditional R2 = 0.79 | Interaction q = 0.98
8000
6000
4000
2000
0
T1
T2
T3
T4
T5
T6
Drug
No
Yes

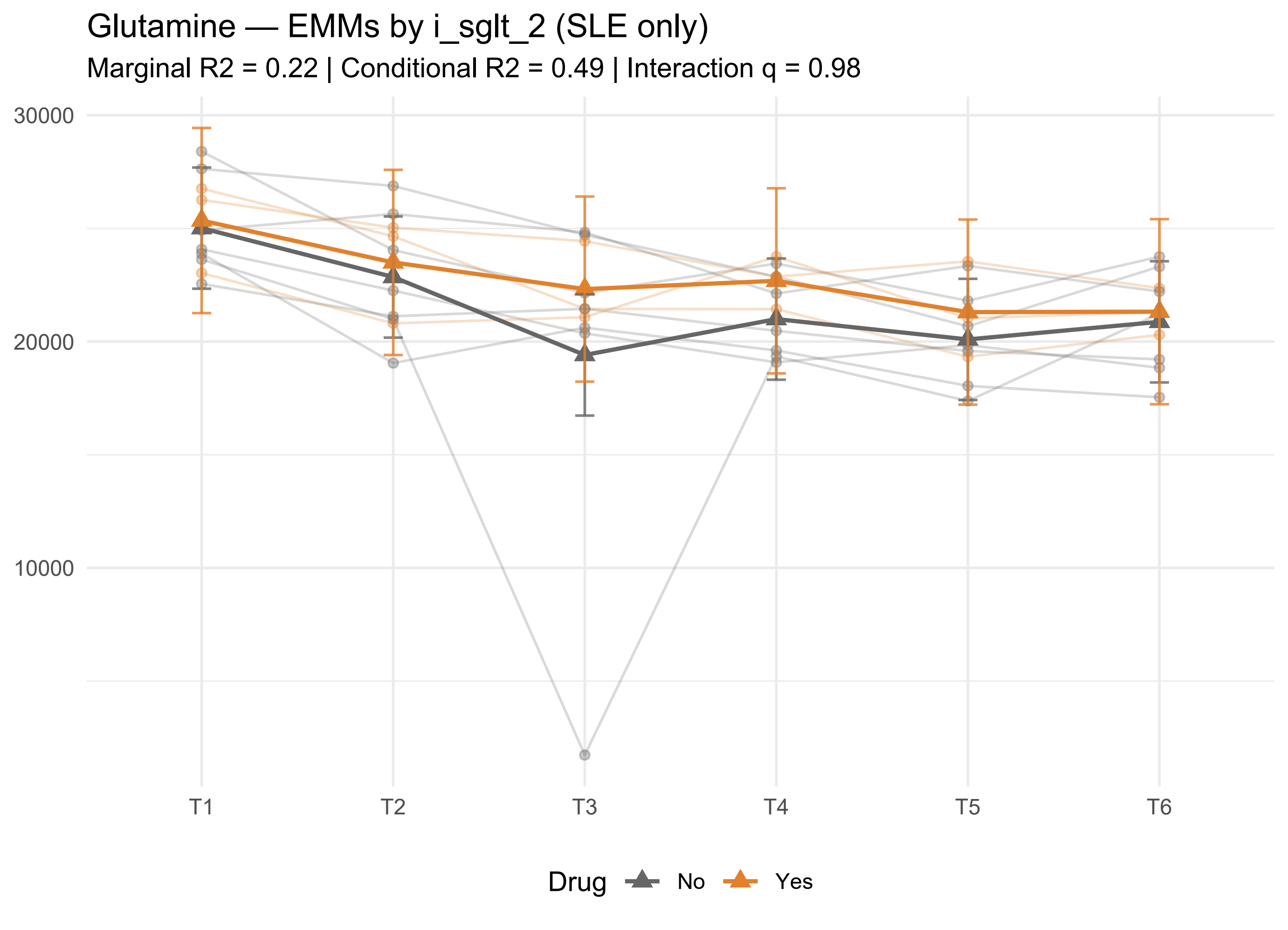

Glutamine — EMMs by i_sglt_2 (SLE only)
Marginal R2 = 0.22 | Conditional R2 = 0.49 | Interaction q = 0.98
30000
20000
10000
T1
T2
T3
T4
T5
T6
Drug
No
Yes

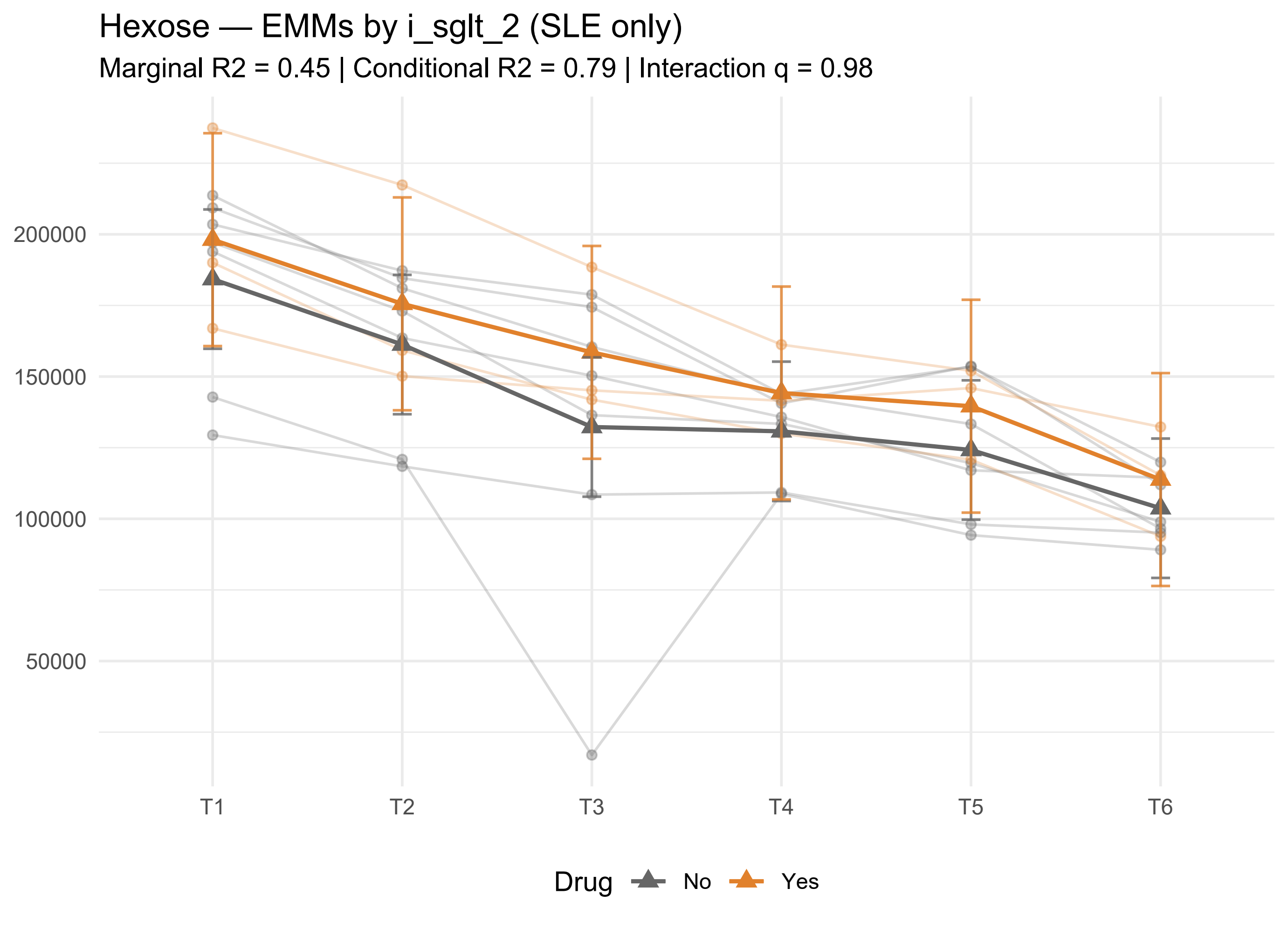

Hexose — EMMs by i_sglt_2 (SLE only)
Marginal R2 = 0.45 | Conditional R2 = 0.79 | Interaction q = 0.98
200000
150000
100000
50000
T1
T2
T3
T4
T5
T6
Drug
No
Yes

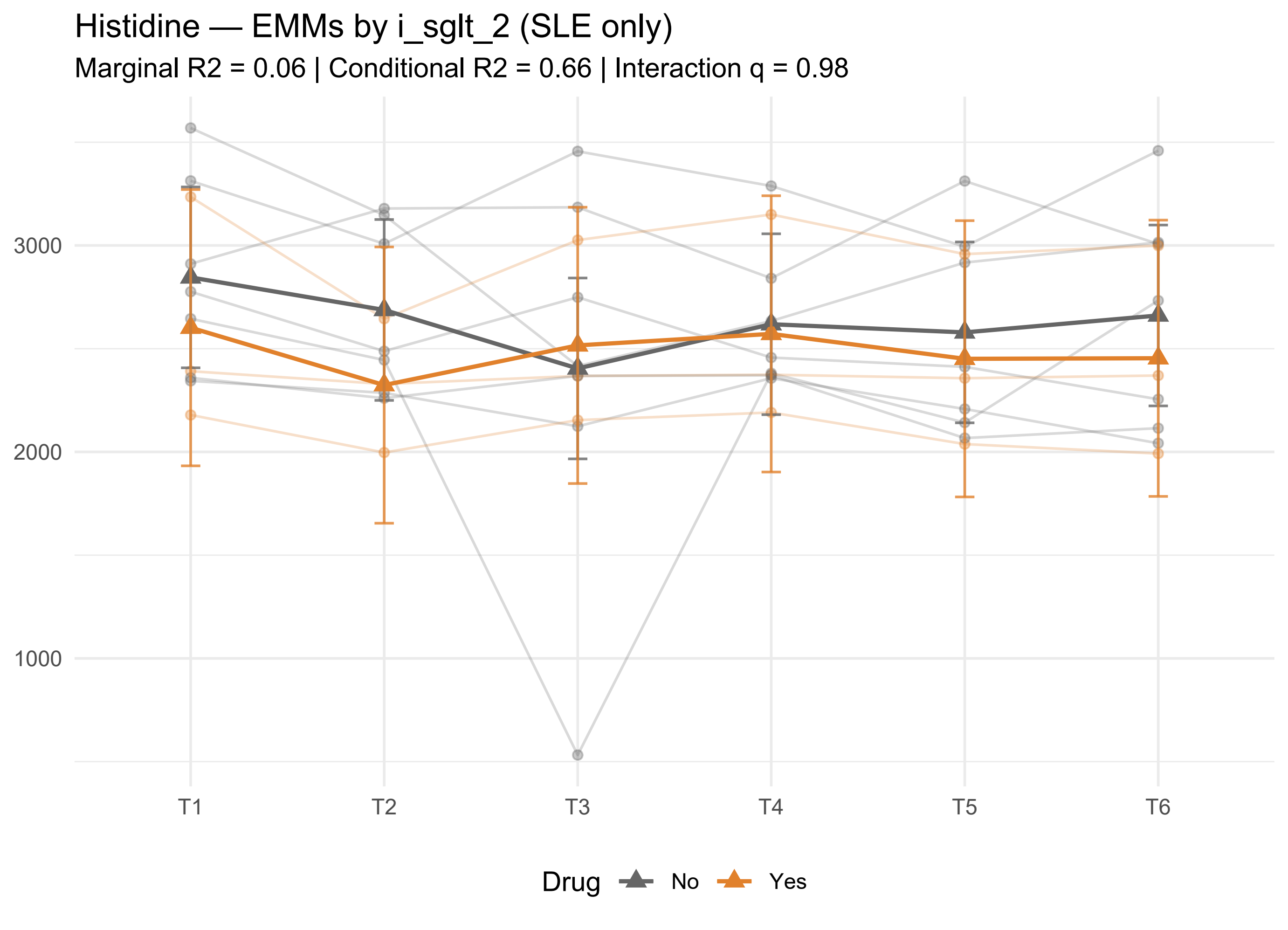

Histidine — EMMs by i_sglt_2 (SLE only)
Marginal R2 = 0.06 | Conditional R2 = 0.66 | Interaction q = 0.98
3000
2000
1000
T1
T2
T3
T4
T5
T6
Drug
No
Yes

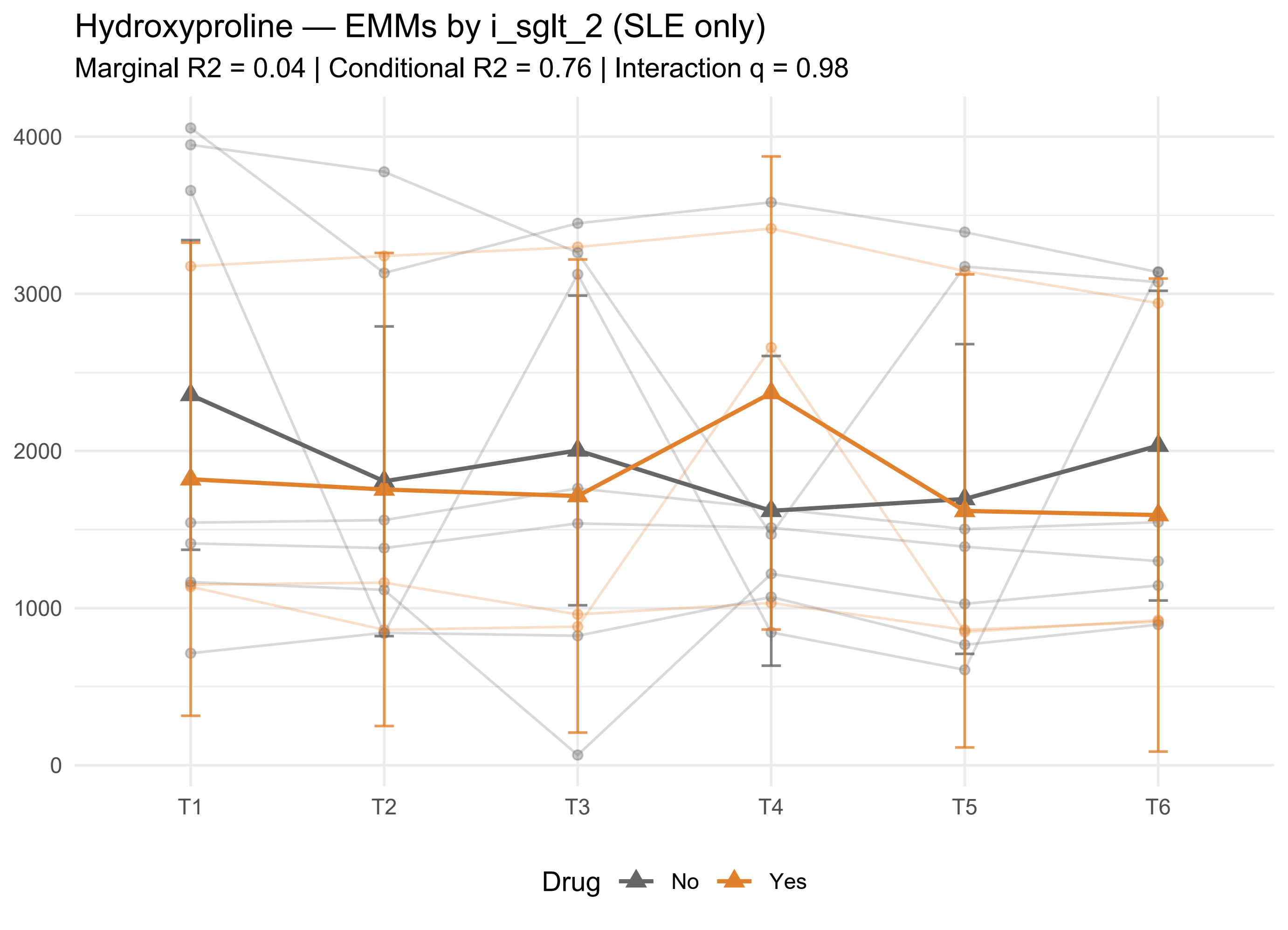
Hydroxyproline — EMMs by i_sglt_2 (SLE only)
Marginal R2 = 0.04 | Conditional R2 = 0.76 | Interaction q = 0.98
4000
3000
2000
1000
0
T1
T2
T3
T4
T5
T6
Drug
No
Yes

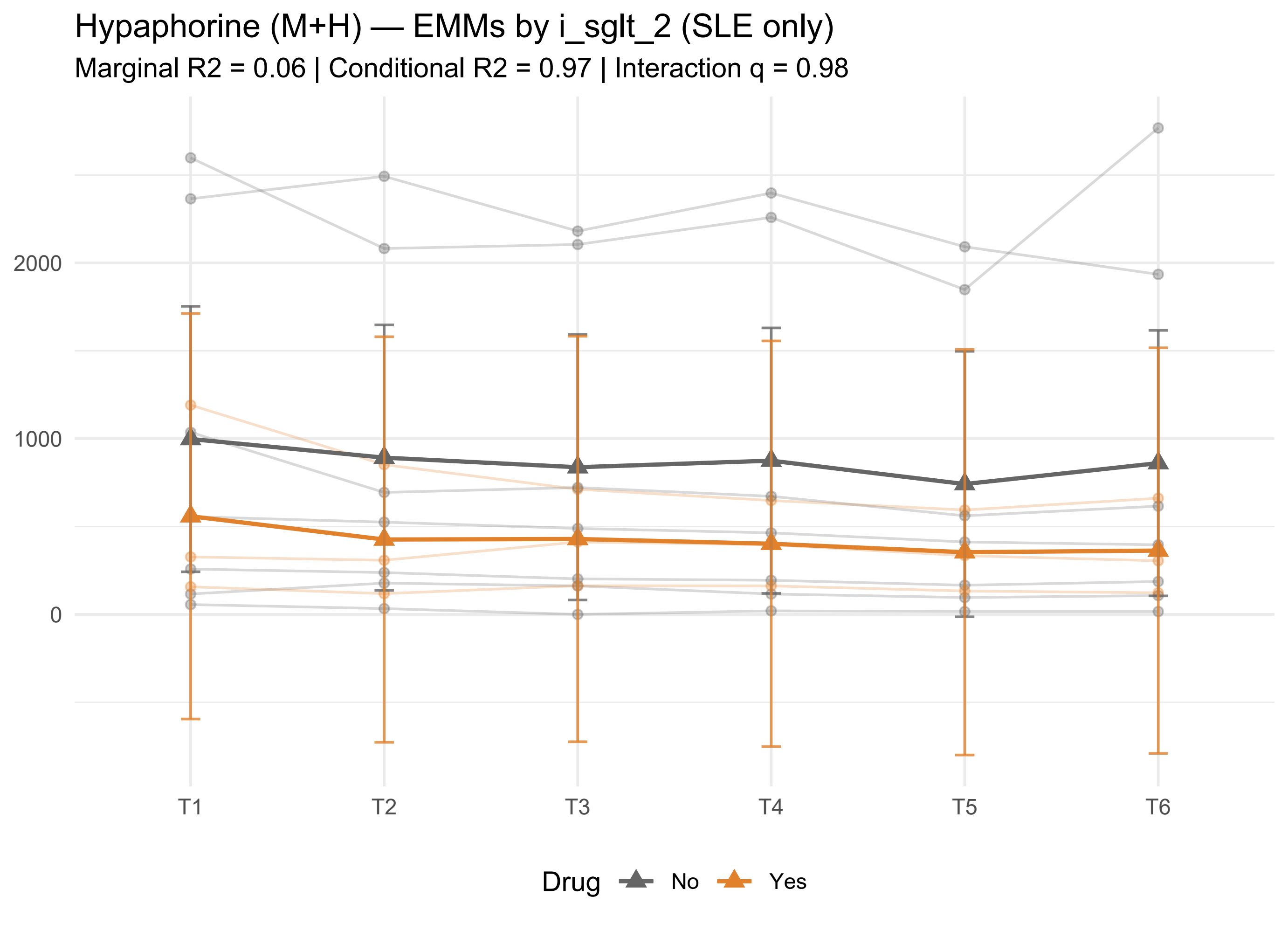

Hypaphorine (M+H) — EMMs by i_sglt_2 (SLE only)
Marginal R2 = 0.06 | Conditional R2 = 0.97 | Interaction q = 0.98
2000
1000
0
T1
T2
T3
T4
T5
T6
Drug
No
Yes

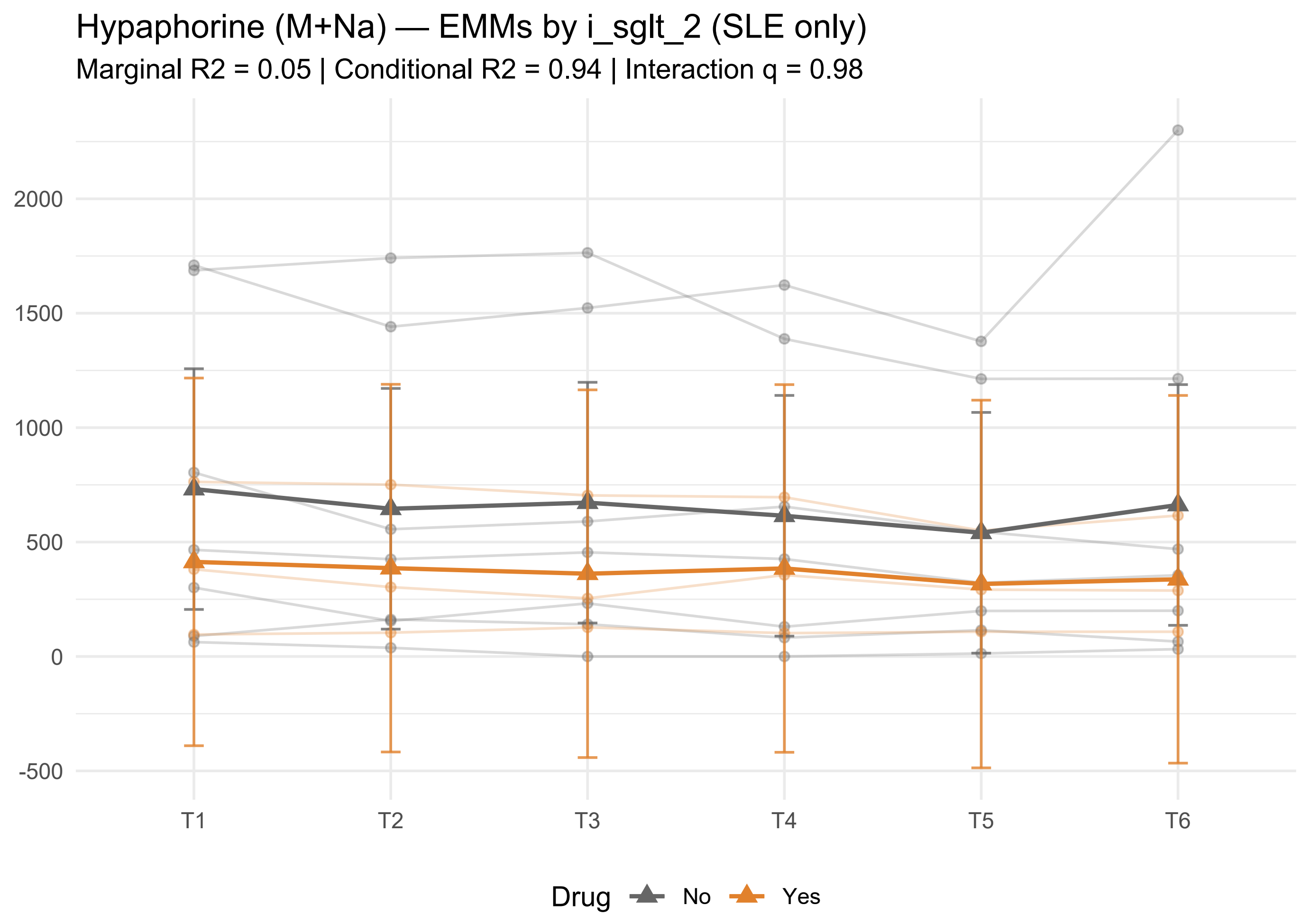
Hypaphorine (M+Na) — EMMs by i_sglt_2 (SLE only)
Marginal R2 = 0.05 | Conditional R2 = 0.94 | Interaction q = 0.98
2000
1500
1000
500
0
-500
T1
T2
T3
T4
T5
T6
Drug
No
Yes

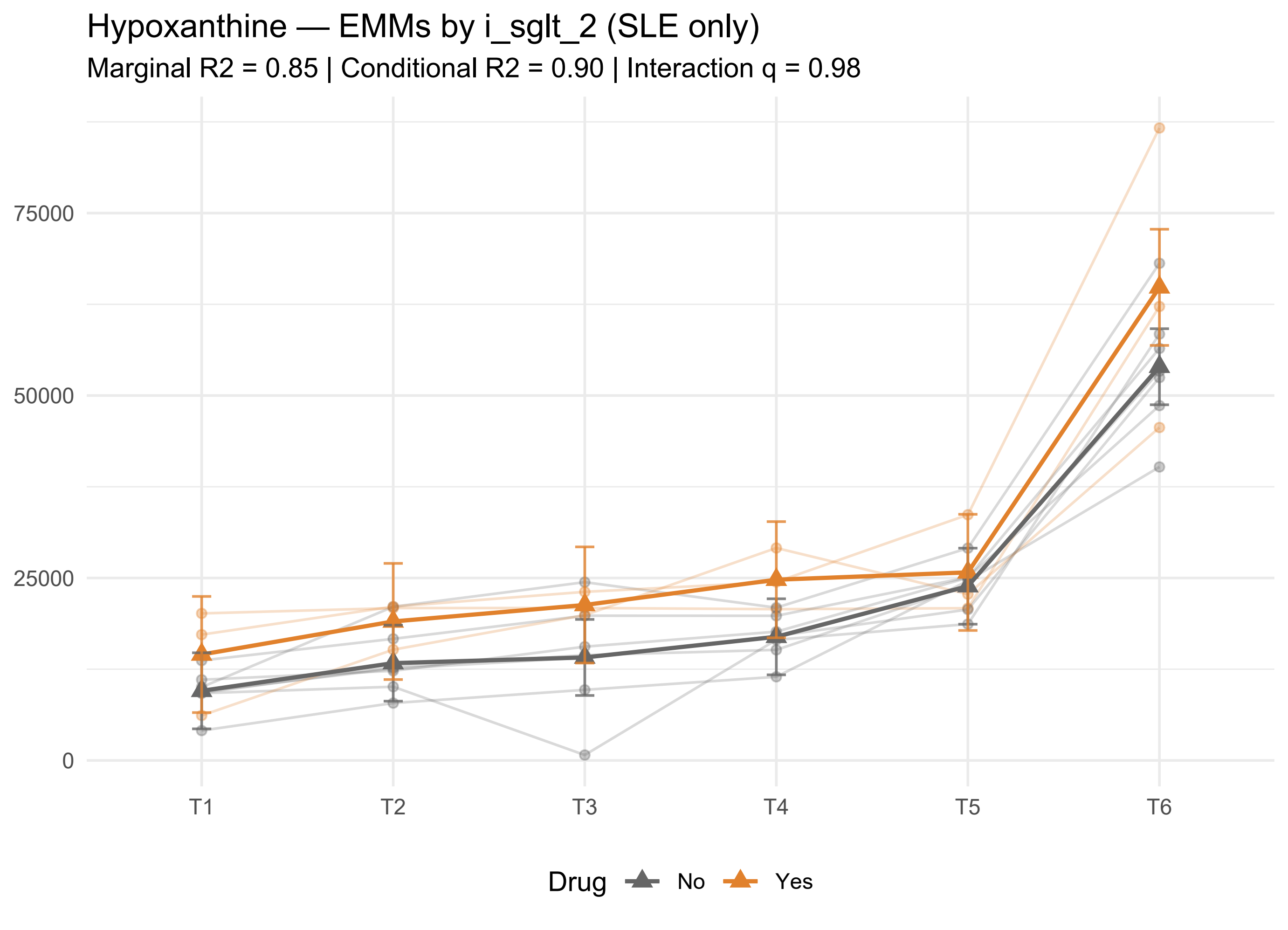
Hypoxanthine — EMMs by i_sglt_2 (SLE only)
Marginal R2 = 0.85 | Conditional R2 = 0.90 | Interaction q = 0.98
75000
50000
25000
0
T1
T2
T3
T4
T5
T6
Drug
No
Yes

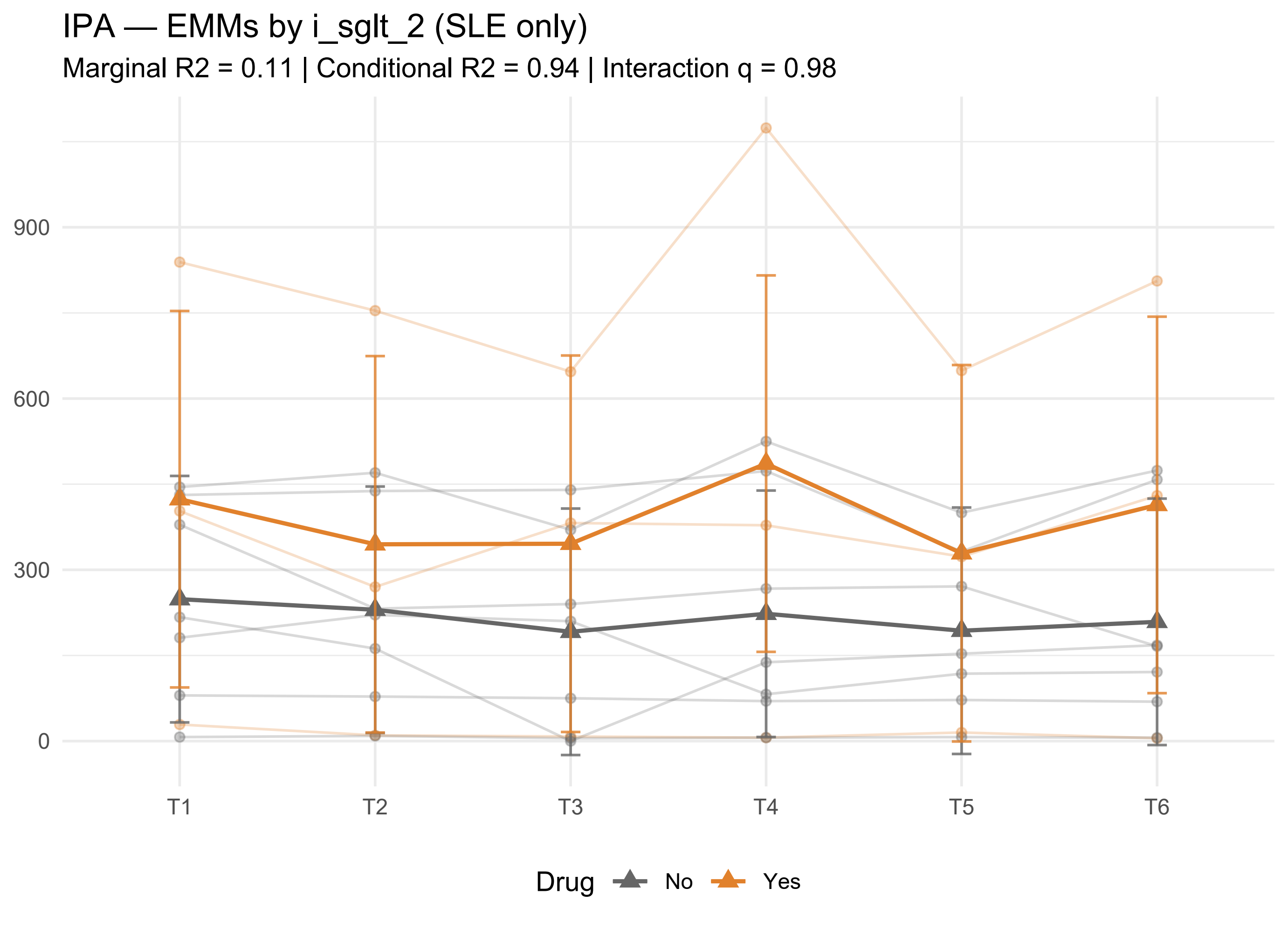

IPA — EMMs by i_sglt_2 (SLE only)
Marginal R2 = 0.11 | Conditional R2 = 0.94 | Interaction q = 0.98
900
600
300
0
T1
T2
T3
T4
T5
T6
Drug
No
Yes

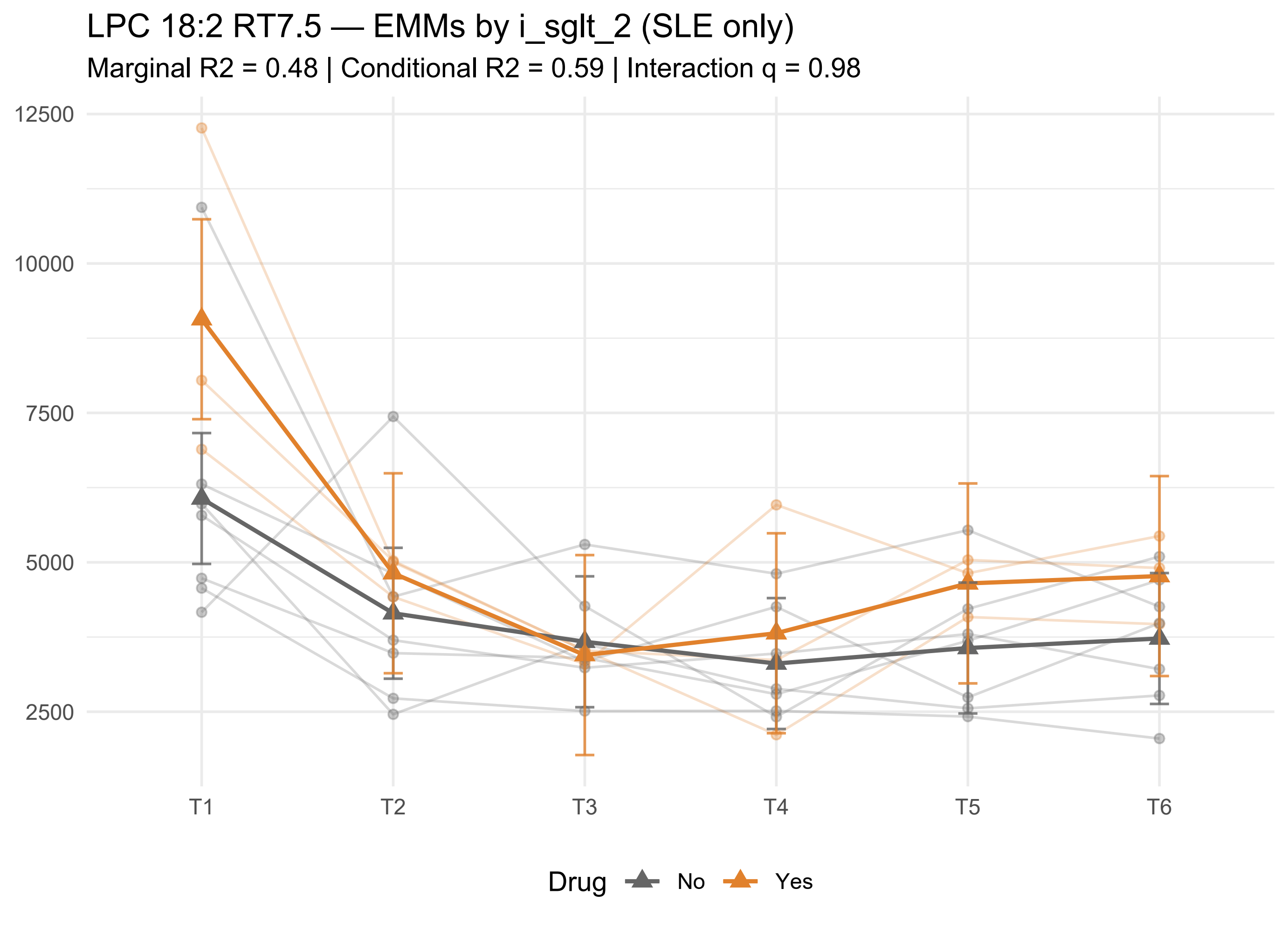

LPC 18:2 RT7.5 — EMMs by i_sglt_2 (SLE only)
Marginal R2 = 0.48 | Conditional R2 = 0.59 | Interaction q = 0.98
12500
10000
7500
5000
2500
T1
T2
T3
T4
T5
T6
Drug
No
Yes

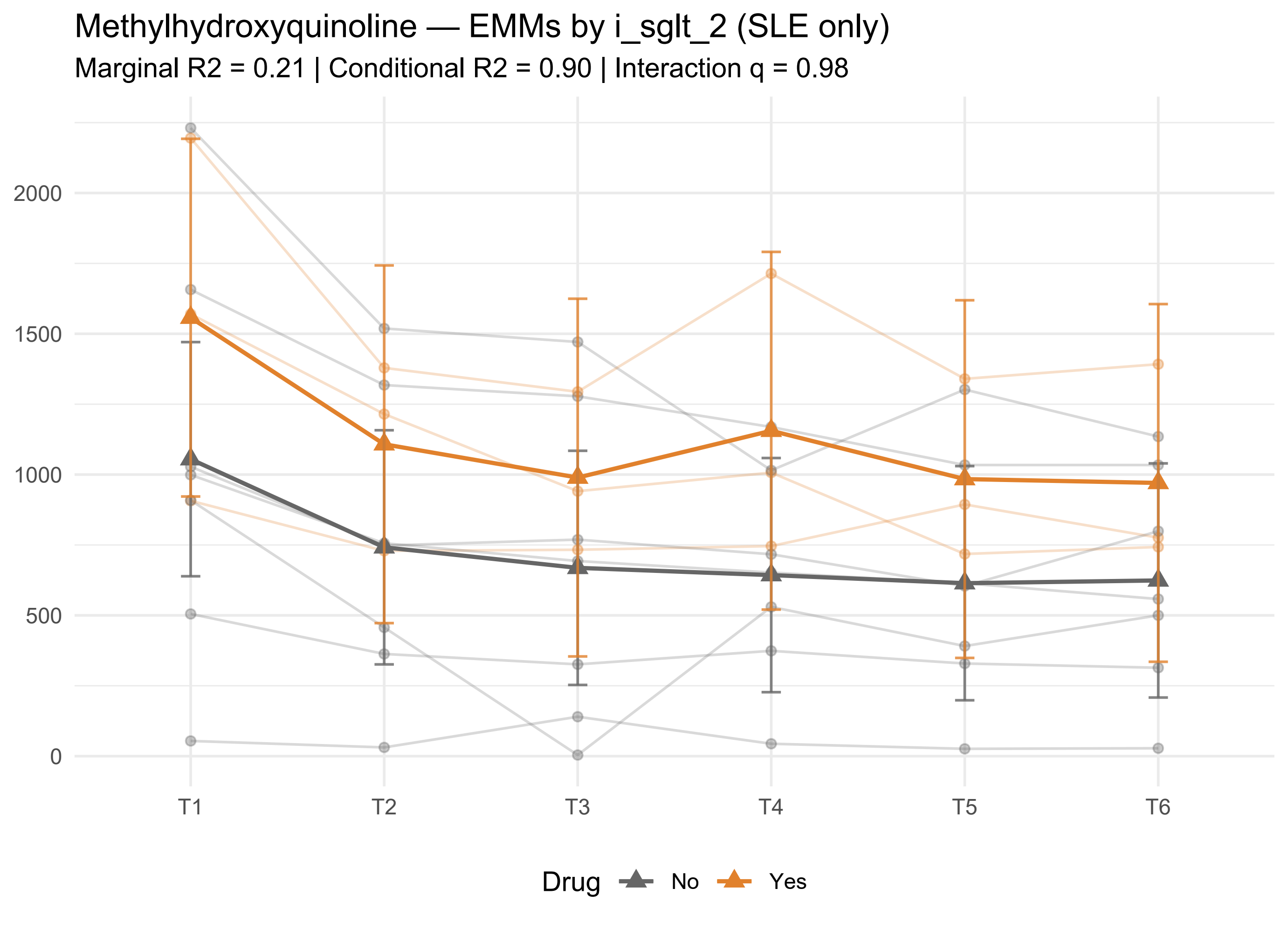

Methylhydroxyquinoline — EMMs by i_sglt_2 (SLE only)
Marginal R2 = 0.21 | Conditional R2 = 0.90 | Interaction q = 0.98
2000
1500
1000
500
0
T1
T2
T3
T4
T5
T6
Drug
No
Yes

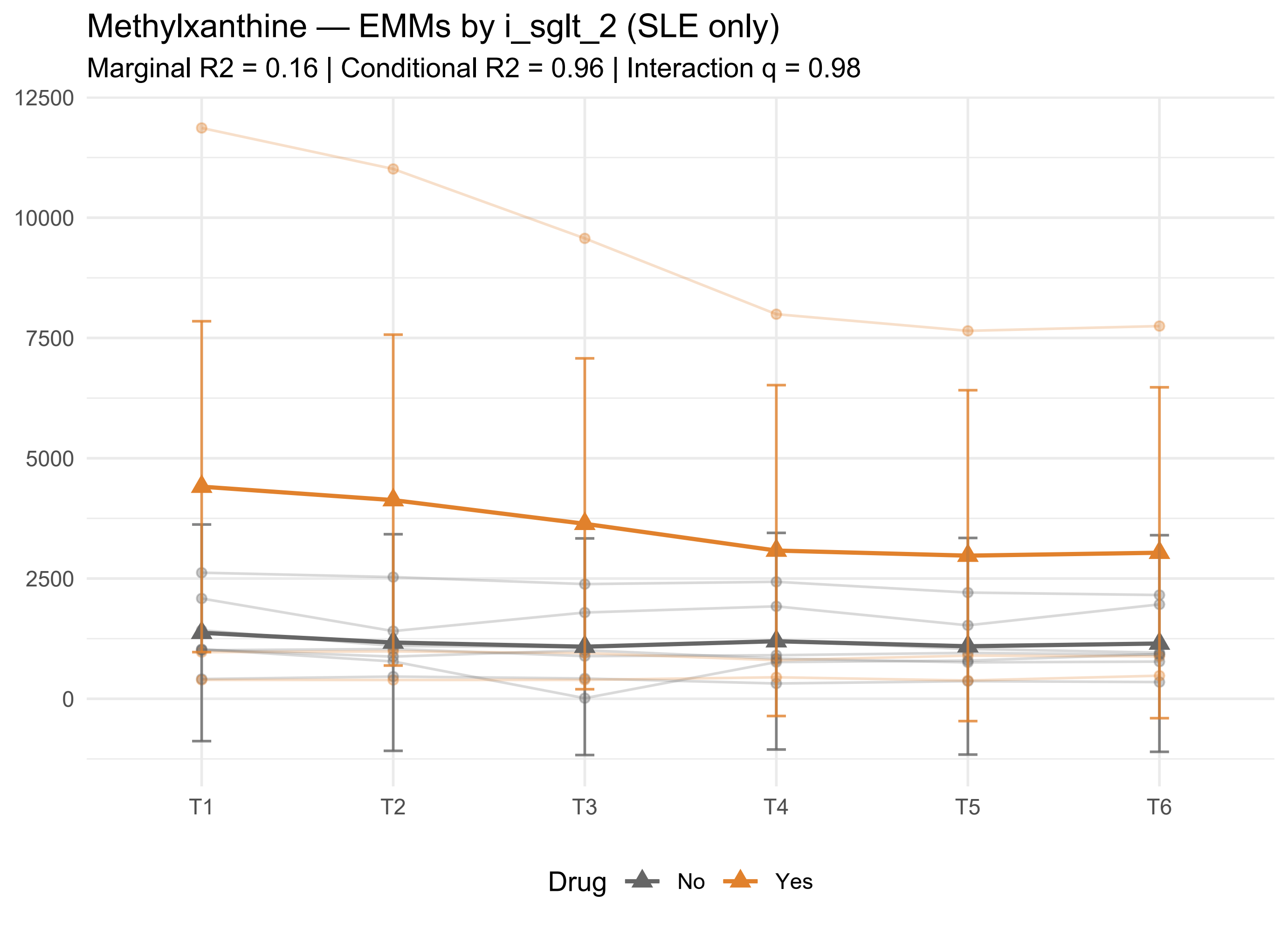

Methylxanthine — EMMs by i_sglt_2 (SLE only)
Marginal R2 = 0.16 | Conditional R2 = 0.96 | Interaction q = 0.98
12500
10000
7500
5000
2500
0
T1
T2
T3
T4
T5
T6
Drug
No
Yes

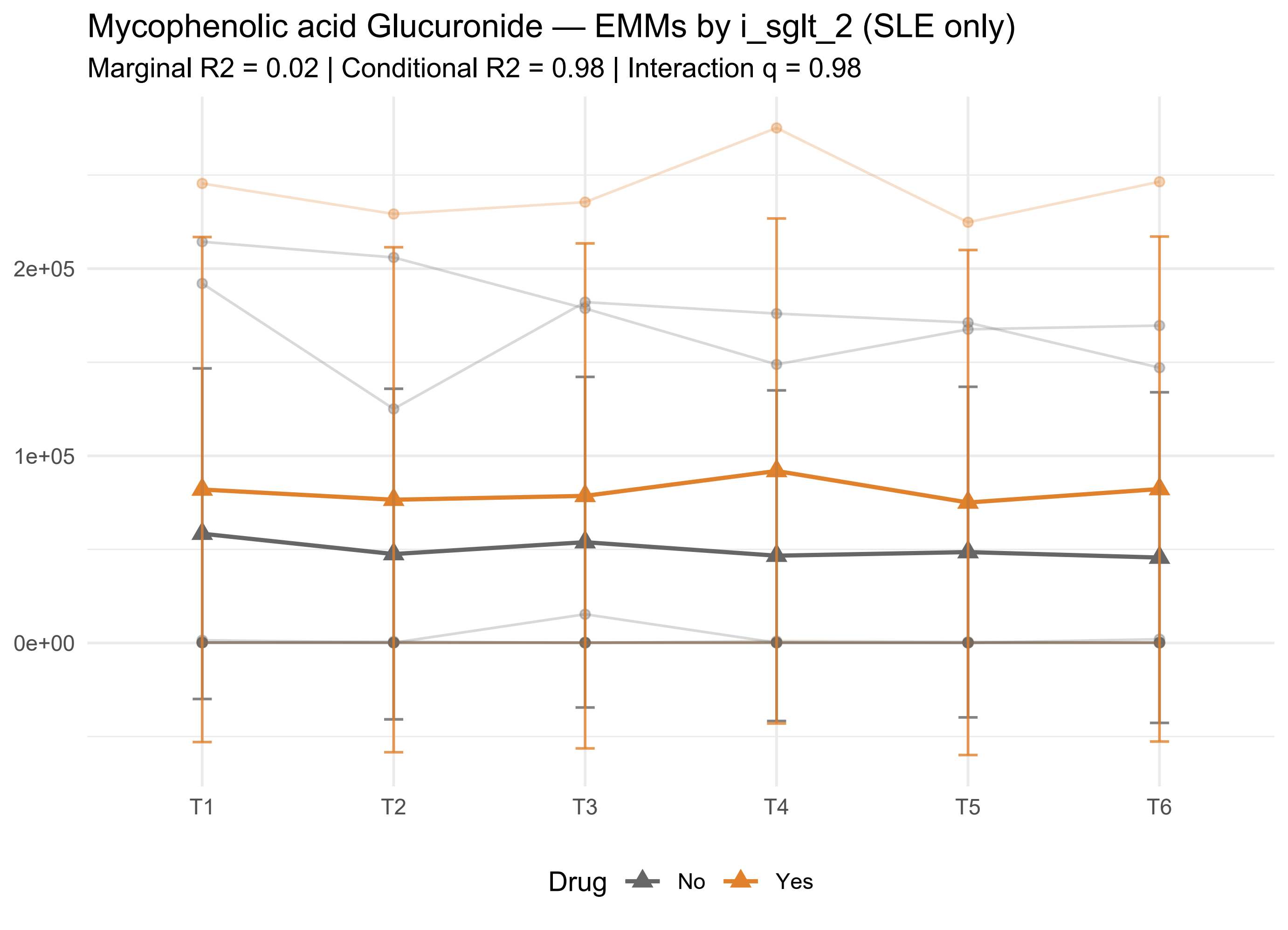
Mycophenolic acid Glucuronide — EMMs by i_sglt_2 (SLE only)
Marginal R2 = 0.02 | Conditional R2 = 0.98 | Interaction q = 0.98
2e+05
1e+05
0e+00
T1
T2
T3
T4
T5
T6
Drug
No
Yes

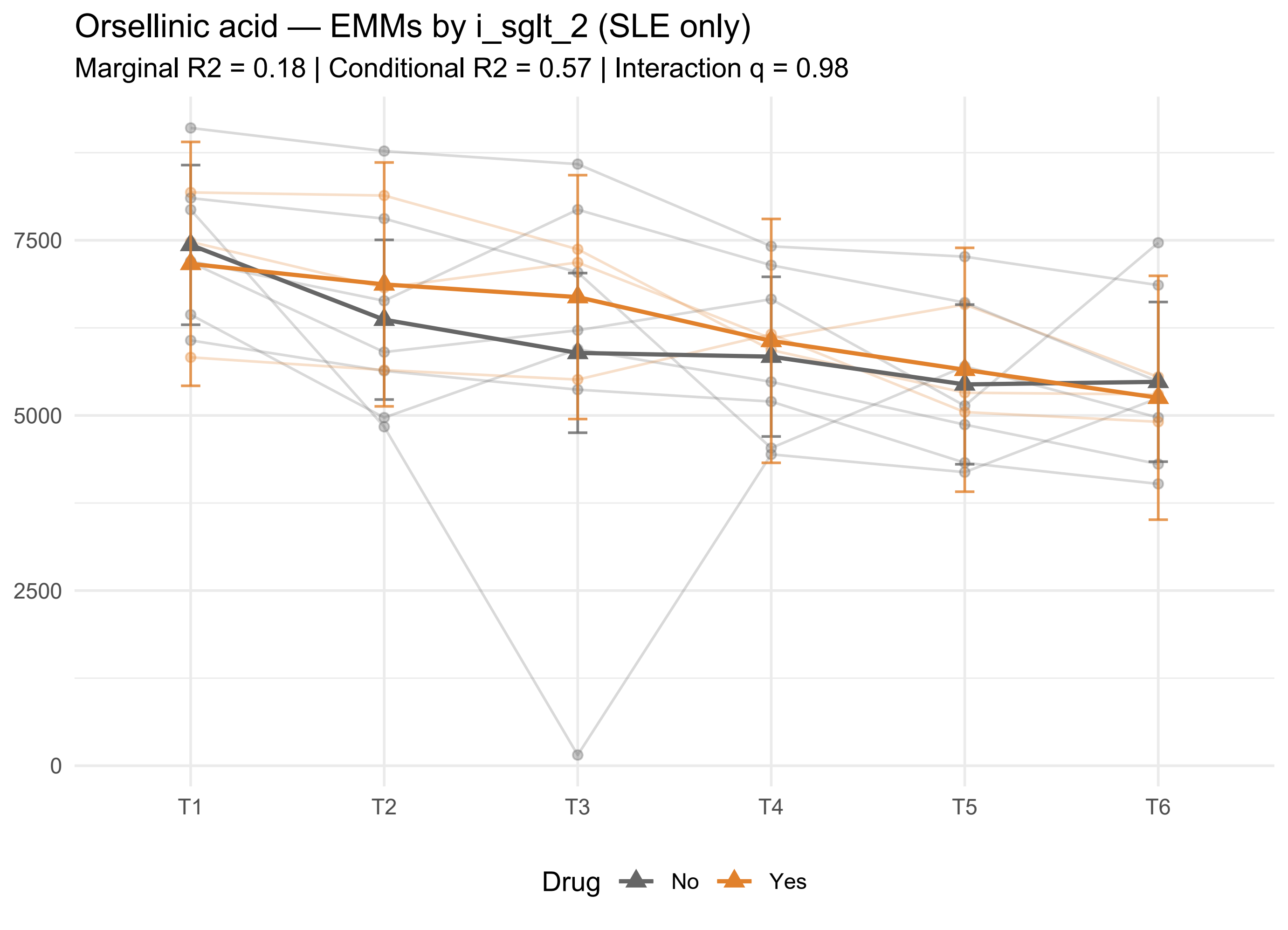

Orsellinic acid — EMMs by i_sglt_2 (SLE only)
Marginal R2 = 0.18 | Conditional R2 = 0.57 | Interaction q = 0.98
7500
5000
2500
0
T1
T2
T3
T4
T5
T6
Drug
No
Yes

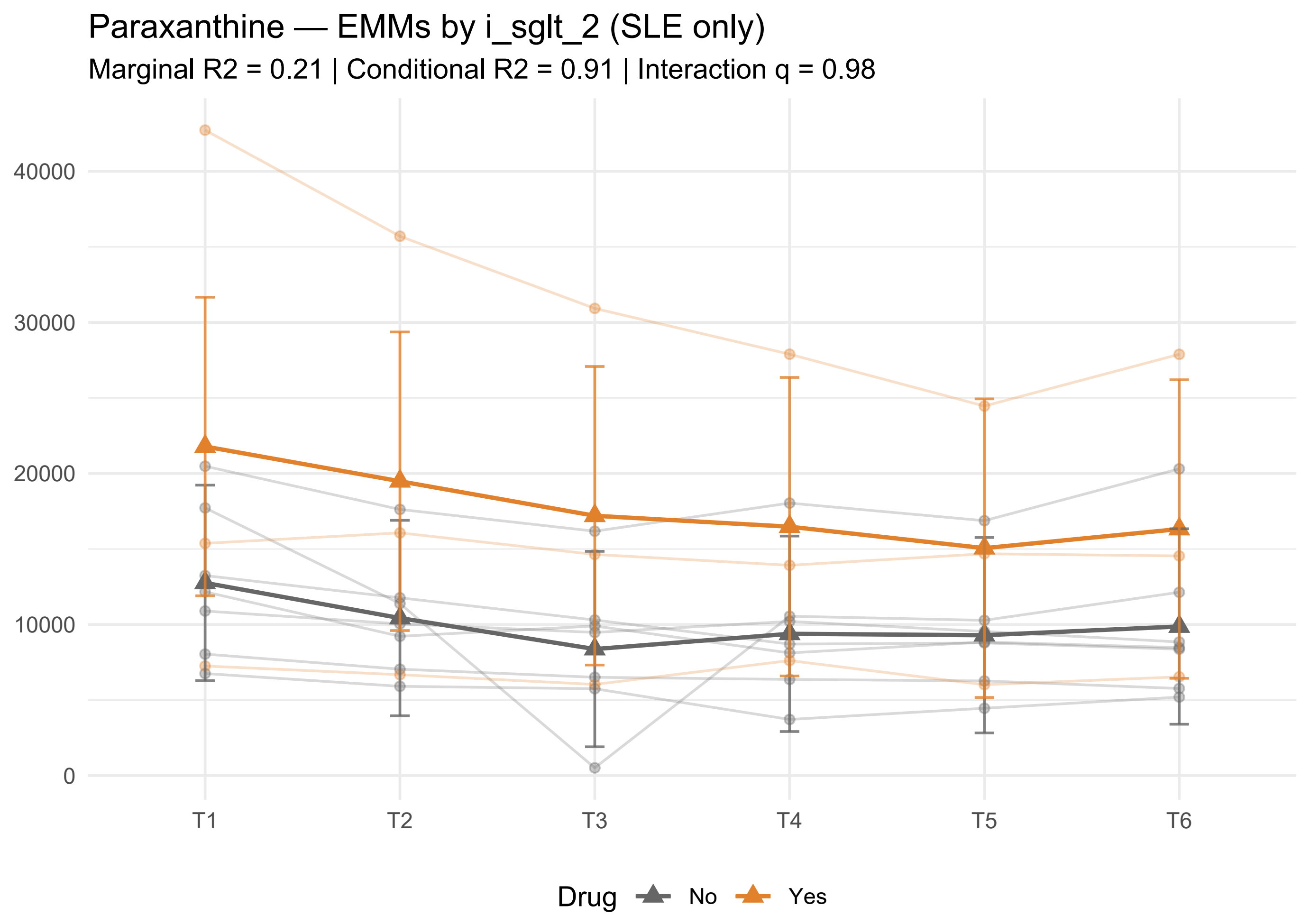

Paraxanthine — EMMs by i_sglt_2 (SLE only)
Marginal R2 = 0.21 | Conditional R2 = 0.91 | Interaction q = 0.98
40000
30000
20000
10000
0
T1
T2
T3
T4
T5
T6
Drug
No
Yes

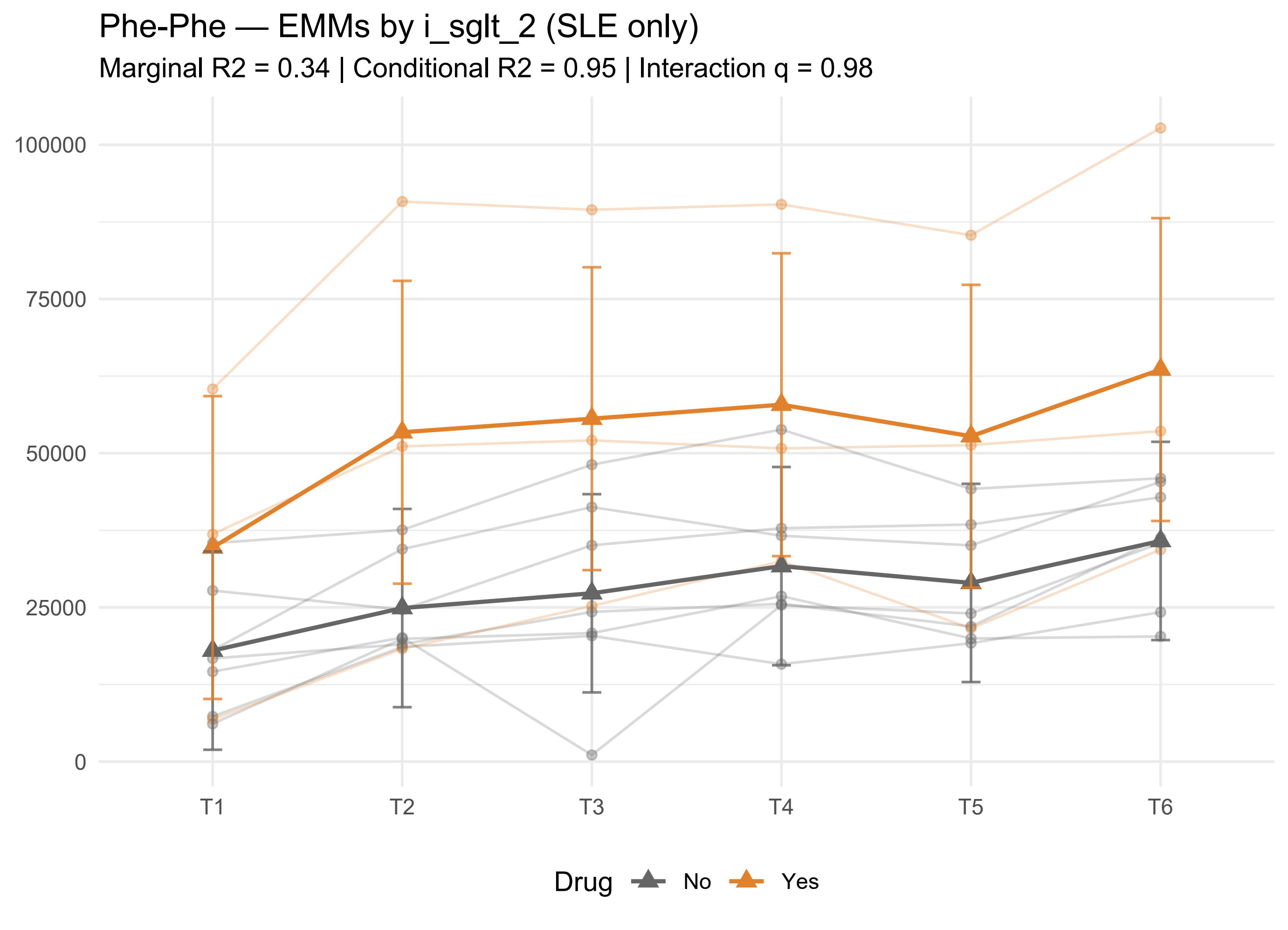

Phe-Phe — EMMs by i_sglt_2 (SLE only)
Marginal R2 = 0.34 | Conditional R2 = 0.95 | Interaction q = 0.98
100000
75000
50000
25000
0
T1
T2
T3
T4
T5
T6
Drug
No
Yes

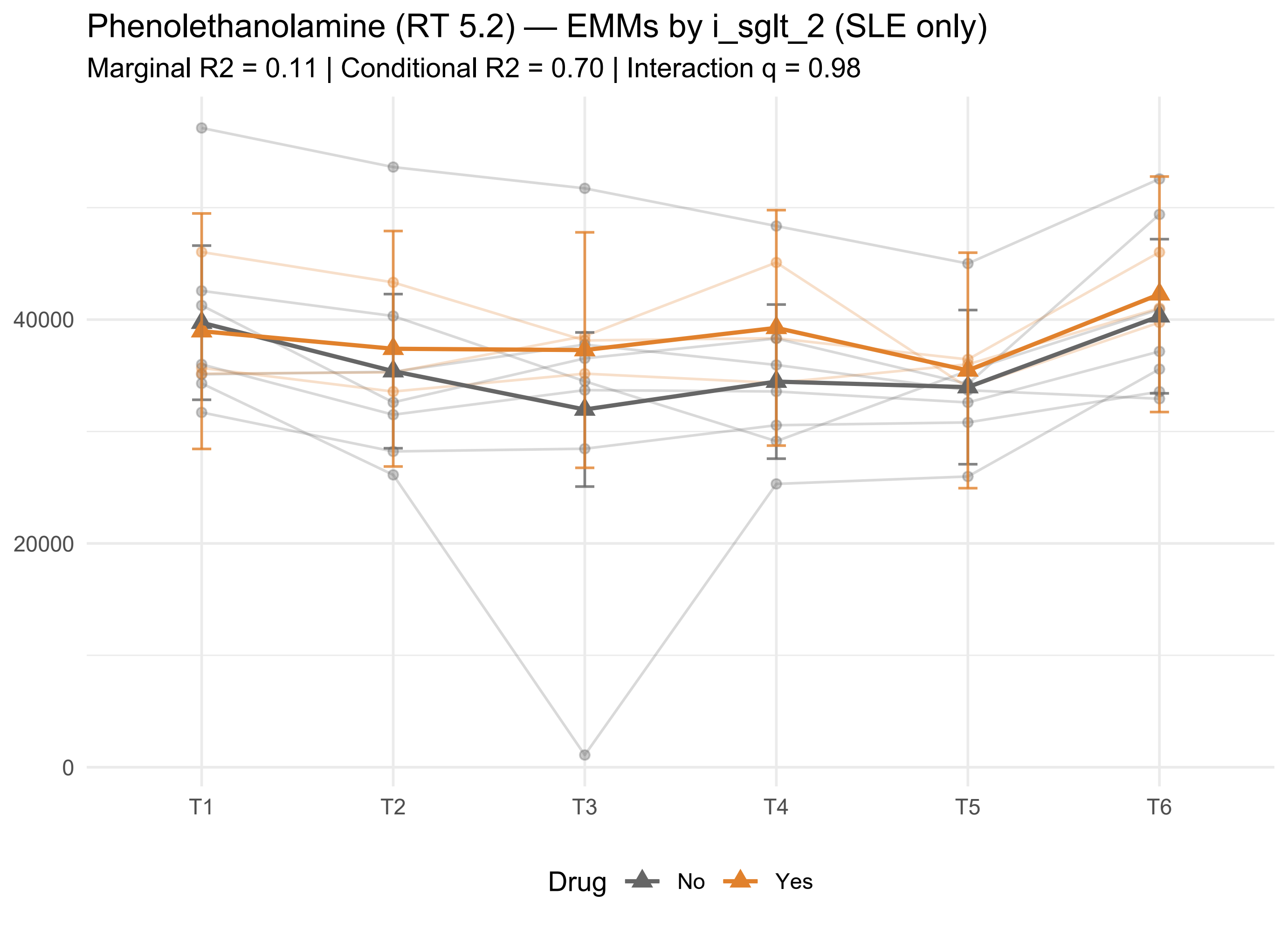
Phenolethanolamine (RT 5.2) — EMMs by i_sglt_2 (SLE only)
Marginal R2 = 0.11 | Conditional R2 = 0.70 | Interaction q = 0.98
40000
20000
0
T1
T2
T3
T4
T5
T6
Drug
No
Yes

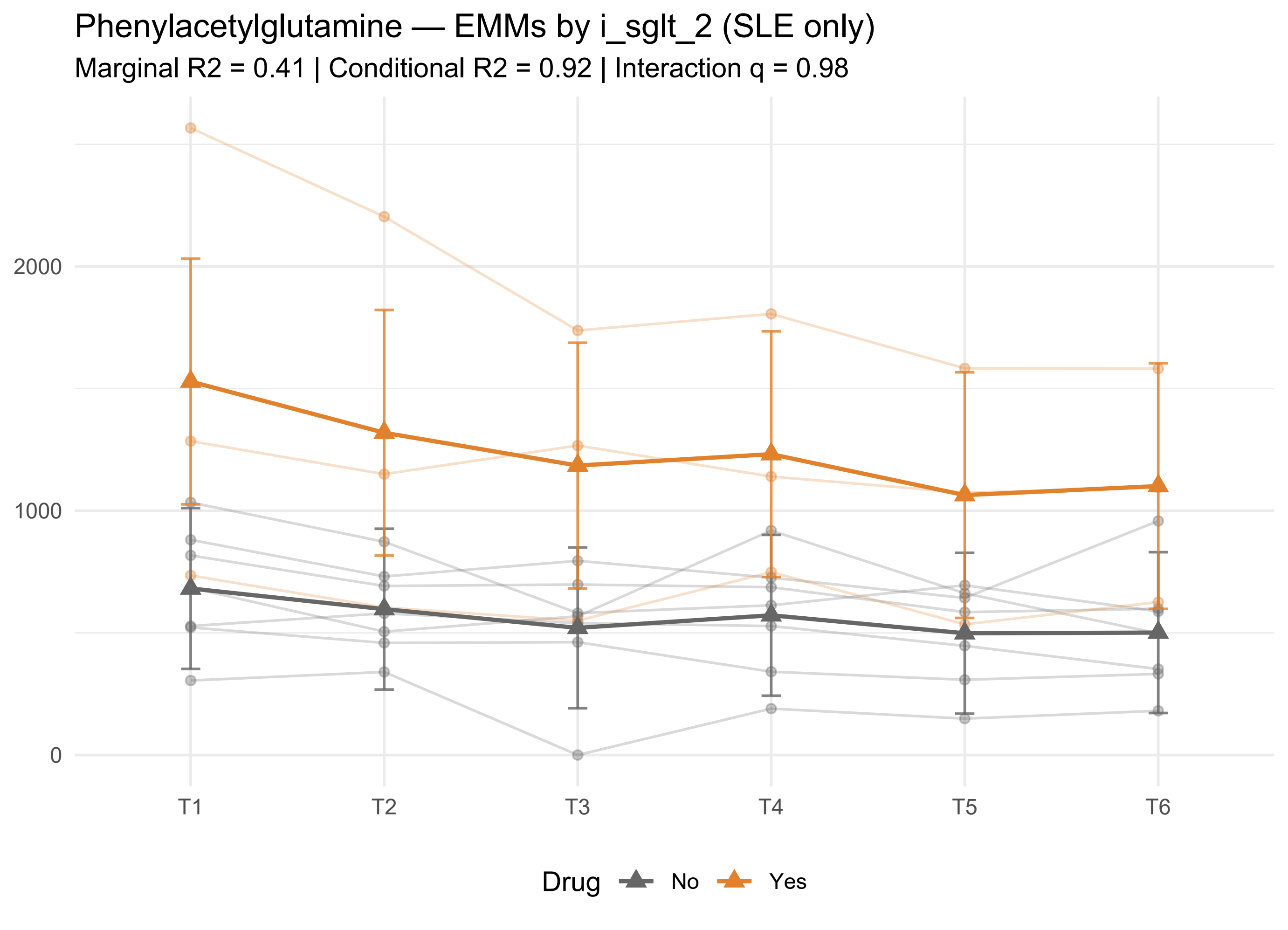

Phenylacetylglutamine — EMMs by i_sglt_2 (SLE only)
Marginal R2 = 0.41 | Conditional R2 = 0.92 | Interaction q = 0.98
2000
1000
0
T1
T2
T3
T4
T5
T6
Drug
No
Yes

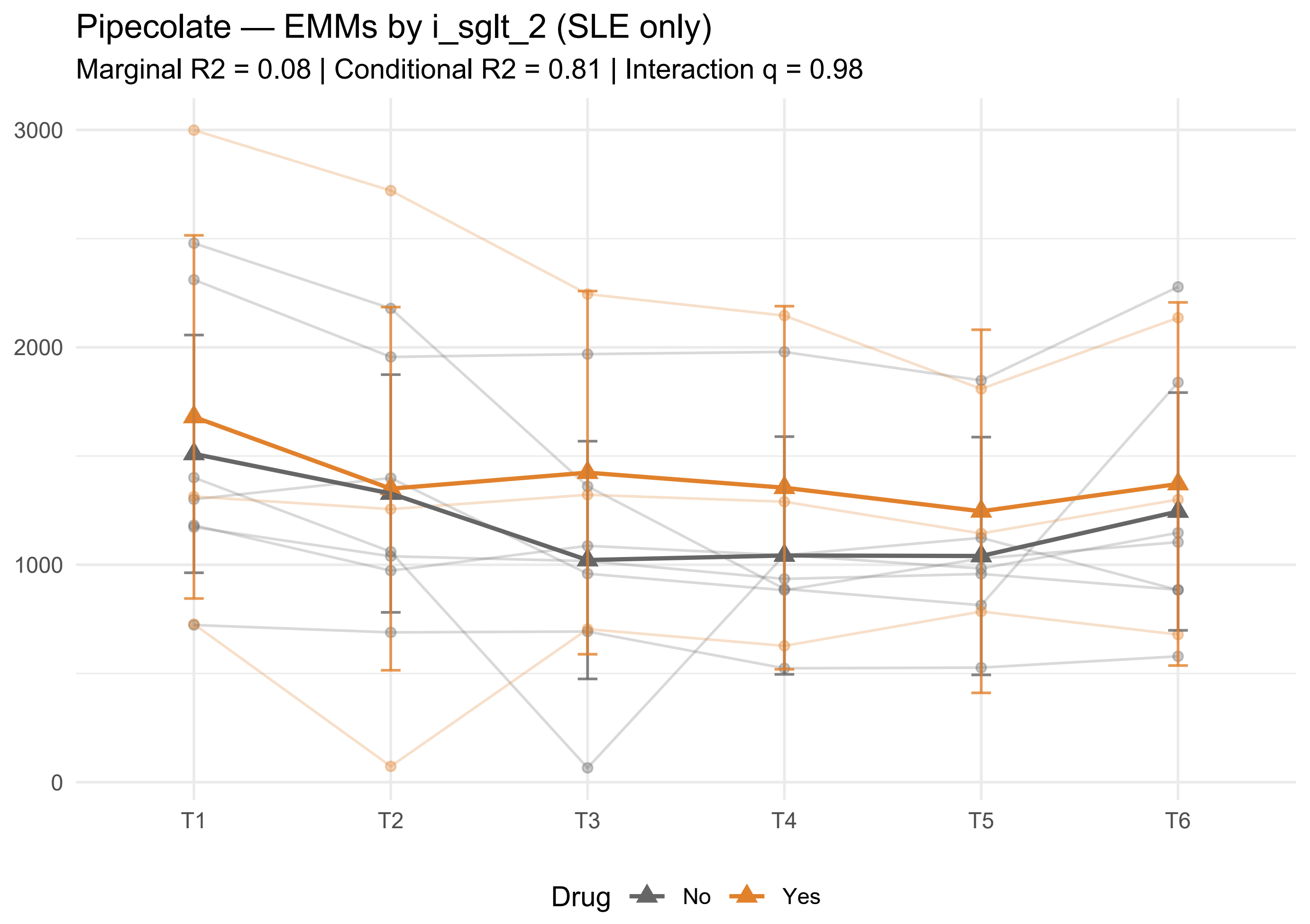

Pipecolate — EMMs by i_sglt_2 (SLE only)
Marginal R2 = 0.08 | Conditional R2 = 0.81 | Interaction q = 0.98
3000
2000
1000
0
T1
T2
T3
T4
T5
T6
Drug
No
Yes

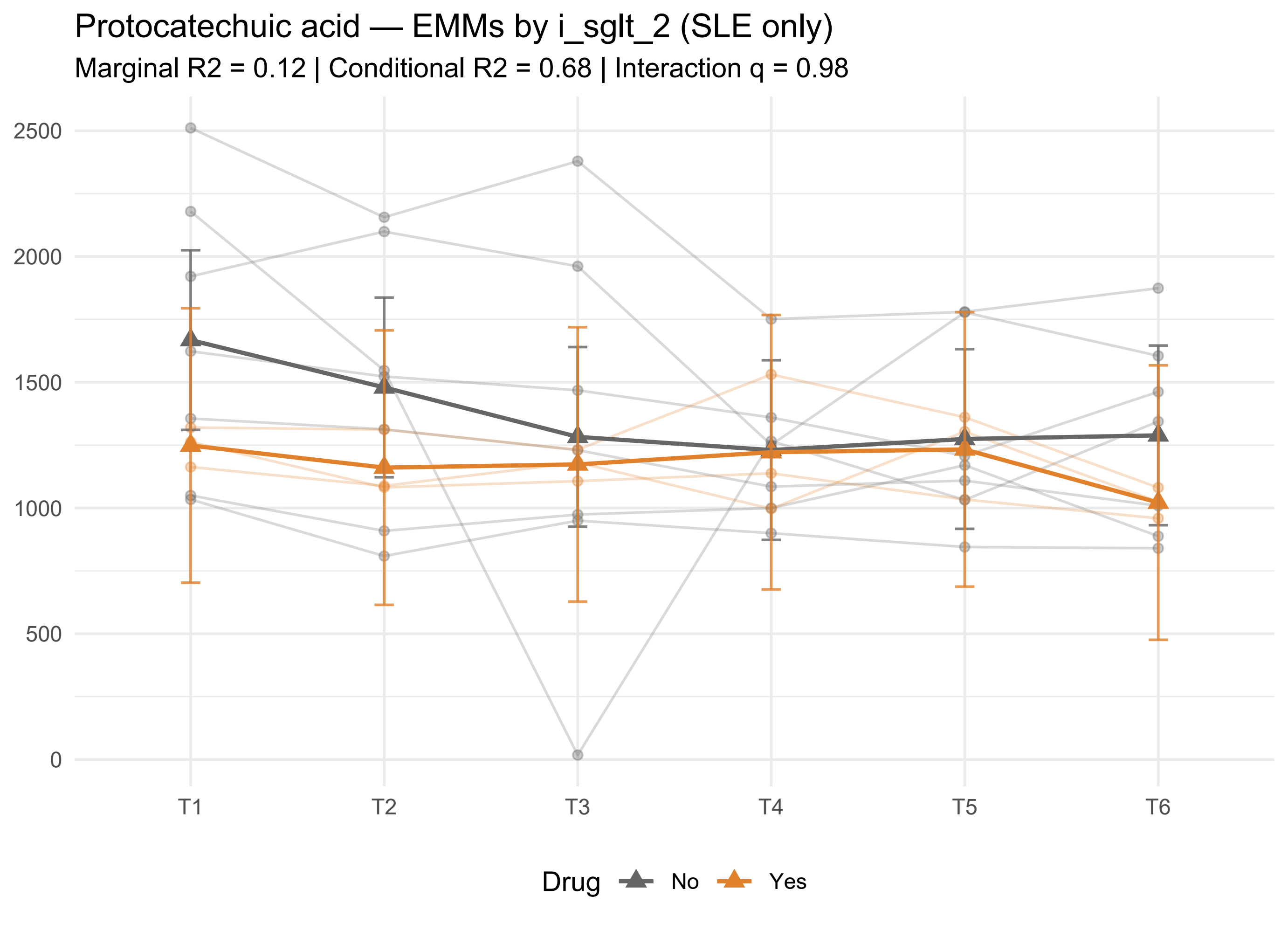
Protocatechuic acid — EMMs by i_sglt_2 (SLE only)
Marginal R2 = 0.12 | Conditional R2 = 0.68 | Interaction q = 0.98
2500
2000
1500
1000
500
0
T1
T2
T3
T4
T5
T6
Drug
No
Yes

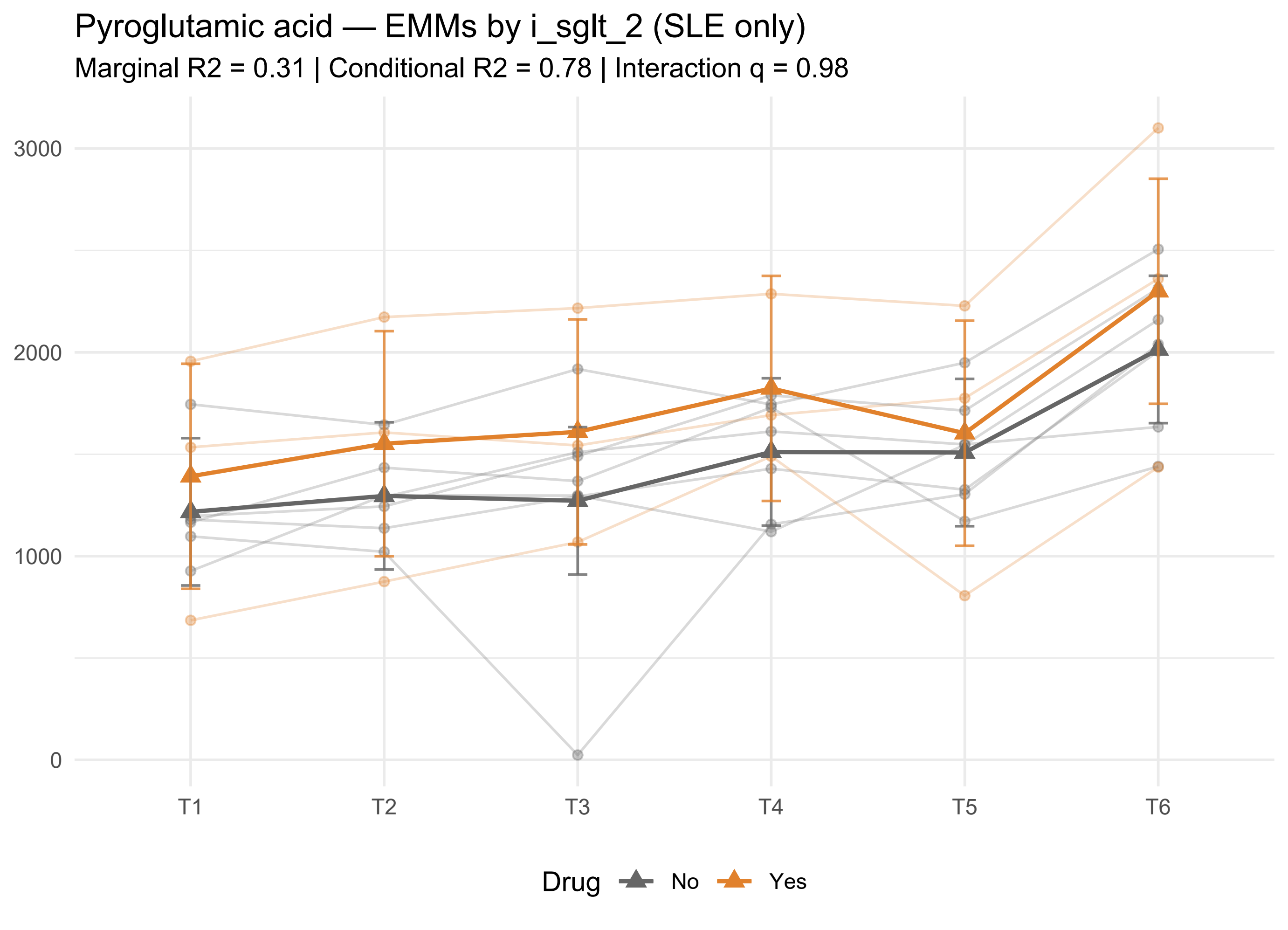

Pyroglutamic acid — EMMs by i_sglt_2 (SLE only)
Marginal R2 = 0.31 | Conditional R2 = 0.78 | Interaction q = 0.98
3000
2000
1000
0
T1
T2
T3
T4
T5
T6
Drug
No
Yes

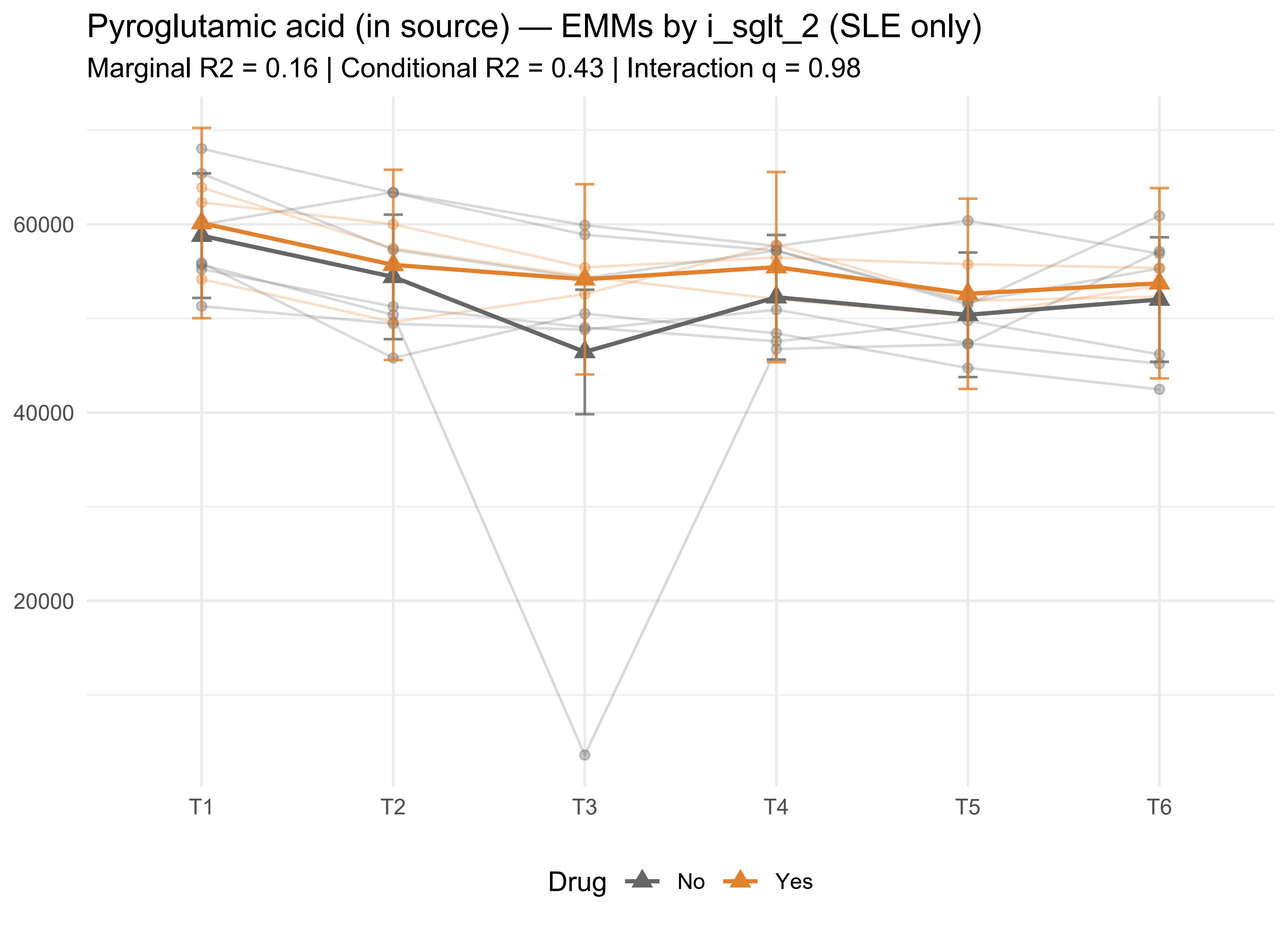

Pyroglutamic acid (in source) — EMMs by i_sglt_2 (SLE only)
Marginal R2 = 0.16 | Conditional R2 = 0.43 | Interaction q = 0.98
60000
40000
20000
T1
T2
T3
T4
T5
T6
Drug
No
Yes

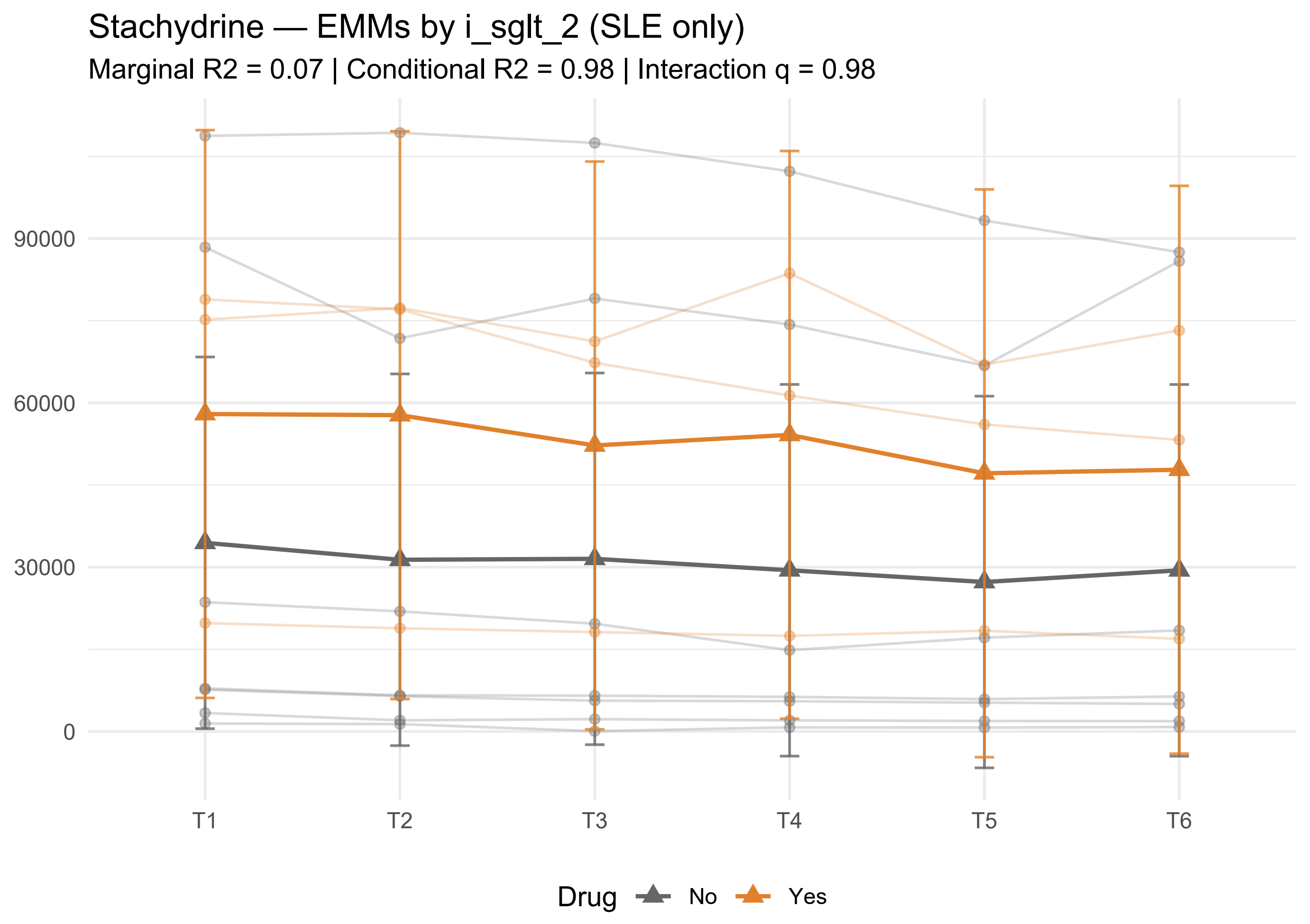

Stachydrine — EMMs by i_sglt_2 (SLE only)
Marginal R2 = 0.07 | Conditional R2 = 0.98 | Interaction q = 0.98
90000
60000
30000
0
T1
T2
T3
T4
T5
T6
Drug
No
Yes

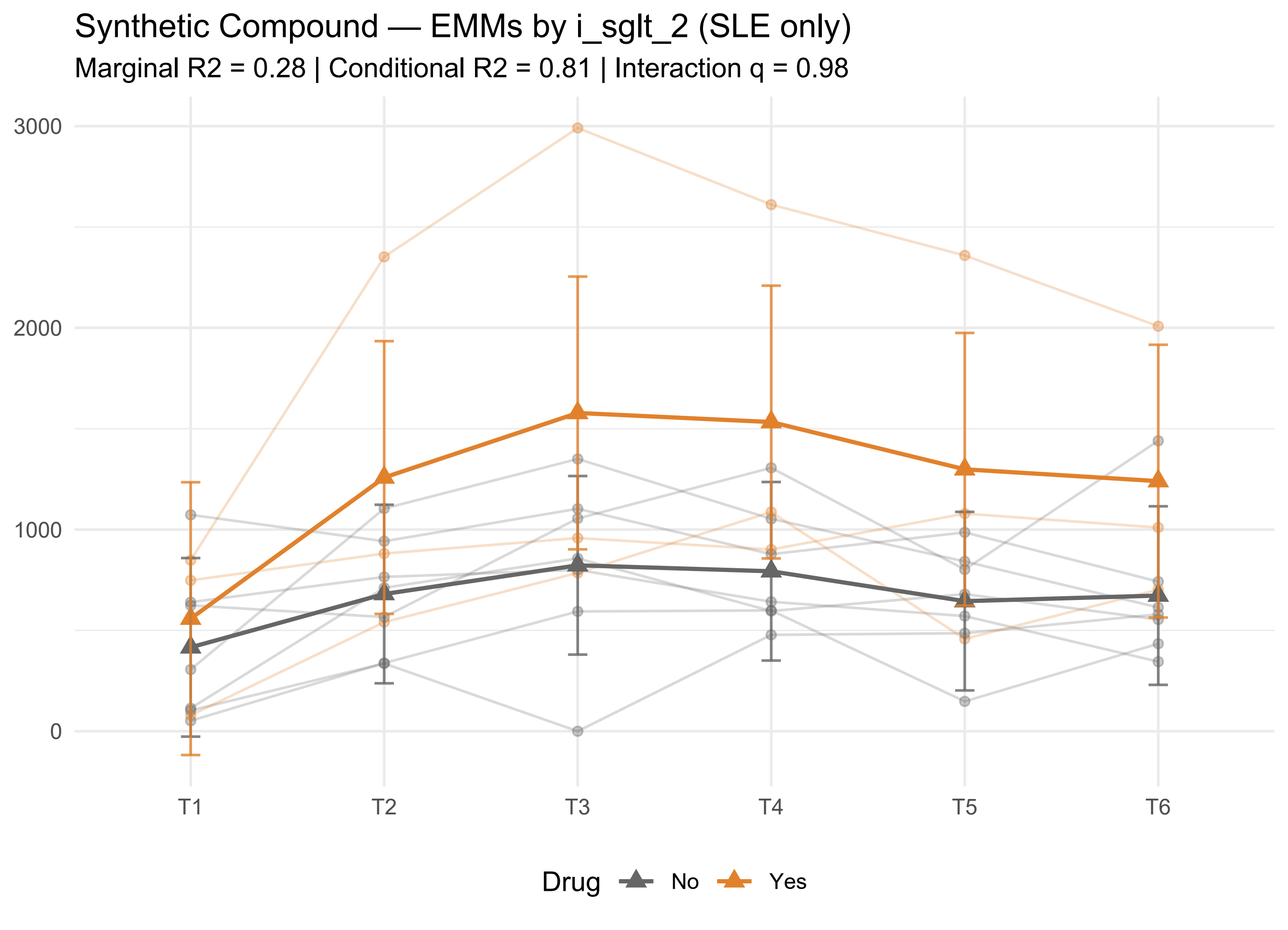

Synthetic Compound — EMMs by i_sglt_2 (SLE only)
Marginal R2 = 0.28 | Conditional R2 = 0.81 | Interaction q = 0.98
3000
2000
1000
0
T1
T2
T3
T4
T5
T6
Drug
No
Yes

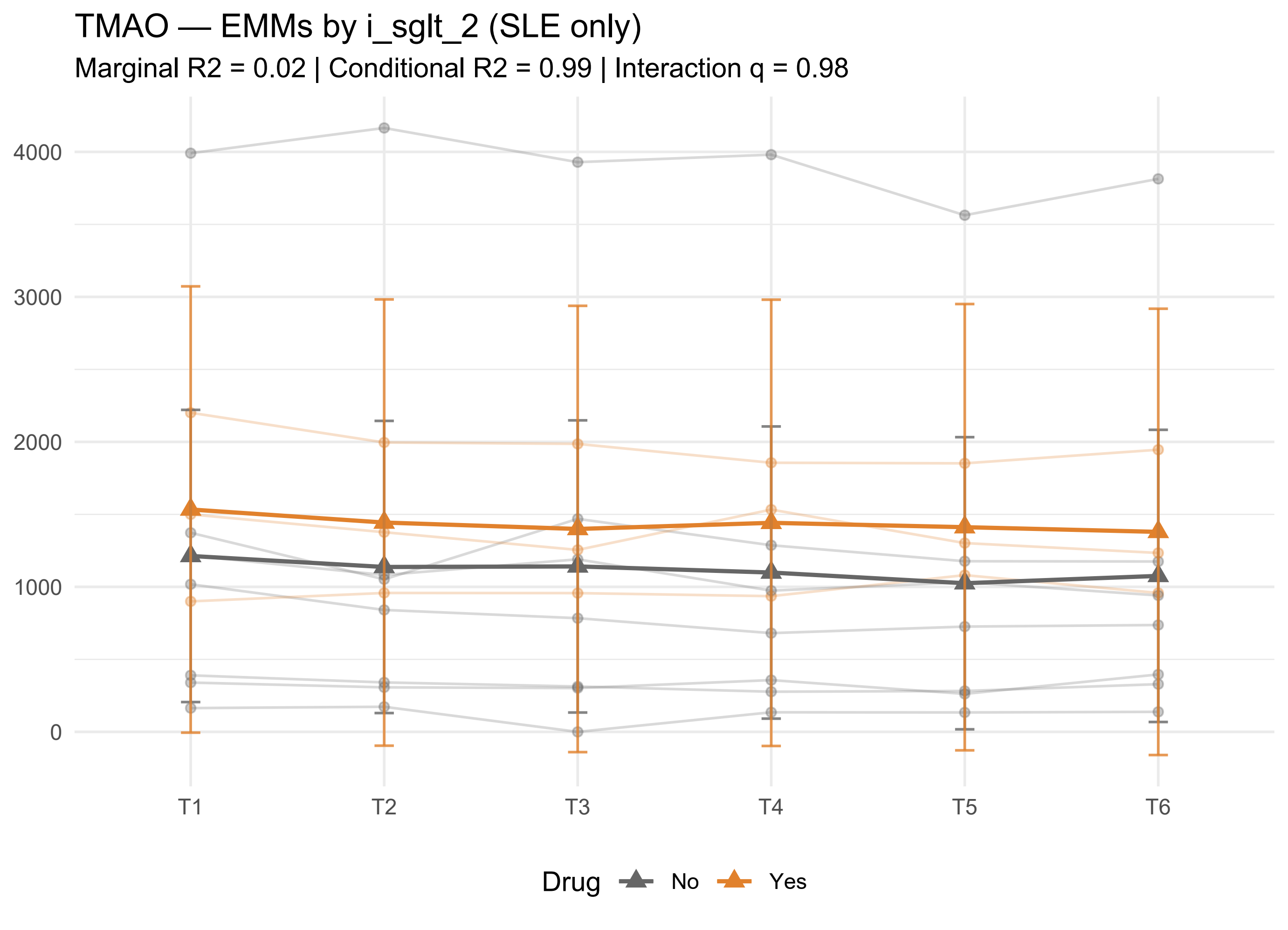

TMAO — EMMs by i_sglt_2 (SLE only)
Marginal R2 = 0.02 | Conditional R2 = 0.99 | Interaction q = 0.98
4000
3000
2000
1000
0
T1
T2
T3
T4
T5
T6
Drug
No
Yes

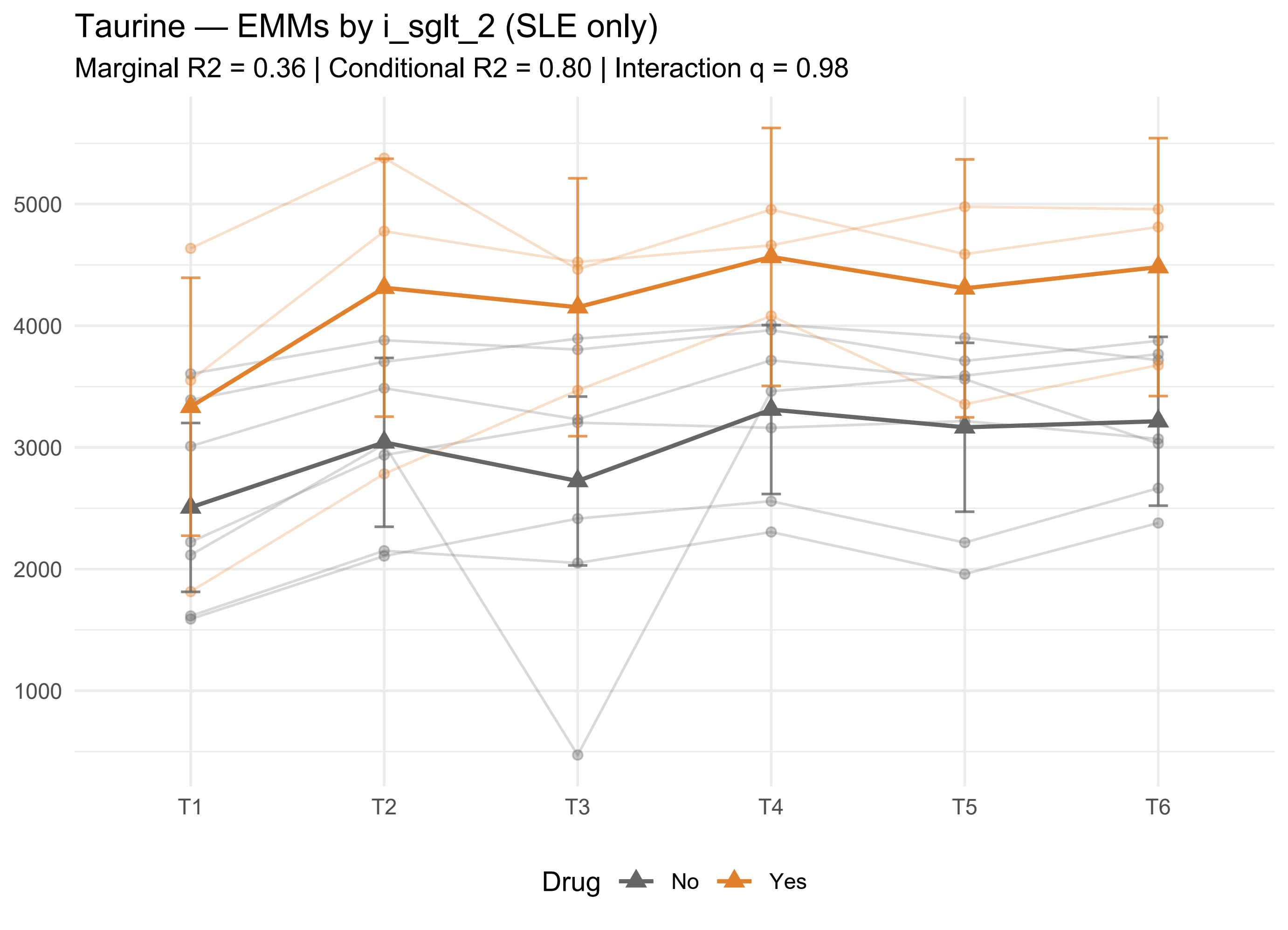

Taurine — EMMs by i_sglt_2 (SLE only)
Marginal R2 = 0.36 | Conditional R2 = 0.80 | Interaction q = 0.98
5000
4000
3000
2000
1000
T1
T2
T3
T4
T5
T6
Drug
No
Yes

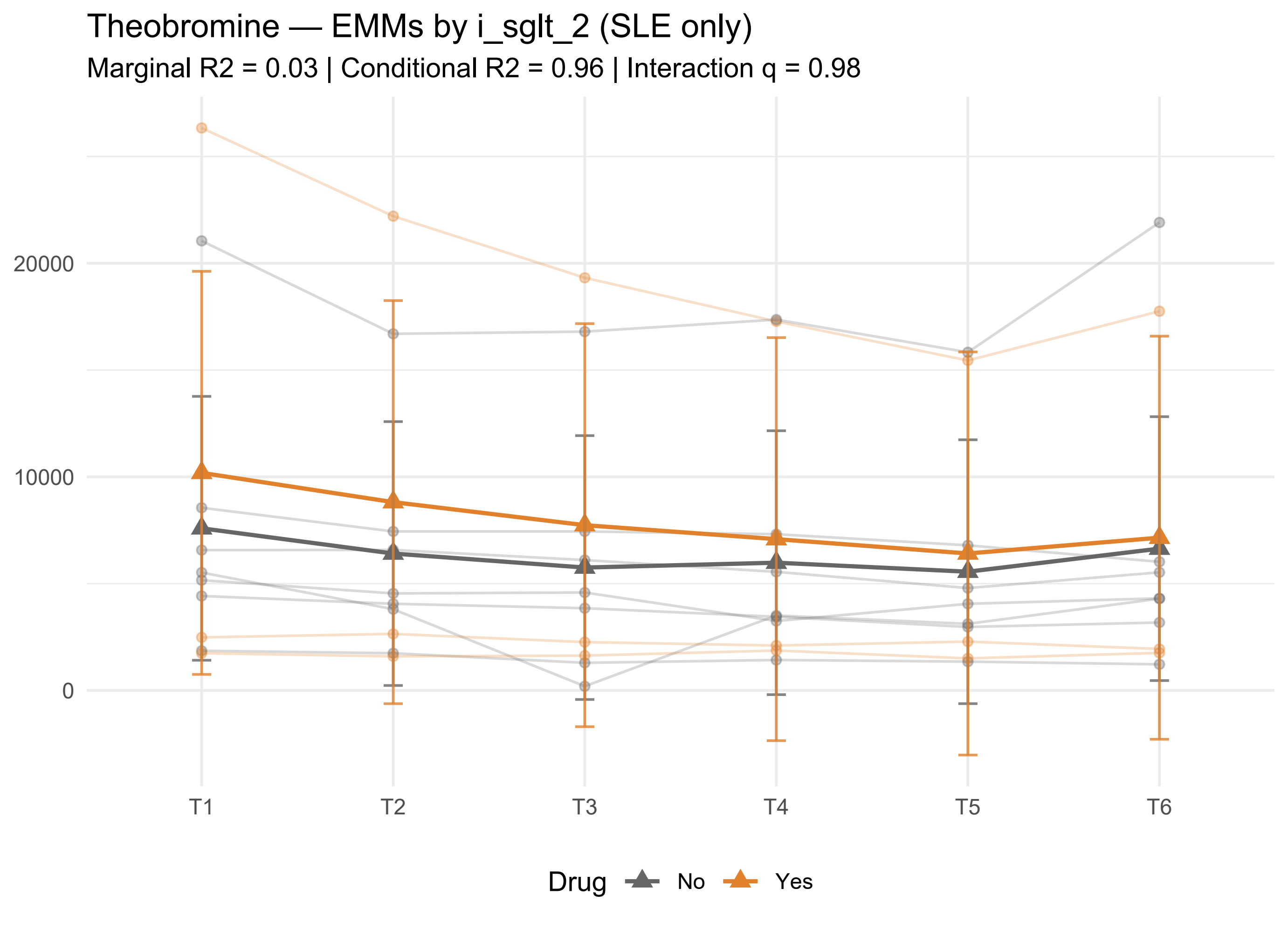
Theobromine — EMMs by i_sglt_2 (SLE only)
Marginal R2 = 0.03 | Conditional R2 = 0.96 | Interaction q = 0.98
20000
10000
0
T1
T2
T3
T4
T5
T6
Drug
No
Yes

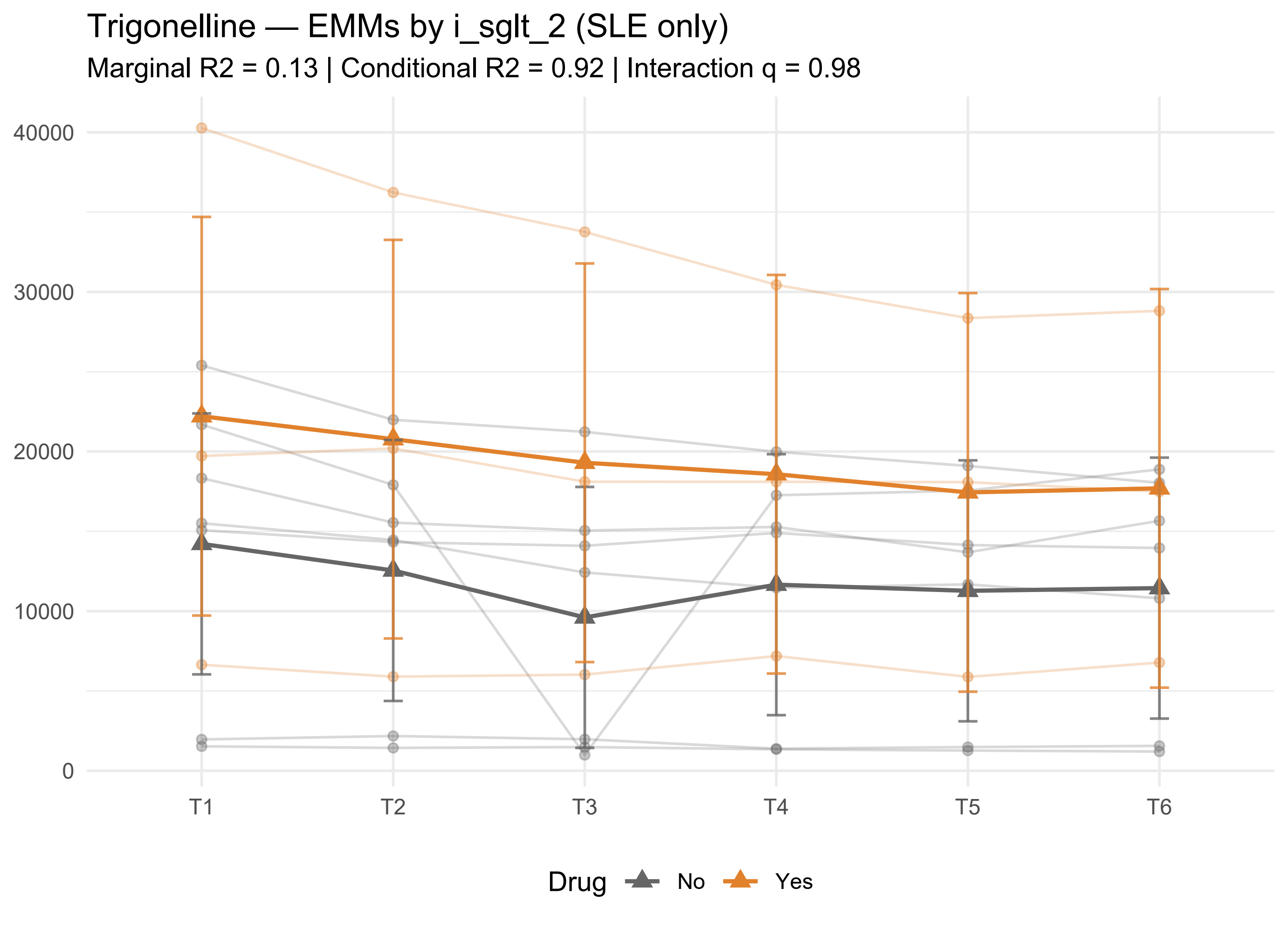

Trigonelline — EMMs by i_sglt_2 (SLE only)
Marginal R2 = 0.13 | Conditional R2 = 0.92 | Interaction q = 0.98
40000
30000
20000
10000
0
T1
T2
T3
T4
T5
T6
Drug
No
Yes

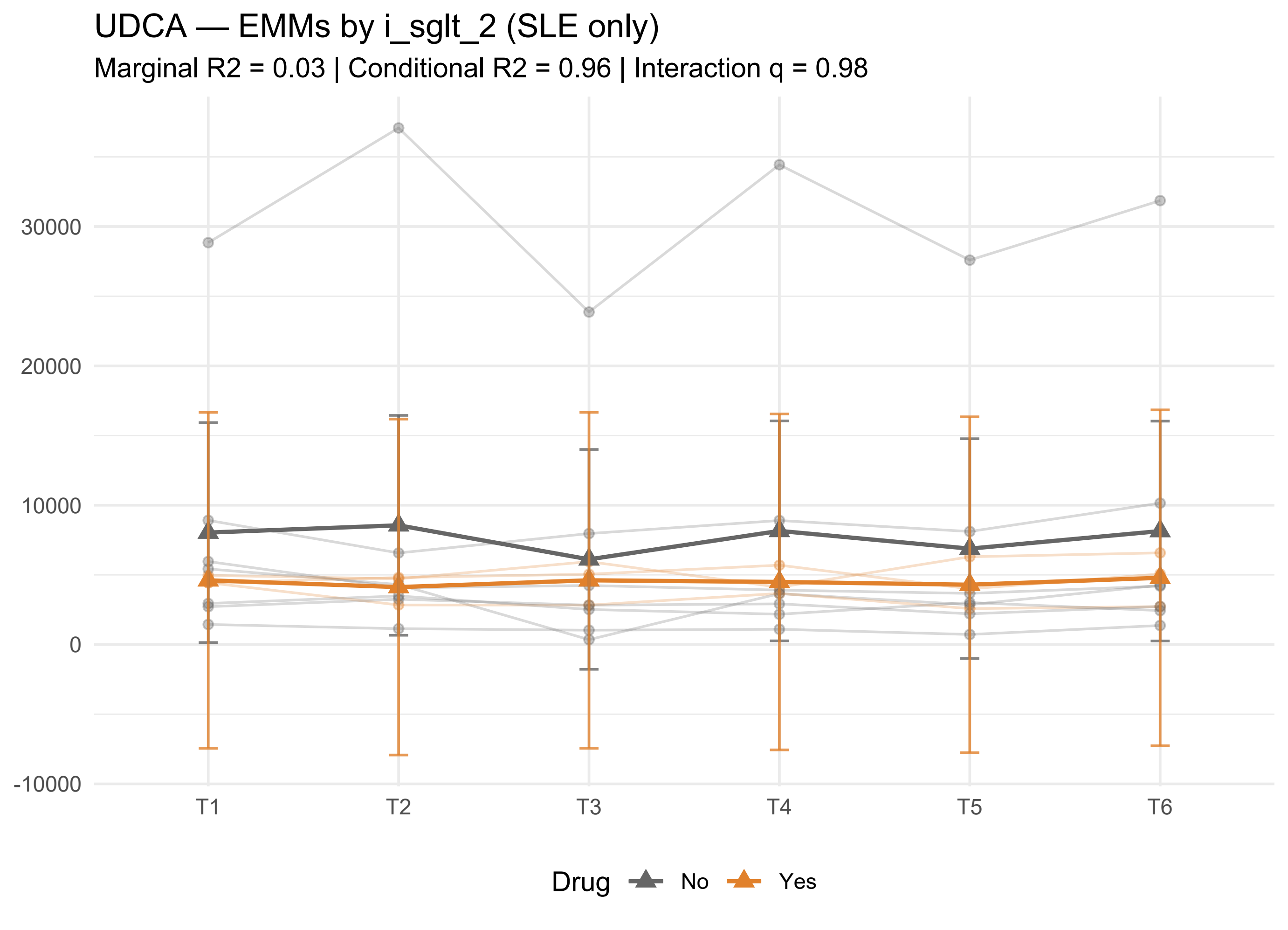

UDCA — EMMs by i_sglt_2 (SLE only)
Marginal R2 = 0.03 | Conditional R2 = 0.96 | Interaction q = 0.98
30000
20000
10000
0
-10000
T1
T2
T3
T4
T5
T6
Drug
No
Yes

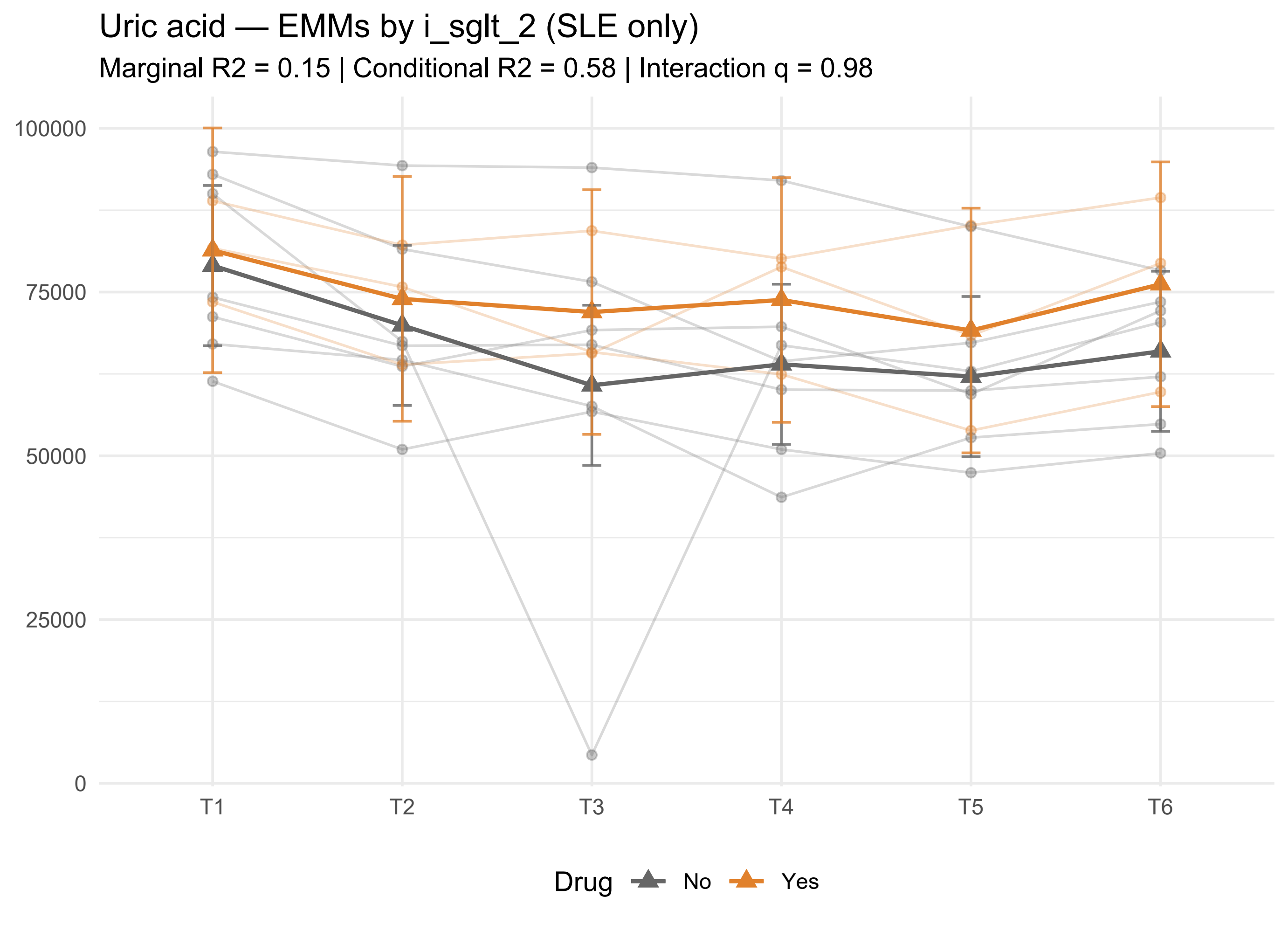
Uric acid — EMMs by i_sglt_2 (SLE only)
Marginal R2 = 0.15 | Conditional R2 = 0.58 | Interaction q = 0.98
100000
75000
50000
25000
0
T1
T2
T3
T4
T5
T6
Drug
No
Yes

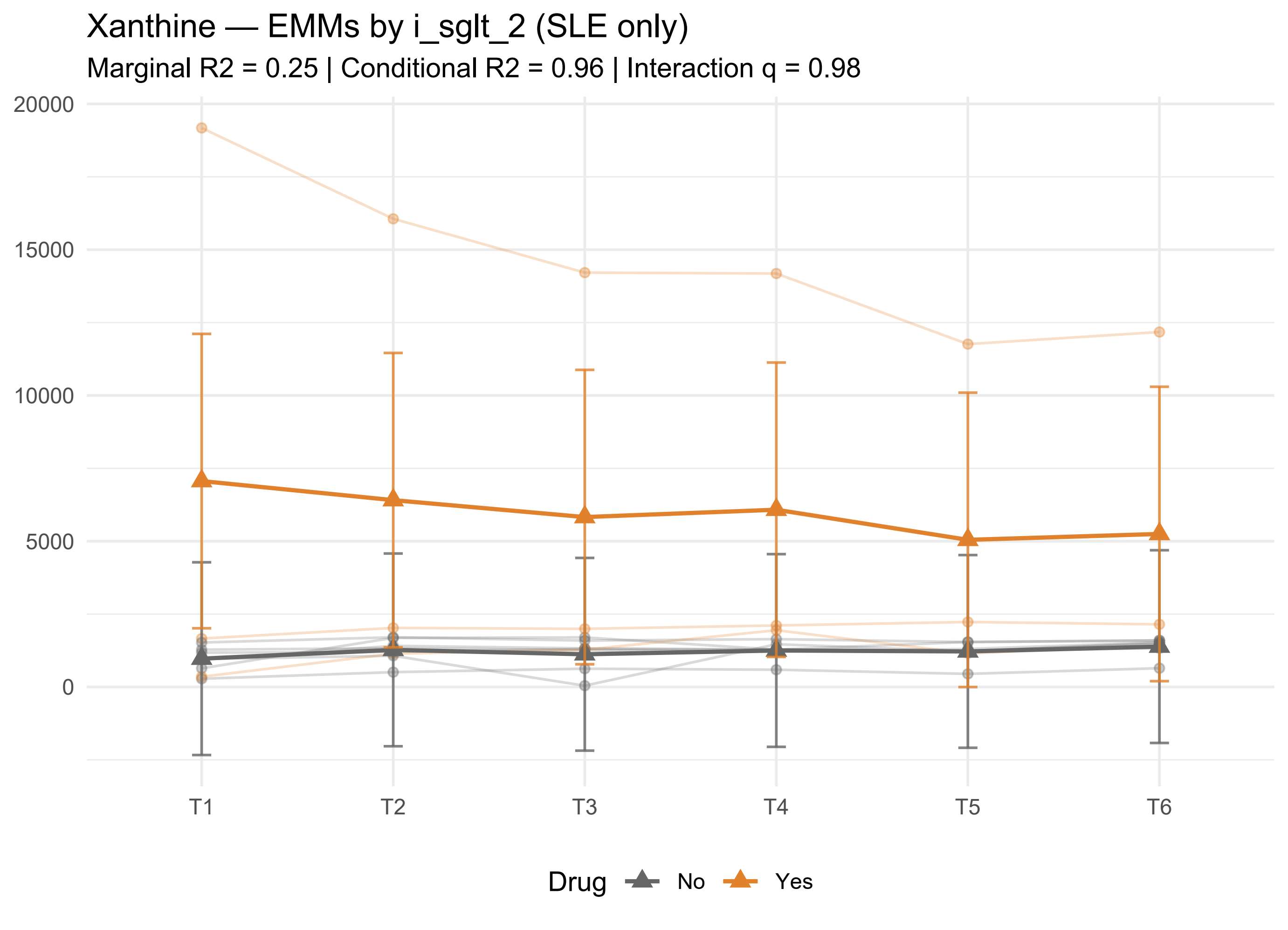
Xanthine — EMMs by i_sglt_2 (SLE only)
Marginal R2 = 0.25 | Conditional R2 = 0.96 | Interaction q = 0.98
20000
15000
10000
5000
0
T1
T2
T3
T4
T5
T6
Drug
No
Yes
