# Supplementary figures and images for: Disease and Medication Context Shape Ex Vivo Metabolite Stability: A Pilot Study in Systemic Lupus Erythematosus
Source: Metabolites. 2025 Nov 12;15(11):738. doi: 10.3390/metabo15110738 (PMC12654355; doi:10.3390/metabo15110738)

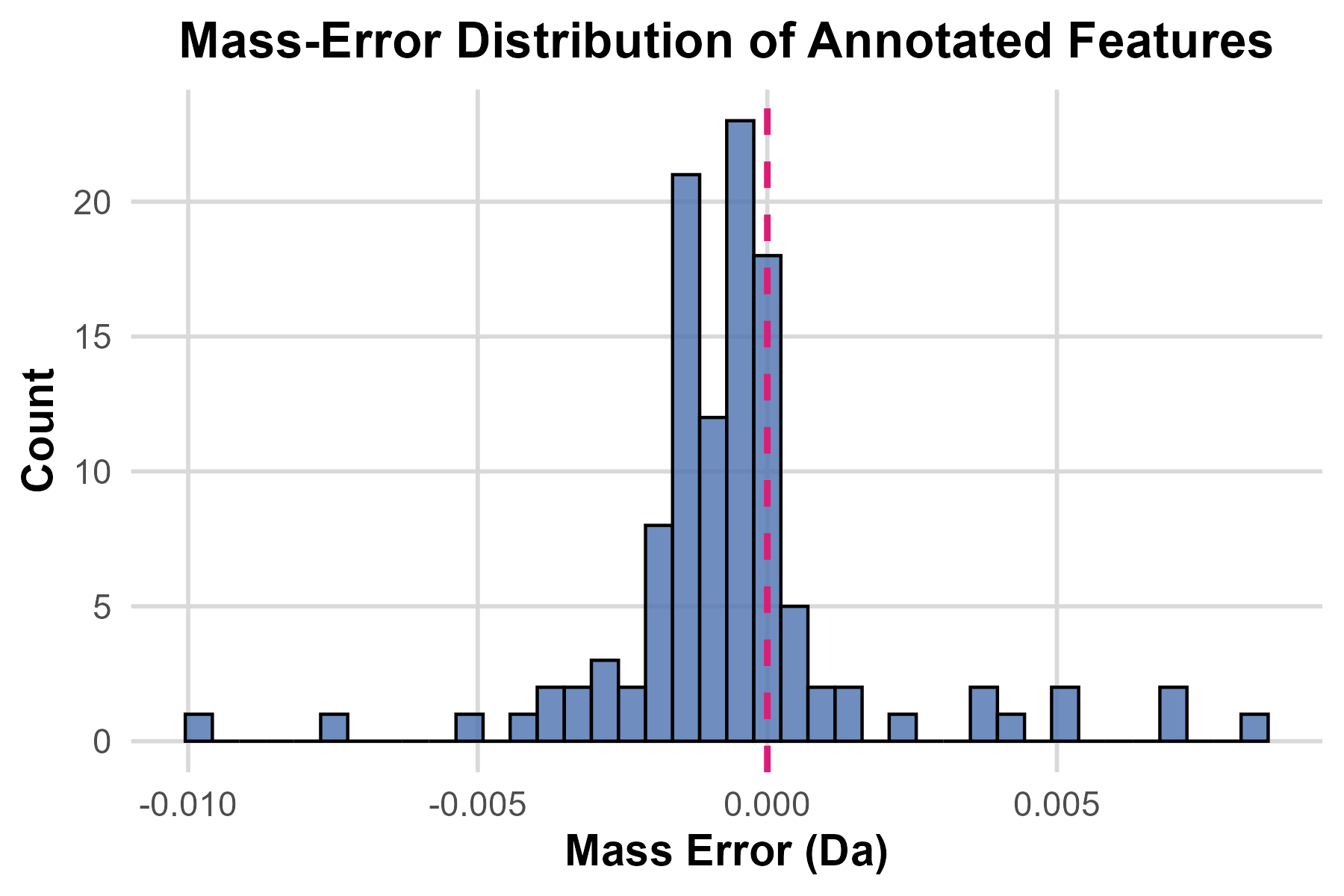

Supplement: Supplementary file 1 [file metabolites-15-00738-s001.zip › Supplementary Figure S1.png]

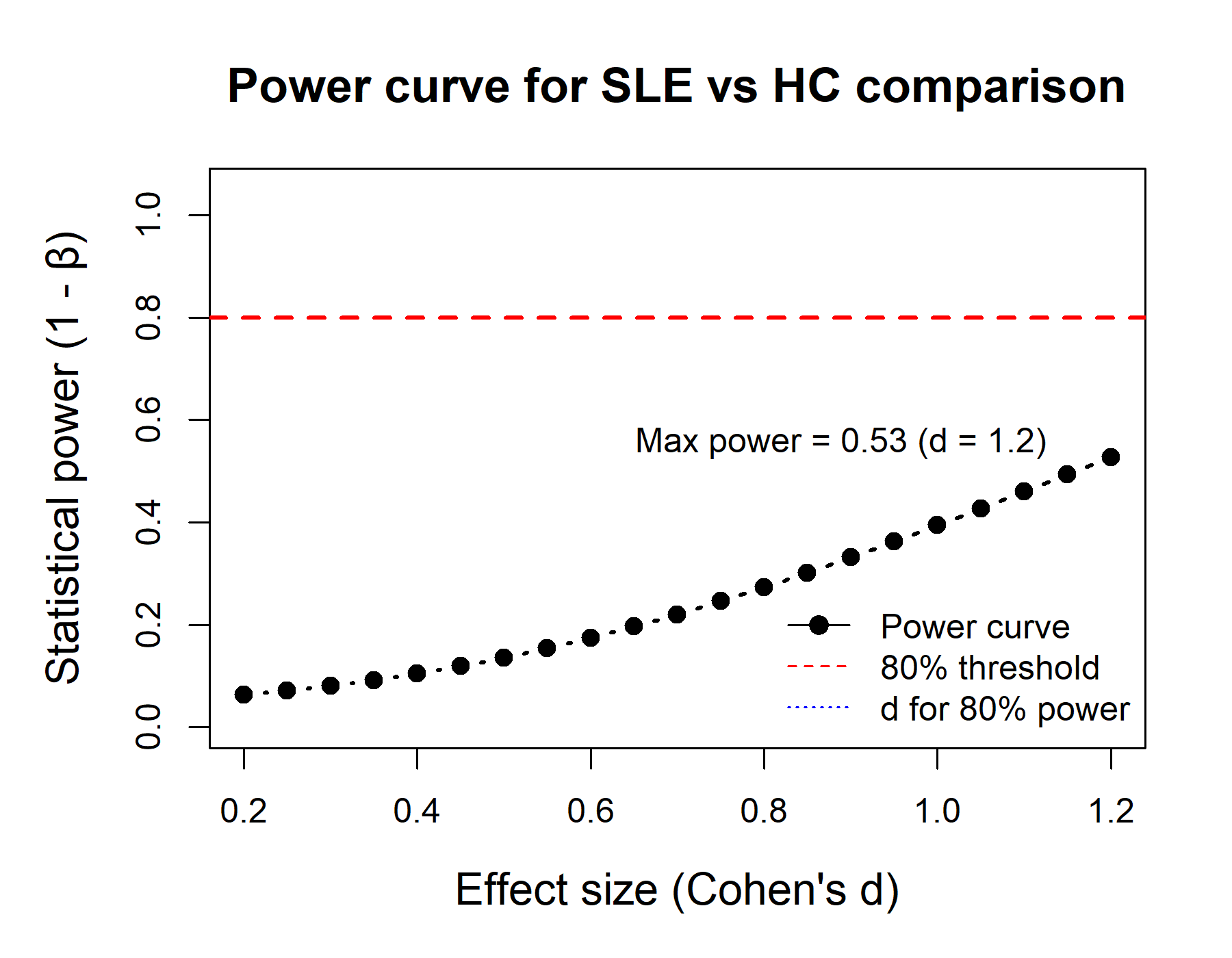

Supplement: Supplementary file 1 [file metabolites-15-00738-s001.zip › Supplementary Figure S2.png]

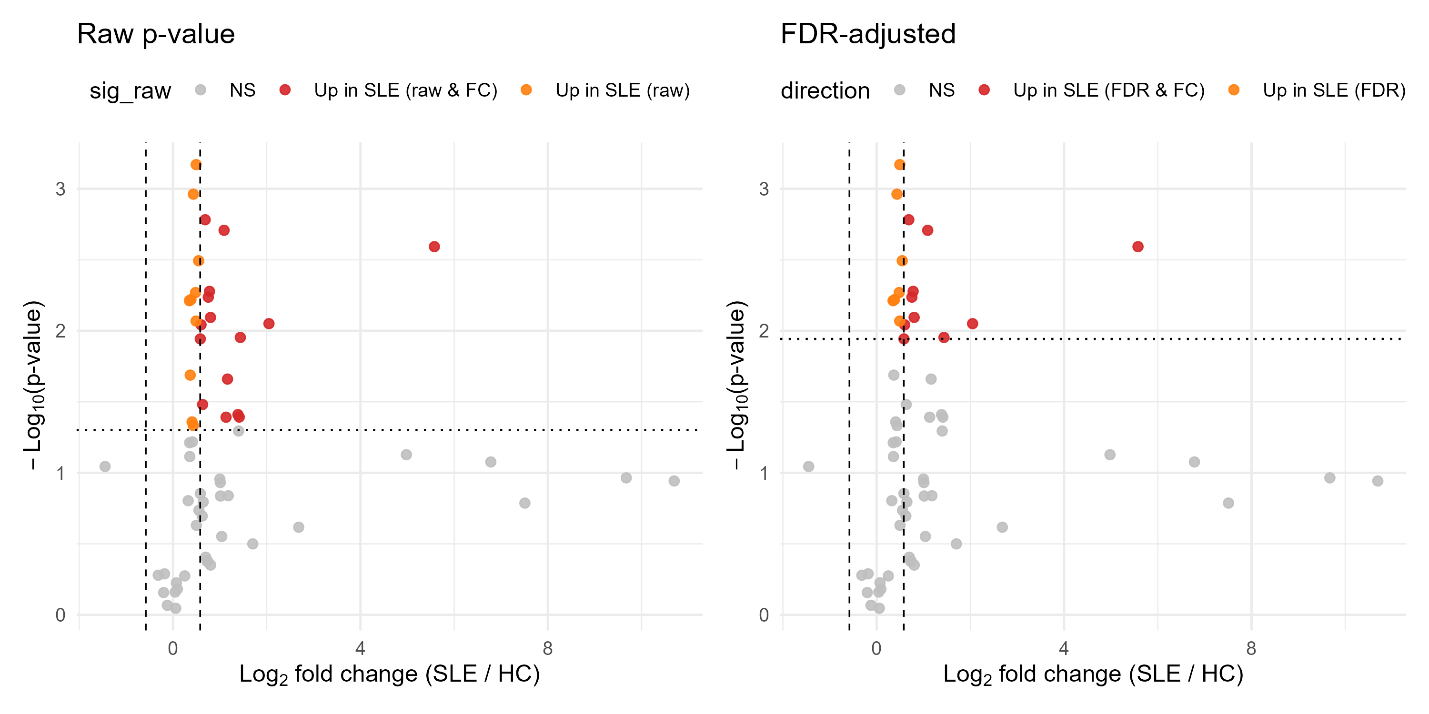

Supplement: Supplementary file 1 [file metabolites-15-00738-s001.zip › Supplementary Figure S3.png]

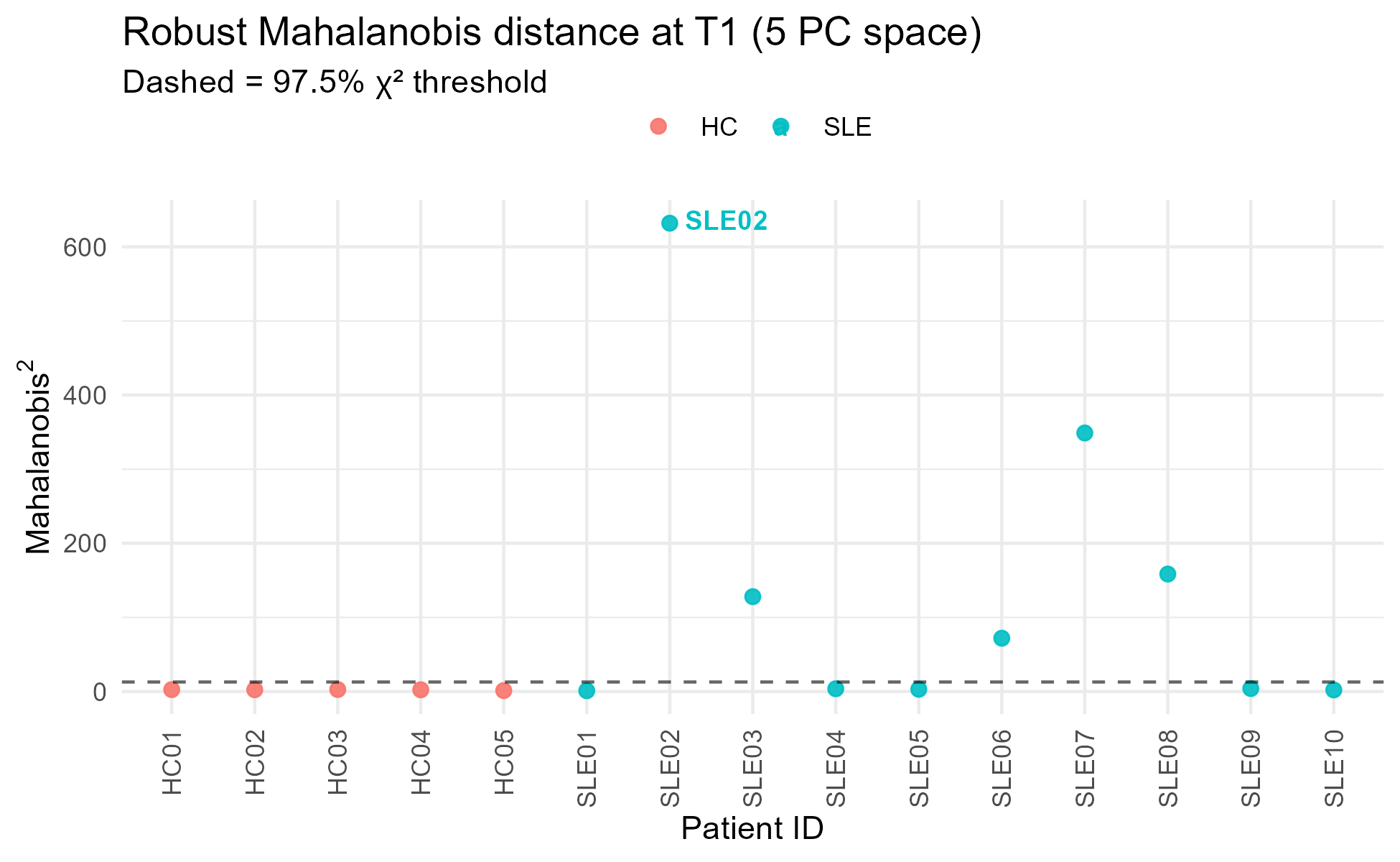

Supplement: Supplementary file 1 [file metabolites-15-00738-s001.zip › Supplementary Figure S4.png]

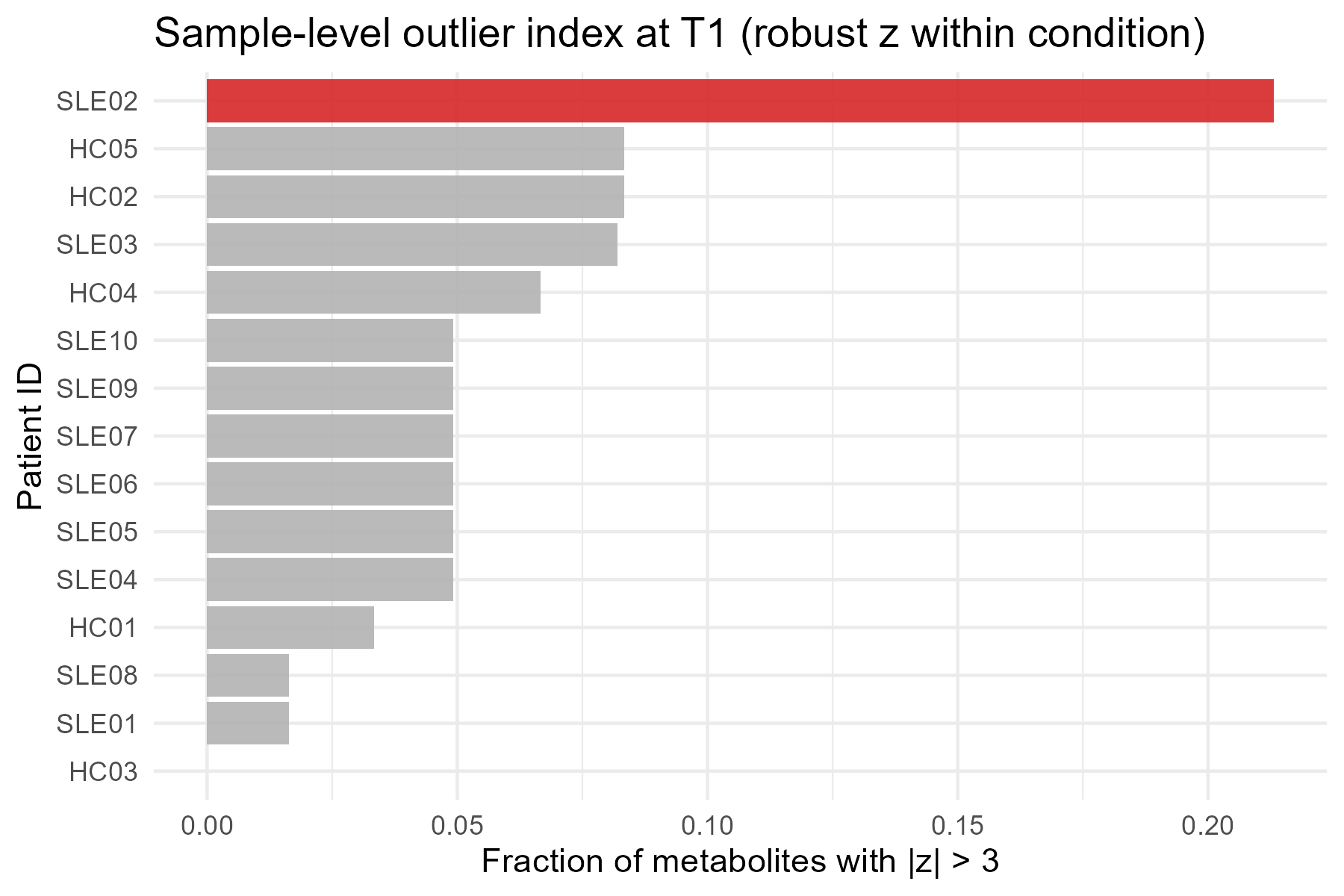

Supplement: Supplementary file 1 [file metabolites-15-00738-s001.zip › Supplementary Figure S5.png]

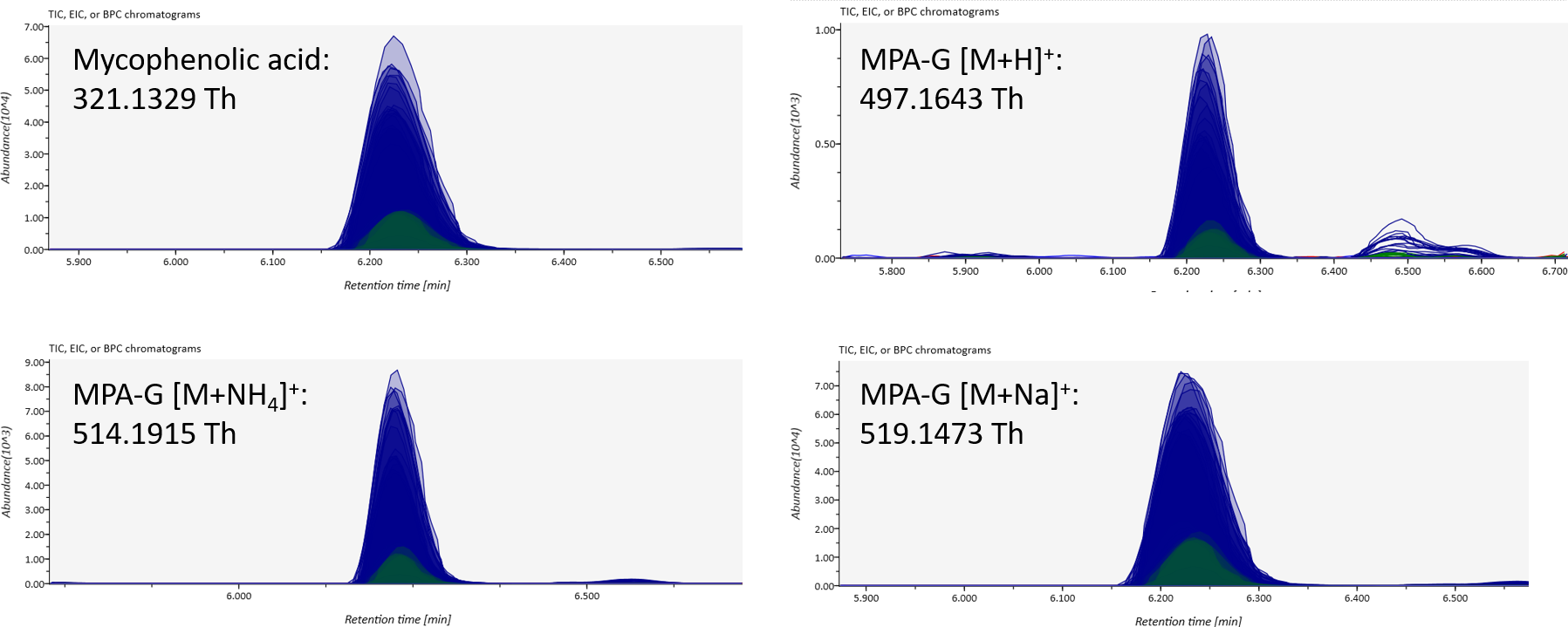

Supplement: Supplementary file 1 [file metabolites-15-00738-s001.zip › Supplementary Figure S8.png]

# Time Courses by Condition – Inosine

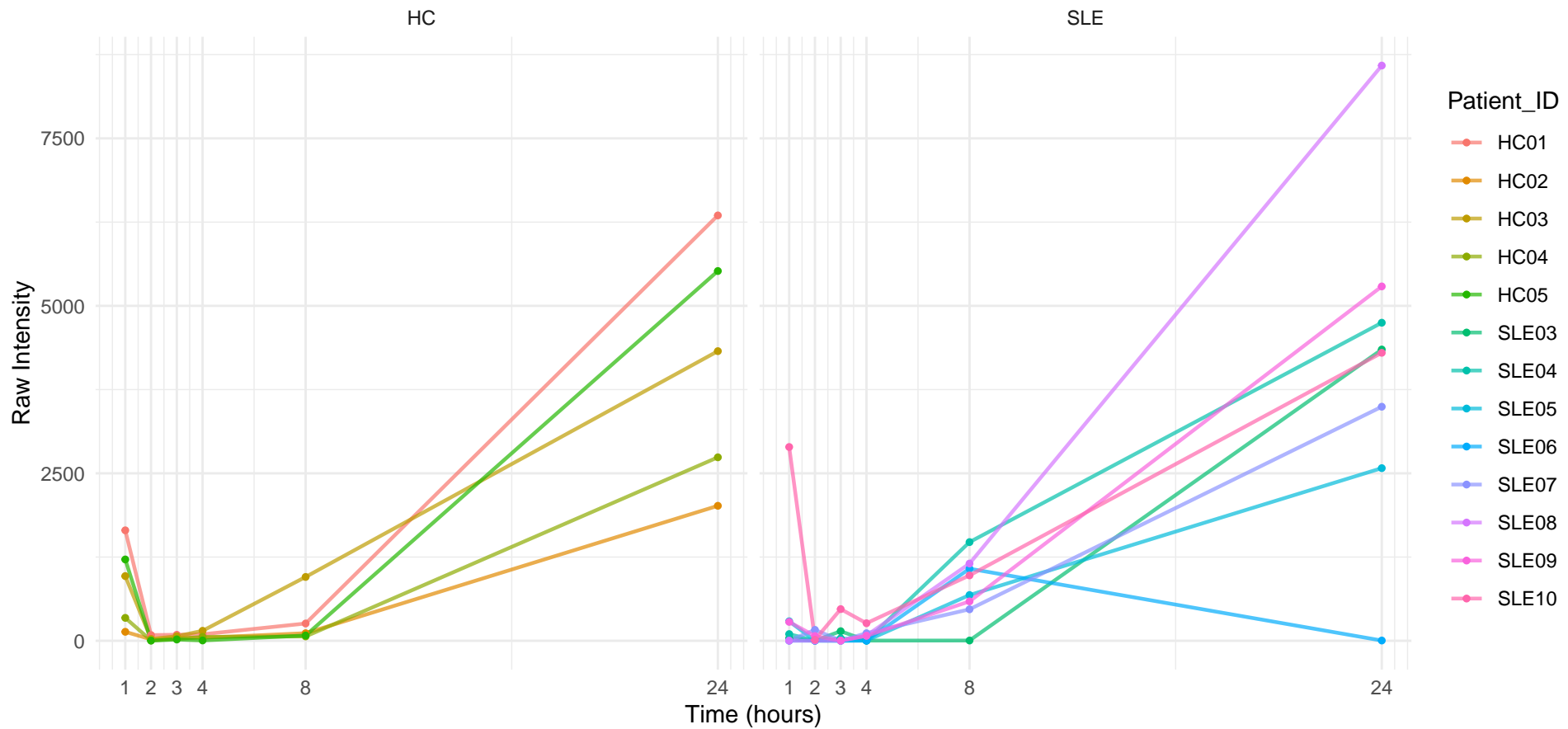

Supplement: Supplementary file 1 [file metabolites-15-00738-s001.zip › Supplementary Figure S9.pdf]
